# A Photoredox C–H Arene Functionalization toward Substituted Methyls

**DOI:** 10.1021/jacs.6c04756

**Published:** 2026-07-10

**Authors:** Jonas Žurauskas, Nojus Radzevičius, Paulius Vaicku̅nas, Gabija Sergejevaitė, Edvinas Orentas

**Affiliations:** Department of Organic Chemistry, 54694 Vilnius University, Naugarduko 24, LT-03225 Vilnius, Lithuania

## Abstract

The ability to introduce modular C1 units directly into arene C–H bonds remains a fundamental challenge in synthetic design, limiting access to highly diversified molecular architectures from simple feedstocks. We disclose a photoredox-neutral platform that enables direct transfer of a methylene pyridinium linchpin to unactivated arenes, transforming inert C–H sites into versatile synthetic handles. The resulting benzylpyridinium products function as broadly addressable intermediates, supporting a wide range of C–C and C–heteroatom bond formation and granting streamlined access to medicinally privileged substituted methyls.

## Graphical abstract

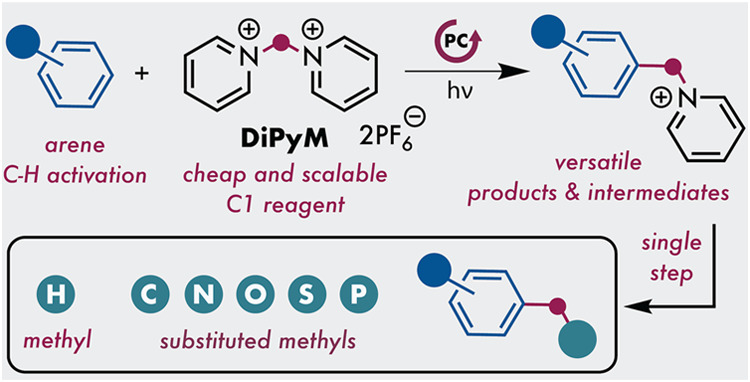

## Introduction

New methods to transform arene C–H bonds into functional handles that go beyond classical electrophilic substitution have the potential to reshape modern synthetic logic, enabling rapid access to structural diversity from simple feedstocks.^1^ While several methodologies now allow direct C­(sp^2^)–H functionalization of arenes, most transformations culminate in a single, static functional group, providing limited possibilities for downstream molecular elaboration. This restriction is particularly acute in medicinal chemistry and late-stage diversification, where a single C–H activation event ideally would serve as a branching node capable of giving rise to many distinct analogs.^2^ The concept of C1 linchpin installation, *i*.*e*., the introduction of a one-carbon fragment that can be flexibly interconverted into pharmaceutically relevant (un)­substituted methyls,^3,4^ offers an attractive solution but remains underdeveloped for unactivated arenes (Figure 1A).

**Figure 1:**
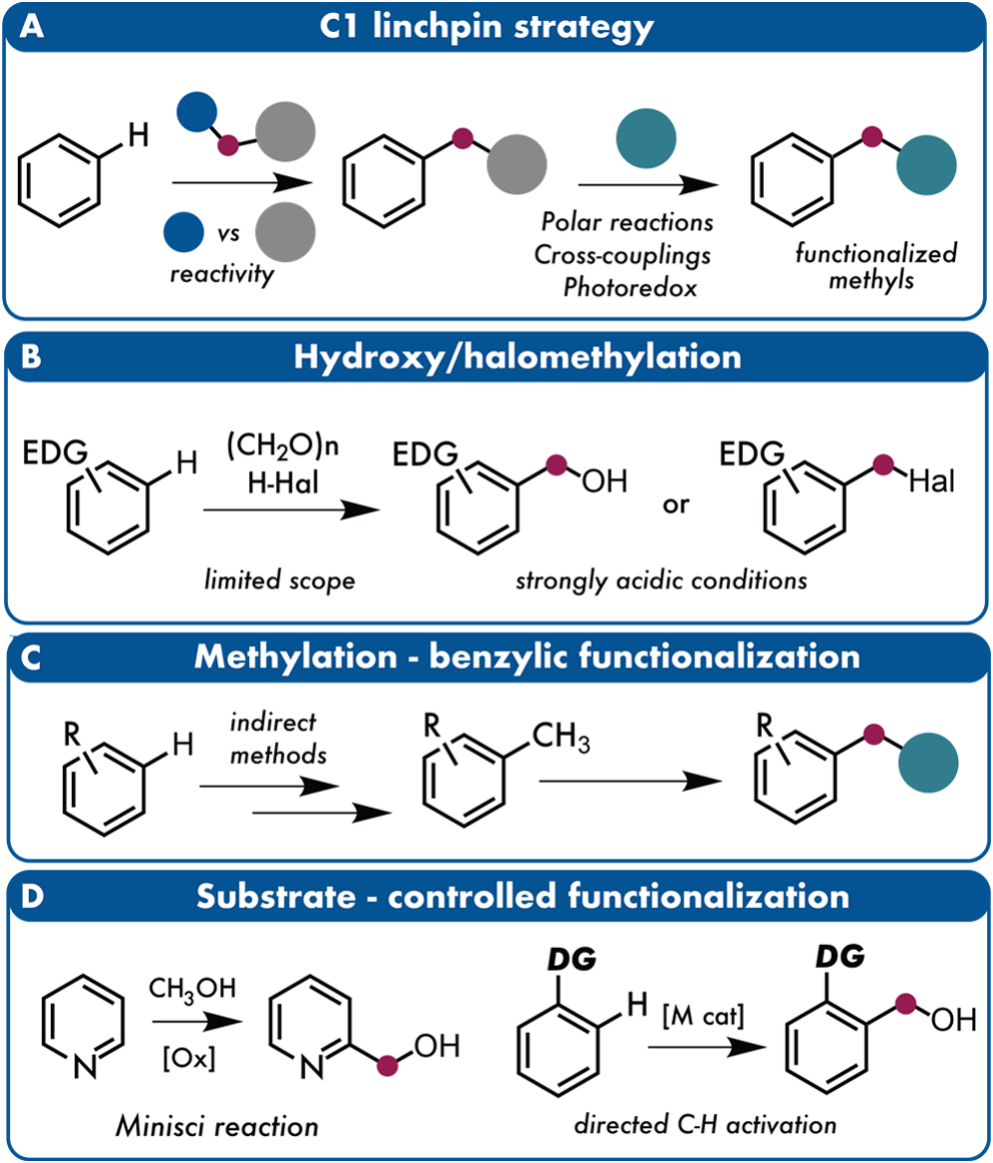
(a) Generic scheme for using the C1 linchpin for consecutive C–H activation and functionalization. (b–d) Commonly utilized C–H arene activation methods toward substituted methyls.

Photoredox catalysis provides an attractive entry point for such functionalization.^5^ Visible-light-mediated single-electron transfer enables the generation of open-shell reagents under mild conditions and allows their interception by unactivated aromatic π-systems with selectivity patterns potentially distinct from classical electrophilic substitution. By merging radical generation with controlled rearomatization, photoredox manifolds can forge C–C bonds directly from arene C–H bonds with bifunctional reagents preserving opportunities for modular postfunctionalization. Despite this potential, a general photoredox strategy for C1 installation into unactivated arenes has not yet been realized.^6^

Hydroxymethylation^7^ and halomethylation (the Blanc reaction)^8^ are examples of the few C1-based synthetic handles that can be directly introduced into aromatic rings. These classical reactions, proceeding through electrophilic substitution, necessitate electron-rich substrates and acidic reaction conditions, and are further complicated by the competing condensation reactions (Figure 1B). Likewise, arene formylation, such as Vilsmeier–Haack,^9^ suffers from very limited scope. Methylation, while often treated as a terminal transformation, can act as a launching point for benzylic halogenation or oxidation to indirectly yield diverse C1 derivatives (Figure 1C). However, controlled methylation constitutes a challenge in its own right and often is accomplished indirectly, in more than one synthetic step.^10^ On the contrary, Minisci-type hydroxymethylation^11^ or formylation^12^ of pyridine and related heteroaromatic substrates represent a well-established synthetic procedures; the chemistry, however, has a heteroarene-limited scope. Similarly, directed transition-metal-catalyzed C–H C1 functionalizations require prefunctionalized substrates, and forcing conditions that not only narrow the reaction scope but also diminish the step-economy advantages that C–H activation seeks to deliver (Figure 1D).^13^

Herein, we report the development of a new photoredox-neutral C–H activation methodology that allows a direct installation of methylene pyridinium moieties into arenes. The reaction products, benzylpyridinium salts, can be regarded as pseudo-benzylic halides with a pyridine leaving group instead of a halide ion with high potential for further synthetic elaboration.

## Results and Discussion

The synthetic approach employs methylenedipyridinium salt (**DiPyM**),^14^ accessed either from cheap solvent-like reagents, dichloromethane and pyridine, or, more efficiently, from dibromomethane or diiodomethane as the methylene source on multigram scale (Figure 2A). Although the use of dichloromethane affords the product in low yield, after filtration of crystalline methylenedipyridinium dichloride, the filtrate can be stored and used to continuously generate additional portions of the product. Subsequent counterion exchange from halide to the redox-inactive and solubility-enhancing hexafluorophosphate anion was accomplished via a standard anion metathesis. The final material is obtained in multigram quantities as a nonhygroscopic, air-stable, free-flowing crystalline solid that can be stored at ambient temperature without protection from light.

**Figure 2:**
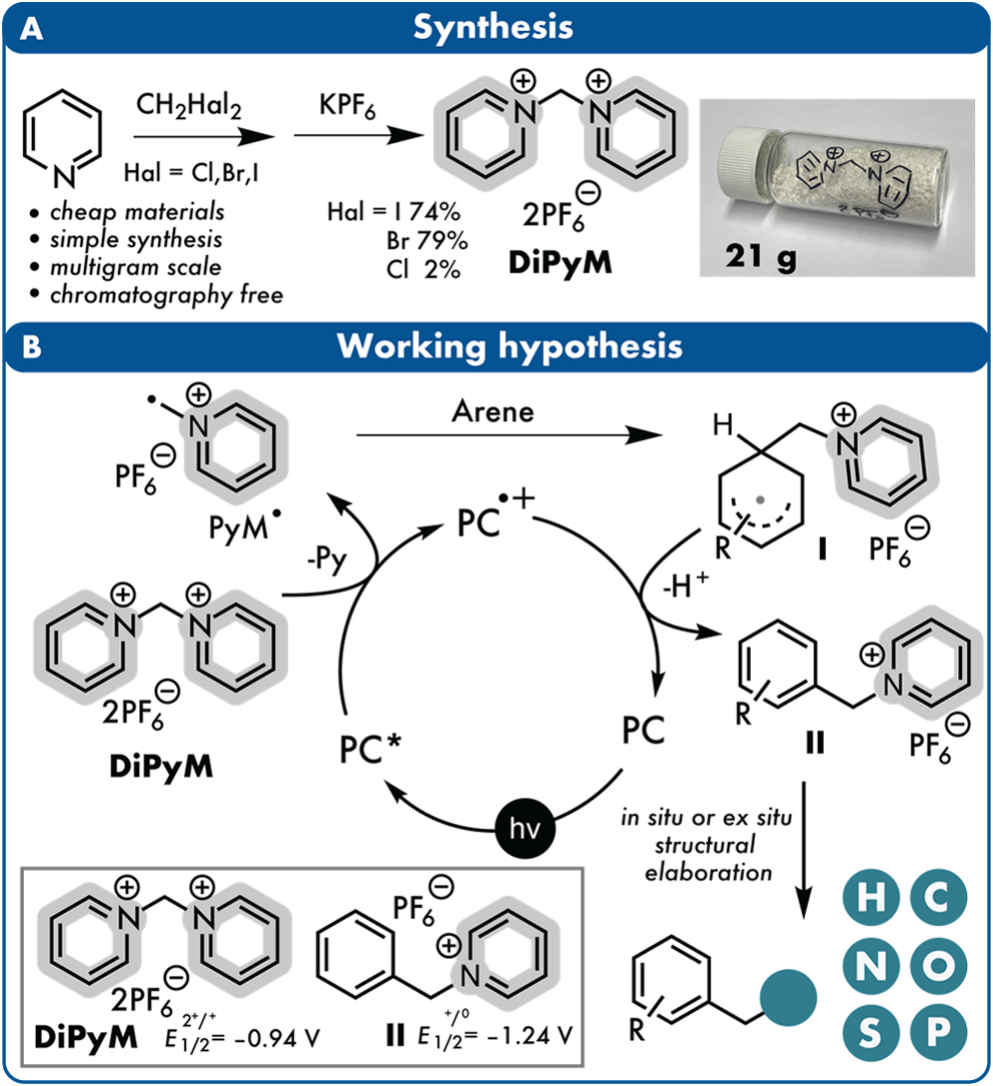
(A) Scalable synthesis of **DiPyM**. (B) Hypothesized photocatalytic cycle for arene C–H functionalization with versatile methylene pyridinium group.

For the proposed catalytic cycle depicted in Figure 2B, the reaction is initiated by oxidative quenching of the excited photocatalyst via single-electron transfer (SET) to the electron-deficient **DiPyM**. Subsequent fragmentation furnishes the **PyM** radical alongside neutral pyridine. Addition of the **PyM** radical to an arene generates cyclohexadienyl radical **I**, which is then oxidized by the oxidized photocatalyst to form the corresponding σ-complex, thereby completing a redox-neutral catalytic cycle. The pyridine base produced in the initial SET step facilitates deprotonation of the σ-complex to give the corresponding pyridinium salt. Given the high electrophilicity of the **PyM** radical, this pyridinium salt byproduct is anticipated to be inert toward further reaction.

The reaction design exploits the substantial difference in redox potentials between **DiPyM** and the product **II**. Selective reduction of **DiPyM** in the presence of **II** is critical to suppress undesired benzylic radical formation and downstream byproducts from **II**. The dicationic nature of **DiPyM** imparts a substantially less negative potential (−0.94 V vs SCE)^15^ relative to **II** (−1.24 V vs SCE),^16^ based on reported cyclic voltammetry data. The benzylpyridinium products **II** resemble Katritzky salts, widely used reagents for C–N bond cleavage to generate carbon-centered radicals,^17–19^ and therefore were anticipated to serve as versatile intermediates for C–C and C–heteroatom bond formation (Figure 2B).^20^ In contrast to Katritzky salts, where the electrophilic carbon is sterically shielded by ortho-phenyl substituents on the pyridine ring, benzylpyridinium salts might be amenable to direct nucleophilic substitution, imparting halide-like reactivity.

The reaction optimization began with a collection of photocatalysts capable of operating under an oxidative quenching regime (Figure 3A,B). As a stringent benchmark, we selected 1,3,5-trichlorobenzene, a challenging arene that lies beyond the reach of most classical C1 electrophilic substitution methods (Figure 1B). The initial screen encompassed highly reducing tribiphenyl amines (TBA),^21^ xanthene dyes (Eosin Y and Rhodamine B),^22^ and representative Ru- and Ir-based complexes.^23^ Gratifyingly, triphenylamine derivatives TBA, having extended conjugation for absorption in the visible region (entries 1–7), furnished notable levels of reactivity at 390 nm irradiation at low catalyst loading (2.5 mol %). Formation of the dimer (**PyM**)_2_ was observed consistently throughout the optimization experiments, and its identity was confirmed unambiguously by independent synthesis (Figure S3). To mitigate this side reaction and efficiently intercept the **PyM** radical, 10 equiv of an electron-deficient arene substrate was used in all optimization experiments.

**Figure 3:**
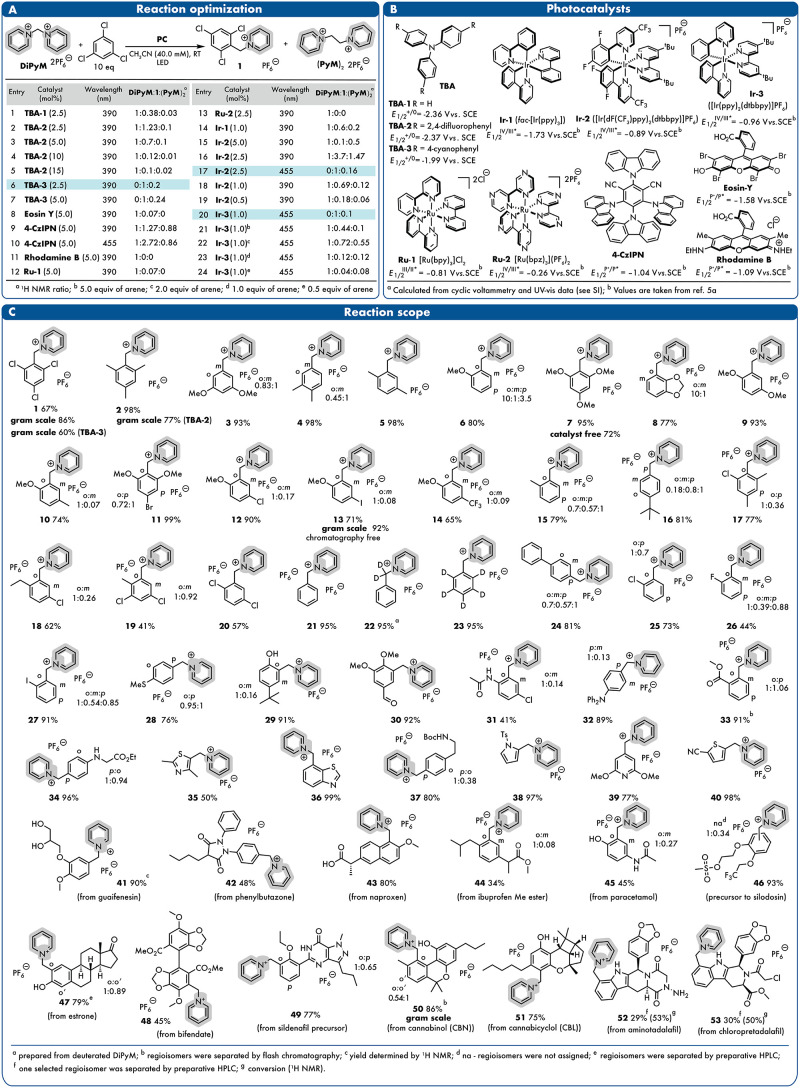
(A) Reaction optimization. (B) Photocatalysts used for optimization. (C) Substrate scope.

In contrast, Eosin Y and Rhodamine B (entries 8 and 11) afforded negligible amounts of the desired product, despite their sufficiently negative reduction potentials. Less reducing Ru-based complexes predominantly returned unreacted starting material (entries 12–13). The donor–acceptor cyanoarene photocatalyst **4-CzIPN** furnished increased product formation under blue-light (455 nm) irradiation; however, this was accompanied by substantial dimer formation (entry 10).

Although the metal-free photocatalyst **TBA-3** was among the best performers for 1,3,5-trichlorobenzene substrate, subsequent screening across a broader substrate set revealed inferior robustness relative to iridium-based photocatalysts. Among these, the most strongly reducing complex, **Ir-1**, performed worse than **TBA-3**, whereas **Ir-2** achieved full conversion under 455 nm irradiation (entries 14–19). Ultimately, **Ir-3** ([Ir­(ppy)_2_(dtbbpy)]­PF_6_) at 1.0 mol % afforded high conversion with the highest selectivity for C–H functionalization (**1**:(**PyM**)_2_ = 10:1; entry 20) and was therefore selected for subsequent studies, also considering its lower cost relative to **Ir-2**.

With the optimized conditions in hand, the substrate scope of the method was explored (Figure 3C). The reaction displays broad generality across electron-rich, electron-neutral, and some electron-poor aryl and heteroaryl substrates, affording the corresponding pyridinium products in moderate to excellent yields (30–98%). Functional groups, including halogens (Cl, F, I), alcohols, aldehydes, esters, amides, carbamates, thioethers, anilines, and phenols, are tolerated. Because of the high electrophilicity of the **PyM** radical, the regioselectivity of the reaction in substituted arenes is governed primarily by electronic effects. In these cases, the formation of regioisomeric mixtures could be viewed as a strategic advantage, as it enables rapid diversification of product space from a single starting material, which is particularly valuable for late-stage functionalization and library generation.^24^ The protocol is readily scalable, proceeding efficiently on gram scale, and can also be performed under metal-free conditions with good yield (compounds **1** and **2**). The chromatography-free scale-up possibility was also demonstrated with compound **13**. In the case of reactive substrates, the arene loading can be reduced to 2.0 equiv without loss of yield. Moreover, the highly electron-rich 1,3,5-trimethoxybenzene furnished the corresponding product **7** in 71% yield even in the absence of the catalyst, most likely via the formation of an electron-donor complex. Isotopic labeling at the benzylic position can be achieved with a deuterated analogue of **DiPyM** (compound **22**). The synthetic utility of the method was further demonstrated through the late-stage functionalization of a diverse set of bioactive molecules and pharmaceutical derivatives, including phenylbutazone, ibuprofen methyl ester, paracetamol, naproxen, guaifenesin, estrone, chloropretadalafil, cannabinoids and a mesylate precursor of silodosin (Figure 3C, **41**–**53**). The transformation was likewise applicable to structurally complex natural products such as estrone **47** and cannabinoids **50** and **51**, as well as to sensitive molecular scaffolds **52** and **53**, underscoring its broad functional-group tolerance and suitability for medicinally relevant substrates. Notably, in the case of estrone, the resulting regioisomers could be readily separated by preparative HPLC, providing access to individual products for further study. Regarding the limitations of this method, nitro- and nitrile-substituted arenes were found to be unreactive, even in the presence of activating methoxy groups (Figure S3).

The benzylic pyridinium intermediates proved to be a highly versatile platform for downstream diversification, enabling rapid access to a broad array of structural motifs from a single C1-installed precursor, demonstrated with **5** as a model compound (Figure 4A). The pyridinium ring could be chemoselectively reduced to the corresponding piperidine **54** using PtO_2_ as the hydrogenation catalyst.^25^ In addition, substitution of the pyridine leaving group with nitrogen nucleophiles proceeded smoothly, delivering the phthalimide, saccharin, and tosylamide derivatives (**55–57**) in good yields (60–95%). These transformations were promoted by “naked” iodide generated in situ from KI and 18-crown-6.^26^ The reaction can also be applied to unmodified aliphatic (**58**) and aromatic (**59**) amines. Under similar conditions, sulfur-based nucleophiles furnished the corresponding sulfone **61**, sulfide **62**, and thioester **63** products in high yields (81–90%). Benzylic alcohol (**68**) could be obtained via an ester intermediate,^26,27^ whereas the corresponding aldehyde **70** was accessed directly from **5** by Kröhnke oxidation using *N,N*-dimethyl-4-nitrosoaniline through an intermediate nitrone.^28^ The phosphonium salt **69**, a precursor for Wittig olefination, formed cleanly upon heating **5** with triphenylphosphine. Notably, reductive removal of the pyridinium group with Pd/H_2_ delivered the formally methylated product **67**, providing a direct entry to the medicinally relevant methyl motif from the same C1 linchpin. Finally, palladium-catalyzed cross-couplings, including Suzuki–Miyaura (**64**), Hiyama (**65**), and Sonogashira (**66**) reactions, efficiently forged C–C bonds with aryl and alkynyl partners (54–96%) under simple Pd­(OAc)_2_/PPh_3_ conditions. To our knowledge, the direct uncatalyzed substitution of pyridine with amine and phosphine nucleophiles, and Sonogashira coupling in simple pyridinium salts are unprecedented.

**Figure 4:**
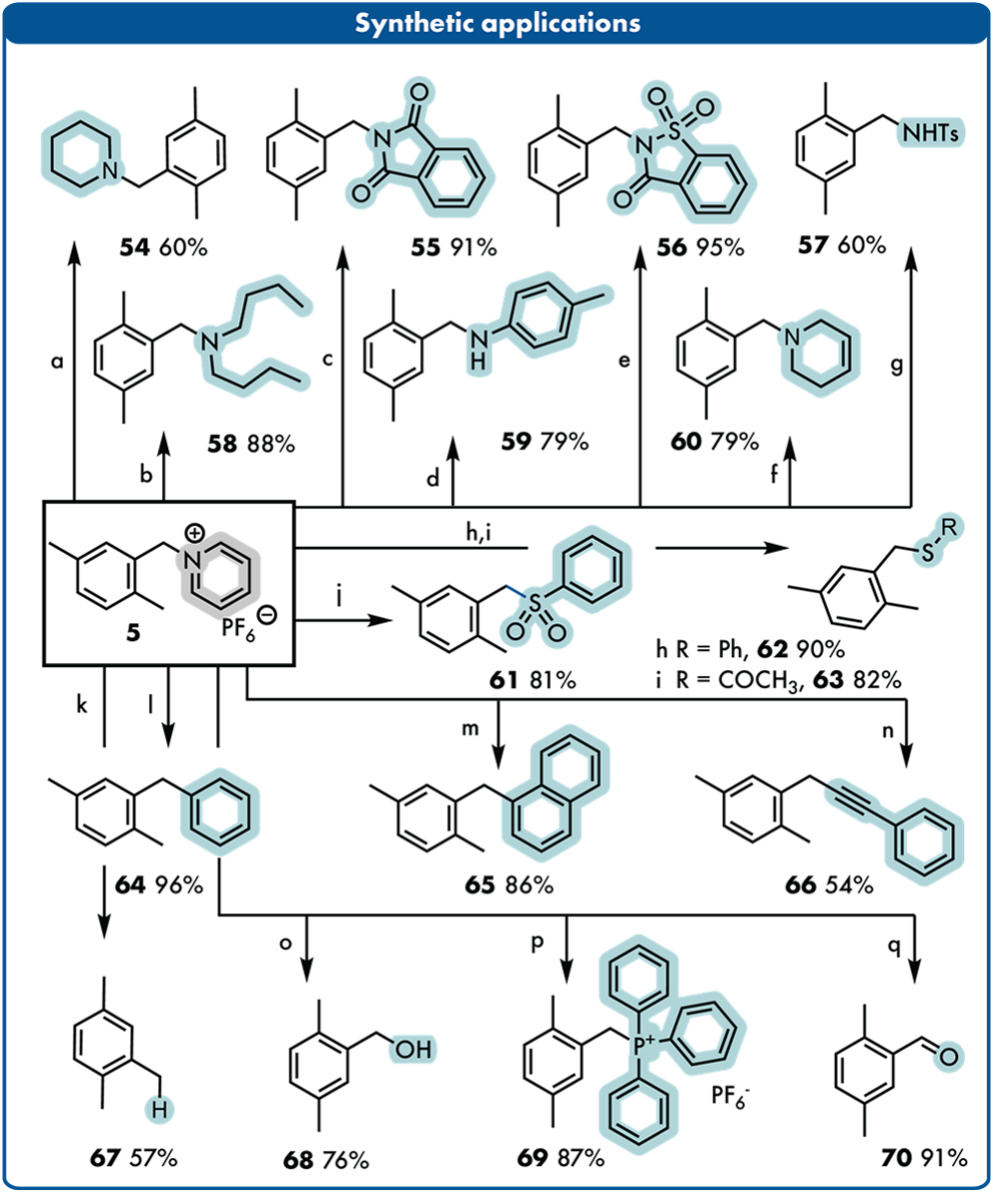
Functionalization of 1-(2,5-dimethylbenzyl)­pyridin-1-ium hexafluorophosphate **5**. Reagents and conditions: (a) H_2_, PtO_2_ (0.2 equiv), TEA (2.0 equiv), MeOH; (b) Dibutylamine, KI (2.0 equiv), 18-crown-6 (1.0 equiv), PhMe, 130 °C; (c) Potassium phthalimide (2.0 equiv), KO^t^Bu (1.5 equiv), KI (2.0 equiv), 18-crown-6 (1.0 equiv), PhMe, 130 °C; (d) *p*-methylaniline (2.0 equiv), KI (2.0 equiv), 18-crown-6 (1.0 equiv), PhMe, 130 °C; (e) Sodium saccharin (2.0 equiv), KI (2.0 equiv), 18-crown-6 (1.0 equiv), PhMe, 130 °C; (f) NaBH_4_, MeOH; (g) TsNH_2_ (2.0 equiv), KO^t^Bu (1.5 equiv), KI (2.0 equiv), 18-crown-6 (1.0 equiv), PhMe, 130 °C; (h) Thiophenol (1.1 equiv), KO^t^Bu (1.1 equiv), PhMe, 130 °C; (i) Potassium thioacetate (2.0 equiv), KI (2.0 equiv), 18-crown-6 (1.0 equiv), dioxane, 130 °C; (j) Sodium benzenesulfinate (2.0 equiv), KI (2.0 equiv), 18-crown-6 (1.0 equiv), PhMe, 130 °C; (k) H_2_, Pd/C, MeOH; (l) PhB­(OH)_2_ (2.0 equiv) DIPEA (3.0 equiv), Pd­(OAc)_2_ (0.05 equiv), PPh_3_ (0.15 equiv), EtOH, 100 °C; (m) Trimethoxy­(naphthalen-1-yl)­silane, TBAF·3H_2_O (2.0 equiv), Pd­(OAc)_2_ (0.05 equiv), PPh_3_ (0.15 equiv), EtOH, 100 °C; (n) Phenylacetylene, K_2_CO_3_ (3.0 equiv), Pd­(OAc)_2_ (0.05 equiv), PPh_3_ (0.15 equiv), CH_3_CN, 100 °C; (o) 1. Potassium acetate (2.0 equiv), KI (2.0 equiv), 18-crown-6 (1.0 equiv), dioxane, 130 °C. 2. KOH, EtOH; (p) PPh_3_, PhMe; (q) 1. *N*,*N*-Dimethyl-4-nitrosoaniline HCl (2.0 equiv), KOH (3.0 equiv), H_2_O. 2. HCl, H_2_O. DIPEA—*N*,*N*-diisopropylethyl amine, TBAF—tetrabutyl ammonium fluoride, TEA—triethylamine.

Further synthetic applications were demonstrated by performing postfunctionalization using crude reaction mixtures (Figure 5). As illustrated with caffeine, the corresponding pyridinium salt **71** was obtained in 76% yield according to ^1^H NMR; however, the compound proved sensitive to silica gel. Instead, the crude product was subjected to morpholine and sodium benzenesulfinate to afford the functionalized caffeines **72** and **73**, respectively, in good yields. The consecutive functionalization was also demonstrated with *N*-tosylpyrrole **74** in the synthesis of ester **75** (Figure 5). New derivatives of cannabinol were obtained by performing functionalization on separated regioisomers of **50**. Thus, the isomer **o’-50** was converted in good yields into benzyl derivative **76**, amine **77**, and aldehyde **78**. Interestingly, regioisomer **o-50** readily underwent cyclization under basic conditions to afford a cannabinoid derivative **79** featuring a unique tetracyclic scaffold. Performing nucleophilic substitution with *N*-methylpiperazine on the crude mixture of **49** provided readily separable derivatives **o-80** and p**-80**, the latter representing a variation of Sildenafil (Viagra), where sulfonamide is replaced by an aminomethyl fragment.

**Figure 5:**
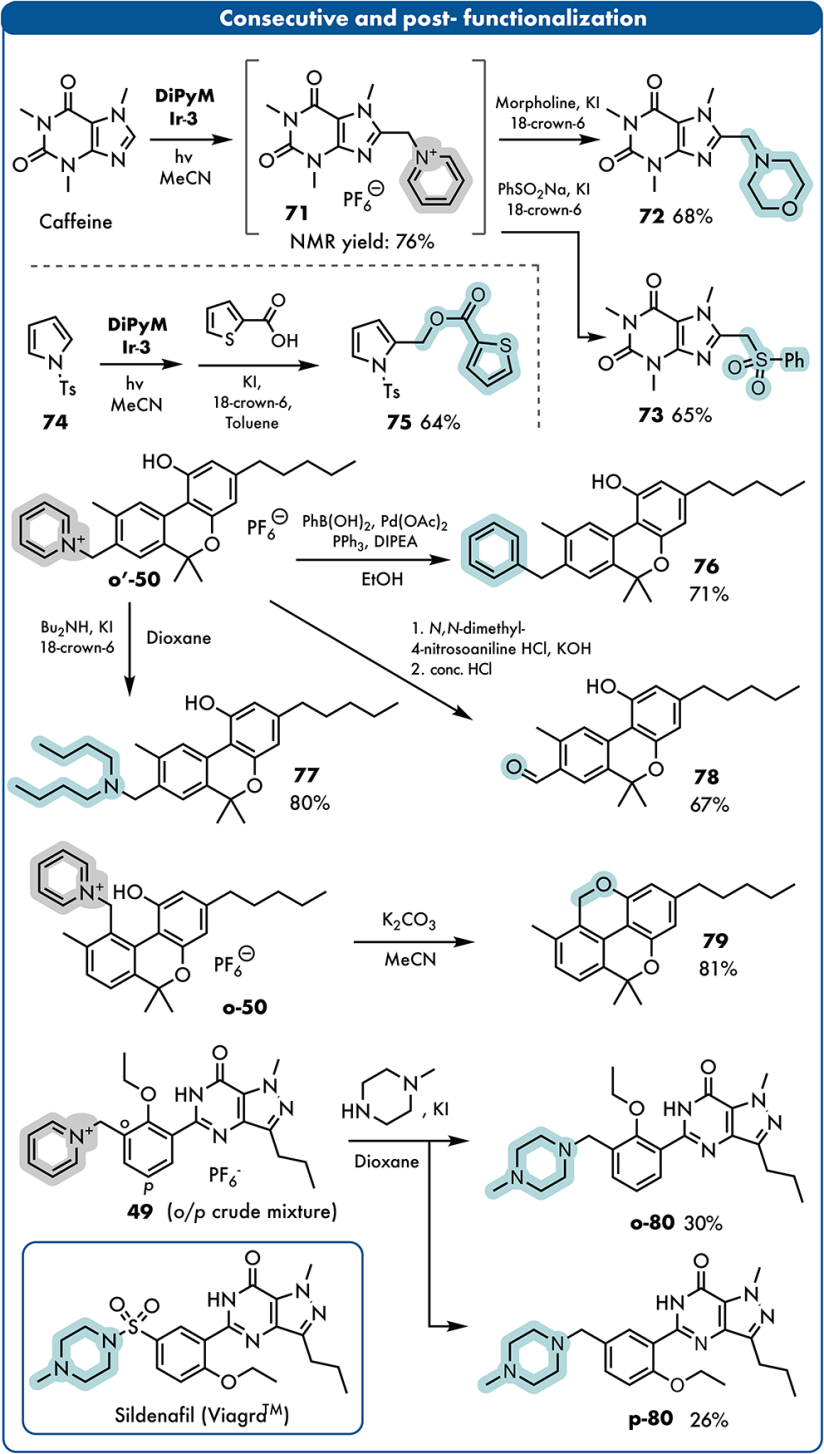
Telescoped functionalization of pyridinium salts.

Regarding the reaction mechanism, detection of the dimer (**PyM**)_2_ provides direct evidence for the intermediacy of the **PyM** radical. A series of control experiments further supports the catalytic cycle depicted in Figure 2B. In the absence of arene, the starting material remains largely intact, and only a small amount of (**PyM**)_2_ is formed. This observation indicates that the electrophilic **PyM** radical is not a competent reagent for the Minisci reaction with the pyridinium byproduct. This conclusion was further reinforced by conducting the reaction in the presence of exogenous pyridinium hexafluorophosphate, which remained unreactive under the standard conditions. Finally, the small kinetic isotope effect (KIE = 1.1) observed with a deuterated benzene substrate is consistent with a rapid rearomatization step accompanying the oxidation of radical intermediate **I**. As expected, the reaction was completely inhibited by the TEMPO radical scavenger (Figure S7).

## Conclusions

This work establishes a broadly applicable, photoredox-neutral C–H functionalization that directly installs a versatile C1 handle into arenes and represents the first example of the application of geminal bispyridinium derivatives in synthetic photochemistry. The resulting benzylpyridinium products serve as versatile intermediates, enabling diverse C–C and C–heteroatom bond formations from a single activation event, including access to formal methylation. Beyond providing a general platform for arene diversification and late-stage functionalization in medicinal chemistry, this strategy may also enable applications outside traditional synthetic chemistry. For example, the intrinsic cationic nature of the arylmethyl-pyridinium motif renders these products particularly attractive as precursors for mitochondria-targeting molecular architectures.^29^ Finally, pyridinium scaffolds are known to exhibit rich photophysical behavior, including charge-transfer emission,^30^ aggregation-induced emission,^31^ and environment-sensitive fluorescence.^32^

## Supplementary Material



## Notes

Detailed experimental procedures and analytical data (PDF)

## References

[1] Docherty J. H., Lister T. M., Mcarthur G., Findlay M. T., Domingo-Legarda P., Kenyon J., Choudhary S., Larrosa I. (2023). Transition-Metal-Catalyzed C–H Bond Activation
for the Formation of C–C Bonds in Complex Molecules. Chem. Rev..

[2] Guillemard L., Kaplaneris N., Ackermann L., Johansson M. J. (2021). Late-Stage C–H Functionalization
Offers New
Opportunities in Drug Discovery. Nat. Rev. Chem..

[3] Tsien J., Péter Á., Zeng X., Wang S., Jiang B., Emmanuel M. A., Oderinde M. S., Bolduc P. N., Nicastri M. C., Dey S., Collins M. R., Lee J. W., Bravo M., Richardson P. F., Sach N. W., Bernier L., Palkowitz M. D., Qiao J. X., Kawamata Y., Baran P. S. (2025). Accelerating Medicinal
Chemistry: A C­(sp3)-Rich Fragment Toolbox for Redox-Neutral Cross-Coupling. Angew. Chem., Int. Ed..

[4] Schönherr H., Cernak T. (2013). Profound Methyl Effects in Drug Discovery and a Call
for New C-H Methylation Reactions. Angew. Chem.,
Int. Ed..

[5] Holmberg-Douglas N., Nicewicz D. A. (2022). Photoredox-Catalyzed
C–H Functionalization Reactions. Chem.
Rev..

[6] Aragón J., Sun S., Fernández S., Lloret-Fillol J. (2024). Dichloromethane
as C1 Synthon for the Photoredox Catalytic
Cyclopropanation of Aromatic Olefins. Angew.
Chem., Int. Ed..

[7] Branytska O., Neumann R. (2004). Synthesis of Aromatic Aldehydes by
Oxidative Hydroxymethylation. Synlett.

[8] c Laali, K. K. Formaldehyde–Hydrogen Chloride. In Encyclopedia of Reagents for Organic Synthesis; John Wiley & Sons, Ltd, 2001.

[9] Neena N., Chaudhri V., Singh F. V., China H., Dohi T., Kumar R. (2023). Synthetic Utility of the Vilsmeier–Haack
Reagent in Organic
Synthesis. Synlett.

[10] Huang J., Chen Z., Wu J. (2021). Recent Progress
in Methyl-Radical-Mediated Methylation or Demethylation Reactions. ACS Catal..

[11] Minisci F., Porta O., Recupero F., Punta C., Gambarotti C., Pruna B., Pierini M., Fontana F. (2004). A New, Convenient, Highly Selective Free-Radical Hydroxymethylation
of Heteroaromatic Bases by Persulfate Oxidation of Ethylene Glycol
and Glycerol, Catalysed by AgNO_3_. Synlett.

[12] Xu Z., Zhang L. (2021). Methanol as a Formylating Agent in Nitrogen Heterocycles. Org. Biomol. Chem..

[13] Zhang G.-F., Li Y., Xie X.-Q., Ding C.-R. (2017). Ru-Catalyzed Regioselective Direct Hydroxymethylation
of (Hetero)­Arenes via C–H Activation. Org. Lett..

[14] Almarzoqi B., George A. V., Isaacs N. S. (1986). The Quarternisation
of Tertiary Amines with Dihalomethane. Tetrahedron.

[15] Tcyrulnikov N. A., Varadharajan R., Tikhomirova A. A., Pattabiraman M., Ramamurthy V., Wilson R. M. (2019). Modulation of Reduction Potentials
of Bis­(Pyridinium)­Alkane Dications through Encapsulation within Cucurbit[7]­Uril. J. Org. Chem..

[16] Zhu X.-Q., Tan Y., Cao C.-T. (2010). Thermodynamic
Diagnosis of the Properties and Mechanism
of Dihydropyridine-Type Compounds as Hydride Source in Acetonitrile
with “Molecule ID Card.”. J. Phys.
Chem. B.

[17] Klauck F. J. R., James M. J., Glorius F. (2017). Deaminative Strategy for the Visible-Light-Mediated
Generation of Alkyl Radicals. Angew. Chem.,
Int. Ed..

[18] He F.-S., Ye S., Wu J. (2019). Recent Advances
in Pyridinium Salts as Radical Reservoirs in Organic Synthesis. ACS Catal..

[19] Sowmiah S., Esperança J. M.
S. S., Rebelo L. P. N., Afonso C. A. M. (2018). Pyridinium
Salts: From Synthesis to Reactivity and Applications. Org. Chem. Front..

[20] Hanumanthu R., Weaver J. D. (2024). Cooperative
Catalytic
Coupling of Benzyl Chlorides and Bromides with Electron-Deficient
Alkenes. Org. Lett..

[21] Wu S., Žurauskas J., Domański M., Hitzfeld P. S., Butera V., Scott D. J., Rehbein J., Kumar A., Thyrhaug E., Hauer J., Barham J. P. (2021). Hole-Mediated Photoredox Catalysis:
Tris­(p-Substituted)­Biarylaminium Radical Cations as Tunable, Precomplexing
and Potent Photooxidants. Org. Chem. Front..

[22] Romero N.
A., Nicewicz D. A. (2016). Organic
Photoredox Catalysis. Chem. Rev..

[23] Prier C. K., Rankic D. A., MacMillan D. W. C. (2013). Visible Light Photoredox Catalysis
with Transition Metal Complexes: Applications in Organic Synthesis. Chem. Rev..

[24] Ham W. S., Hillenbrand J., Jacq J., Genicot C., Ritter T. (2019). Divergent
Late-Stage (Hetero)­Aryl C–H Amination by the Pyridinium Radical
Cation. Angew. Chem., Int. Ed..

[25] Hamilton T. S., Adams R. (1928). Reduction of Pyridine
Hydrochloride and Pyridonium Salts by Means
of Hydrogen and Platinum-Oxide Platinum Black. XVIII. J. Am. Chem. Soc..

[26] Jiang Z., He S., Yan Y., Liu Z., Xu X., Jin Z. (2024). Molecular
Editing of Toluene by Sequential Meta-C–H/Benzylic C–N
Deaminative Functionalizations. Org. Lett..

[27] McGrath A., Zhang R., Shafiq K., Cernak T. (2023). Repurposing Amine and
Carboxylic Acid Building Blocks with an Automatable Esterification
Reaction. Chem. Commun..

[28] Ehrlich P., Sachs F. (1899). Über Kondensationen
von aromatischen Nitroverbindungen mit Methylenderivaten. Chem. Ber..

[29] Zhong X., Yang Q., Chen Y., Jiang Y., Wang B., Shen J. (2019). A Mitochondria-Targeted
Fluorescent Probe Based on Coumarin–Pyridine
Derivatives for Hypochlorite Imaging in Living Cells and Zebrafish. J. Mater. Chem. B.

[30] Carlotti B., Consiglio G., Elisei F., Fortuna C. G., Mazzucato U., Spalletti A. (2014). Intramolecular Charge Transfer of Push–Pull
Pyridinium Salts in the Singlet Manifold. J.
Phys. Chem. A.

[31] Leduskrasts K., Suna E. (2019). Aggregation Induced
Emission by Pyridinium–Pyridinium Interactions. RSC Adv..

[32] Chen W., Elfeky S. A., Nonne Y., Male L., Ahmed K., Amiable C., Axe P., Yamada S., James T. D., Bull S. D., Fossey J. S. (2011). A Pyridinium Cation−π
Interaction Sensor for the Fluorescent Detection of Alkyl Halides. Chem. Commun..

