# Supplementary material for: A Photoredox C–H Arene Functionalization toward Substituted Methyls
Source: J Am Chem Soc. 2026 Jul 10;148(28):29876–82. doi: 10.1021/jacs.6c04756 (PMC13397540; doi:10.1021/jacs.6c04756)
Supplement: Supplementary file 1 [file ja6c04756_si_001.pdf]

Supporting Information

A Photoredox C-H Arene Functionalization Towards Substituted Methyls

*Jonas Žurauskas, Nojus Radzevičius, Paulius Vaickūnas, Gabija Sergejevaitė, and Edvinas Orentas**

Department of Organic Chemistry, Vilnius University, LT-03225 Vilnius, Lithuania

Table of Contents

General *3*

Synthetic Procedures *4*

Control experiments *56*

Determination of excited state reduction potentials of photocatalysts TBA 1-3 .... *58*

Copies of NMR spectra *59*

General.

Synthesis and characterization. Reagents were purchased from commercial sources and were used as received. Reactions were monitored by ^{1}H NMR and thin layer chromatography (TLC) carried out on 0.25 mm Merck silica plates (60F254), using UV light as the visualizing agent. Flash silica gel chromatography was performed using Fluorochem silica gel (60, particle size 0.043 – 0.063 mm). NMR spectra were recorded on Bruker DRX400 instruments in $CDCl_3$, CD_3CN, and d_6-DMSO, and were calibrated using residual protons in deuterated solvents as an internal reference ($CDCl_3$: ^{1}H NMR δ = 7.27 ppm, ^{13}C NMR δ = 77.16 ppm; CD_3CN: ^{1}H NMR δ = 1.94 ppm, ^{13}C NMR δ = 118.26 ppm; d_6-DMSO: ^{1}H NMR δ = 2.50 ppm, ^{13}C NMR δ = 39.52 ppm). The following abbreviations were used to explain NMR peak multiplicities: s = singlet, d = doublet, t = triplet, q = quartet, m = multiplet, and combinations thereof. ROESY experiments were performed where appropriate for regioisomer identification. Excitation and emission spectra were recorded using an Edinburgh Instruments FLS980 spectrometer. UV–Vis absorption spectra were recorded using a Lambda 950 spectrometer (PerkinElmer).

LC-MS analyses were conducted using a 6224 TOF mass spectrometer coupled to a 1260 Infinity high-performance liquid chromatography (HPLC) system (Agilent Technologies, Santa Clara, CA, USA). The HPLC system was equipped with a binary pump, an autosampler, a column compartment, and diode array detector (DAD). Chromatographic separations were performed on a 2.1 × 100 mm, 1.9 µm Hypersil GOLD™ Peptide column (Thermo Fisher Scientific). The column temperature was maintained at 40 °C, and the flow rate was set to 0.4 ml/min. UV chromatograms were recorded at 254 nm. The mobile phases consisted of water containing 0.1% formic acid (solvent A) and acetonitrile containing 0.1% formic acid (solvent B). Elution was performed using a linear gradient from 5% to 100% solvent B over 14 min, followed by a 2 min wash step at 100% B and a 4 min re-equilibration period, resulting in a total run time of 20 min.

Mass spectrometric detection was carried out in positive electrospray ionization (ESI+) mode. The source parameters were as follows: drying gas flow rate, 12 l/min; drying gas temperature 325 °C; nebulizer pressure 35 psig; capillary voltage 3500 V; and fragmentor voltage 100 V.

Preparative purification was carried out using a Büchi C-950 flash preparative chromatography system equipped with a 10 × 150 mm, 10 µm ReproSil C18 semi-preparative column (Dr. Maisch GmbH). Separations were performed at ambient temperature using a flow rate of 10 ml/min and an injection volume of 2.5 mL. The mobile phases consisted of water containing 0.1% formic acid (solvent A) and acetonitrile containing 0.1% formic acid (solvent B). Elution was achieved using a linear gradient from 5% to 100% solvent B over 45 min. Fractions corresponding to the target compounds were collected based on UV detection with an absorbance threshold of 0.02 AU and were subsequently combined and concentrated under reduced pressure to afford the purified products.

Synthetic Procedures

General Procedure I (Photocatalytic reaction). An oven-dried 25 mL vial equipped with a Teflon-coated magnetic stirring bar (3 mm × 12 mm) was charged with photocatalyst [Ir(ppy)$_2$(dtbbpy)]PF$_6$ (1.8 mg, 2.0 μmol, 1 mol%), **DiPyM 2PF$_6$** (92 mg, 0.20 mmol) and the corresponding (hetero)arene derivative (0.40–4.00 mmol, depending on the electron deficiency of the (hetero)arene). The vial was sealed with a rubber septum and flushed with dry argon for 5 min. Dry MeCN (5.0 mL) was then added via syringe, and the reaction mixture was further degassed by bubbling dry argon through the solution for an additional 5 min.
The sealed vial was placed directly in front of a 455 nm LED light source (Osram LZ4-40B208-0000; λ_{max} = 457 nm; 8.3 W electrical input, 5.0–6.0 W radiant flux) operated at 15.6 V. Temperature control was achieved using an industrial cooling fan (2 A, 18 V, 20 cm diameter) positioned approximately 10 cm from the reaction vial, maintaining the internal reaction temperature at 40 °C throughout irradiation.
After irradiation for 16–48 h, the reaction mixture was concentrated under reduced pressure. The resulting residue was dissolved in a minimal volume of MeCN/CH_2Cl_2 (1:9, v/v) and directly loaded onto a silica gel column. Purification by flash column chromatography (MeCN/CH_2Cl_2 gradient from 1:9 to 2:8, v/v) afforded the desired coupled products.

Notes:

Light source positioning. It is critical to maintain a fixed and reproducible distance between the reaction vial and the light source. The vial must be positioned directly above the high-power LED and cooled efficiently with continuous forced air circulation.

Atmosphere control. All reactions must be performed under rigorously oxygen- and moisture-free conditions. Thorough degassing of solvents and maintenance of an inert argon atmosphere are essential for reproducibility.

LED array and power supply. Each set of four LED modules, mounted on CPU heatsinks, was powered using a Consort EV231 power supply operated at 54 V and 396 mA (21 W). The integrated time counter of the power supply was used to accurately record irradiation times.

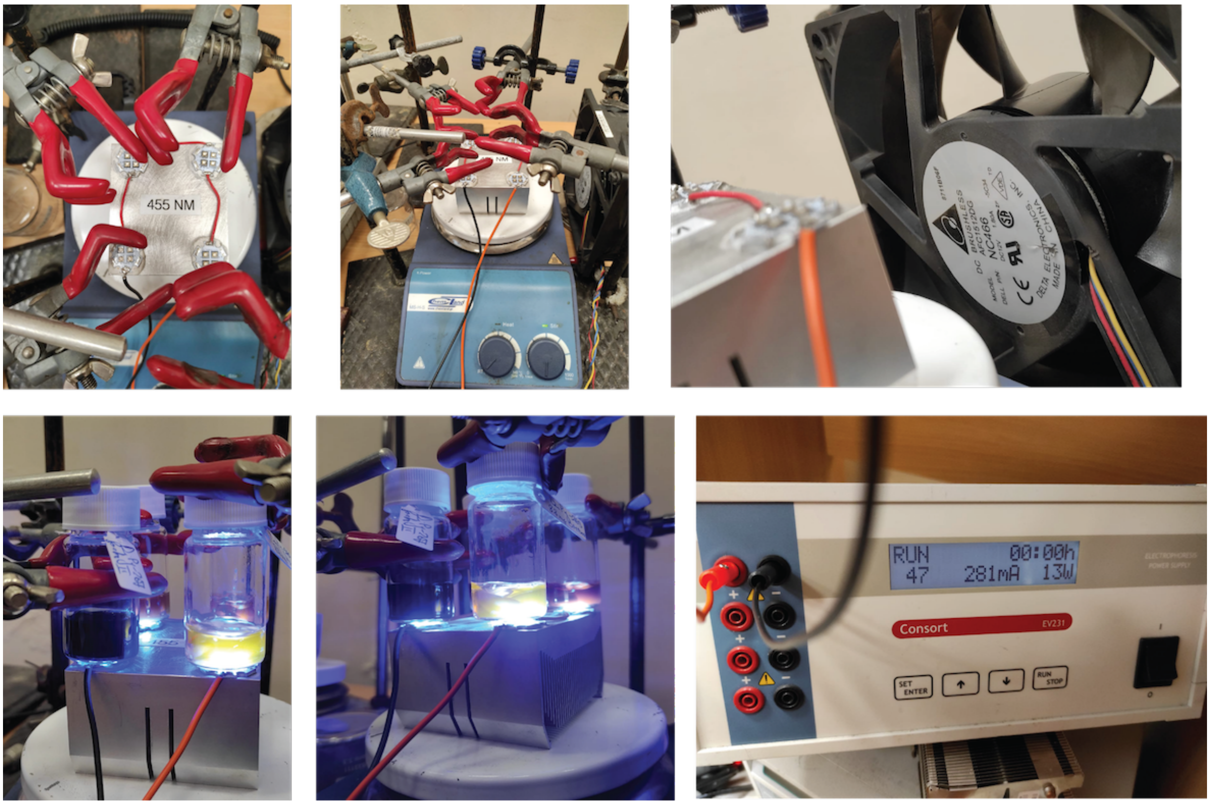

Figure S1. Small scale reaction setup.

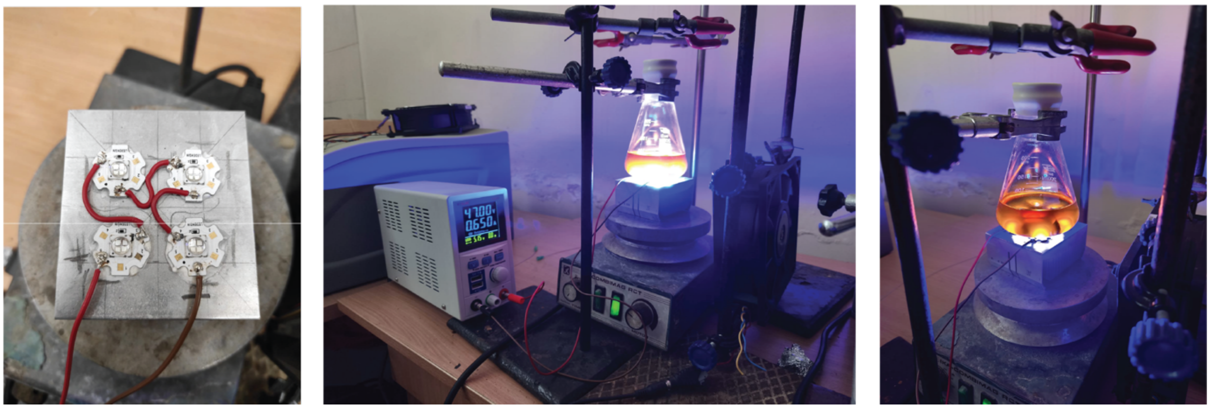

Figure S2. Gram-scale reaction setup

NMR conversion analysis. Reaction conversion was determined by ^{1}H NMR spectroscopy. An aliquot (300 μL) of the reaction mixture was withdrawn by syringe and transferred to a 5 mL round-bottom flask. Solvent was removed under reduced pressure, after which CD_3CN (0.50 mL) was added. The mixture was sonicated for 10 s and transferred to an NMR tube for analysis.

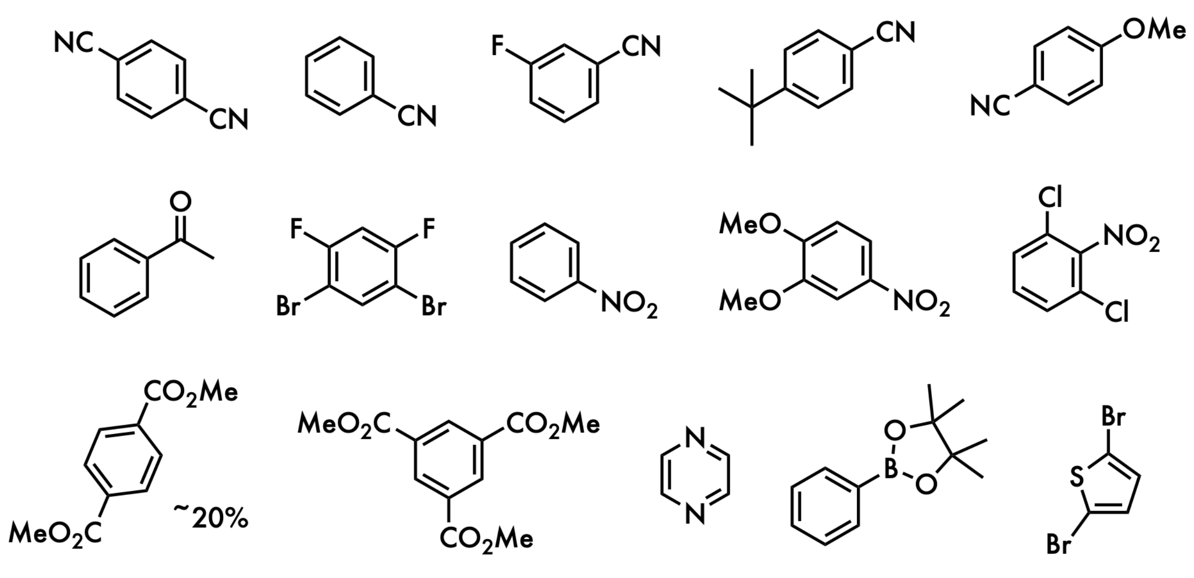

Figure S3. Unreactive substrates.

General procedure II (Nucleophilic substitution). A screw cap vial was loaded with pyridinium salt (0.29 mmol, 1 equiv), nucleophile (0.58 mmol, 2.0 equiv), base (if needed, KOtBu or DIPEA, 0.44 mmol, 1.5 equiv), potassium iodide (97 mg, 0.58 mmol, 2.0 equiv) and 18-crown-6 (77 mg, 0.29 mmol, 1.0 equiv). After flushing with argon, solvent (dry toluene or dioxane, 5 mL) was added, the vial was sealed and stirred vigorously with heating in an oil bath for 16 h. The reaction mixture was diluted with DCM (10 mL) and water (20 mL). The layers were separated, and the aqueous layer was further extracted with DCM (2x10 mL). The combined organic layers were dried with Na_2SO_4, filtered and concentrated to dryness. The crude material was purified by column chromatography to provide the product.

General procedure III (Preparation of pyridinium salt references for regioisomers assignments). **A** *From benzyl halides*: In a screw-cap vial, benzyl halide (1.0 mmol) was dissolved in pyridine (1.5 mL). The vial was sealed and stirred in an oil bath at 90°C for 2 hours. After concentration under reduced pressure, the residue was dissolved in water (5.0 mL), and an aqueous solution of KPF_6 (2.0 mmol in 5.0 mL) was added under vigorous stirring. The resulting suspension was filtered through a sintered glass funnel, and the filter cake was washed with water (3 × 10 mL) and petroleum ether (2 × 10 mL). The solid was then dried under vacuum for 16 hours to afford the corresponding pyridinium salt as an off-white solid.
Note: In some cases, after the addition of KPF_6, the pyridinium salt precipitated as an oily solid that could not be easily filtered. In such cases, the mixture was diluted with dichloromethane (10 mL), and the layers were separated. The aqueous layer was further extracted with DCM (2 × 10 mL). The combined organic layers were dried over Na_2SO_4, filtered, and concentrated to dryness. The resulting solid was dried under vacuum to afford the corresponding pyridinium salt as a tan, waxy solid (>90% yield).
B *From benzyl alcohols*: In a screw-cap vial, benzyl alcohol (1.0 mmol) and p-toluenesulfonyl chloride (2.0 mmol) were dissolved in pyridine (5.0 mL). The tube was sealed and stirred in a 90°C oil bath for 2 hours. The mixture was diluted with water (5.0 mL), and a solution of KPF_6 (3.0 mmol) in water (10 ml) was added under vigorous stirring. The resulting suspension was filtered through a sintered glass filter funnel, and the filter cake was washed with water (3x10 mL) and petroleum ether (2x10 mL).

The resulting solid was dried under vacuum for 16 hours to provide the corresponding pyridinium salt as an off-white solid (>90% yield).

Preparation of 1,1'-methylenebis(pyridin-1-ium) hexafluorophosphate (DiPyM $2PF_6$)

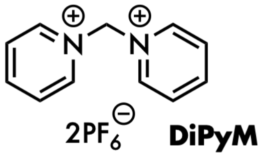

Procedure 1. Pyridine (19.5 g, 246.4 mmol, 19.9 mL, ρ = 0.98 g·cm^{-3}, 4.0 equiv) was added to MeCN (250 mL) in a 500 mL round-bottom flask. CH_2I_2 (5.0 mL, 61.6 mmol, ρ = 3.30 g·cm^{-3}, 1.0 equiv) was added dropwise via syringe over 1 min. The reaction mixture was heated to reflux under air for 16 h. After cooling to room temperature, the resulting yellow solid was collected by filtration through a sintered glass funnel, washed with cold MeCN (50 mL), and dried under reduced pressure. The crude solid was dissolved in warm deionized water (750 mL) with vigorous stirring. An aqueous solution of KPF_6 (34 g, 0.37 mol, 3.0 equiv in 370 mL H_2O) was added in one portion, immediately inducing formation of a yellowish precipitate. The suspension was stirred for 15 min, after which the solid was collected by filtration and thoroughly washed with deionized water (500 mL). Drying under vacuum overnight afforded **DiPyM $2PF_6$** as an off-white microcrystalline solid (21.0 g, 74% yield).

^{1}H NMR (400 MHz, CD_3CN) δ 9.04 – 8.93 (m, 4H), 8.75 (tt, *J* = 7.9, 1.4 Hz, 2H), 8.29 – 8.16 (m, 4H), 6.99 (s, 2H).

^{13}C NMR (101 MHz, CD_3CN) δ 149.2, 146.3, 129.2, 77.6

^{31}P NMR (162 MHz, CD_3CN) δ -131.5, -135.9, -140.2, -144.6, -144.6, -149.0, -149.0, -153.3, -157.7.

^{19}F NMR (376 MHz, CD_3CN) δ -71.7, -73.5.

HRMS-ESI(+) Calcd. for $C_{11}H_{12}N^{2+}$ $[M]^{2+}$: 86.0495; Found: 86.0494.

Procedure 2. Pyridine (197.8 g, 2.50 mol, 202 mL, 2.5 equiv) was added to CH_2Cl_2 (85.0 g, 1.00 mol, 64.0 mL, ρ = 1.33 g·cm^{-3}, 1.0 equiv) in a 500 mL round-bottom flask. The reaction mixture was heated to reflux under air for 36 h using a reflux condenser. The resulting white solid was collected by filtration through a sintered glass funnel, washed with CH_2Cl_2 (100 mL), and dried. The solid was then dissolved in deionized water (300 mL) with vigorous stirring, and an aqueous solution of KPF_6 (68.03 g, 0.37 mol, 6.0 equiv in 370 mL H_2O) was added, inducing formation of a white precipitate. The suspension was stirred for 15 min, filtered, and the solid was thoroughly washed with deionized water (250 mL). Drying under vacuum overnight afforded **DiPyM $2PF_6$** as a white microcrystalline solid (8.0 g, 2% yield).

Procedure 3 (cost and yield optimum). Pyridine (19.5 g, 246.4 mmol, 19.9 mL, ρ = 0.98 g·cm^{-3}, 4.0 equiv) was added to MeCN (20 mL) in a 150 mL pressure vial. CH_2Br_2 (4.3 mL, 61.6 mmol, ρ = 2.5 g·cm^{-3}, 1.0 equiv) was added and the reaction mixture was heated at 85°C under air for 72 h. After cooling to room temperature, the mixture containing white precipitate was diluted with diethyl ether (100 mL), filtered through a sintered glass funnel, washed with diethyl ether, and dried under reduced pressure.

The crude solid (20.31 g) was dissolved in deionized water (300 mL) with vigorous stirring. An aqueous solution of NH_4PF_6 (30 g, 0.18 mol, 3.0 equiv in 100 mL H_2O) was added in one portion, immediately inducing formation of a thick white precipitate. The suspension was stirred for 15 min, after which the solid was collected by filtration and thoroughly washed with deionized water. Drying under vacuum overnight afforded **DiPyM 2PF$_6$** as a white solid (22.5 g, 79% yield).

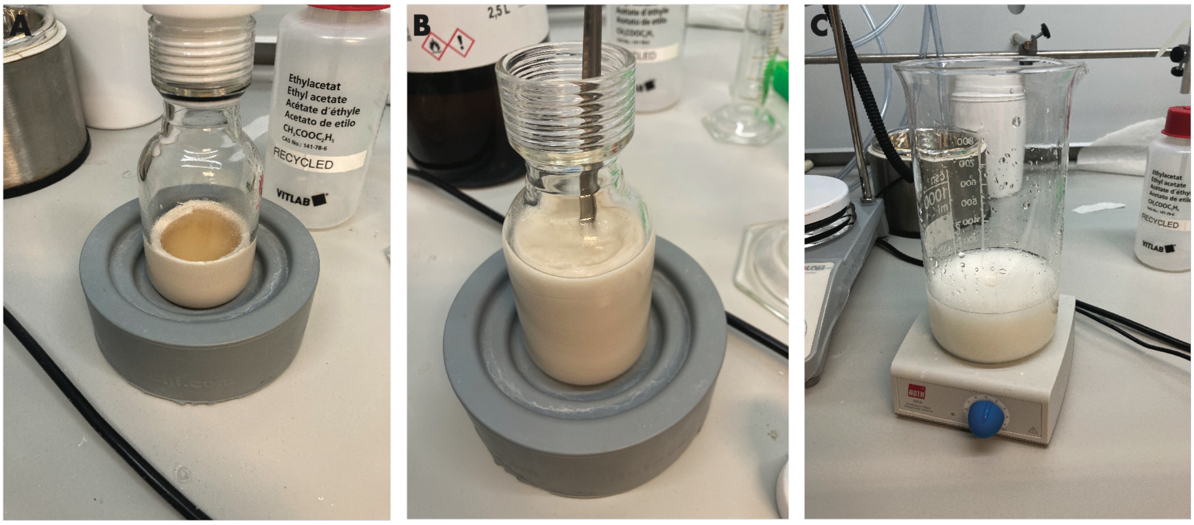

Figure S4. (A) Reaction mixture. (B) Reaction mixture diluted with diethyl ether. (C) Precipitation of **DiPyM 2PF$_6$** during the anion metathesis reaction.

Preparation of di(1λ^4-pyridin-1-yl)methane-*d*$_2$ hexafluorophosphate (*d*$_2$-DiPyM 2PF$_6$)

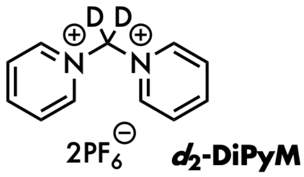

Pyridine (4.40 g, 55.59 mmol, 4.8 mL, ρ = 0.98 g·cm^{-3}, 6.0 equiv) was added to MeCN (25.0 mL) in a 100 mL round-bottom flask, followed by dropwise addition of CD_2I_2 (0.75 mL, 9.26 mmol, ρ = 3.32 g·cm^{-3}, 1.0 equiv) via syringe over 1 min. The reaction mixture was heated to reflux under air for 16 h. After cooling to room temperature, the resulting yellow solid was collected by filtration through a sintered glass funnel, washed with cold MeCN (50 mL), and dried. The solid was dissolved in warm deionized water (50 mL) with vigorous stirring, and an aqueous solution of KPF_6 (8.53 g, 46.3 mmol, 5.0 equiv in 50 mL H_2O) was added in one portion, inducing formation of a white precipitate (anion metathesis). The suspension was stirred for 15 min, filtered, and the solid was thoroughly washed with deionized water (100 mL). Drying under vacuum overnight afforded ***d*$_2$-DiPyM 2PF$_6$** as a white microcrystalline solid (3.3 g, 77% yield).

^{1}H NMR (400 MHz, CD_3CN) δ 8.99 (dd, *J* = 6.7, 1.6 Hz, 4H), 8.81 – 8.69 (m, 2H), 8.31 – 8.16 (m, 4H).

^{13}C NMR (101 MHz, CD_3CN) δ 150.7, 146.0, 130.4, 78.4, 78.1, 77.9, 77.6.

^{31}P NMR (162 MHz, CD_3CN) δ -131.5, -135.9, -140.2, -144.6, -149.0, -153.3, -157.7.

^{19}F NMR (376 MHz, CD_3CN) δ -71.5, -73.4.

Preparation of 1,1'-(ethane-1,2-diyl)bis(pyridin-1-ium) hexafluorophosphate $(PyM)_2$ $2PF_6$

Pyridine (25.3 g, 319.4 mmol, 25.8 mL, ρ = 0.98 g·cm^{-3}, 6.0 equiv) was added to MeCN (150.0 mL) in a 250 mL round-bottom flask, followed by dropwise addition of 1,2-dibromoethane (10.0 g, 4.6 mL, 52.23 mmol, ρ = 2.17 g·cm^{-3}, 1.0 equiv) via syringe over 1 min. The reaction mixture was heated to reflux under air for 16 h. After cooling to room temperature, the resulting yellow solid was collected by filtration through a sintered glass funnel, washed with cold MeCN (50 mL), and dried. The solid was dissolved in warm deionized water (350 mL) with vigorous stirring, and an aqueous solution of KPF_6 (49.0 g, 266.15 mmol, 5.0 equiv in 350 mL H_2O) was added in one portion, inducing formation of a white precipitate (anion metathesis). The suspension was stirred for 15 min, filtered, and the solid was thoroughly washed with deionized water (100 mL). Drying under vacuum overnight afforded *(***$(PyM)_2$ $2PF_6$** as a white microcrystalline solid (21.2 g, 86% yield).

^{1}H NMR (400 MHz, CD_3CN) δ 8.72 – 8.51 (m, 6H), 8.10 (t, *J* = 7.1 Hz, 4H), 5.08 (s, 4H).

^{13}C NMR (101 MHz, CD_3CN) δ 148.51, 146.01, 130.22, 118.43, 60.69.

^{31}P NMR (162 MHz, CD_3CN) δ -131.5, -135.9, -140.3, -144.6, -149.0, -153.4, -157.7.

^{19}F NMR (376 MHz, CD_3CN) δ -71.6, -73.5.

HRMS-ESI(+) Calcd. for $C_{12}H_{14}N^{2+}$ $[M]^{2+}$: 93.0573; Found: 93.0572.

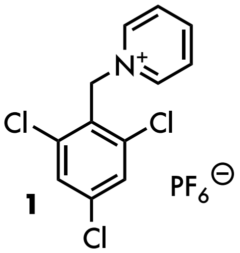

1

Following **General Procedure I**: **DiPyM $2PF_6$** (92 mg, 0.20 mmol), 1,3,5-trichlorobenzene (363 mg, 2.00 mmol, 10.0 equiv), and $[Ir(ppy)_2(dtbbpy)]PF_6$ (1.8 mg, 2.0 μmol, 1 mol%) were combined and irradiated with 455 nm LEDs for 16 h. Purification by column chromatography (MeCN/DCM = 1:9) afforded **1** as a white microcrystalline solid (57 mg, 67%).

Scale-up procedure. When the reaction was performed on a 10-fold scale using four 455 nm LED modules in a 250 mL Erlenmeyer flask (75 mL MeCN), **1** was obtained as a white microcrystalline solid (722 mg, 86%);

Scale-up procedure II. When the reaction was performed on a 10-fold scale using four 455 nm LED modules in a 250 mL Erlenmeyer flask (75 mL MeCN) and **TBA-3** (2.5 mol%, 27.4 mg), **1** was obtained as a white microcrystalline solid (500 mg, 60%).

^{1}H NMR (400 MHz, CD_3CN) δ 8.73 – 8.65 (m, 2H), 8.55 (tt, *J* = 7.8, 1.4 Hz, 1H), 8.08 – 7.98 (m, 2H), 7.68 (s, 2H), 5.97 (s, 2H).

^{13}C NMR (101 MHz, CD_3CN) δ 147.3, 138.4, 138.0, 129.9, 129.2, 127.6, 59.5.

^{31}P NMR (162 MHz, CD_3CN) δ -131.6, -135.9, -140.3, -144.6, -149.0, -153.4, -157.7.

^{19}F NMR (376 MHz, CD_3CN) δ -71.9, -73.8.

HRMS-ESI(+) Calcd. for $C_{12}H_9Cl_3N^+$ $[M]^+$: 271.9801; Found: 271.9803.

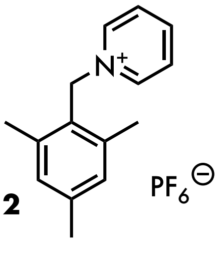

2

Following **General Procedure I**: **DiPyM 2PF₆** (92 mg, 0.20 mmol), mesitylene (48.1 mg, 0.40 mmol, 55.7 µL, ρ = 0.864 g/cm^3, 2.0 equiv), and $[Ir(ppy)_2(dtbbpy)]PF_6$ (1.8 mg, 2.0 µmol, 1 mol%) were combined and irradiated with 455 nm LEDs for 16 h. Purification by column chromatography (MeCN/DCM = 1:9) afforded **2** as a white microcrystalline solid (70 mg, 98%).

Scale-up procedure I. When the reaction was performed on a 10-fold scale using four 455 nm LED modules in a 250 mL Erlenmeyer flask (50 mL MeCN), **1** was obtained as a white microcrystalline solid (620 mg, 87%).

Scale-up procedure II. When the reaction was performed on a 10-fold scale using four 455 nm LED modules in a 250 mL Erlenmeyer flask (50 mL MeCN) and **TBA-2** (2.5 mol, 29 mg), **1** was obtained as a white microcrystalline solid (650 mg, 91%).

^{1}H NMR (400 MHz, CD_3CN) δ 8.59 – 8.41 (m, 3H), 8.10 – 7.90 (m, 2H), 7.06 (s, 2H), 5.76 (s, 2H), 2.32 (s, 3H), 2.22 (s, 6H).

^{13}C NMR (101 MHz, CD_3CN) δ 147.0, 144.5, 141.8, 140.4, 130.9, 129.6, 125.8, 59.8, 21.2, 19.9.

^{31}P NMR (162 MHz, CD_3CN) δ -131.5, -135.9, -140.3, -144.6, -149.0, -153.3, -157.7.

^{19}F NMR (376 MHz, CD_3CN) δ -71.8, -73.7.

HRMS-ESI(+) Calcd. for $C_{15}H_{18}N^+$ $[M]^+$: 212.1434; Found: 212.1439.

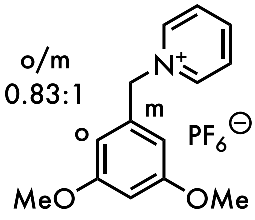

3

Following General Procedure I: **DiPyM 2PF$_6$** (92 mg, 0.20 mmol), 1,3-dimethoxybenzene (55.3 mg, 0.4 mmol, 203 µL, ρ = 1.11 g/cm^3, 2.0 equiv), and [Ir(ppy)$_2$(dtbbpy)]PF$_6$ (1.8 mg, 2.0 µmol, 1 mol%) were combined and irradiated with 455 nm LEDs for 16 h. Purification by column chromatography (MeCN/DCM = 1:9) afforded an inseparable mixture of regioisomers **3** as a white microcrystalline solid (70 mg, 93%).

^{1}H NMR (400 MHz, CD$_3$CN) (major regioisomer) δ 8.86 – 8.77 (m, 2H), 8.76 – 8.67 (m, 1H), 8.57 – 8.32 (m, 1H), 7.95 (q, *J* = 6.6 Hz, 2H), 7.41 (t, *J* = 8.5 Hz, 1H), 6.71 (d, *J* = 8.5 Hz, 2H), 5.68 (s, 2H), 3.88 (s, 6H).

^{13}C NMR (101 MHz, CD$_3$CN) (mixture of regioisomers) δ 164.0, 160.4, 159.8, 146.7, 146.6, 145.8, 145.4, 133.6, 133.5, 129.1, 129.0, 114.3, 110.3, 106.3, 105.1, 99.8, 61.8, 56.9, 56.4, 56.3, 55.9, 55.2.

^{31}P NMR (162 MHz, CD$_3$CN) δ -131.5, -135.9, -140.2, -144.6, -148.9, -153.3, -157.7.

^{19}F NMR (376 MHz, CD$_3$CN) δ -71.8, -73.6.

HRMS-ESI(+) Calcd. for $C_{14}H_{16}NO_2^+$ [M]$^+$: 230.1181; Found: 230.1174.

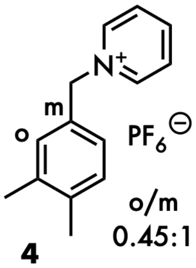

4

Following General Procedure I: **DiPyM 2PF$_6$** (92 mg, 0.20 mmol), *o*-xylene (43 mg, 0.4 mmol, 48 µL, ρ = 0.88 g/cm^3, 2.0 equiv.), and [Ir(ppy)$_2$(dtbbpy)]PF$_6$ (1.8 mg, 2.0 µmol, 1 mol%) were combined and irradiated with 455 nm LEDs for 16 h. Purification by column chromatography (MeCN/DCM = 1:9) afforded an inseparable mixture of regioisomers of **4** (67 mg, 98%).

^{1}H NMR (400 MHz, CD$_3$CN) (mixture of regioisomers) δ 8.79 – 8.73 (m, 2H), 8.67 – 8.62 (m, 0.8H), 858 – 8.48 (m, 1.2H), 8.04 (t, *J* = 7.0 Hz, 2H), 7.35 (d, *J* = 7.6 Hz, 0.45H), 7.28 – 7.23 (m, 2H), 7.23 – 7.18 (m, 1H), 5.80 (s, 0.9H), 5.65 (s, 2H), 2.34 (s, 1.3H), 2.29 (s, 3H), 2.28 (s, 3H), 2.18 (s, 1.3H).

^{13}C NMR (101 MHz, CD$_3$CN) (mixture of regioisomers) δ 146.7, 146.7, 144.9, 144.8, 139.4, 139.3, 138.7, 137.2, 132.4, 131.1, 130.9, 130.8, 129.2, 129.1, 129.1, 129.0, 127.2, 127.1, 65.0, 63.6, 20.2, 19.4, 19.2, 15.1.

^{31}P NMR (162 MHz, CD$_3$CN) δ -131.5, -135.9, -140.2, -144.6, -149.0, -153.3, -157.7.

^{19}F NMR (376 MHz, CD$_3$CN) δ -71.7, -73.6.

HRMS-ESI(+) Calcd. for $C_{14}H_{14}N^+$ [M]$^+$: 198.1277; Found: 198.1278.

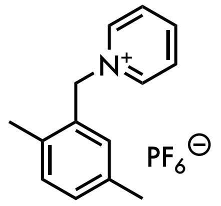

5

Following General Procedure I: **DiPyM 2PF$_6$** (92 mg, 0.20 mmol), *p*-xylene (43 mg, 0.4 mmol, 49 uL, ρ = 0.861 g/cm^3, 2.0 equiv), and [Ir(ppy)$_2$(dtbbpy)]PF$_6$ (1.8 mg, 2.0 μmol, 1 mol%) were combined and irradiated with 455 nm LEDs for 16 h. Purification by column chromatography (MeCN/DCM = 1:9) afforded **5** as a white microcrystalline solid (67 mg, 98%).

^{1}H NMR (400 MHz, CD$_3$CN) δ 8.63 (d, *J* = 6.1 Hz, 2H), 8.52 (t, *J* = 7.8 Hz, 1H), 8.02 (t, *J* = 7.0 Hz, 2H), 7.23 (d, *J* = 1.2 Hz, 2H), 7.08 (s, 1H), 5.70 (s, 2H), 2.22 (s, 3H).

^{13}C NMR (101 MHz, CD$_3$CN) δ 147.6, 145.7, 138.2, 136.0, 132.6, 132.5, 132.2, 131.7, 129.9, 64.0, 21.2, 19.1.

^{19}F NMR (376 MHz, CD$_3$CN) δ -72.0, -73.9.

^{31}P NMR (162 MHz, CD$_3$CN) δ -135.9, -140.3, -144.6, -149.0, -153.4.

HRMS-ESI(+) Calcd. for $C_{14}H_{14}N^+$ [M]$^+$: 198.1277; Found: 198.1281.

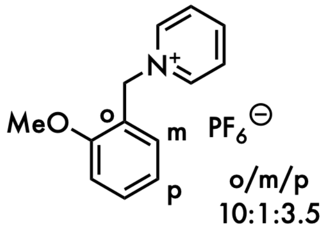

6

Following General Procedure I: **DiPyM 2PF$_6$** (92 mg, 0.20 mmol), anisole (43.3 mg, 0.4 mmol, 43 μL, ρ = 1.0 g/cm^3, 2.0 equiv), and [Ir(ppy)$_2$(dtbbpy)]PF$_6$ (1.8 mg, 2.0 μmol, 1 mol%) were combined and irradiated with 455 nm LEDs for 16 h. Purification by column chromatography (MeCN/DCM = 1:9) afforded an inseparable mixture of regioisomers of **6** as a white microcrystalline solid (55 mg, 80%).

^{1}H NMR (400 MHz, CDCl$_3$) (mixture of regioisomers) δ 8.77 – 8.73 (m, 1.07H), 8.72 – 8.69 (m, 0.34H), 8.55 – 8.42 (m, 0.72H), 8.04 – 7.92 (m, 1.44H), 7.56 – 7.35 (m, 1.43H), 7.16 – 6.93 (m, 1.51H), 5.66 (s, 0.1H), 5.64 (s, 1H), 5.63 (s, 0.35H), 3.80 (s, 1H), 3.79 (s, 0.15H), 3.78 (s, 1.5H).

^{13}C NMR (101 MHz, CDCl$_3$) (mixture of regioisomers) δ 159.1, 146.8, 145.5, 133.1, 132.5, 132.0, 129.4, 129.0, 125.5, 121.9, 121.9, 115.7, 112.5, 65.0, 61.9, 56.2, 56.1.

^{31}P NMR (162 MHz, CD$_3$CN) δ -126.2, -130.5, -134.9, -139.3, -143.6, -148.0, -152.4.

^{19}F NMR (376 MHz, CD$_3$CN) δ -66.6, -68.5.

HRMS-ESI(+) Calcd. for $C_{13}H_{14}NO^+$ [M]$^+$: 200.1075; Found: 200.1086.

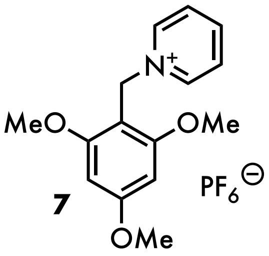

7

Following General Procedure I: **DiPyM 2PF$_6$** (92 mg, 0.20 mmol), 1,3,5-trimethoxybenzene (67 mg, 0.4 mmol, 2.0 equiv), and [Ir(ppy)$_2$(dtbbpy)]PF$_6$ (1.8 mg, 2.0 μmol, 1 mol%) were combined and irradiated with 455 nm LEDs for 16 h. Purification by column chromatography (MeCN/DCM = 1:9) afforded **7** as a white microcrystalline solid (77 mg, 95%).

Catalyst-free reaction. **DiPyPF$_6$ 2PF$_6$** (92.4 mg, 0.2 mmol) and 1,3,5-trimethoxybenzene (363 mg, 2.0 mmol, 10.0 equiv.) were combined and irradiated with 455 nm LEDs for 16 h. Purification by column chromatography (MeCN/DCM = 1:9) afforded **7** as a white microcrystalline solid (58.0 mg, 72%).

^{1}H NMR (400 MHz, CD$_3$CN) δ 8.88 – 8.67 (m, 2H), 8.42 (tt, *J* = 7.8, 1.4 Hz, 1H), 7.94 (t, *J* = 7.0 Hz, 2H), 6.25 (s, 2H), 5.59 (s, 2H), 3.87 (s, 6H), 3.81 (s, 3H).

^{13}C NMR (101 MHz, CD$_3$CN) δ 164.5, 160.8, 146.5, 145.6, 129.0, 102.9, 91.7, 56.9, 56.4, 55.4.

^{31}P NMR (162 MHz, CD$_3$CN) δ -131.5, -135.9, -140.2, -144.6, -148.9, -153.3, -157.7.

^{19}F NMR (376 MHz, CD$_3$CN) δ -71.0, -73.8.

HRMS-ESI(+) Calcd. for $C_{15}H_{18}NO_3^+$ $[M]^+$: 260.1281; Found: 260.1284.

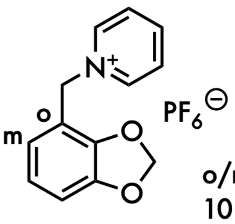

o/m
10:1

8

Following General Procedure I: **DiPyM 2PF$_6$** (92 mg, 0.20 mmol), benzo[*d*][1,3]dioxole (48.9 mg, 0.4 mmol, 46 μL, ρ = 1.06 g/cm^3, 2.0 equiv), and [Ir(ppy)$_2$(dtbbpy)]PF$_6$ (1.8 mg, 2.0 μmol, 1 mol%) were combined and irradiated with 455 nm LEDs for 16 h. Purification by column chromatography (MeCN/DCM = 1:9) afforded an inseparable mixture of regioisomers of **8** as a white microcrystalline solid (55 mg, 77%).

^{1}H NMR (400 MHz, CD$_3$CN) (major regioisomer) δ 8.74 – 8.68 (m, 2H), 8.50 (tt, *J* = 7.8, 1.4 Hz, 1H), 8.01 (t, *J* = 7.0 Hz, 2H), 7.00 (dd, *J* = 7.9, 1.9 Hz, 1H), 6.93 (d, *J* = 1.7 Hz, 1H), 6.91 (d, *J* = 7.9 Hz, 1H), 6.00 (s, 2H), 5.59 (s, 2H).

^{13}C NMR (101 MHz, CD$_3$CN) (major regioisomer) δ 149.9, 149.4, 147.0, 145.0, 129.3, 126.9, 124.8, 110.1, 109.6, 102.9, 65.1.

^{31}P NMR (162 MHz, CD_3CN) δ -131.5, -135.9, -140.2, -144.6, -149.0, -153.3, -157.7.

^{19}F NMR (376 MHz, CD_3CN) δ -71.7, -73.6.

HRMS-ESI(+) Calcd. for $C_{13}H_{12}NO_2^+$ $[M]^+$: 214.0868; Found: 214.0861.

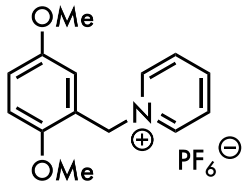

9

Following General Procedure I: **DiPyM 2PF$_6$** (92 mg, 0.20 mmol), 1,4-dimethoxybenzene (55 mg, 0.4 mmol, 2.0 equiv), and $[Ir(ppy)_2(dtbbpy)]PF_6$ (1.8 mg, 2.0 μmol, 1 mol%) were combined and irradiated with 455 nm LEDs for 16 h. Purification by column chromatography (MeCN/DCM = 1:9) afforded **9** as a white microcrystalline solid (70 mg, 93%).

^1H NMR (400 MHz, CD_3CN) δ 8.83 – 8.72 (m, 2H), 8.47 (tt, J = 7.8, 1.4 Hz, 1H), 8.03 – 7.91 (m, 2H), 7.14 (d, J = 2.9 Hz, 1H), 7.01 (dd, J = 9.0, 2.9 Hz, 1H), 6.96 (d, J = 9.0 Hz, 1H), 5.60 (s, 2H), 3.78 (s, 3H), 3.74 (s, 3H).

^{13}C NMR (101 MHz, $CDCl_3$) δ 154.7, 153.1, 147.0, 145.8, 145.6, 129.1, 122.7, 117.3, 113.6, 61.9, 56.7, 56.5.

^{31}P NMR (162 MHz, $CDCl_3$) δ -126.2, -130.5, -134.9, -139.3, -143.6, -148.0, -152.4.

^{19}F NMR (376 MHz, CD_3CN) δ -66.4, -68.3.

HRMS-ESI(+) Calcd. for $C_{14}H_{16}NO_2^+$ $[M]^+$: 230.1181; Found: 230.1183.

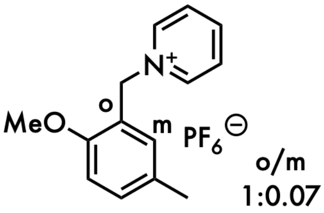

10

Following General Procedure I: **DiPyM 2PF$_6$** (92 mg, 0.20 mmol), 1-methoxy-4-methylbenzene (49 mg, 0.4 mmol, 50 μL, ρ = 0.97 g/cm^3, 2.0 equiv), and $[Ir(ppy)_2(dtbbpy)]PF_6$ (1.8 mg, 2.0 μmol, 1 mol%) were combined and irradiated with 455 nm LEDs for 16 h. Purification by column chromatography (MeCN/DCM = 1:9) afforded an inseparable mixture of regioisomers of **10** as a white microcrystalline solid (53 mg, 74%).

^1H NMR (400 MHz, CD_3CN) (major regioisomer) δ 8.80 – 8.73 (m, 2H), 8.46 (tt, J = 7.8, 1.4 Hz, 1H), 7.97 (t, J = 7.0 Hz, 2H), 7.35 (d, J = 2.3 Hz, 1H), 7.30 – 7.24 (m, 1H), 6.92 (d, J = 8.4 Hz, 1H), 5.60 (s, 2H), 3.76 (s, 3H), 2.30 (s, 3H).

^{13}C NMR (101 MHz, CD_3CN) (major regioisomer) δ 157.0, 146.9, 145.6, 133.3, 133.1, 131.5, 129.0, 121.7, 112.5, 62.0, 56.3, 20.3.

^{31}P NMR (162 MHz, CD_3CN) δ -131.4, -135.8, -140.2, -144.5, -148.9, -153.2, -157.6.

^{19}F NMR (376 MHz, CD_3CN) δ -71.7, -73.6.

HRMS-ESI(+) Calcd. for $C_{14}H_{16}NO^+$ $[M]^+$: 214.1232; Found: 214.1226.

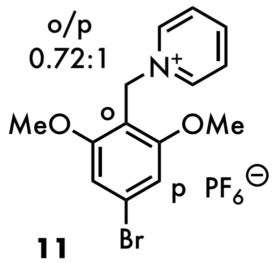

11

Following General Procedure I: **DiPyM 2PF$_6$** (92 mg, 0.20 mmol), 1-bromo-3,5-dimethoxybenzene (87 mg, 0.4 mmol, 2.0 equiv), and $[Ir(ppy)_2(dtbbpy)]PF_6$ (1.8 mg, 2.0 μmol, 1 mol%) were combined and irradiated with 455 nm LEDs for 16 h. Purification by column chromatography (MeCN/DCM = 1:9) afforded an inseparable mixture of regioisomers of **11** as a white microcrystalline solid (90 mg, 99%).

^1H NMR (400 MHz, CD_3CN) (mixture of regioisomers) δ 8.74 (ddd, J = 7.0, 6.0, 1.5 Hz, 3H), 8.45 (dtt, J = 15.7, 7.8, 1.4 Hz, 2H), 7.95 (dt, J = 14.3, 7.0 Hz, 3H), 6.92 (d, J = 2.4 Hz, 2H), 6.62 (d, J = 2.3 Hz, 1H), 5.77 (s, 2H), 5.62 (s, 2H), 3.88 (s, 4H), 3.83 (d, J = 2.3 Hz, 6H).

^{13}C NMR (101 MHz, CD_3CN) (mixture of regioisomers) δ 163.9, 161.4, 160.2, 146.9, 146.6, 145.7, 145.4, 129.1, 129.0, 127.4, 126.3, 118.3, 114.1, 111.1, 110.6, 109.6, 109.0, 100.5, 99.4, 60.3, 57.3, 57.1, 56.7, 56.3, 54.8.

^{31}P NMR (162 MHz, CD_3CN) δ -126.3, -130.6, -135.0, -139.3, -143.7, -148.1, -152.4.

^{19}F NMR (376 MHz, $CDCl_3$) δ -66.7, -68.5.

HRMS-ESI(+) Calcd. for $C_{14}H_{15}BrNO_2^+$ $[M]^+$: 308.0286; Found: 308.0277.

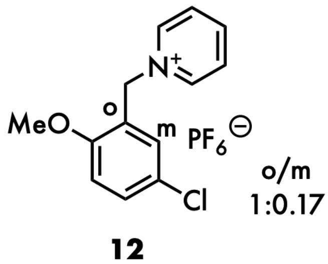

12

Following General Procedure I: **DiPyM 2PF$_6$** (92 mg, 0.20 mmol), 1-chloro-4-methoxybenzene (143 mg, 1.0 mmol, 123 μL, ρ = 1.16 g/cm^3, 5.0 equiv), and $[Ir(ppy)_2(dtbbpy)]PF_6$ (1.8 mg, 2.0 μmol, 1 mol%) were combined and irradiated with 455 nm LEDs for 16 h. Purification by column chromatography (MeCN/DCM = 1:9) afforded an inseparable mixture of regioisomers of **12** as a white microcrystalline solid (68 mg, 90%).

^{1}H NMR (400 MHz, CD_3CN) (major regioisomer) δ 8.78 – 8.74 (m, 2H), 8.48 (tt, *J* = 7.8, 1.4 Hz, 1H), 8.03 – 7.95 (m, 2H), 7.57 (d, *J* = 2.7 Hz, 1H), 7.45 (dd, *J* = 8.8, 2.6 Hz, 1H), 7.01 (d, *J* = 8.9 Hz, 1H), 5.61 (s, 2H), 3.78 (s, 3H).

^{13}C NMR (101 MHz, CD_3CN) (major regioisomer) δ 157.9, 146.9, 145.6, 132.4, 132.1, 129.0, 126.3, 123.5, 114.1, 61.1, 56.6.

^{31}P NMR (162 MHz, CD_3CN) δ -131.5, -135.9, -140.2, -144.6, -148.9, -153.3, -157.7.

^{19}F NMR (376 MHz, CD_3CN) δ -71.7, -73.6.

HRMS-ESI(+) Calcd. for $C_{13}H_{13}ClNO^+$ $[M]^+$: 234.0686; Found: 234.0681.

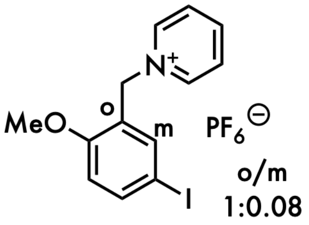

13

Following **General Procedure I**: **DiPyM 2PF₆** (92 mg, 0.20 mmol), 1-iodo-4-methoxybenzene (94 mg, 0.4 mmol, 54 µL, ρ = 1.72 g/cm^3, 2.0 equiv), and $[Ir(ppy)_2(dtbbpy)]PF_6$ (1.8 mg, 2.0 µmol, 1 mol%) were combined and irradiated with 455 nm LEDs for 16 h. Purification by column chromatography (MeCN/DCM = 1:9) afforded an inseparable mixture of regioisomers of **13** as a white microcrystalline solid (67 mg, 71%).

Scale-up procedure (chromatography-free). The reaction was performed on a 20-fold scale using four 455 nm LED modules in a 250 mL Erlenmeyer flask (150 mL MeCN). The reaction mixture was transferred to a round-bottom flask and the solvent was removed with a rotary evaporator. The crude mixture was diluted with water (15 mL) and sonicated in an ultrasound bath for 5 min. The suspension was filtered, and the filter cake was washed with water (2x15 mL) and petroleum ether (5x15 mL). The resulting solid was dried under vacuum for 16 hours to provide the corresponding pyridinium salt as a tan solid (1.73 g, 92%)

^{1}H NMR (400 MHz, CD_3CN) (major regioisomer) δ 8.79 – 8.72 (m, 2H), 8.47 (tt, *J* = 7.8, 1.4 Hz, 1H), 8.02 – 7.94 (m, 2H), 7.87 (d, *J* = 2.3 Hz, 1H), 7.77 (dd, *J* = 8.7, 2.3 Hz, 1H), 6.85 (d, *J* = 8.7 Hz, 1H), 5.57 (s, 2H), 3.77 (s, 3H).

^{13}C NMR (101 MHz, CD_3CN)) (major regioisomer) δ 159.2, 147.0, 145.7, 141.6, 140.9, 129.1, 124.5, 115.1, 83.0, 61.0, 56.6.

^{31}P NMR (162 MHz, CD_3CN) δ -131.5, -135.9, -140.2, -144.6, -149.0, -153.3, -157.7.

^{19}F NMR (376 MHz, CD_3CN) δ -71.8, -73.6.

HRMS-ESI(+) Calcd. for $C_{13}H_{13}INO^+$ $[M]^+$: 326.0042; Found: 326.0045.

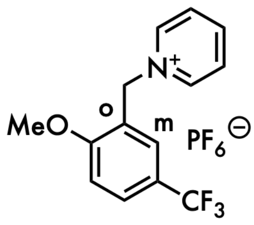

14

Following **General Procedure I**: **DiPyM 2PF$_6$** (92 mg, 0.20 mmol), 1-methoxy-4-(trifluoromethyl)benzene (147 mg, 1.0 mmol, 147 µL, ρ = 1.20 g/cm^3, 5.0 equiv), and [Ir(ppy)$_2$(dtbbpy)]PF$_6$ (1.8 mg, 2.0 µmol, 1 mol%) were combined and irradiated with 455 nm LEDs for 16 h. Purification by column chromatography (MeCN/DCM = 1:9) afforded an inseparable mixture of regioisomers of **14** as a white microcrystalline solid (54 mg, 65%).

^{1}H NMR (400 MHz, CD$_3$CN) (major regioisomer) δ 8.82 – 8.75 (m, 2H), 8.49 (tt, *J* = 7.8, 1.4 Hz, 1H), 8.04 – 7.96 (m, 2H), 7.91 (d, *J* = 2.4 Hz, 1H), 7.83 – 7.76 (m, 1H), 7.18 (d, *J* = 8.7 Hz, 1H), 5.69 (s, 2H), 3.86 (s, 3H).

^{13}C NMR (101 MHz, CD$_3$CN) (major regioisomer) δ 161.9, 147.0, 145.7, 130.4, 130.3, 130.29, 130.25, 129.78, 129.75, 129.71, 129.67, 129.0, 126.5, 123.9, 123.6, 123.3, 122.9, 122.6, 122.5, 118.3, 113.0, 61.2, 56.9.

^{31}P NMR (162 MHz, CD$_3$CN) δ -131.5, -135.9, -140.2, -144.6, -149.0, -153.3, -157.7.

^{19}F NMR (376 MHz, CD$_3$CN) δ -61.9, -62.2, -71.7, -73.6.

HRMS-ESI(+) Calcd. for $C_{14}H_{13}F_3NO^+$ $[M]^+$: 268.0949; Found: 268.0950.

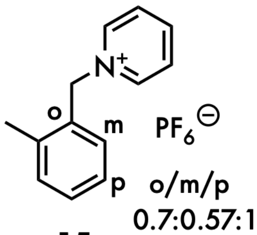

15

Following **General Procedure I**: **DiPyM 2PF$_6$** (92 mg, 0.20 mmol), toluene (51 mg, 0.4 mmol, 59 µL, ρ = 0.867 g/cm^3, 2.0 equiv), and [Ir(ppy)$_2$(dtbbpy)]PF$_6$ (1.8 mg, 2.0 µmol, 1 mol%) were combined and irradiated with 455 nm LEDs for 16 h. Purification by column chromatography (MeCN/DCM = 1:9) afforded an inseparable mixture of regioisomers of **15** as a white microcrystalline solid (58 mg, 79%).

^{1}H NMR (400 MHz, CD$_3$CN) (mixture of regioisomers) δ 8.75 – 8.68 (m, 1.2H), 8.66 – 8.58 (m, 0.8H), 8.54 – 8.43 (m, 1H), 8.05 – 7.95 (m, 2H), 7.42 – 7.34 (m, 0.45H) 7.34 – 7.28 (m, 1.5H), 7.28 – 7.24 (m, 1H), 7.24 – 7.20 (m, 1H), 5.74 (s, 1H), 5.64 (s, 1H), 2.32 (s, 1H), 2.32 (s, 1H), 2.25 (s, 1H).

^{13}C NMR (101 MHz, CD$_3$CN) δ 147.0, 147.0, 141.0, 140.4, 138.7, 133.6, 132.1, 131.3, 131.3, 131.2, 131.1, 130.9, 130.7, 130.5, 130.2, 130.0, 129.4, 129.4, 129.2, 128.1, 127.8, 127.7, 126.9, 65.3, 65.1, 63.3, 21.1, 21.0, 19.0.

^{31}P NMR (162 MHz, CD_3CN) δ -131.46, -135.83, -140.19, -144.56, -148.92, -153.29, -157.65.

^{19}F NMR (376 MHz, CD_3CN) δ -71.6, -73.5.

HRMS-ESI(+) Calcd. for $C_{13}H_{14}N^+$ $[M]^+$: 184.1121; Found: 184.1120.

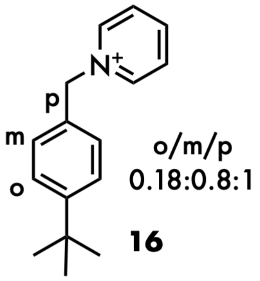

16

Following **General Procedure I**: **DiPyM 2PF₆** (92 mg, 0.20 mmol), *tert*-butylbenzene (49.32 mg, 0.4 mmol, 49 μL, ρ = 0.867 g/cm^3, 2.0 equiv), and $[Ir(ppy)_2(dtbbpy)]PF_6$ (1.8 mg, 2.0 μmol, 1 mol%) were combined and irradiated with 455 nm LEDs for 16 h. Purification by column chromatography (MeCN/DCM = 1:9) afforded an inseparable mixture of regioisomers of **16** as a white microcrystalline solid (60 mg, 81%). The assignment of regioisomers was done with the help of reference compound **R1** (**Figure S260**).

^1H NMR (400 MHz, CD_3CN) δ 8.84 – 8.67 (m, 2H), 8.57 – 8.45 (m, 1H), 8.03 (t, *J* = 7.0 Hz, 2H), 7.57 – 7.49 (m, 2H), 7.40 (dd, *J* = 8.7, 6.7 Hz, 1.6H), 7.25 (dt, *J* = 7.6, 1.4 Hz, 0.4H), 5.70 (s, 0.8H), 5.69 (s, 1H), 5.67 (s, 0.2H), 1.31 (s, 4H), 1.30 (s, 5H).

^{13}C NMR (101 MHz, CD_3CN) δ 153.9, 153.6, 147.8, 147.2, 147.0, 147.0, 146.6, 145.2, 144.9, 144.8, 144.1, 138.8, 134.3, 133.8, 133.7, 133.6, 133.4, 132.3, 131.9, 131.1, 130.9, 130.1, 129.7, 129.4, 129.4, 129.2, 129.1, 128.9, 128.5, 127.9, 127.7, 127.3, 127.3, 127.1, 123.3, 119.7, 119.5, 112.1, 111.1, 65.6, 65.0, 62.3, 35.3, 35.2, 31.4, 31.2.

^{31}P NMR (162 MHz, CD_3CN) δ -131.5, -135.8, -140.2, -144.6, -148.9, -153.3, -157.7.

^{19}F NMR (376 MHz, CD_3CN) δ -71.6, -73.5.

HRMS-ESI(+) Calcd. for $C_{16}H_{20}N^+$ $[M]^+$: 226.1596; Found: 226.1593.

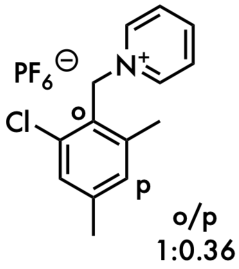

17

Following **General Procedure I**: **DiPyM 2PF₆** (92 mg, 0.20 mmol), 1-chloro-3,5-dimethylbenzene (56 mg, 0.4 mmol, 54 μL, ρ = 1.04 g/cm^3, 2.0 equiv), and $[Ir(ppy)_2(dtbbpy)]PF_6$ (1.8 mg, 2.0 μmol, 1 mol%) were combined and irradiated with 455 nm LEDs for 16 h. Purification by column chromatography (MeCN/DCM = 1:9)

afforded an inseparable mixture of regioisomers of **17** as a white microcrystalline solid (58 mg, 77%).

^{1}H NMR (400 MHz, CD_3CN) (major regioisomer) δ 8.62 – 8.55 (m, 2H), 8.51 – 8.46 (m, 1H), 8.01 (dd, *J* = 8.5, 5.5 Hz, 3H), 7.28 (s, 2H), 5.86 (s, 2H), 2.37 (s, 3H), 2.34 (s, 3H).

^{13}C NMR (101 MHz, CD_3CN) (mixture of regioisomers) δ 147.1, 147.0, 144.7, 144.4, 143.7, 142.7, 142.3, 136.7, 136.4, 131.9, 129.7, 129.5, 129.4, 129.3, 127.5, 125.5, 118.2, 59.8, 59.1, 20.9, 20.1, 19.7.

^{31}P NMR (162 MHz, CD_3CN) δ -131.5, -135.9, -140.2, -144.6, -149.0, -153.3, -157.7.

^{19}F NMR (376 MHz, CD_3CN) δ -71.8, -73.7.

HRMS-ESI(+) Calcd. for $C_{14}H_{15}ClN^+$ $[M]^+$: 232.0888; Found: 232.0893.

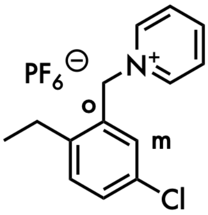

o/m
1:0.26

18

Following General Procedure I: **DiPyM 2PF$_6$** (92 mg, 0.20 mmol), 1-chloro-4-ethylbenzene (110 mg, 1.0 mmol, 105 µL, ρ = 1.11 g/cm^3, 5.0 equiv), and $[Ir(ppy)_2(dtbbpy)]PF_6$ (1.8 mg, 2.0 µmol, 1 mol%) were combined and irradiated with 455 nm LEDs for 16 h. Purification by column chromatography (MeCN/DCM = 1:9) afforded **18** as a white microcrystalline solid, regioisomeric mixture (43.0 mg, 62%).

^{1}H NMR (400 MHz, CD_3CN) (mixture of regioisomers) δ 8.73 (d, *J* = 5.5 Hz, 2H), 8.65 (d, *J* = 5.6 Hz, 1H), 8.54 (td, *J* = 7.8, 3.9 Hz, 1H), 8.03 (t, *J* = 7.1 Hz, 3H), 7.48 – 7.44 (m, 1H), 7.43 (d, *J* = 1.6 Hz, 2H), 7.35 (dd, *J* = 8.2, 2.1 Hz, 1H), 5.79 (s, 2H), 5.75 (s, 1H), 2.67 (q, *J* = 7.6 Hz, 2H), 1.22 (t, *J* = 7.6 Hz, 3H), 1.09 (t, *J* = 7.5 Hz, 1H).

^{13}C NMR (101 MHz, CD_3CN) (mixture of regioisomers) δ 147.5, 145.9, 145.7, 143.7, 133.1, 132.9, 132.7, 132.5, 132.3, 131.3, 131.3, 131.2, 130.9, 129.7, 129.5, 118.4, 63.4, 62.3, 28.7, 25.5, 15.7, 15.0.

^{31}P NMR (162 MHz, CD_3CN) δ -131.5, -135.9, -140.2, -144.7, -149.0, -153.3, -157.7.

^{19}F NMR (376 MHz, CD_3CN) δ -71.8, -73.7.

HRMS-ESI(+) Calcd. for $C_{14}H_{15}ClN^+$ $[M]^+$: 232.0888; Found: 232.0895.

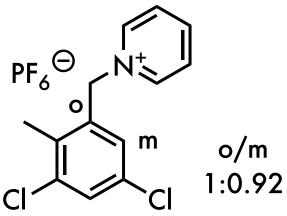

19

Following **General Procedure I**: **DiPyM 2PF$_6$** (92 mg, 0.20 mmol), 2,4-dichloro-1-methylbenzene (244 mg, 2.0 mmol, 196 µL, ρ = 1.25 g/cm^3, 10.0 equiv), and [Ir(ppy)$_2$(dtbbpy)]PF$_6$ (1.8 mg, 2.0 µmol, 1 mol%) were combined and irradiated with 455 nm LEDs for 16 h. Purification by column chromatography (MeCN/DCM = 1:9) afforded an inseparable mixture of regioisomers of **19** as a white microcrystalline solid (30 mg, 41%).

^{1}H NMR (400 MHz, CD_3CN) δ 8.70 (ddd, *J* = 9.6, 6.6, 1.4 Hz, 7H), 8.66 – 8.47 (m, 6H), 8.03 (ddd, *J* = 10.6, 8.0, 5.4 Hz, 8H), 7.59 (s, 1H), 7.48 (d, *J* = 2.2 Hz, 3H), 6.03 (s, 2H), 5.75 (s, 2H), 2.40 (s, 3H), 2.37 (s, 3H).

^{13}C NMR (101 MHz, CD_3CN) δ 148.3, 147.4, 145.5, 145.3, 142.7, 138.4, 138.1, 137.5, 137.5, 135.3, 135.0, 134.9, 133.2, 131.0, 130.2, 129.7, 129.5, 129.4, 129.4, 128.7, 128.5, 62.5, 60.9, 20.5, 19.4.

^{31}P NMR (162 MHz, CD_3CN) δ -131.5, -135.9, -140.3, -144.6, -149.0, -153.4, -157.7.

^{19}F NMR (376 MHz, CD_3CN) δ -71.8, -73.7.

HRMS-ESI(+) Calcd. for $C_{13}H_{12}Cl_2N^+$ $[M]^+$: 252.0347; Found: 252.0342.

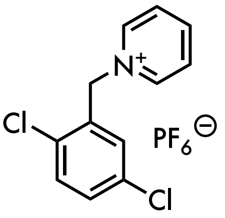

20

Following **General Procedure I**: **DiPyM 2PF$_6$** (92 mg, 0.20 mmol), 1,4-dichlorobenzene (294 mg, 2.0 mmol, 10.0 equiv), and [Ir(ppy)$_2$(dtbbpy)]PF$_6$ (1.8 mg, 2.0 µmol, 1 mol%) were combined and irradiated with 455 nm LEDs for 16 h. Purification by column chromatography (MeCN/DCM = 1:9) afforded **20** as a white microcrystalline solid (44 mg, 57%).

^{1}H NMR (400 MHz, CD_3CN) δ 8.73 (dd, *J* = 6.7, 1.5 Hz, 2H), 8.55 (tt, *J* = 7.8, 1.4 Hz, 1H), 8.09 – 8.00 (m, 2H), 7.59 (t, *J* = 1.5 Hz, 1H), 7.52 (s, 1H), 7.52 (d, *J* = 0.7 Hz, 1H), 5.79 (s, 2H).

^{13}C NMR (101 MHz, CD_3CN) δ 147.3, 145.4, 133.9, 133.7, 132.8, 132.5, 132.39, 132.36, 129.2, 62.2.

^{31}P NMR (162 MHz, CD_3CN) δ -131.5, -135.9, -140.3, -144.6, -149.0, -153.3, -157.7.

^{19}F NMR (376 MHz, CD_3CN) δ -71.7, -73.6.

HRMS-ESI(+) Calcd. for $C_{12}H_{10}Cl_2N^+$ $[M]^+$: 238.0190; Found: 238.0189.

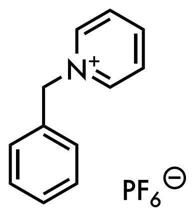

21

Following **General Procedure I**: **DiPyM 2PF$_6$** (92 mg, 0.20 mmol), benzene (31 mg, 0.4 mmol, 36 µL, ρ = 0.88 g/cm^3, 2.0 equiv), and [Ir(ppy)$_2$(dtbbpy)]PF$_6$ (1.8 mg, 2.0 µmol, 1 mol%) were combined and irradiated with 455 nm LEDs for 16 h. Purification by column chromatography (MeCN/DCM = 1:9) afforded **21** as a white microcrystalline solid (60 mg, 95%).

Scale-up procedure. When the reaction was performed on a 10-fold scale using four 455 nm LED modules in a 250 mL Erlenmeyer flask (50 mL MeCN), **21** was obtained as a white microcrystalline solid (570 mg, 90%).

^{1}H NMR (400 MHz, CD$_3$CN) δ 8.86 – 8.65 (m, 2H), 8.52 (tt, *J* = 7.8, 1.4 Hz, 1H), 8.03 (t, *J* = 7.0 Hz, 2H), 7.60 – 7.31 (m, 5H), 5.71 (s, 2H).

^{13}C NMR (101 MHz, CD$_3$CN) δ 147.2, 145.4, 133.9, 130.9, 130.6, 130.5, 130.1, 130.0, 129.6, 65.4.

^{31}P NMR (162 MHz, CD$_3$CN) δ -131.5, -135.9, -140.3, -144.6, -149.0, -153.3, -157.7.

^{19}F NMR (376 MHz, CD$_3$CN) δ -71.8, -73.7.

HRMS-ESI(+) Calcd. for $C_{12}H_{12}N^+$ $[M]^+$: 170.0970; Found: 170.0968.

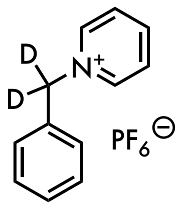

22

Following **General Procedure I**: **d2-DiPyM 2PF$_6$** (92 mg, 0.20 mmol), benzene (168 mg, 2.0 mmol, 178 µL, ρ = 0.88 g/cm^3, 10.0 equiv.), and [Ir(ppy)$_2$(dtbbpy)]PF$_6$ (1.8 mg, 2.0 µmol, 1 mol%) were combined and irradiated with 455 nm LEDs for 16 h. Purification by column chromatography (MeCN/DCM = 1:9) afforded **22** as a white microcrystalline solid (60 mg, 95%).

Note: The product obtained was used in subsequent transformations without chromatographic purification. Exposure of compound **22** to SiO$_2$ led to partial D/H exchange.

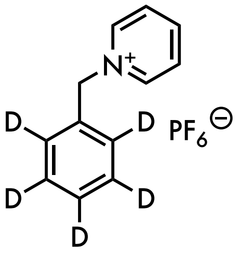

23

Following **General Procedure I**: **DiPyM 2PF$_6$** (92 mg, 0.20 mmol), C_6D_6 (168.3 mg, 2.0 mmol, 177 μL, ρ = 0.95 g/cm^3, 10.0 equiv), and [Ir(ppy)$_2$(dtbbpy)]PF$_6$ (1.8 mg, 2.0 μmol, 1 mol%) were combined and irradiated with 455 nm LEDs for 16 h. Purification by column chromatography (MeCN/DCM = 1:9) afforded **23** as a white microcrystalline solid (61 mg, 95%).

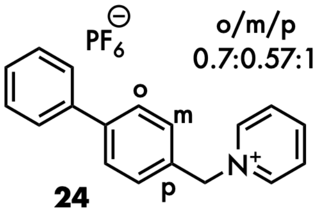

24

Following **General Procedure I**: **DiPyM 2PF$_6$** (92 mg, 0.20 mmol), biphenyl (154 mg, 1.0 mmol, 5.0 equiv), and [Ir(ppy)$_2$(dtbbpy)]PF$_6$ (1.8 mg, 2.0 μmol, 1 mol%) were combined and irradiated with 455 nm LEDs for 16 h. Purification by column chromatography (MeCN/DCM = 1:9) afforded an inseparable mixture of regioisomers of **24** as a white microcrystalline solid (63 mg, 81%). The assignment of regiosisomers were done with the help of reference compounds **R2 and R3** (**Figures S262** and **S264**).

^{1}H NMR (400 MHz, CD_3CN) (mixture of regioisomers) δ 8.79 (td, *J* = 6.6, 1.5 Hz, 2.27H), 8.52 (tt, *J* = 7.8, 1.4 Hz, 1.15H), 8.09 – 8.00 (m, 2.26H), 7.81 – 7.70 (m, 3.17H), 7.69 – 7.62 (m, 2.36H), 7.60 – 7.30 (m, 8.23H), 7.18 – 7.07 (m, 0.5H), 5.77 (s, 0.34H), 5.75 (s, 2H), 5.71 (s, 0.59H).

^{13}C NMR (101 MHz, CD_3CN) (mixture of regioisomers) δ 147.1, 146.7, 145.3, 145.1, 143.6, 143.2, 142.9, 140.6, 140.4, 140.1, 134.4, 132.8, 131.81, 131.75, 131.3, 131.1, 131.0, 130.6, 130.1, 129.9, 129.8, 129.6, 129.50, 129.48, 129.4, 129.3, 129.2, 129.0, 128.90, 128.85, 128.82, 128.78, 128.74, 128.1, 127.8, 125.8, 65.3, 65.0, 64.0.

^{31}P NMR (162 MHz, CD_3CN) δ -131.5, -135.82, -135.83, -140.2, -144.6, -148.9, -153.3, -157.6.

^{19}F NMR (376 MHz, CD_3CN) δ -71.7, -73.6.

HRMS-ESI(+) Calcd. for $C_{18}H_{16}N^+$ [M]$^+$: 246.1283; Found: 246.1284.

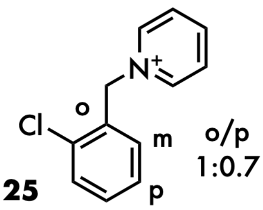

25

Following **General Procedure I**: **DiPyM 2PF$_6$** (92 mg, 0.20 mmol), chlorobenzene (225 mg, 2.0 mmol, 202 μL, ρ = 1.11 g/cm^3, 10.0 equiv), and [Ir(ppy)$_2$(dtbbpy)]PF$_6$ (1.8 mg, 2.0 μmol, 1 mol%) were combined and irradiated with 455 nm LEDs for 16 h. Purification by column chromatography (MeCN/DCM = 1:9) afforded an inseparable mixture of regioisomers of **25** as a white microcrystalline solid (51 mg, 73%). The assignment of regiosisomers were done with the help of reference compounds **R4 and R5** (**Figures S266** and **S268**).

^{1}H NMR (400 MHz, CD$_3$CN) (mixture of regioisomers) δ 8.74 (ddd, *J* = 6.7, 5.1, 1.5 Hz, 3.25H), 8.53 (tdt, *J* = 7.9, 4.2, 1.4 Hz, 1.63H), 8.04 (t, *J* = 7.1 Hz, 3.24H), 7.57 – 7.36 (m, 6.49H), 5.83 (s, 2H), 5.70 (s, 1.40H).

^{13}C NMR (101 MHz, CD$_3$CN) (mixture of regioisomers) δ 147.3, 147.2, 147.2, 145.5, 136.2, 135.7, 135.4, 135.3, 133.3, 132.9, 132.5, 131.9, 131.8, 131.2, 131.0, 130.7, 130.3, 130.0, 129.5, 129.5, 129.3, 129.0, 128.5, 64.4, 63.1.

^{31}P NMR (162 MHz, CD$_3$CN) δ -131.5, -135.9, -140.2, -144.6, -149.0, -153.3, -157.7.

^{19}F NMR (376 MHz, CD$_3$CN) δ -71.7, -73.6.

HRMS-ESI(+) Calcd. for $C_{12}H_{11}ClN^+$ [M]$^+$: 204.0580; Found: 204.0580.

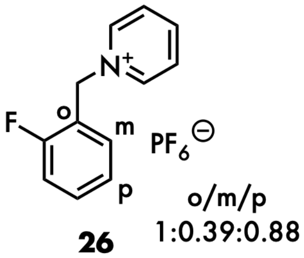

26

Following **General Procedure I**: **DiPyM 2PF$_6$** (92 mg, 0.20 mmol), fluorobenzene (192 mg, 2.0 mmol, 188 μL, ρ = 1.02 g/cm^3, 10.0 equiv), and [Ir(ppy)$_2$(dtbbpy)]PF$_6$ (1.8 mg, 2.0 μmol, 1 mol%) were combined and irradiated with 455 nm LEDs for 16 h. Purification by column chromatography (MeCN/DCM = 1:9) afforded an inseparable mixture of regioisomers of **26** as a white microcrystalline solid (29 mg, 44%).

^{1}H NMR (400 MHz, CD$_3$CN) (mixture of regioisomers) δ 8.81 – 8.69 (m, 4.6H), 8.55 – 8.49 (m, 2.32H), 8.03 (q, *J* = 6.5 Hz, 4.6H), 7.69 – 7.39 (m, 3.8H), 7.36 – 7.13 (m, 5.5H), 5.77 (s, 2H), 5.71 (s, 1.88H), 5.69 (s, 0.92H).

^{13}C NMR (101 MHz, CD$_3$CN) (mixture of regioisomers) δ 164.9, 163.3, 162.5, 160.8, 147.3, 147.1, 145.4, 136.0, 136.0, 133.6, 133.5, 132.6, 132.6, 132.5, 132.4, 132.3, 129.5, 129.4, 126.2, 126.2, 126.0, 126.0, 121.0, 120.9, 117.6, 117.4, 117.3, 117.0, 117.0, 116.8, 116.8, 64.4, 59.8.

^{31}P NMR (162 MHz, CD_3CN) δ -131.5, -135.9, -140.3, -144.6, -149.0, -153.3, -157.7.

^{19}F NMR (376 MHz, CD_3CN) δ -71.9, -73.8, -112.9, -113.1, -117.3.

HRMS-ESI(+) Calcd. for $C_{12}H_{11}FN^+$ $[M]^+$: 188.0870; Found: 188.0876.

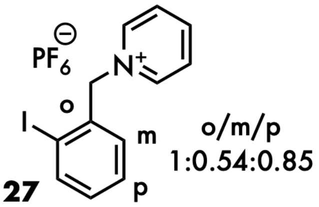

27

Following **General Procedure I**: **DiPyM 2PF₆** (92 mg, 0.20 mmol), iodobenzene (408.02 mg, 2.0 mmol, 223 μL, ρ = 1.83 g/cm^3, 10.0 equiv), and $[Ir(ppy)_2(dtbbpy)]PF_6$ (1.8 mg, 2.0 μmol, 1 mol%) were combined and irradiated with 455 nm LEDs for 16 h. Purification by column chromatography (MeCN/DCM = 1:9) afforded an inseparable mixture of regioisomers of **27** as a white microcrystalline solid (80 mg, 91%). The assignment of regiosisomers were done with the help of reference compounds **R7 and R8** (**Figures S272** and **S274**).

^1H NMR (400 MHz, CD_3CN) (mixture of regioisomers) δ 8.77 – 8.68 (m, 8.54H), 8.65 – 8.50 (m, 4.21H), 8.12 – 8.00 (m, 9.23H), 7.91 – 7.80 (m, 3H), 7.56 (td, *J* = 7.6, 1.3 Hz, 1H), 7.48 (dt, *J* = 7.8, 1.4 Hz, 0.61H), 7.43 (dd, *J* = 7.7, 1.7 Hz, 1H), 7.31 – 7.19 (m, 3.42H), 5.82 (s, 2H), 5.68 (s, 1.74H), 5.68 (s, 1.1H).

^{13}C NMR (101 MHz, CD_3CN) (mixture of regioisomers) δ 148.0, 147.2, 146.99, 146.96, 145.4, 145.0, 142.4, 141.3, 139.5, 139.1, 138.6, 135.6, 135.4, 133.3, 132.6, 132.5, 131.9, 131.7, 130.2, 129.28, 129.26, 129.1, 128.2, 100.2, 96.1, 95.0, 68.8, 64.3, 64.0.

^{31}P NMR (162 MHz, CD_3CN) δ -131.5, -135.9, -140.2, -144.6, -149.0, -153.3, -157.7.

^{19}F NMR (376 MHz, CD_3CN) δ -71.8, -73.7.

HRMS-ESI(+) Calcd. for $C_{12}H_{11}IN^+$ $[M]^+$: 295.9936; Found: 295.9933.

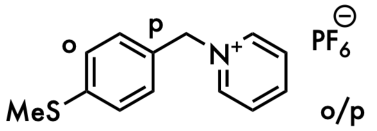

28

Following **General Procedure I**: **DiPyM 2PF₆** (92 mg, 0.20 mmol), thioanisole (47 mg, 47 μL, ρ = 1.05 g/cm^3, 0.4 mmol, 2.0 equiv), and $[Ir(ppy)_2(dtbbpy)]PF_6$ (1.8 mg, 2.0 μmol, 1 mol%) were combined and irradiated with 455 nm LEDs for 16 h. Purification by column chromatography (MeCN/DCM = 1:9) afforded an inseparable mixture of regioisomers of **28** as a white microcrystalline solid (55 mg, 76%). The assignment of regiosisomers were done with the help of reference compound **R6** (**Figure S270**).

^1H NMR (400 MHz, CD_3CN) (mixture of regioisomers) δ 8.76 – 8.63 (m, 3.51H), 8.54 – 8.46 (m, 2H), 8.06 – 7.96 (m, 3.55H), 7.55 – 7.41 (m, 3H), 7.40 – 7.29 (m, 4.38H), 5.78 (s, 2H), 5.65 (s, 1.89H), 2.49 (s, 3H), 2.46 (s, 2.6H).

^{13}C NMR (101 MHz, CD_3CN) (mixture of regioisomers) δ 186.0, 171.0, 151.7, 149.9, 146.9, 144.8, 137.0, 136.4, 135.1, 134.2, 133.9, 133.7, 133.1, 131.6, 131.5, 122.7, 69.4, 68.1, 20.7, 19.4.

^{31}P NMR (162 MHz, CD_3CN) δ -130.6, -135.0, -139.3, -143.7, -148.1.

^{19}F NMR (376 MHz, CD_3CN) δ -66.7, -68.5.

HRMS-ESI(+) Calcd. for $C_{13}H_{14}NS^+$ $[M]^+$: 216.0841; Found: 216.0838.

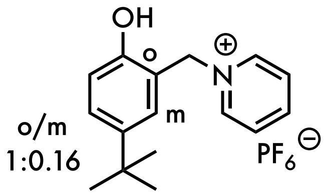

29

Following **General Procedure I**: **DiPyM 2PF$_6$** (92 mg, 0.20 mmol), 4-(*tert*-butyl)phenol (60.1 mg, 0.4 mmol, 2.0 equiv), and [Ir(ppy)$_2$(dtbbpy)]PF$_6$ (1.8 mg, 2.0 μmol, 1 mol%) were combined and irradiated with 455 nm LEDs for 16 h. Purification by column chromatography (MeCN/DCM = 1:9) afforded an inseparable mixture of regioisomers of **29** as a white microcrystalline solid (71 mg, 91%).

^{1}H NMR (400 MHz, CD_3CN) (major regioisomer) δ 8.83 – 8.72 (m, 2H), 8.46 (tt, *J* = 7.8, 1.4 Hz, 1H), 7.97 (t, *J* = 7.3 Hz, 2H), 7.53 (d, *J* = 2.4 Hz, 1H), 7.51 (s, 1H), 7.37 (dd, *J* = 8.5, 2.5 Hz, 1H), 6.86 (d, *J* = 8.6 Hz, 1H), 5.62 (s, 2H), 1.30 (s, 9H), 1.27 (s, 1H).

^{13}C NMR (101 MHz, CD_3CN) (major regioisomer) δ 153.9, 146.4, 145.1, 144.2, 129.4, 129.3, 128.6, 119.4, 115.9, 61.8, 34.4, 31.2.

^{31}P NMR (162 MHz, CD_3CN) δ -131.5, -135.9, -140.3, -144.6, -149.0, -153.4, -157.7.

^{19}F NMR (376 MHz, CD_3CN) δ -71.9, -73.8.

HRMS-ESI(+) Calcd. for $C_{16}H_{20}NO^+$ $[M]^+$: 242.1539; Found: 242.1544.

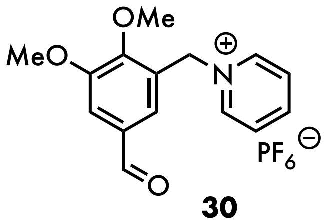

30

Following **General Procedure I**: **DiPyM 2PF$_6$** (92 mg, 0.20 mmol), 3,4-dimethoxybenzaldehyde (67 mg, 0.4 mmol, 2.0 equiv), and [Ir(ppy)$_2$(dtbbpy)]PF$_6$ (1.8 mg, 2.0 μmol, 1 mol%) were combined and irradiated with 455 nm LEDs for 16 h. Purification by column chromatography (MeCN/DCM = 1:9) afforded **30** as a white microcrystalline solid (74 mg, 92%).

^1H NMR (400 MHz, CD_3CN) δ 9.87 (s, 1H), 8.75 (dd, *J* = 6.7, 1.5 Hz, 2H), 8.49 (tt, *J* = 7.8, 1.5 Hz, 1H), 7.99 (t, *J* = 7.2 Hz, 2H), 7.59 (s, 1H), 7.28 (s, 1H), 5.97 (s, 2H), 4.00 (s, 3H), 3.96 (s, 3H).

^{13}C NMR (101 MHz, CD_3CN) δ 193.1, 154.1, 150.9, 146.5, 145.2, 128.4, 128.1, 125.6, 119.4, 117.9, 117.3, 62.1, 56.7, 56.5.

^{31}P NMR (162 MHz, CD_3CN) δ -131.5, -135.9, -140.3, -144.6, -149.0, -153.4, -157.7.

^{19}F NMR (376 MHz, CD_3CN) δ -71.9, -73.8.

HRMS-ESI(+) Calcd. for $C_{15}H_{16}NO_3^+$ $[M]^+$: 258.1125; Found: 258.1125.

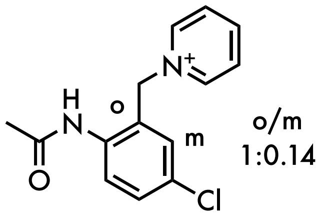

31

Following **General Procedure I**: **DiPyM 2PF$_6$** (92 mg, 0.20 mmol), *N*-(4-chlorophenyl)acetamide (170 mg, 1.0 mmol, 5.0 equiv), and $[Ir(ppy)_2(dtbbpy)]PF_6$ (1.8 mg, 2.0 μmol, 1 mol%) were combined and irradiated with 455 nm LEDs for 16 h. Purification by column chromatography (MeCN/DCM = 1:9) afforded an inseparable mixture of regioisomers of **31** as a white microcrystalline solid (33 mg, 41%).

^1H NMR (400 MHz, CD_3CN) (major regioisomer) δ 8.68 – 8.59 (m, 2H), 8.55 – 8.46 (m, 1H), 8.18 (s, 1H), 8.01 (t, *J* = 7.1 Hz, 2H), 7.49 (dd, *J* = 8.5, 2.5 Hz, 1H), 7.38 (d, *J* = 2.4 Hz, 1H), 7.31 (d, *J* = 8.5 Hz, 1H), 5.64 (s, 2H), 2.02 (s, 3H).

^{13}C NMR (101 MHz, CD_3CN) δ 171.1, 147.3, 145.6, 136.5, 133.1, 131.5, 131.4, 130.0, 129.3, 128.5, 61.5, 23.2.

^{31}P NMR (162 MHz, CD_3CN) δ -131.5, -135.9, -140.3, -144.6, -149.0, -153.4, -157.7.

^{19}F NMR (376 MHz, CD_3CN) δ -71.9, -73.8.

HRMS-ESI(+) Calcd. for $C_{14}H_{14}ClN_2O^+$ $[M]^+$: 261.0795; Found: 261.0794.

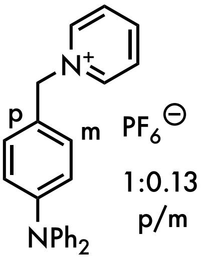

32

Following **General Procedure I**: **DiPyM 2PF$_6$** (92 mg, 0.20 mmol), triphenylamine (98.13 mg, 0.4 mmol, 2.0 equiv), and $[Ir(ppy)_2(dtbbpy)]PF_6$ (1.8 mg, 2.0 μmol, 1 mol%) were combined and irradiated with 455 nm LEDs for 16 h. Purification by column

chromatography (MeCN/DCM = 1:9) afforded **32** as a white microcrystalline solid (86.0 mg, 89%).

^{1}H NMR (400 MHz, CD_3CN) (major regioisomer) δ 8.77 – 8.68 (m, 2H), 8.50 (tt, *J* = 7.8, 1.4 Hz, 1H), 8.02 (t, *J* = 7.0 Hz, 2H), 7.37 – 7.28 (m, 6H), 7.14 – 6.96 (m, 8H), 6.78 – 6.72 (m, 1H), 5.62 (s, 2H).

^{13}C NMR (101 MHz, CD_3CN) δ 150.4, 148.0, 147.0, 145.1, 131.5, 130.6, 129.4, 126.0, 125.0, 123.2, 122.9, 118.3, 65.0.

^{31}P NMR (162 MHz, CD_3CN) δ -131.5, -135.9, -140.2, -144.6, -149.0, -153.3, -157.7.

^{19}F NMR (376 MHz, CD_3CN) δ -71.9, -73.7.

HRMS-ESI(+) Calcd. for $C_{24}H_{21}N_2^+$ $[M]^+$: 337.1699; Found: 337.1703.

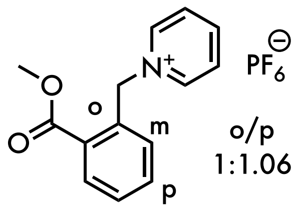

33

Following General Procedure I: **DiPyM 2PF$_6$** (92 mg, 0.20 mmol), methyl benzoate (272 mg, 2.0 mmol, 252 µL, ρ = 1.08 g/cm^3, 10.0 equiv), and $[Ir(ppy)_2(dtbbpy)]PF_6$ (1.8 mg, 2.0 µmol, 1 mol%) were combined and irradiated with 455 nm LEDs for 16 h. Purification by column chromatography (MeCN/DCM = 1:5) afforded *p*-isomer (35 mg) and *o*-isomer (33 mg) of **33** as a white microcrystalline solid (68 mg, 91%).

***o*-33**

^{1}H NMR (400 MHz, CD_3CN) (ortho isomer) δ 8.60 (d, *J* = 5.6 Hz, 2H), 8.47 – 8.34 (m, 1H), 8.05 (dd, *J* = 7.8, 1.4 Hz, 1H), 7.91 (t, *J* = 7.2 Hz, 2H), 7.65 (td, *J* = 7.6, 1.5 Hz, 1H), 7.57 (td, *J* = 7.7, 1.3 Hz, 1H), 7.47 – 7.36 (m, 1H), 5.94 (s, 2H), 3.73 (s, 3H).

^{13}C NMR (101 MHz, CD_3CN) (ortho isomer) δ 167.6, 147.1, 145.6, 134.7, 133.8, 133.7, 132.9, 131.6, 130.7, 129.1, 118.4, 63.8, 53.3.

^{19}F NMR (376 MHz, CD_3CN) δ -71.8, -73.7.

^{31}P NMR (162 MHz, CD_3CN) δ -135.9, -140.2, -144.6, -149.0, -153.3.

***p*-33**

^{1}H NMR (400 MHz, CD_3CN) δ 8.66 (d, *J* = 5.5 Hz, 2H), 8.51 – 8.38 (m, 1H), 8.00 – 7.95 (m, 3H), 7.43 (d, *J* = 8.6 Hz, 2H), 5.69 (d, *J* = 2.3 Hz, 2H), 3.80 (s, 3H).

^{13}C NMR (101 MHz, CD_3CN) δ 166.6, 147.1, 145.3, 138.1, 134.3, 132.1, 130.8, 129.8, 129.3, 64.4, 52.6.

^{19}F NMR (376 MHz, CD_3CN) δ -71.9, -73.8.

^{31}P NMR (162 MHz, CD_3CN) δ -131.5, -135.9, -140.3, -144.6, -149.0, -153.4, -157.7.

HRMS-ESI(+) Calcd. for $C_{14}H_{14}NO_2^+$ $[M]^+$: 228.1025; Found: 228.1026.

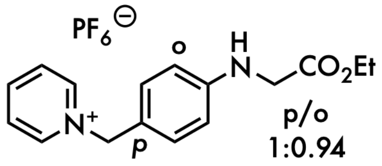

34

Following **General Procedure I**: **DiPyM 2PF$_6$** (92 mg, 0.20 mmol), ethyl phenylglycinate (125.7 mg, 0.7 mmol, 3.5 equiv), and [Ir(ppy)$_2$(dtbbpy)]PF$_6$ (1.8 mg, 2.0 µmol, 1 mol%) were combined and irradiated with 455 nm LEDs for 16 h. Purification by column chromatography (MeCN/DCM = 1:9) afforded **34** as a white microcrystalline solid (80 mg, 96%).

^{1}H NMR (400 MHz, CD$_3$CN) (mixture of regioisomers) δ 8.61 (d, *J* = 5.5 Hz, 2H), 8.56 (d, *J* = 5.6 Hz, 2H), 8.47 – 8.27 (m, 2H), 7.91 (dd, *J* = 9.7, 4.3 Hz, 4H), 7.32 – 7.25 (m, 1H), 7.21 (dd, *J* = 7.5, 1.4 Hz, 1H), 7.19 – 7.14 (m, 2H), 6.77 (td, *J* = 7.5, 1.0 Hz, 1H), 6.60 (d, *J* = 8.4 Hz, 1H), 6.59 – 6.53 (m, 2H), 5.59 (s, 2H), 5.46 (s, 2H), 5.00 (s, 1H), 4.66 (s, 1H), 4.06 (dq, *J* = 11.1, 7.1 Hz, 5H), 3.82 (dd, *J* = 6.1, 1.8 Hz, 4H), 1.23 – 1.04 (m, 7H).

^{13}C NMR (101 MHz, CD$_3$CN) (mixture of regioisomers) δ 171.9, 171.8, 150.3, 147.6, 147.1, 146.9, 144.9, 133.7, 132.9, 131.8, 129.4, 129.2, 121.6, 119.4, 118.4, 116.7, 113.9, 113.2, 61.9, 61.8, 45.64, 45.5, 14.5.

^{19}F NMR (376 MHz, CD$_3$CN) δ -72.07, -73.89.

^{31}P NMR (162 MHz, CD$_3$CN) δ -131.5, -135.9, -140.3, -144.6, -149.0, -153.3, -157.7.

HRMS-ESI(+) Calcd. for $C_{16}H_{19}N_2O_2^+$ [M+]: 271.1447; Found: 271.1441.

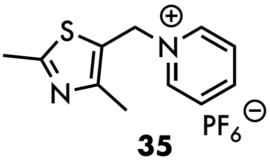

35

Following **General Procedure I**: **DiPyM 2PF$_6$** (92 mg, 0.20 mmol), 2,4-dimethylthiazole (45 mg, 0.4 mmol, 43 µL, ρ = 1.06 g/cm^3, 2.0 equiv), and [Ir(ppy)$_2$(dtbbpy)]PF$_6$ (1.8 mg, 2.0 µmol, 1 mol%) were combined and irradiated with 455 nm LEDs for 16 h. Purification by column chromatography (MeCN/DCM = 1:9) afforded **35** as a white microcrystalline solid (35 mg, 50%).

^{1}H NMR (400 MHz, CD$_3$CN) δ 8.70 – 8.60 (m, 2H), 8.52 (tt, *J* = 7.8, 1.4 Hz, 1H), 8.02 (t, *J* = 7.1 Hz, 2H), 5.82 (s, 2H), 2.63 (s, 3H), 2.42 (s, 3H).

^{13}C NMR (101 MHz, CD$_3$CN) δ 168.9, 155.7, 147.3, 144.7, 129.5, 121.3, 56.7, 19.4, 15.3.

^{31}P NMR (162 MHz, CD$_3$CN) δ -131.5, -135.9, -140.2, -144.6, -148.9, -153.3, -157.7.

^{19}F NMR (376 MHz, CD$_3$CN) δ -71.9, -73.7.

HRMS-ESI(+) Calcd. for $C_{11}H_{13}N_2S^+$ $[M]^+$: 205.0799; Found: 205.0796.

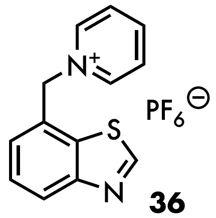

36

Following **General Procedure I**: **DiPyM 2PF₆** (92 mg, 0.20 mmol), benzo[*d*]thiazole (54.1 mg, 0.4 mmol, 2.0 equiv), and $[Ir(ppy)_2(dtbbpy)]PF_6$ (1.8 mg, 2.0 µmol, 1 mol%) were combined and irradiated with 455 nm LEDs for 16 h. Purification by column chromatography (MeCN/DCM = 1:9) afforded **36** as a white microcrystalline solid (74 mg, 99%).

^1H NMR (400 MHz, CD_3CN) δ 9.19 (s, 1H), 9.04 – 8.88 (m, 2H), 8.53 – 8.39 (m, 1H), 8.20 (dd, *J* = 8.2, 1.1 Hz, 1H), 7.97 (t, *J* = 7.1 Hz, 2H), 7.78 – 7.68 (m, 1H), 7.63 – 7.51 (m, 1H), 6.17 (s, 2H).

^{13}C NMR (101 MHz, CD_3CN) δ 157.9, 152.8, 147.0, 145.9, 135.9, 129.1, 128.6, 127.9, 126.8, 125.5, 118.2, 62.9.

^{31}P NMR (162 MHz, CD_3CN) δ -130.6, -134.9, -139.3, -143.6, -148.0.

^{19}F NMR (376 MHz, CD_3CN) δ -66.6, -68.5.

HRMS-ESI(+) Calcd. for $C_{13}H_{11}N_2S^+$ $[M]^+$: 227.0643; Found: 227.0643.

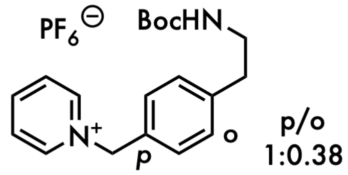

37

Following **General Procedure I**: **DiPyM 2PF₆** (92 mg, 0.20 mmol), tert-butyl phenethylcarbamate (88.5 mg, 0.4 mmol, 2.0 equiv), and $[Ir(ppy)_2(dtbbpy)]PF_6$ (1.8 mg, 2.0 µmol, 1 mol%) were combined and irradiated with 455 nm LEDs for 16 h. Purification by column chromatography (MeCN/DCM = 1:9) afforded slightly impure **37**, which was dissolved in a minimal amount of MeCN (~1mL), precipitated out with water (10 mL), and filtered to give a white microcrystalline solid (84 mg, 92%).

^1H NMR (400 MHz, CD_3CN) (mixture of regioisomers) δ 8.76 (d, *J* = 5.6 Hz, 2.49H), 8.54 (t, *J* = 7.8 Hz, 1.28H), 8.05 (t, *J* = 7.0 Hz, 2.51H), 7.54 – 7.42 (m, 0.89H), 7.39 (q, *J* = 5.1 Hz, 1.82H), 7.33 (d, *J* = 8.2 Hz, 2.19H), 7.31 – 7.18 (m, 0.94H), 5.88 (s, 0.67H), 5.70 (s, 2H), 5.51 (s, 0.33H), 5.32 (s, 1H), 3.28 (q, *J* = 6.9 Hz, 2.02H), 3.22 – 3.10 (m, 0.67H), 2.78 (tt, *J* = 8.2, 4.1 Hz, 2.79H), 1.42 (s, 3.71H), 1.37 (s, 9.03H).

^{13}C NMR (101 MHz, CD_3CN) (mixture of regioisomers) δ 147.2, 146.4, 145.4, 136.8, 132.2, 131.4, 131.0, 130.9, 130.5, 130.2, 129.6, 129.5, 128.6, 128.0, 123.4, 79.2, 65.2, 62.9, 49.3, 42.3, 40.3, 36.6, 36.2, 33.9, 28.6, 26.5.

^{19}F NMR (376 MHz, CD_3CN) δ -71.8, -73.7.

^{31}P NMR (162 MHz, CD_3CN) δ -131.5, -135.9, -140.3, -144.6, -149.0, -153.3, -157.7.

HRMS-ESI(+) Calcd. for $C_{19}H_{25}N_2O_2^+$ [M+]: 313.1916; Found: 313.1959.

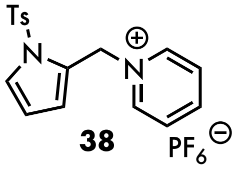

38

Following **General Procedure I**: **DiPyM 2PF₆** (92 mg, 0.20 mmol), 1-tosyl-1*H*-pyrrole (49 mg, 0.22 mmol, 1.1 equiv), and $[Ir(ppy)_2(dtbbpy)]PF_6$ (1.8 mg, 2.0 μmol, 1 mol%) were combined and irradiated with 455 nm LEDs for 16 h. Purification by column chromatography (MeCN/DCM = 1:9) afforded **38** as a white microcrystalline solid (89 mg, 97%).

^1H NMR (400 MHz, CD_3CN) δ 8.66 – 8.56 (m, 2H), 8.48 (tt, *J* = 7.8, 1.4 Hz, 1H), 7.98 – 7.90 (m, 2H), 7.62 – 7.57 (m, 2H), 7.54 (dd, *J* = 3.4, 1.7 Hz, 1H), 7.41 – 7.27 (m, 2H), 6.90 – 6.74 (m, 1H), 6.50 (t, *J* = 3.4 Hz, 1H), 5.82 (s, 2H), 2.39 (s, 3H).

^{13}C NMR (101 MHz, CD_3CN) δ 147.5, 147.3, 144.7, 135.7, 131.4, 129.0, 127.5, 127.3, 124.5, 123.3, 113.8, 57.1, 21.5.

^{31}P NMR (162 MHz, CD_3CN) δ -131.5, -135.8, -140.2, -144.5, -148.9, -153.3, -157.6.

^{19}F NMR (376 MHz, CD_3CN) δ -71.7, -73.6.

HRMS-ESI(+) Calcd. for $C_{17}H_{17}N_2O_2S^+$ $[M]^+$: 313.1011; Found: 313.1012.

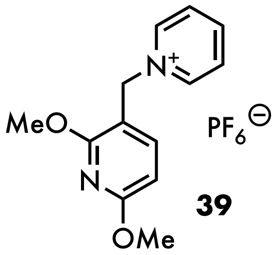

39

Following **General Procedure I**: **DiPyM 2PF₆** (92 mg, 0.20 mmol), 2,6-dimethoxypyridine (56 mg, 0.4 mmol, 53.0 μL, ρ = 1.05 g/cm³, 2.0 equiv), and $[Ir(ppy)_2(dtbbpy)]PF_6$ (1.8 mg, 2.0 μmol, 1 mol%) were combined and irradiated with 455 nm LEDs for 16 h. Purification by column chromatography (MeCN/DCM = 1:9) afforded **39** as a white microcrystalline solid (58 mg, 77%).

^1H NMR (400 MHz, CD_3CN) δ 8.81 – 8.68 (m, 2H), 8.47 (tt, *J* = 7.8, 1.4 Hz, 1H), 7.98 (t, *J* = 7.0 Hz, 2H), 7.80 (d, *J* = 8.1 Hz, 1H), 6.42 (dd, *J* = 8.1, 0.7 Hz, 1H), 5.54 (s, 2H), 3.90 (s, 3H), 3.89 (s, 3H).

^{13}C NMR (101 MHz, CD_3CN) δ 165.5, 162.3, 146.8, 144.2, 129.0, 107.1, 102.4, 60.9, 54.4, 54.3.

^{31}P NMR (162 MHz, CD3CN) δ -131.5, -135.8, -140.2, -144.6, -148.9, -153.3, -157.6.

^{19}F NMR (376 MHz, CD3CN) δ -71.8, -73.7.

HRMS-ESI(+) Calcd. for $C_{13}H_{15}N_2O_2^+$ $[M]^+$: 231.1134; Found: 231.1132.

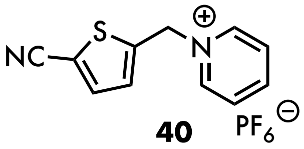

40

Following **General Procedure I**: **DiPyM 2PF$_6$** (92 mg, 0.20 mmol), thiophene-2-carbonitrile (44 mg, 0.4 mmol, 37 μL, ρ = 1.17 g/cm^3, 2.0 equiv), and [Ir(ppy)$_2$(dtbbpy)]PF$_6$ (1.8 mg, 2.0 μmol, 1 mol%) were combined and irradiated with 455 nm LEDs for 16 h. Purification by column chromatography (MeCN/DCM = 1:9) afforded **40** as a white microcrystalline solid (68 mg, 98%).

^{1}H NMR (400 MHz, CD_3CN) δ 8.70 – 8.60 (m, 2H), 8.56 – 8.47 (m, 1H), 8.18 (s, 1H), 8.02 (dt, *J* = 14.2, 6.3 Hz, 3H), 7.38 (d, *J* = 2.4 Hz, 1H), 7.31 (d, *J* = 8.5 Hz, 1H), 5.64 (s, 2H), 2.02 (s, 3H).

^{13}C NMR (101 MHz, CD_3CN) δ 147.5, 144.9, 141.7, 139.5, 132.1, 129.4, 113.9, 112.7, 58.4.

^{19}F NMR (376 MHz, CD_3CN) δ -71.8, -73.6.

^{31}P NMR (162 MHz, CD_3CN) δ -131.5, -135.9, -140.2, -144.6, -149.0, -153.3, -157.7.

HRMS-ESI(+) Calcd. for $C_{11}H_9N_2S^+$ $[M]^+$: 201.0486; Found: 201.0484.

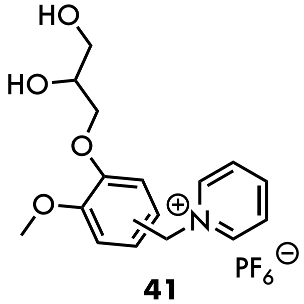

41

Following **General Procedure I**: **DiPyM 2PF$_6$** (92 mg, 0.20 mmol), 3-(2-methoxyphenoxy)propane-1,2-diol (79 mg, 0.40 mmol, 2.0 equiv), and [Ir(ppy)$_2$(dtbbpy)]PF$_6$ (1.8 mg, 2.0 μmol, 1 mol%) were combined and irradiated with 455 nm LEDs for 16 h. Reaction yield was determined by ^{1}H NMR spectroscopy using internal standard (1,3,5-trimethoxybenzene). An aliquot (300 μL) of the reaction mixture was withdrawn by syringe and transferred to a 5.0 mL round-bottom flask. Solvent was removed under reduced pressure, after which CD_3CN (0.50 mL) was added. The mixture was sonicated for 10 s and transferred to an NMR tube for analysis. Determined NMR yield: 90%.

HRMS-ESI(+) Calcd. for $C_{16}H_{20}NO_4^+$ $[M]^+$: 290.1392; Found: 290.1385.

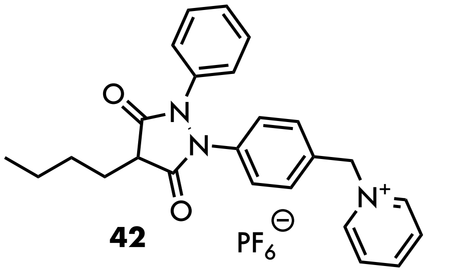

42

Following **General Procedure I**: **DiPyM 2PF$_6$** (92 mg, 0.20 mmol), 4-butyl-1,2-diphenylpyrazolidine-3,5-dione (123 mg, 0.40 mmol, 2.0 equiv), and [Ir(ppy)$_2$(dtbbpy)]PF$_6$ (1.8 mg, 2.0 μmol, 1 mol%) were combined and irradiated with 455 nm LEDs for 16 h. Purification by column chromatography (MeCN/DCM = 1:9) afforded **42** as a white microcrystalline solid (52 mg, 48%).

^{1}H NMR (400 MHz, CD$_3$CN) δ 8.67 (ddd, *J* = 6.6, 4.1, 1.4 Hz, 2H), 8.63 (dt, *J* = 7.9, 1.4 Hz, 1H), 8.16 – 8.03 (m, 2H), 7.38 – 7.32 (m, 4H), 7.30 – 7.26 (m, 2H), 7.16 – 7.12 (m, 4H), 5.07 (s, 2H), 2.28 (s, 1H), 2.09 – 2.03 (m, 2H), 1.47 – 1.37 (m, 4H), 0.92 (t, *J* = 7.0 Hz, 3H).

^{13}C NMR (101 MHz, CD$_3$CN) δ 169.7, 148.9, 146.5, 135.1, 130.2, 129.9, 129.8, 129.2, 125.0, 124.6, 63.1, 55.9, 34.6, 27.0, 23.1, 13.8.

^{31}P NMR (162 MHz, CD$_3$CN) δ -131.5, -135.9, -140.2, -144.6, -149.0, -153.3, -157.7.

^{19}F NMR (376 MHz, CD$_3$CN) δ -71.9, -73.7.

HRMS-ESI(+) Calcd. for $C_{25}H_{26}N_3O_2^+$ [M]$^+$: 400.2020; Found: 400.2016.

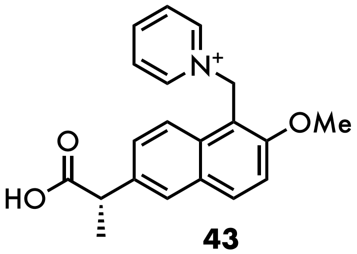

43

Following **General Procedure I**: **DiPyM 2PF$_6$** (92 mg, 0.20 mmol), (*S*)-2-(6-methoxynaphthalen-2-yl)propanoic acid (70 mg, 0.30 mmol, 1.5 equiv), and [Ir(ppy)$_2$(dtbbpy)]PF$_6$ (1.8 mg, 2.0 μmol, 1 mol%) were combined and irradiated with 455 nm LEDs for 16 h. Purification by column chromatography (MeCN/DCM = 1:9) afforded **43** as a white microcrystalline solid (66 mg, 80%).

^{1}H NMR (400 MHz, CD$_3$CN) δ 8.78 – 8.65 (m, 2H), 8.43 (tt, *J* = 7.8, 1.4 Hz, 1H), 8.07 (d, *J* = 9.2 Hz, 1H), 8.02 (d, *J* = 8.8 Hz, 1H), 7.93 (t, *J* = 7.1 Hz, 2H), 7.85 (d, *J* = 1.9 Hz, 1H), 7.59 (dd, *J* = 8.8, 2.0 Hz, 1H), 7.48 (d, *J* = 9.2 Hz, 1H), 6.11 (s, 2H), 3.96 (s, 3H), 3.92 – 3.88 (m, 1H), 1.53 (s, 3H).

^{13}C NMR (101 MHz, CD$_3$CN) δ 180.3, 162.5, 151.2, 149.6, 142.2, 138.3, 137.0, 134.4, 133.8, 133.54, 133.50, 132.6, 127.2, 118.8, 117.4, 61.6, 60.9, 49.7, 23.0.

^{31}P NMR (162 MHz, CDCl$_3$) δ -126.2, -130.6, -134.9, -139.3, -143.7, -148.0, -152.4.

^{19}F NMR (376 MHz, $CDCl_3$) δ -66.5, -68.4.

HRMS-ESI(+) Calcd. for $C_{20}H_{20}NO_3^+$ $[M]^+$: 322.1443; Found: 322.1443.

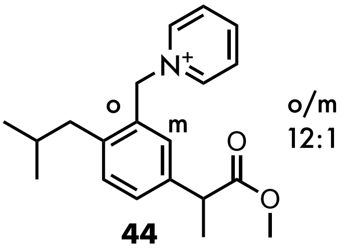

44

Following **General Procedure I**: **DiPyM 2PF$_6$** (92 mg, 0.20 mmol), methyl 2-(4-isobutylphenyl)propanoate (88 mg, 0.40 mmol, 2.0 equiv), and $[Ir(ppy)_2(dtbbpy)]PF_6$ (1.8 mg, 2.0 µmol, 1 mol%) were combined and irradiated with 455 nm LEDs for 16 h. Purification by column chromatography (MeCN/DCM = 1:9) afforded an inseparable mixture of regioisomers of **44** as a white microcrystalline solid (31 mg, 34%).

^1H NMR (400 MHz, CD_3CN) (mixture of regioisomers) δ 8.62 (td, *J* = 6.5, 1.4 Hz, 2H), 8.53 (tq, *J* = 7.7, 1.7 Hz, 1H), 8.03 (td, *J* = 6.9, 4.7 Hz, 2H), 7.42 – 7.30 (m, 2H), 7.13 (d, *J* = 1.9 Hz, 1H), 5.75 (s, 2H), 3.74 (q, *J* = 7.2 Hz, 1H), 3.59 (d, *J* = 0.7 Hz, 2H), 2.49 (d, *J* = 7.3 Hz, 2H), 1.94 (td, *J* = 2.5, 0.6 Hz, 1H), 1.42 – 1.39 (m, 3H), 0.87 (dd, *J* = 9.2, 6.6 Hz, 6H).

^{13}C NMR (101 MHz, CD_3CN) δ 171.1, 147.3, 145.6, 136.5, 133.1, 131.53, 131.47, 131.4, 130.0, 129.4, 129.3, 128.5, 122.9, 61.5, 23.2.

^{31}P NMR (162 MHz, CD_3CN) δ -131.52, -135.89, -140.25, -144.61, -148.98, -153.34, -157.70.

^{19}F NMR (376 MHz, CD_3CN) δ -71.9, -73.7.

HRMS-ESI(+) Calcd. for $C_{20}H_{26}NO_2^+$ $[M]^+$: 312.1964; Found: 312.1963.

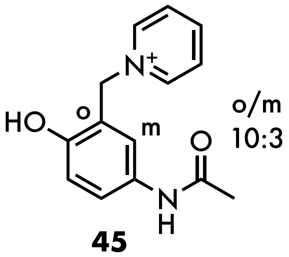

45

Following **General Procedure I**: **DiPyM 2PF$_6$** (92 mg, 0.20 mmol), *N*-(4-hydroxyphenyl)acetamide (61 mg, 0.40 mmol, 2.0 equiv), and $[Ir(ppy)_2(dtbbpy)]PF_6$ (1.8 mg, 2.0 µmol, 1 mol%) were combined and irradiated with 455 nm LEDs for 16 h. Purification by column chromatography (MeCN/DCM = 1:9) afforded an inseparable mixture of regioisomers of **45** as a white microcrystalline solid (35 mg, 45%).

^1H NMR (400 MHz, CD_3CN) (major regioisomer) δ 8.83 – 8.76 (m, 1H), 8.56 – 8.43 (m, 1H), 8.26 (s, 0H), 8.06 – 7.92 (m, 1H), 7.76 (d, *J* = 2.6 Hz, 0H), 7.39 (dd, *J* = 8.7, 2.6 Hz, 0H), 6.89 (d, *J* = 8.7 Hz, 1H), 5.64 (s, 1H), 2.06 (s, 2H).

^{13}C NMR (101 MHz, CD_3CN) (major regioisomer) δ 169.4, 152.5, 146.5, 145.1, 132.5, 128.7, 124.0, 123.7, 119.9, 116.5, 61.4, 23.7.

^{19}F NMR (376 MHz, CD_3CN) δ -71.8, -73.6.

^{31}P NMR (162 MHz, CD_3CN) δ -131.5, -135.8, -140.2, -144.6, -148.9, -153.3, -157.7.

HRMS-ESI(+) Calcd. for $C_{14}H_{15}N_2O_2^+$ $[M]^+$: 243.1128; Found: 243.1122.

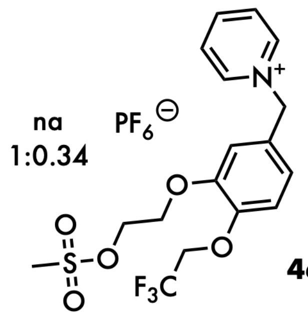

46

Following **General Procedure I**: **DiPyM 2PF6** (92 mg, 0.20 mmol), 2-(2-(2,2,2-trifluoroethoxy)phenoxy)ethyl methanesulfonate (126 mg, 0.4 mmol, 2.0 equiv), and $[Ir(ppy)_2(dtbbpy)]PF_6$ (1.8 mg, 2.0 μmol, 1 mol%) were combined and irradiated with 455 nm LEDs for 16 h. Purification by column chromatography (MeCN/DCM = 1:9) afforded an inseparable mixture of regioisomers of **46** as a white microcrystalline solid (102 mg, 93%).

^{1}H NMR (400 MHz, CD_3CN) (mixture of regioisomers) δ 8.78 – 8.69 (m, 2.76H), 8.53 (t, J = 7.8 Hz, 1.43H), 8.04 (t, J = 7.1 Hz, 2.84H), 7.22 (dd, J = 8.3, 2.1 Hz, 1.11H), 7.18 – 7.15 (m, 1.34H), 7.14 – 7.09 (m, 1.75H), 5.66 (s, 0.7H), 5.65 (s, 2H), 4.64 – 4.49 (m, 5.96H), 4.39 – 4.29 (m, 3H), 4.25 (dd, J = 5.1, 4.1 Hz, 1H), 3.12 (s, 3H), 3.07 (s, 1H).

^{13}C NMR (101 MHz, CD_3CN) (mixture of regioisomers) δ 150.3, 149.4, 147.86, 146.8, 144.9, 129.1, 128.2, 126.4, 125.8, 125.7, 123.8, 123.1, 116.8, 116.1, 115.1, 72.8, 69.44, 69.38, 67.7, 67.5, 67.2, 64.5, 60.4, 37.3, 37.1.

^{19}F NMR (376 MHz, CD_3CN) δ -71.8, -73.7, -74.7.

^{31}P NMR (162 MHz, CD_3CN) δ -131.5, -135.9, -140.22, -144.6, -149.0, -153.3, -157.7.

HRMS-ESI(+) Calcd. for $C_{17}H_{19}F_3NO_5S^+$ [M+]: 406.0936; Found: 406.0923.

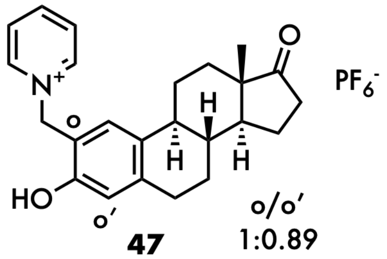

47 o/o′ 1:0.89

Following **General Procedure I**: **DiPyM 2PF$_6$** (924 mg, 2.0 mmol), estrone (650 mg, 2.4 mmol, 1.2 equiv), and [Ir(ppy)$_2$(dtbbpy)]PF$_6$ (18 mg, 20.0 μmol, 1 mol%) were dissolved in 1:1 (v/v) MeCN:1,2-DCE mixture (100 mL) and irradiated with 455 nm LEDs for 1 h. Purification by column chromatography (MeCN/DCM = 1:9) afforded a mixture of regioisomers of **47** as a white microcrystalline solid (800 mg, 79%).

The regiosisomers were separated by preparative HPLC

***o*-47**

^{1}H NMR (400 MHz, CD$_3$CN) δ 8.77 (d, J = 6.4 Hz, 2H), 8.45 (tt, J = 7.8, 1.3 Hz, 1H), 7.97 (t, J = 6.6 Hz, 2H), 7.45 (s, 1H), 7.41 (s, 1H), 6.63 (s, 1H), 5.58 (s, 2H), 2.87 – 2.79 (m, 2H), 2.48 – 2.37 (m, 2H), 2.26 – 2.18 (m, 1H), 2.12 – 1.96 (m, 3H), 1.88 – 1.81 (m, 1H), 1.64 – 1.32 (m, 6H), 0.87 (s, 3H).

^{13}C NMR (101 MHz, CD$_3$CN) δ 221.26, 154.35, 146.76, 145.43, 142.02, 133.44, 129.78, 129.04, 118.02, 116.59, 61.98, 50.98, 48.66, 44.59, 38.97, 36.32, 32.46, 29.96, 27.02, 26.70, 22.14, 14.20.

***o’*-47**

^{1}H NMR (400 MHz, CD$_3$CN) δ 8.66 (d, J = 5.8 Hz, 2H), 8.46 (tt, J = 7.7, 1.4 Hz, 1H), 7.96 (t, J = 7.0 Hz, 2H), 7.54 (s, 1H), 7.34 (d, J = 8.7 Hz, 1H), 6.81 (d, J = 8.6 Hz, 1H), 5.79 – 5.64 (m, 2H), 2.95 – 2.86 (m, 1H), 2.79 – 2.69 (m, 1H), 2.48 – 2.34 (m, 2H), 2.28 – 2.20 (m, 1H), 2.12 – 1.98 (m, 3H), 1.87 – 1.82 (m, 1H), 1.64 – 1.34 (m, 6H), 0.86 (s, 3H).

^{13}C NMR (101 MHz, CD$_3$CN) δ 221.1, 155.0, 146.7, 145.2, 139.1, 133.9, 130.0, 129.2, 117.8, 114.1, 57.5, 50.9, 48.5, 44.9, 38.2, 36.3, 32.4, 27.5, 26.9, 22.1, 14.1.

HRMS-ESI(+) Calcd. for $C_{24}H_{28}NO_2^+$ [M+]: 362.2115; Found: 362.2114.

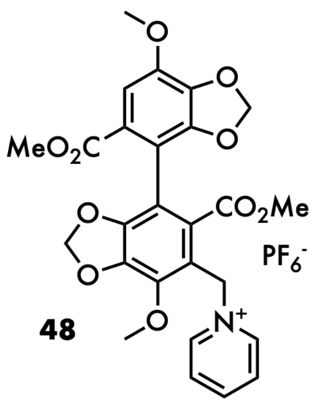

48

Following **General Procedure I**: **DiPyM 2PF$_6$** (92 mg, 0.2 mmol), dimethyl 7,7'-dimethoxy-[4,4'-bibenzo[d][1,3]dioxole]-5,5'-dicarboxylate (Bifendate) (100 mg, 0.24 mmol, 1.2 equiv), and [Ir(ppy)$_2$(dtbbpy)]PF$_6$ (1.8 mg, 2.0 µmol, 1 mol%) were dissolved in 1:1 (v/v) MeCN:1,2-DCE mixture (10 mL) and irradiated with 455 nm LEDs for 1 h. Purification by column chromatography (MeCN/DCM = 1:9) afforded **48** as a white microcrystalline solid (60 mg, 45%).

^{1}H NMR (400 MHz, CD$_3$CN) δ 8.85 (d, *J* = 5.1 Hz, 2H), 8.50 (tt, *J* = 7.8, 1.4 Hz, 1H), 8.02 (t, *J* = 7.0 Hz, 2H), 7.33 (s, 1H), 6.03 – 5.98 (m, 4H), 5.71 – 5.61 (m, 2H), 4.01 (s, 3H), 3.93 (s, 3H), 3.63 (s, 3H), 3.50 (s, 3H).

^{13}C NMR (101 MHz, CD$_3$CN) δ 168.4, 167.0, 150.6, 148.6, 147.0, 145.6, 144.0, 142.9, 139.2, 138.5, 129.8, 129.0, 124.9, 117.5, 112.03, 112.00, 111.0, 103.9, 103.8, 60.7, 58.9, 57.3, 53.4, 52.7

HRMS-ESI(+) Calcd. for $C_{26}H_{24}NO_{10}^+$ [M+]: 510.1395; Found: 510.1378.

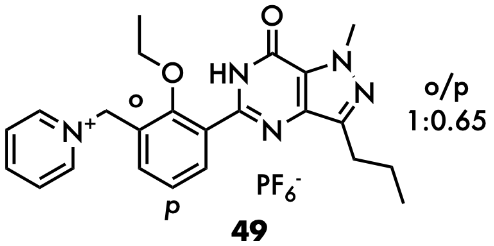

49

Following **General Procedure I**: **DiPyM 2PF$_6$** (92 mg, 0.2 mmol), 5-(2-ethoxyphenyl)-1-methyl-3-propyl-1,6-dihydro-7*H*-pyrazolo[4,3-*d*]pyrimidin-7-one (125 mg, 0.4 mmol, 2.0 equiv), and [Ir(ppy)$_2$(dtbbpy)]PF$_6$ (1.8 mg, 2.0 µmol, 1 mol%) were dissolved in 1:1 (v/v) MeCN:1,2-DCE mixture (10 mL) and irradiated with 455 nm LEDs for 1 h. Purification by column chromatography (MeCN/DCM = 1:9) afforded an inseparable mixture of regioisomers of **49** as a white microcrystalline solid (85 mg, 77%).

Scale-up procedure. The reaction was performed on a 10-fold scale using four 455 nm LED modules in a 250 mL Erlenmeyer flask (1:1 (v/v) MeCN:1,2-DCE mixture (100 mL)). After removing the solvents under reduced pressure, the crude mixture was suspended in DCM (50 mL), sonicated for 30s, and filtered through a pad of cotton. The filtrate was concentrated under reduced pressure, and purified by column chromatography (5:1 to 3:1 DCM:CH$_3$CN) to provide an inseparable mixture of isomers of **49** (423 mg, 40%)

^{1}H NMR (400 MHz, CD$_3$CN) (major regioisomer) δ 10.15 (s, 1H), 8.68 (d, *J* = 6.3 Hz, 2H), 8.49 (tt, *J* = 7.7, 1.6 Hz, 1H), 7.96 (t, *J* = 7.2 Hz, 2H), 7.60 (t, *J* = 8.1 Hz, 1H), 7.26 (d, *J* = 8.5 Hz, 1H), 7.15 (d, *J* = 4.9 Hz, 1H), 5.68 (s, 2H), 4.15 (s, 3H), 4.12 (q, *J* = 7.0 Hz, 2H), 2.53 (t, *J* = 7.6 Hz, 2H), 1.55 (dt, *J* = 15.1, 7.5 Hz, 2H), 1.29 (t, *J* = 6.9 Hz, 3H), 0.85 (t, *J* = 7.4 Hz, 3H).

^{13}C NMR (101 MHz, CD$_3$CN) (major regioisomer) δ 158.7, 154.8, 148.0, 147.4, 147.06, 145.5, 138.4, 133.5, 132.8, 129.1, 128.5, 125.8, 124.5, 115.3, 65.7, 62.8, 38.7, 28.2, 23.0, 14.9, 14.2.

HRMS-ESI(+) Calcd. for $C_{23}H_{26}N_5O_2^+$ [M]$^+$: 404.2081; Found: 404.20832.

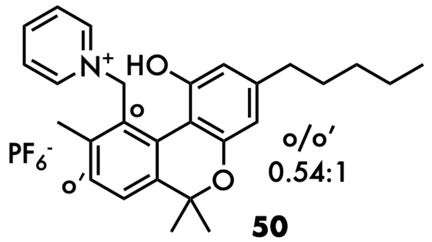

50

Following General Procedure I: **DiPyM 2PF$_6$** (924 mg, 2.0 mmol), 6,6,9-trimethyl-3-pentyl-6H-benzo[c]chromen-1-ol (CBN) (678 mg, 2.2 mmol, 1.2 equiv), and [Ir(ppy)$_2$(dtbbpy)]PF$_6$ (18 mg, 20.0 μmol, 1 mol%) were dissolved in 1:1 (v/v) MeCN:1,2-DCE mixture (100 mL) and irradiated with 455 nm LEDs for 1 h. Purification by column chromatography (MeCN/DCM = 1:9) afforded ***o'*-50** and ***o*-50** as white microcrystalline solids. Total yield: 940 mg, 86%.

***o'*-50**

^{1}H NMR (400 MHz, CD_3CN) δ 8.65 (d, *J* = 5.4 Hz, 2H), 8.51 (tt, *J* = 7.8, 1.4 Hz, 1H), 8.39 (s, 1H), 8.01 (t, *J* = 7.1 Hz, 2H), 7.59 (s, 1H), 7.29 (s, 1H), 6.42 (d, *J* = 1.6 Hz, 1H), 6.34 (d, *J* = 1.6 Hz, 1H), 5.73 (s, 2H), 2.50 (t, *J* = 7.4 Hz, 2H), 2.25 (s, 3H), 1.63 – 1.56 (m, 2H), 1.55 (s, 6H), 1.37 – 1.28 (m, 4H), 0.90 (t, *J* = 6.9 Hz, 3H).

^{13}C NMR (101 MHz, CD_3CN) δ 155.8, 155.6, 147.1, 146.7, 145.1, 138.9, 137.8, 130.9, 129.9, 129.4, 129.1, 126.7, 110.6, 110.6, 108.3, 77.7, 63.5, 36.1, 32.1, 31.3, 27.1, 23.1, 19.4, 14.3.

***o*-50**

^{1}H NMR (400 MHz, CD_3CN) δ 8.46 (d, *J* = 6.0 Hz, 2H), 8.41 (tt, *J* = 7.7, 1.4 Hz, 1H), 7.88 (t, *J* = 6.9 Hz, 2H), 7.40 (d, *J* = 7.9 Hz, 1H), 7.27 (d, *J* = 7.9 Hz, 1H), 6.41 (d, *J* = 1.5 Hz, 1H), 6.36 (d, *J* = 1.6 Hz, 1H), 5.88 (s, 2H), 2.49 (t, *J* = 7.5 Hz, 2H), 2.08 (s, 3H), 1.61 – 1.27 (m, 12H), 0.89 (t, *J* = 6.9 Hz, 3H).

^{13}C NMR (101 MHz, CD_3CN) δ 157.1, 154.0, 147.0, 146.6, 144.9, 143.7, 139.5, 131.0, 130.5, 128.7, 127.0, 125.6, 111.5, 110.8, 109.8, 79.7, 63.1, 36.1, 32.1, 31.4, 26.6, 23.2, 20.0, 14.4.

HRMS-ESI(+) Calcd. for $C_{27}H_{32}NO_2^+$ $[M]^+$: 402.2428; Found: 402.2424.

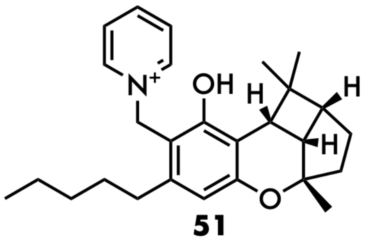

51

Following **General Procedure I**: **DiPyM 2PF$_6$** (92 mg, 0.2 mmol), 1,1,3a-trimethyl-6-pentyl-1a,1a1,2,3,3a,8b-hexahydro-1*H*-4-oxabenzo[*f*]cyclobuta[*cd*]inden-8-ol (CBL) (76 mg, 0.24 mmol, 1.2 equiv), and [Ir(ppy)$_2$(dtbbpy)]PF$_6$ (1.8 mg, 2.0 μmol, 1 mol%)

were dissolved in 1:1 (v/v) MeCN:1,2-DCE mixture (10 mL) and irradiated with 455 nm LEDs for 1 h. Purification by column chromatography (MeCN/DCM = 1:9) afforded **51** as a white microcrystalline solid (83 mg, 75%).

^{1}H NMR (400 MHz, CD_3CN) δ 8.62 (d, *J* = 6.0 Hz, 2H), 8.45 (t, *J* = 7.8 Hz, 1H), 7.95 (t, *J* = 7.0 Hz, 2H), 7.19 (s, 1H), 6.40 (s, 1H), 5.67 – 5.54 (m, 2H), 3.01 (d, *J* = 9.4 Hz, 1H), 2.74 – 2.59 (m, 2H), 2.48 (dd, *J* = 9.6, 7.4 Hz, 1H), 2.34 – 2.29 (m, 1H), 1.56 – 1.42 (m, 5H), 1.40 – 1.28 (m, 7H), 1.18 (s, 3H), 1.06 – 1.00 (m, 1H), 0.92 – 0.86 (m, 3H), 0.68 (s, 3H).

^{13}C NMR (101 MHz, CD_3CN) δ 158.2, 154.3, 146.6, 145.1, 143.5, 128.9, 111.8, 111.2, 109.0, 85.2, 58.2, 47.0, 39.8, 38.4, 38.2, 36.8, 33.9, 33.5, 32.3, 32.1, 27.3, 26.1, 23.2, 18.0, 14.3.

HRMS-ESI(+) Calcd. for $C_{27}H_{36}NO_2^+$ $[M]^+$: 406.2741; Found: 406.2743.

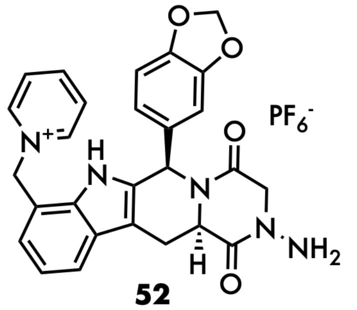

52

Following General Procedure I: **DiPyM 2PF$_6$** (92 mg, 0.2 mmol), 2-amino-6-(benzo[*d*][1,3]dioxol-5-yl)-2,3,6,7,12,12a-hexahydropyrazino[1',2':1,6]pyrido[3,4-b]indole-1,4-dione (AminoTadalafil) (94 mg, 0.24 mmol, 1.2 equiv), and $[Ir(ppy)_2(dtbbpy)]PF_6$ (1.8 mg, 2.0 μmol, 1 mol%) were combined, dissolved in 1:1 (v/v) MeCN:1,2-DCE mixture (20 mL) and irradiated with 455 nm LEDs for 1 h. Purification by preparative HPLC (MeCN/H_2O) afforded **52** (37 mg, 29%) as a white microcrystalline solid.

^{1}H NMR (400 MHz, CD_3CN) δ 10.37 (s, 1H), 8.71 (d, *J* = 6.1 Hz, 2H), 8.55 (t, *J* = 7.9 Hz, 1H), 8.04 (t, *J* = 7.1 Hz, 2H), 7.44 (d, *J* = 8.2 Hz, 1H), 7.12 (t, *J* = 7.8 Hz, 1H), 6.90 (dd, *J* = 8.0, 1.9 Hz, 1H), 6.85 (d, *J* = 1.9 Hz, 1H), 6.76 – 6.69 (m, 2H), 6.26 – 6.09 (m, 2H), 6.09 (s, 1H), 5.88 (d, *J* = 9.1 Hz, 2H), 4.21 (dd, *J* = 11.7, 4.1 Hz, 1H), 4.14 (dd, *J* = 17.4, 1.9 Hz, 1H), 4.01 (d, *J* = 17.4 Hz, 1H), 3.57 (dd, *J* = 15.6, 4.0 Hz, 1H), 3.06 (dd, *J* = 15.1, 11.7 Hz, 1H).

^{13}C NMR (101 MHz, CD_3CN) δ 167.0, 165.6, 148.6, 147.6, 147.4, 145.5, 138.3, 138.1, 137.1, 129.5, 125.0, 124.3, 122.9, 121.9, 120.9, 114.4, 108.8, 107.7, 105.6, 102.4, 63.6, 57.3, 56.7, 54.1, 26.7.

HRMS-ESI(+) Calcd. for $C_{27}H_{24}N_5O_4^+$ $[M]^+$: 482.1828; Found:482.1810.

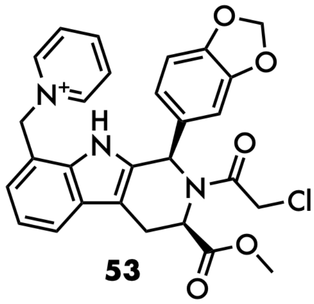

53

Following **General Procedure I**: **DiPyM 2PF$_6$** (92 mg, 0.2 mmol), methyl (1R,3R)-1-(benzo[d][1,3]dioxol-5-yl)-2-(2-chloroacetyl)-2,3,4,9-tetrahydro-1H-pyrido[3,4-b]indole-3-carboxylate (Chloropretadalafil) (103 mg, 0.24 mmol, 1.2 equiv), and [Ir(ppy)$_2$(dtbbpy)]PF$_6$ (1.8 mg, 2.0 µmol, 1 mol%) were dissolved in 1:1 (v/v) MeCN:1,2-DCE mixture (20 mL) and irradiated with 455 nm LEDs for 16 h. Purification by preparative HPLC (MeCN/H$_2$O) afforded **53** (39 mg, 30%) as a white microcrystalline solid.

^{1}H NMR (400 MHz, CD$_3$CN) δ 9.56 (s, 1H), 8.72 (d, *J* = 6.1 Hz, 2H), 8.53 (t, *J* = 7.4 Hz, 1H), 8.02 (t, *J* = 7.0 Hz, 2H), 7.49 (d, *J* = 8.2 Hz, 1H), 7.23 (t, J = 7.8 Hz, 1H), 7.01 (d, *J* = 7.3 Hz, 1H), 6.79 (s, 1H), 6.73 (d, *J* = 8.0 Hz, 1H), 6.69 (s, 1H), 6.57 (d, *J* = 8.1 Hz, 1H), 6.21 – 6.07 (m, 2H), 5.93 (d, *J* = 3.0 Hz, 2H), 4.86 (d, J = 6.5 Hz, 1H), 4.49 (d, *J* = 13.4 Hz, 1H), 4.27 (d, *J* = 13.6 Hz, 1H), 3.39 (d, *J* = 15.5 Hz, 1H), 3.08 (d, *J* = 26.6 Hz, 4H).

^{13}C NMR (101 MHz, CD$_3$CN) δ 171.4, 167.8, 148.4, 148.3, 147.2, 145.2, 138.3, 134.2, 133.3, 129.5, 125.6, 123.9, 123.5, 123.4, 123.3, 114.6, 110.5, 108.5, 106.8, 102.5, 63.5, 53.8, 52.8, 52.7, 43.8, 23.8.

HRMS-ESI(+) Calcd. for $C_{28}H_{25}ClN_3O_5^+$ [M]$^+$: 518.1483; Found: 518.1491.

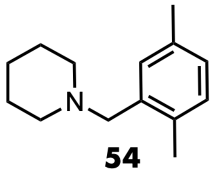

54

In a 50 mL round-bottom flask, **5** (100 mg, 0.29 mmol, 1.0 equiv), TEA (60 mg, 0.58 mmol, 2.0 equiv) and PtO$_2$ (15 mg, 0.066 mmol, 0.2 equiv) were combined in MeOH (15 mL). The flask was equipped with a three-way adapter connected to a hydrogen balloon. Using a vacuum pump, the flask was evacuated and back-filled with hydrogen 3 times, and then stirred under a hydrogen atmosphere at room temperature for 16 h. The mixture was filtered through a pad of celite, and the filter cake was washed with DCM (3x10 mL). The filtrate was concentrated. The crude material was purified by column chromatography (30:1 DCM:MeOH, silica pre-deactivated with 10% TEA during slurry packing) to provide **54** (56 mg, 95%).

^{1}H NMR (400 MHz, CDCl$_3$) δ 7.15 (d, *J* = 2.0 Hz, 1H), 7.07 (d, *J* = 7.6 Hz, 1H), 7.00 (dd, *J* = 7.5, 1.9 Hz, 1H), 3.42 (s, 2H), 2.43 (s, 4H), 2.36 (s, 6H), 1.61 (p, *J* = 5.6 Hz, 4H), 1.54 – 1.44 (m, 2H).

^{13}C NMR (101 MHz, $CDCl_3$) δ 136.9, 134.8, 134.3, 130.6, 130.1, 127.5, 61.6, 54.8, 26.2, 24.7, 21.1, 19.0.

HRMS-ESI(+) Calcd. for $C_{14}H_{22}N^+$ $[M+H]^+$: 204.1747; Found: 204.1753.

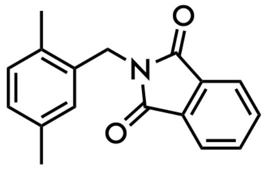

55

Following **General Procedure II**: **5** (100 mg, 0.29 mmol, 1.0 equiv), phthalimide (86 mg, 0.58 mmol, 2.0 equiv), KOtBu (49 mg, 0.44 mmol, 1.5 equiv), potassium iodide (97 mg, 0.58 mmol, 2.0 equiv), and 18-crown-6 (77 mg, 0.29 mmol, 1.0 equiv) were combined in dry toluene (5.0 mL) under argon and stirred at 130°C in an oil bath for 16 h. The crude material was purified by column chromatography (10:1 to 2:1 PE:DCM) to provide **55** (46 mg, 60%).

^1H NMR (400 MHz, $CDCl_3$) δ 7.86 (dd, J = 5.4, 3.1 Hz, 2H), 7.73 (dd, J = 5.5, 3.0 Hz, 2H), 7.05 (d, J = 7.9 Hz, 2H), 6.99 – 6.95 (m, 1H), 4.83 (s, 2H), 2.44 (s, 3H), 2.25 (s, 3H).

^{13}C NMR (101 MHz, $CDCl_3$) δ 168.4, 135.8, 134.2, 134.1, 133.0, 132.3, 130.5, 129.0, 128.6, 123.5, 39.3, 21.2, 19.1.

HRMS-ESI(+) Calcd. for $C_{17}H_{16}NO_2^+$ $[M+H]^+$: 266.1176; Found: 266.1176.

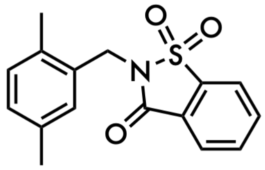

56

Following **General Procedure II**: **5** (100 mg, 0.29 mmol, 1.0 equiv), sodium saccharin (120 mg, 0.58 mmol, 2.0 equiv), potassium iodide (97 mg, 0.58 mmol, 2.0 equiv), and 18-crown-6 (77 mg, 0.29 mmol, 1.0 equiv) were combined in dry toluene (5.0 mL) under argon and stirred at 130°C in an oil bath for 16h. The crude material was purified by column chromatography (10:1 to 2:1 PE:DCM) to provide **56** (80 mg, 91%).

^1H NMR (400 MHz, $CDCl_3$) δ 8.11 – 8.06 (m, 1H), 7.95 – 7.82 (m, 3H), 7.23 – 7.20 (m, 1H), 7.08 (d, J = 7.7 Hz, 1H), 7.03 (dd, J = 8.1, 1.6 Hz, 1H), 4.92 (s, 2H), 2.42 (s, 3H), 2.29 (s, 3H).

^{13}C NMR (101 MHz, $CDCl_3$) δ 159.2, 138.0, 136.0, 135.0, 134.5, 133.3, 132.0, 130.6, 129.6, 129.2, 127.4, 125.5, 121.1, 40.7, 21.2, 19.0.

HRMS-ESI(+) Calcd. for $C_{16}H_{15}NO_3SNa^+$ $[M+Na]^+$: 324.0665; Found: 324.0662.

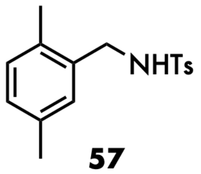

57

Following **General Procedure II**: **5** (100 mg, 0.29 mmol, 1.0 equiv), p-toluenesulfonamide (103 mg, 0.58 mmol, 2.0 equiv), KO^{t}Bu (49 mg, 0.44 mmol, 1.5 equiv), potassium iodide (97 mg, 0.58 mmol, 2.0 equiv) and 18-crown-6 (77 mg, 0.29 mmol, 1.0 equiv) were combined in dry toluene (5.0 mL) under argon and stirred at 130°C in an oil bath for 16 h. The crude material was purified by column chromatography (10:1 to 2:1 PE:DCM) to provide **57** (46 mg, 60%).

^{1}H NMR (400 MHz, $CDCl_3$) δ 7.80 – 7.73 (m, 2H), 7.31 (d, *J* = 8.0 Hz, 2H), 7.03 – 6.94 (m, 2H), 6.89 (d, *J* = 1.7 Hz, 1H), 4.55 – 4.41 (m, 1H), 4.05 (d, *J* = 5.9 Hz, 2H), 2.44 (s, 3H), 2.23 (s, 3H), 2.19 (s, 3H).

^{13}C NMR (101 MHz, $CDCl_3$) δ 143.6, 136.8, 135.8, 133.8, 133.5, 130.6, 129.8, 129.71, 129.0, 127.4, 45.5, 21.7, 20.9, 18.4.

HRMS-ESI(+) Calcd. for $C_{16}H_{19}NO_2SNa^+$ $[M+Na]^+$: 312.1029; Found: 312.1026.

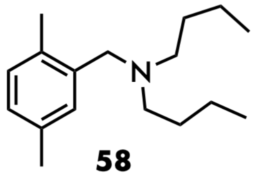

58

Following **General Procedure II**: **5** (100 mg, 0.29 mmol, 1.0 equiv), dibutylamine (74 mg, 0.58 mmol, 2.0 equiv), potassium iodide (97 mg, 0.58 mmol, 2.0 equiv), and 18-crown-6 (77 mg, 0.29 mmol, 1.0 equiv) were combined in dry toluene (5.0 mL) under argon and stirred at 130°C in an oil bath for 16 h. The crude material was purified by column chromatography (30:1 DCM:MeOH, silica pre-deactivated with 10% TEA during slurry packing) to provide **58** (63 mg, 88%).

^{1}H NMR (400 MHz, $CDCl_3$) δ 7.20 (d, *J* = 2.0 Hz, 1H), 7.06 (d, *J* = 7.6 Hz, 1H), 6.99 (dd, *J* = 7.7, 1.9 Hz, 1H), 3.51 (s, 2H), 2.47 – 2.40 (m, 4H), 2.36 (s, 6H), 1.53 – 1.44 (m, 4H), 1.38 – 1.28 (m, 4H), 0.92 (t, *J* = 7.3 Hz, 6H).

^{13}C NMR (101 MHz, $CDCl_3$) δ 138.1, 134.7, 133.9, 130.4, 129.9, 127.1, 57.1, 53.7, 29.2, 21.1, 20.7, 18.8, 14.1.

HRMS-ESI(+) Calcd. for $C_{17}H_{30}N^+$ $[M+H]^+$: 248.2373; Found: 248.2377.

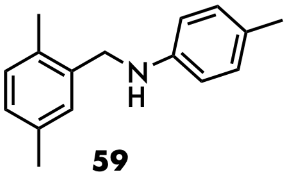

59

Following **General Procedure II**: **5** (100 mg, 0.29 mmol, 1.0 equiv), p-toluidine (62 mg, 0.58 mmol, 2.0 equiv), potassium iodide (97 mg, 0.58 mmol, 2.0 equiv), and 18-

crown-6 (77 mg, 0.29 mmol, 1 eq) were combined in dry toluene (5.0 mL) under argon and stirred at 130°C in an oil bath for 16 h. The crude material was purified by column chromatography (1:1 PE:DCM) to provide **59** (52 mg, 79%).

^{1}H NMR (400 MHz, $CDCl_3$) δ 7.24 (d, *J* = 1.9 Hz, 1H), 7.17 (d, *J* = 7.6 Hz, 1H), 7.11 – 7.06 (m, 3H), 6.67 – 6.62 (m, 2H), 4.27 (s, 2H), 3.84 – 3.66 (br s, 1H), 2.40 (s, 3H), 2.38 (s, 3H), 2.34 (s, 3H).

^{13}C NMR (101 MHz, $CDCl_3$) δ 146.2, 137.0, 135.6, 133.2, 130.3, 129.8, 129.1, 128.0, 126.6, 112.8, 46.8, 21.0, 20.4, 18.5.

HRMS-ESI(+) Calcd. for $C_{16}H_{20}N^+$ $[M+H]^+$: 226.1590; Found: 226.1586.

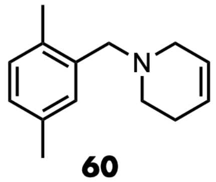

60

In a screw cap vial equipped with a stirring bar, **5** (100 mg, 0.29 mmol, 1.0 equiv) was suspended in MeOH (5.0 mL). $NaBH_4$ (22 mg, 0.58 mmol, 2.0 equiv) was added in portions over 5 min. The reaction was stirred for an additional 30 min, after which TLC analysis showed full conversion. The reaction mixture was diluted with DCM (10 mL) and water (20 mL). The layers were separated, and the aqueous layer was further extracted with DCM (2x10 mL). The combined organic layers were dried with Na_2SO_4, filtered and concentrated to dryness. The crude material was purified by column chromatography (60:1 DCM:MeOH, silica pre-deactivated with 10% TEA during slurry packing) to provide **60** (52 mg, 79%).

^{1}H NMR (400 MHz, $CDCl_3$) δ 7.17 (d, *J* = 1.9 Hz, 1H), 7.06 (d, *J* = 7.6 Hz, 1H), 7.00 (dd, *J* = 7.6, 1.9 Hz, 1H), 5.78 (m, 1H), 5.69 (m, 1H), 3.52 (s, 2H), 3.01 (m, 2H), 2.59 (t, *J* = 5.7 Hz, 2H), 2.34 (d, *J* = 4.6 Hz, 6H), 2.18 (m, 2H).

^{13}C NMR (101 MHz, $CDCl_3$) δ 136.2, 134.7, 133.9, 130.1, 129.9, 129.8, 127.3, 125.4, 125.0, 60.3, 52.9, 49.6, 26.1, 20.8, 18.7.

HRMS-ESI(+) Calcd. for $C_{14}H_{20}N^+$ $[M+H]^+$: 202.1590; Found: 202.1595.

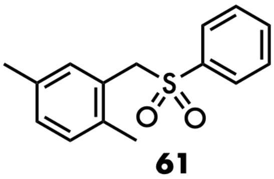

61

Following **General Procedure II**: **5** (100 mg, 0.29 mmol, 1.0 equiv), sodium benzenesulfinate (96 mg, 0.58 mmol, 2.0 equiv), potassium iodide (97 mg, 0.58 mmol, 2.0 equiv), and 18-crown-6 (77 mg, 0.29 mmol, 1.0 equiv) were combined in dry toluene (5.0 mL) under argon and stirred at 130°C in an oil bath for 16 h. The crude material was purified by column chromatography (10:1 to 2:1 PE:DCM) to provide **61** (61 mg, 81%).

^{1}H NMR (400 MHz, $CDCl_3$) δ 7.68-7.62 (m, 3H), 7.62 – 7.51 (m, 2H), 7.06 – 6.96 (m, 2H), 6.82 (d, *J* = 1.8 Hz, 1H), 4.33 (s, 2H), 2.21 (s, 3H), 2.04 (s, 3H).

Spectral data for **61** are consistent with literature values.[S1]

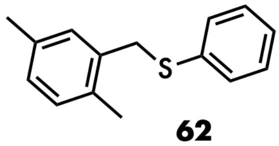

62

Following **General Procedure II**: **5** (100 mg, 0.29 mmol, 1.0 equiv), thiophenol (35 mg, 0.32 mmol, 1.1 equiv), and KO^{t}Bu (36 mg, 0.32 mmol, 1.1 equiv) were combined in dry toluene (5.0 mL) under argon and stirred at 130°C in an oil bath for 16 h. The crude material was purified by column chromatography (10:1 PE:DCM) to provide **62** (60 mg, 90%).

^{1}H NMR (400 MHz, $CDCl_3$) δ 7.24 (dd, *J* = 7.1, 1.5 Hz, 2H), 7.20 – 7.15 (m, 2H), 7.13 – 7.07 (m, 1H), 6.95 (d, *J* = 7.5 Hz, 1H), 6.88 (d, *J* = 8.0 Hz, 2H), 3.98 (s, 2H), 2.25 (s, 3H), 2.16 (s, 3H).

^{13}C NMR (101 MHz, $CDCl_3$) δ 137.0, 135.6, 134.8, 133.6, 130.7, 130.5, 130.1, 128.9, 128.3, 126.4, 37.4, 21.0, 18.8.

HRMS-ESI(+) Calcd. for $C_{15}H_{16}SNa^+$ $[M+Na]^+$: 251.0865; Found: 251.0865.

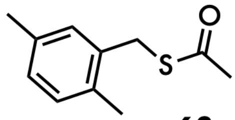

63

Following **General Procedure II**: **5** (100 mg, 0.29 mmol, 1.0 equiv), potassium thioacetate (66 mg, 0.58 mmol, 2.0 equiv), potassium iodide (97 mg, 0.58 mmol, 2.0 equiv), and 18-crown-6 (77 mg, 0.29 mmol, 1.0 equiv) were combined in dioxane (5.0 mL) under argon and stirred at 110°C in an oil bath for 16 h. The crude material was purified by column chromatography (3:1 PE:DCM) to provide **63** (46 mg, 82%).

^{1}H NMR (400 MHz, $CDCl_3$) δ 7.10 (s, 1H), 7.05 (d, *J* = 7.7 Hz, 1H), 6.98 (d, *J* = 7.8, 1H), 4.12 (s, 2H), 2.35 (s, 3H), 2.30 (s, 3H), 2.29 (s, 3H).

^{13}C NMR (101 MHz, $CDCl_3$) δ 195.6, 135.9, 134.9, 133.5, 130.7, 130.5, 128.5, 31.8, 30.5, 21.0, 19.0.

HRMS-ESI(+) Calcd. for $C_{11}H_{14}OSNa^+$ $[M+Na]^+$: 217.0658; Found: 217.0658.

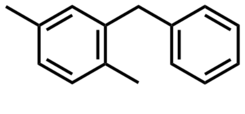

64

A screw cap vial was loaded with **5** (100 mg, 0.29 mmol, 1.0 equiv), phenylboronic acid (71 mg, 0.58 mmol, 2.0 equiv), PPh_3 (11.5 mg, 0.043 mmol, 0.15 equiv) and

DIPEA (112 mg, 0.87 mmol, 3.0 equiv). After flushing with argon, EtOH (5 mL) was added, and the mixture was degassed by purging the mixture with argon through a needle while vigorously stirring for 20 minutes. Afterwards, $Pd(OAc)_2$ (3.3 mg, 0.015 mmol, 0.05 equiv) was added, the vial was sealed and stirred in a 100°C oil bath for 16 h. The reaction mixture was diluted with DCM (10 mL) and water (20 mL). The layers were separated, and the aqueous layer was further extracted with DCM (2x10 mL). The combined organic layers were dried with Na_2SO_4, filtered, and concentrated to dryness. The crude material was purified by column chromatography (10:1 PE:DCM) to provide **64** (55 mg, 96%).

^{1}H NMR (400 MHz, $CDCl_3$) δ 7.22 – 7.16 (m, 2H), 7.14 – 7.08 (m, 1H), 7.08 – 7.03 (m, 2H), 6.99 (d, *J* = 7.6 Hz, 1H), 6.92 – 6.85 (m, 2H), 3.88 (s, 2H), 2.22 (s, 3H), 2.13 (s, 3H).

Spectral data for **64** are consistent with literature values.[S2]

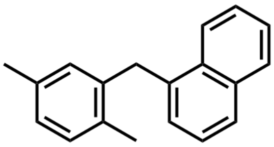

65

A screw cap vial was loaded with **5** (100 mg, 0.29 mmol, 1.0 equiv), trimethoxy(naphthalen-1-yl)silane (144 mg, 0.58 mmol, 2.0 equiv), PPh_3 (11.5 mg, 0.043 mmol, 0.15 equiv) and TBAF·$3H_2O$ (184 mg, 0.58 mmol, 2.0 equiv). After flushing with argon, EtOH (5.0 mL) was added, and the mixture was degassed by purging the mixture with argon through a needle while vigorously stirring for 20 minutes. Afterwards, $Pd(OAc)_2$ (3.3 mg, 0.015 mmol, 0.05 equiv) was added, the vial was sealed, and it was stirred in a 100°C oil bath for 16 h. The reaction mixture was diluted with DCM (10 mL) and water (20 mL). The layers were separated, and the aqueous layer was further extracted with DCM (2x10 mL). The combined organic layers were dried with Na_2SO_4, filtered, and concentrated to dryness. The crude material was purified by column chromatography (10:1 PE:DCM) to provide **65** (61 mg, 86%).

^{1}H NMR (400 MHz, $CDCl_3$) δ 7.96 – 7.88 (m, 1H), 7.83 – 7.74 (m, 1H), 7.65 (d, *J* = 8.2 Hz, 1H), 7.45 – 7.34 (m, 2H), 7.27 (dd, *J* = 8.2, 7.1 Hz, 1H), 7.12 – 7.01 (m, 1H), 6.92 (ddd, *J* = 9.3, 7.4, 1.5 Hz, 2H), 6.70 (d, *J* = 1.9 Hz, 1H), 4.27 (s, 2H), 2.17 (s, 3H), 2.12 (s, 3H).

Spectral data for **65** are consistent with literature values.[S3]

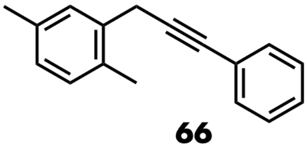

66

A screw cap vial was loaded with **5** (100 mg, 0.29 mmol, 1.0 equiv), phenylacetylene (60 mg, 0.58 mmol, 2.0 equiv), PPh_3 (11.5 mg, 0.043 mmol, 0.15 equiv) and K_2CO_3 (120 mg, 0.87 mmol, 3.0 equiv). After flushing with argon, CH_3CN (5.0 mL) was added, and the mixture was degassed by purging the mixture with argon through a needle

while vigorously stirring for 20 minutes. Afterwards, $Pd(OAc)_2$ (3.3 mg, 0.015 mmol, 0.05 equiv) was added, the vial was sealed, and it was stirred in a 100°C oil bath for 16 h. The reaction mixture was diluted with DCM (10 mL) and water (20 mL). The layers were separated, and the aqueous layer was further extracted with DCM (2x10 mL). The combined organic layers were dried with Na_2SO_4, filtered, and concentrated to dryness. The crude material was purified by column chromatography (10:1 PE:DCM) to provide **66** (35 mg, 54%).

^{1}H NMR (400 MHz, $CDCl_3$) δ 7.44-7.38 (m, 2H), 7.34 – 7.24 (m, 4H), 7.08 – 6.94 (m, 2H), 3.69 (s, 2H), 2.31 (s, 6H).

Spectral data for **66** are consistent with literature values.[S4]

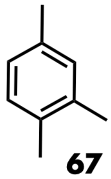

67

In a 50 mL round-bottom flask, **5** (100 mg, 0.29 mmol, 1.0 equiv) and 10% Pd/C (60% wet, 75 mg, 0.029 mmol, 0.1 equiv) were dissolved in MeOH (15 mL). The flask was equipped with a three-way adapter connected to a hydrogen balloon. Using a vacuum pump, the flask was evacuated and back-filled with hydrogen 3 times and then stirred under a hydrogen atmosphere at room temperature for 16 h. The mixture was diluted with water (20 mL), filtered through a pad of Celite, and washed with an additional portion of water (10 mL). The product was collected by washing with DCM (3x10 mL). Concentration under reduced pressure provided **67** (20 mg, 57%).

Note: *The product yield is likely higher; however, some material may have been lost during drying under vacuum.*

^{1}H NMR (400 MHz, $CDCl_3$) δ 7.04 (d, J = 7.6 Hz, 1H), 6.98 (s, 1H), 6.93 (d, J = 7.6 Hz, 1H), 2.31 (s, 3H), 2.25 (m, 6H).

Spectral data for **67** are consistent with literature values.[S5]

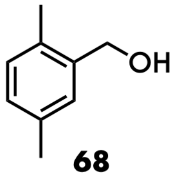

68

Following **General Procedure II**: **5** (100 mg, 0.29 mmol, 1.0 equiv), sodium acetate (48 mg, 0.58 mmol, 2.0 equiv), potassium iodide (97 mg, 0.58 mmol, 2.0 equiv), and 18-crown-6 (77 mg, 0.29 mmol, 1.0 equiv) were combined in dioxane (5 mL) under argon and stirred at 110°C in an oil bath for 16 h. The reaction mixture was diluted with DCM (10 mL) and water (20 mL). The layers were separated, and the aqueous layer was further extracted with DCM (2x10 mL). The combined organic layers were dried with Na_2SO_4, filtered, and concentrated to dryness.

The crude material was dissolved in 5.0 mL of EtOH, and KOH (81 mg, 1.45 mmol, 5.0 equiv) was added. The mixture was stirred at room temperature for 16 h. The

reaction mixture was diluted with DCM (10 mL) and 1.0 M HCl (20 mL). The layers were separated, and the aqueous layer was further extracted with DCM (2x10 mL). The combined organic layers were dried with Na_2SO_4, filtered, and concentrated to dryness. The crude material was purified by column chromatography (3:1 PE:EA) to provide **68** (30 mg, 76%).

^{1}H NMR (400 MHz, $CDCl_3$) δ 7.18 (s, 1H), 7.08 (d, J = 7.7 Hz, 1H), 7.03 (d, J = 7.7, 1H), 4.65 (s, 2H), 2.34 (s, 3H), 2.32 (s, 3H), 1.92 (br s, 1H).

Spectral data for **68** are consistent with literature values.[S6]

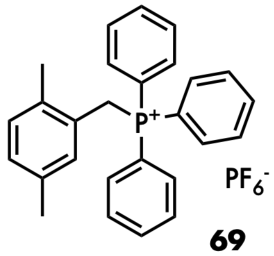

69

Following **General Procedure II**: **5** (100 mg, 0.29 mmol, 1.0 equiv), PPh_3 (76 mg, 0.29 mmol, 1.0 equiv), and potassium iodide (97 mg, 0.58 mmol, 2.0 equiv) were combined in toluene (5.0 mL), sealed, and heated in a microwave reactor at 140°C for 30 minutes. The resulting mixture was diluted with DCM (10 mL), filtered, concentrated, and purified by column chromatography (10:1 DCM:CH_3CN) to provide **69** (133 mg, 87%).

^{1}H NMR (400 MHz, $CDCl_3$) δ 7.84 – 7.78 (m, 3H), 7.64 (td, J = 7.9, 3.5 Hz, 6H), 7.43 (ddt, J = 12.6, 7.1, 1.2 Hz, 6H), 6.99 (dt, J = 7.9, 2.3 Hz, 1H), 6.91 (d, J = 7.8 Hz, 1H), 6.57 (t, J = 2.4 Hz, 1H), 4.44 (d, J = 13.8 Hz, 2H), 2.03 (s, 3H), 1.61 (s, 3H).

^{13}C NMR (101 MHz, $CDCl_3$) δ 136.60, 136.56, 135.60, 135.57, 135.5, 135.4, 134.1, 134.0, 131.7, 131.6, 131.28, 131.25, 130.6, 130.4, 130.12, 130.08, 124.8, 124.7, 117.6, 116.7, 28.3, 27.8, 20.8, 18.87, 18.85.

^{31}P NMR (162 MHz, $CDCl_3$) δ 21.2, -131.1, -135.5, -139.9, -144.3, -148.7, -153.1, -157.5.

^{19}F NMR (376 MHz, $CDCl_3$) δ -71.9, -73.8.

HRMS-ESI(+) Calcd. for $C_{27}H_{26}P^+$ $[M]^+$: 381.1767; Found: 381.1778.

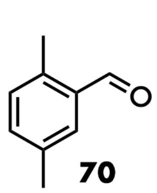

70

A screw cap vial was loaded with **5** (100 mg, 0.29 mmol, 1.0 equiv), N,N-dimethyl-4-nitrosoaniline hydrochloride (109 mg, 0.58 mmol, 2.0 equiv) and KOH (49 mg, 3.0 equiv). After flushing with argon, EtOH (5.0 mL) was added, the vial was sealed and stirred vigorously at room temperature for 16 h. Concentrated HCl (1.0 mL) was added dropwise, and the mixture was vigorously stirred for an additional hour. The reaction mixture was diluted with DCM (10 mL) and water (20 mL). The layers were separated,

and the aqueous layer was further extracted with DCM (2x10 mL). The combined organic layers were dried with Na_2SO_4, filtered, and concentrated to dryness. The crude material was purified by column chromatography (10:1 PE:DCM) to provide **70** (35 mg, 91%).

^{1}H NMR (400 MHz, $CDCl_3$) δ 10.20 (s, 1H), 7.56 (d, *J* = 2.0 Hz, 1H), 7.25 (dd, *J* = 7.8, 2.0 Hz, 1H), 7.11 (d, *J* = 7.7 Hz, 1H), 2.58 (s, 3H), 2.34 (s, 3H).

Spectral data for **70** are consistent with literature values.[S7]

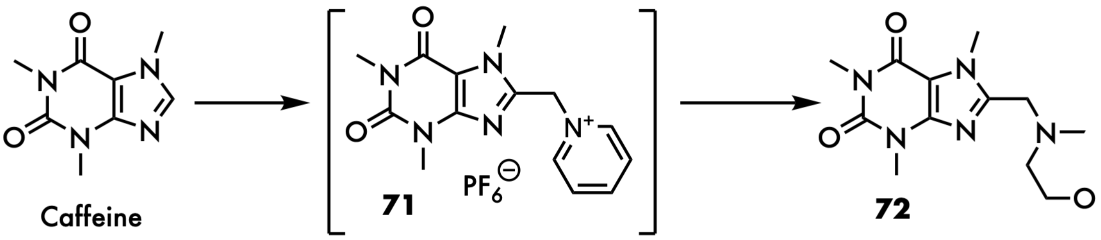

Preparation of stock solution. Following **General Procedure I**: **DiPyM 2PF$_6$** (920 mg, 2.0 mmol), caffeine (388 mg, 2.0 mmol, 1.0 equiv), and [Ir(ppy)$_2$(dtbbpy)]PF$_6$ (18 mg, 20 μmol, 1 mol%) were combined and irradiated with 455 nm LEDs in a 250 mL Erlenmeyer flask (50 mL MeCN) for 16 h.

HRMS-ESI(+) Calcd. for $C_{14}H_{16}N_5O_2^+$ $[M]^+$: 286.1299; Found 286.1296.

The ^{1}H NMR spectrum of the crude mixture indicates ~75% yield of the intermediate **71** (**Figure S5**).

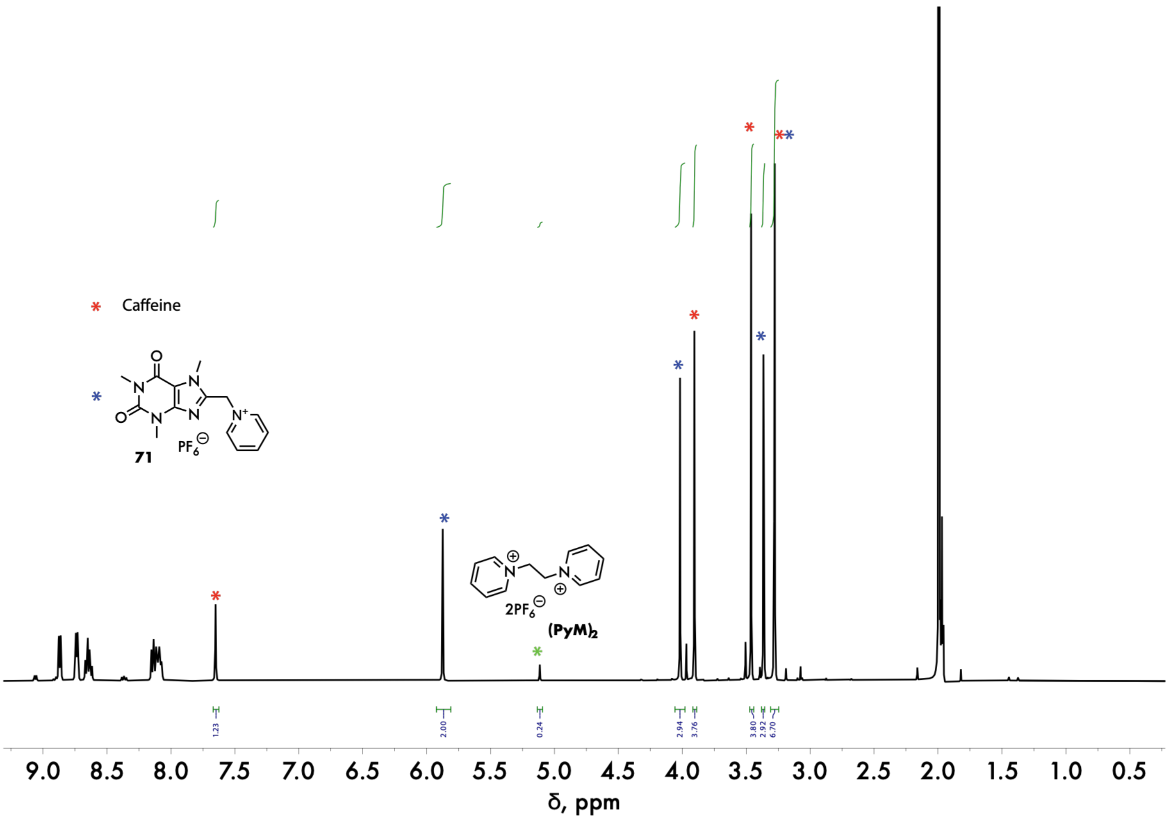

Figure S5. ^{1}H NMR (400 MHz, CD_3CN) spectrum of the reaction mixture of caffeine and **DiPyM $2PF_6$**.

6.7 mL of stock solution was transferred to a microwave vial, and the solvents were removed with a rotary evaporator. The vial was loaded with morpholine (35 mg, 0.4 mmol, 2.0 equiv), potassium iodide (67 mg, 0.4 mmol, 2.0 equiv), and 18-crown-6 (53 mg, 0.2 mmol, 1.0 equiv). After flushing with argon, dioxane (5.0 mL) was added, the vial was sealed, and it was heated in a microwave reactor at 130°C for 1h. The reaction mixture was diluted with DCM (10 mL) and water (20 mL). The layers were separated, and the aqueous layer was further extracted with DCM (2x10 mL). The combined organic layers were dried with Na_2SO_4, filtered, and concentrated to dryness. The crude material was purified by column chromatography (40:1 to 20:1 EA:MeOH) to provide **72** (40 mg, 68%).

^{1}H NMR (400 MHz, $CDCl_3$) δ 4.02 (s, 3H), 3.68 (t, J = 4.6 Hz, 4H), 3.64 (s, 2H), 3.54 (s, 3H), 3.37 (s, 3H), 2.49 (d, J = 4.6 Hz, 4H).

Spectral data for **72** are consistent with literature values.[S8]

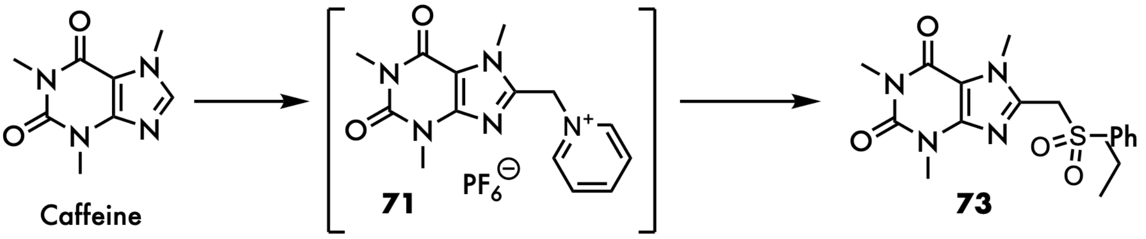

6.7 mL of stock solution was transferred to a screw-cap vial, and the solvents were removed with a rotary evaporator. The vial was loaded with sodium benzenesulfinate (66 mg, 0.4 mmol, 2.0 equiv), potassium iodide (67 mg, 0.4 mmol, 2.0 equiv) and 18-crown-6 (53 mg, 0.2 mmol, 1.0 equiv). After flushing with argon, dioxane (5.0 mL) was added, the vial was sealed, and it was stirred vigorously in a 110°C oil bath for 16 h. The reaction mixture was diluted with DCM (10 mL) and water (20 mL). The layers were separated, and the aqueous layer was further extracted with DCM (2x10 mL). The combined organic layers were dried with Na_2SO_4, filtered, and concentrated to dryness. The crude material was purified by column chromatography (DCM as eluent) to provide **73** (46 mg, 64%).

^{1}H NMR (400 MHz, $CDCl_3$) δ 7.79 – 7.74 (m, 2H), 7.70 (ddt, *J* = 8.7, 7.0, 1.3 Hz, 1H), 7.58 – 7.52 (m, 2H), 4.57 (s, 2H), 4.06 (s, 3H), 3.39 (s, 3H), 3.35 (s, 3H).

Spectral data for **73** are consistent with literature values.[S9]

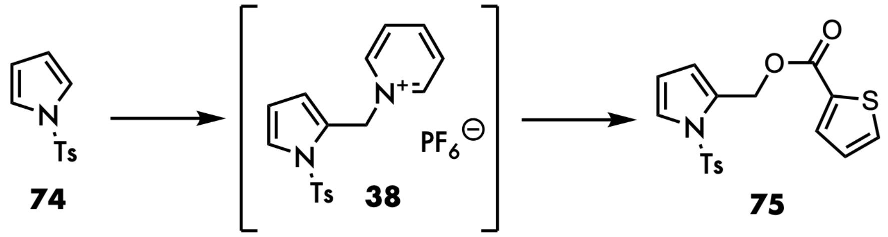

Following **General Procedure I**: **DiPyM 2PF$_6$** (92 mg, 0.20 mmol), 1-tosyl-1*H*-pyrrole (49 mg, 0.4 mmol, 2.0 equiv), and [Ir(dF(CF_3)ppy)$_2$(dtbbpy)]PF_6 (1.8 mg, 2.0 µmol, 1 mol%) were combined and irradiated with 455 nm LEDs for 16 h.

The above reaction mixture was transferred to a screw cap vial and the solvents were removed with a rotary evaporator. The vial was loaded with thiophene-2-carboxylic acid (51 mg, 0.4 mmol, 2 equiv), DIPEA (78 mg, 0.6 mmol, 3 equiv), potassium iodide (67 mg, 0.4 mmol, 2 equiv) and 18-crown-6 (53 mg, 0.2 mmol, 1 equiv). After flushing with argon, dioxane (5.0 mL) was added, the vial was sealed and stirred vigorously in a 110°C oil bath for 16 h. The reaction mixture was diluted with DCM (10 mL) and water (20 mL). The layers were separated, and the aqueous layer was further extracted with DCM (2x10 mL). The combined organic layers were dried with Na_2SO_4, filtered, and concentrated to dryness. The crude material was purified by column chromatography (1:1 PE:DCM) to provide **75** (46 mg, 64%).

^{1}H NMR (400 MHz, $CDCl_3$) δ 7.73 – 7.68 (m, 2H), 7.58 – 7.51 (m, 2H), 7.37 (dd, *J* = 3.3, 1.8 Hz, 1H), 7.19 (d, *J* = 8.2 Hz, 2H), 7.04 (dd, *J* = 5.0, 3.8 Hz, 1H), 6.45 (dd, *J* = 3.5, 1.8 Hz, 1H), 6.28 (t, *J* = 3.4 Hz, 1H), 5.45 (s, 2H), 2.31 (s, 3H).

^{13}C NMR (101 MHz, $CDCl_3$) δ 161.8, 145.1, 136.3, 133.7, 133.5, 132.6, 130.1, 128.65, 127.7, 127.0, 124.5, 118.1, 111.6, 58.2, 21.7.

HRMS-ESI(+) Calcd. for $C_{17}H_{16}NO_4S_2^+$ $[M]^+$: 362.0515; Found: 362.0525.

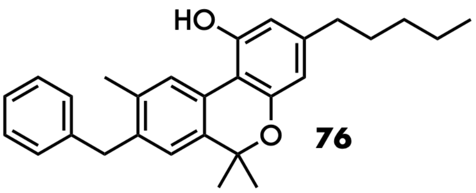

76

A screw cap vial was loaded with **o'-50** (64 mg, 0.12 mmol, 1.0 equiv), phenylboronic acid (29 mg, 0.24 mmol, 2.0 equiv), PPh3 (4.6 mg, 0.018 mmol, 0.15 equiv) and DIPEA (45 mg, 0.36 mmol, 3.0 equiv). After flushing with argon, EtOH (5 mL) was added, and the mixture was degassed by purging the mixture with argon through a needle while vigorously stirring for 20 minutes. Afterwards, $Pd(OAc)_2$ (1.3 mg, 5.8 µmol, 0.05 equiv) was added, the vial was sealed and stirred in a 100°C oil bath for 16 h. The reaction mixture was diluted with DCM (10 mL) and water (20 mL). The layers were separated, and the aqueous layer was further extracted with DCM (2x10 mL). The combined organic layers were dried with Na_2SO_4, filtered, and concentrated to dryness. The crude material was purified by column chromatography (10:1 PE:EA) to provide **76** (33 mg, 71%).

^1H NMR (400 MHz, $CDCl_3$) δ 8.18 (s, 1H), 7.34 – 7.24 (m, 2H), 7.22 – 7.17 (m, 1H), 7.14 – 7.11 (m, 2H), 7.00 (s, 1H), 6.43 (d, *J* = 1.6 Hz, 1H), 6.30 (d, *J* = 1.6 Hz, 1H), 4.02 (s, 2H), 2.50 (t, *J* = 7.6 Hz, 2H), 2.25 (s, 3H), 1.64 – 1.53 (m, 8H), 1.38 – 1.27 (m, 2H), 0.89 (t, *J* = 6.9 Hz, 3H).

^{13}C NMR (101 MHz, $CDCl_3$) δ 154.6, 153.2, 144.4, 140.5, 137.6, 137.5, 135.8, 128.73, 128.5, 127.8, 126.1, 126.0, 124.6, 110.8, 110.0, 108.7, 77.4, 39.7, 35.8, 31.6, 30.6, 27.3, 22.7, 19.9, 14.2.

HRMS-ESI(+) Calcd. for $C_{28}H_{33}O_2^+$ $[M+H]^+$: 401.2475; Found 401.2464.

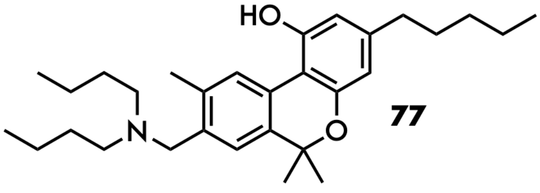

77

Following General Procedure II: **o'-50** (38 mg, 0.07 mmol, 1.0 equiv), dibutylamine (18 mg, 0.14 mmol, 2.0 equiv), potassium iodide (23 mg, 0.14 mmol, 2.0 equiv), and 18-crown-6 (18 mg, 0.07 mmol, 1.0 equiv) were combined in dioxane (5.0 mL) under argon and stirred at 110°C in an oil bath for 16 h. The crude material was purified by column chromatography (5:1 PE:EA) to provide **77** (25 mg, 80%).

^1H NMR (400 MHz, $CDCl_3$) δ 8.11 (s, 1H), 7.29 (s, 1H), 6.41 (d, *J* = 1.6 Hz, 1H), 6.26 (d, *J* = 1.6 Hz, 1H), 3.53 (s, 2H), 2.51 – 2.39 (m, 6H), 2.35 (s, 3H), 1.61 – 1.53 (m, 8H), 1.50 – 1.42 (m, 4H), 1.34 – 1.25 (m, 8H), 0.88 (t, *J* = 7.2 Hz, 9H).

^{13}C NMR (101 MHz, $CDCl_3$) δ 154.6, 153.2, 144.3, 137.1, 137.0, 135.9, 127.4, 125.9, 123.9, 110.7, 110.0, 108.8, 77.4, 56.8, 53.9, 35.7, 31.6, 30.6, 29.2, 27.3, 22.7, 20.9, 19.5, 14.6, 14.2.

HRMS-ESI(+) Calcd. for $C_{30}H_{46}NO_2^+$ $[M+H]^+$: 452.3523; Found 452.3506.

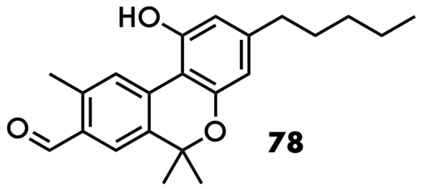

78

A screw cap vial was loaded with **o'-50** (29 mg, 0.05 mmol, 1.0 equiv), N,N-dimethyl-4-nitrosoaniline hydrochloride (20 mg, 0.10 mmol, 2.0 equiv) and KOH (9 mg, 0.15 mmol, 3.0 equiv). After flushing with argon, EtOH (5.0 mL) was added, the vial was sealed and stirred vigorously at room temperature for 16 h. Concentrated HCl (1.0 mL) was added dropwise, and the mixture was vigorously stirred for an additional hour. The reaction mixture was diluted with DCM (10 mL) and water (20 mL). The layers were separated, and the aqueous layer was further extracted with DCM (2x10 mL). The combined organic layers were dried with Na_2SO_4, filtered, and concentrated to dryness. The crude material was purified by column chromatography (10:1 PE:EA) to provide **78** (12 mg, 67%).

^1H NMR (400 MHz, $CDCl_3$) δ 10.28 (s, 1H), 8.33 (s, 1H), 7.68 (s, 1H), 6.45 (d, *J* = 1.6 Hz, 1H), 6.31 (d, *J* = 1.6 Hz, 1H), 5.56 (s, 1H), 2.70 (s, 3H), 2.51 (t, *J* = 7.5 Hz, 2H), 1.65 – 1.56 (m, 8H), 1.35 – 1.28 (m, 4H), 0.89 (t, *J* = 6.9 Hz, 3H).

^{13}C NMR (101 MHz, $CDCl_3$) δ 192.13, 155.55, 154.03, 146.75, 140.13, 137.58, 133.48, 132.16, 129.30, 125.33, 111.11, 110.08, 107.88, 77.33, 35.84, 31.58, 30.50, 27.18, 22.67, 19.50, 14.16.

HRMS-ESI(+) Calcd. for $C_{22}H_{27}O_3^+$ $[M+H]^+$: 399.1955; Found 399.1945.

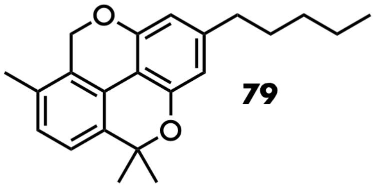

79

In a screw cap vial, ***o*-50** (65 mg, 0.12 mmol, 1.0 equiv) was dissolved in CH_3CN (5 mL), and K_2CO_3 (49 mg, 0.36 mmol, 3.0 equiv) was added. The mixture was stirred vigorously for 40 hours. After removing the solvent under reduced pressure, the crude mixture was suspended in petroleum ether (15 mL) and loaded onto a short plug of silica. After washing with an additional portion of PE (15 mL), the product was collected by washing with 1:1 DCM:PE (20 mL). Concentration under reduced pressure provided **79** (31 mg, 81%).

^1H NMR (400 MHz, $CDCl_3$) δ 7.04 – 6.96 (m, 2H), 6.37 (d, *J* = 1.4 Hz, 1H), 6.36 (d, J = 1.4 Hz, 1H), 5.24 (s, 2H), 2.52 (t, *J* = 7.6 Hz, 2H), 2.22 (s, 3H), 1.66 – 1.57 (m, 8H), 1.40 – 1.27 (m, 4H), 0.90 (t, *J* = 6.8 Hz, 3H).

^{13}C NMR (101 MHz, $CDCl_3$) δ 152.3, 151.4, 145.9, 133.2, 131.8, 129.3, 125.8, 122.0, 121.2, 110.3, 108.9, 105.7, 78.7, 66.1, 36.5, 31.6, 30.9, 28.2, 22.7, 17.8, 14.2.

HRMS-ESI(+) Calcd. for $C_{22}H_{27}O_2^+$ $[M+H]^+$: 323.2006; Found 323.1998.

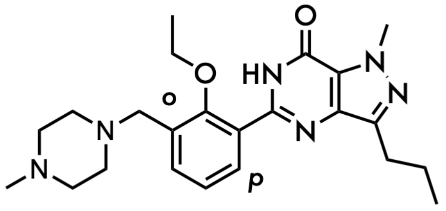

80

Following General Procedure II: **49** (144 mg, 0.26 mmol, 1.0 equiv), 1-methylpiperazine (278 mg, 2.6 mmol, 10 equiv), and potassium iodide (87 mg, 0.52 mmol, 2.0 equiv) were combined in dioxane (5.0 mL) under argon and stirred at 110°C in an oil bath for 16 h. The crude material was purified by column chromatography (20:1:0.1 DCM:MeOH:NH_3(aq)) to provide **80** (62 mg, 56%).

***o*-80 (**30%**)**

^1H NMR (400 MHz, $CDCl_3$) δ 12.28 (s, 1H), 7.29 (t, *J* = 8.0 Hz, 1H), 6.95 (d, J = 8.4 Hz, 1H), 6.86 (d, *J* = 7.5 Hz, 1H), 4.25 (s, 3H), 4.02 (q, *J* = 6.9 Hz, 2H), 3.39 (s, 2H), 2.86 (t, *J* = 7.7 Hz, 2H), 2.66 – 2.26 (m, 8H), 2.24 (s, 3H), 1.86 – 1.75 (m, 2H), 1.28 (t, *J* = 6.9 Hz, 3H), 0.97 (t, *J* = 7.4 Hz, 3H).

^{13}C NMR (101 MHz, $CDCl_3$) δ 158.1, 155.1, 148.0, 146.3, 138.8, 137.4, 130.6, 124.9, 124.2, 123.6, 113.3, 64.9, 61.2, 54.9, 52.3, 45.9, 38.3, 27.8, 22.7, 14.8, 14.1.

***p*-80** (26%)

^1H NMR (400 MHz, $CDCl_3$) δ 11.11 (s, 1H), 8.37 (d, J = 8.1 Hz, 1H), 7.06 (dd, *J* = 8.1, 1.4 Hz, 1H), 7.04 (d, *J* = 1.4 Hz, 1H), 4.31 – 4.24 (m, 5H), 3.52 (s, 2H), 2.90 (t, *J* = 7.8 Hz, 3H), 2.55 – 2.33 (m, 8H), 2.30 (s, 3H), 1.90 – 1.79 (m, 3H), 1.58 (t, *J* = 7.0 Hz, 3H), 1.01 (t, *J* = 7.4 Hz, 3H).

^{13}C NMR (101 MHz, $CDCl_3$) δ 156.6, 154.1, 148.5, 146.6, 143.8, 138.9, 130.9, 124.5, 122.5, 119.0, 113.3, 65.4, 62.7, 55.2, 53.2, 46.1, 38.3, 27.9, 22.5, 14.8, 14.2.

HRMS-ESI(+) Calcd. for $C_{23}H_{33}N_6O_2^+$ $[M+H]^+$: 425.2660; Found 425.2652.

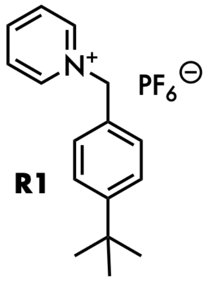

R1

^1H NMR (400 MHz, CD_3CN) δ 8.73 (d, *J* = 6.0 Hz, 2H), 8.51 (t, *J* = 7.8 Hz, 1H), 8.02 (t, *J* = 6.8 Hz, 2H), 7.58 – 7.45 (m, 2H), 7.37 (d, *J* = 8.3 Hz, 2H), 5.67 (s, 2H), 1.30 (s, 9H).

^{13}C NMR (101 MHz, CD_3CN) δ 154.17, 147.20, 145.37, 131.03, 129.87, 129.62, 127.45, 65.21, 35.41, 31.37.

HRMS-ESI(+) Calcd. for $C_{16}H_{20}N^+$ $[M]^+$: 226.1590; Found: 226.1595.

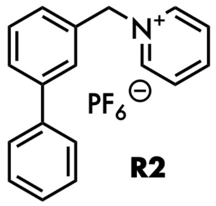

R2

^1H NMR (400 MHz, CD_3CN) δ 8.80 (d, *J* = 5.6 Hz, 2H), 8.52 (t, *J* = 7.8 Hz, 1H), 8.03 (t, *J* = 7.0 Hz, 2H), 7.77 – 7.72 (m, 2H), 7.68 – 7.63 (m, 2H), 7.59 – 7.52 (m, 1H), 7.49 (td, *J* = 6.8, 1.6 Hz, 2H), 7.46 – 7.38 (m, 2H), 5.77 (s, 2H).

^{13}C NMR (101 MHz, CD_3CN) δ 147.26, 145.51, 143.10, 140.73, 134.51, 131.10, 130.05, 129.65, 129.37, 129.10, 129.02, 128.96, 128.00, 65.44.

HRMS-ESI(+) Calcd. for $C_{18}H_{16}N^+$ $[M]^+$: 246.1277; Found: 246.1267.

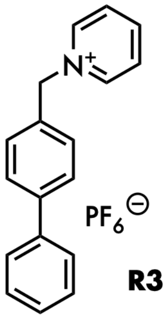

R3

^1H NMR (400 MHz, CD_3CN) δ 8.81 (d, *J* = 5.7 Hz, 2H), 8.56 (t, *J* = 7.8 Hz, 1H), 8.07 (t, *J* = 6.9 Hz, 2H), 7.78 (d, *J* = 8.2 Hz, 2H), 7.69 (d, *J* = 7.3 Hz, 2H), 7.59 – 7.48 (m, 4H), 7.44 (t, *J* = 7.3 Hz, 1H), 5.78 (s, 2H).

^{13}C NMR (101 MHz, CD_3CN) δ 147.29, 145.47, 143.34, 140.59, 132.95, 130.73, 130.04, 129.66, 129.05, 128.90, 127.99, 65.13.

HRMS-ESI(+) Calcd. for $C_{18}H_{16}N^+$ $[M]^+$: 246.1277; Found: 246.1284.

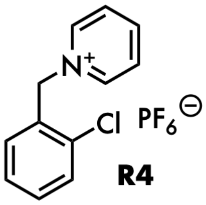

R4

^{1}H NMR (400 MHz, CD_3CN) δ 8.72 (d, J = 5.9 Hz, 2H), 8.53 (t, J = 7.8 Hz, 1H), 8.03 (t, J = 6.7 Hz, 2H), 7.65 – 7.37 (m, 4H), 5.82 (s, 2H).

^{13}C NMR (101 MHz, $CDCl_3$) δ 147.52, 145.68, 135.48, 133.43, 133.05, 131.41, 131.17, 129.50, 129.23, 63.26.

HRMS-ESI(+) Calcd. for $C_{12}H_{11}ClN^+$ $[M]^+$: 204.0575; Found: 204.0584.

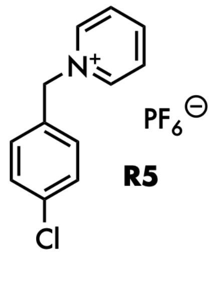

R5

^{1}H NMR (400 MHz, CD_3CN) δ 8.71 (d, J = 6.1 Hz, 2H), 8.52 (t, J = 7.9 Hz, 1H), 8.03 (t, J = 6.8 Hz, 2H), 7.49 (d, J = 8.6 Hz, 2H), 7.43 (d, J = 8.4 Hz, 2H), 5.68 (s, 2H).

^{13}C NMR (101 MHz, CD_3CN) δ 147.38, 145.52, 131.98, 130.51, 129.67, 64.55.

HRMS-ESI(+) Calcd. for $C_{12}H_{11}ClN^+$ $[M]^+$: 204.0575; Found: 204.0556.

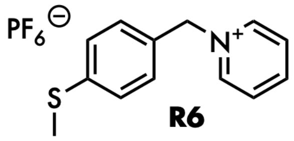

R6

^{1}H NMR (400 MHz, CD_3CN) δ 8.71 (d, J = 5.9 Hz, 2H), 8.50 (t, J = 7.8 Hz, 1H), 8.01 (t, J = 6.9 Hz, 2H), 7.37 (d, J = 8.4 Hz, 2H), 7.35 – 7.30 (m, 2H), 5.65 (s, 2H), 2.49 (s, 3H).

^{13}C NMR (101 MHz, CD_3CN) δ 147.19, 145.33, 142.49, 130.82, 129.94, 129.58, 127.28, 65.02, 15.10.

HRMS-ESI(+) Calcd. for $C_{13}H_{14}NS^+$ $[M]^+$: 216.0841; Found: 216.0873.

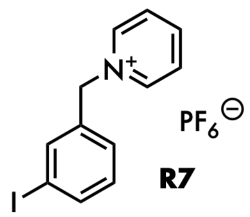

R7

^{1}H NMR (400 MHz, CD_3CN) δ 8.72 (d, J = 5.9 Hz, 2H), 8.52 (t, J = 7.8 Hz, 1H), 8.03 (t, J = 6.9 Hz, 2H), 7.84 (d, J = 8.6 Hz, 2H), 7.44 (d, J = 7.7 Hz, 1H), 7.24 (t, J = 7.7 Hz, 1H), 5.64 (s, 2H).

^{13}C NMR (101 MHz, CD_3CN) δ 147.40, 145.55, 139.86, 139.03, 136.02, 132.26, 129.69, 95.36, 64.40.

HRMS-ESI(+) Calcd. for $C_{12}H_{11}IN^+$ $[M]^+$: 295.9931; Found: 295.9915.

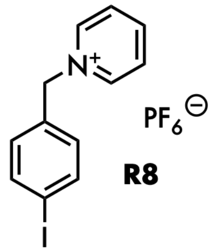

R8

^1H NMR (400 MHz, CD_3CN) δ 8.71 (d, J = 5.7 Hz, 2H), 8.52 (t, J = 7.8 Hz, 1H), 8.02 (t, J = 6.9 Hz, 2H), 7.84 (d, J = 8.3 Hz, 2H), 7.21 (d, J = 8.3 Hz, 2H), 5.65 (s, 2H).

^{13}C NMR (101 MHz, CD_3CN) δ 199.95, 147.38, 145.52, 139.55, 133.65, 132.11, 129.66, 96.46, 64.73.

HRMS-ESI(+) Calcd. for $C_{12}H_{11}IN^+$ $[M]^+$: 295.9931; Found: 295.9938

Control experiments

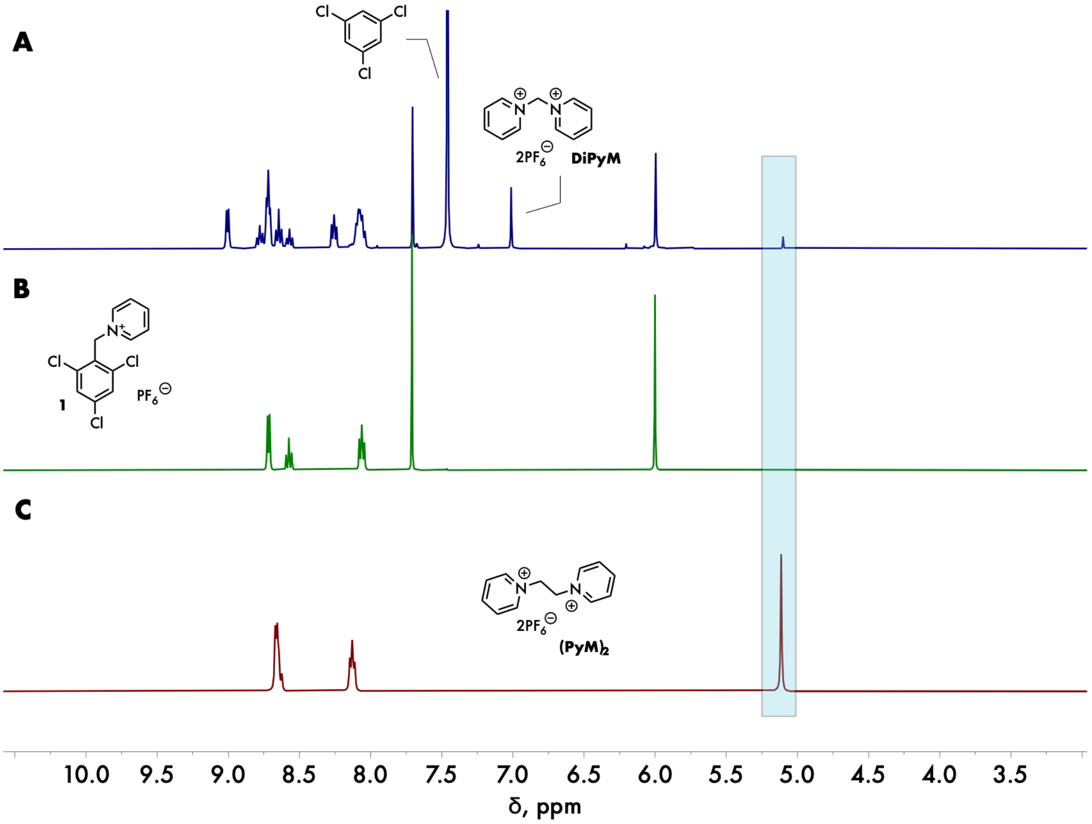

Figure S6. (A) ^1H NMR (400 MHz, CD_3CN) spectrum of 1,3,5-trichlorobenzene + **DiPyM 2PF$_6$** reaction mixture showing the formation of the product (B) and minor amount of (**PyM**)$_2$ dimer (C).

Kinetic Isotope Effect

Following **General Procedure I**: The reaction of **DiPyM 2PF$_6$** (92 mg, 0.2 mmol), C_6H_6 (78 mg, 1.0 mmol, 5.0 equiv.), C_6D_6 (84 mg, 1.0 mmol, 5.0 equiv.), photocatalyst [Ir(ppy)$_2$(dtbbpy)]PF$_6$ (1.8 mg, 2.0 μmol, 1 mol%) were combined and irradiated with 455 nm LEDs for 16 h. Crude reaction mixture was analyzed using ^1H NMR. KIE was determined to be 1.1.

Reaction with TEMPO trap

Following **General Procedure I**: The reaction of **DiPyM 2PF$_6$** (92 mg, 0.2 mmol), mesitylene (72 mg, 0.6 mmol, 3.0 equiv.), photocatalyst [Ir(ppy)$_2$(dtbbpy)]PF$_6$ (1.8 mg, 2.0 μmol, 1 mol%), TEMPO (31 mg, 0.2 mmol, 1.0 equiv.) were combined and irradiated with 455 nm LEDs for 16 h. Crude reaction mixture was analyzed using ^1H NMR. No target product detected. TEMPO effectively quenches productive reaction (**Figure S7**).

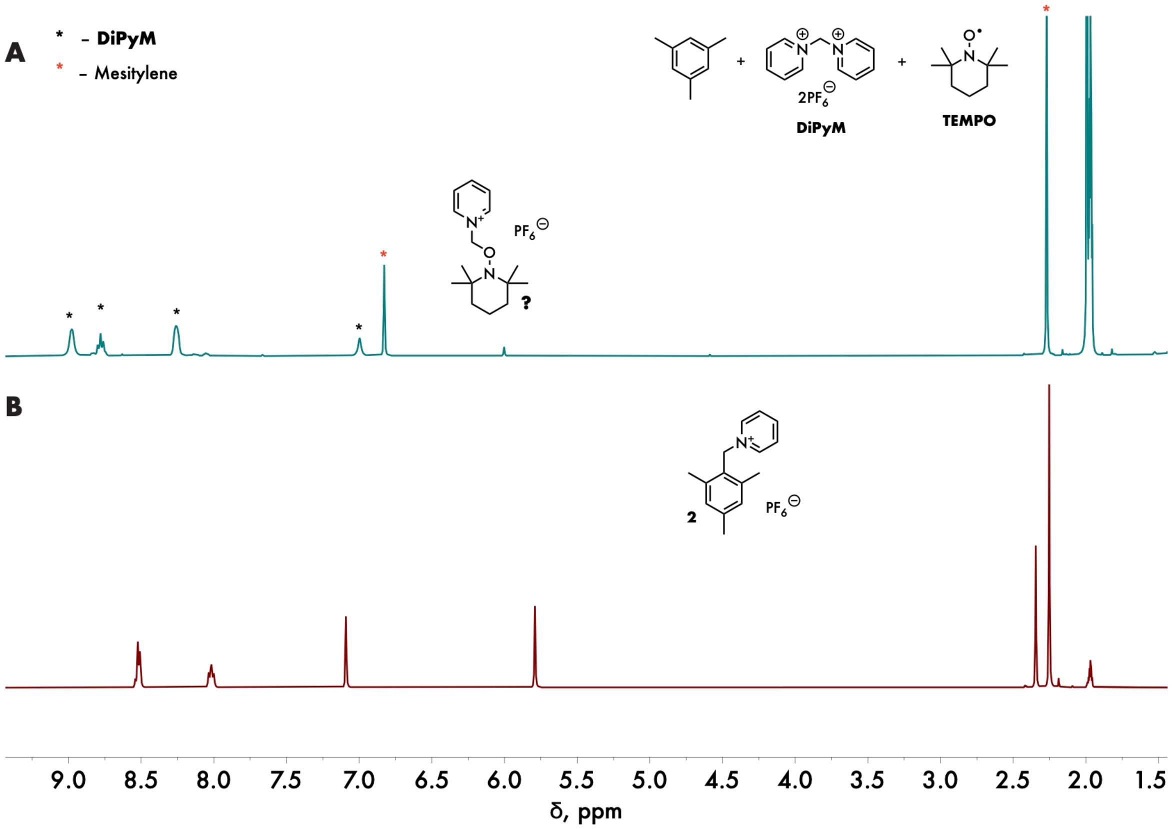

Figure S7. (A) ^{1}H NMR (400 MHz, CD_3CN) spectrum of mesitylene + **DiPyM 2PF$_6$** + **TEMPO** reaction mixture showing no formation of the expected product **2** (B).

Reaction with pyridinium HPF$_6$ substrate

Following **General Procedure I**: The reaction of **DiPyM 2PF$_6$** (92 mg, 0.2 mmol), pyridinium HPF_6 (225 mg, 1.0 mmol, 5.0 equiv.), photocatalyst $[Ir(ppy)_2(dtbbpy)]PF_6$ (1.8 mg, 2.0 µmol, 1 mol%), were combined and irradiated with 455 nm LEDs for 16 h. Crude reaction mixture was analyzed using ^{1}H NMR. No conversion of the **DiPyM 2PF$_6$** was observed.

Reaction without arene substrate

Following **General Procedure I**: The reaction of **DiPyM 2PF$_6$** (92 mg, 0.2 mmol), photocatalyst $[Ir(ppy)_2(dtbbpy)]PF_6$ (1.8 mg, 2.0 µmol, 1 mol%), were combined and irradiated with 455 nm LEDs for 16 h. Crude reaction mixture was analyzed using ^{1}H NMR. No conversion of the **DiPyM 2PF$_6$** was observed.

Reaction with pyridine substrate

Following **General Procedure I**: The reaction of **DiPyM 2PF$_6$** (92 mg, 0.2 mmol), pyridine (158 mg, 2.0 mmol, 10.0 equiv.) photocatalyst $[Ir(ppy)_2(dtbbpy)]PF_6$ (1.8 mg, 2.0 µmol, 1 mol%), were combined and irradiated with 455 nm LEDs for 16 h. Crude reaction mixture was analyzed using ^{1}H NMR. No conversion of the **DiPyM 2PF$_6$** was observed.

Determination of excited state reduction potentials of photocatalysts TBA 1-3

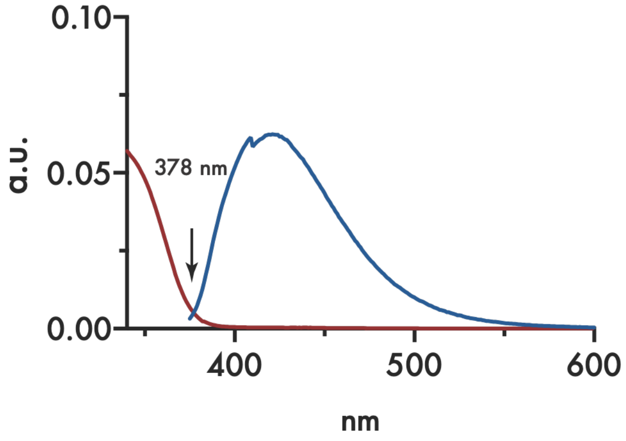

Figure S8. Normalized absorption and emission spectra of **TBA-1**. Excited state redox potential $E_{1/2}^{+1/0}$ = +0.92 V (ref. 5a) – 3.28 V = -2.36 V.

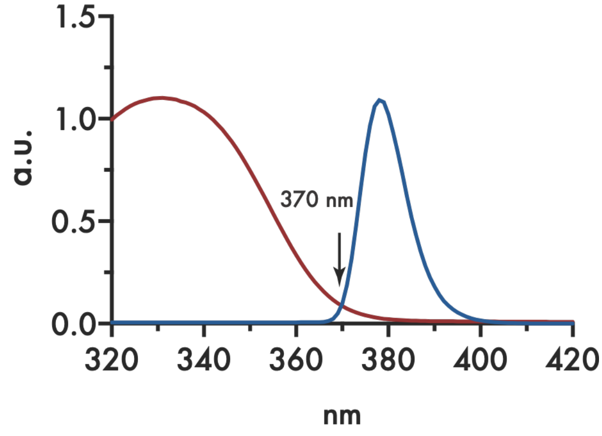

Figure S9. Normalized absorption and emission spectra of **TBA-2**. Excited state redox potential $E_{1/2}^{+1/0}$ = +0.98 V (ref. 5a) – 3.35 V = -2.37 V.

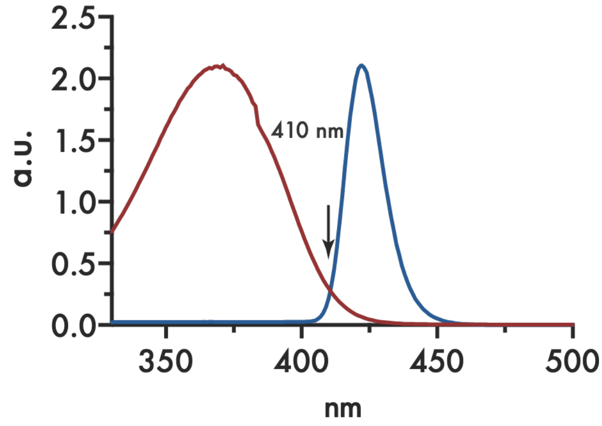

Figure S10. Normalized absorption and emission spectra of **TBA-3**. Excited state redox potential $E_{1/2}^{+1/0}$ = +1.03 (ref. 5a) – 3.02 V = -1.99 V.

Copies of NMR spectra

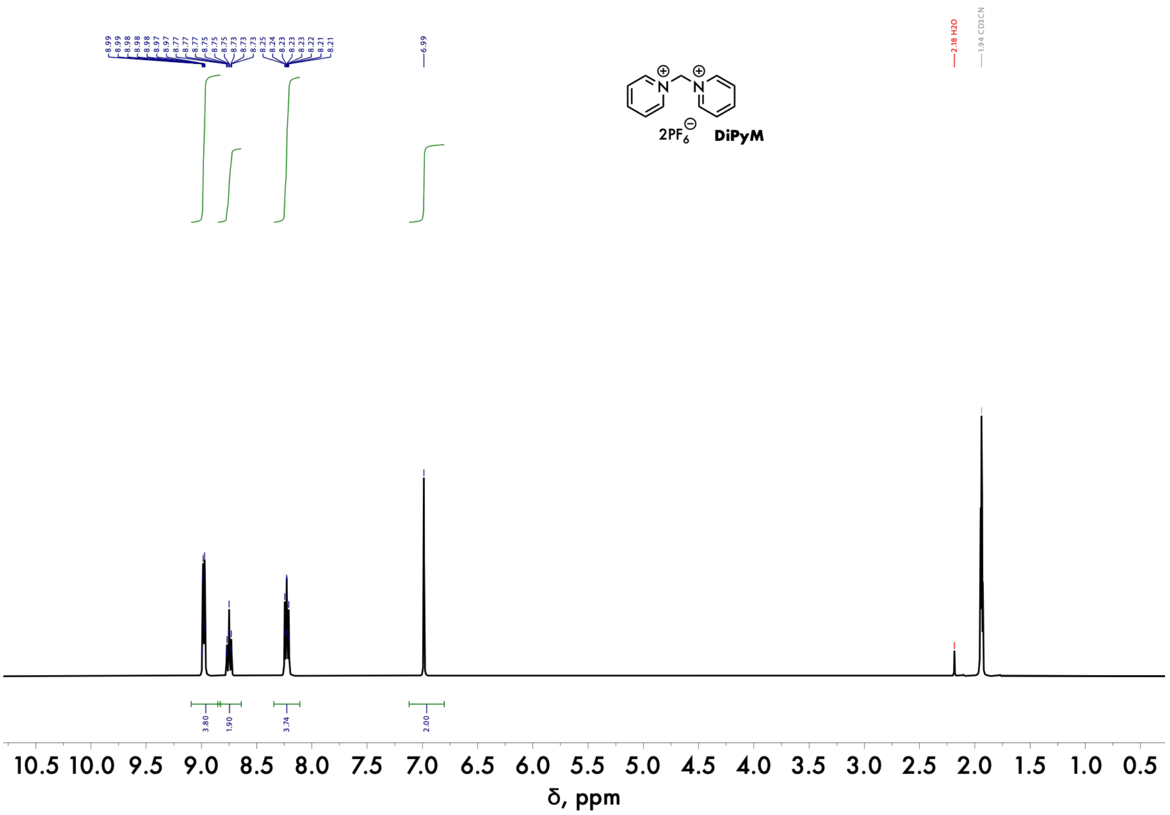

Figure S11. ^{1}H NMR (400 MHz, CD_3CN) spectrum of **DiPyM 2PF₆**.

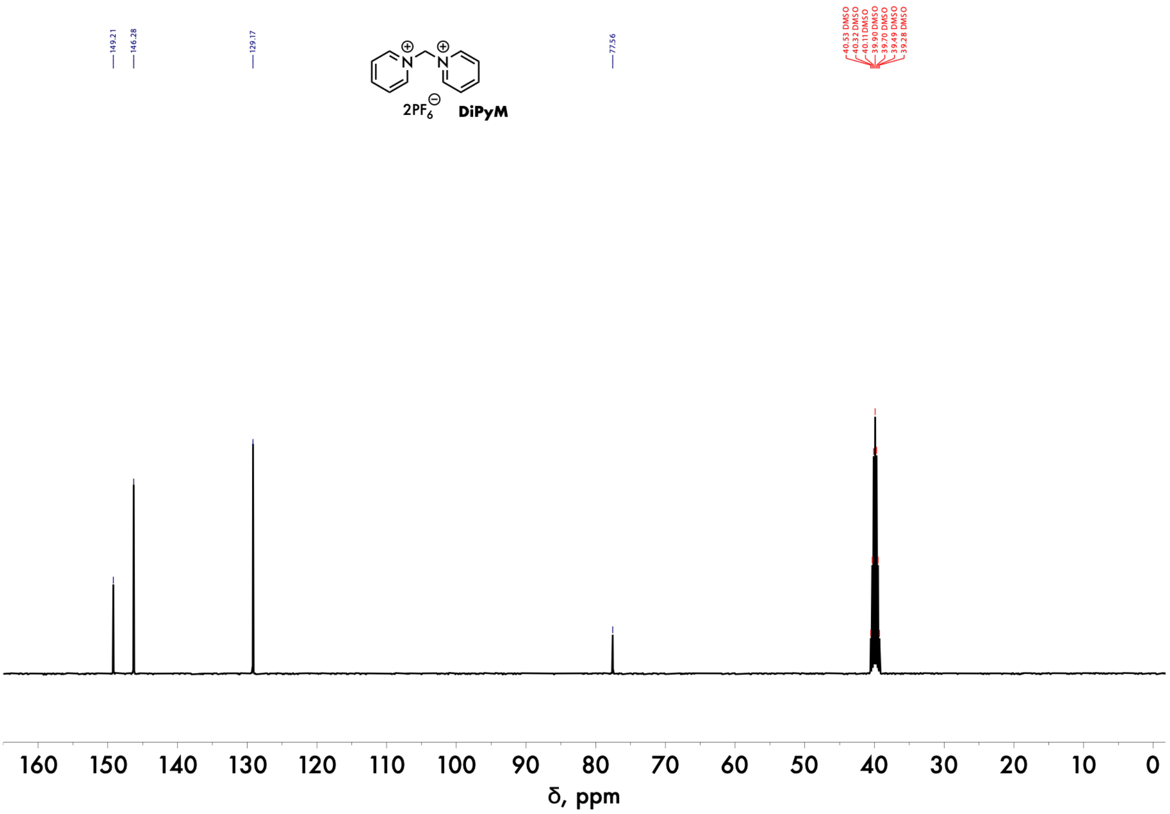

Figure S12. ^{13}C NMR (101 MHz, d_6-DMSO) spectrum of **DiPyM 2PF₆**.

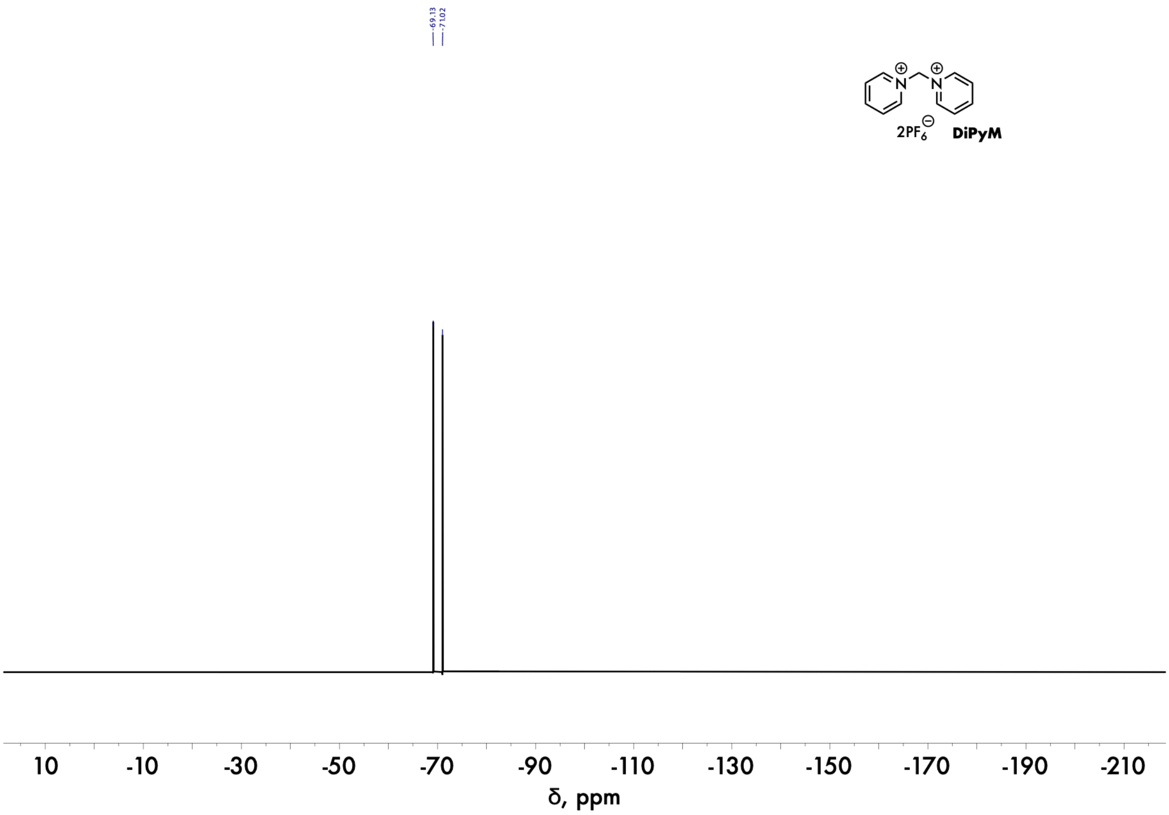

Figure S13. ^{19}F NMR (376 MHz, d_6-DMSO) spectrum of **DiPyM 2PF$_6$**.

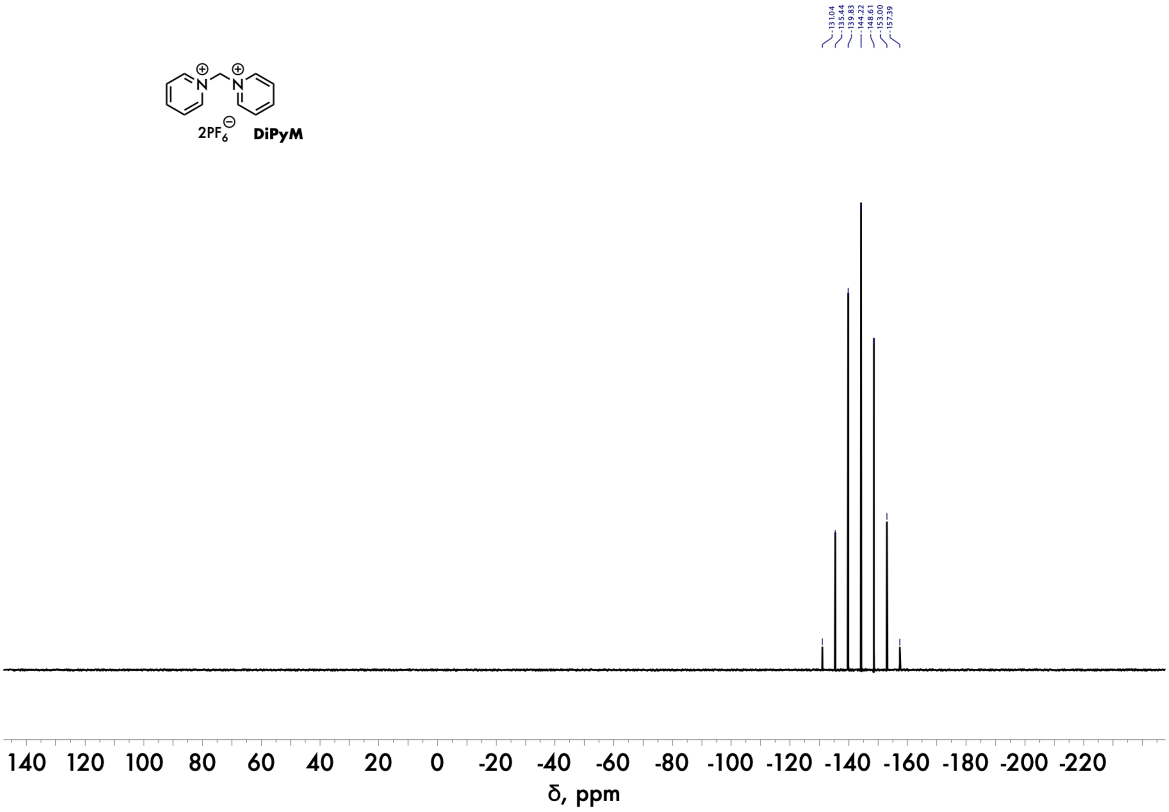

Figure S14. ^{31}P NMR (162 MHz, d_6-DMSO) spectrum of **DiPyM 2PF$_6$**.

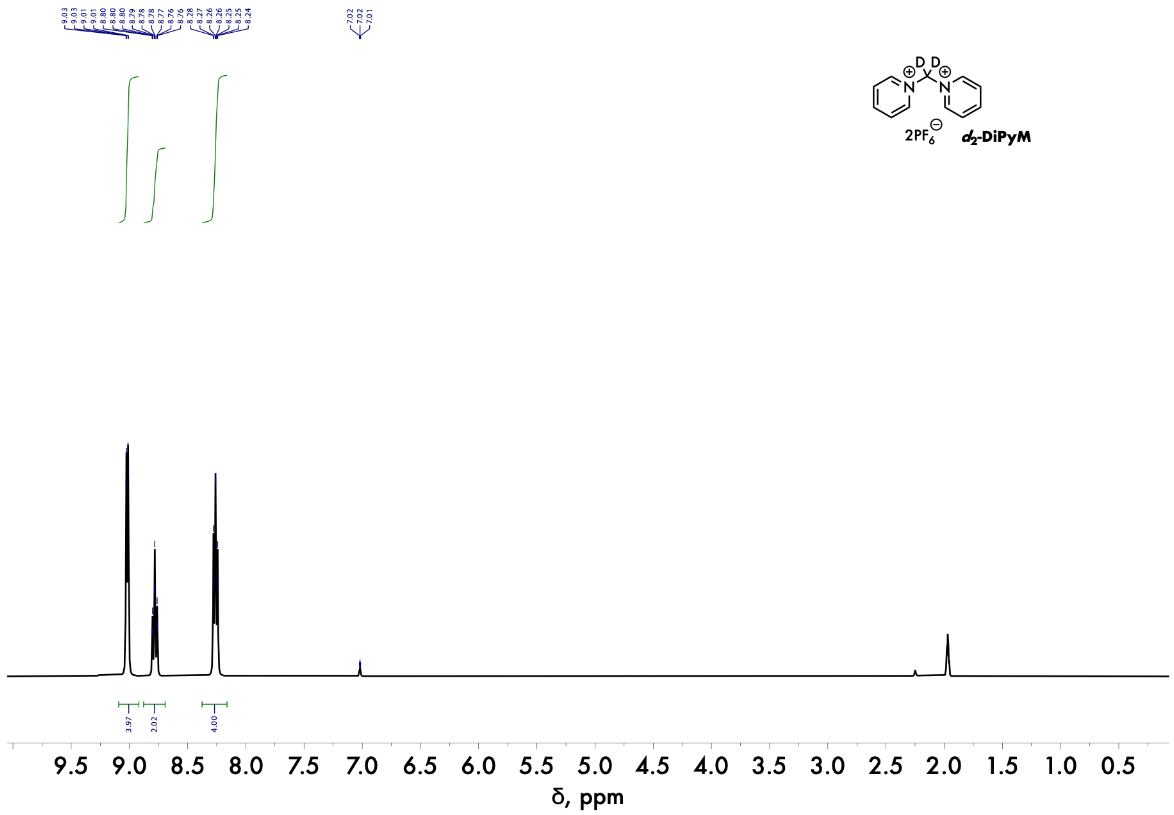

Figure S15. ^{1}H NMR (400 MHz, CD_3CN) spectrum of ***d*₂-DiPyM 2PF₆**.

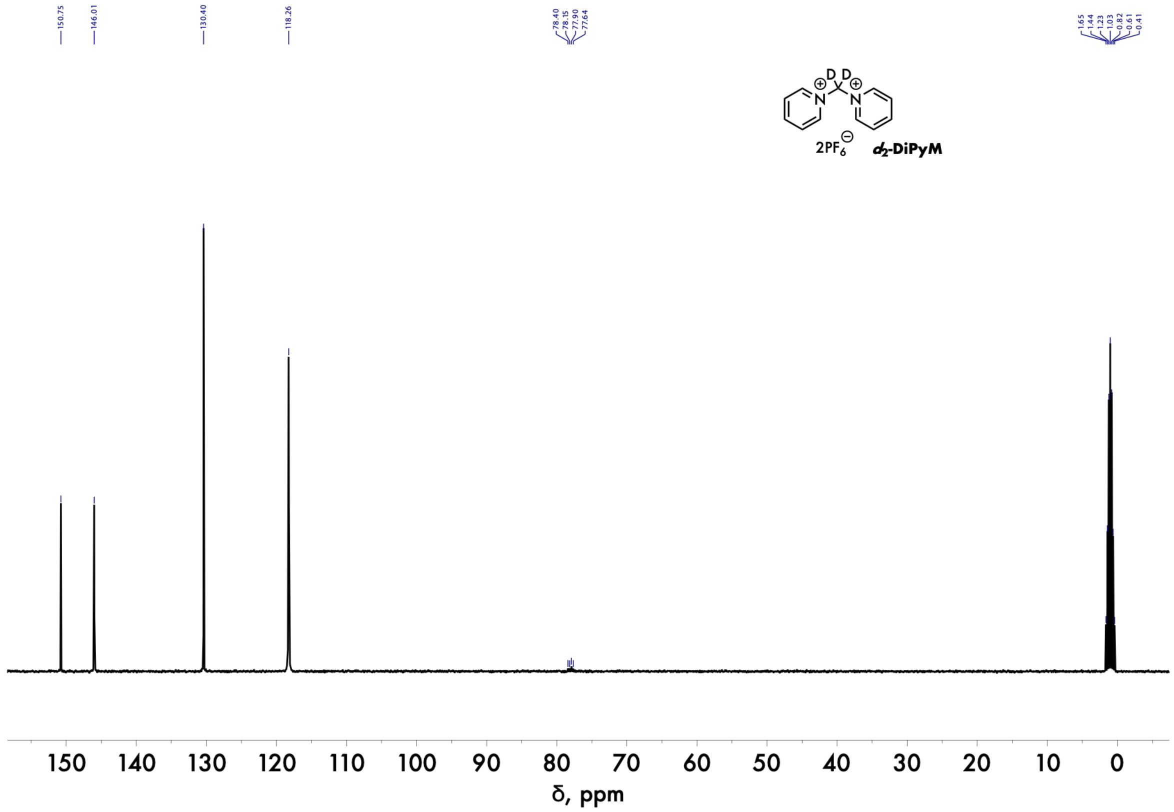

Figure S16. ^{13}C NMR (101 MHz, CD_3CN) spectrum of ***d*₂-DiPyM 2PF₆**.

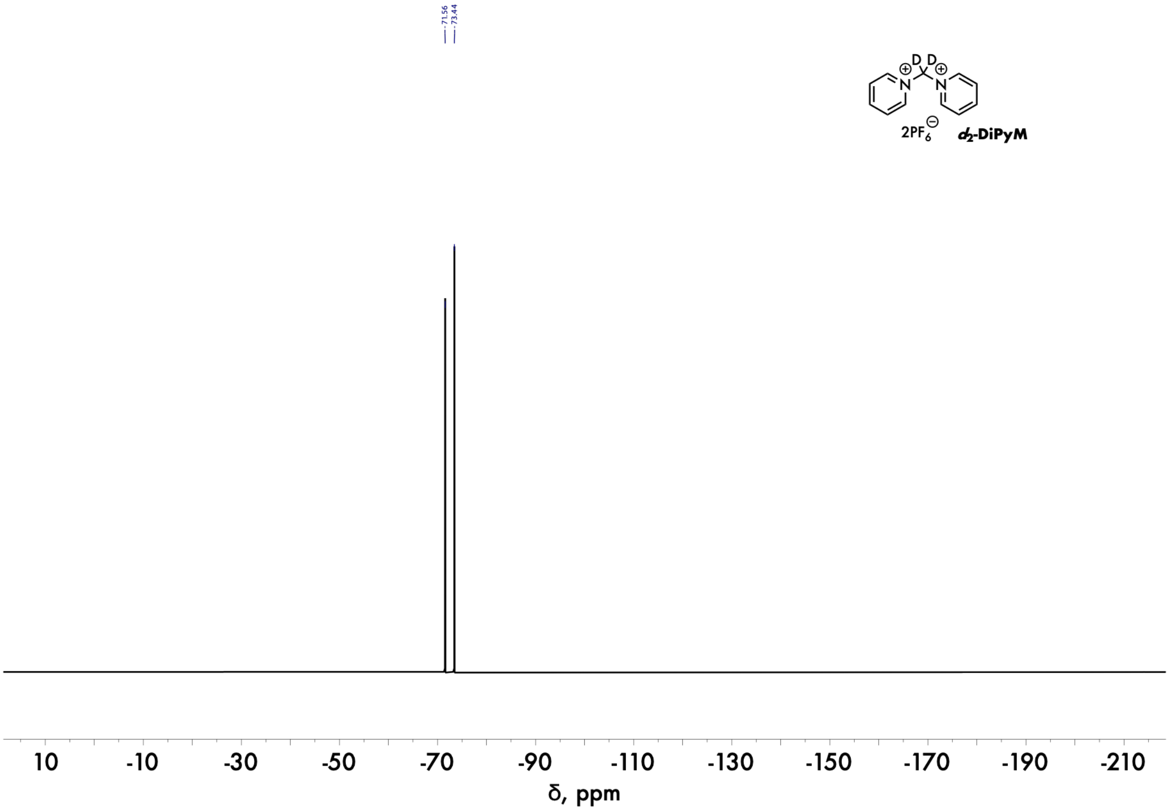

Figure S17. ^{19}F NMR (376 MHz, CD_3CN) spectrum of ***d*₂-DiPyM 2PF₆**.

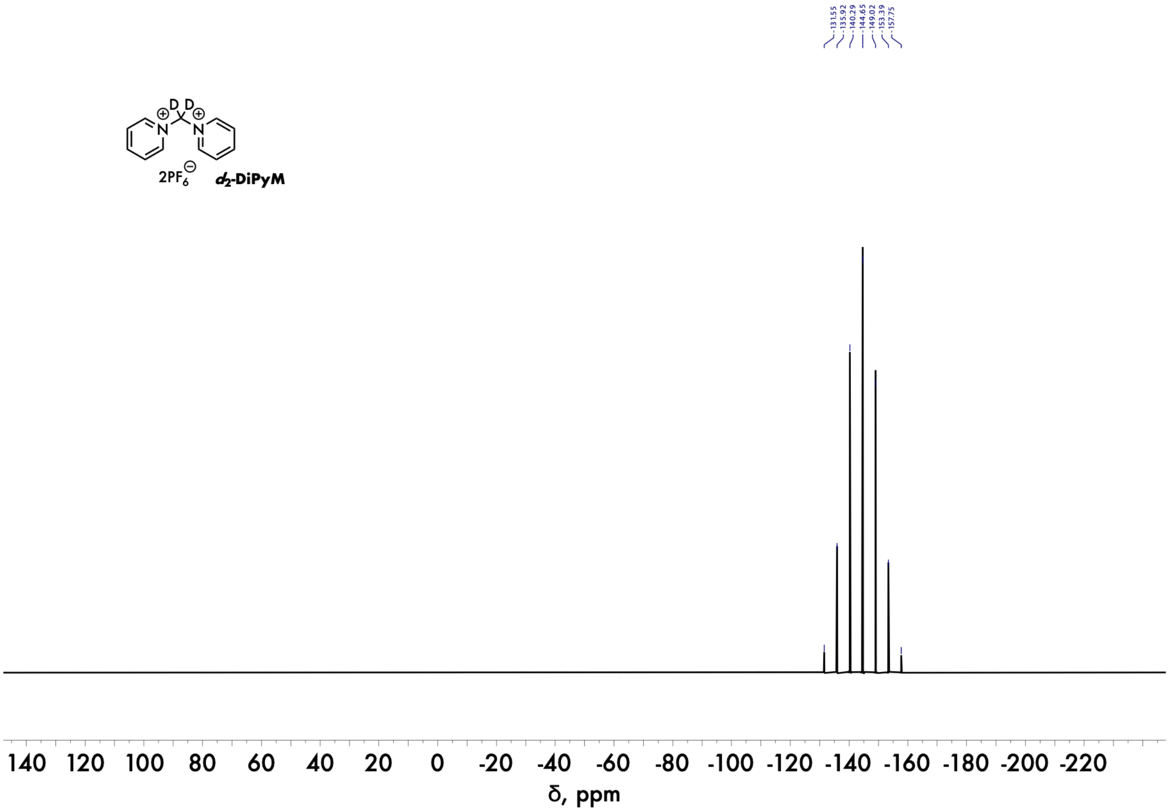

Figure S18. ^{31}P NMR (162 MHz, CD_3CN) spectrum of ***d*₂-DiPyM 2PF₆**.

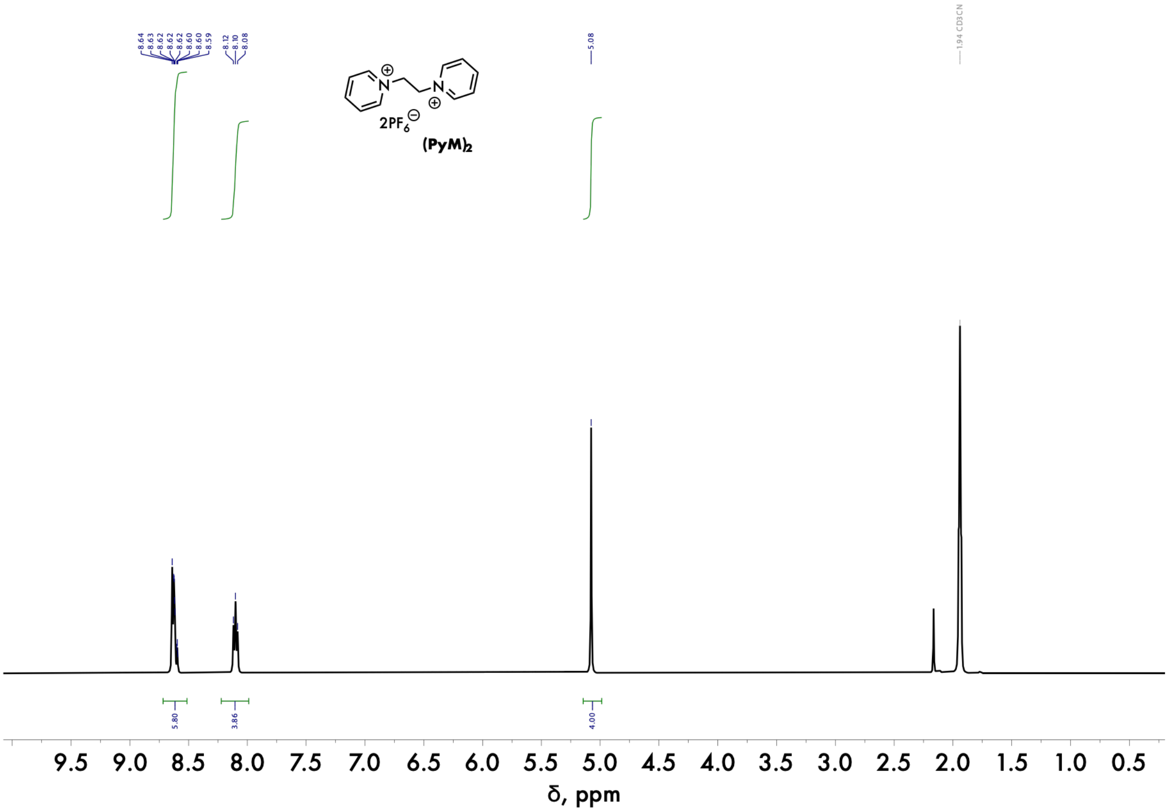

Figure S19. ^{1}H NMR (400 MHz, CD_3CN) spectrum of **(PyM)$_2$ 2PF$_6$**.

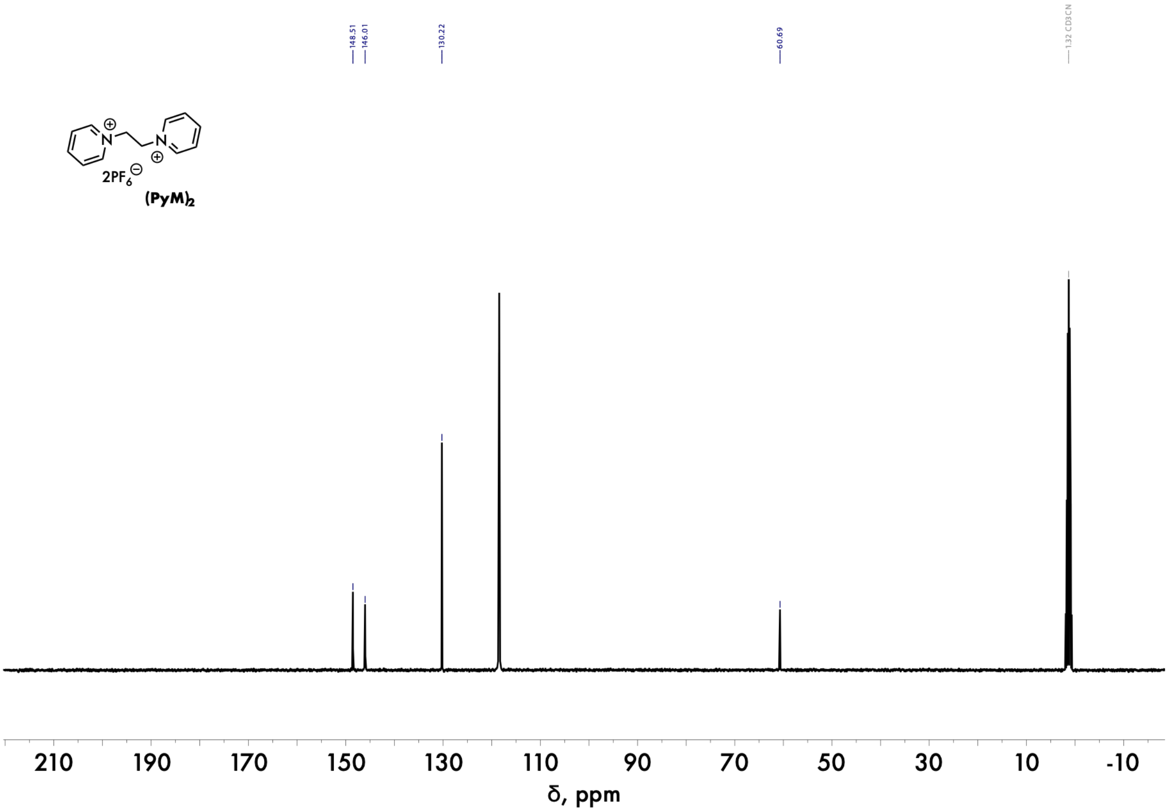

Figure S20. ^{13}C NMR (101 MHz, CD_3CN) spectrum of (**PyM**)$_2$.

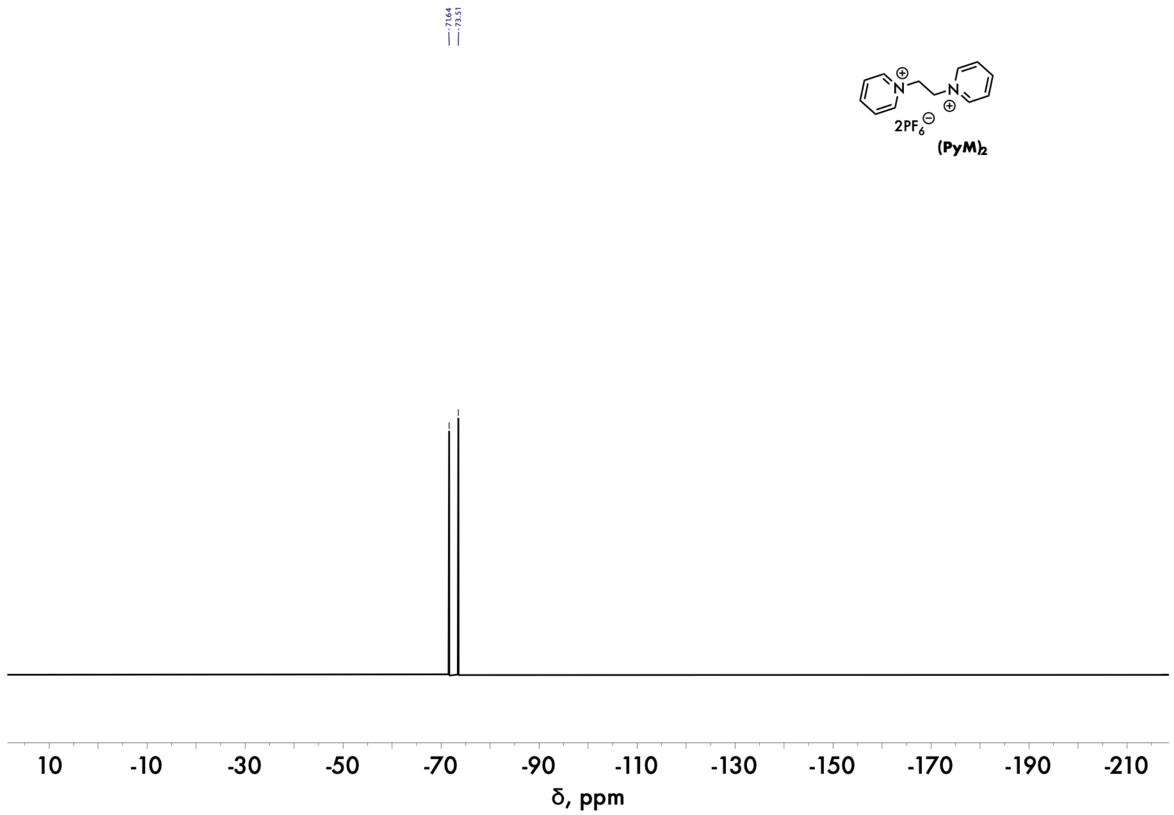

Figure S21. ^{19}F NMR (376 MHz, CD_3CN) spectrum of (**PyM**)$_2$ **2PF$_6$**

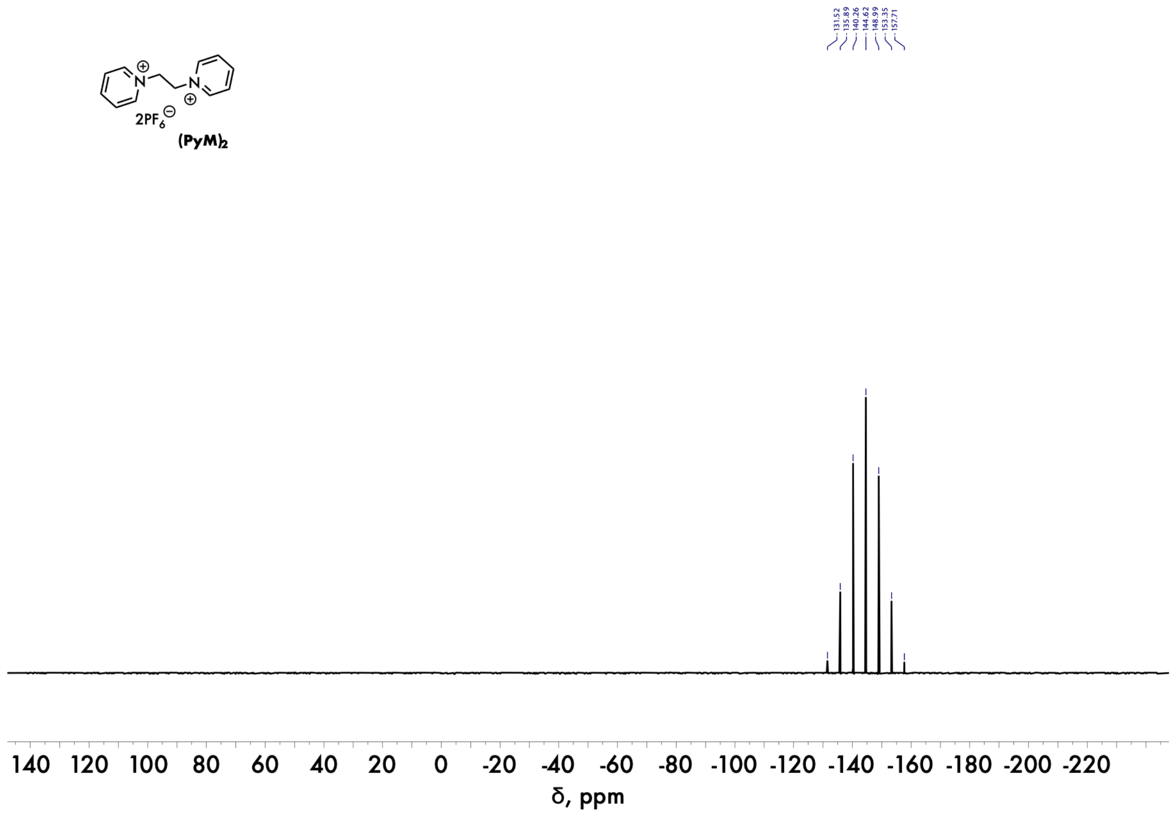

Figure S22. ^{31}P NMR (162 MHz, CD_3CN) spectrum of (**PyM**)$_2$ **2PF$_6$**.

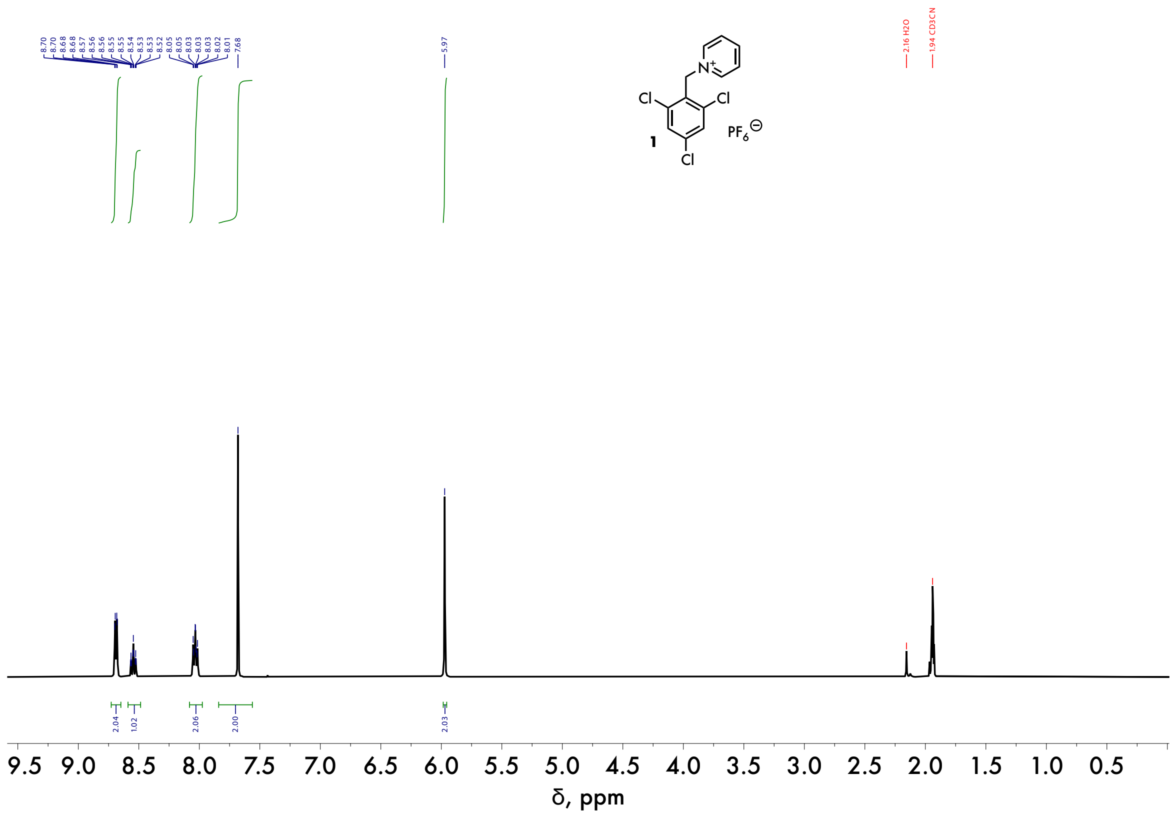

Figure S23. ^{1}H NMR (400 MHz, CD_3CN) spectrum of **1**.

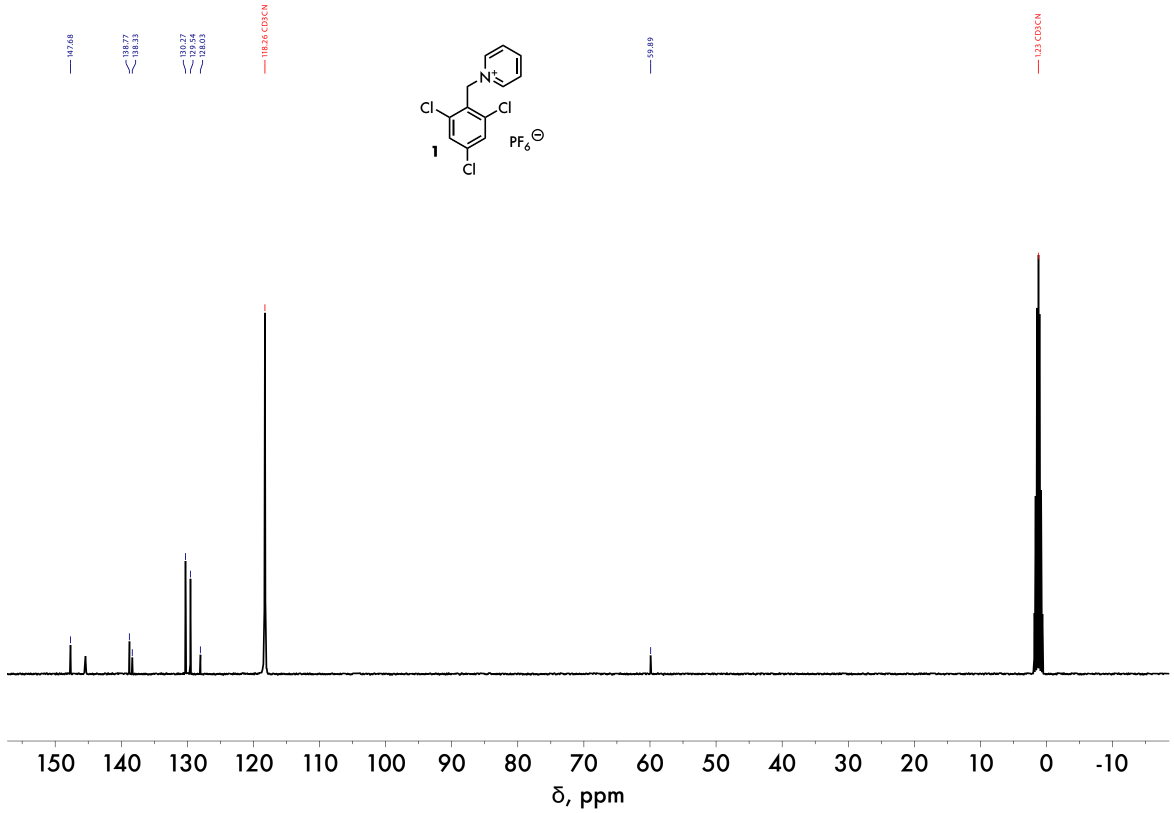

Figure S24. ^{13}C NMR (101 MHz, CD_3CN) spectrum of **1**.

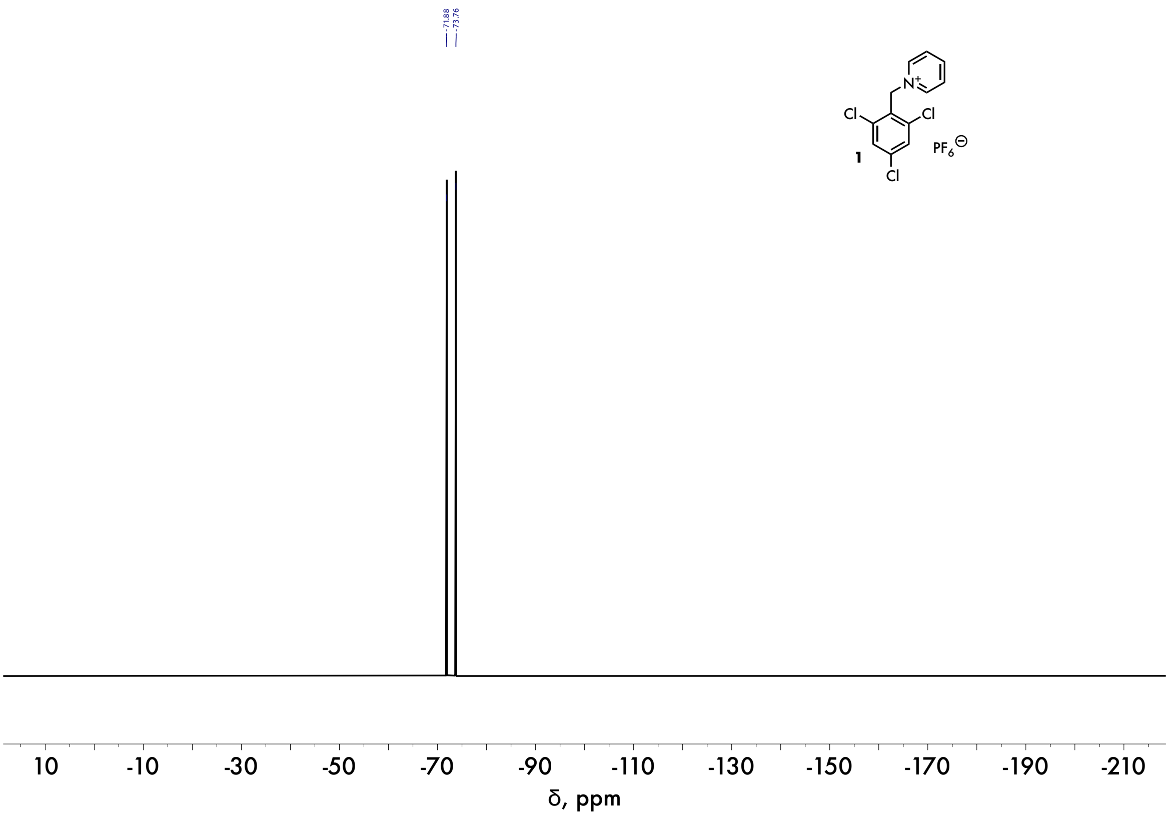

Figure S25. ^{19}F NMR (376 MHz, CD_3CN) spectrum of **1**.

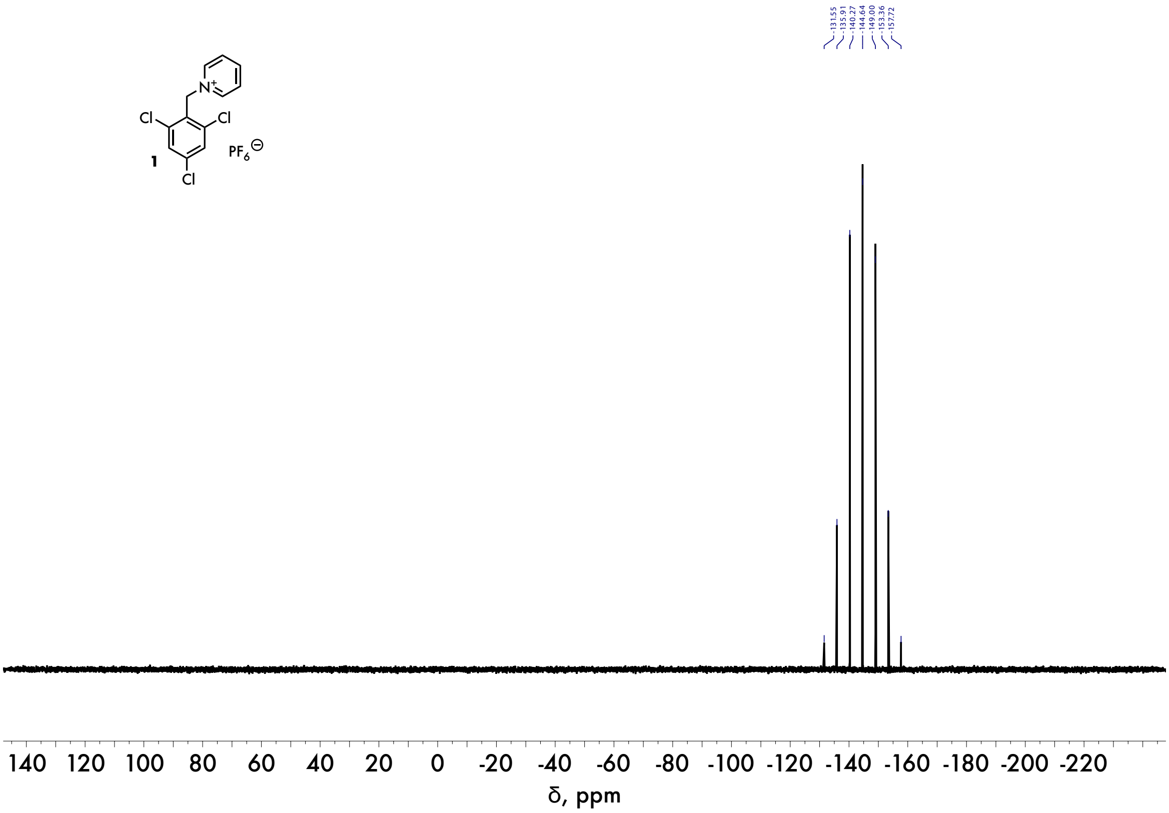

Figure S26. ^{31}P NMR (162 MHz, CD_3CN) spectrum of **1**.

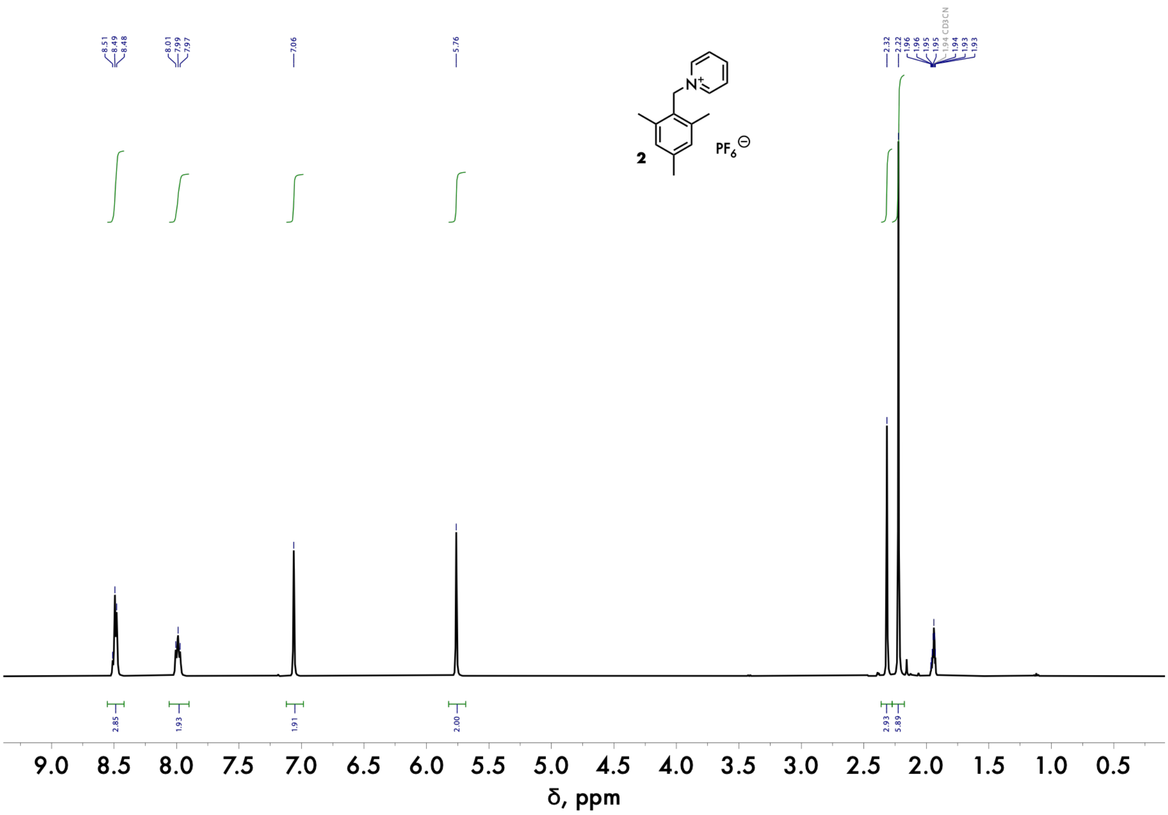

Figure S27. ^{1}H NMR (400 MHz, CD_3CN) spectrum of **2**.

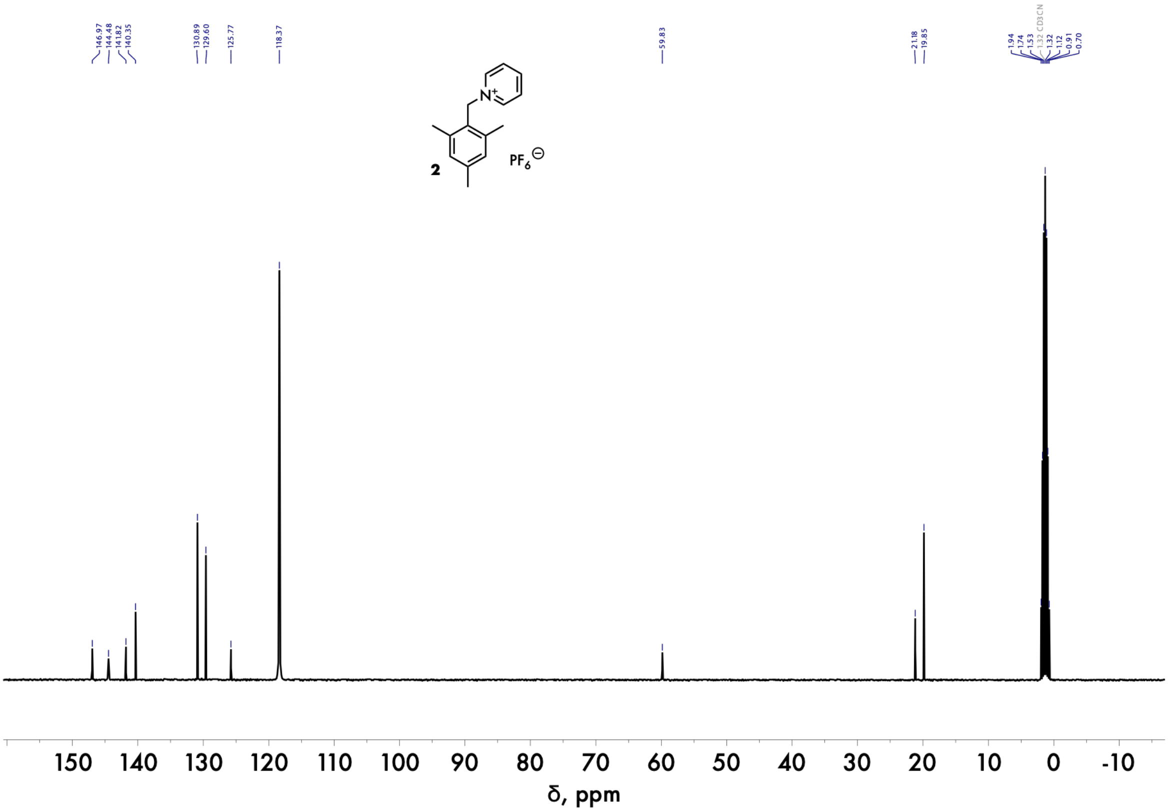

Figure S28. ^{13}C NMR (101 MHz, CD_3CN) spectrum of **2**.

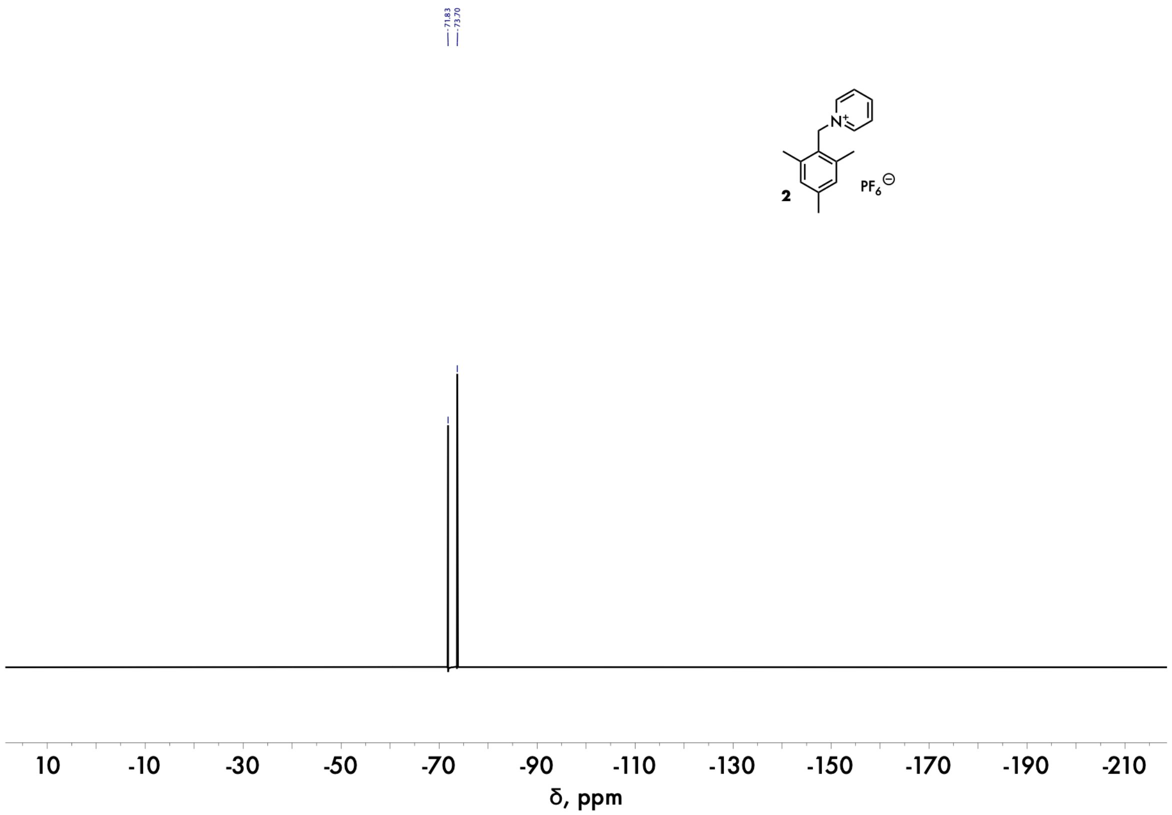

Figure S29. ^{19}F NMR (376 MHz, CD_3CN) spectrum of **2**.

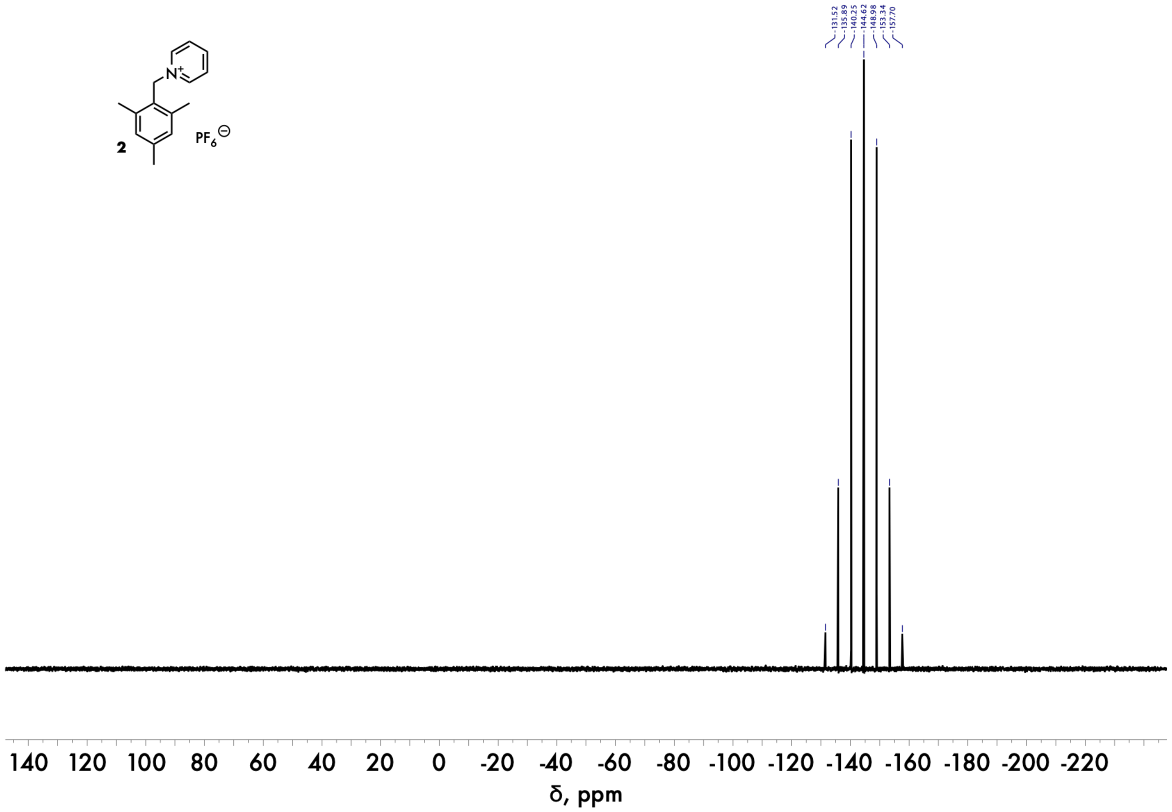

Figure S30. ^{31}P NMR (162 MHz, CD_3CN) spectrum of **2**.

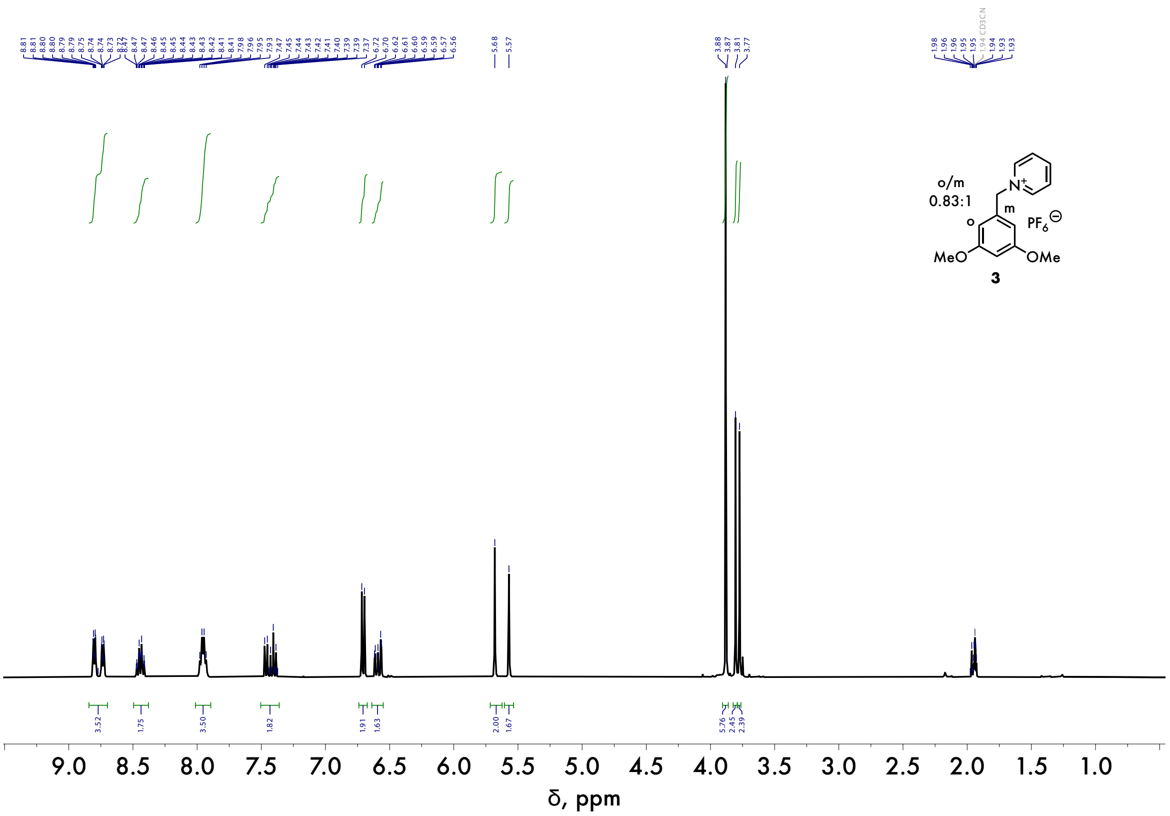

Figure S31. ^{1}H NMR (400 MHz, CD_3CN) spectrum of **3**.

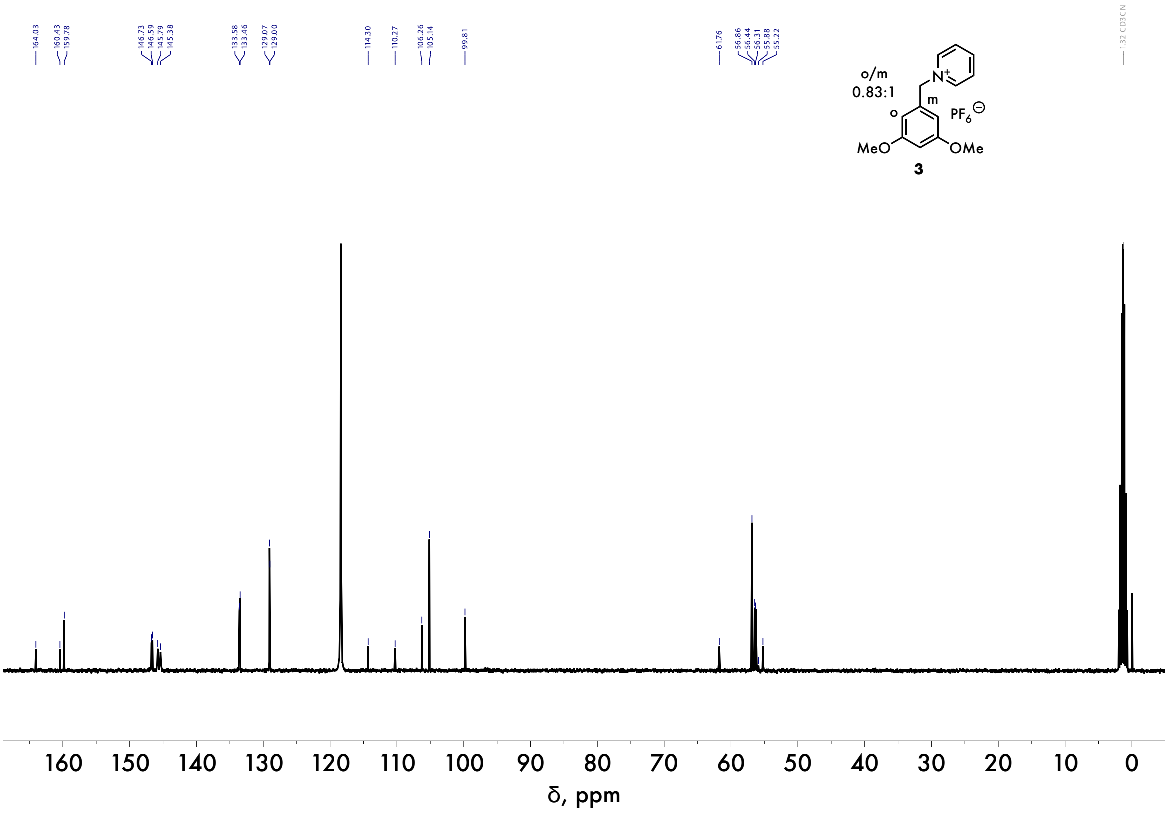

Figure S32. ^{13}C NMR (101 MHz, CD_3CN) spectrum of **3**.

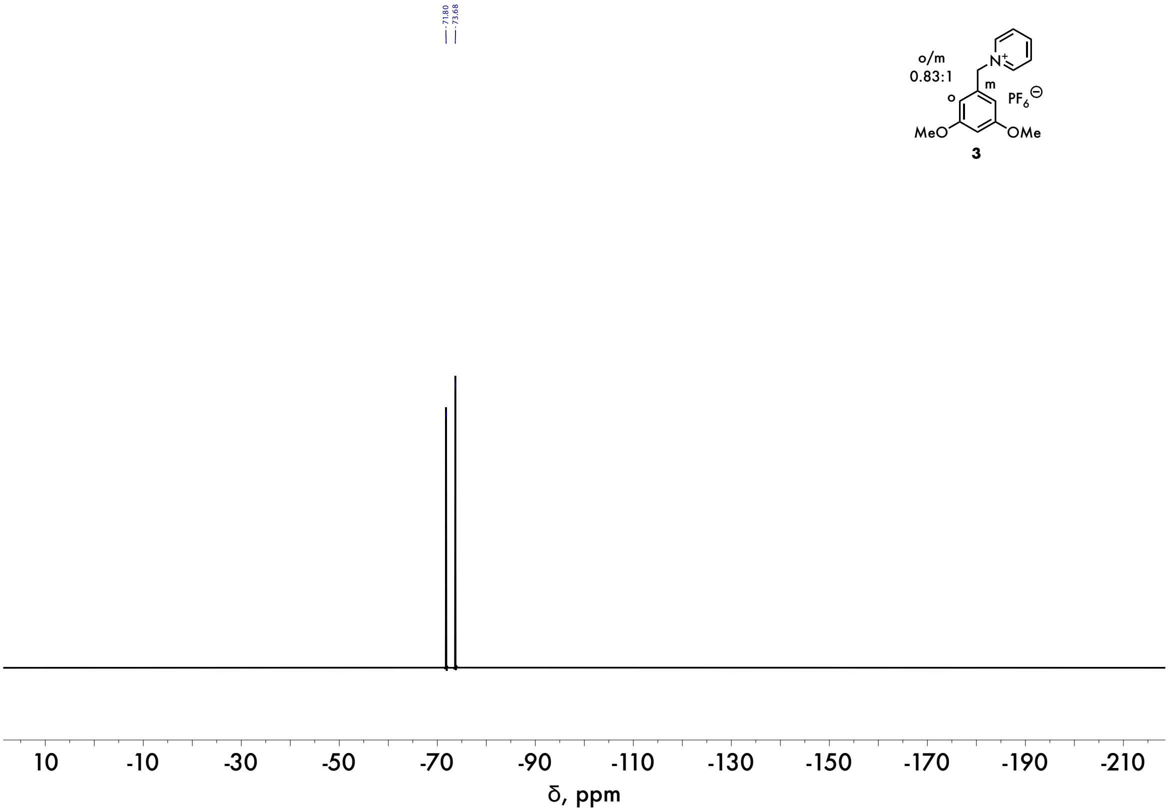

Figure S33. ^{19}F NMR (376 MHz, CD_3CN) spectrum of **3**.

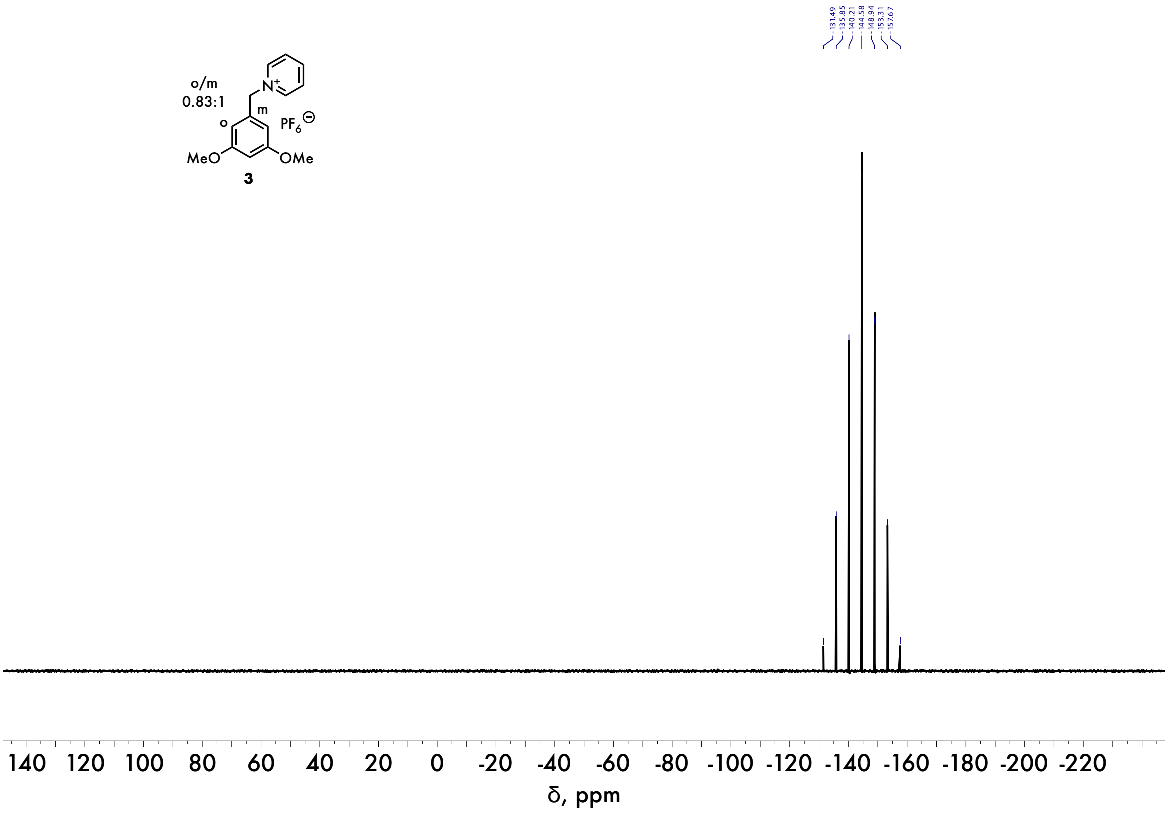

Figure S34. ^{31}P NMR (162 MHz, CD_3CN) spectrum of **2**.

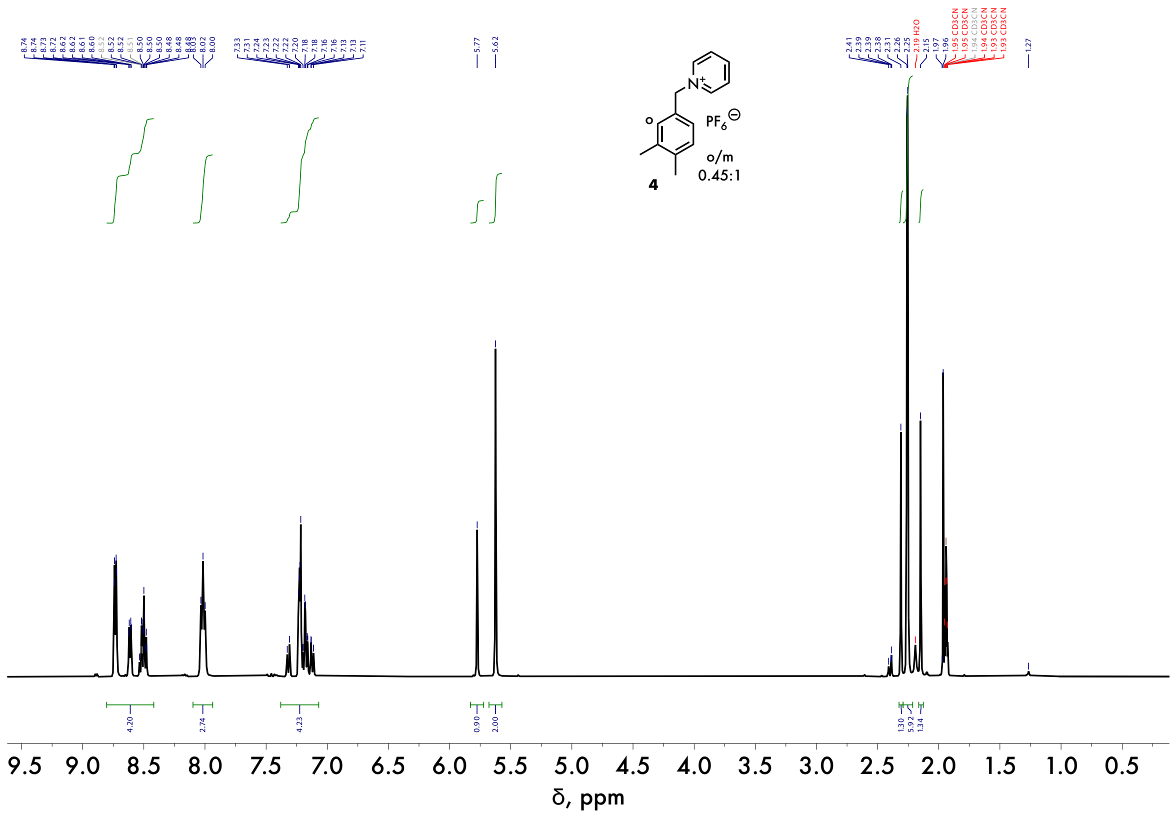

Figure S35. ^{1}H NMR (400 MHz, CD_3CN) spectrum of **4**.

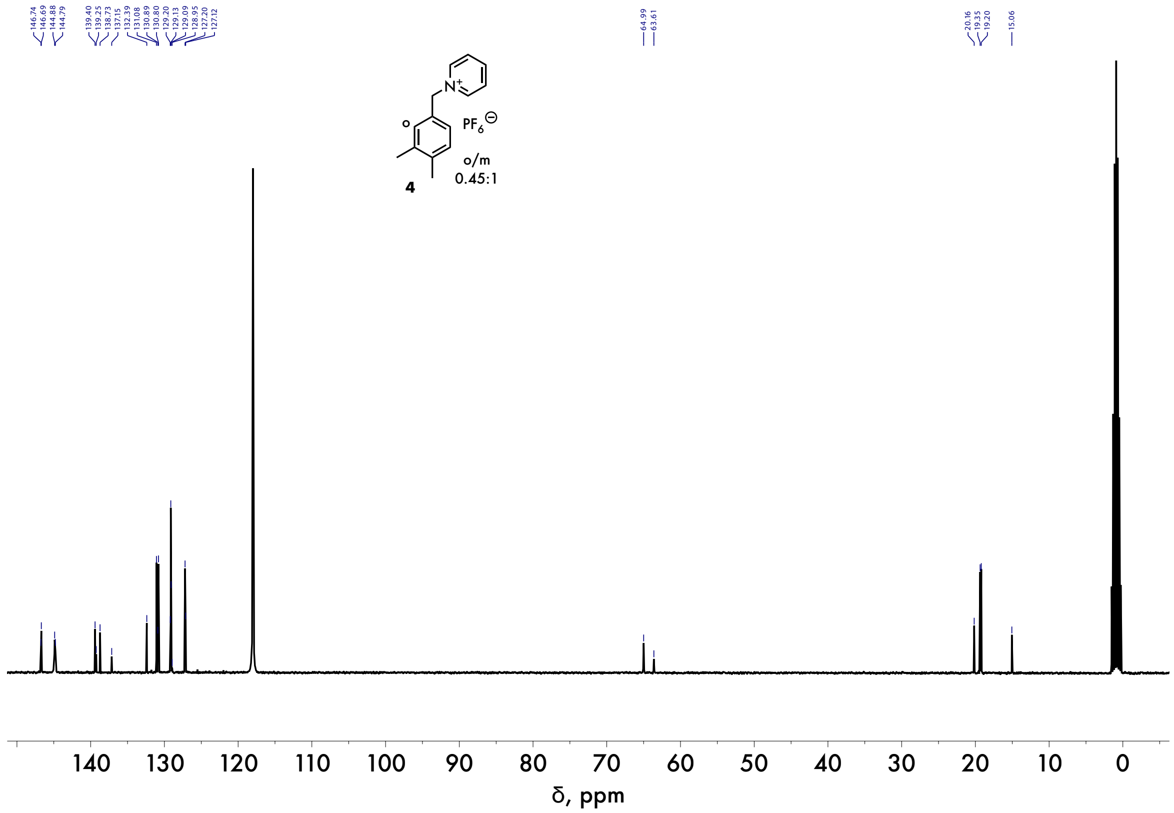

Figure S36. ^{13}C NMR (101 MHz, CD_3CN) spectrum of **4**.

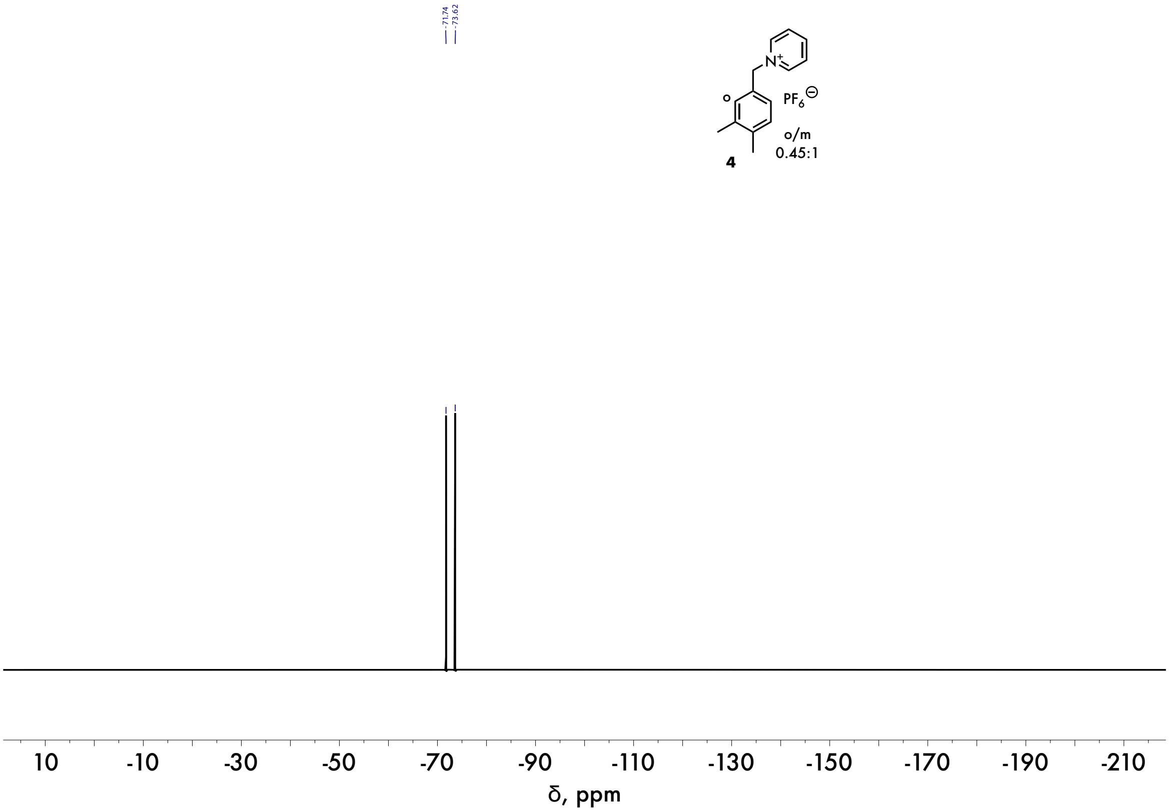

Figure S37. ^{19}F NMR (376 MHz, CD_3CN) spectrum of **4**.

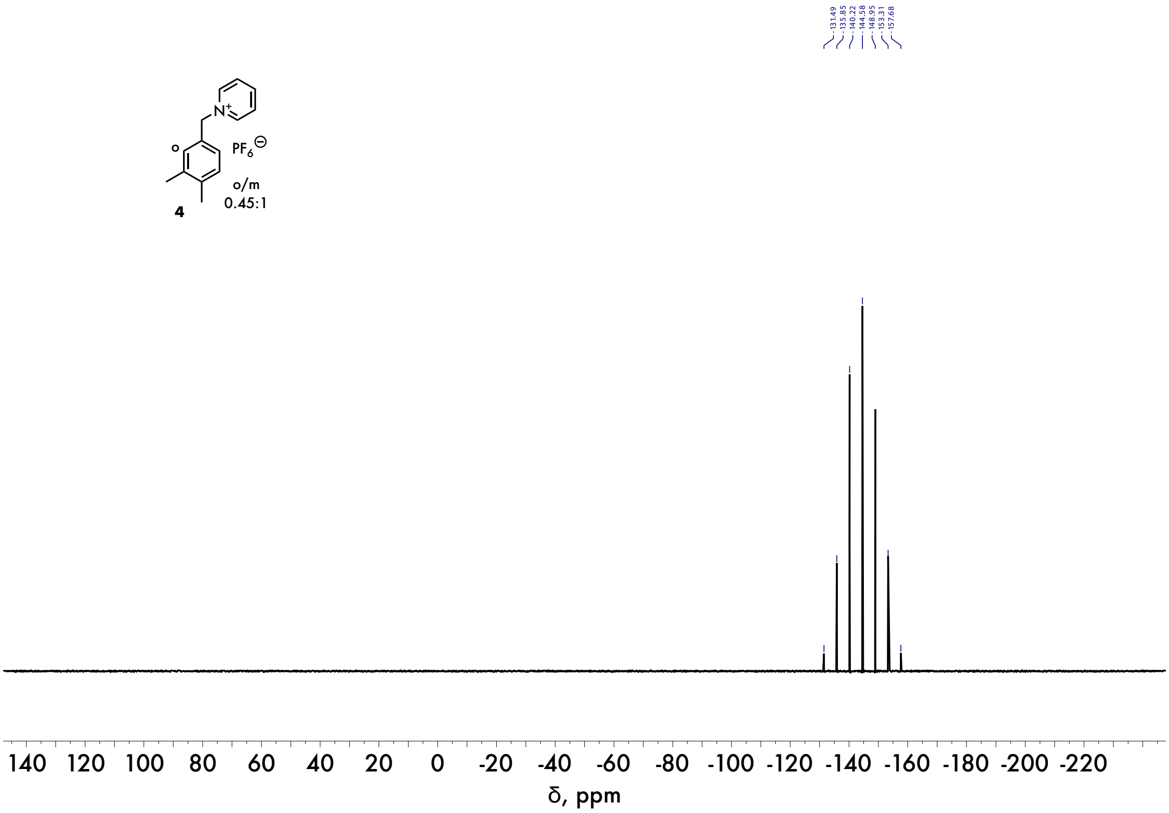

Figure S38. ^{31}P NMR (162 MHz, CD_3CN) spectrum of **4**.

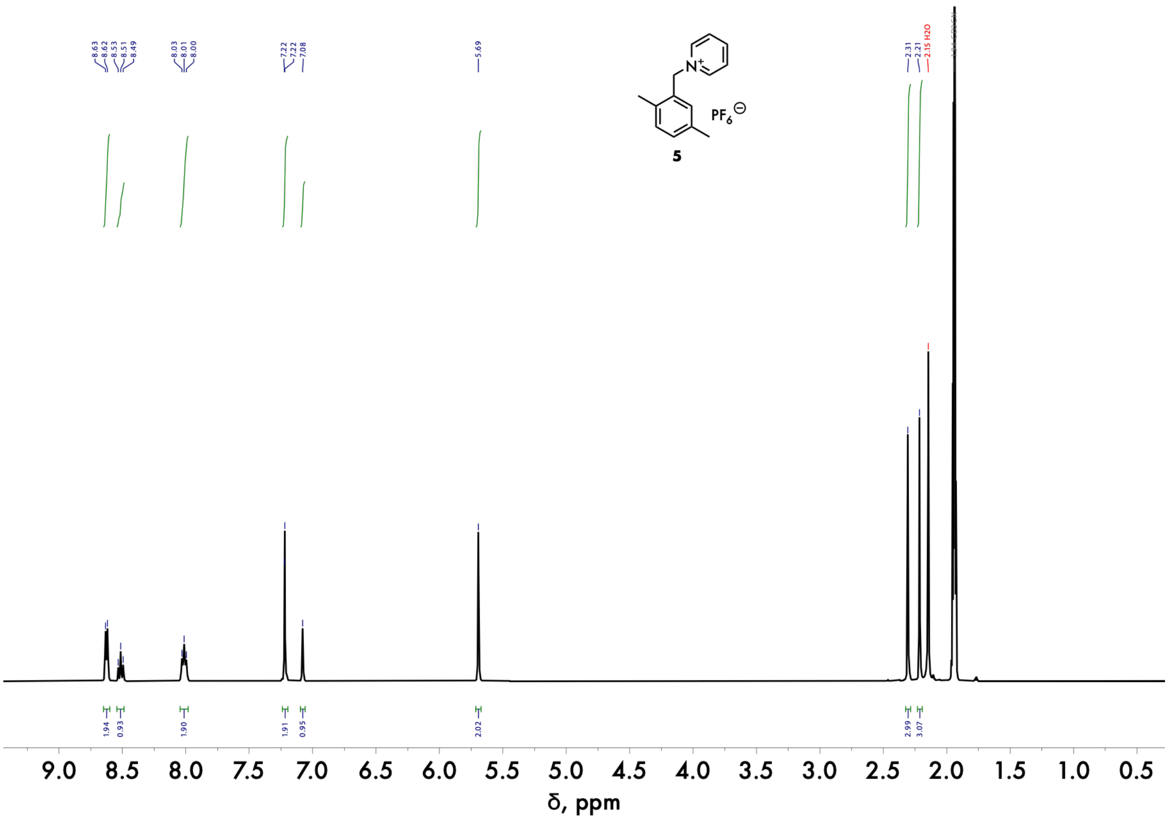

Figure S39. ^{1}H NMR (400 MHz, CD_3CN) spectrum of **5**.

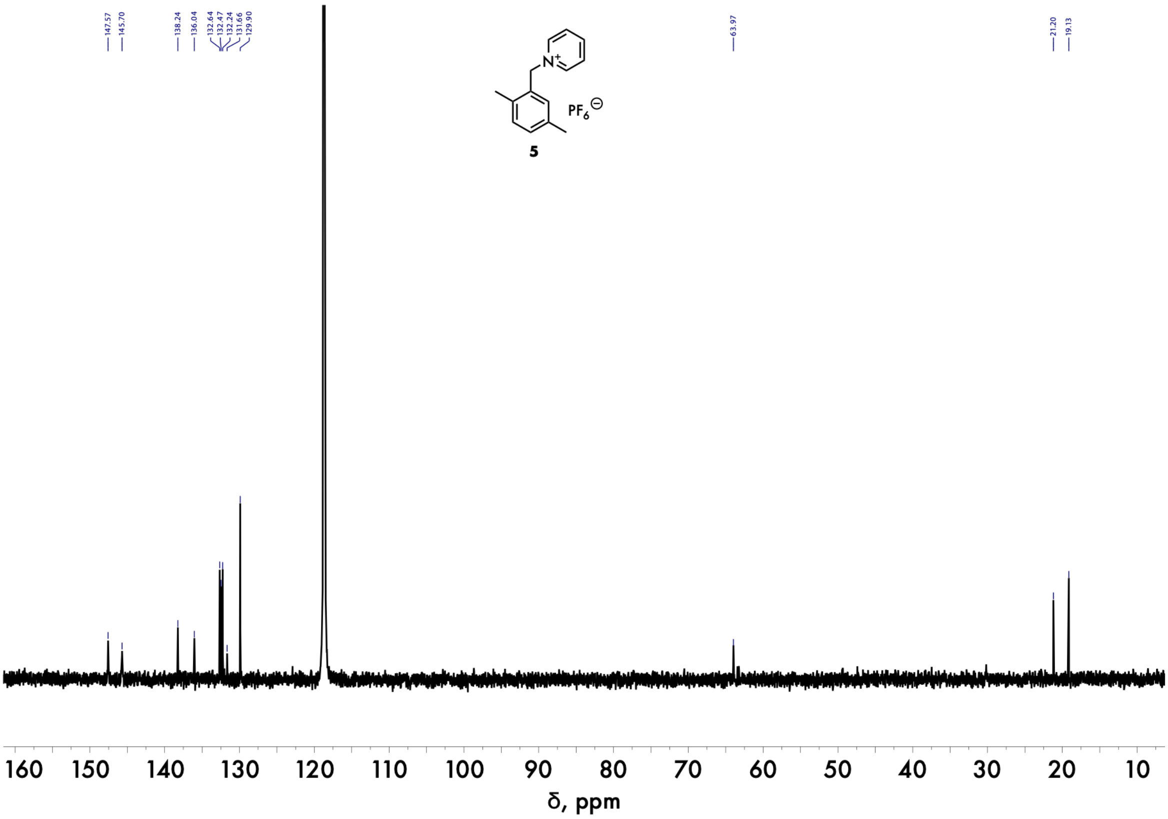

Figure S40. ^{13}C NMR (101 MHz, CD_3CN) spectrum of **5**.

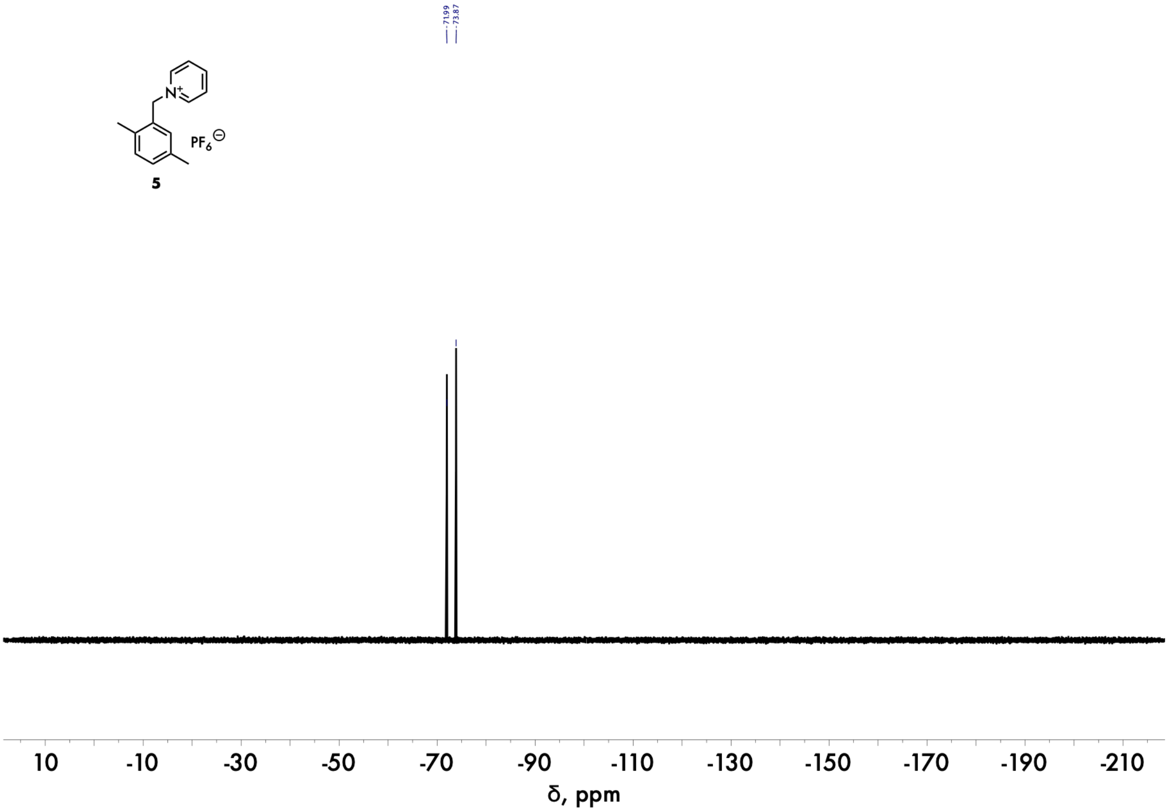

Figure S41. ^{19}F NMR (376 MHz, CD_3CN) spectrum of **5**.

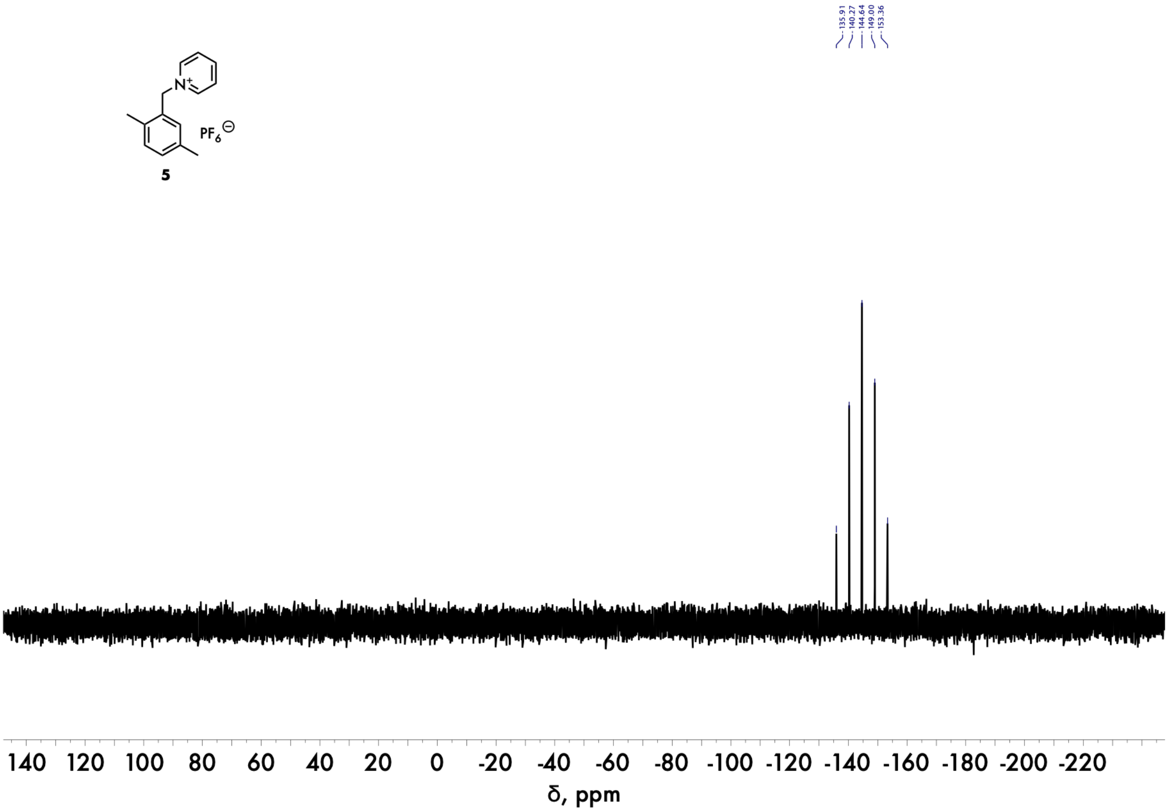

Figure S42. ^{31}P NMR (162 MHz, CD_3CN) spectrum of **5**.

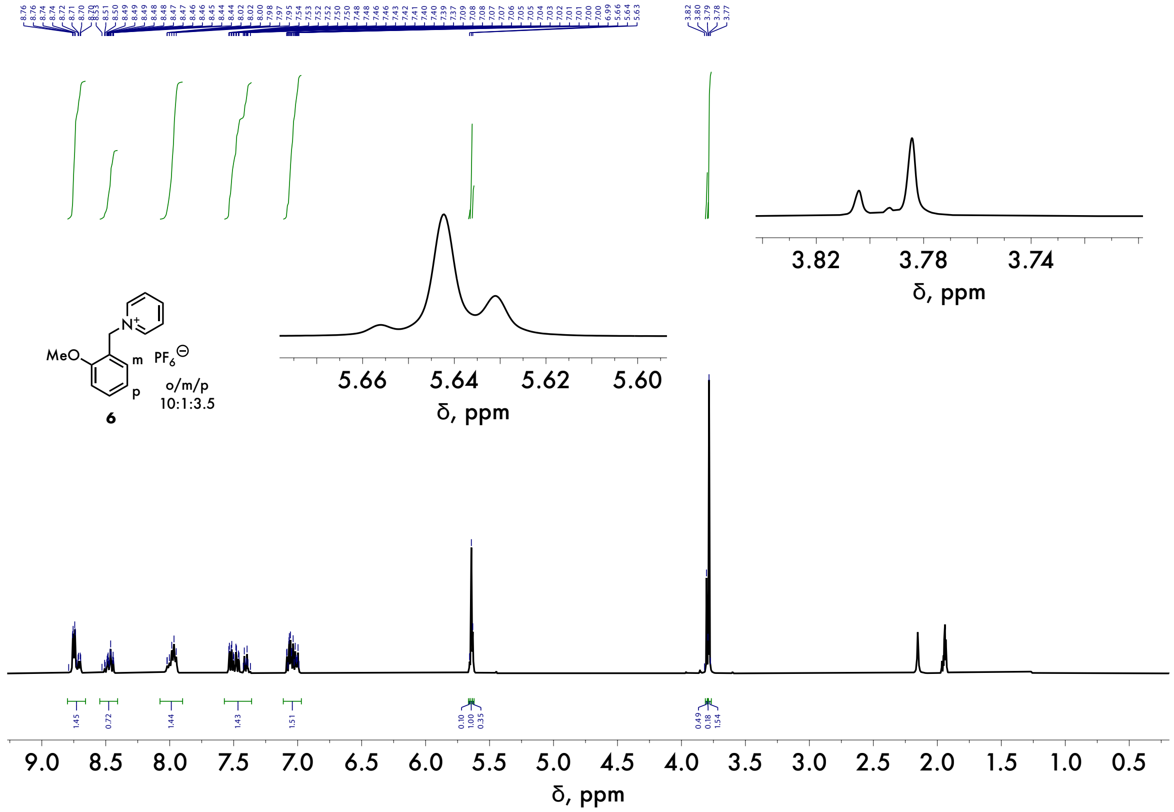

Figure S43. ^{1}H NMR (400 MHz, CD_3CN) spectrum of **6**.

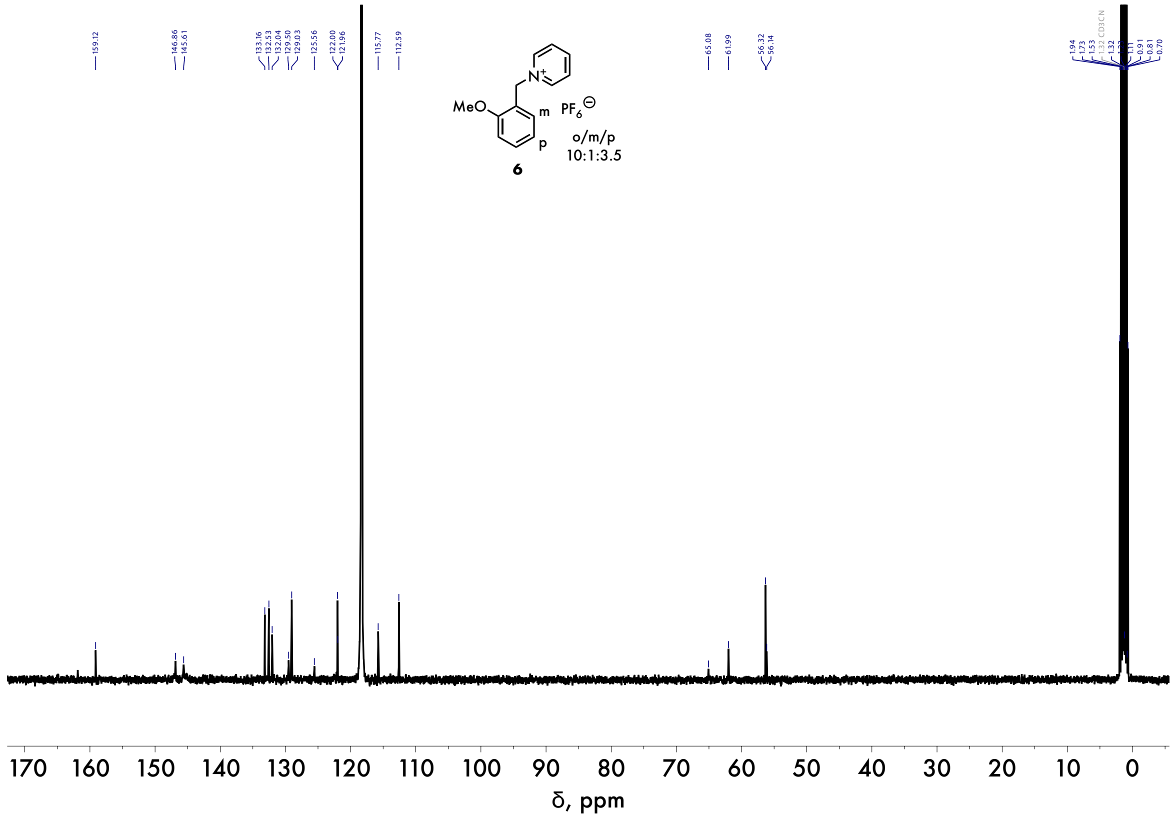

Figure S44. ^{13}C NMR (101 MHz, CD_3CN) spectrum of **6**.

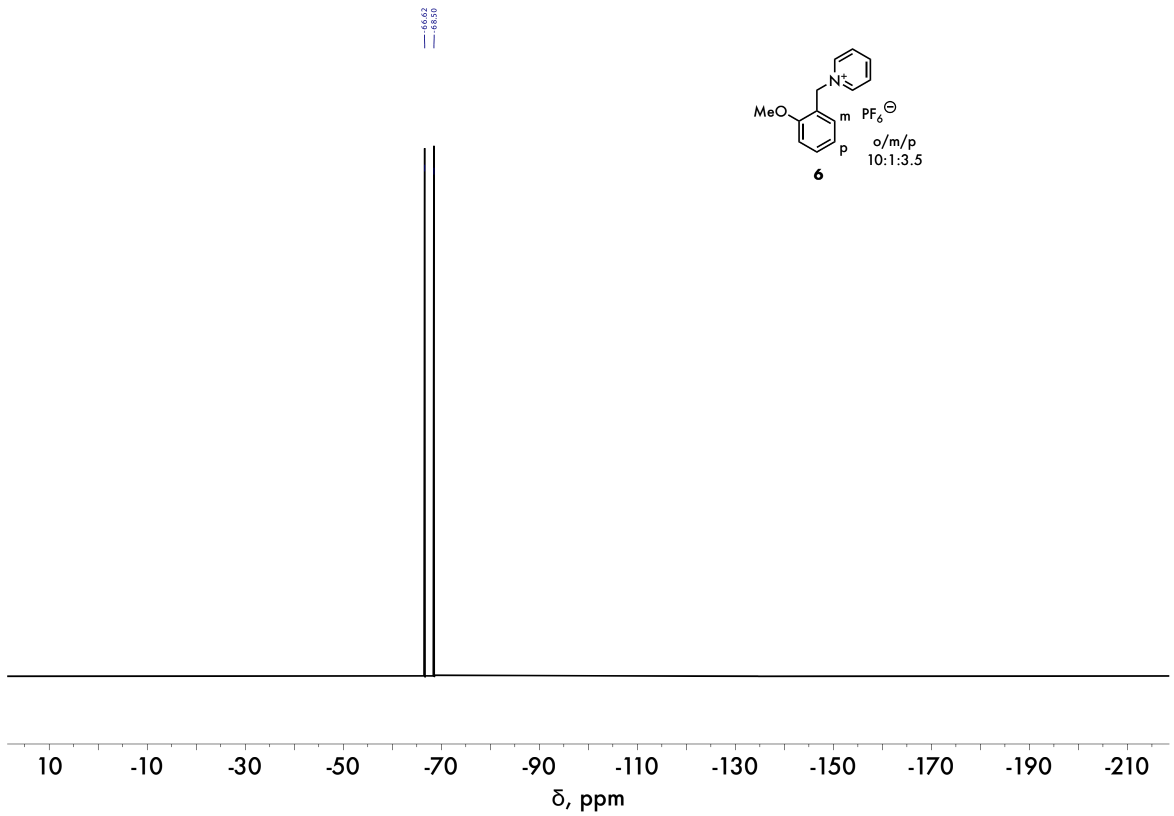

Figure S45. ^{19}F NMR (376 MHz, CD_3CN) spectrum of **6**.

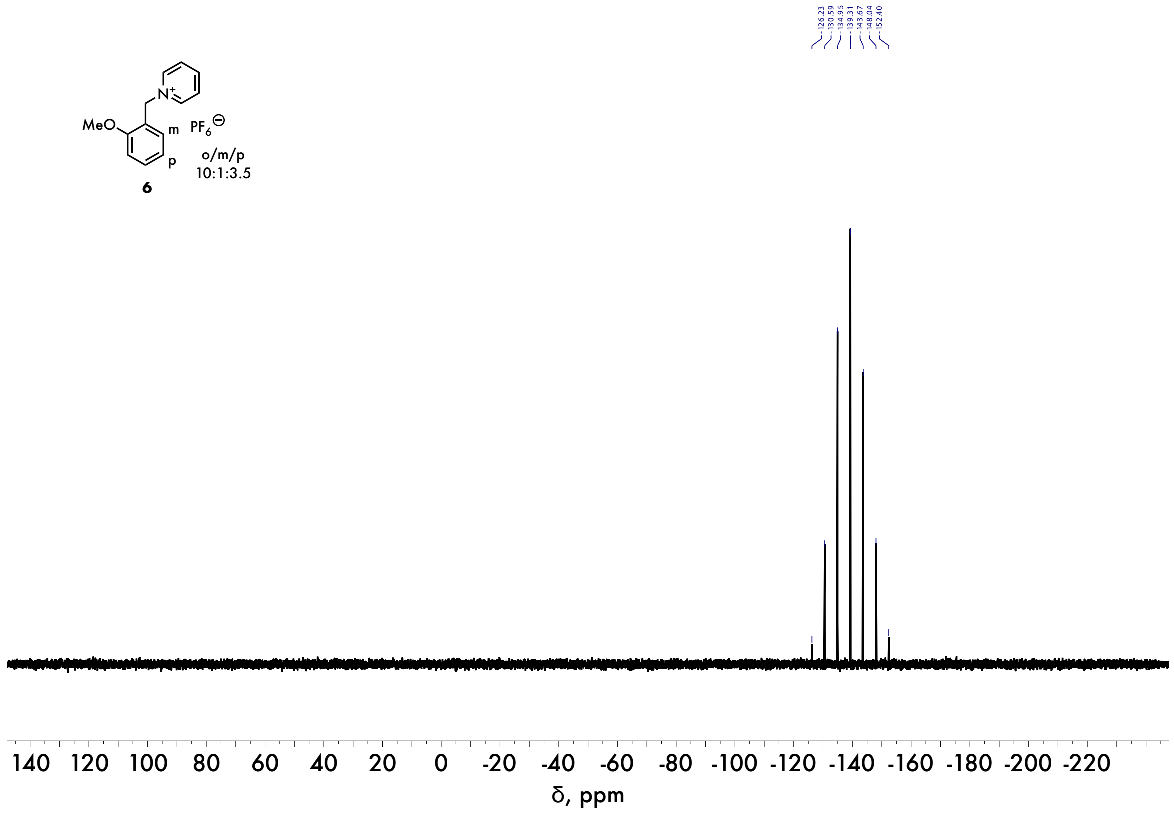

Figure S46. ^{31}P NMR (162 MHz, CD_3CN) spectrum of **6**.

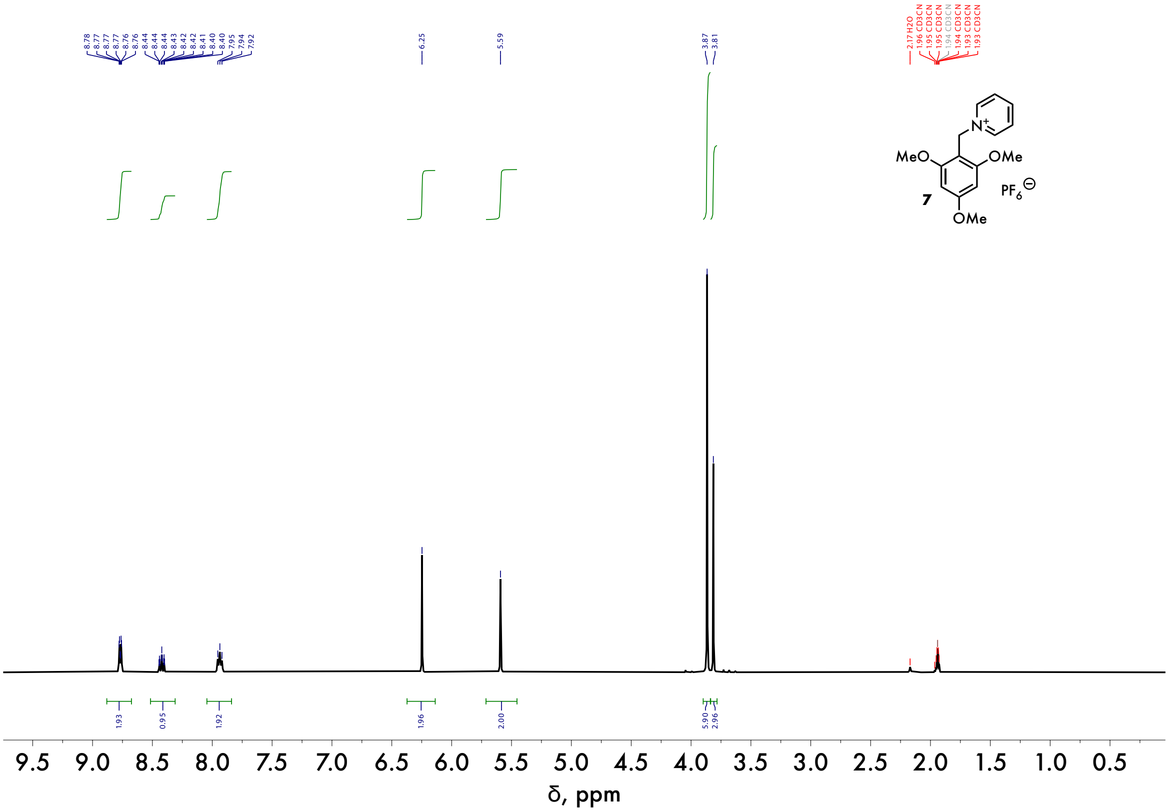

Figure S47. ^{1}H NMR (400 MHz, CD_3CN) spectrum of **7**.

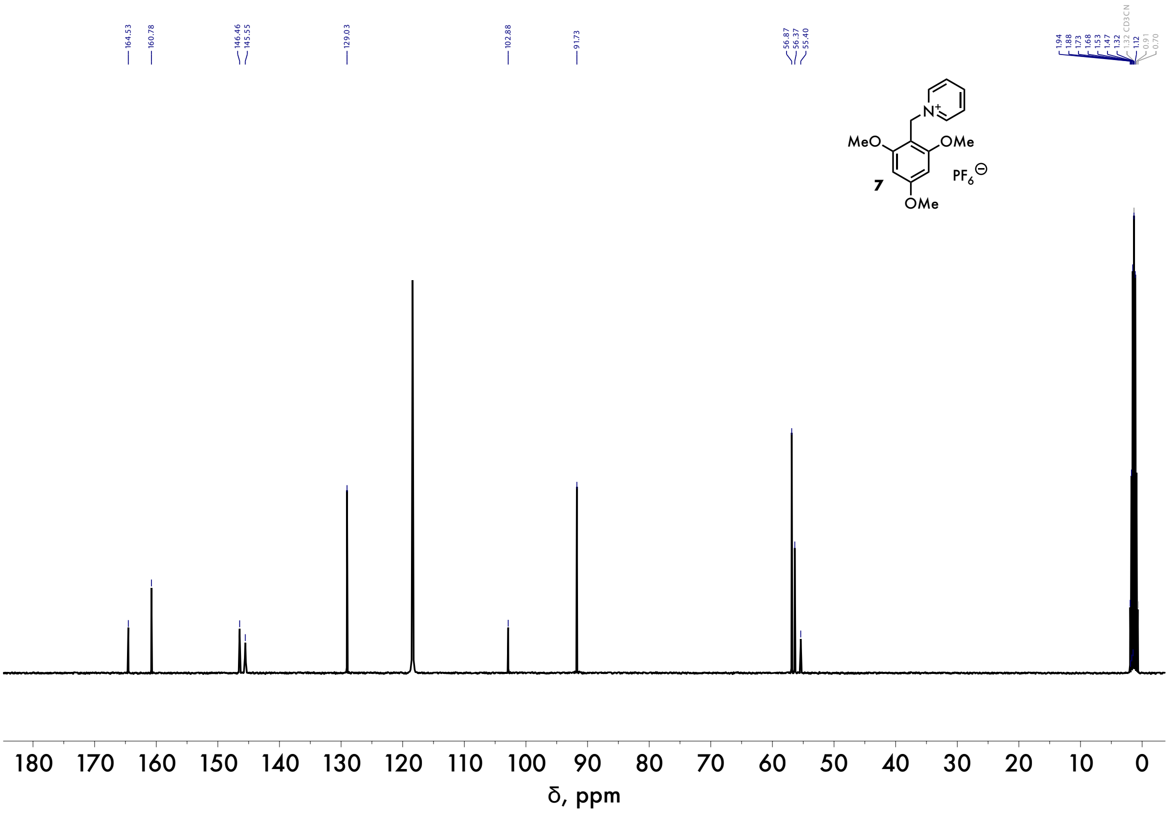

Figure S48. ^{13}C NMR (101 MHz, CD_3CN) spectrum of **7**.

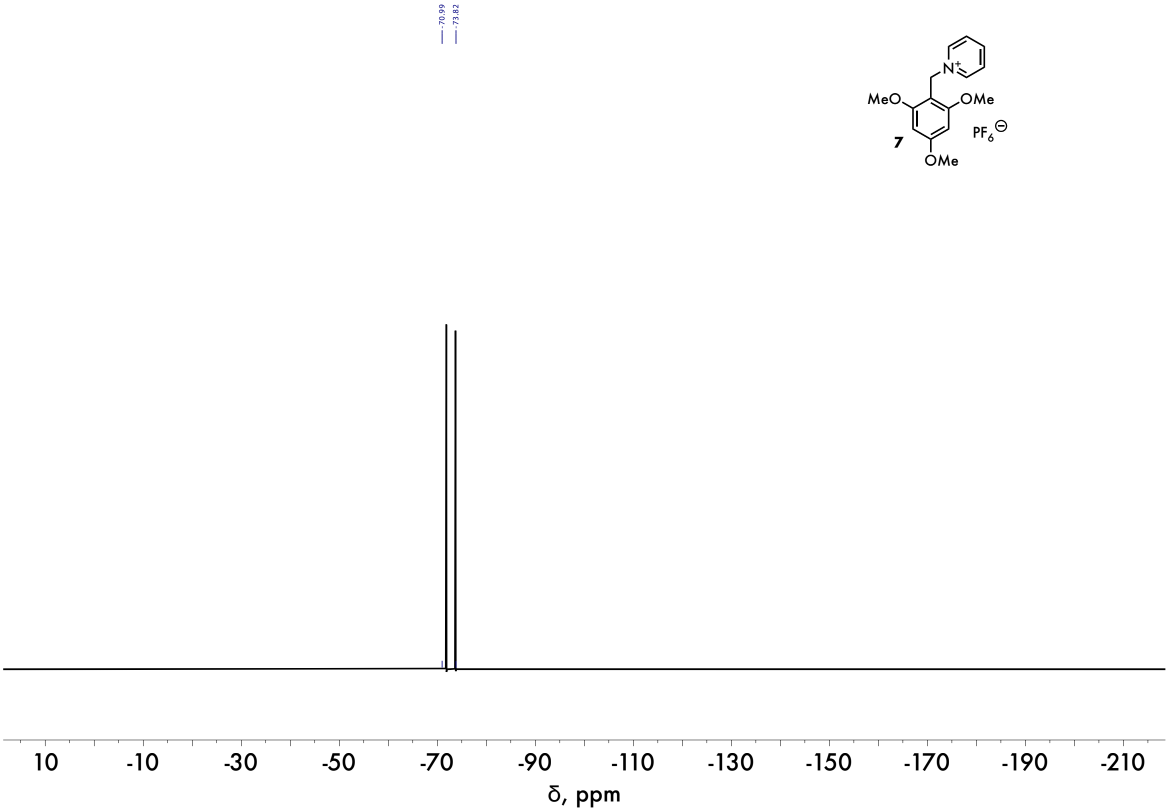

Figure S49. ^{19}F NMR (376 MHz, CD_3CN) spectrum of **7**.

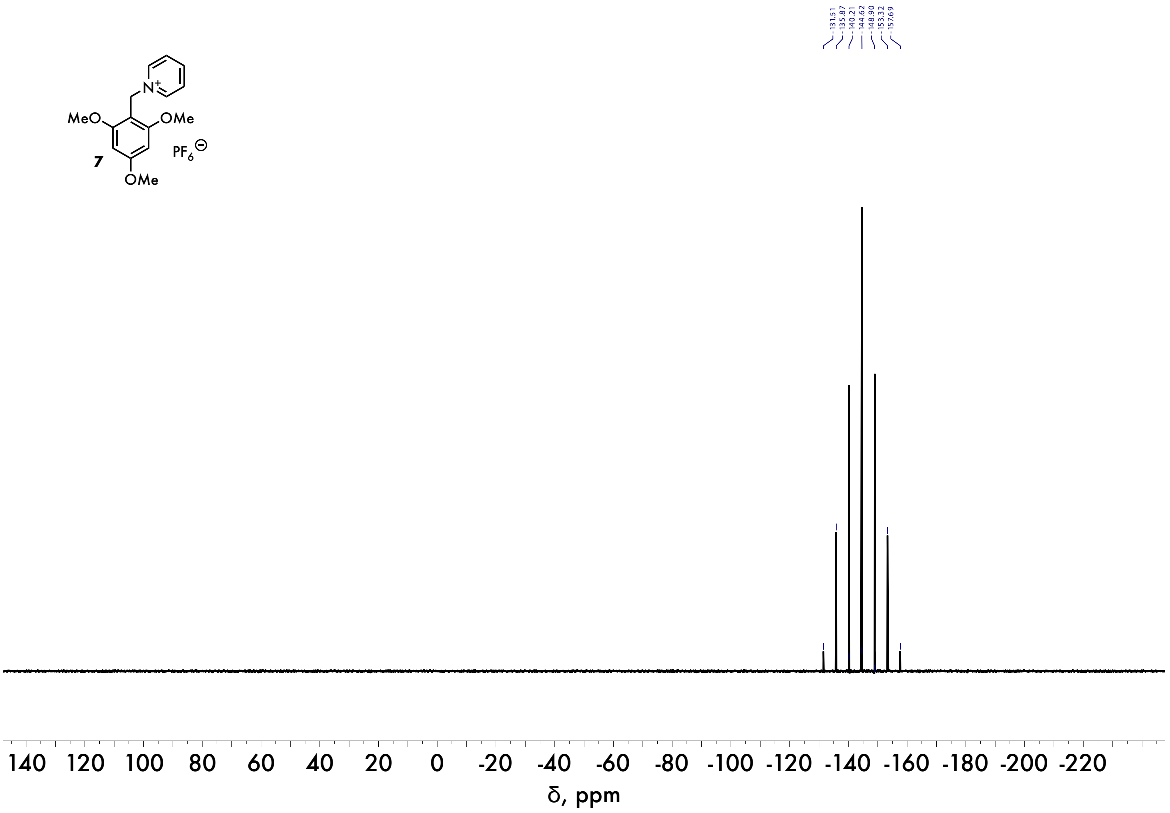

Figure S50. ^{31}P NMR (162 MHz, CD_3CN) spectrum of **7**.

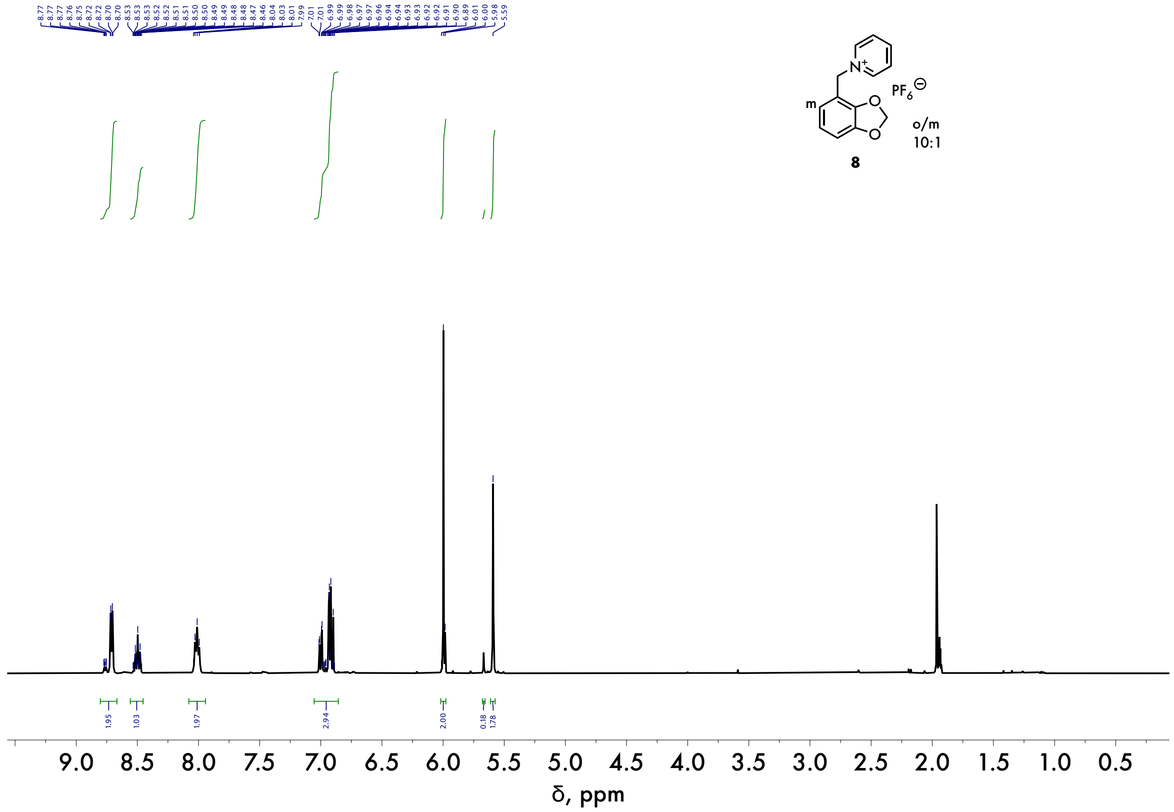

Figure S51. ^{1}H NMR (400 MHz, CD_3CN) spectrum of **8**.

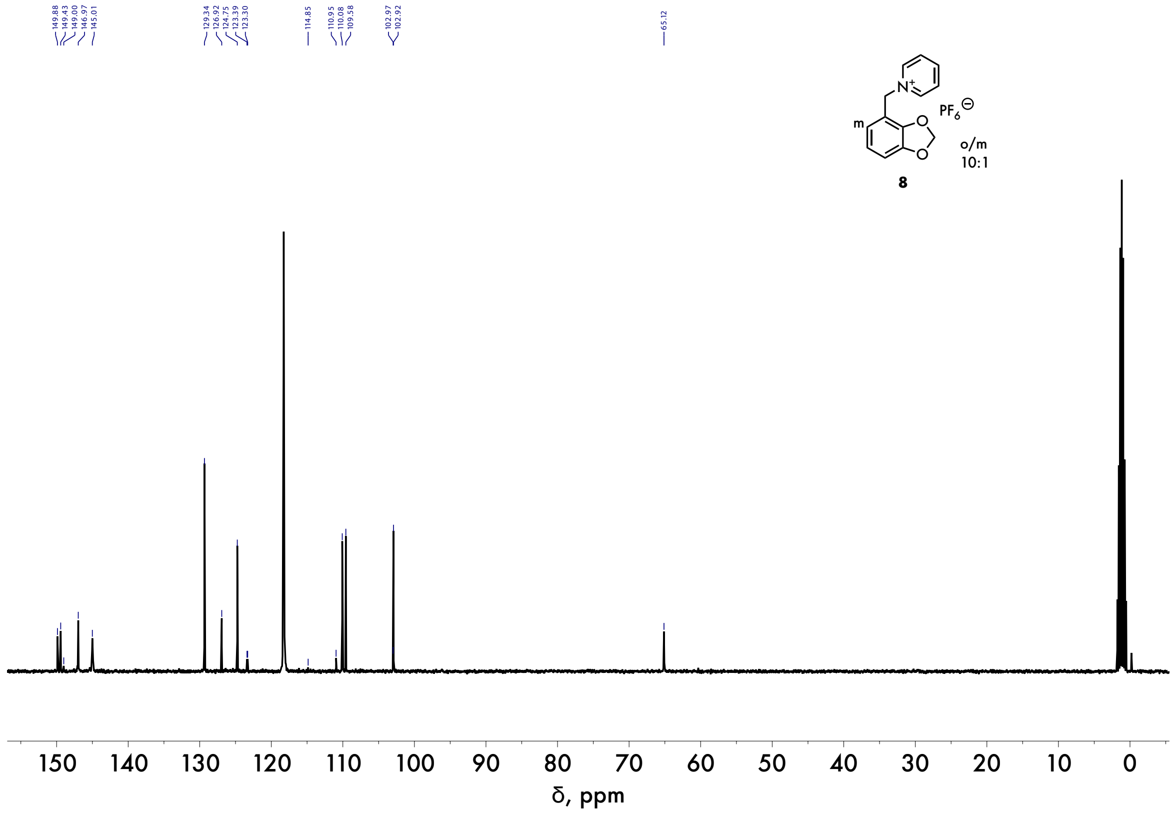

Figure S52. ^{13}C NMR (101 MHz, CD_3CN) spectrum of **8**.

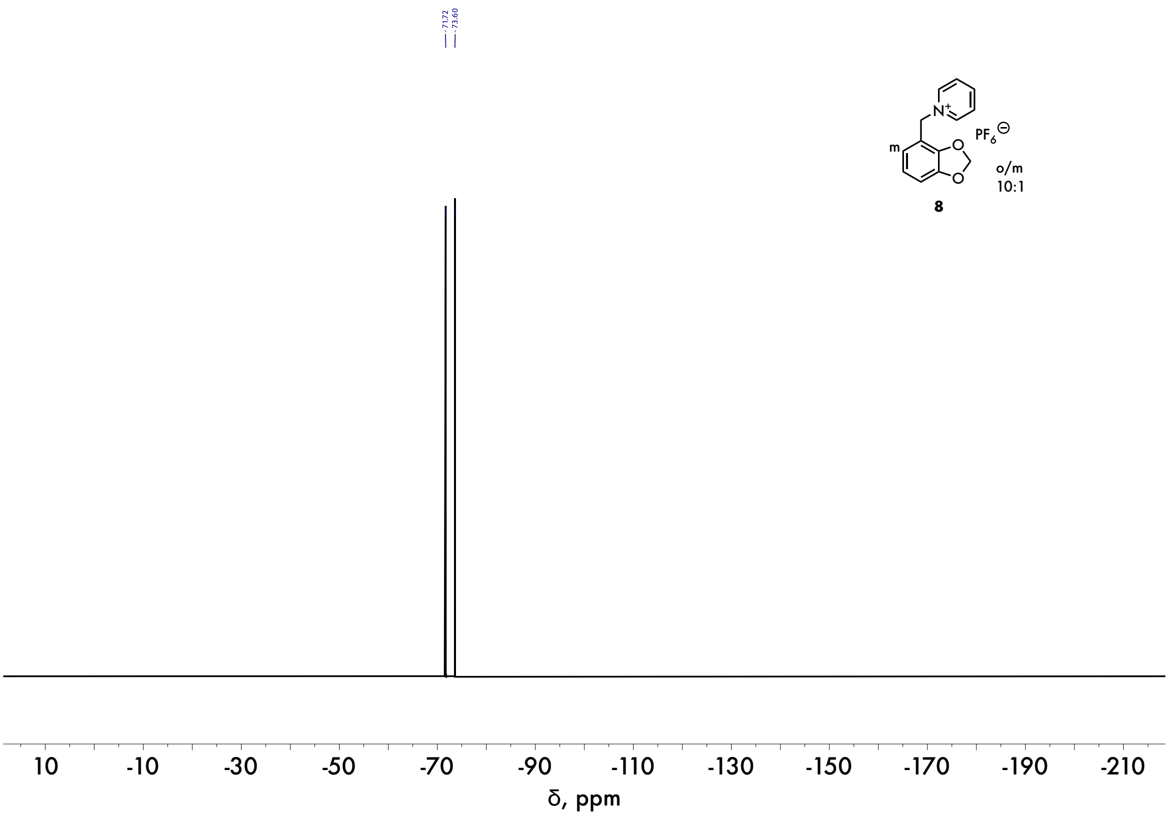

Figure S53. ^{19}F NMR (376 MHz, CD_3CN) spectrum of **8**.

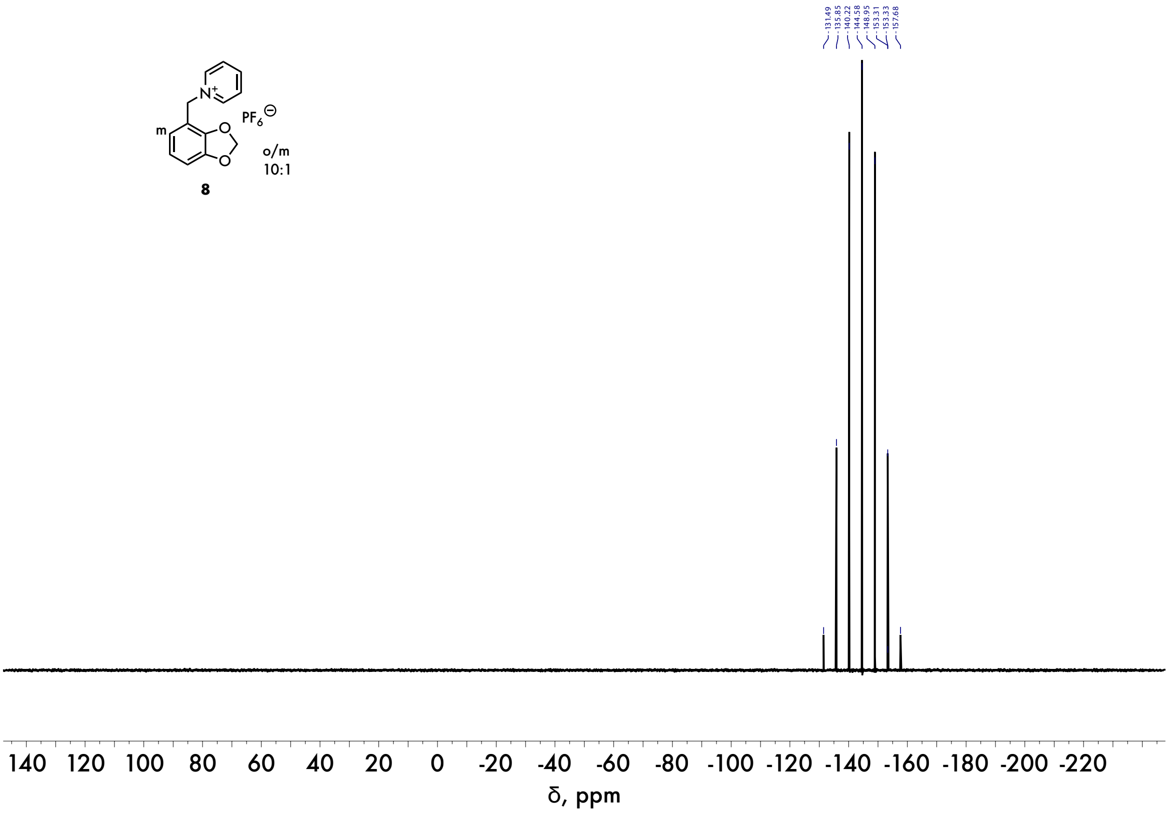

Figure S54. ^{31}P NMR (162 MHz, CD_3CN) spectrum of **8**.

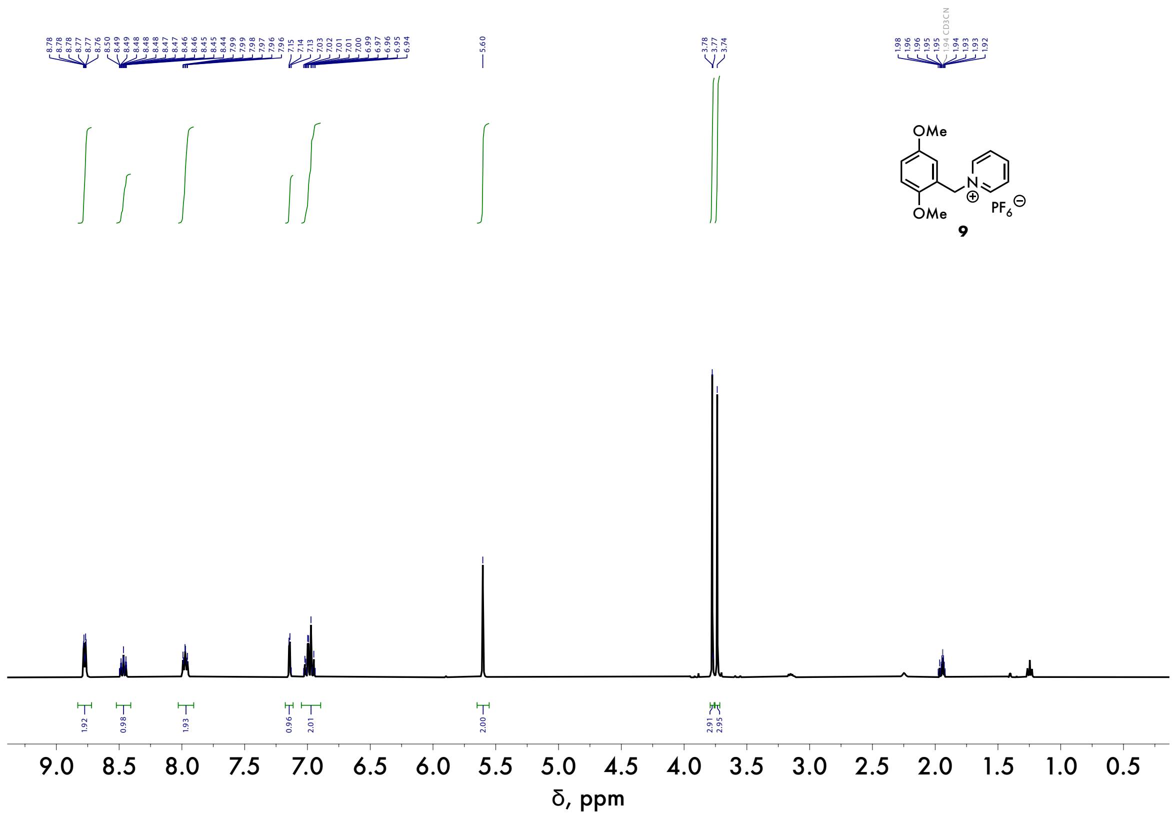

Figure S55. ^{1}H NMR (400 MHz, CD_3CN) spectrum of **9**.

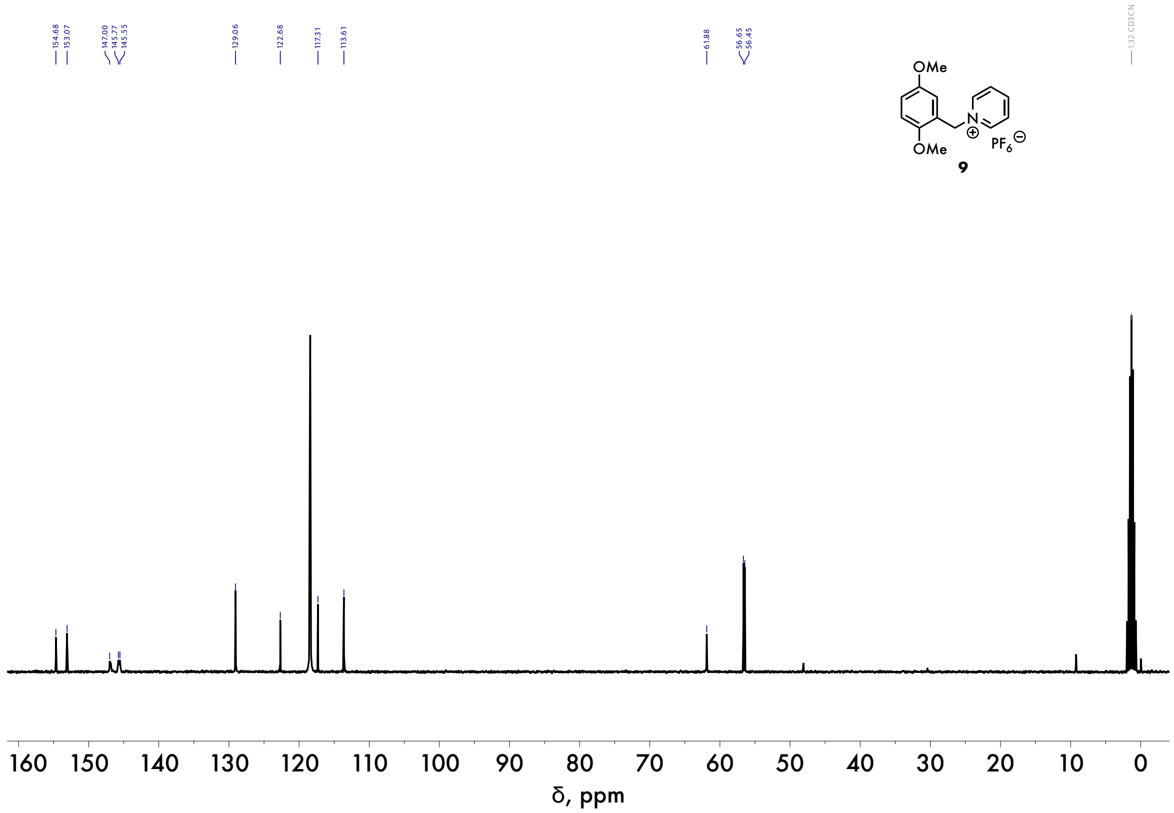

Figure S56. ^{13}C NMR (101 MHz, CD_3CN) spectrum of **9**.

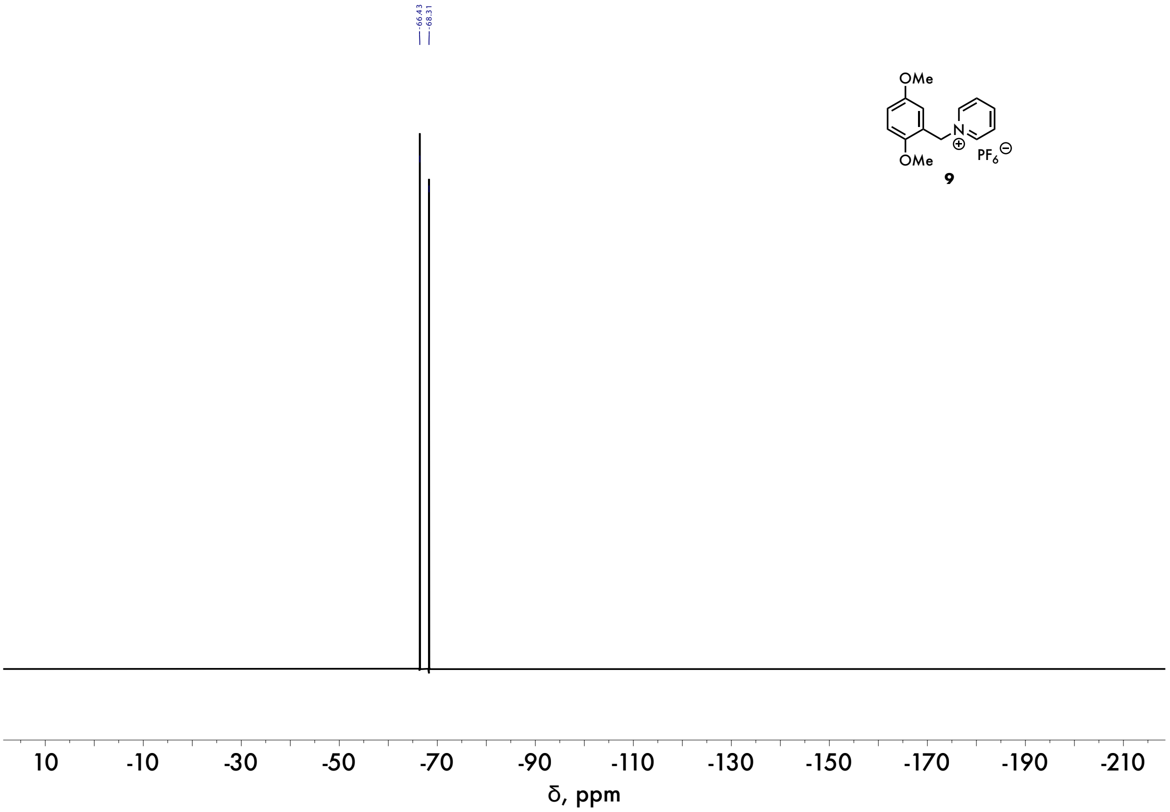

Figure S57. ^{19}F NMR (376 MHz, CD_3CN) spectrum of **9**.

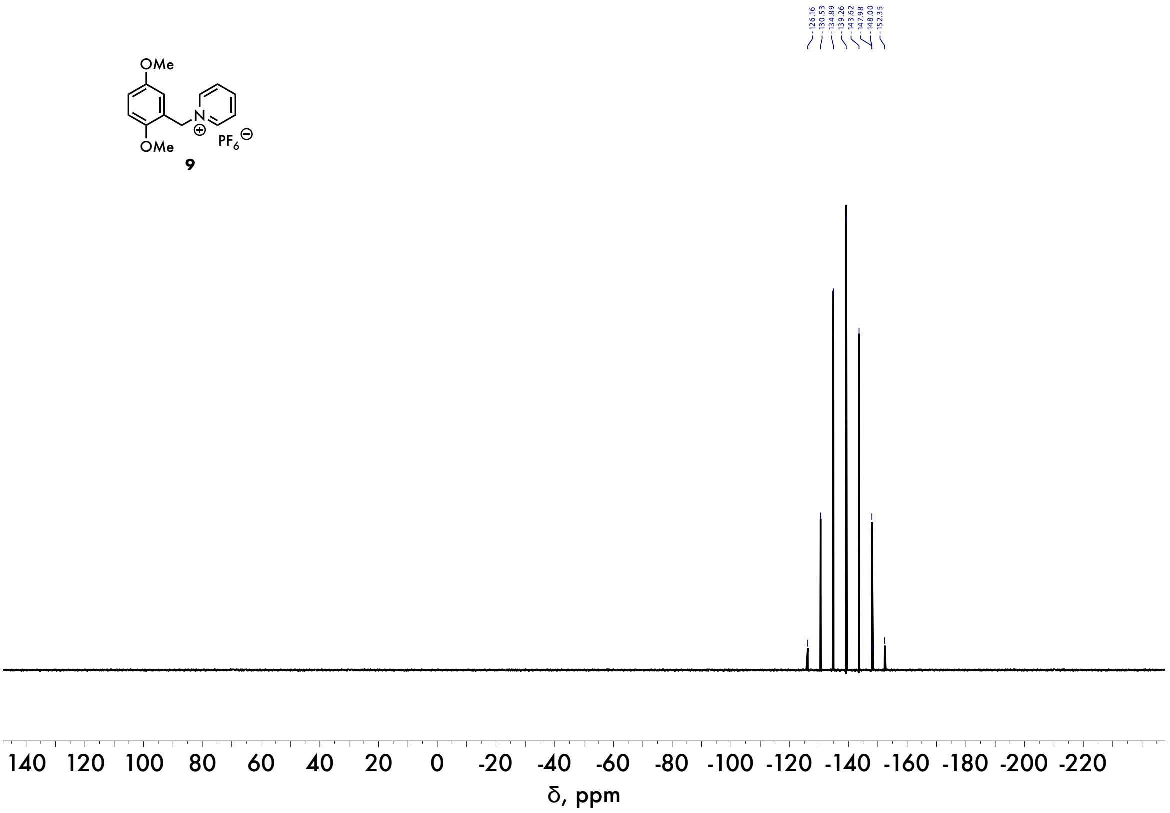

Figure S58. ^{31}P NMR (162 MHz, CD_3CN) spectrum of **9**.

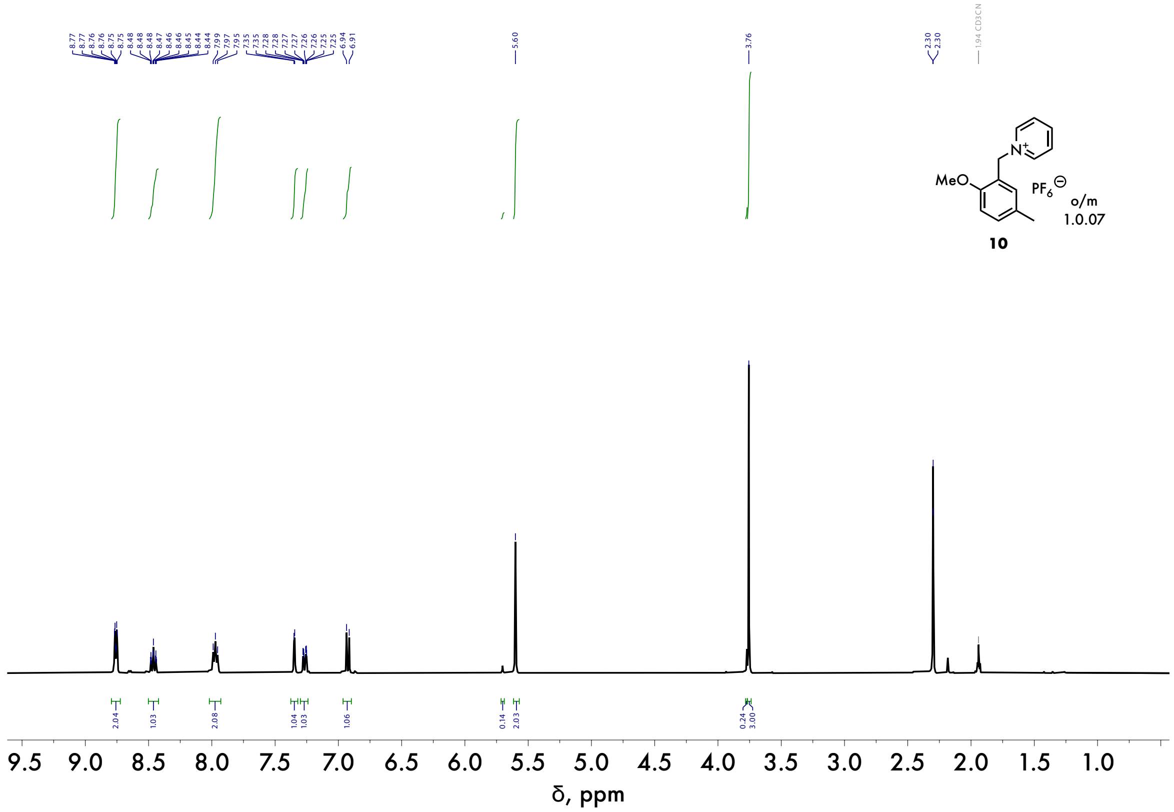

Figure S59. ^{1}H NMR (400 MHz, CD_3CN) spectrum of **10**.

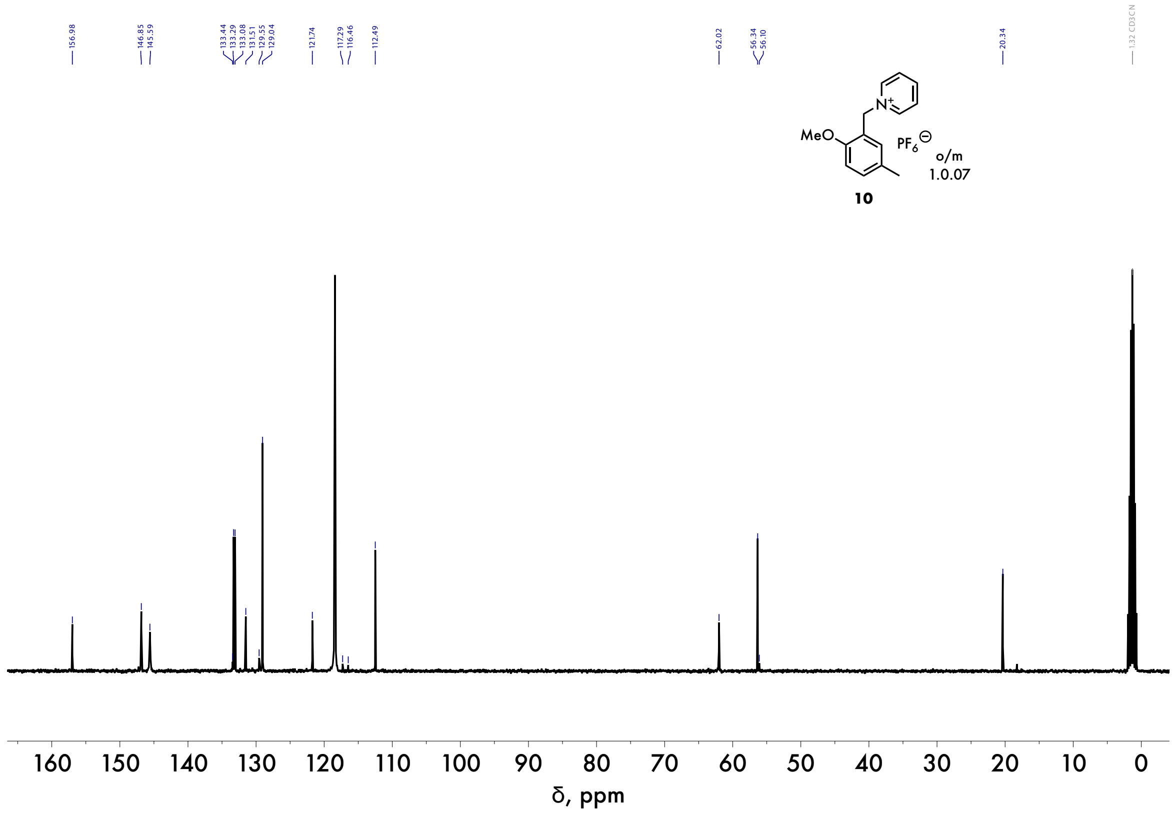

Figure S60. ^{13}C NMR (101 MHz, CD_3CN) spectrum of **10**.

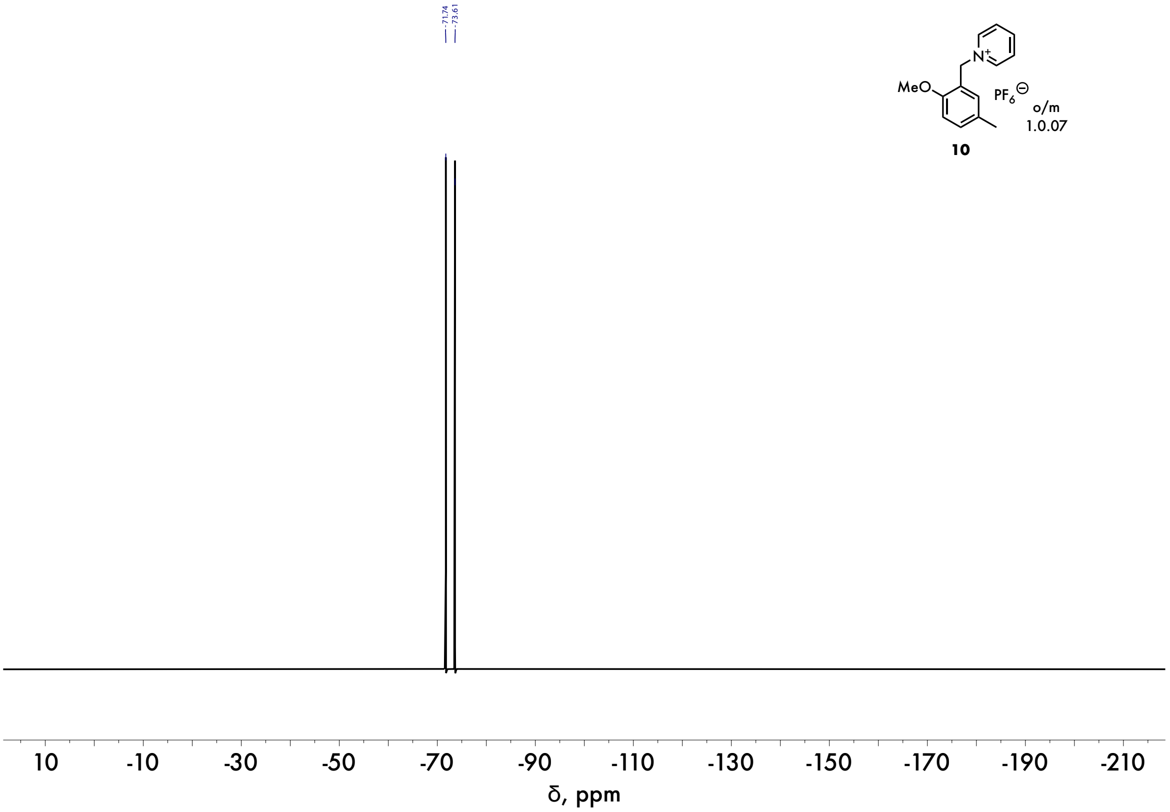

Figure S61. ^{19}F NMR (376 MHz, CD_3CN) spectrum of **10**.

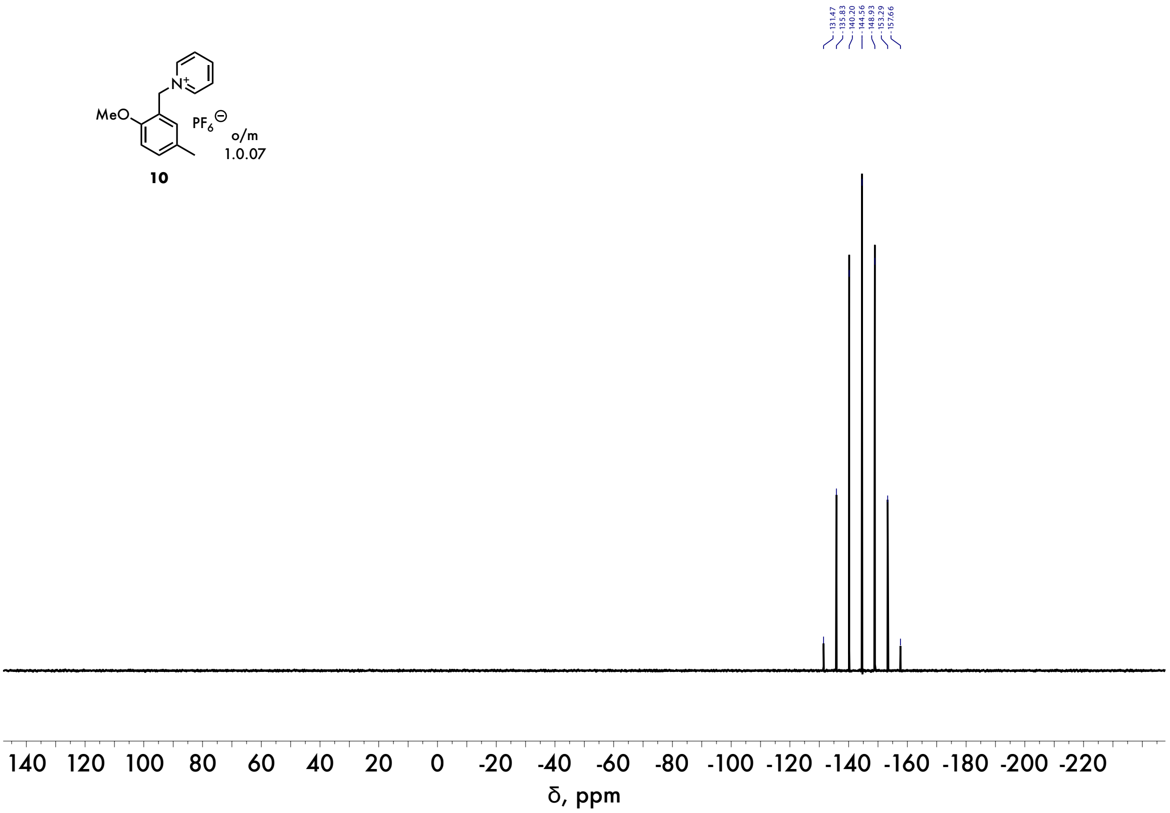

Figure S62. ^{31}P NMR (162 MHz, CD_3CN) spectrum of **10**.

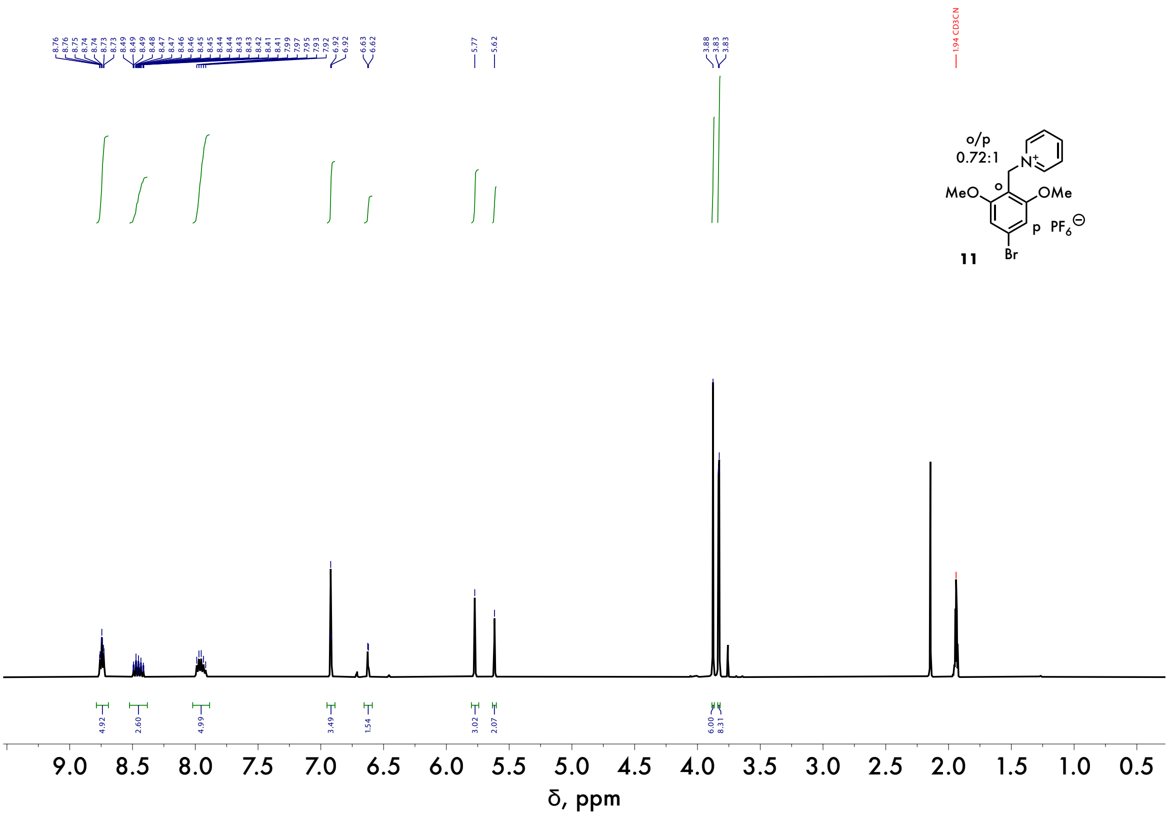

Figure S63. ^{1}H NMR (400 MHz, CD_3CN) spectrum of **11**.

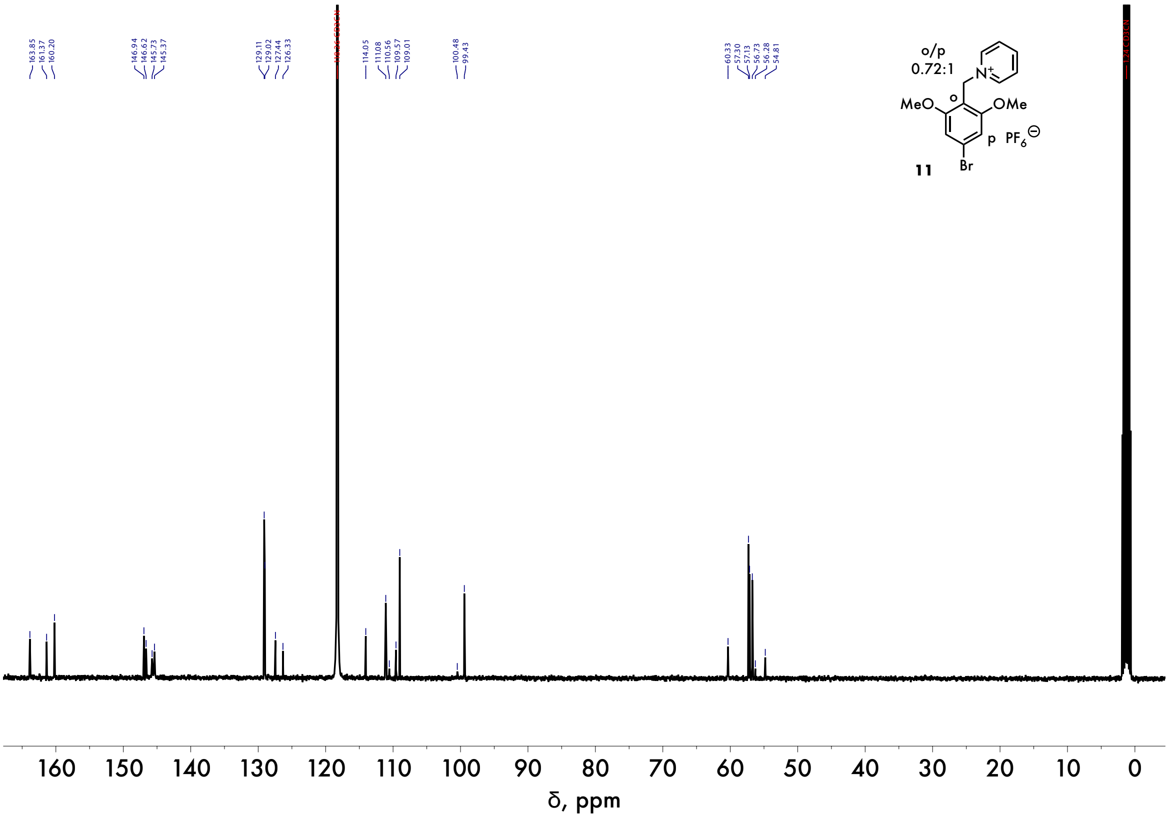

Figure S64. ^{13}C NMR (101 MHz, CD_3CN) spectrum of **11**.

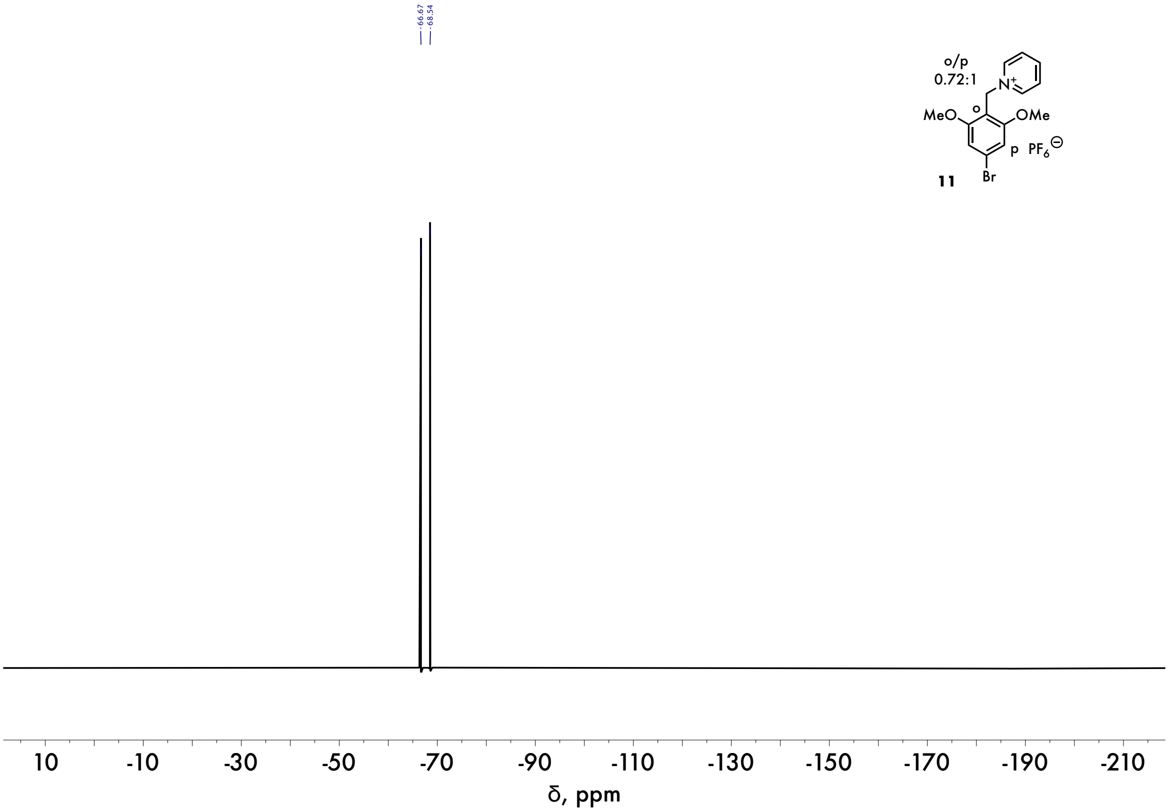

Figure S65. ^{19}F NMR (376 MHz, CD_3CN) spectrum of **11**.

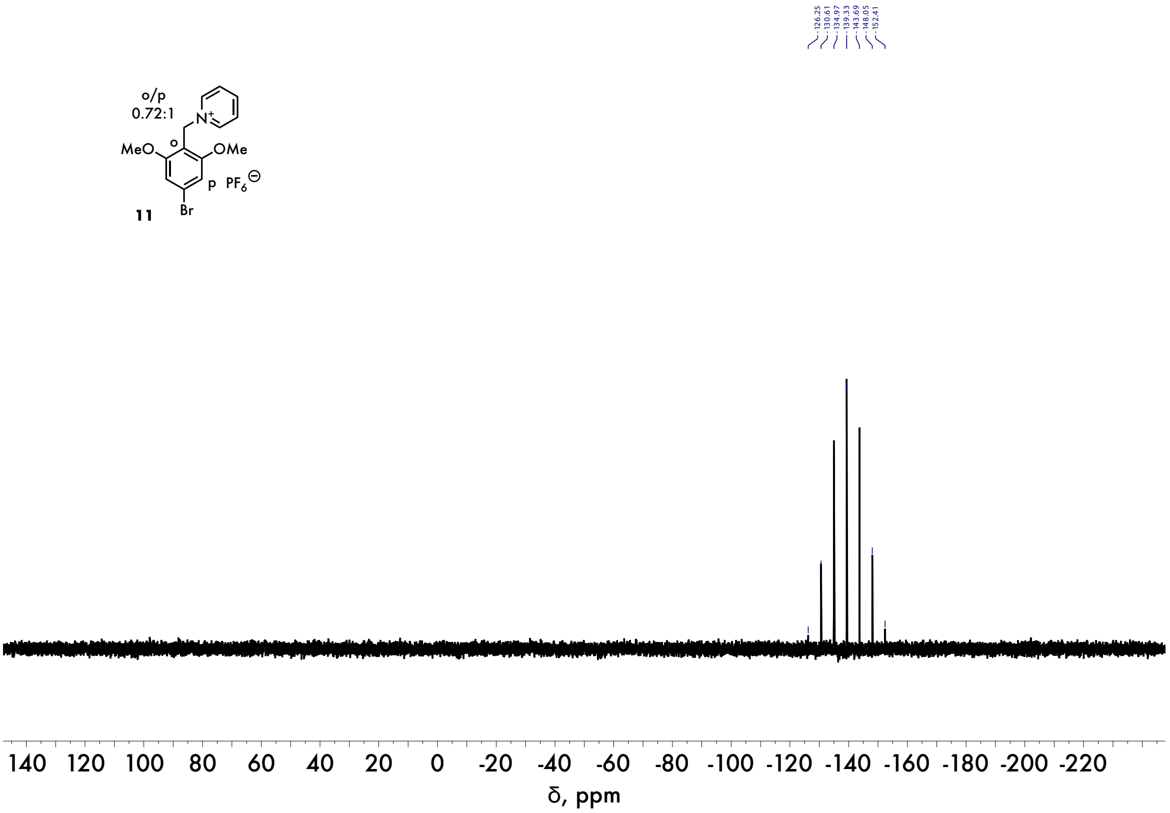

Figure S66. ^{31}P NMR (162 MHz, CD_3CN) spectrum of **11**.

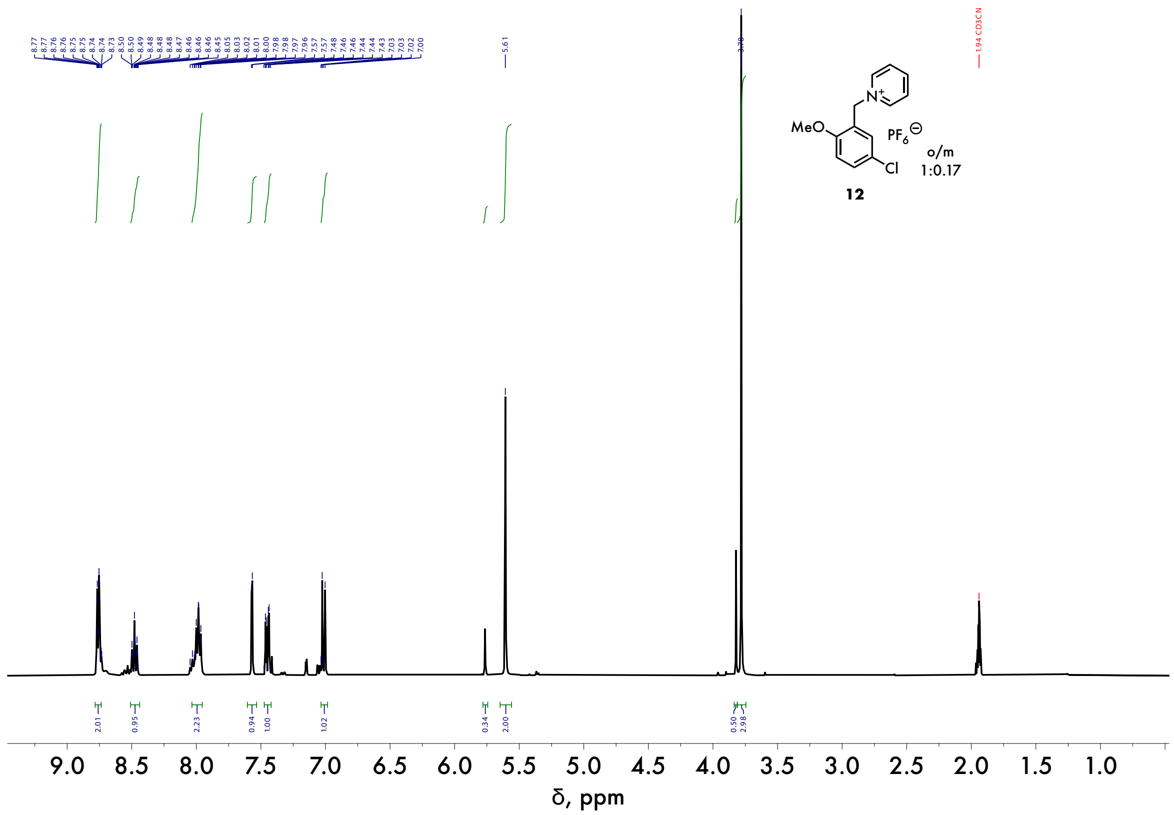

Figure S67. ^{1}H NMR (400 MHz, CD_3CN) spectrum of **12**.

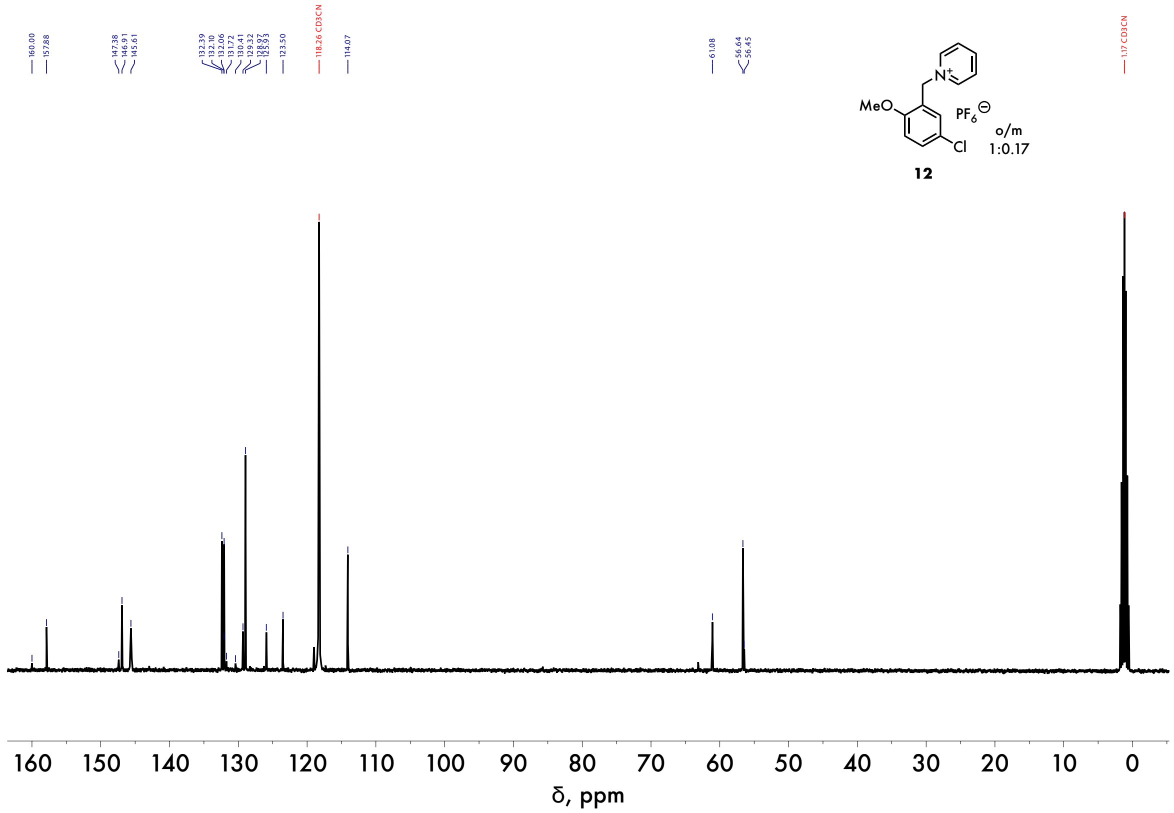

Figure S68. ^{13}C NMR (101 MHz, CD_3CN) spectrum of **12**.

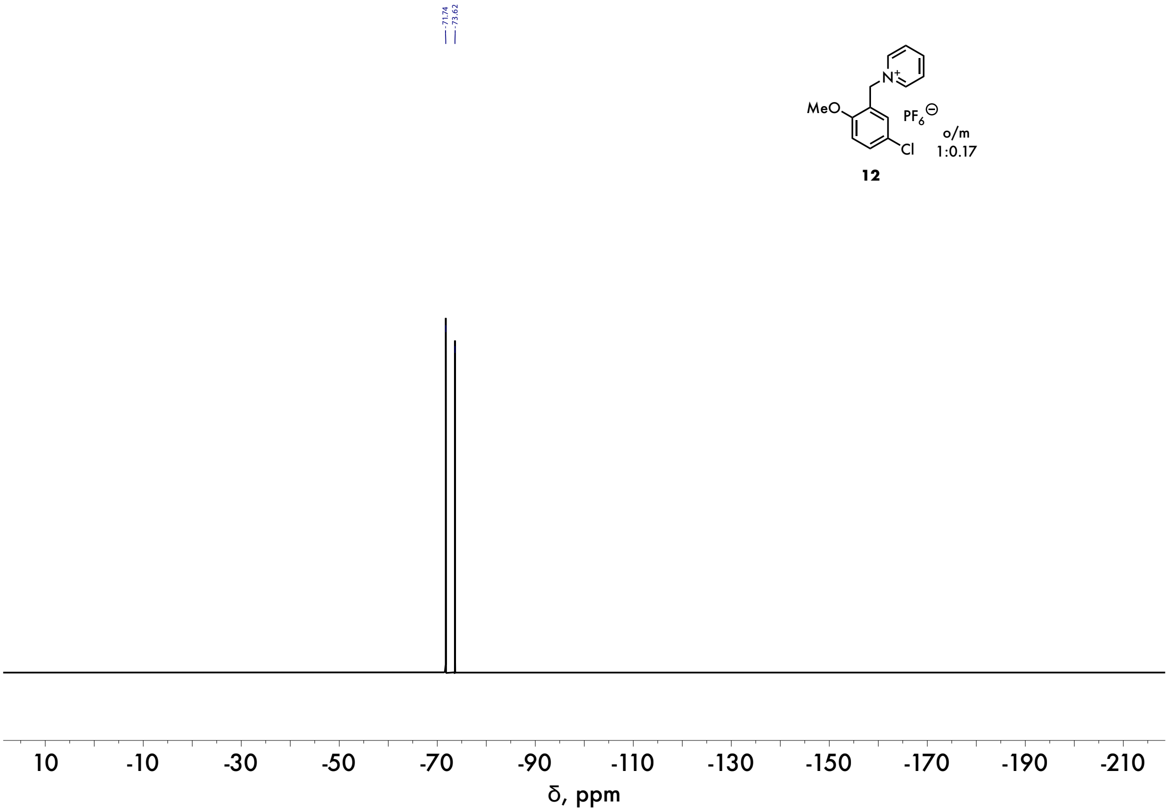

Figure S69. ^{19}F NMR (376 MHz, CD_3CN) spectrum of **12**.

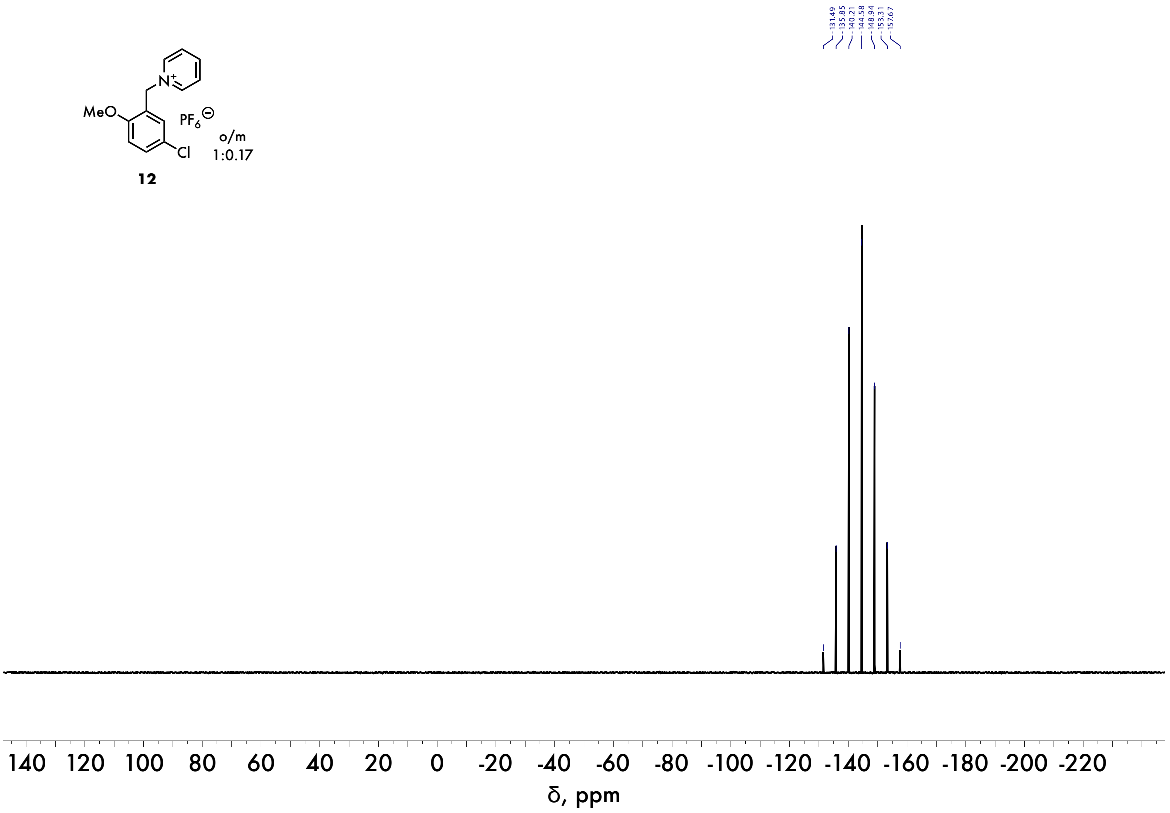

Figure S70. ^{31}P NMR (162 MHz, CD_3CN) spectrum of **12**.

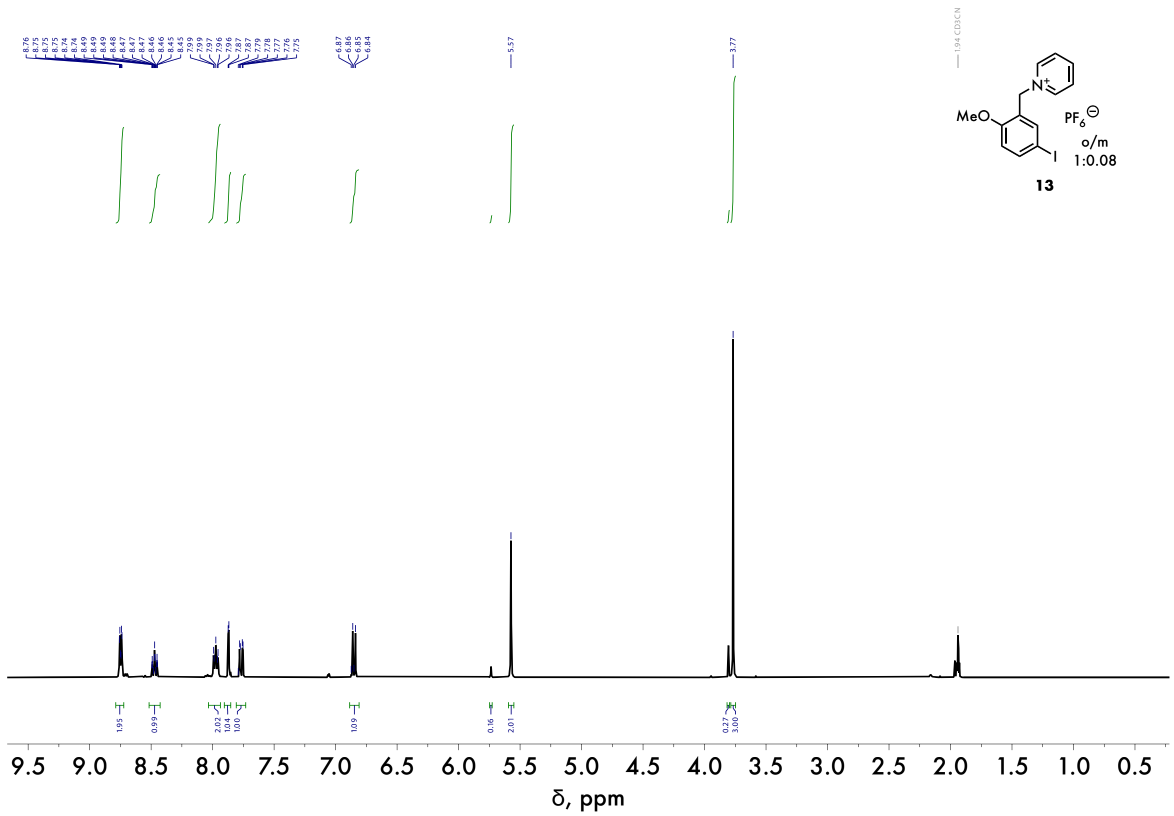

Figure S71. ^{1}H NMR (400 MHz, CD_3CN) spectrum of **13**.

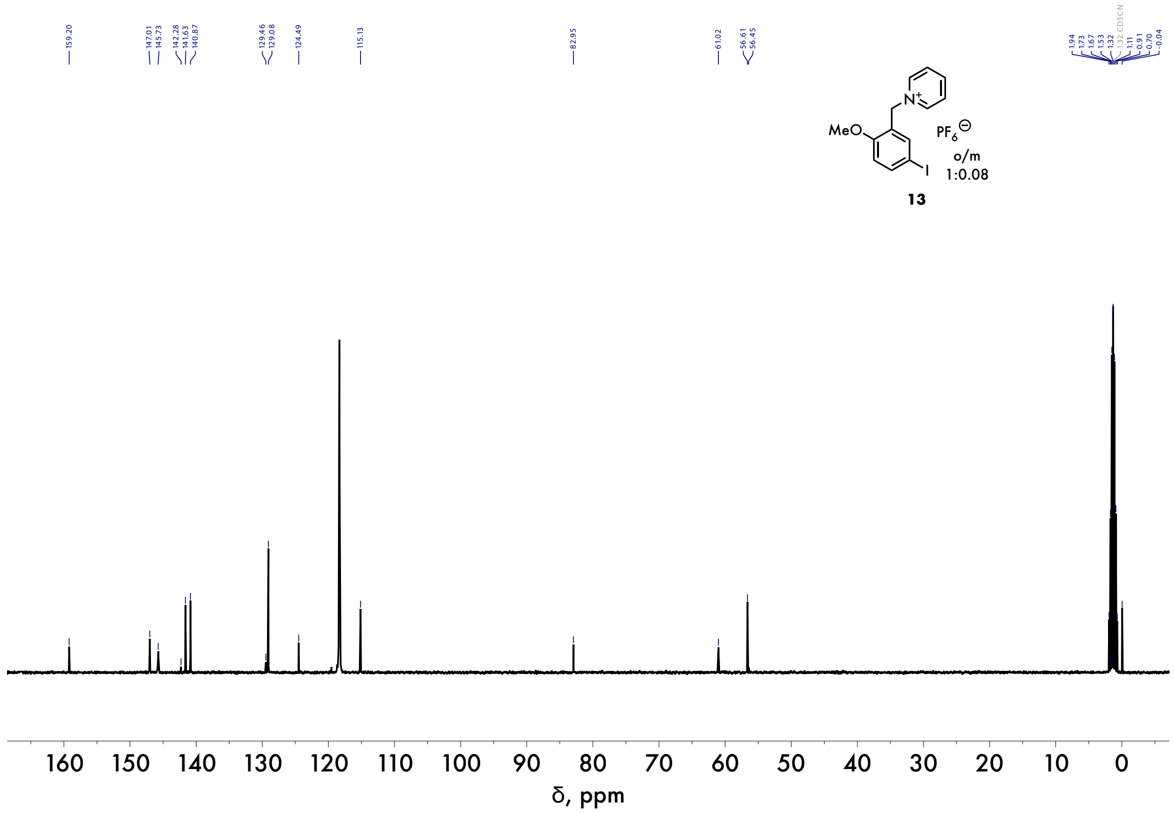

Figure S72. ^{13}C NMR (101 MHz, CD_3CN) spectrum of **13**.

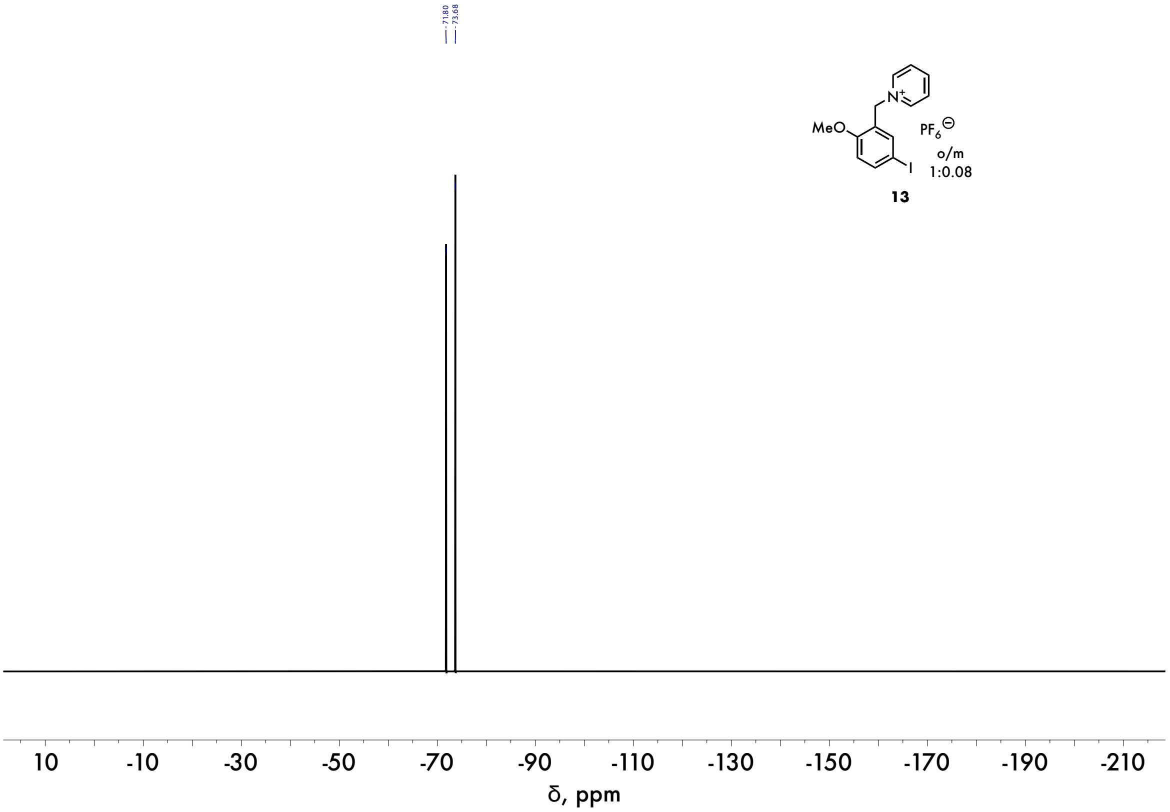

Figure S73. ^{19}F NMR (376 MHz, CD_3CN) spectrum of **13**.

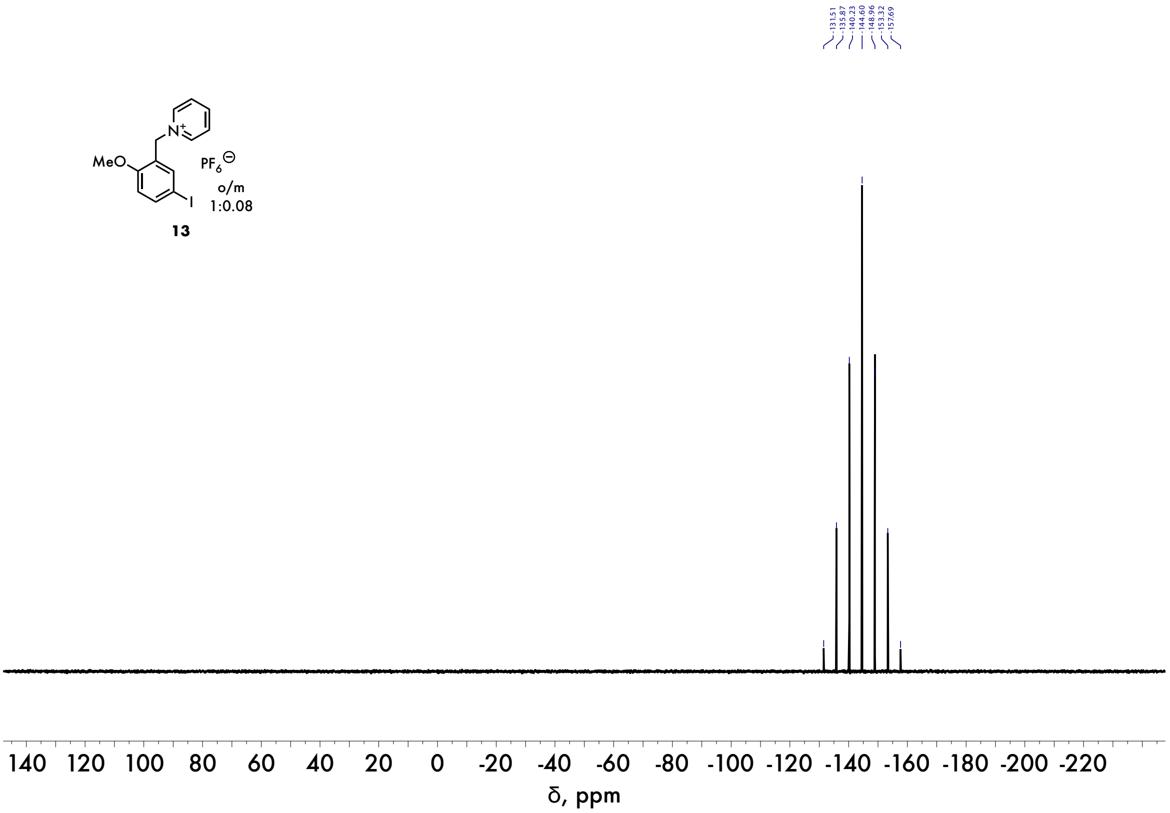

Figure S74. ^{31}P NMR (162 MHz, CD_3CN) spectrum of **13**.

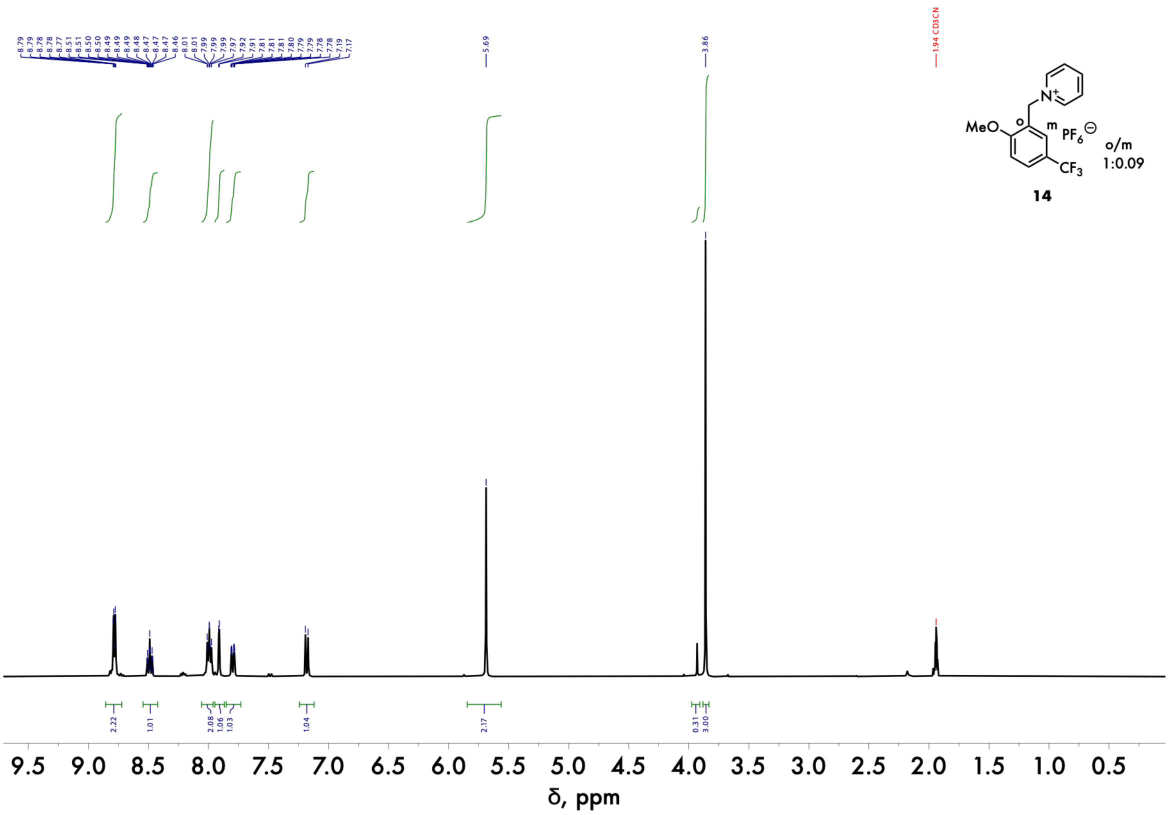

Figure S75. ^{1}H NMR (400 MHz, CD_3CN) spectrum of **14**.

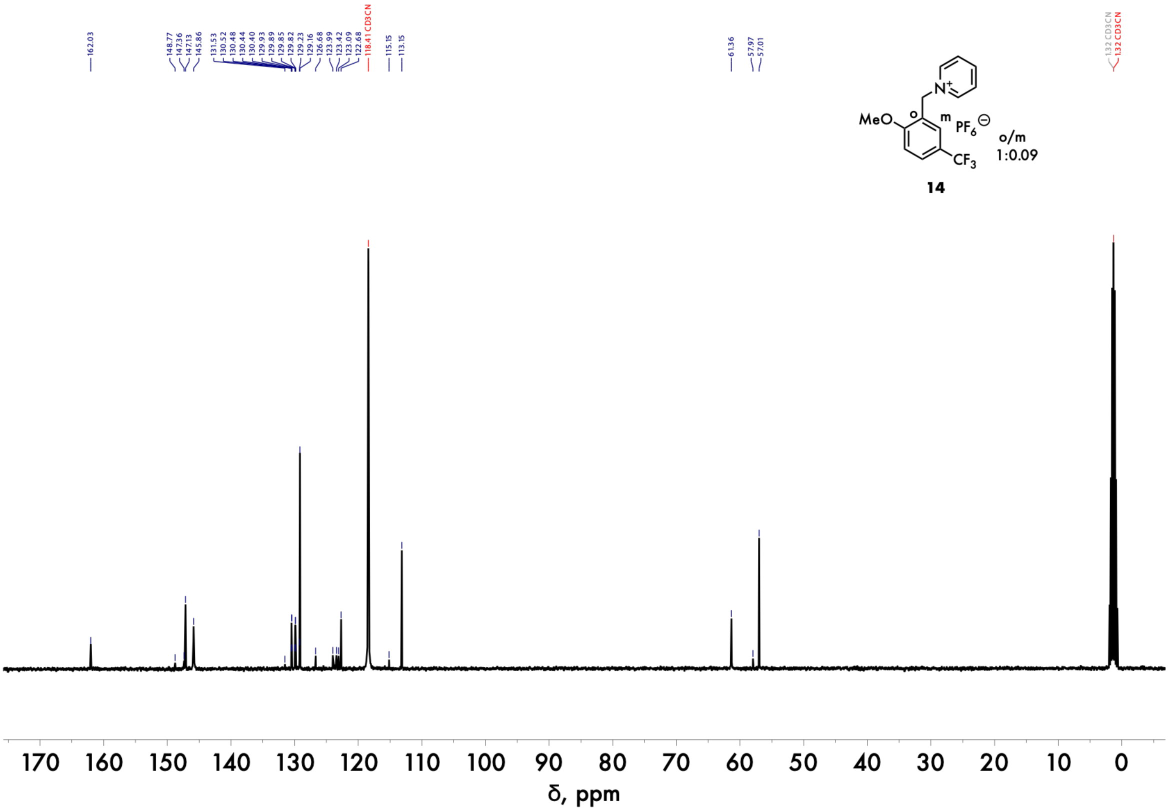

Figure S76. ^{13}C NMR (101 MHz, CD_3CN) spectrum of **14**.

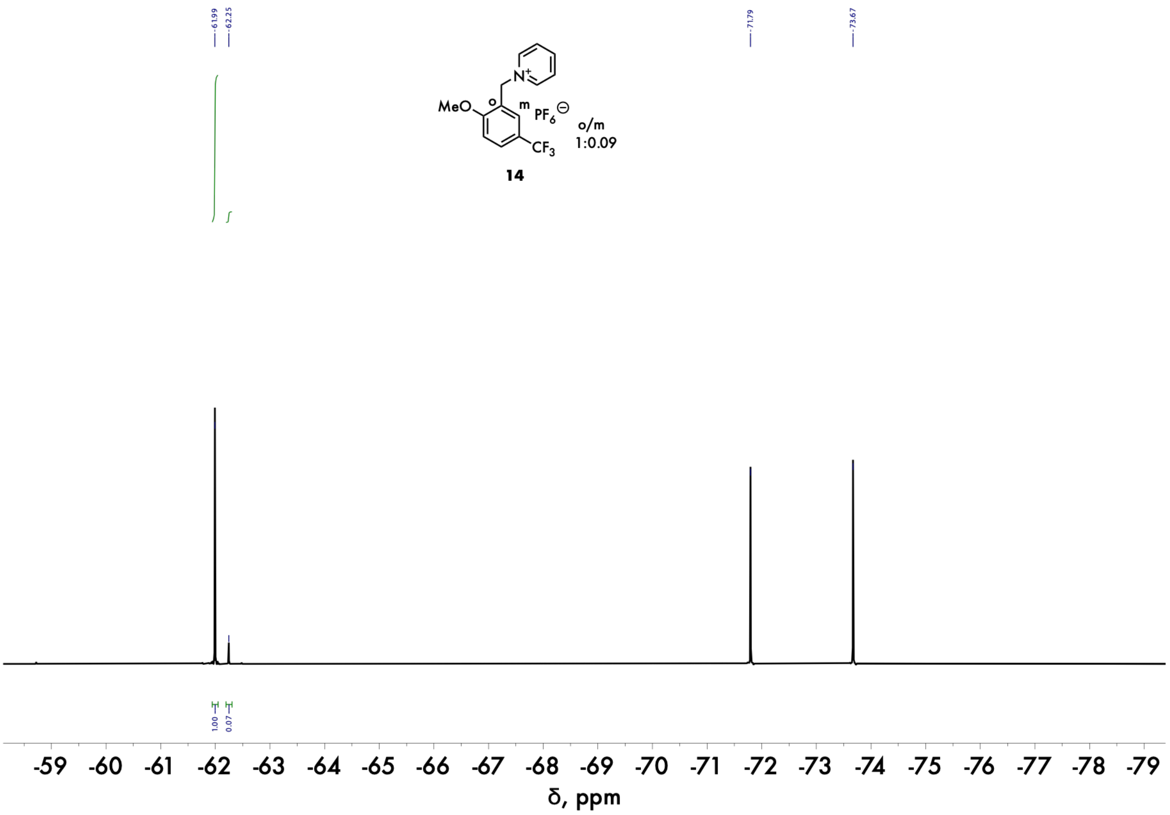

Figure S77. ^{19}F NMR (376 MHz, CD_3CN) spectrum of **14**.

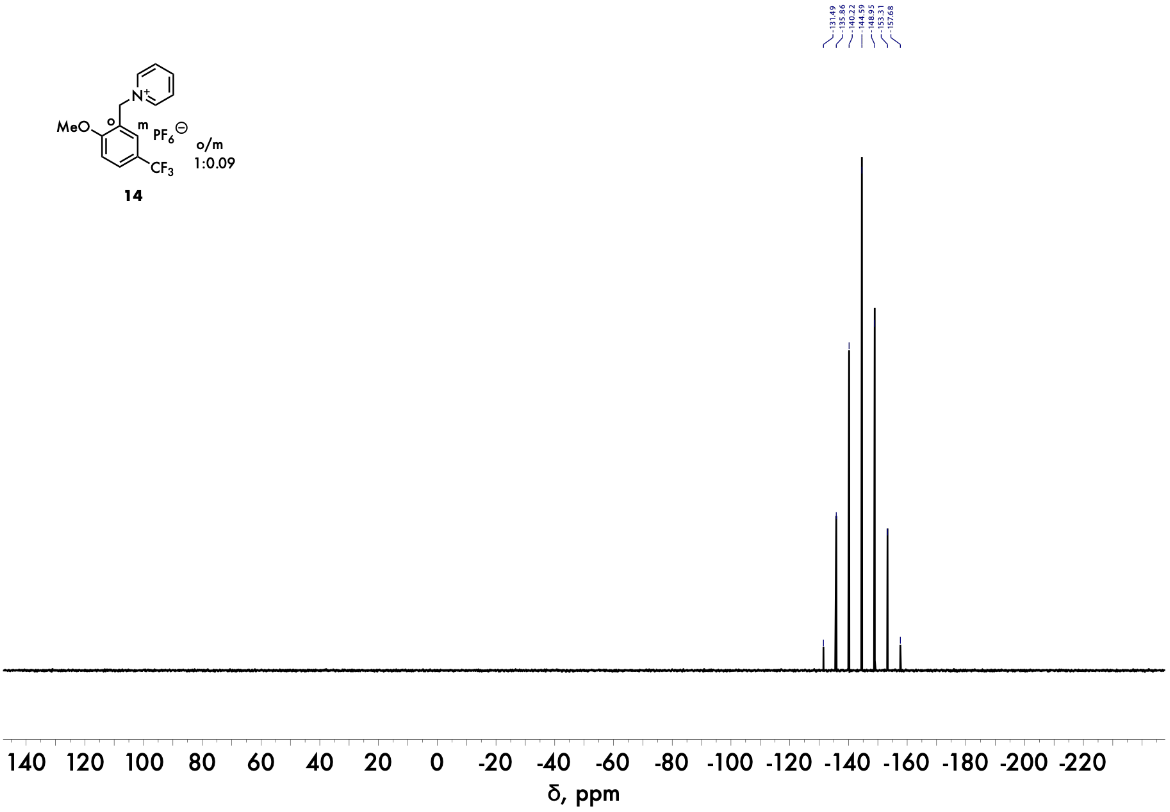

Figure S78. ^{31}P NMR (162 MHz, CD_3CN) spectrum of **14**.

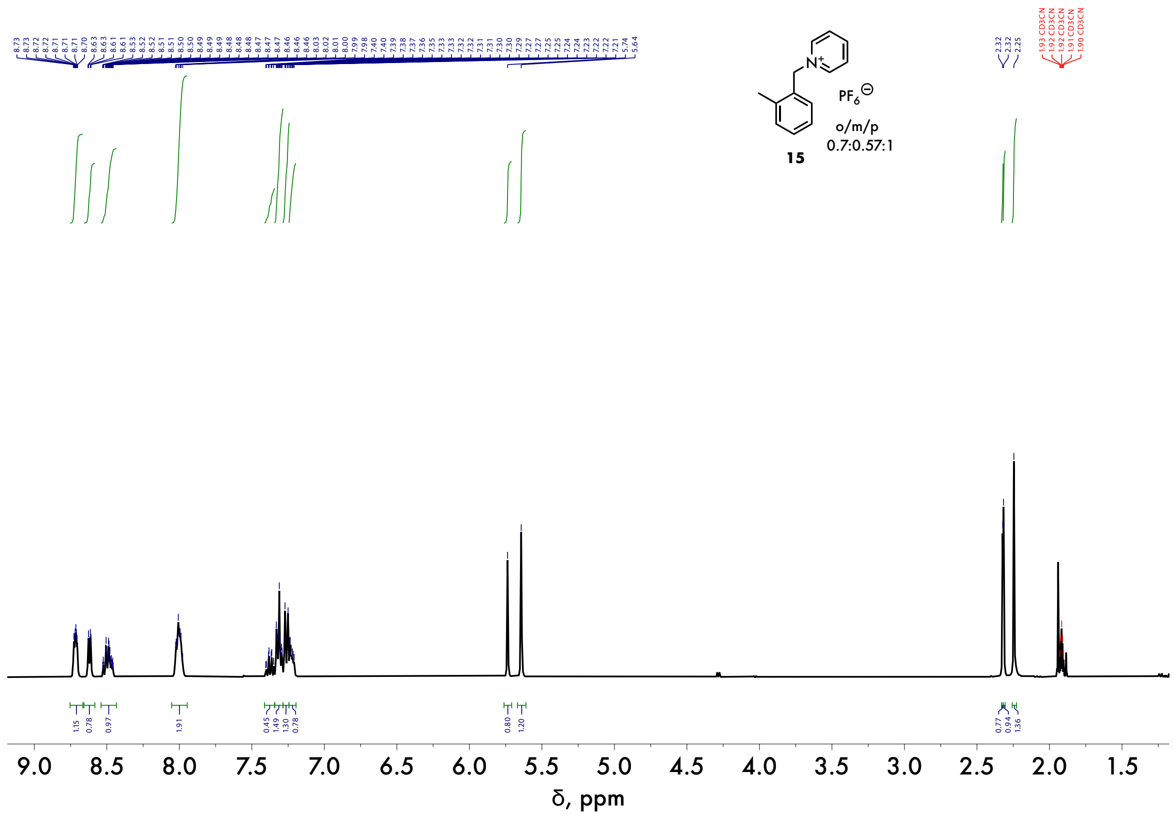

Figure S79. ^{1}H NMR (400 MHz, CD_3CN) spectrum of **15**.

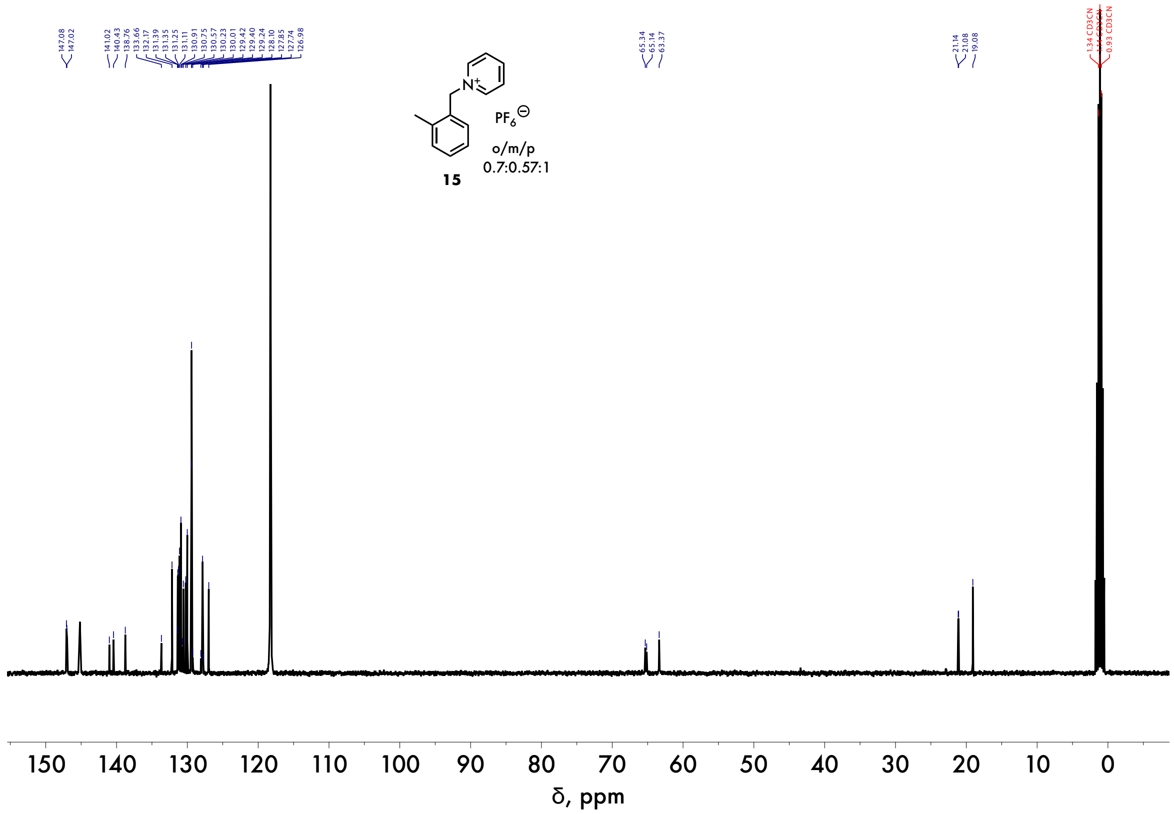

Figure S80. ^{13}C NMR (101 MHz, CD_3CN) spectrum of **15**.

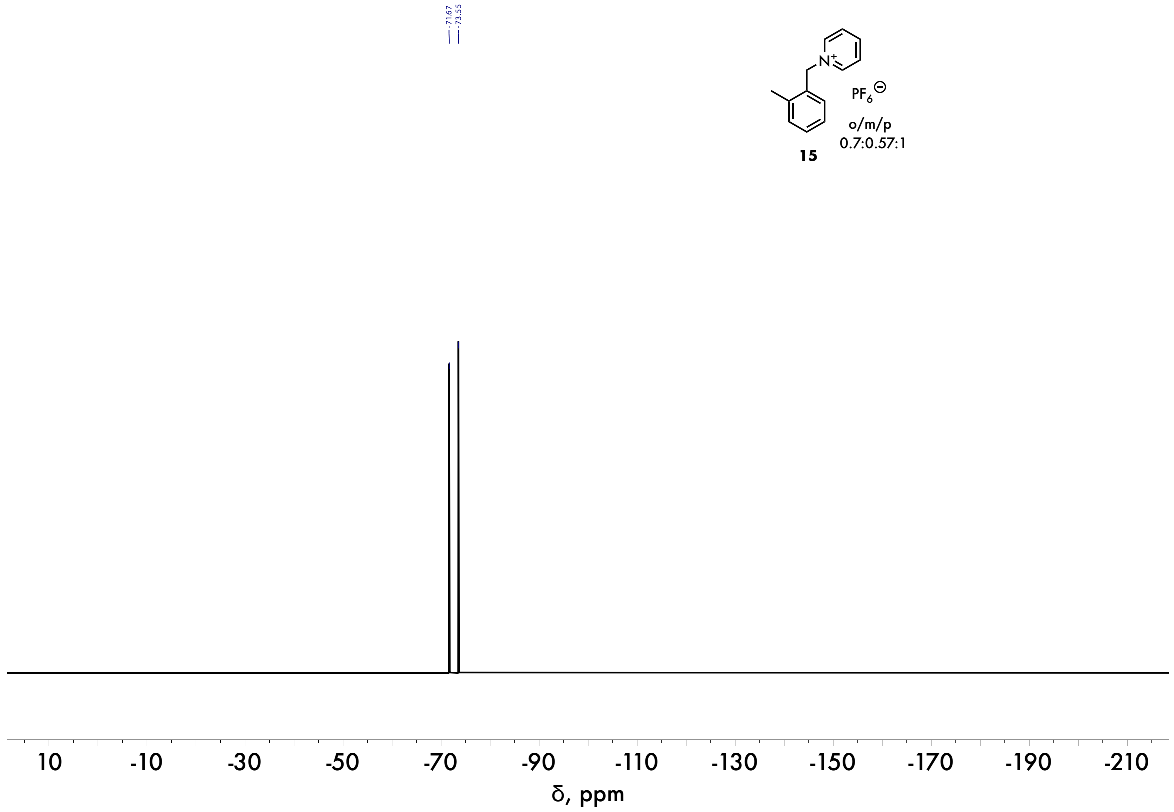

Figure S81. ^{19}F NMR (376 MHz, CD_3CN) spectrum of **15**.

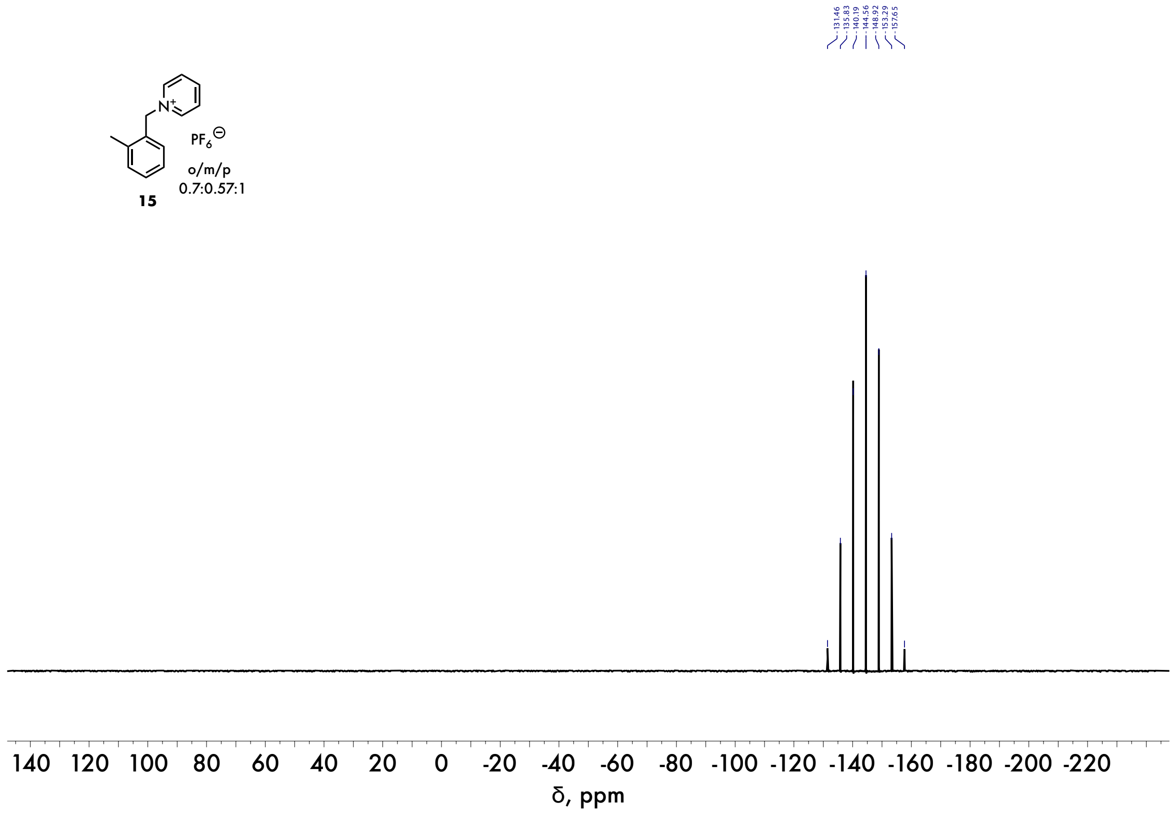

Figure S82. ^{31}P NMR (162 MHz, CD_3CN) spectrum of **15**.

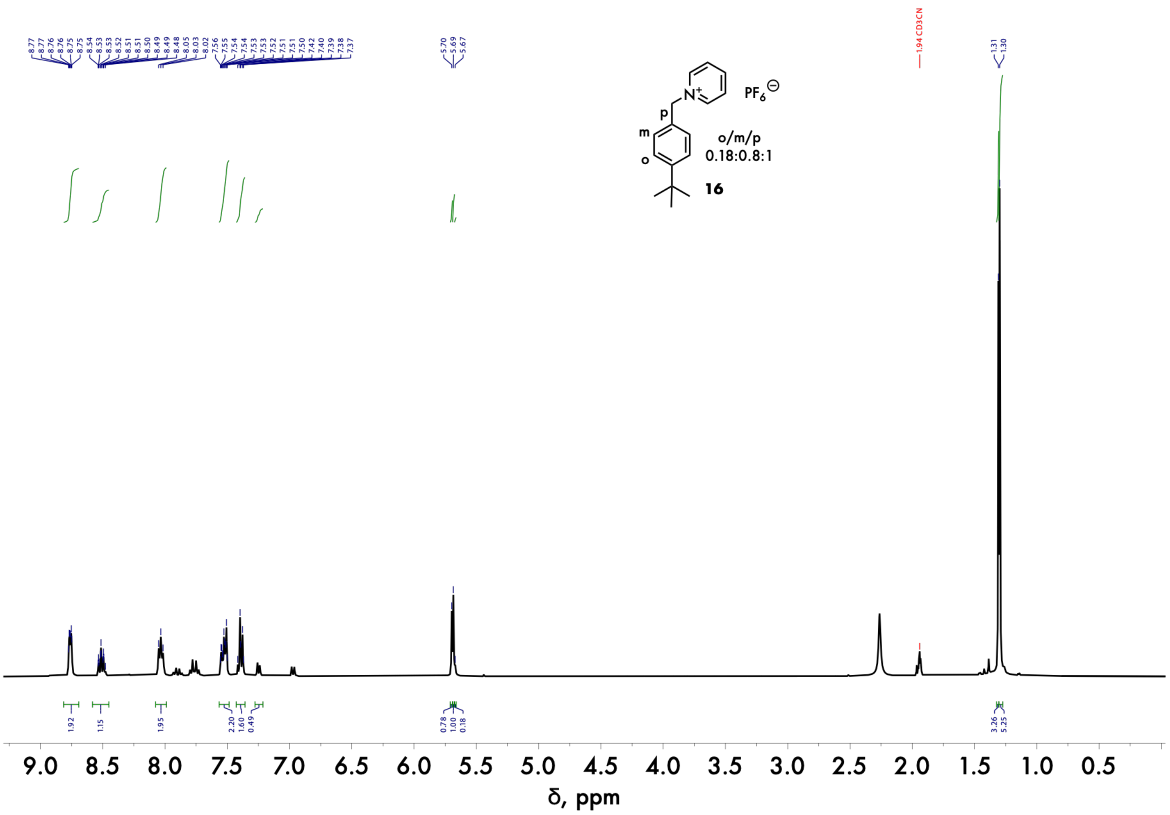

Figure S83. ^{1}H NMR (400 MHz, CD_3CN) spectrum of **16**.

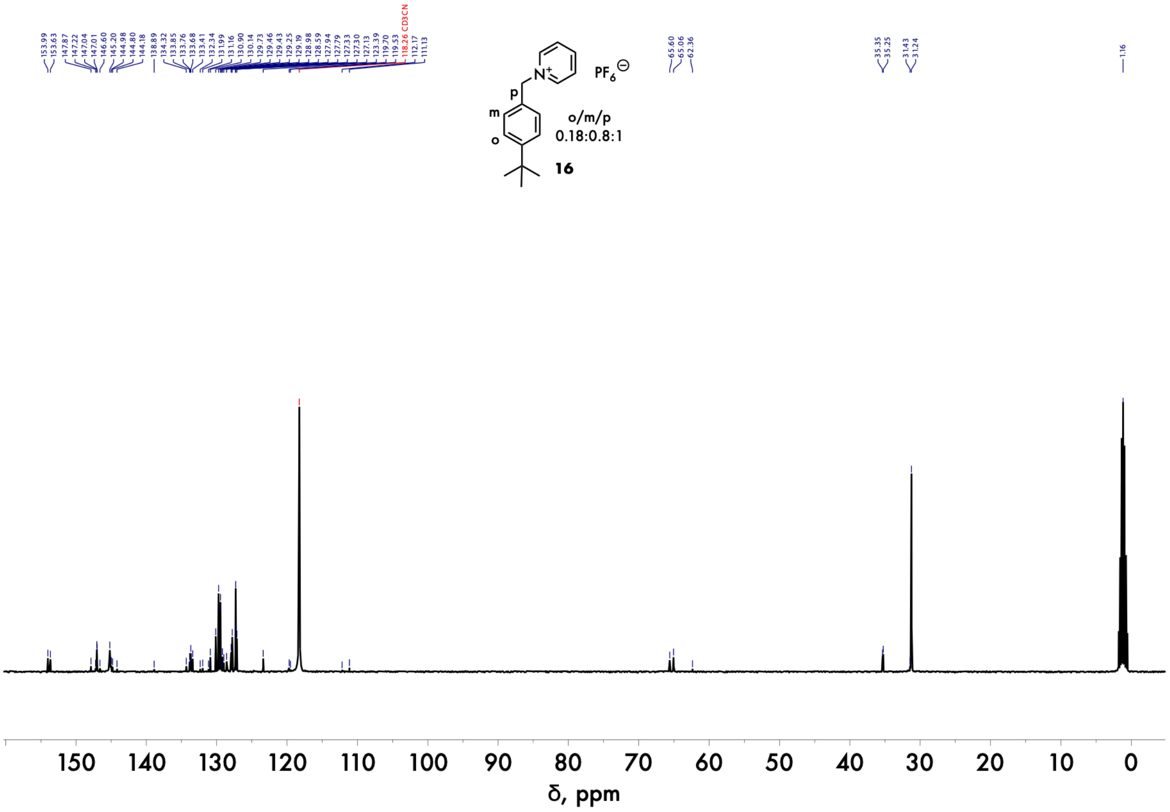

Figure S84. ^{13}C NMR (101 MHz, CD_3CN) spectrum of **16**.

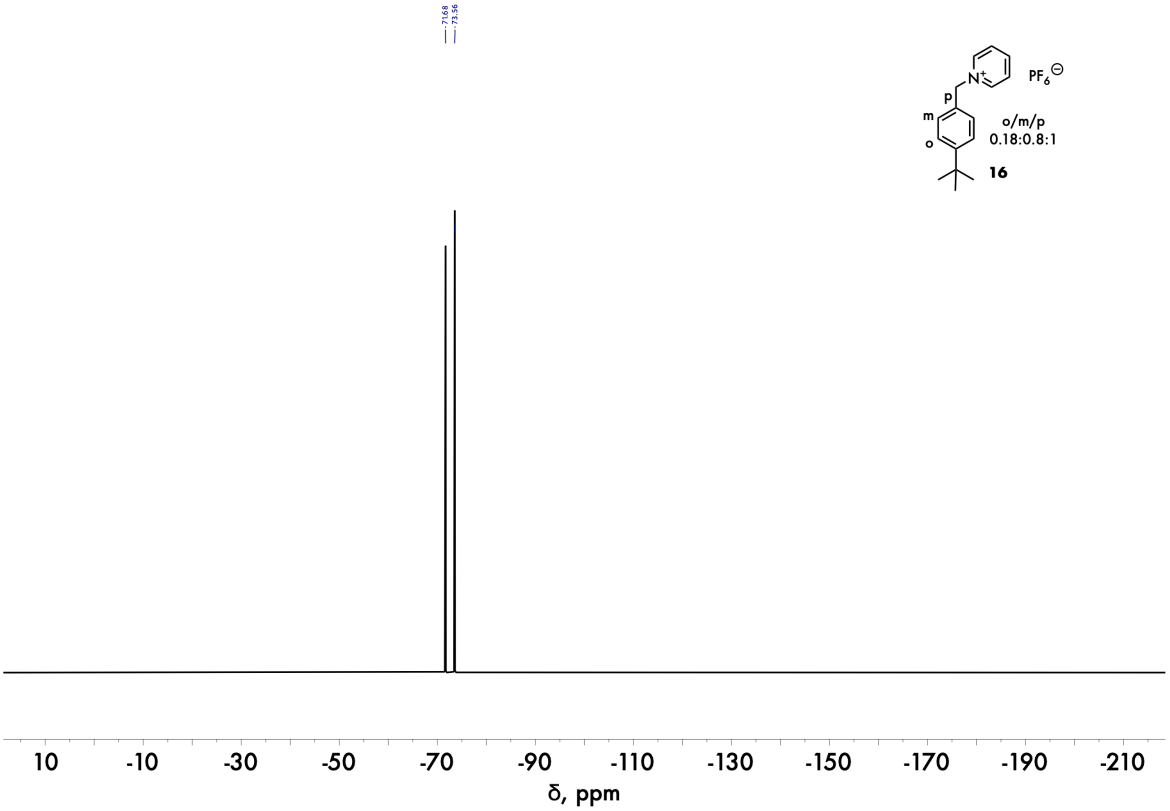

Figure S85. ^{19}F NMR (376 MHz, CD_3CN) spectrum of **16**.

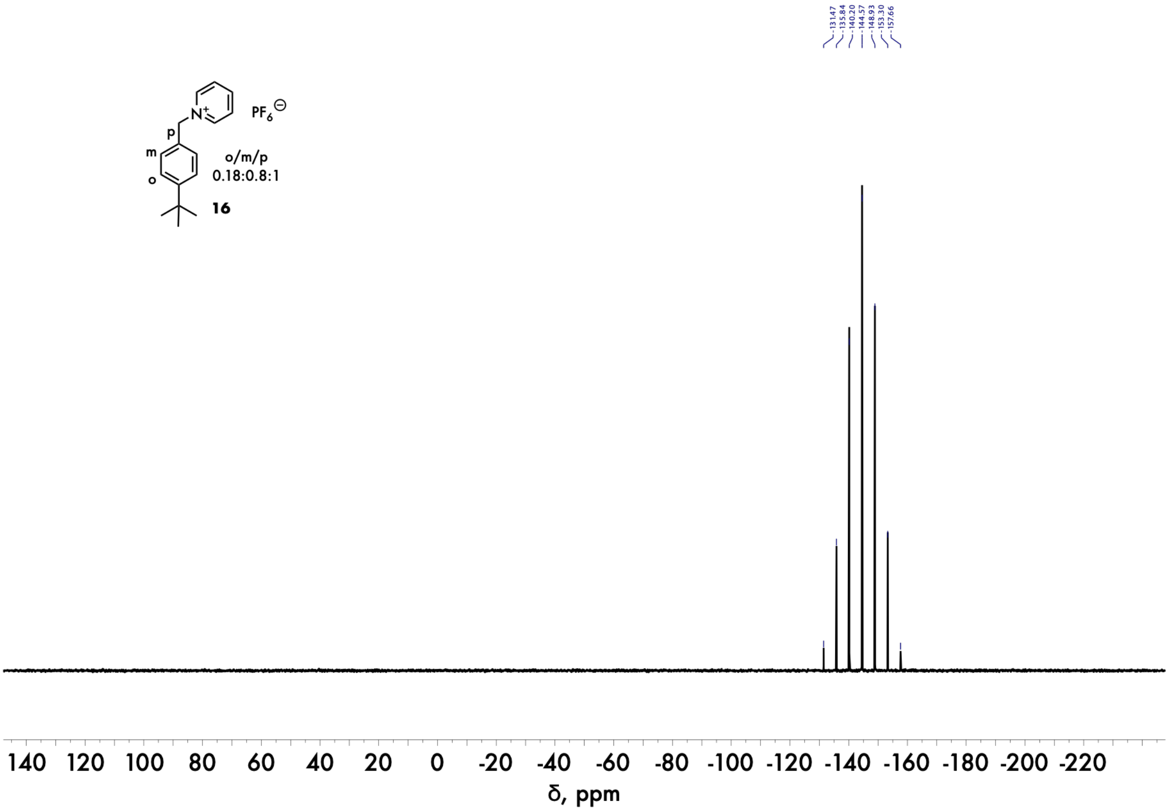

Figure S86. ^{31}P NMR (162 MHz, CD_3CN) spectrum of **16**.

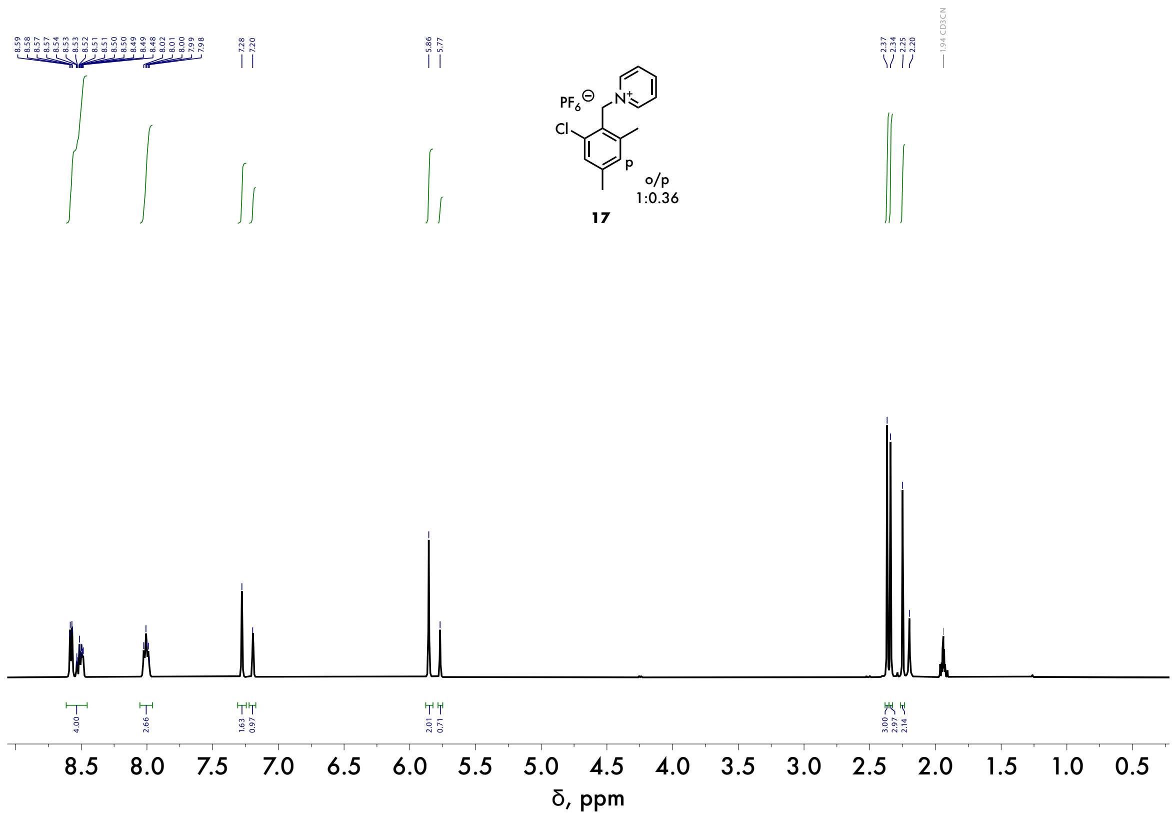

Figure S87. ^{1}H NMR (400 MHz, CD_3CN) spectrum of **17**.

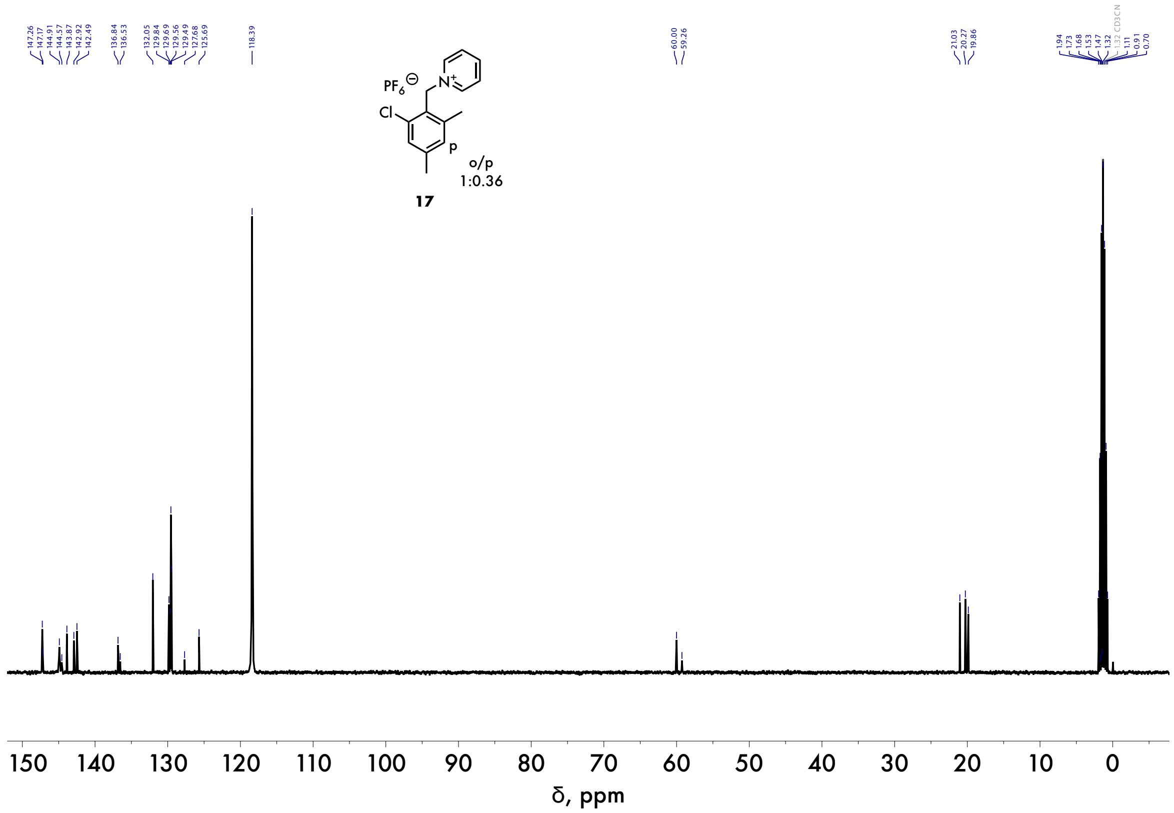

Figure S88. ^{13}C NMR (101 MHz, CD_3CN) spectrum of **17**.

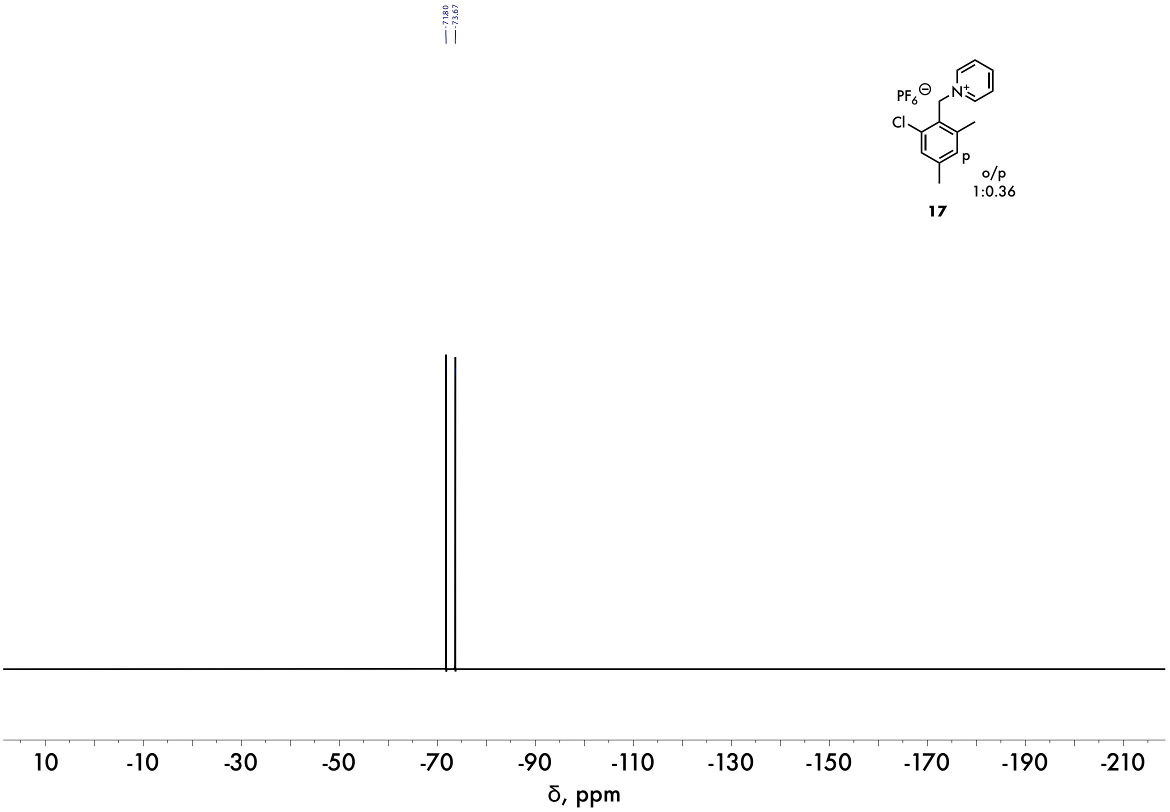

Figure S89. ^{19}F NMR (376 MHz, CD_3CN) spectrum of **17**.

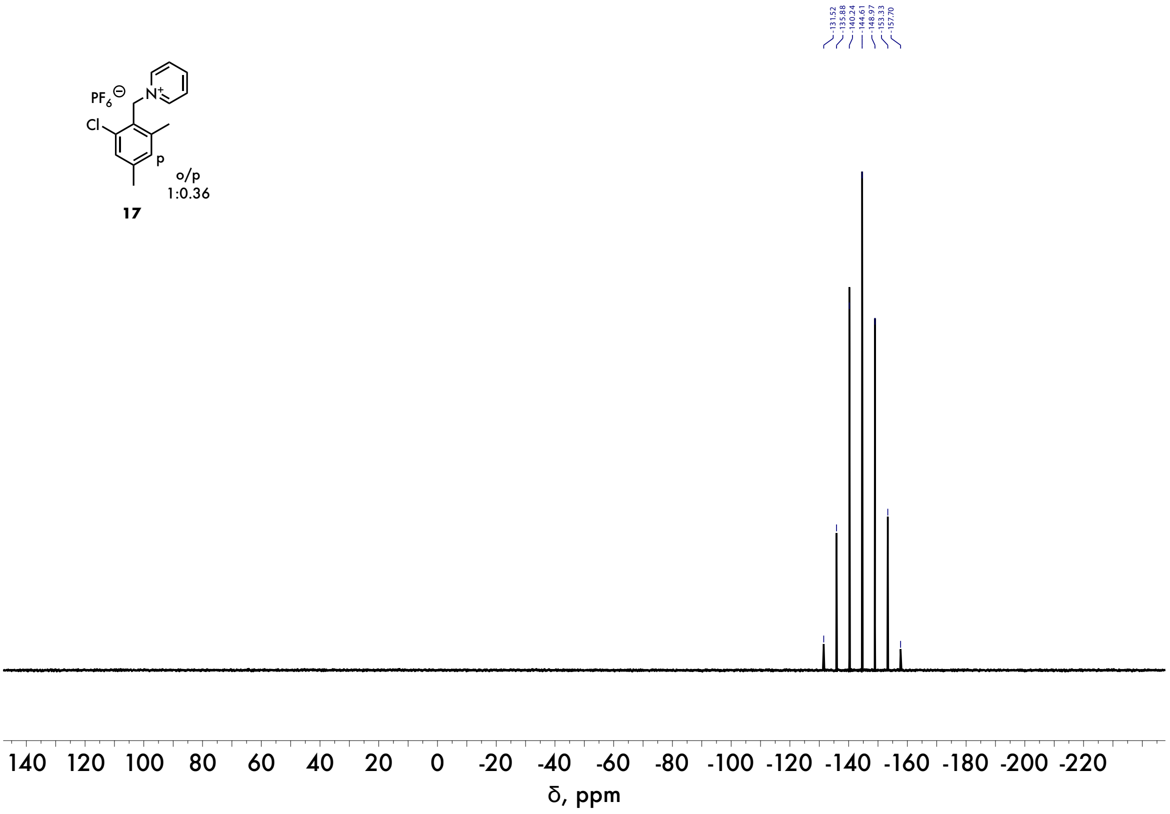

Figure S90. ^{31}P NMR (162 MHz, CD_3CN) spectrum of **17**.

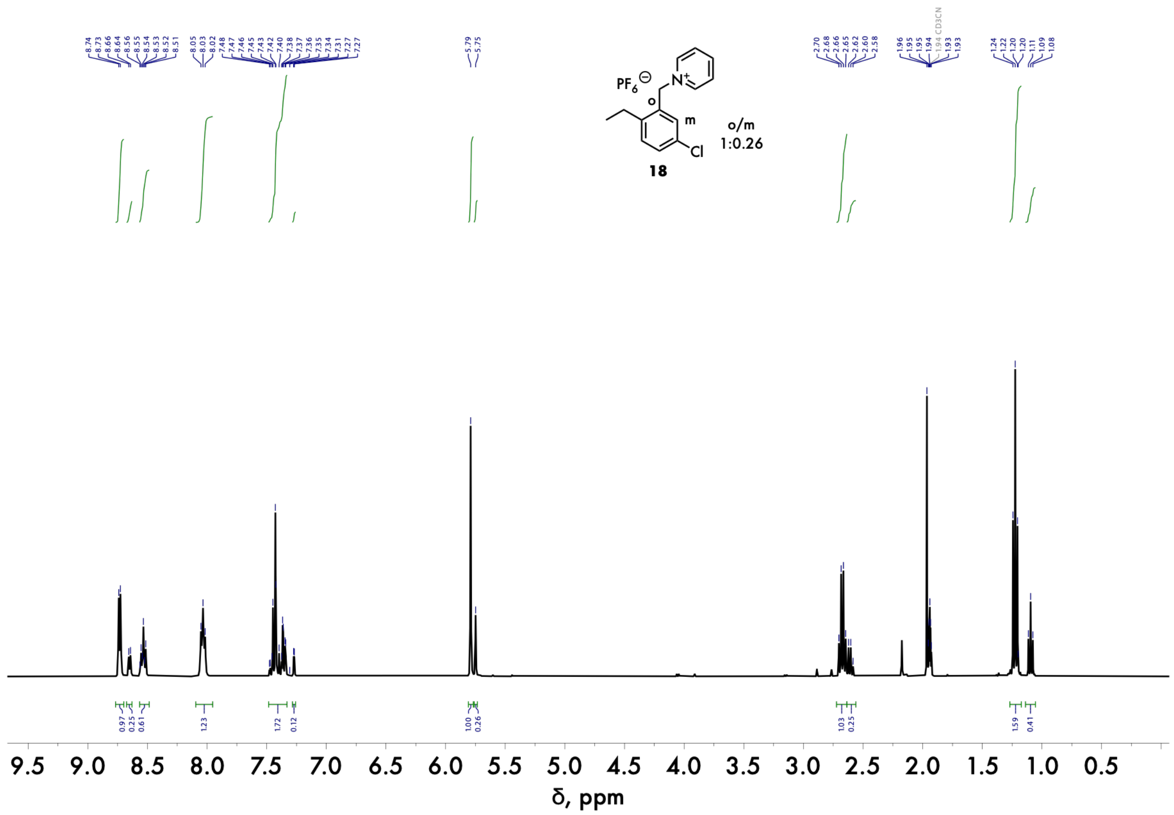

Figure S91. ^{1}H NMR (400 MHz, CD_3CN) spectrum of **18**.

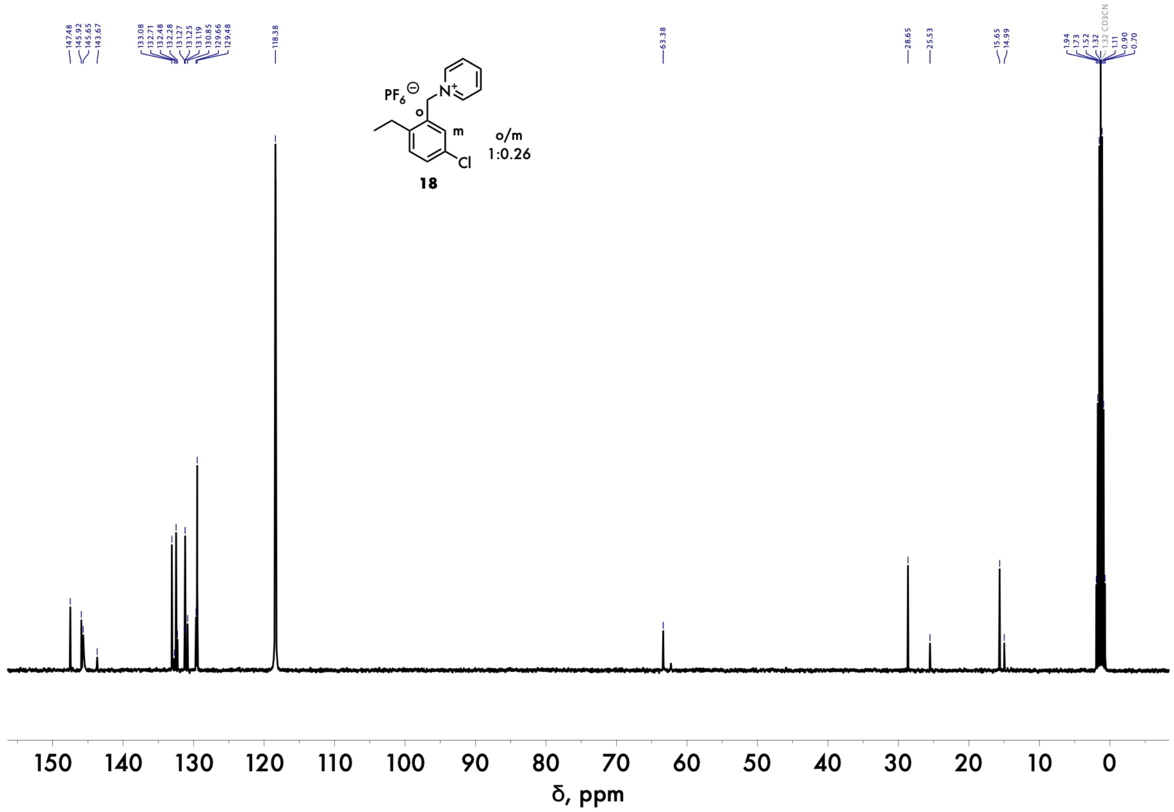

Figure S92. ^{13}C NMR (101 MHz, CD_3CN) spectrum of **18**.

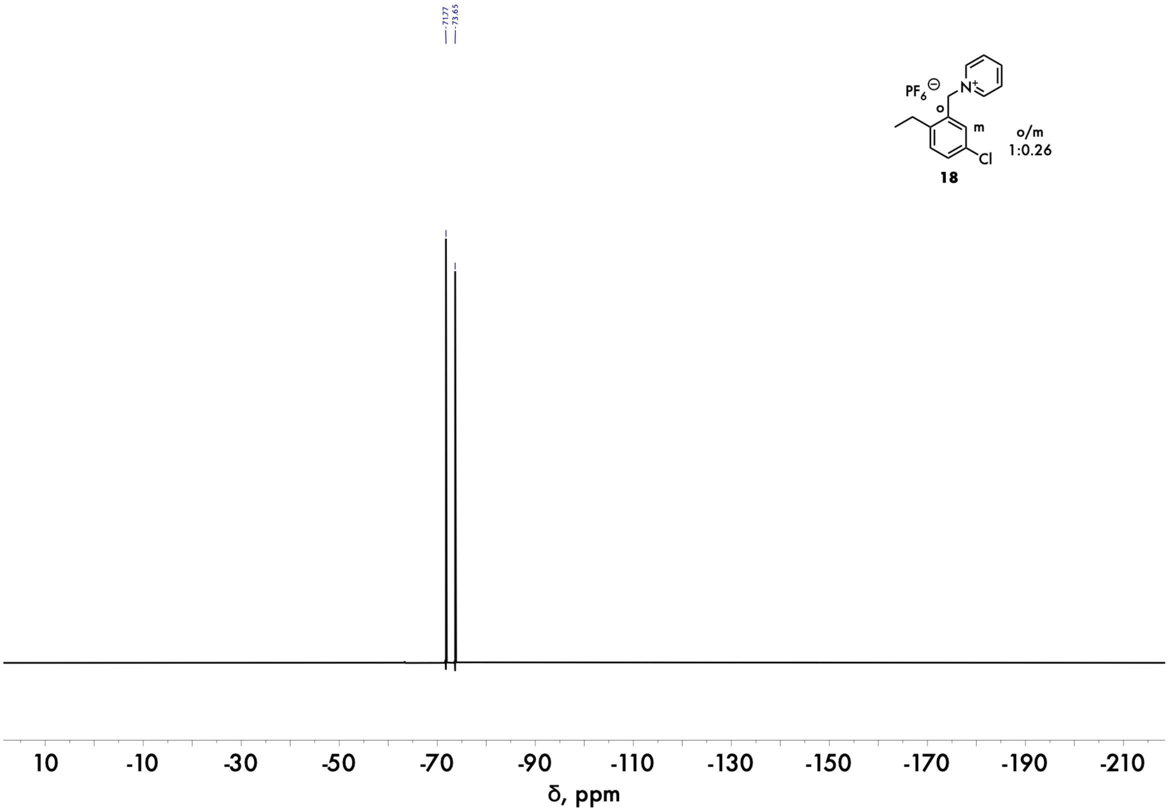

Figure S93. ^{19}F NMR (376 MHz, CD_3CN) spectrum of **18**.

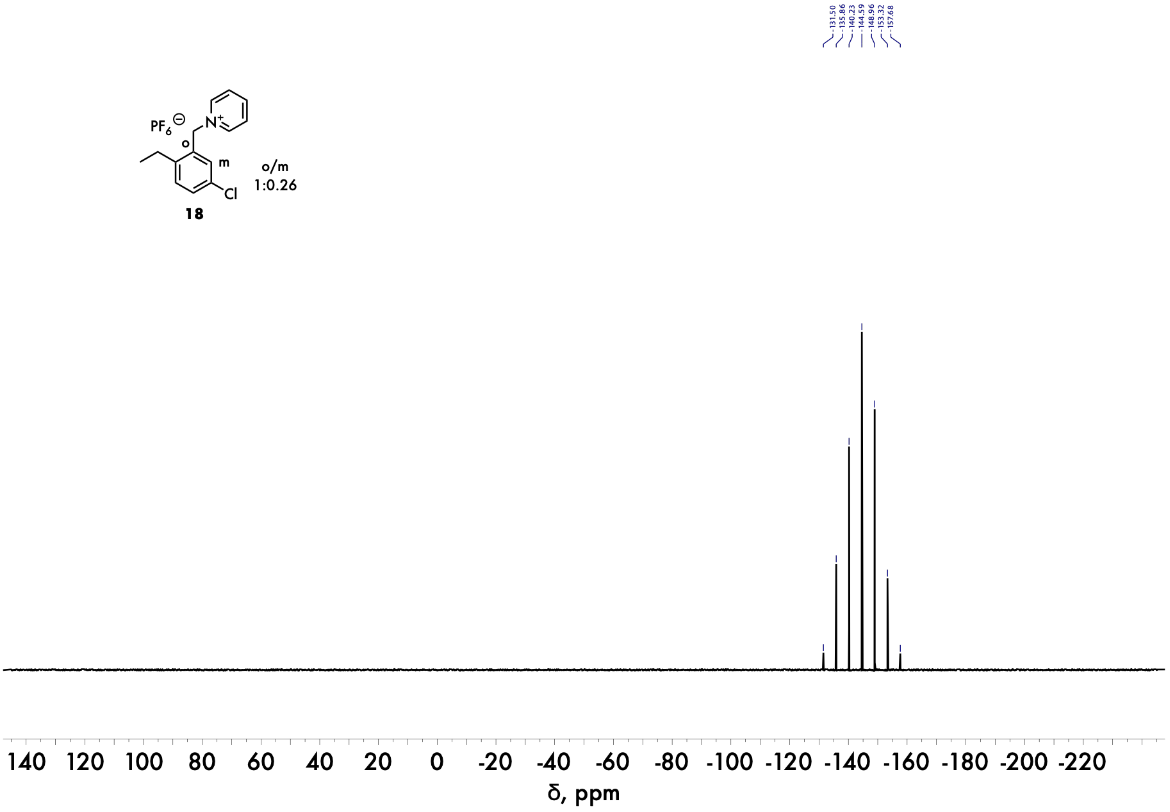

Figure S94. ^{31}P NMR (162 MHz, CD_3CN) spectrum of **18**.

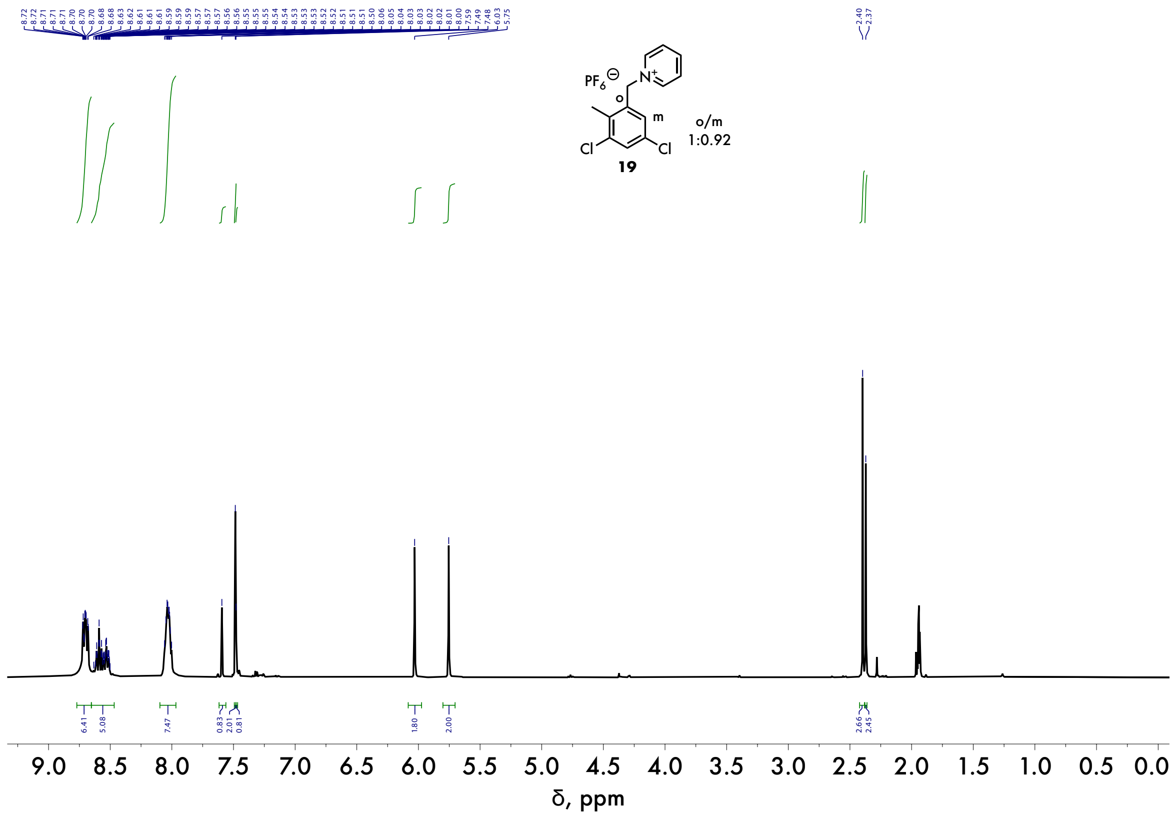

Figure S95. ^{1}H NMR (400 MHz, CD_3CN) spectrum of **19** (the product coeluted with one molecule of Py HPF_6).

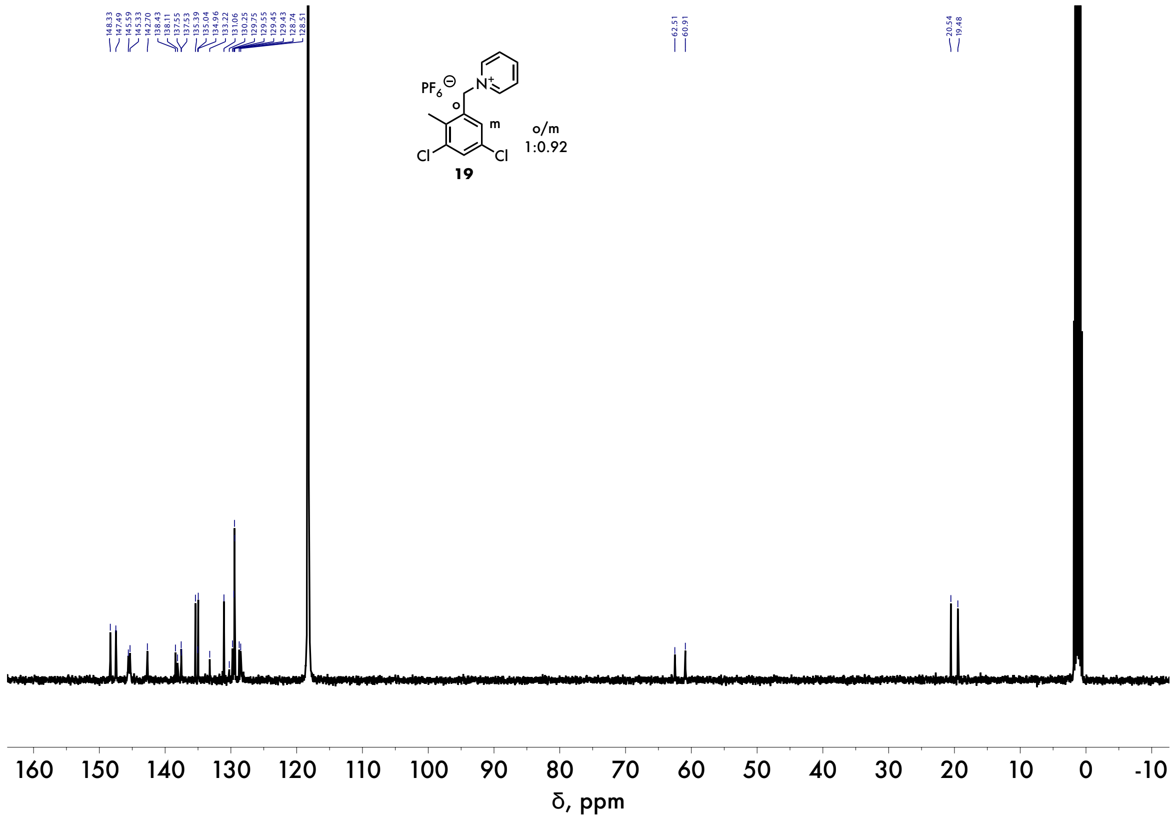

Figure S96. ^{13}C NMR (101 MHz, CD_3CN) spectrum of **19**.

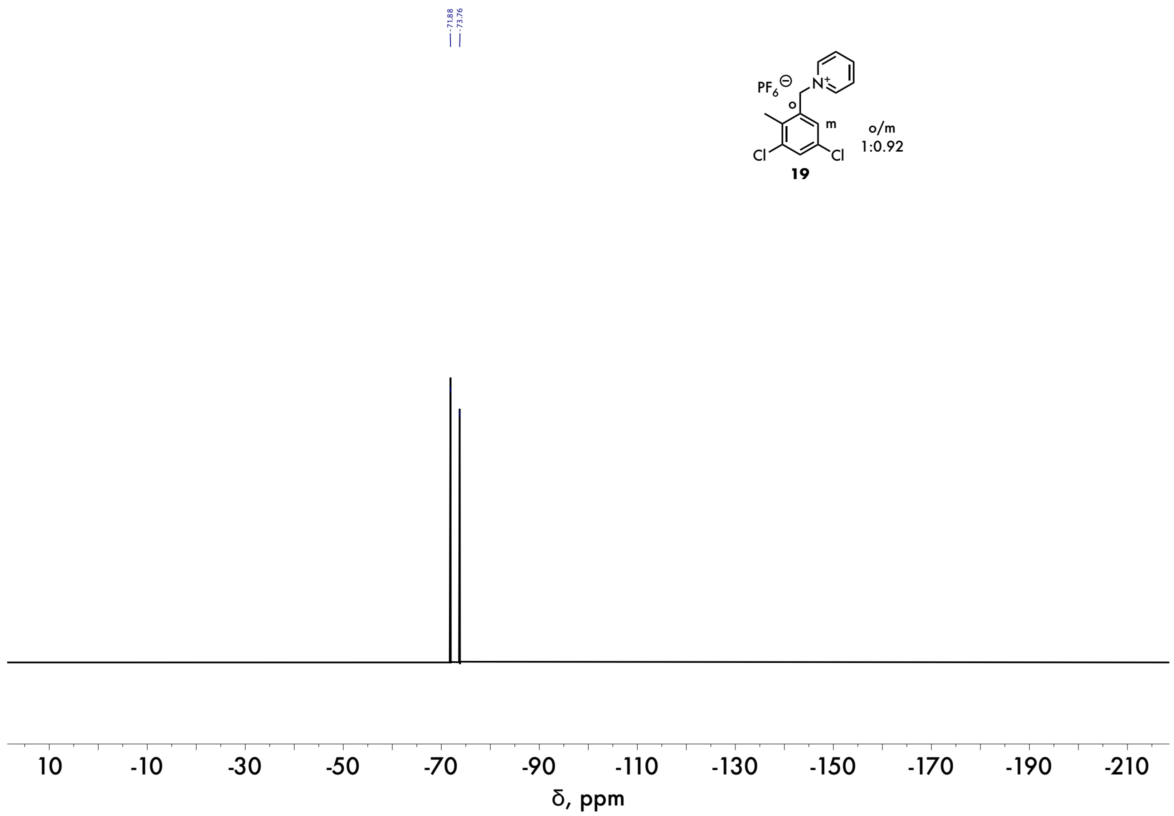

Figure S97. ^{19}F NMR (376 MHz, CD_3CN) spectrum of **19**.

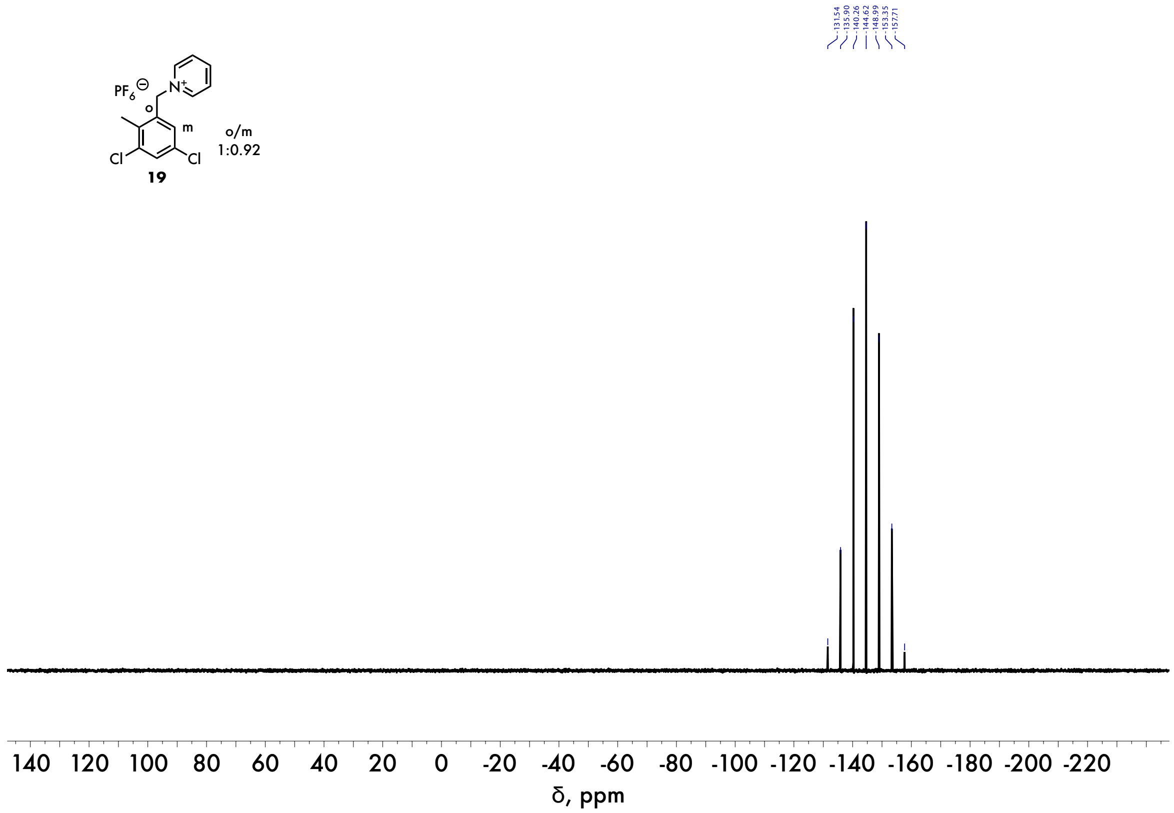

Figure S98. ^{31}P NMR (162 MHz, CD_3CN) spectrum of **19**.

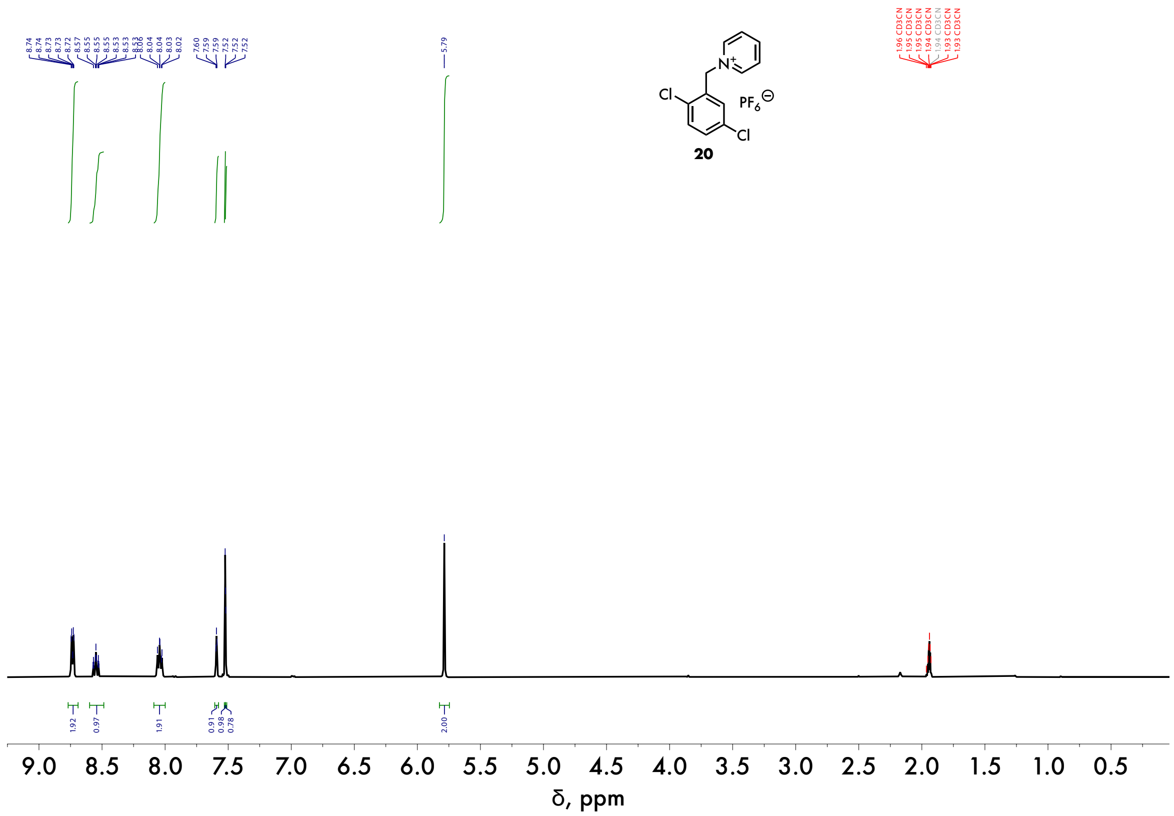

Figure S99. ^{1}H NMR (400 MHz, CD_3CN) spectrum of **20**.

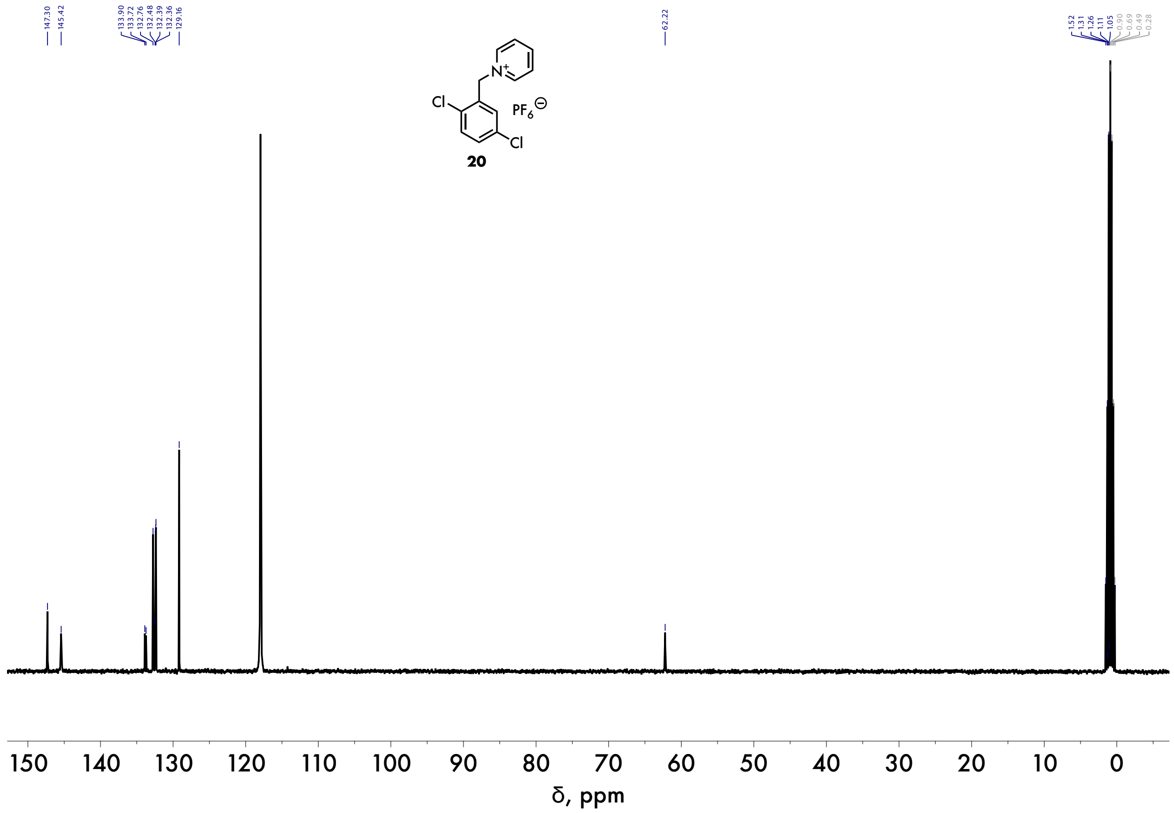

Figure S100. ^{13}C NMR (101 MHz, CD_3CN) spectrum of **20**.

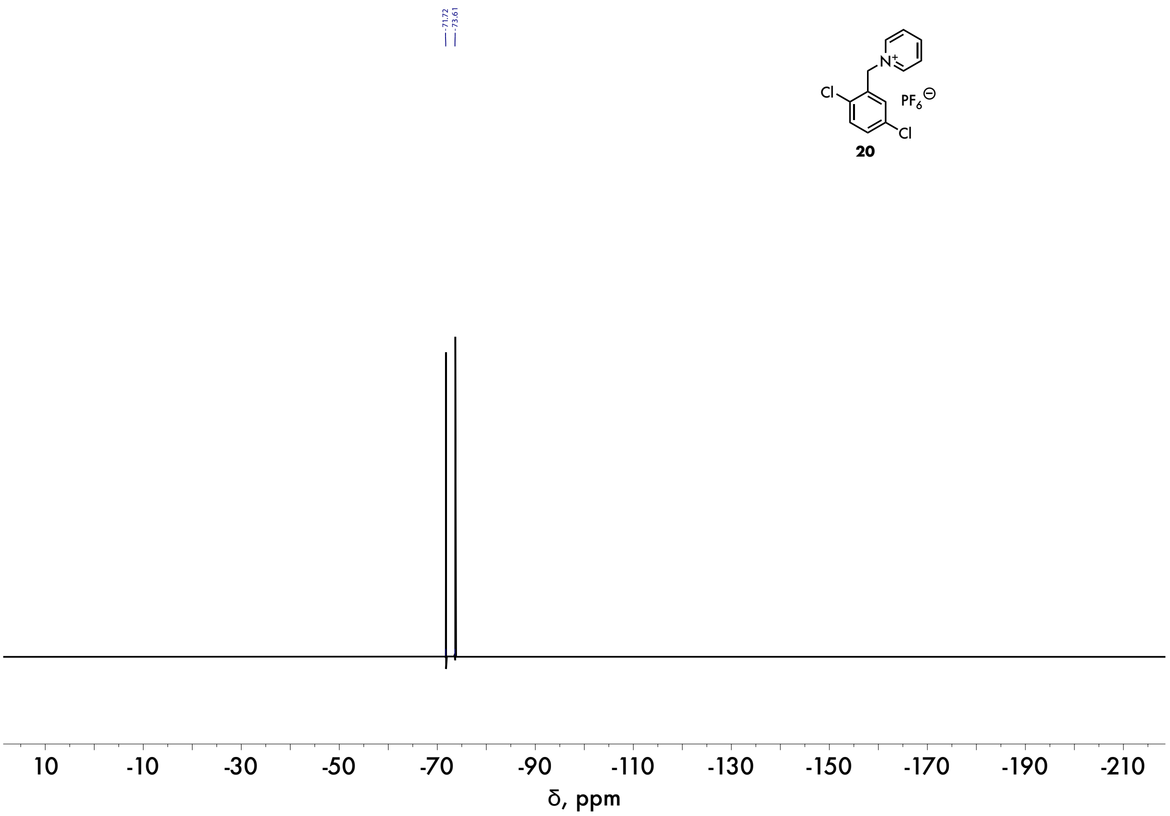

Figure S101. ^{19}F NMR (376 MHz, CD_3CN) spectrum of **20**.

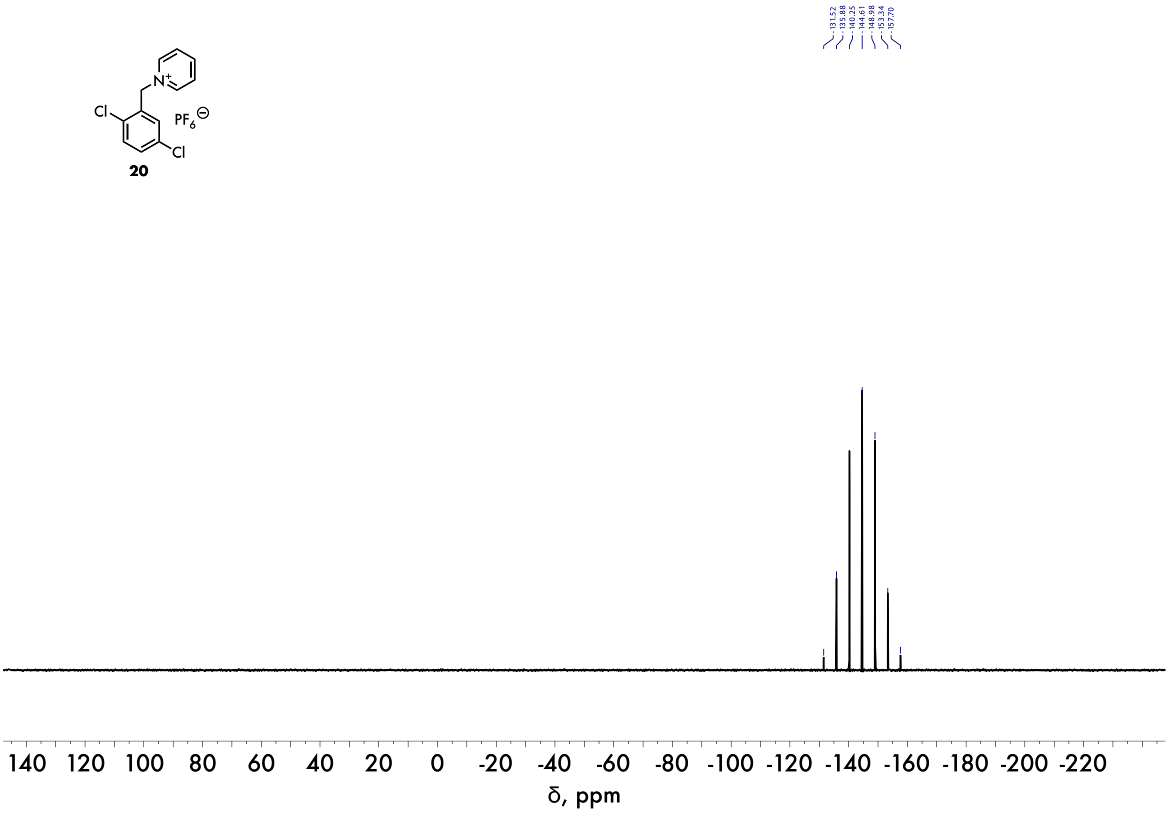

Figure S102. ^{31}P NMR (162 MHz, CD_3CN) spectrum of **20**.

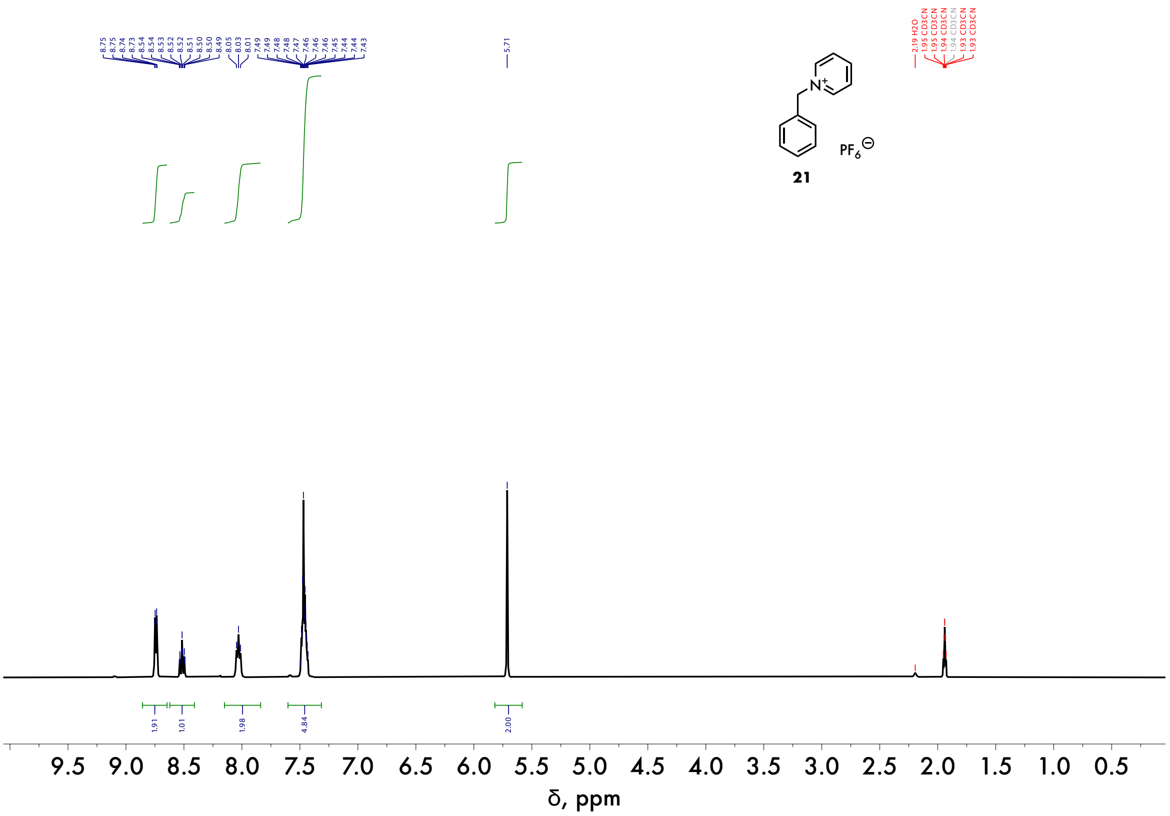

Figure S103. ^{1}H NMR (400 MHz, CD_3CN) spectrum of **21**.

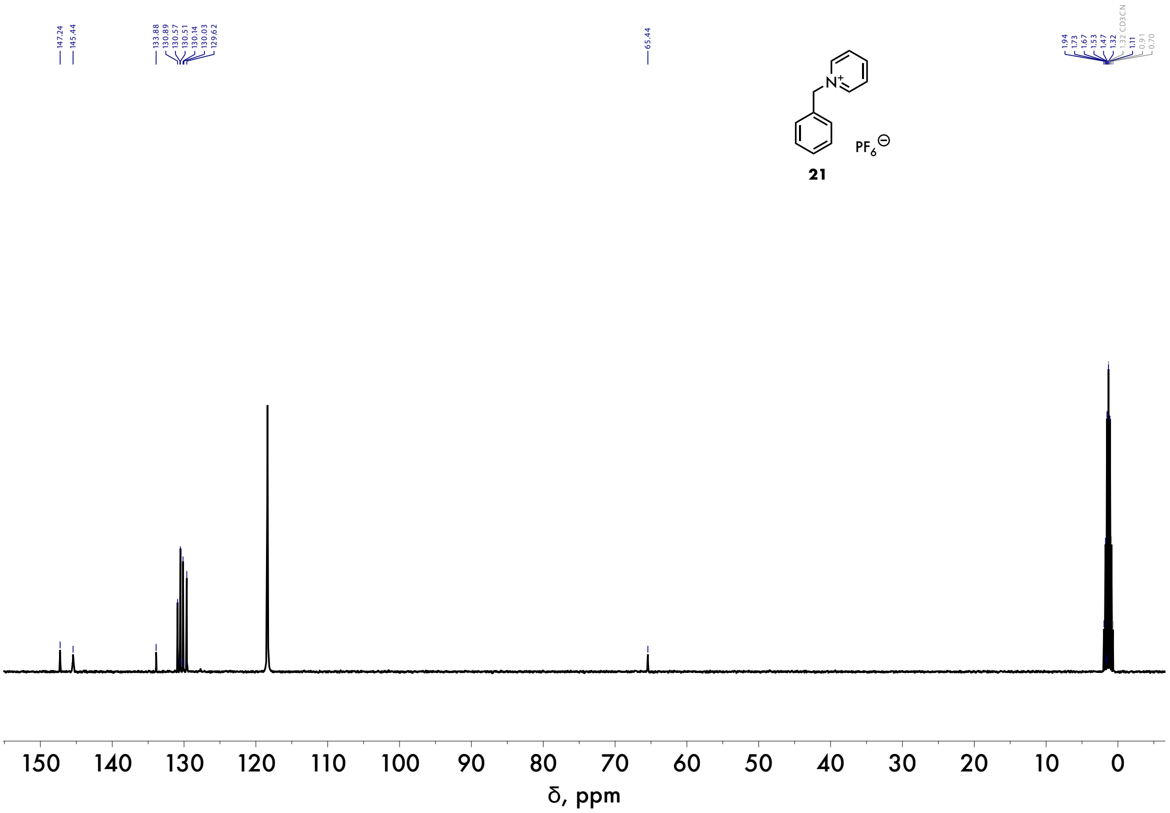

Figure S104. ^{13}C NMR (101 MHz, CD_3CN) spectrum of **21**.

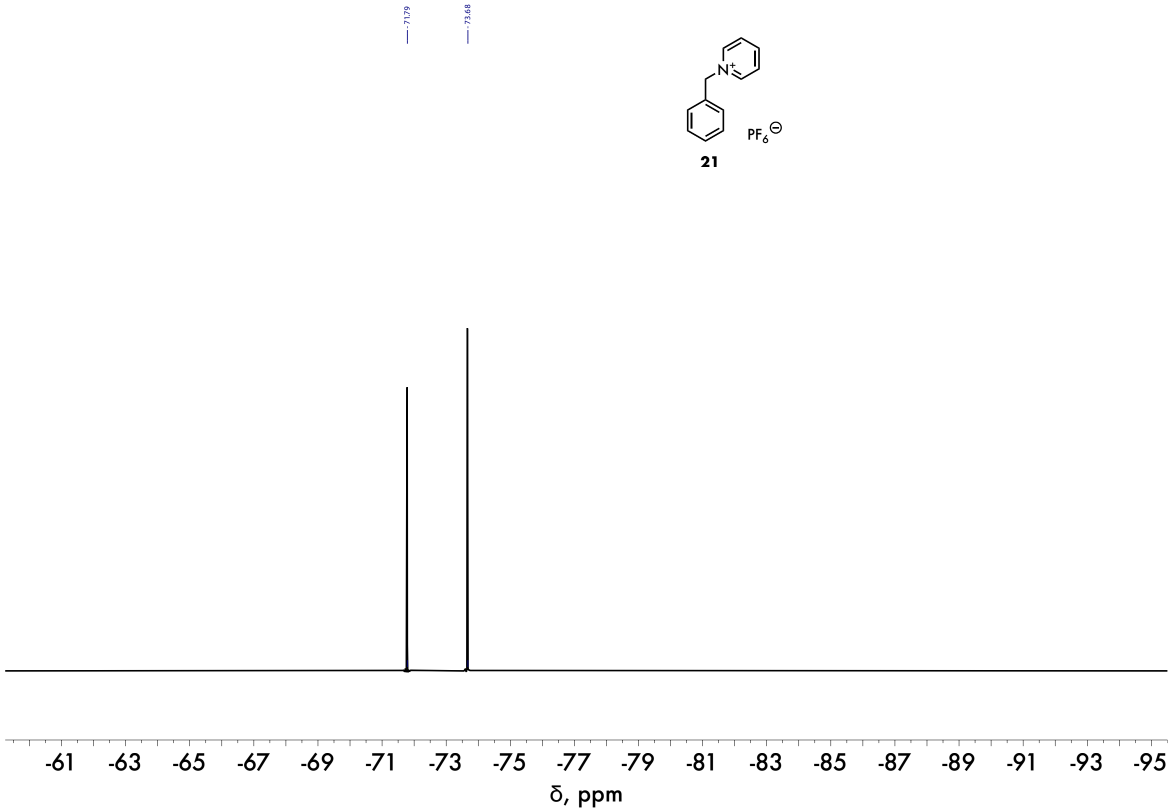

Figure S105. ^{19}F NMR (376 MHz, CD_3CN) spectrum of **21**.

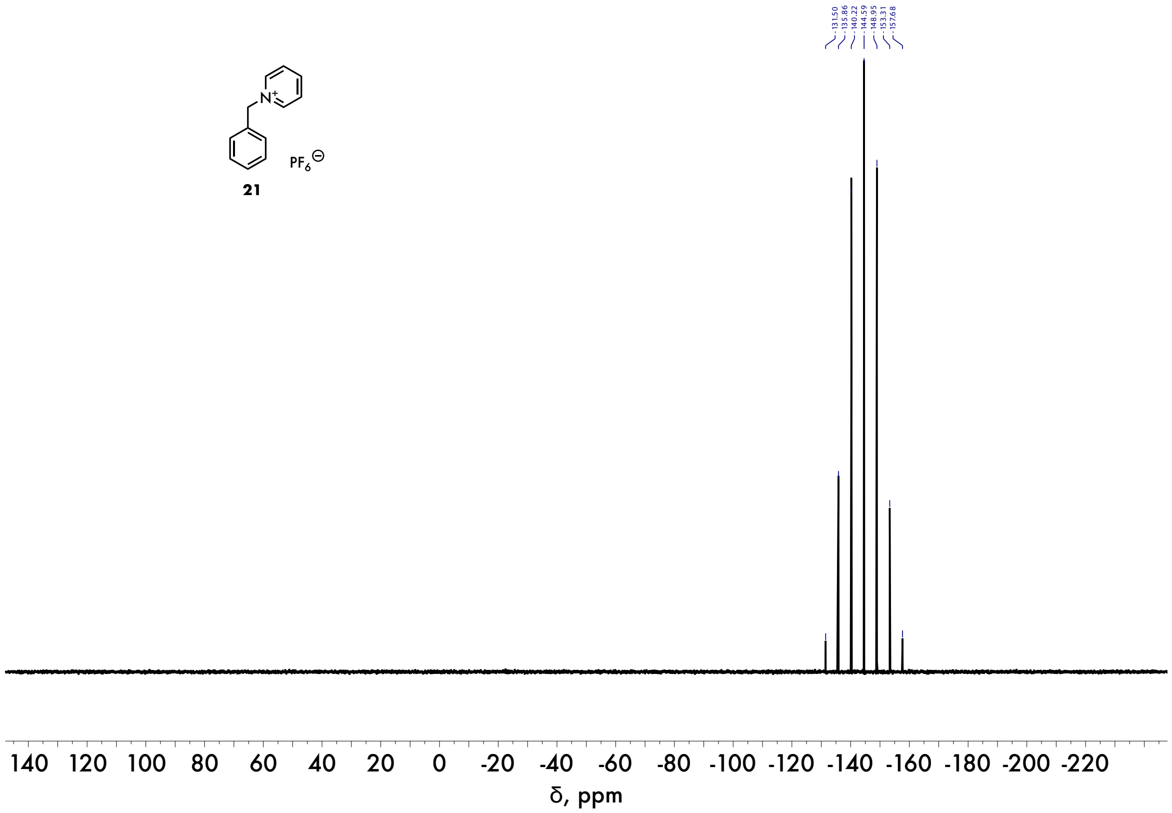

Figure S106. ^{31}P NMR (162 MHz, CD_3CN) spectrum of **21**.

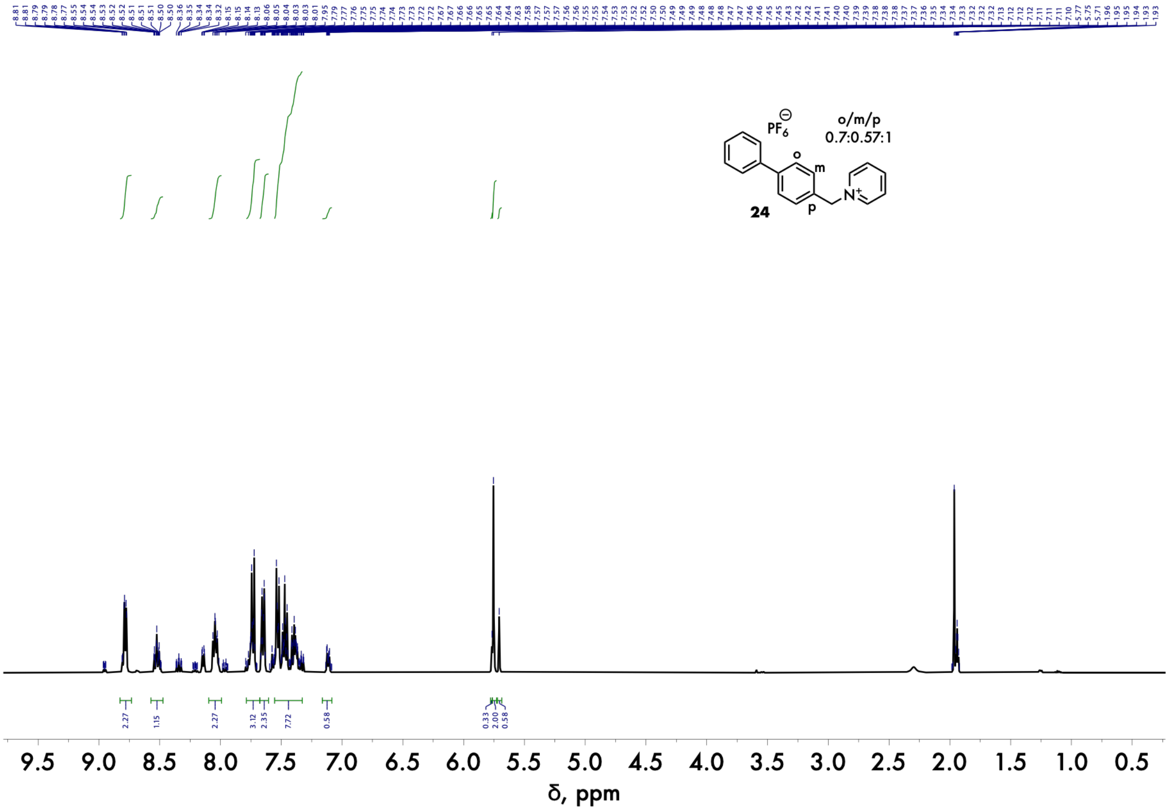

Figure S107. ^{1}H NMR (400 MHz, CD_3CN) spectrum of **24**.

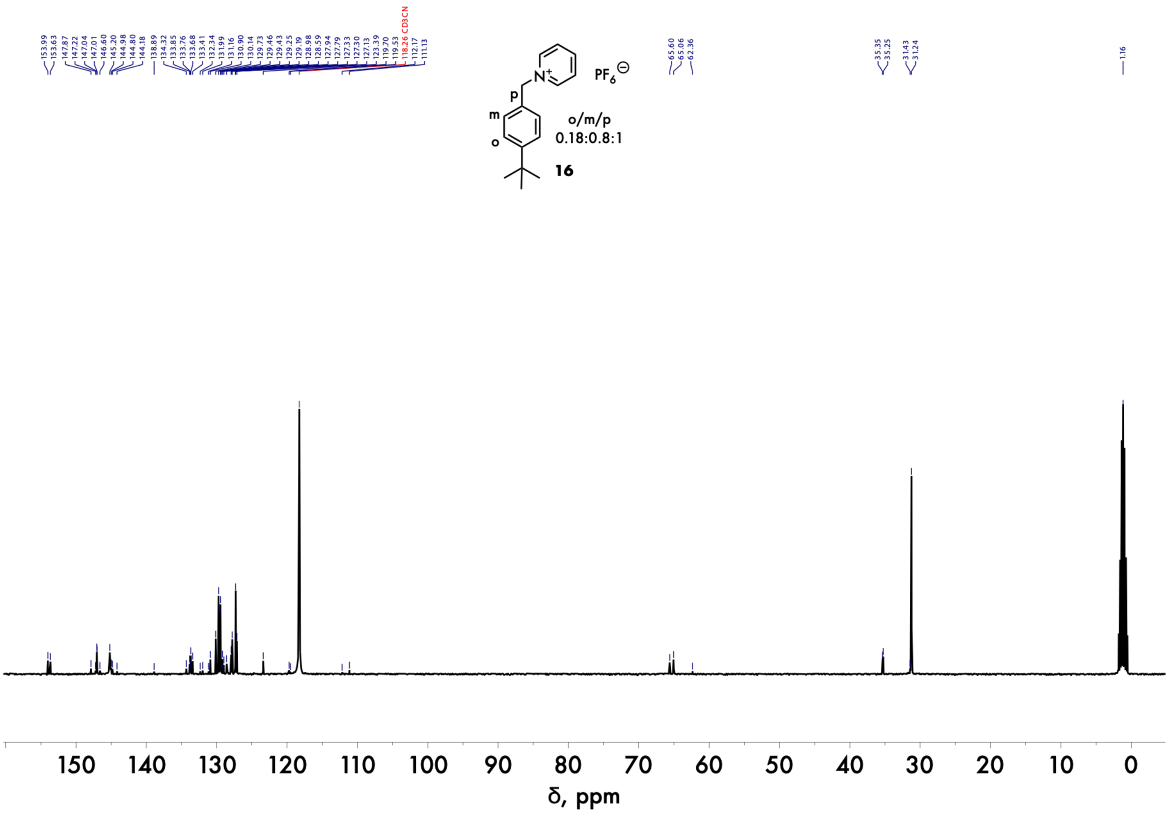

Figure S108. ^{13}C NMR (101 MHz, CD_3CN) spectrum of **24**.

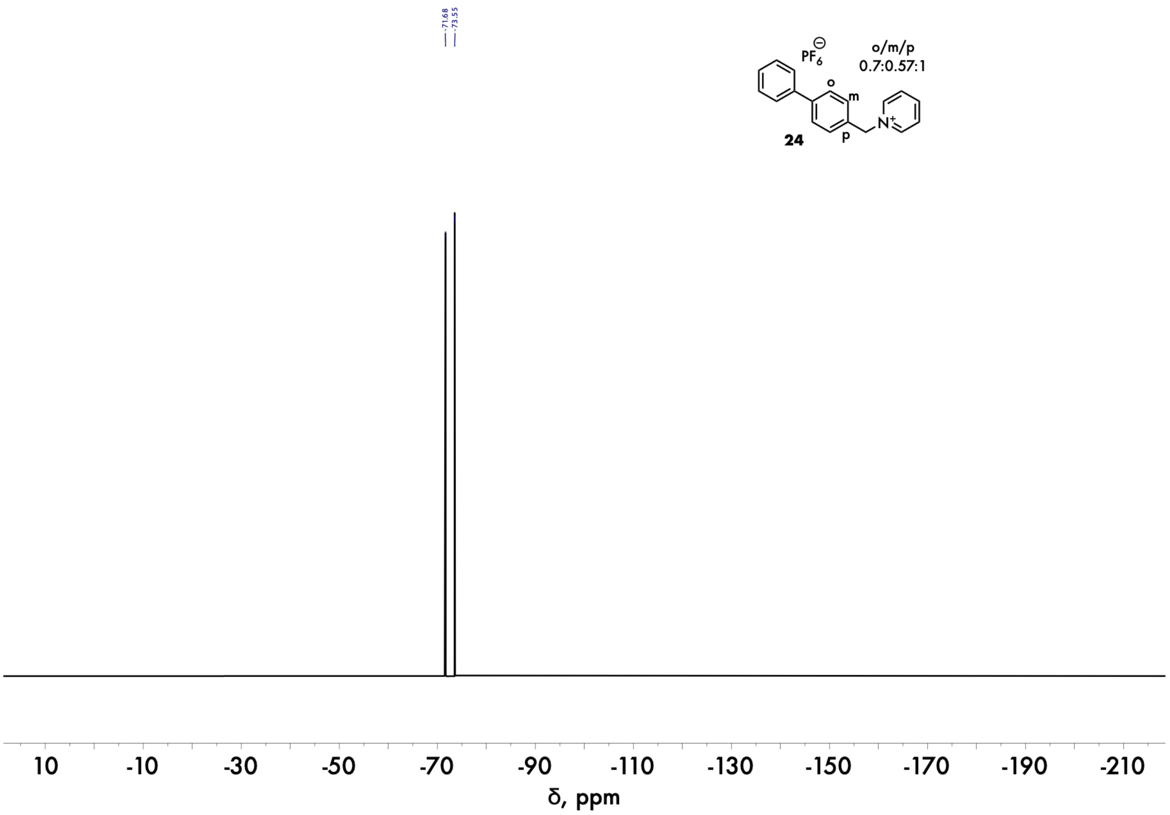

Figure S109. ^{19}F NMR (376 MHz, CD_3CN) spectrum of **24**.

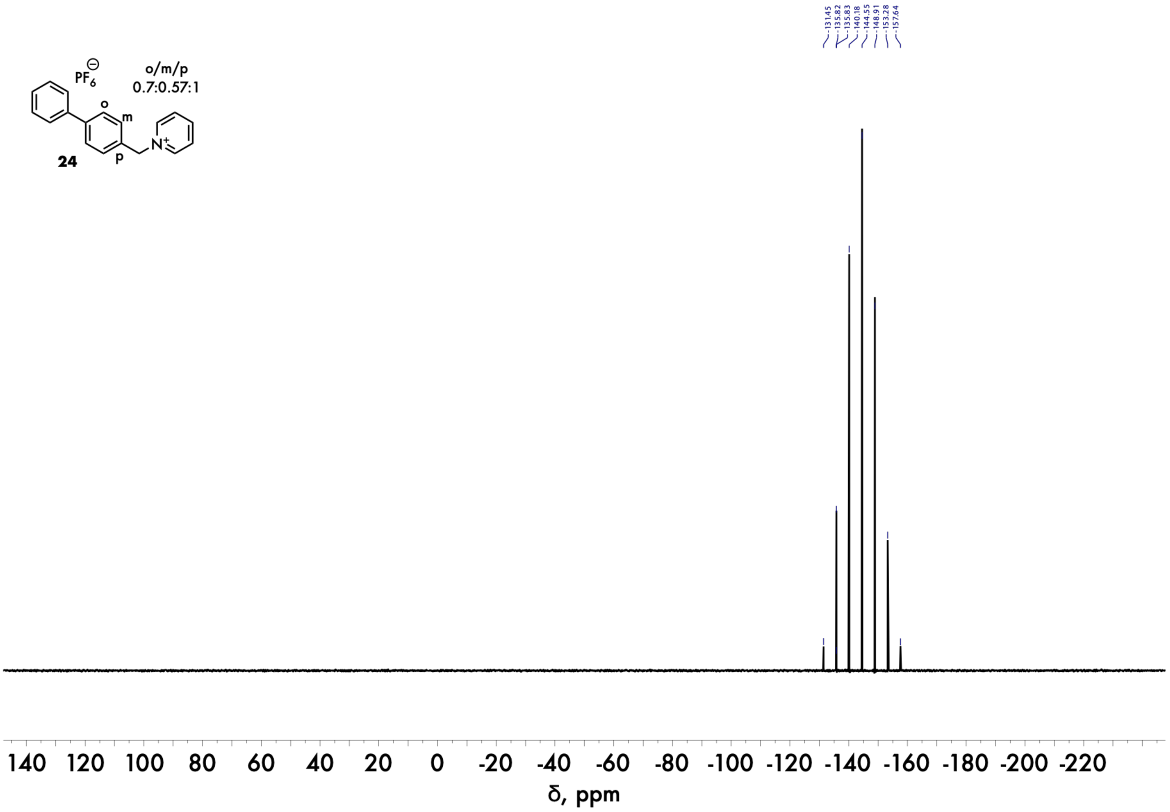

Figure S110. ^{31}P NMR (162 MHz, CD_3CN) spectrum of **24**.

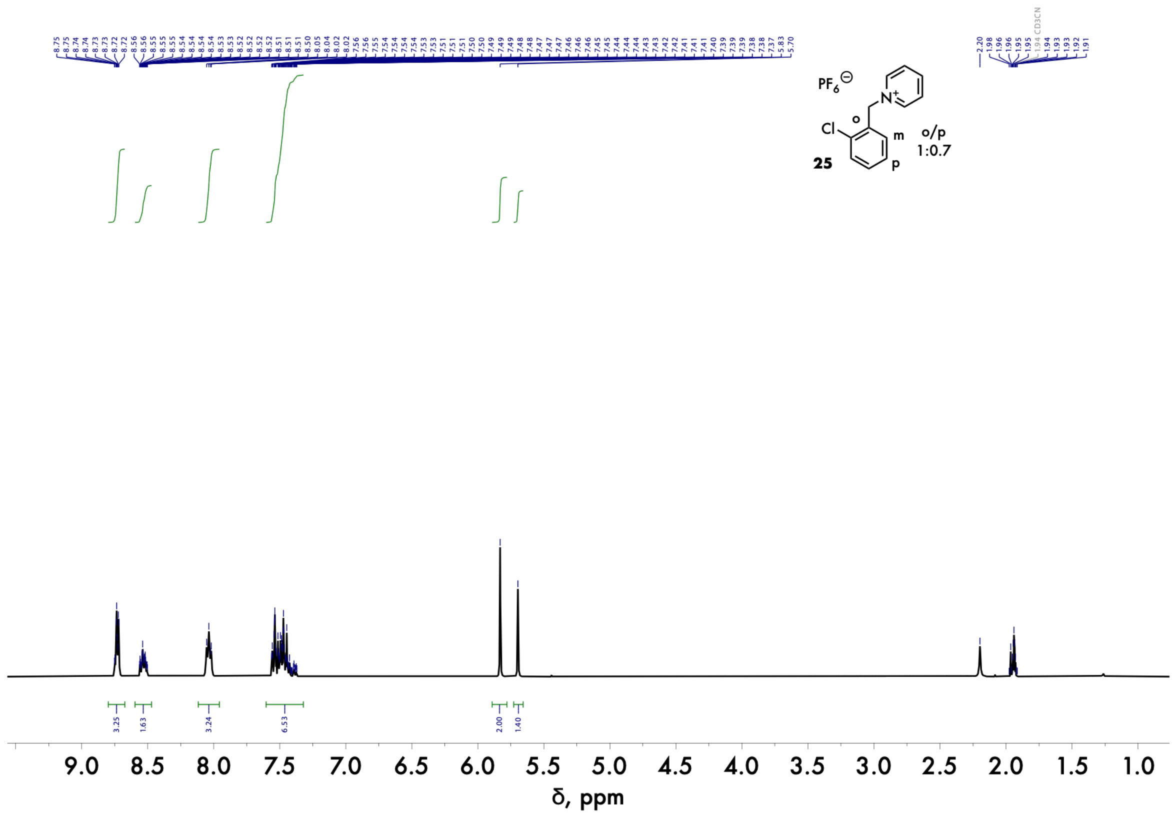

Figure S111. ^{1}H NMR (400 MHz, CD_3CN) spectrum of **25**.

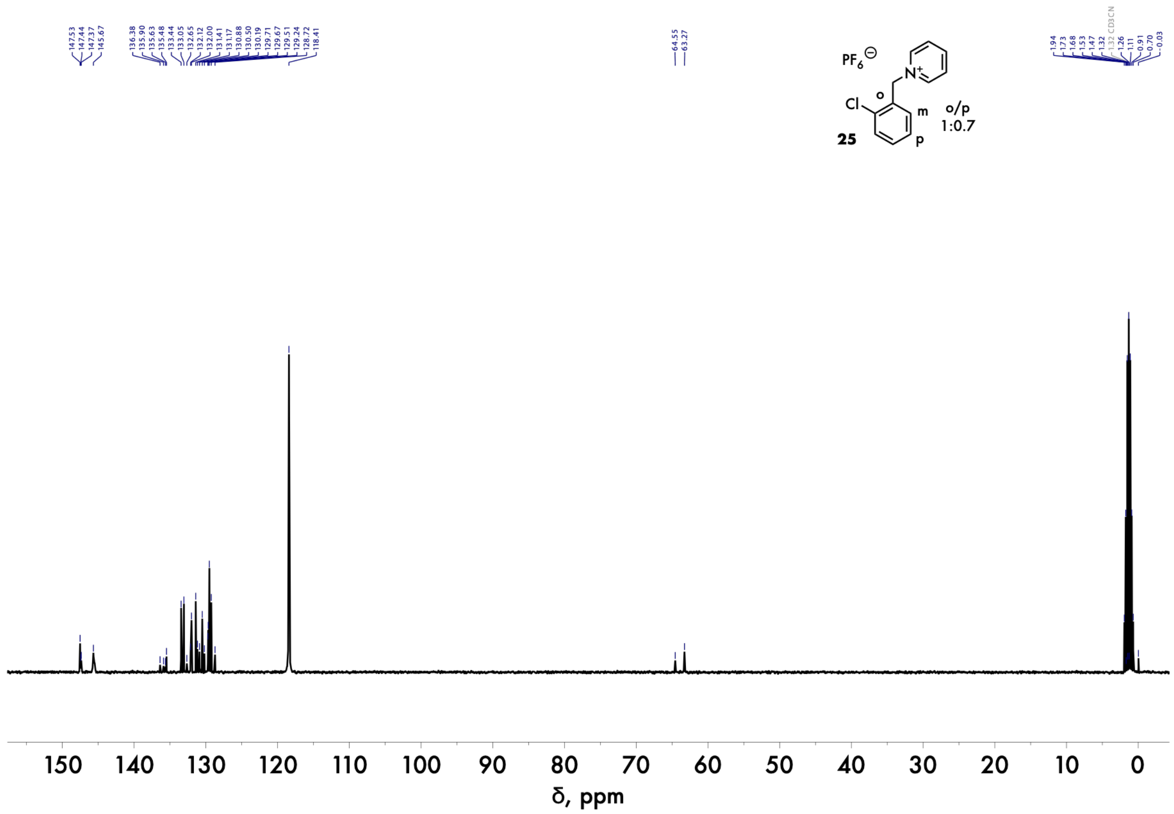

Figure S112. ^{13}C NMR (101 MHz, CD_3CN) spectrum of **25**.

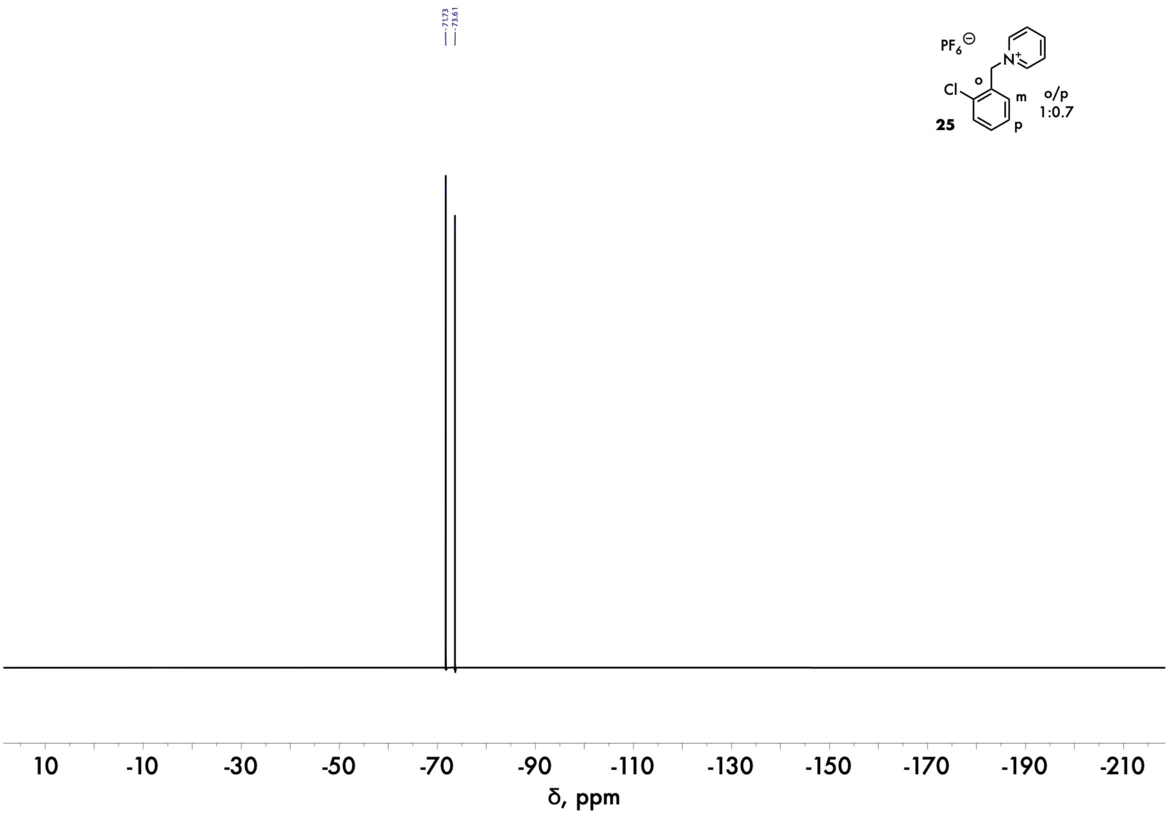

Figure S113. ^{19}F NMR (376 MHz, CD_3CN) spectrum of **25**.

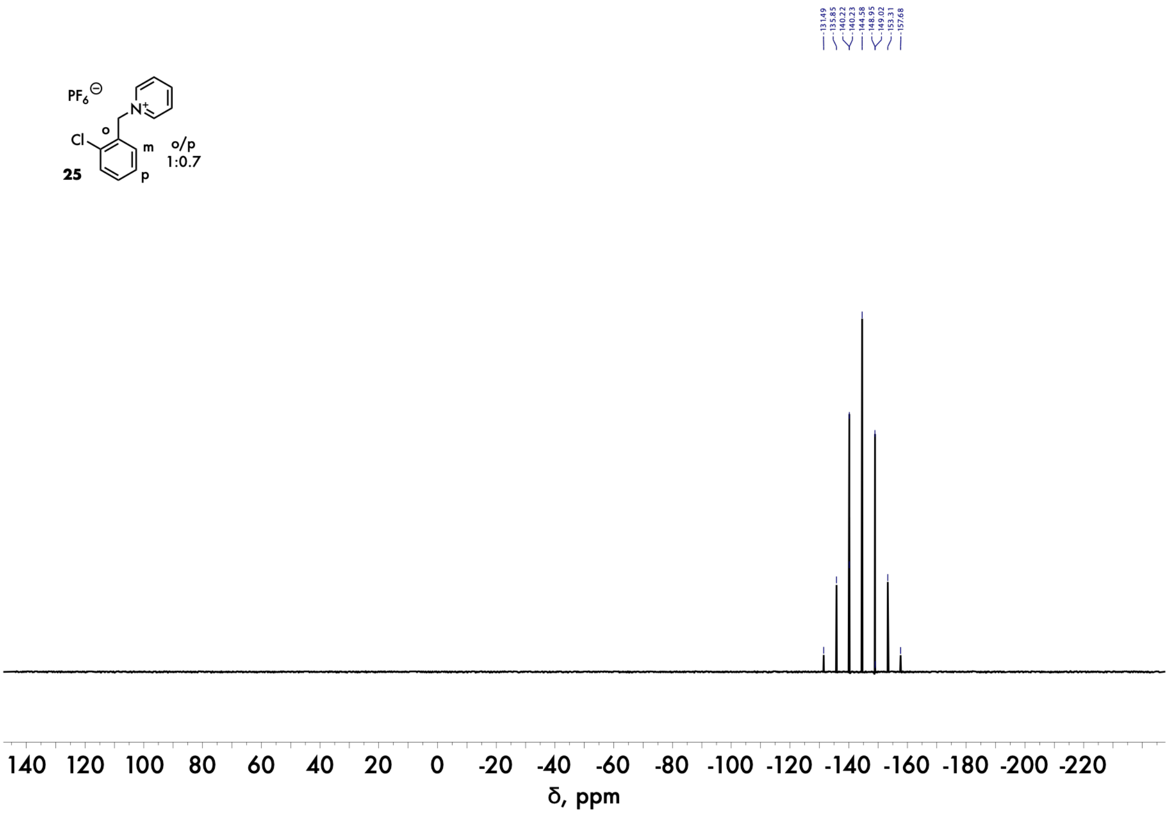

Figure S114. ^{31}P NMR (162 MHz, CD_3CN) spectrum of **25**.

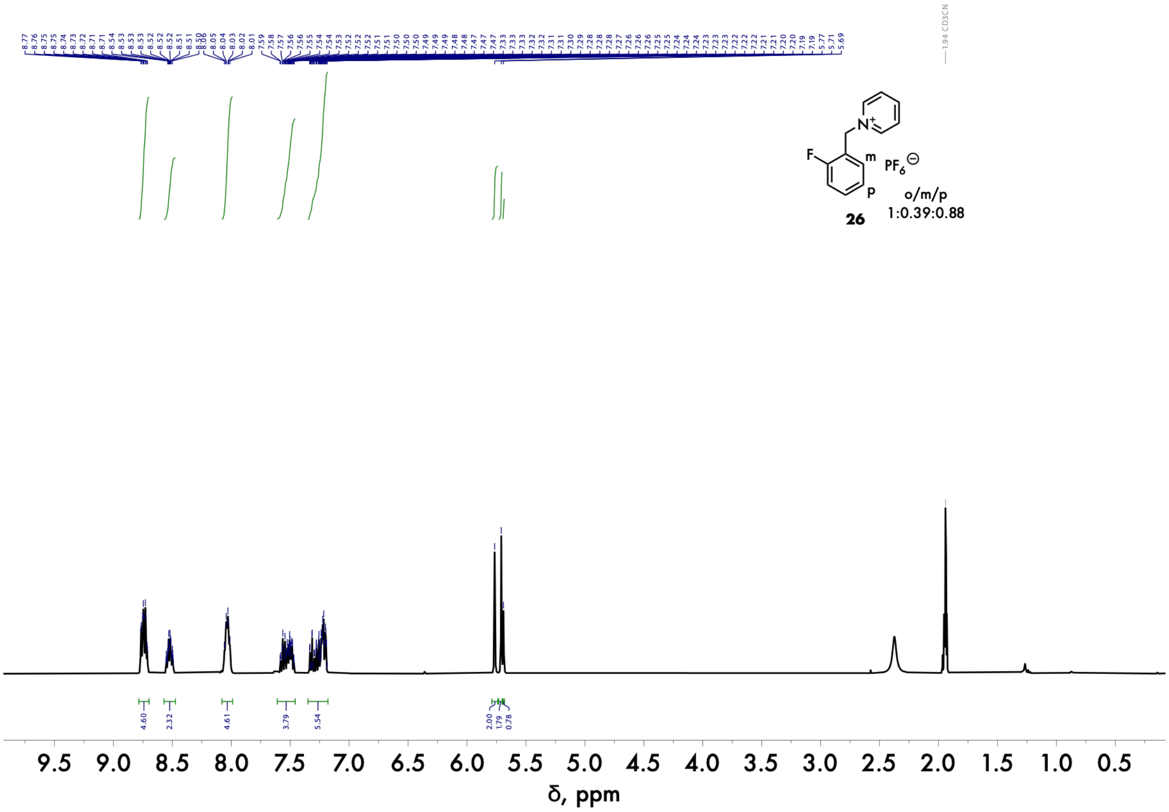

Figure S115. ^{1}H NMR (400 MHz, CD_3CN) spectrum of **26**.

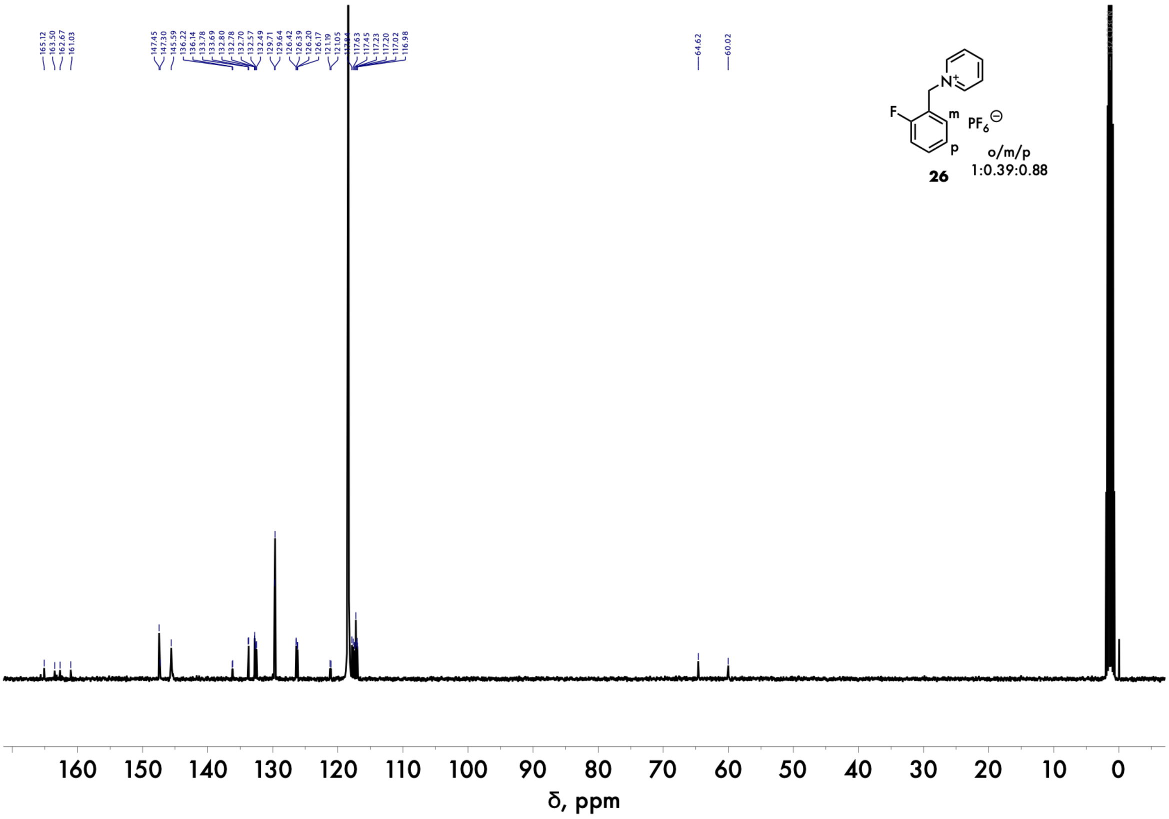

Figure S116. ^{13}C NMR (101 MHz, CD_3CN) spectrum of **26**.

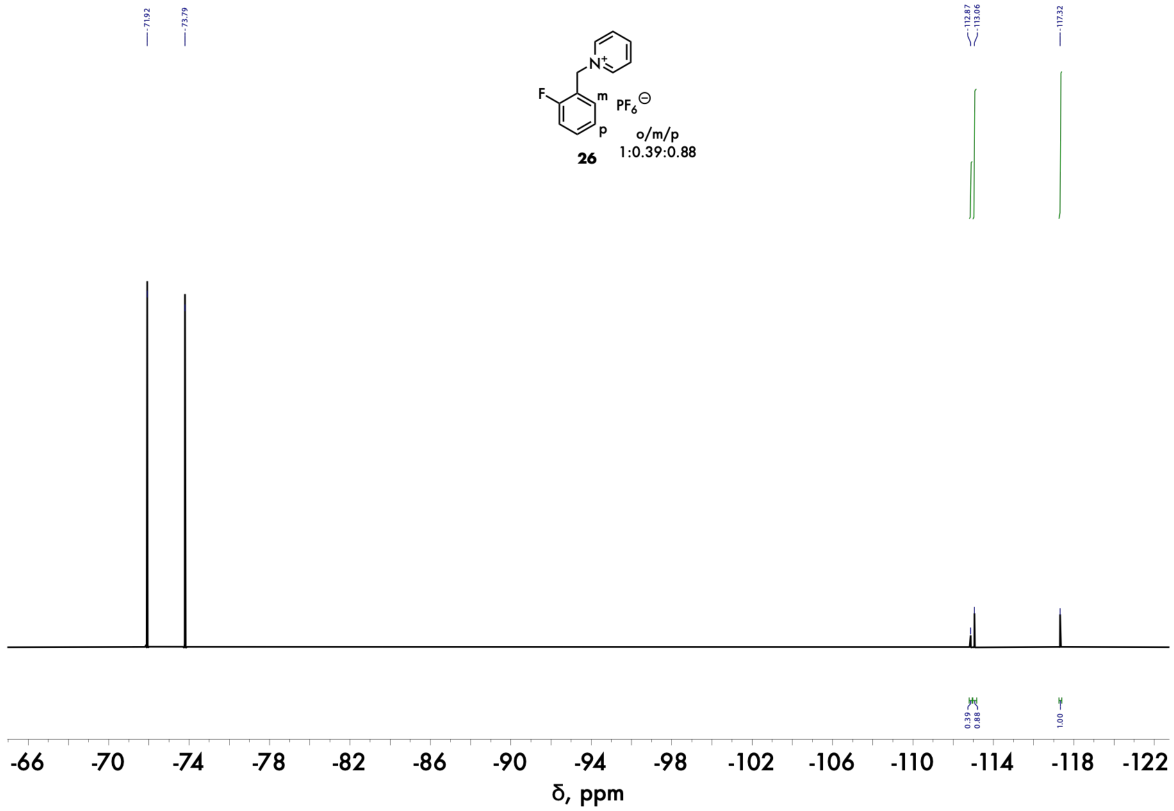

Figure S117. ^{19}F NMR (376 MHz, CD_3CN) spectrum of **26**.

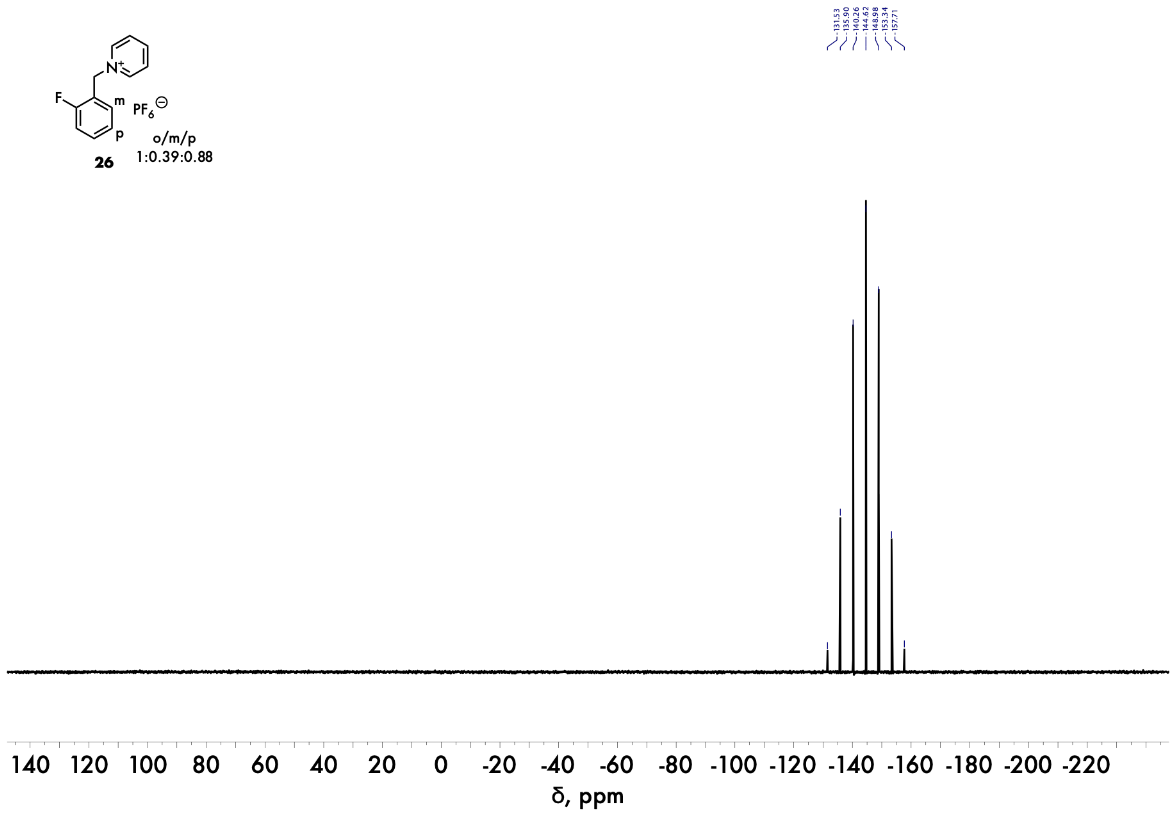

Figure S118. ^{31}P NMR (162 MHz, CD_3CN) spectrum of **26**.

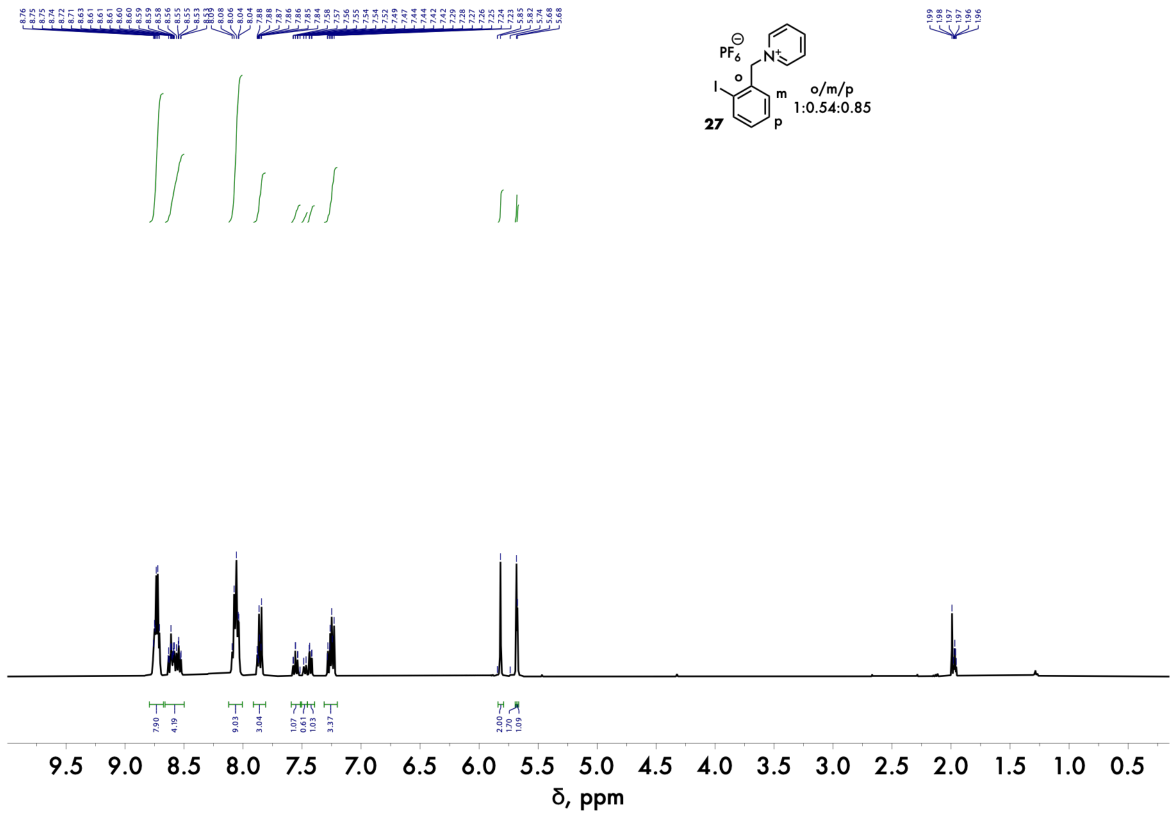

Figure S119. ^{1}H NMR (400 MHz, CD_3CN) spectrum of **27**.

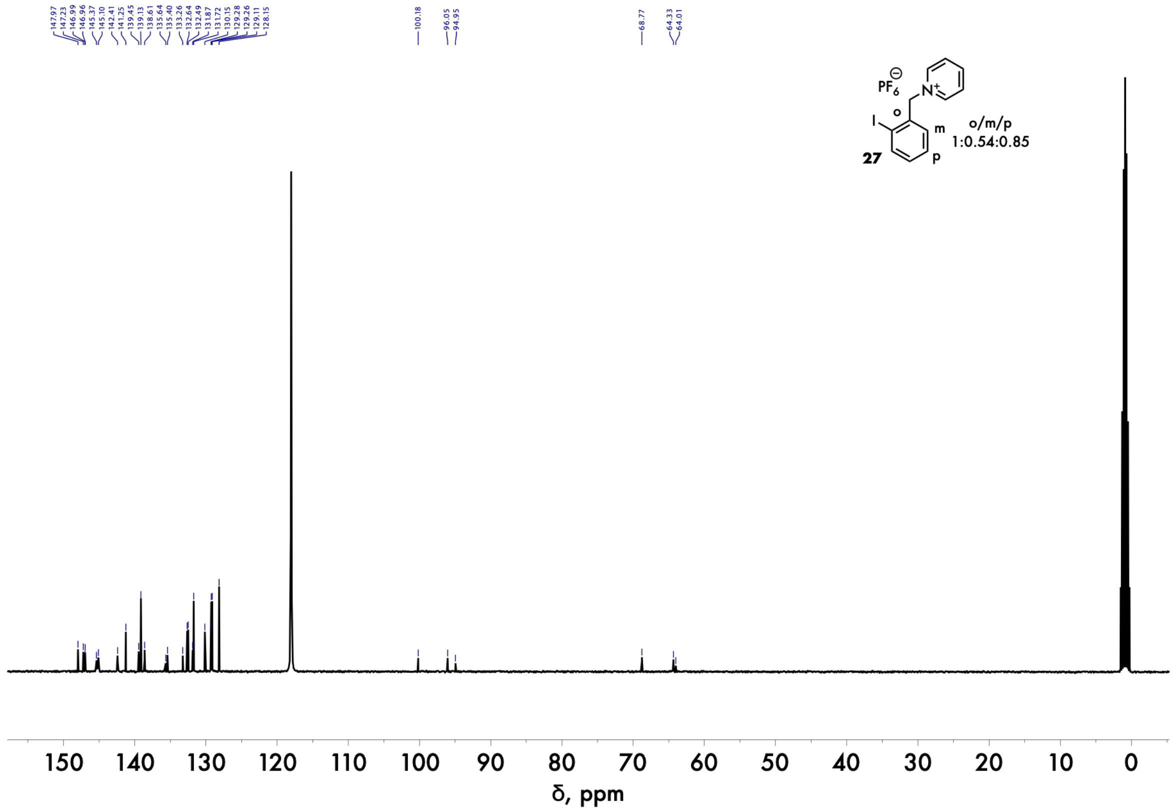

Figure S120. ^{13}C NMR (101 MHz, CD_3CN) spectrum of **27**.

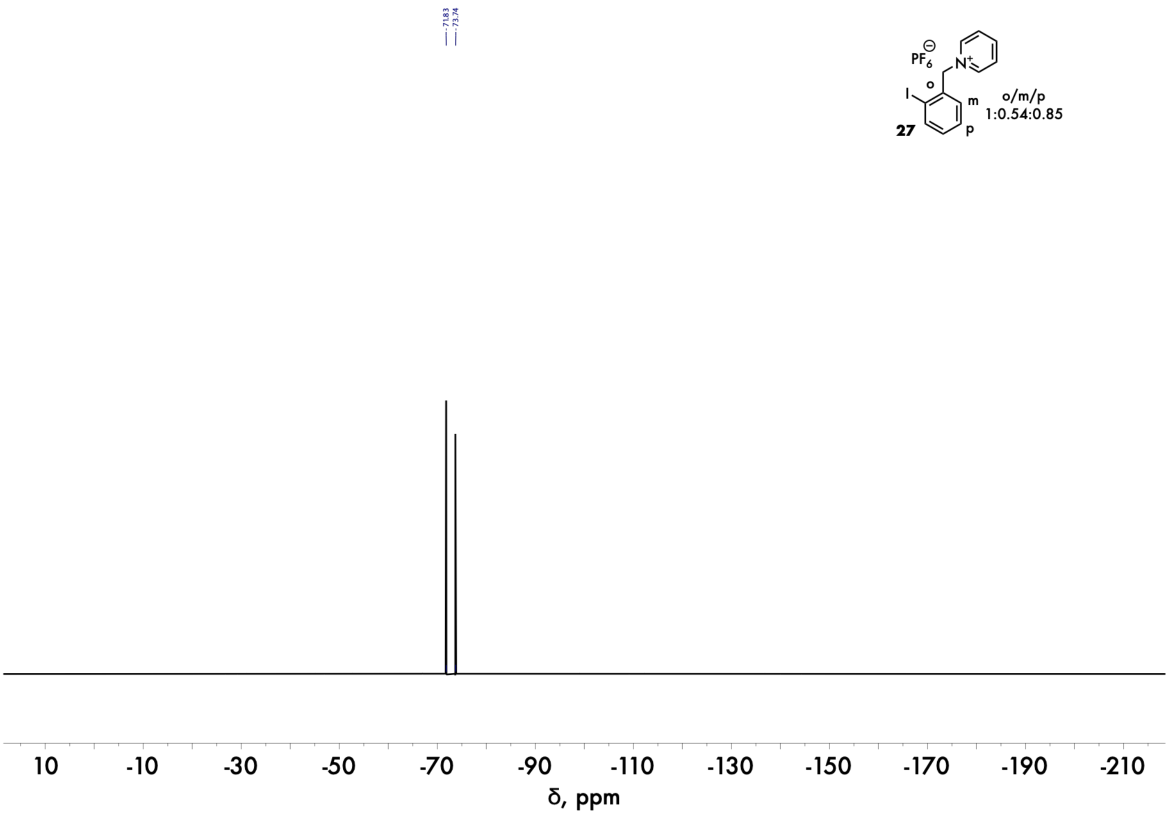

Figure S121. ^{19}F NMR (376 MHz, CD_3CN) spectrum of **27**.

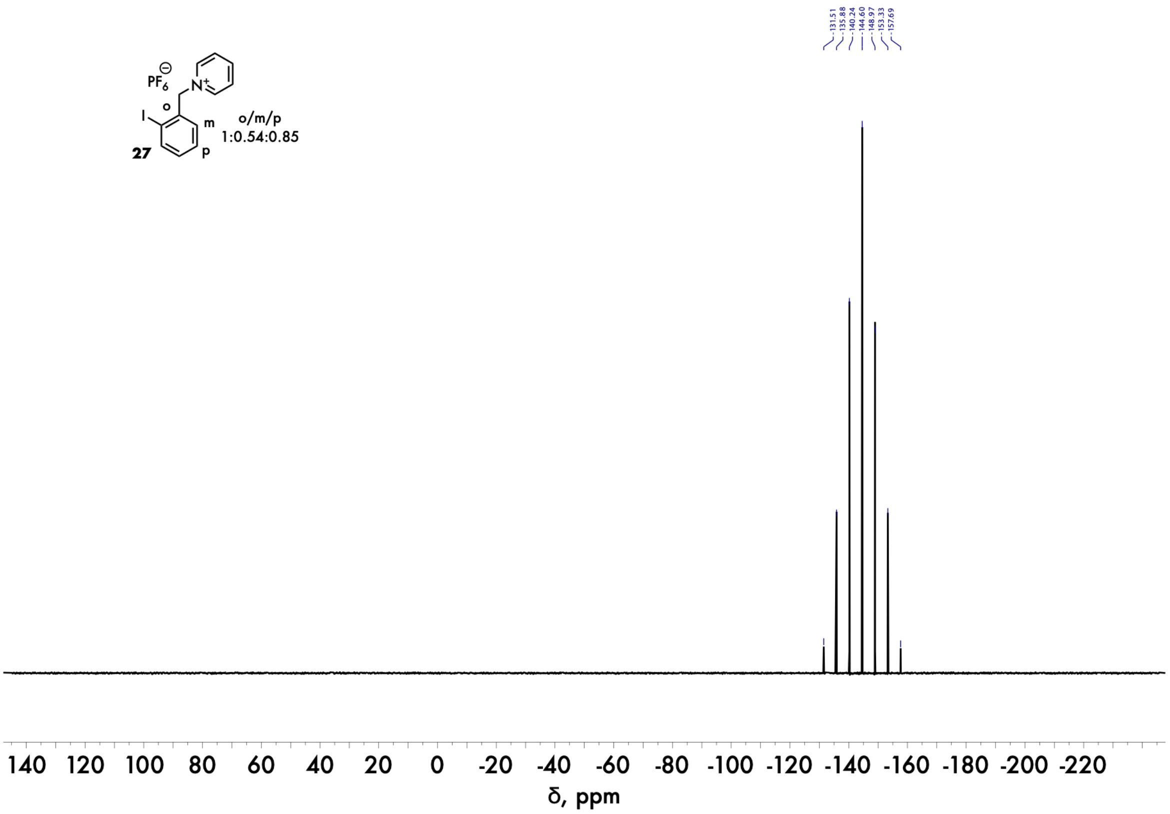

Figure S122. ^{31}P NMR (162 MHz, CD_3CN) spectrum of **27**.

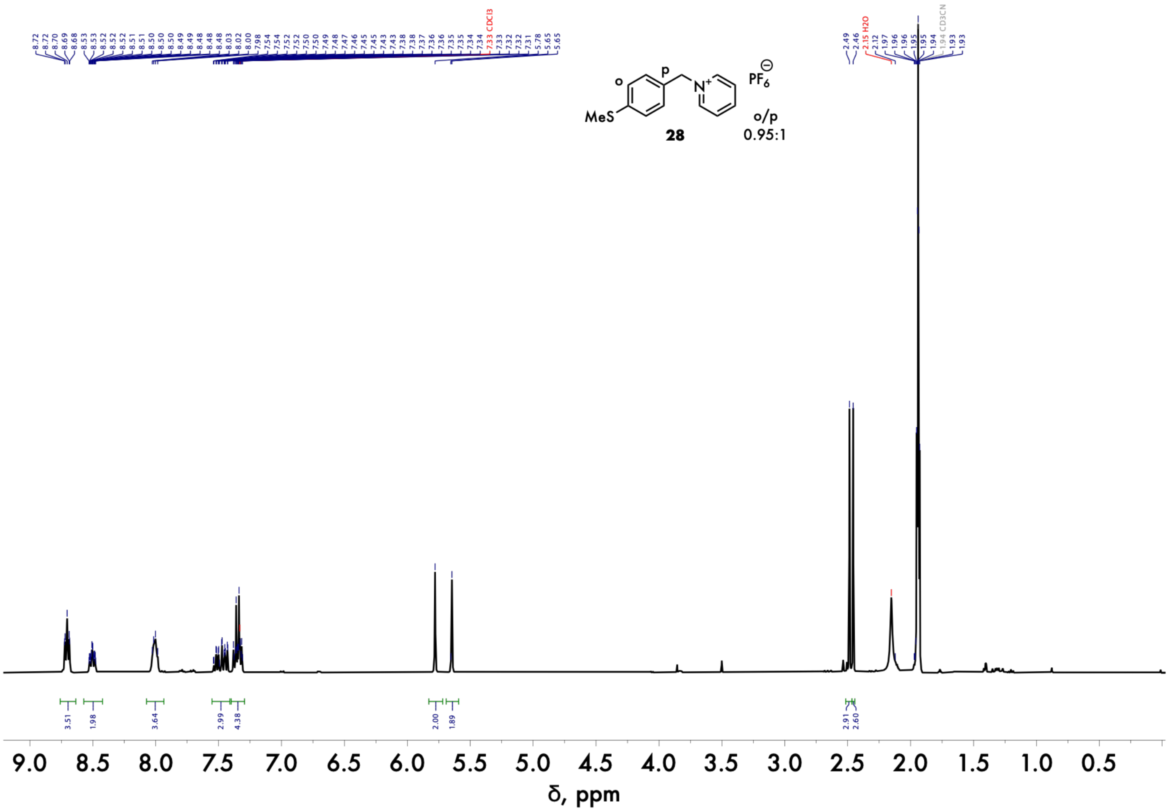

Figure S123. ^1H NMR (400 MHz, CD_3CN) spectrum of **28**.

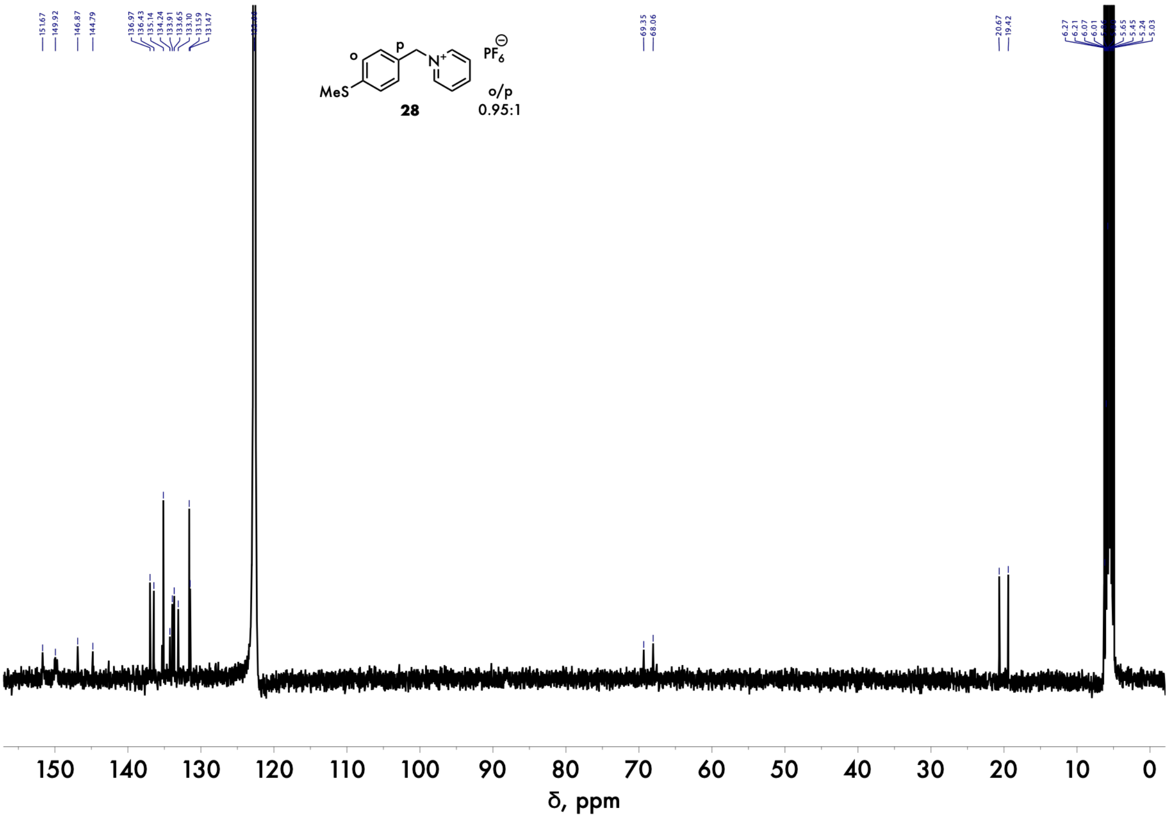

Figure S124. ^{13}C NMR (101 MHz, CD_3CN) spectrum of **28**.

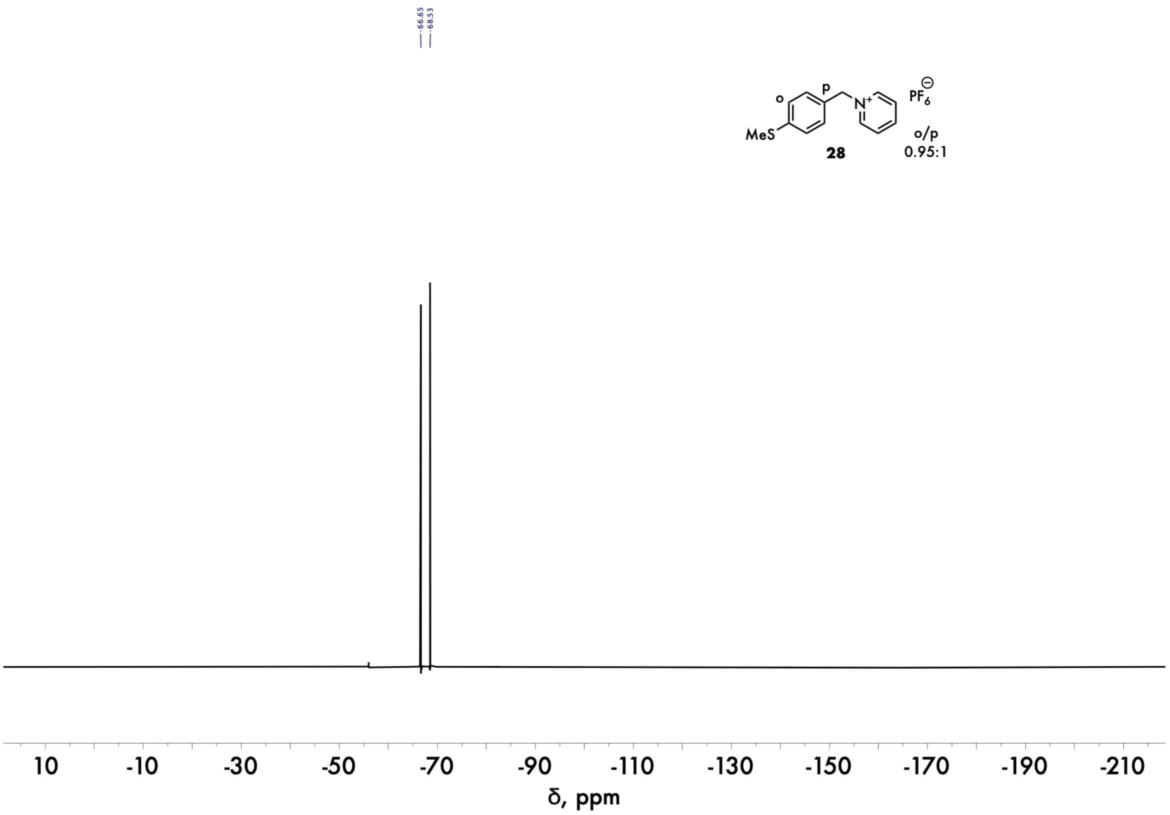

Figure S125. ^{19}F NMR (376 MHz, CD_3CN) spectrum of **28**.

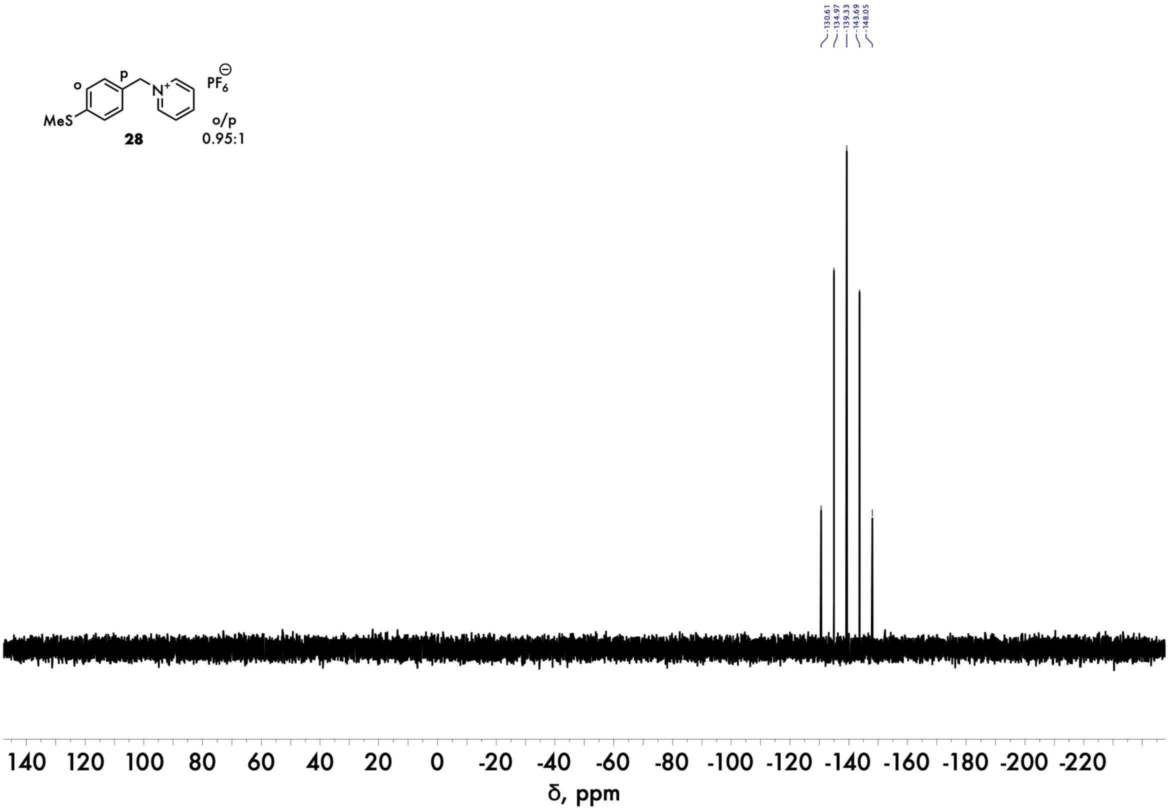

Figure S126. ^{31}P NMR (162 MHz, CD_3CN) spectrum of **28**.

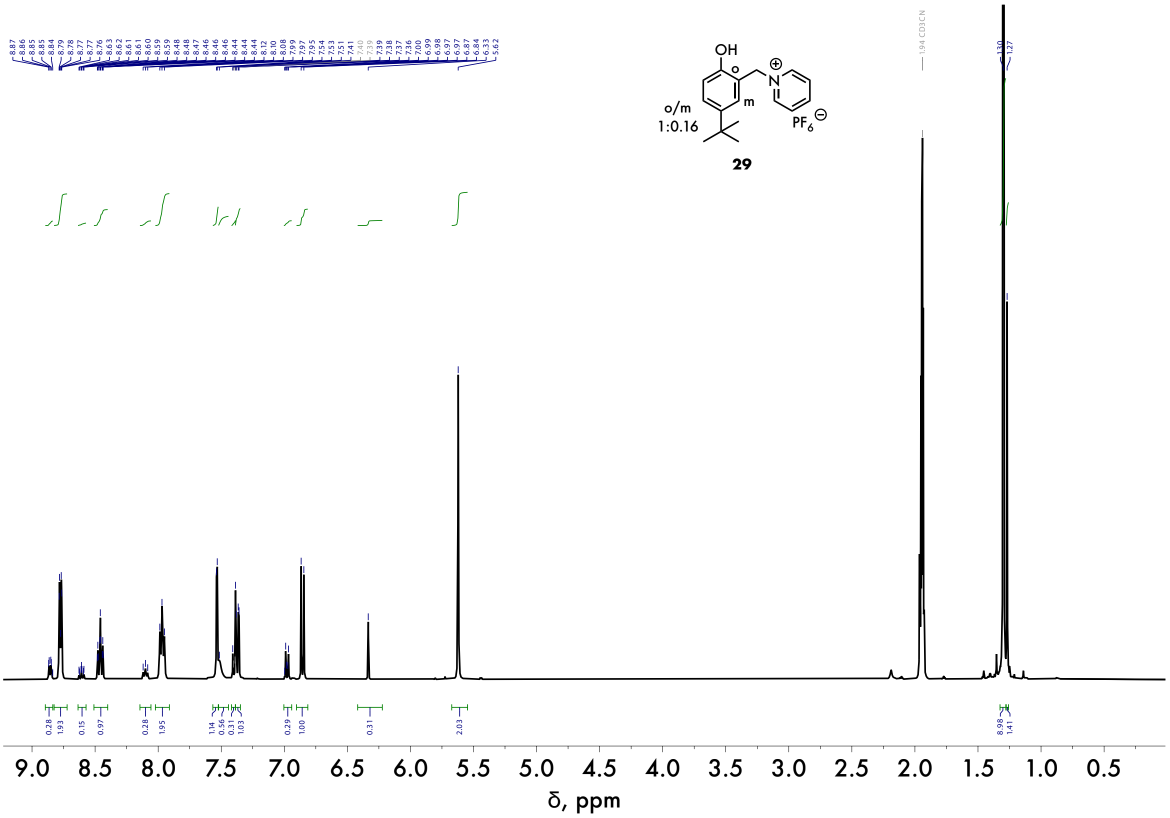

Figure S127. ^{1}H NMR (400 MHz, CD_3CN) spectrum of **29**.

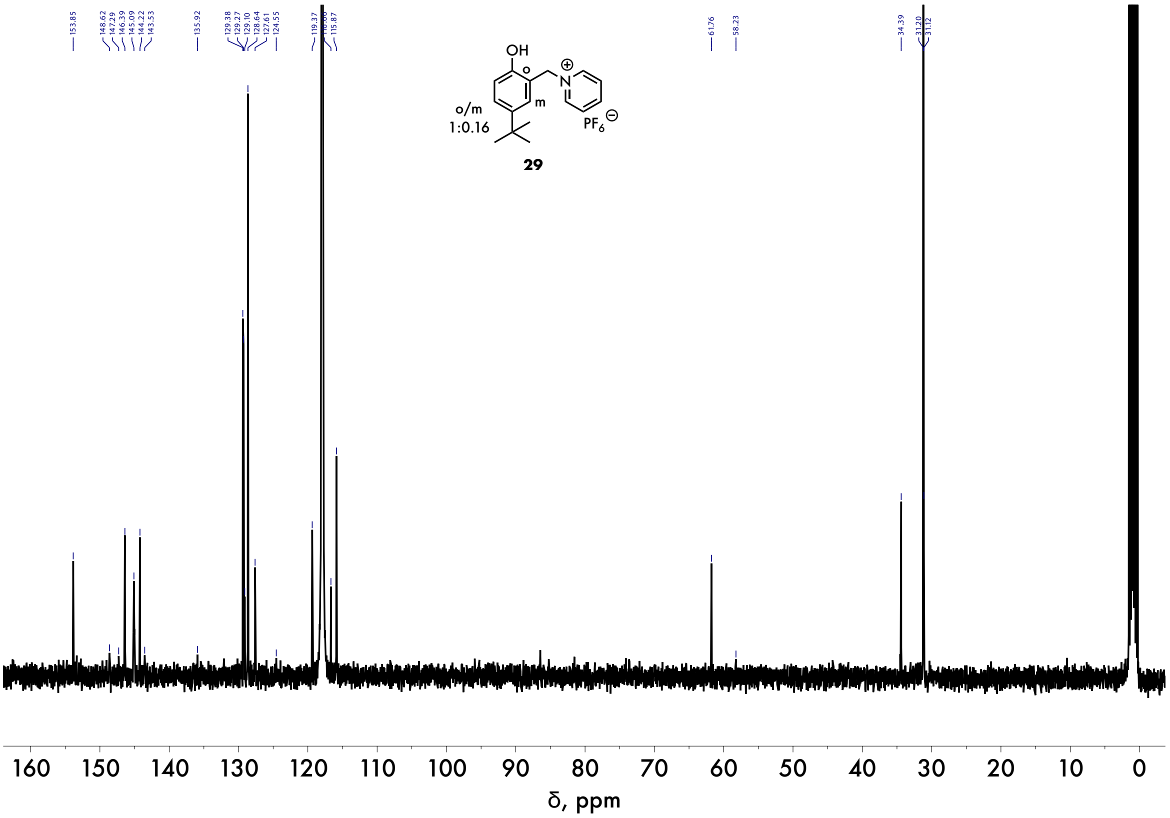

Figure S128. ^{13}C NMR (101 MHz, CD_3CN) spectrum of **29**.

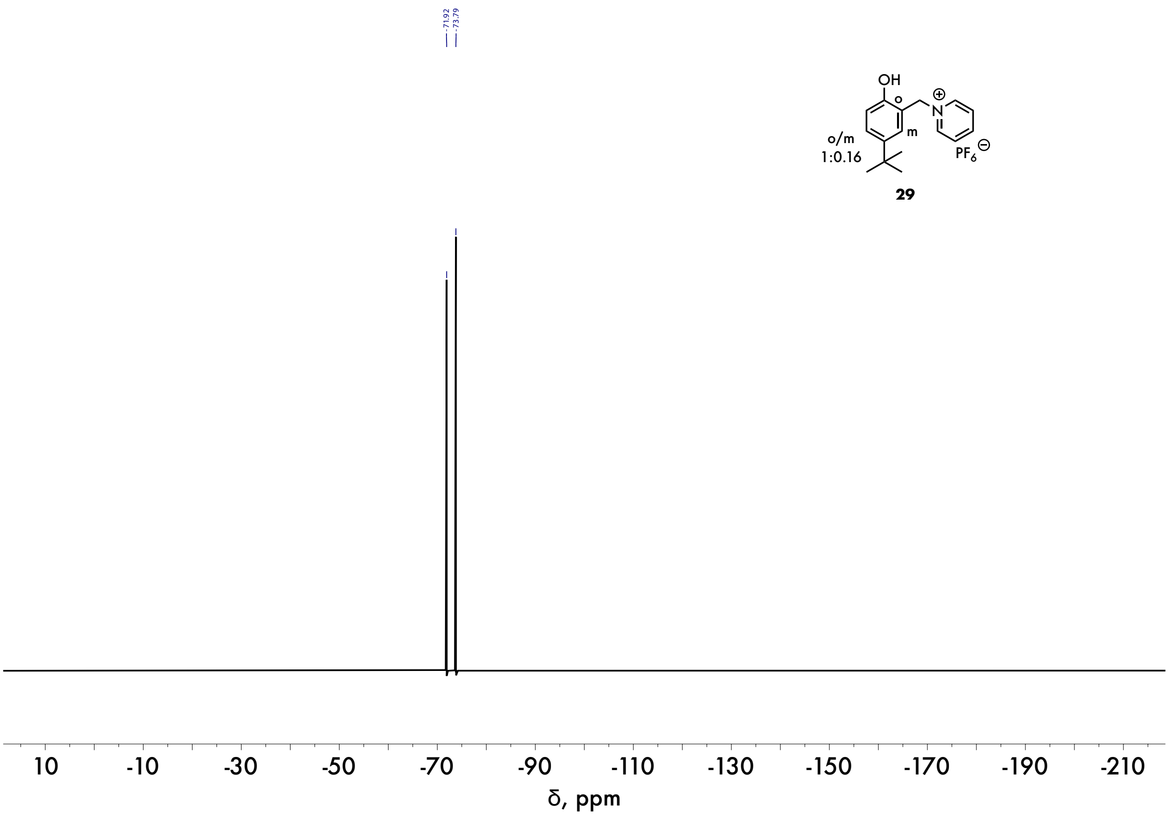

Figure S129. ^{19}F NMR (376 MHz, CD_3CN) spectrum of **29**.

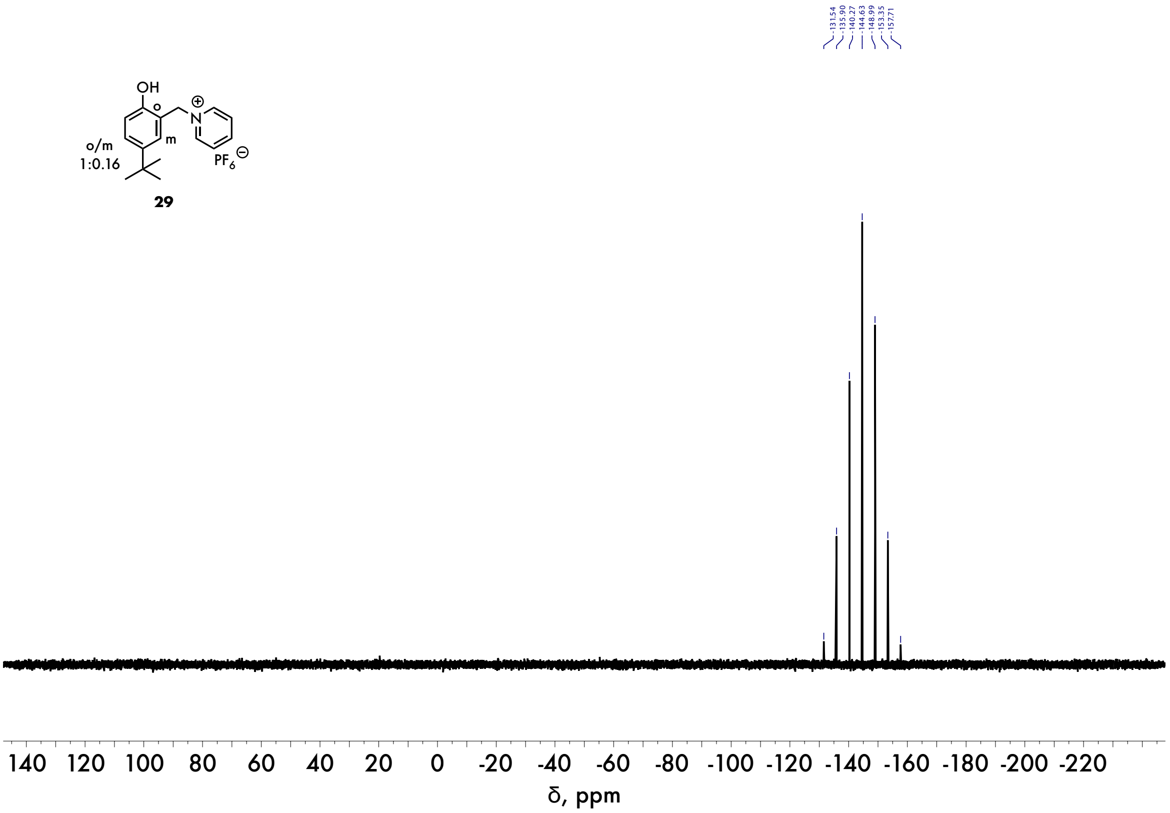

Figure S130. ^{31}P NMR (162 MHz, CD_3CN) spectrum of **29**.

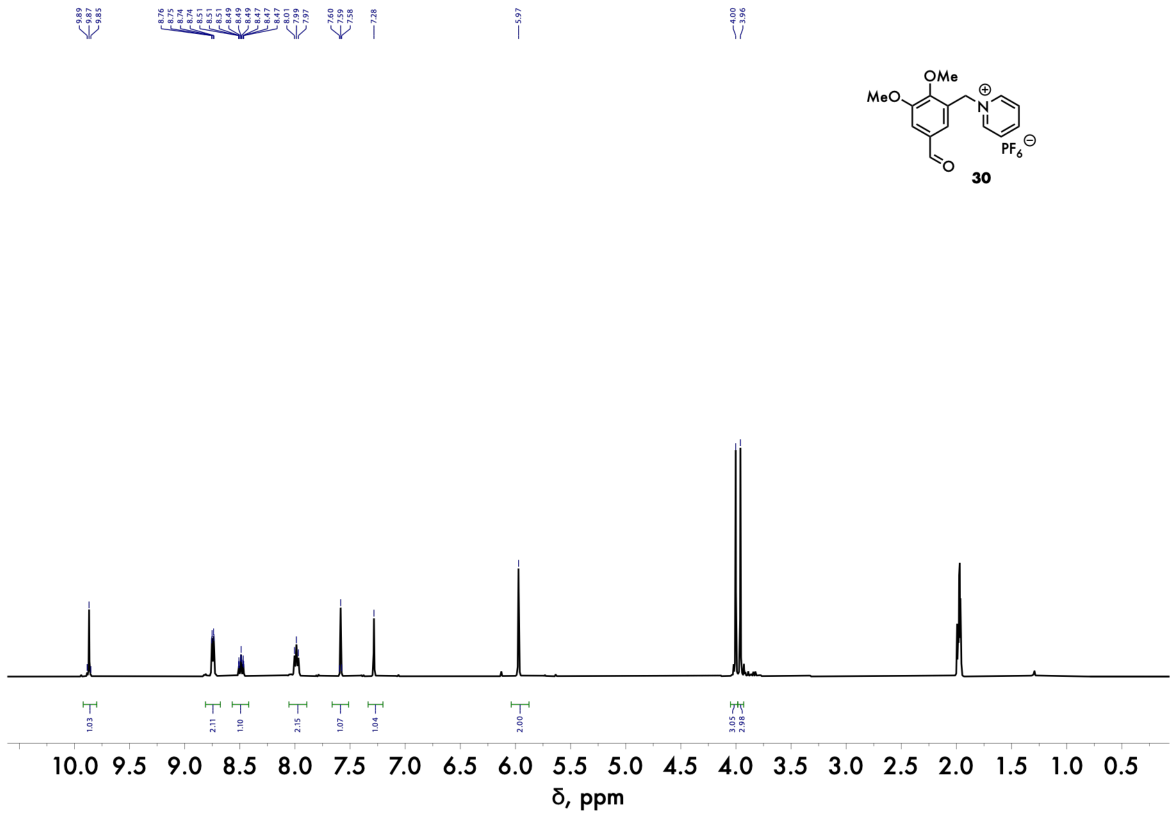

Figure S131. ^{1}H NMR (400 MHz, CD_3CN) spectrum of **30**.

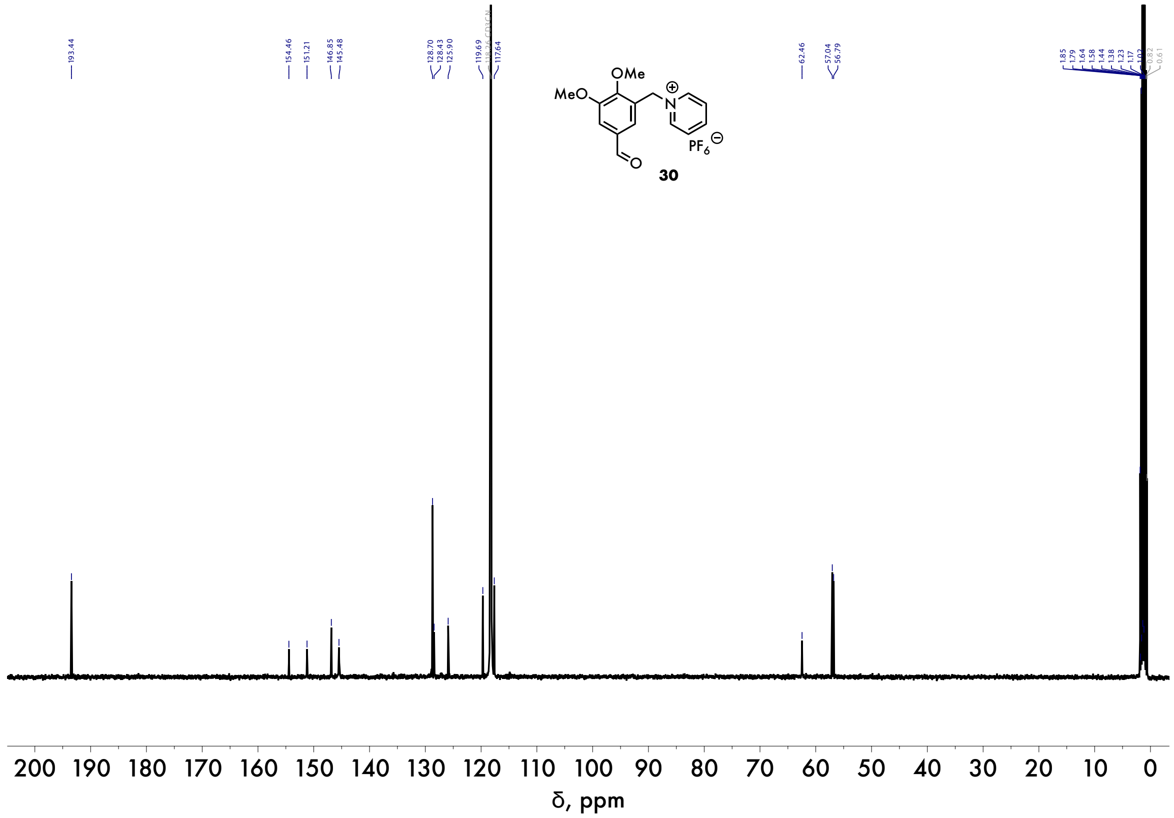

Figure S132. ^{13}C NMR (101 MHz, CD_3CN) spectrum of **30**.

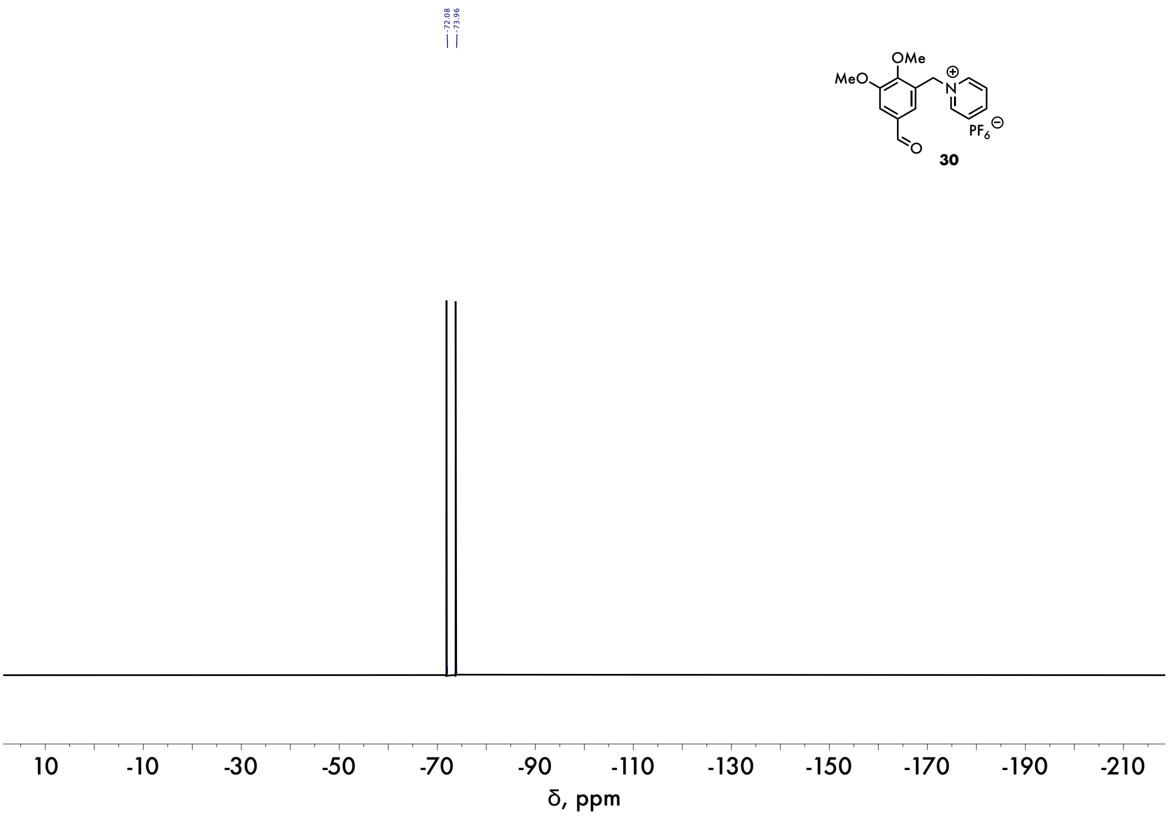

Figure S133. ^{19}F NMR (376 MHz, CD_3CN) spectrum of **30**.

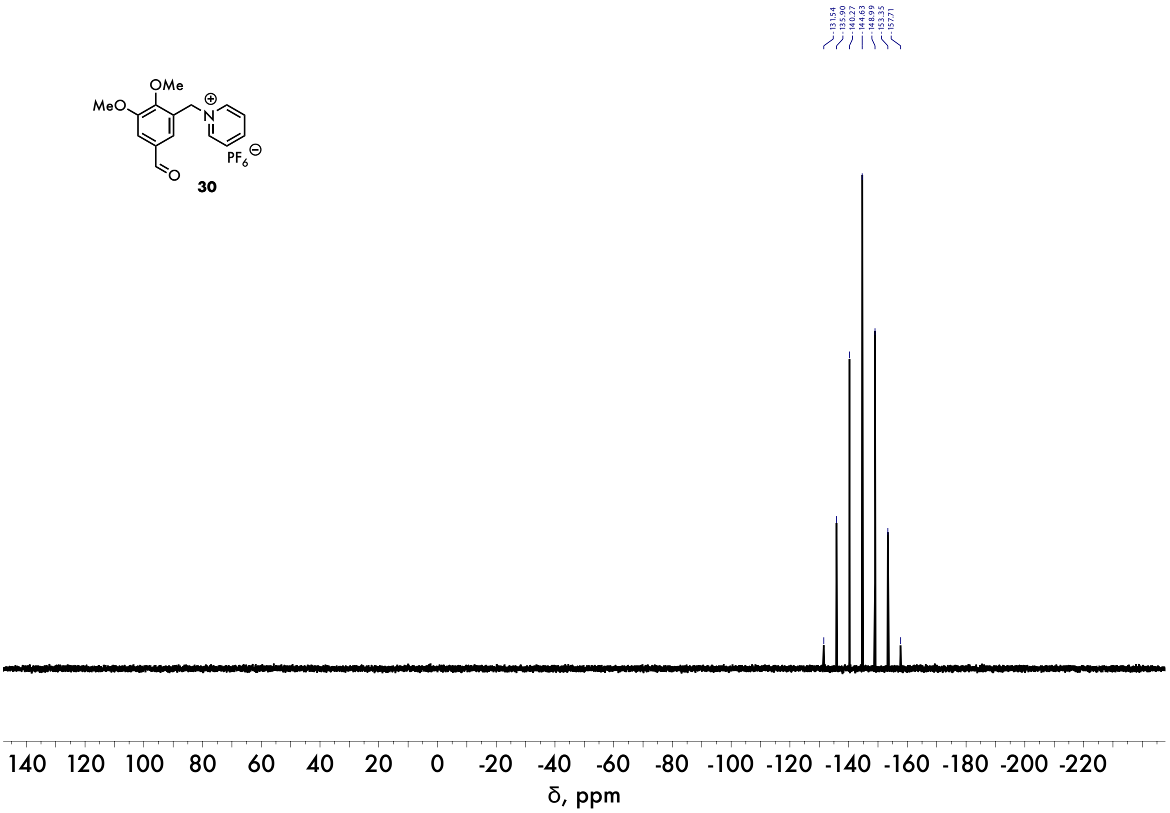

Figure S134. ^{31}P NMR (162 MHz, CD_3CN) spectrum of **30**.

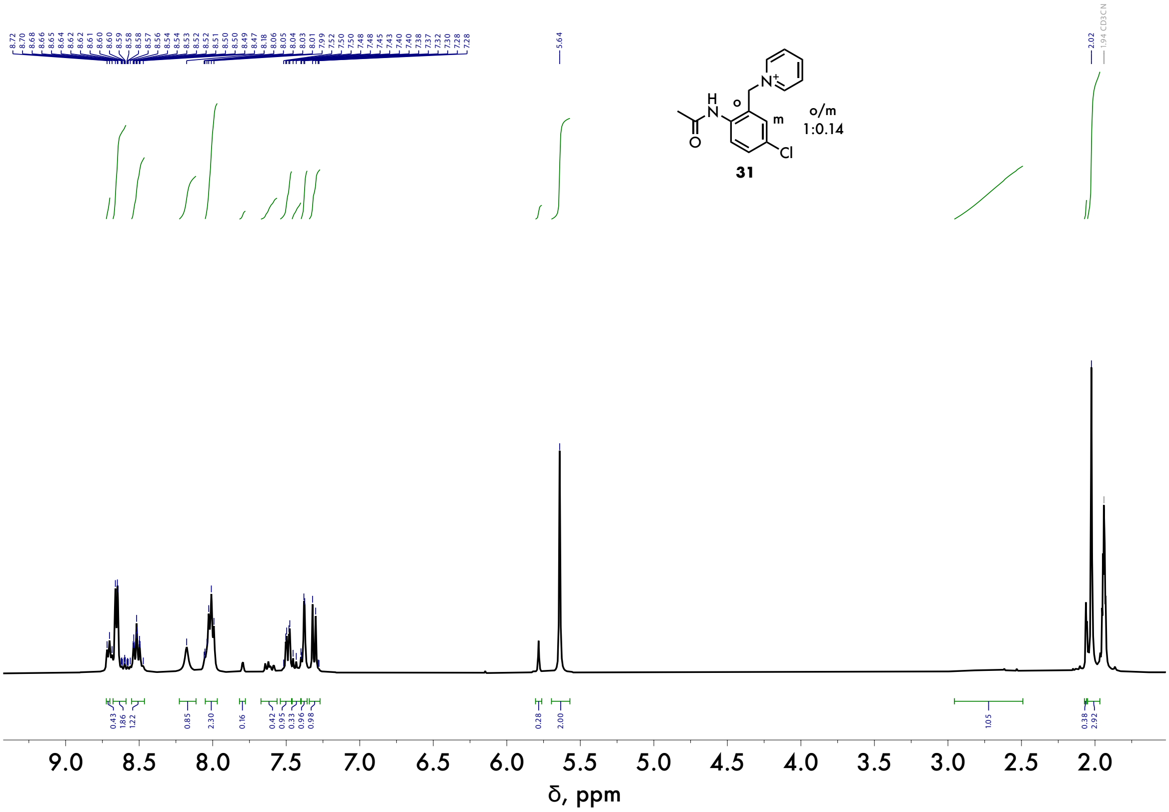

Figure S135. ^{1}H NMR (400 MHz, CD_3CN) spectrum of **31**.

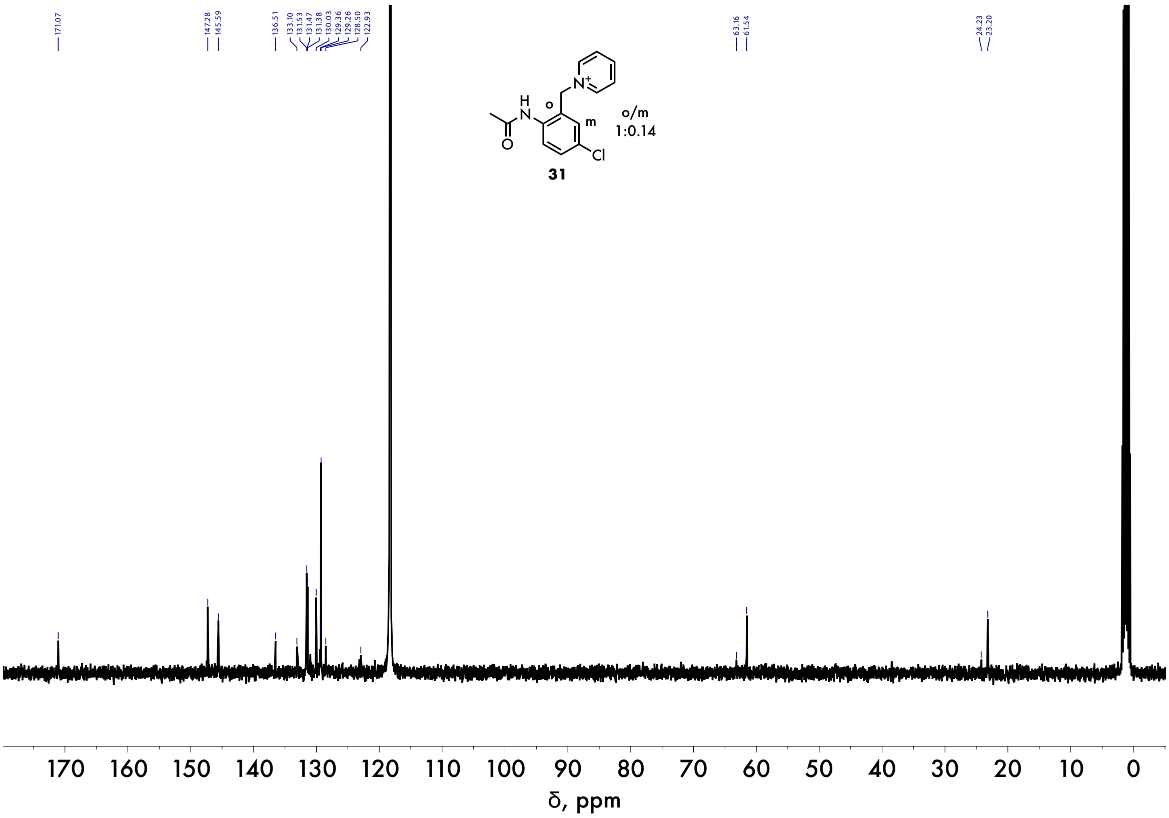

Figure S136. ^{13}C NMR (101 MHz, CD_3CN) spectrum of **31**.

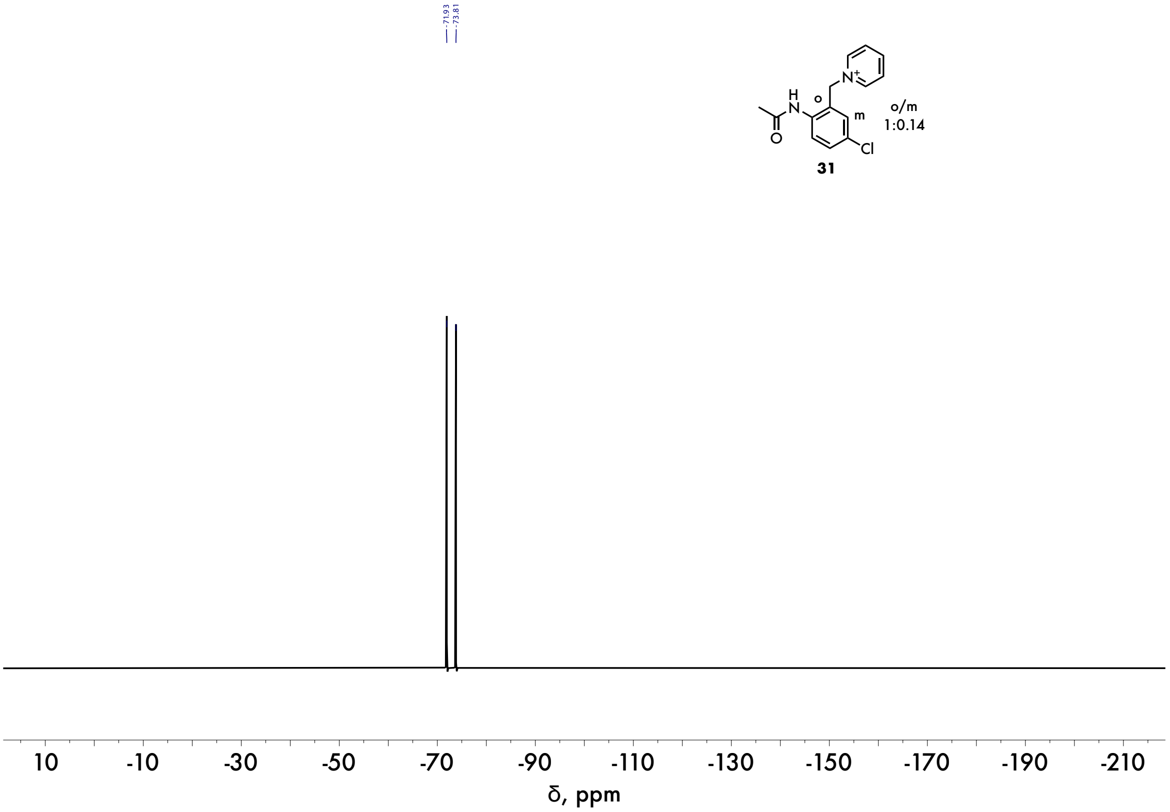

Figure S137. ^{19}F NMR (376 MHz, CD_3CN) spectrum of **31**.

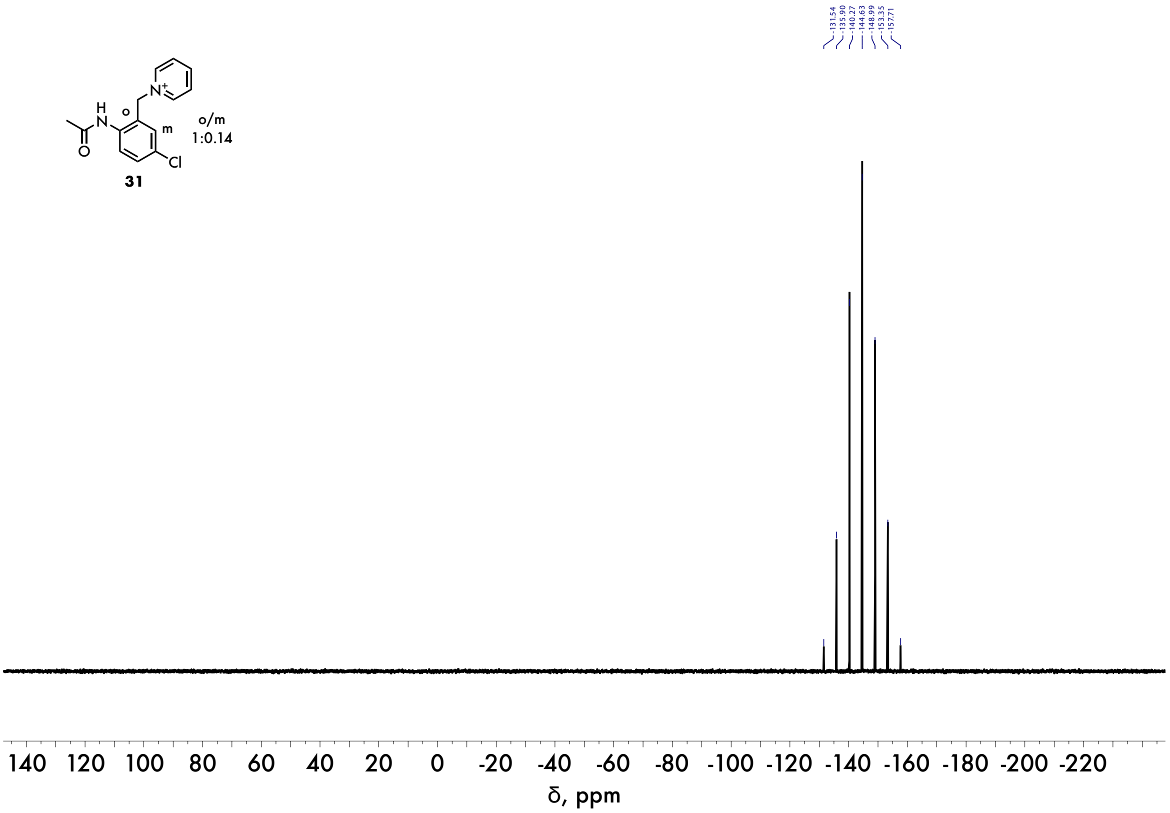

Figure S138. ^{31}P NMR (162 MHz, CD_3CN) spectrum of **31**.

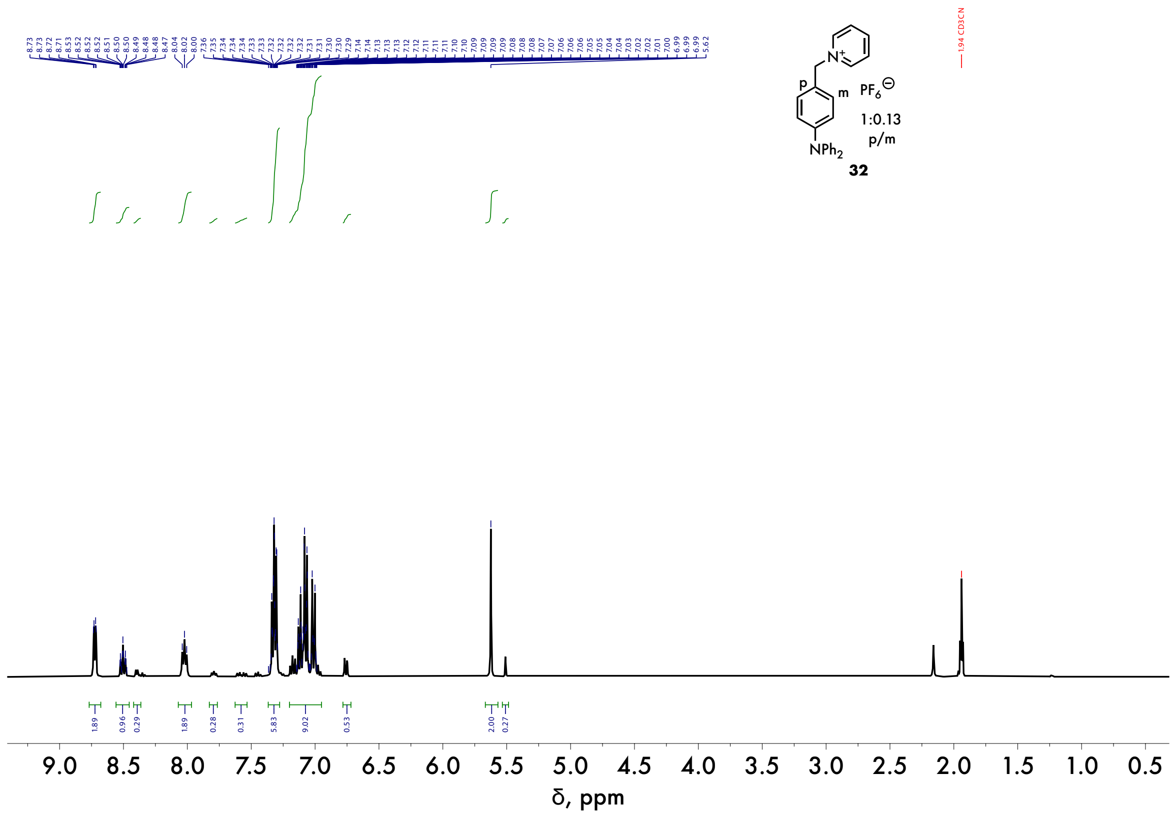

Figure S139. ^{1}H NMR (400 MHz, CD_3CN) spectrum of **32**.

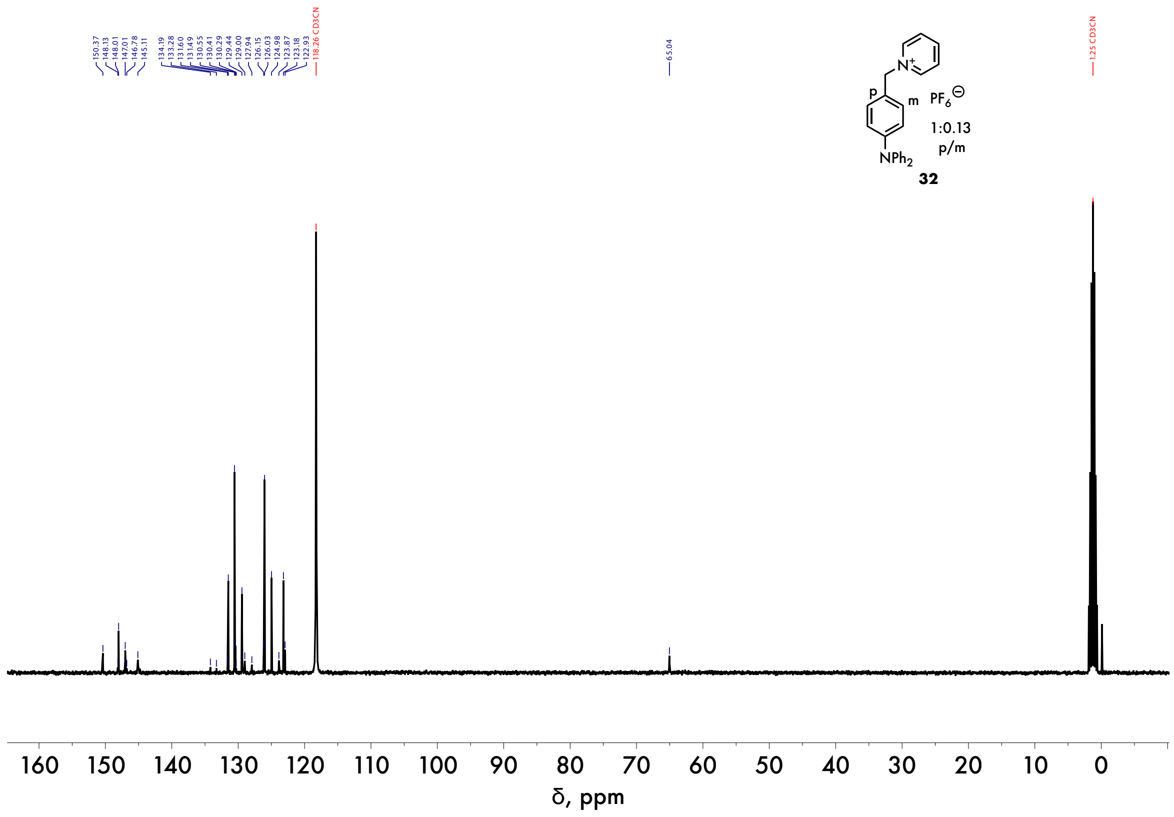

Figure S140. ^{13}C NMR (101 MHz, CD_3CN) spectrum of **32**.

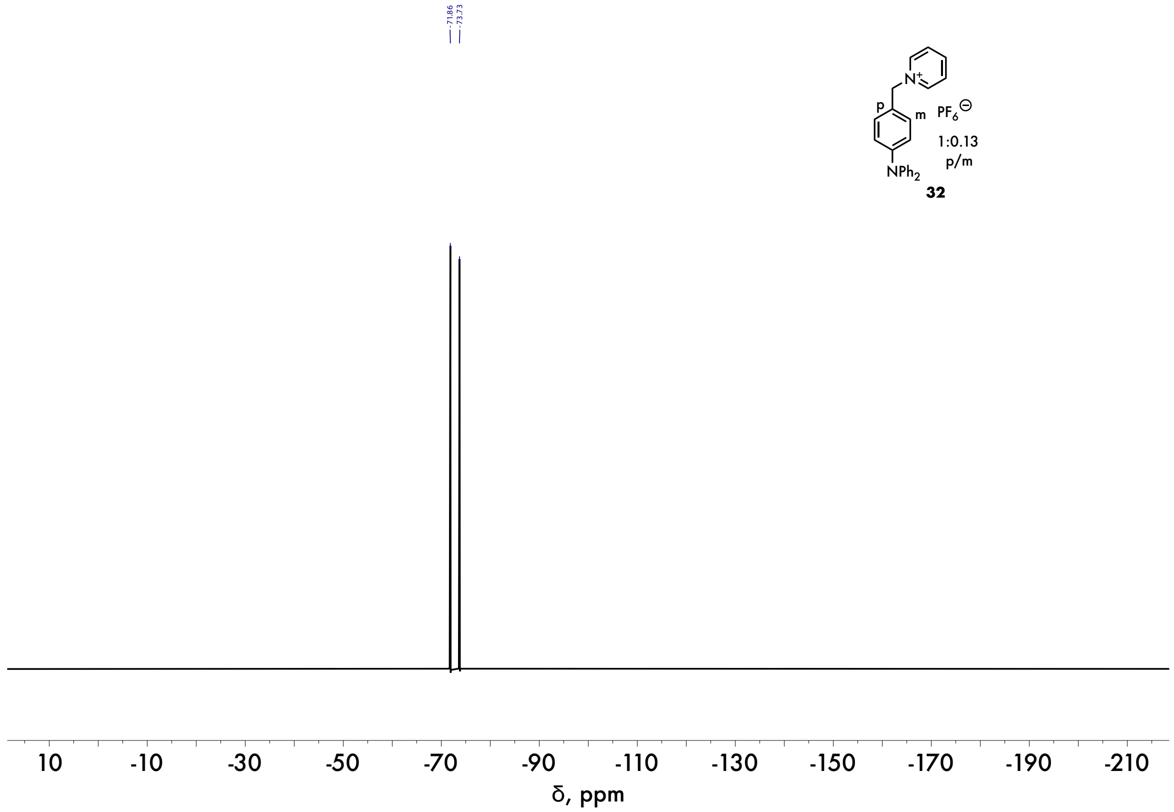

Figure S141. ^{19}F NMR (376 MHz, CD_3CN) spectrum of **32**.

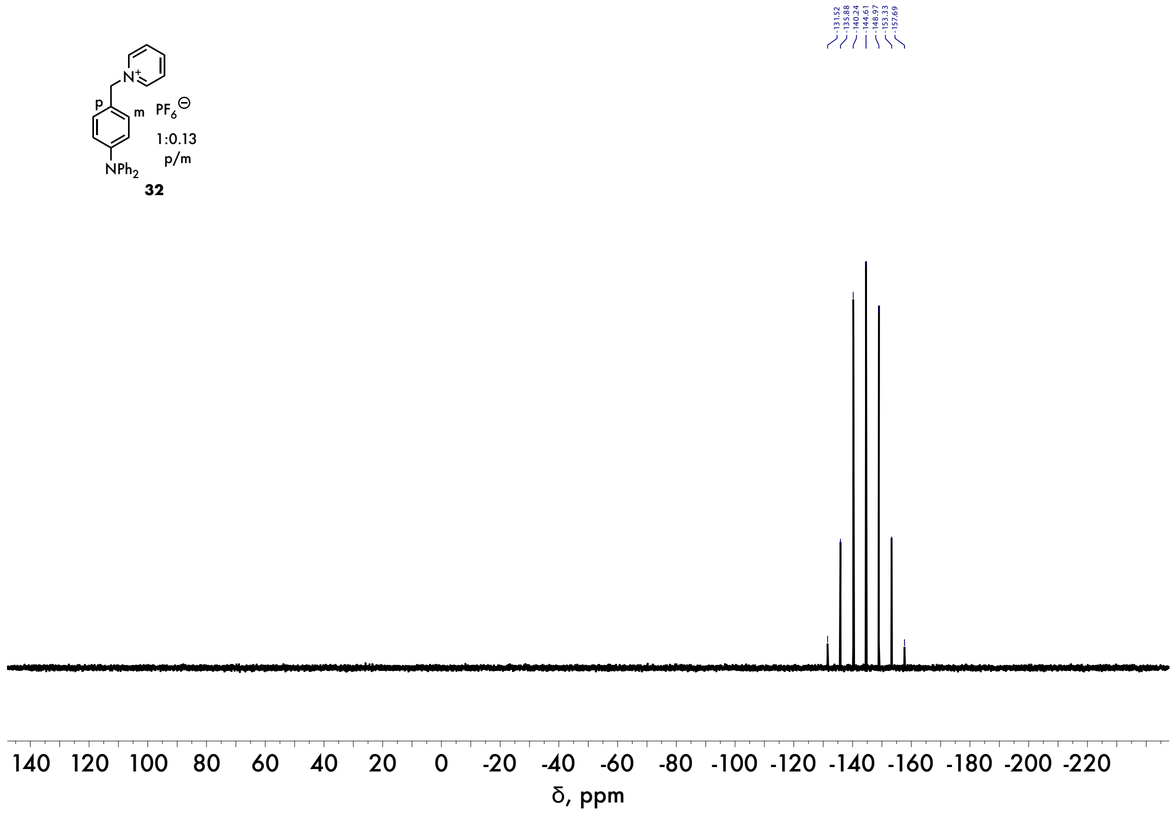

Figure S142. ^{31}P NMR (162 MHz, CD_3CN) spectrum of **32**.

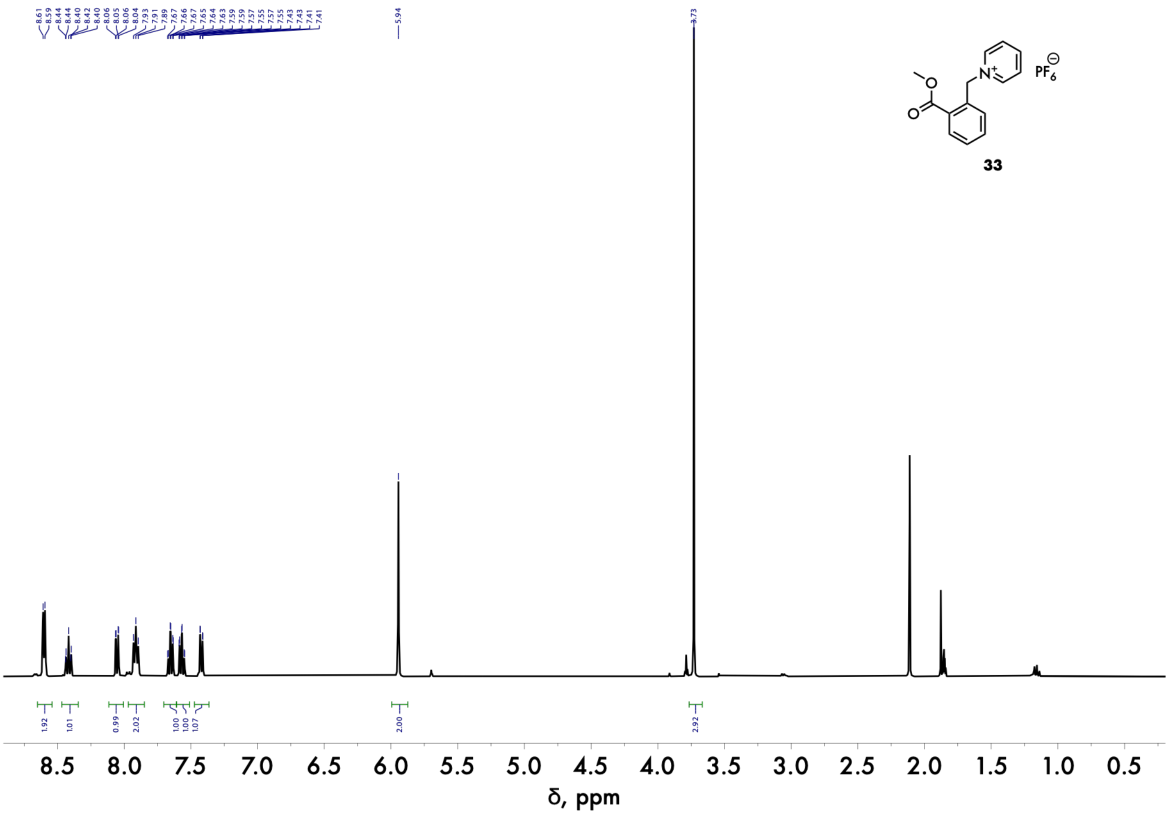

Figure S143. ^{1}H NMR (400 MHz, CD_3CN) spectrum of **33** (o-isomer).

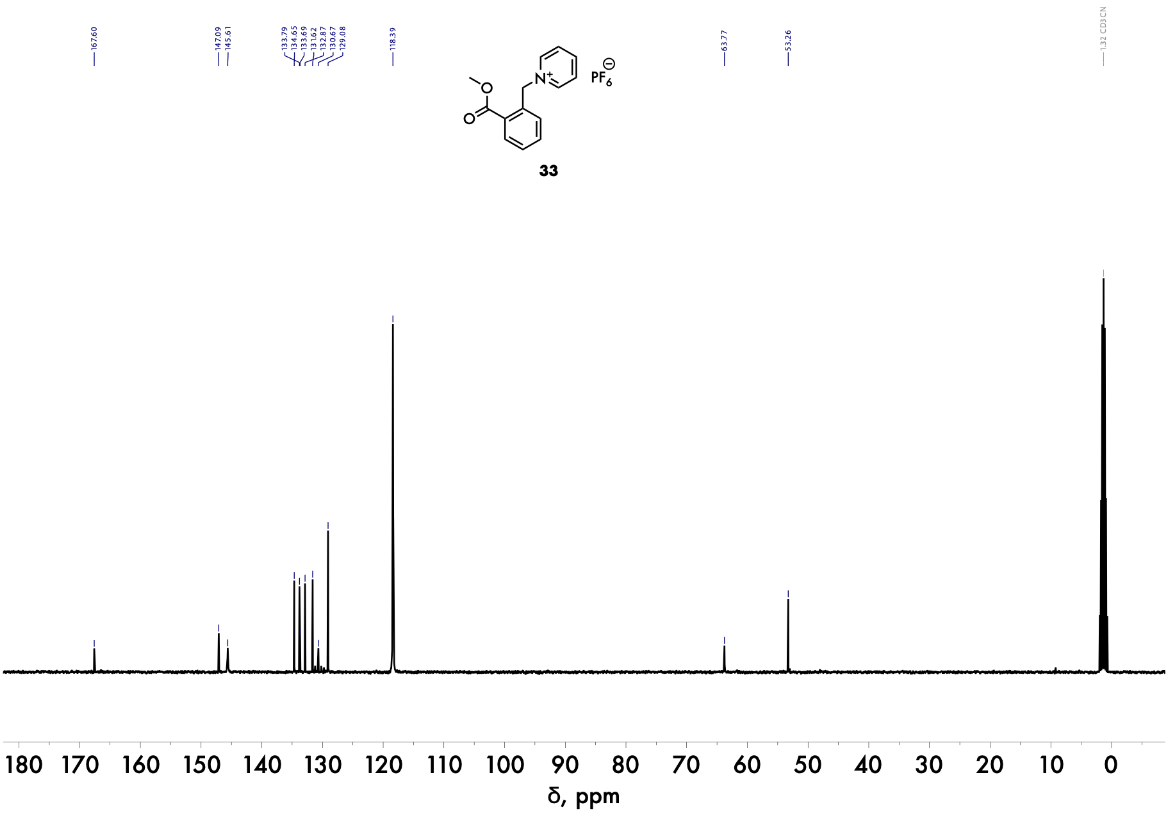

Figure S144. ^{13}C NMR (101 MHz, CD_3CN) spectrum of **33** (o-isomer).

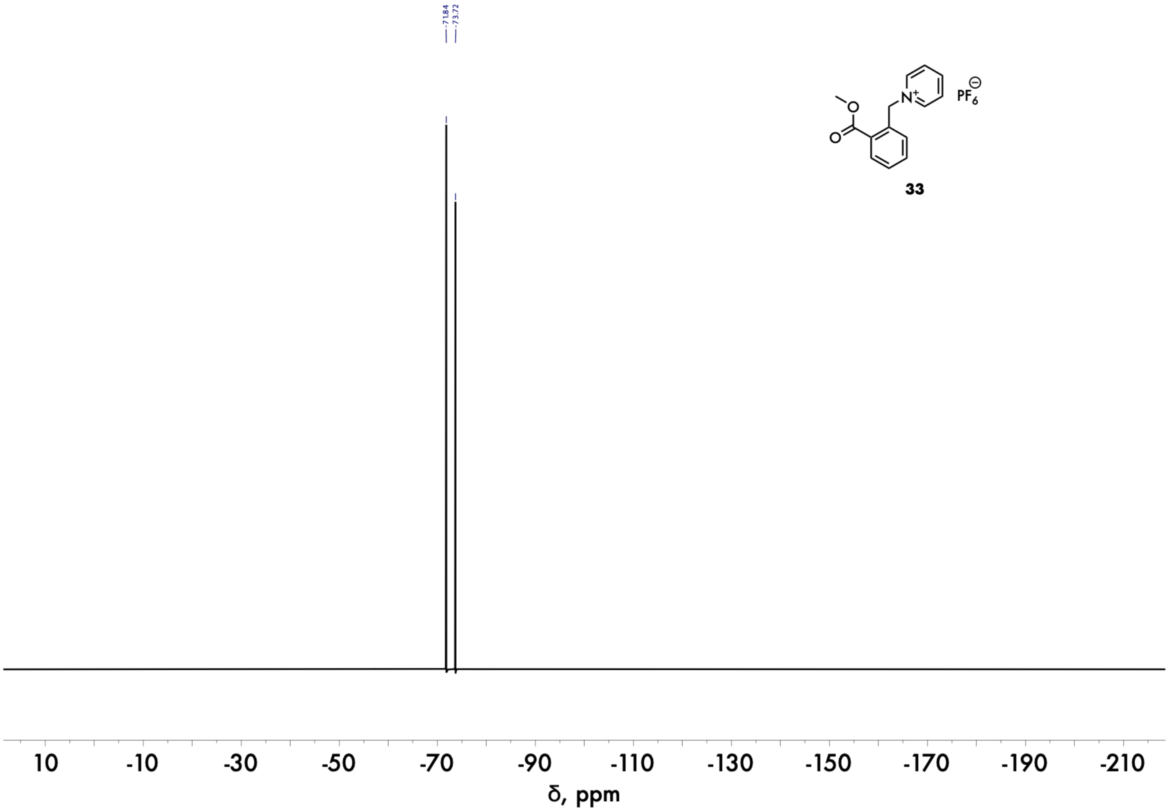

Figure S145. ^{19}F NMR (376 MHz, CD_3CN) spectrum of **33** (o-isomer).

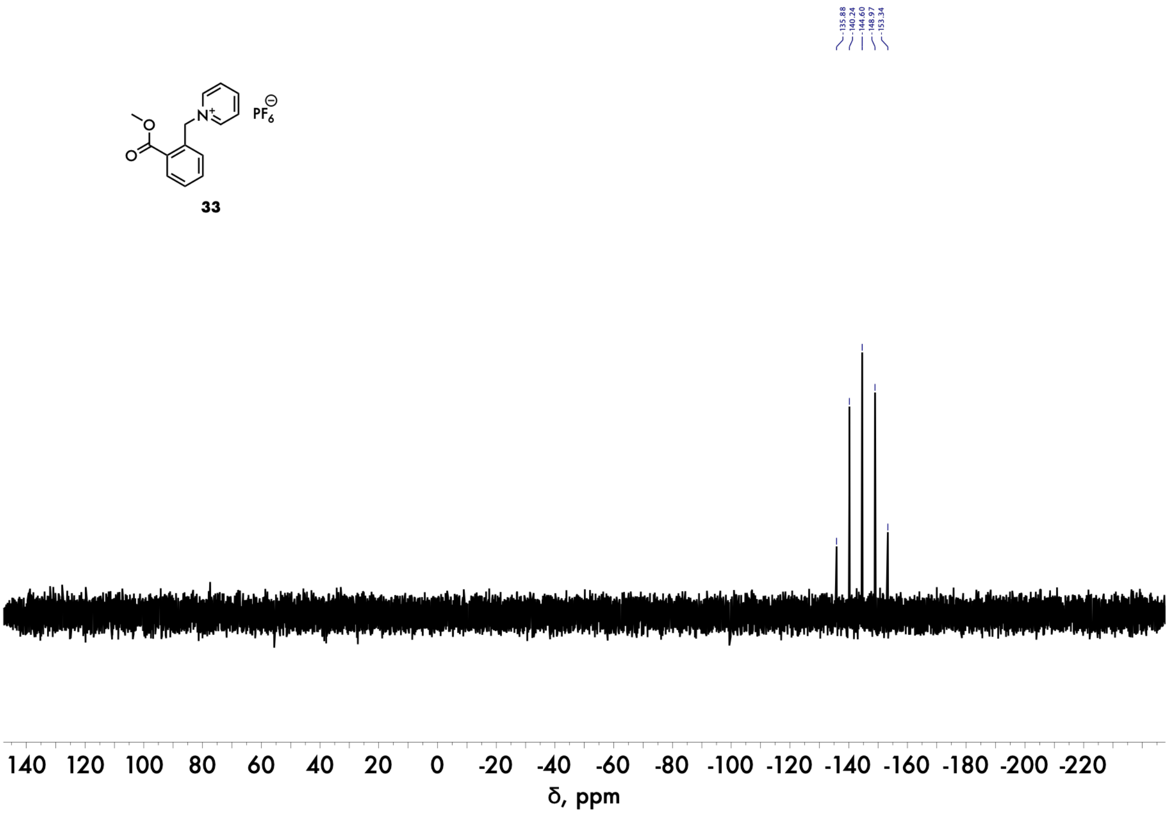

Figure S146. ^{31}P NMR (162 MHz, CD_3CN) spectrum of **33** (o-isomer).

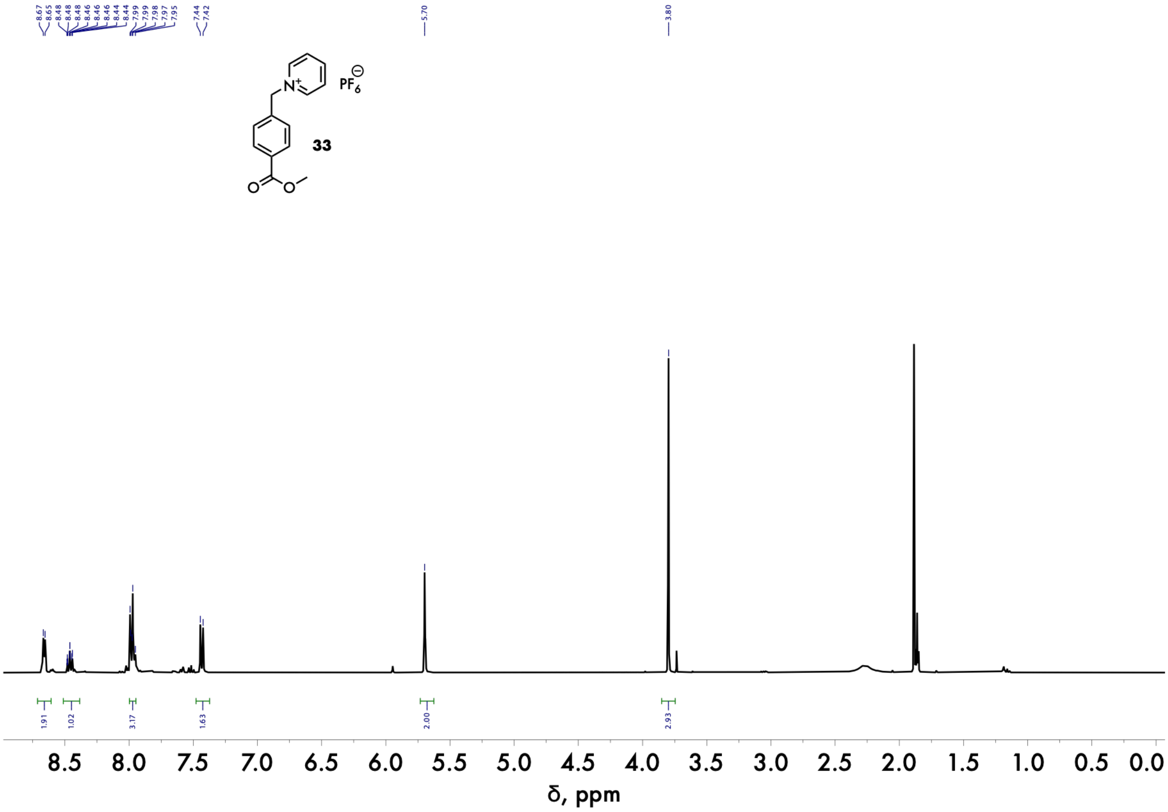

Figure S147. ^{1}H NMR (400 MHz, CD_3CN) spectrum of **33** (p-isomer).

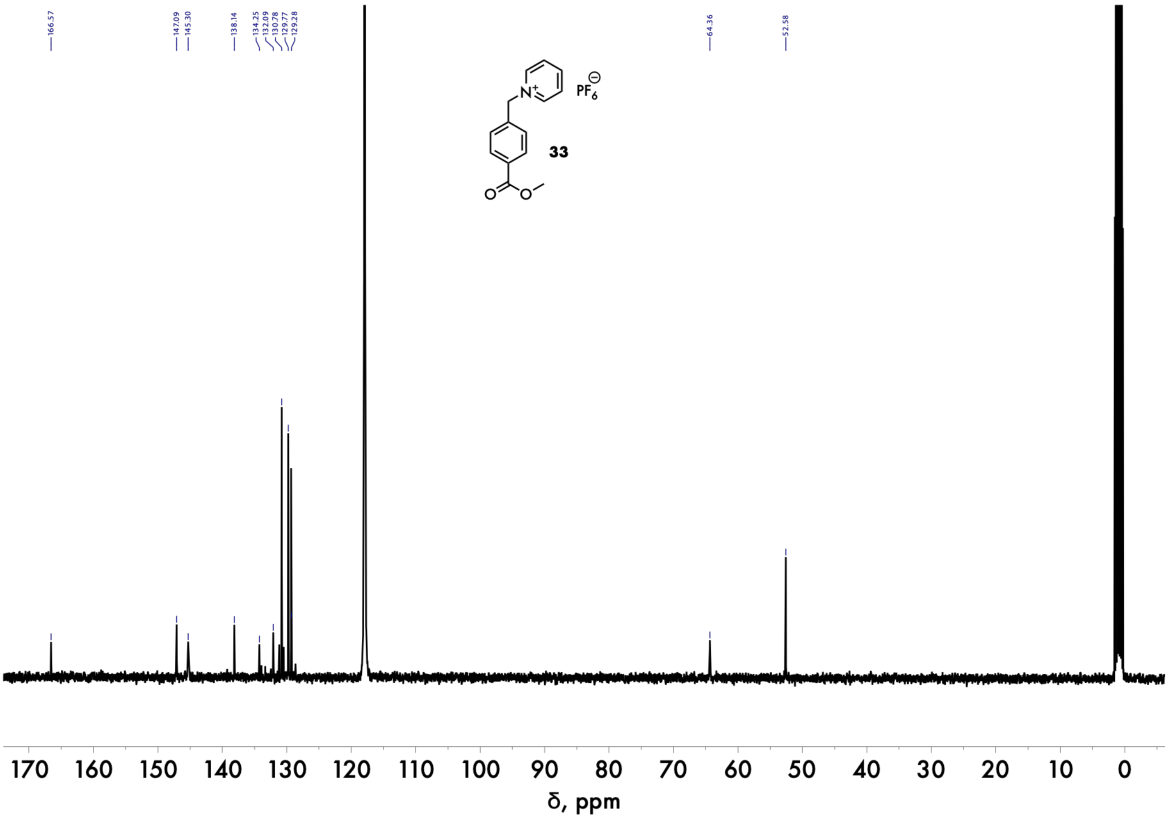

Figure S148. ^{13}C NMR (101 MHz, CD_3CN) spectrum of **33** (p-isomer).

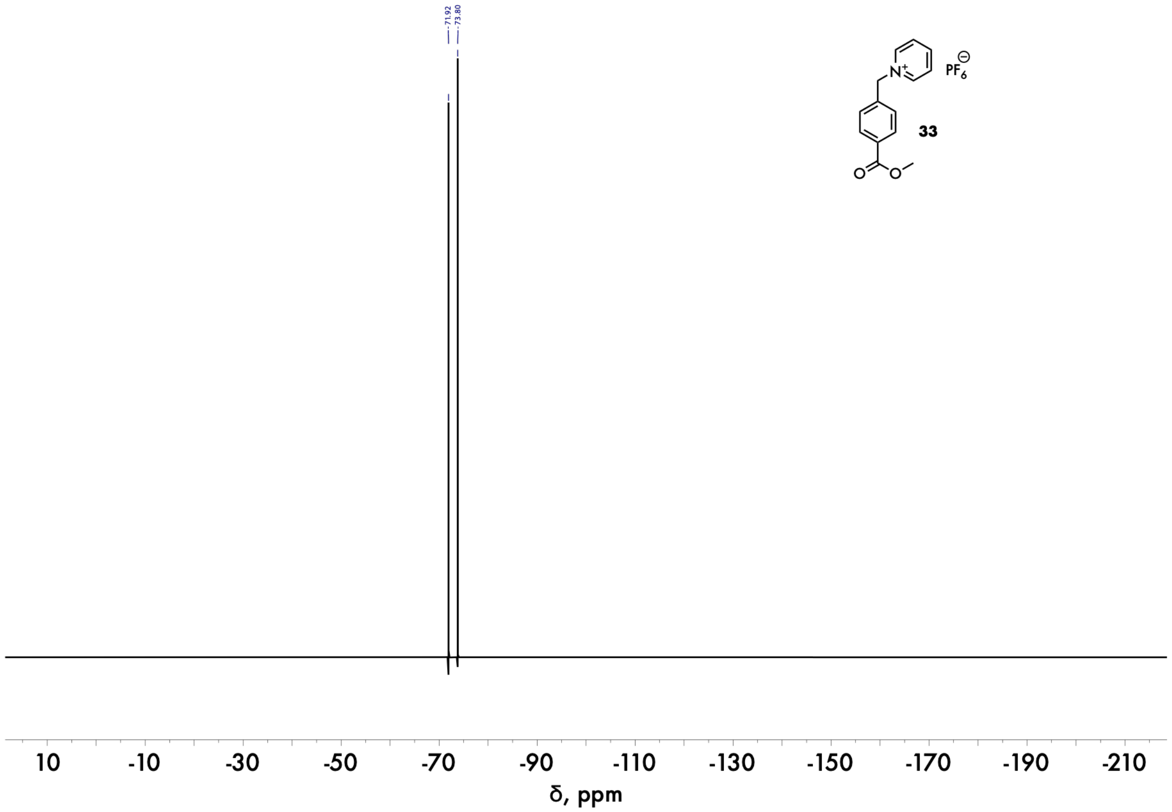

Figure S149. ^{19}F NMR (376 MHz, CD_3CN) spectrum of **33** (p-isomer).

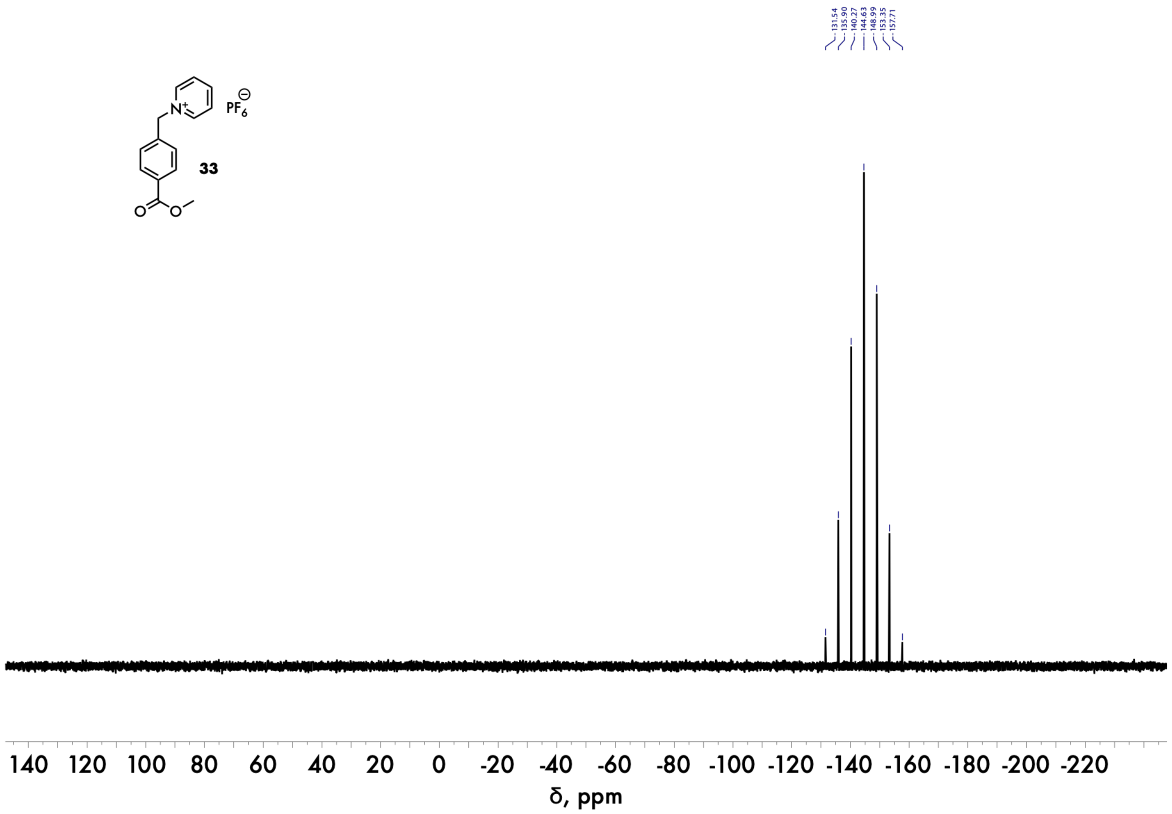

Figure S150. ^{31}P NMR (162 MHz, CD_3CN) spectrum of **33** (p-isomer).

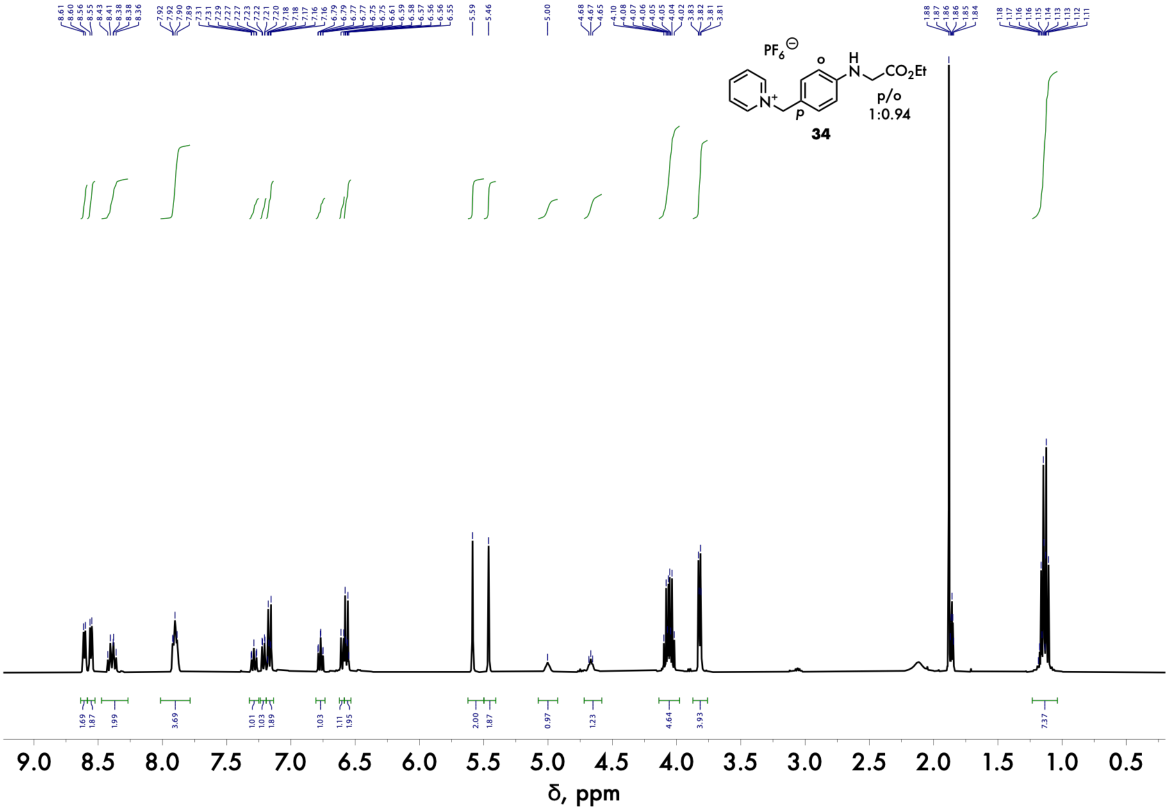

Figure S151. ^{1}H NMR (400 MHz, CD_3CN) spectrum of **34**.

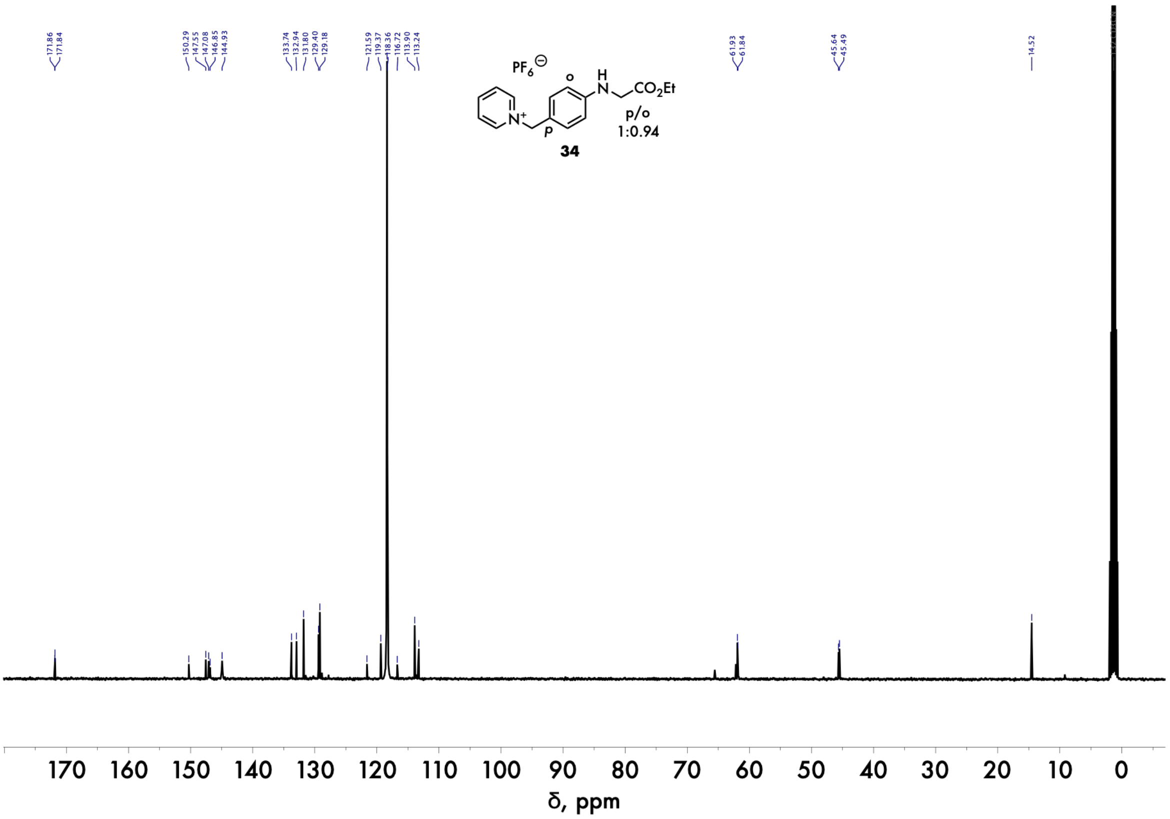

Figure S152. ^{13}C NMR (101 MHz, CD_3CN) spectrum of **34**.

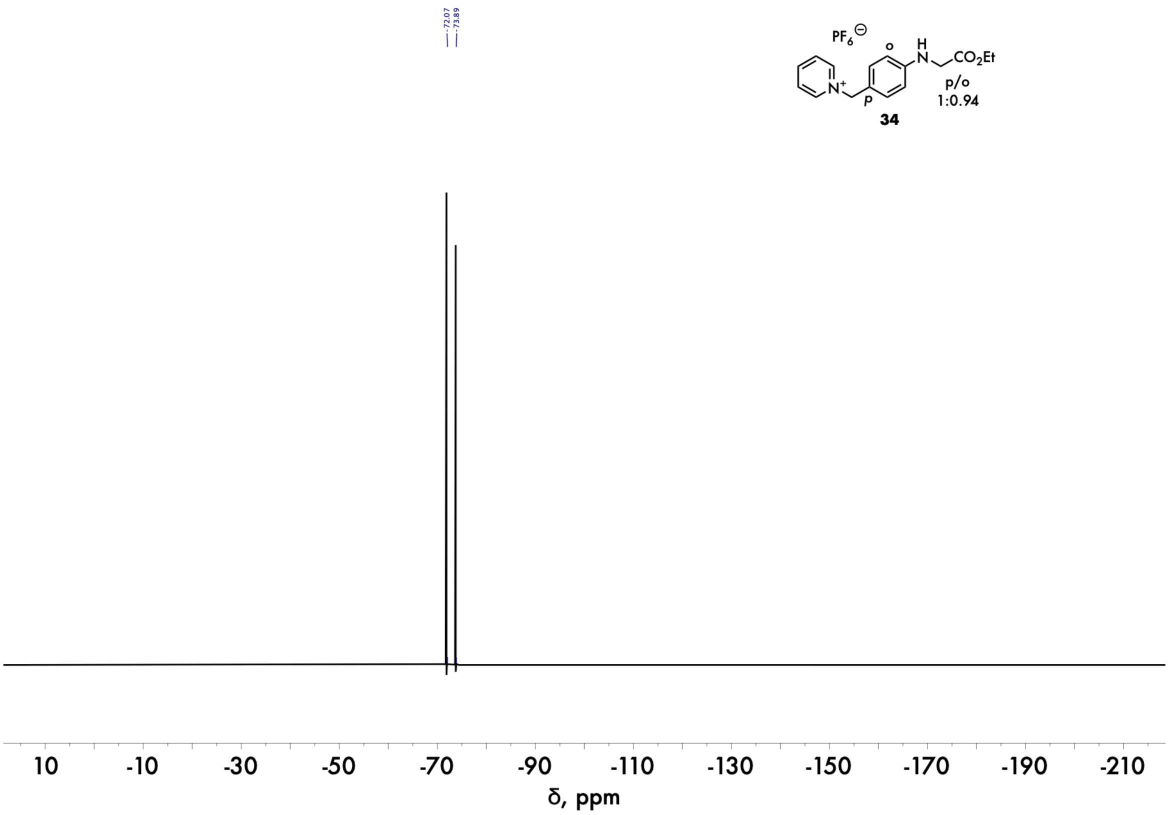

Figure S153. ${}^{19}F$ NMR (376 MHz, CD_3CN) spectrum of **34**.

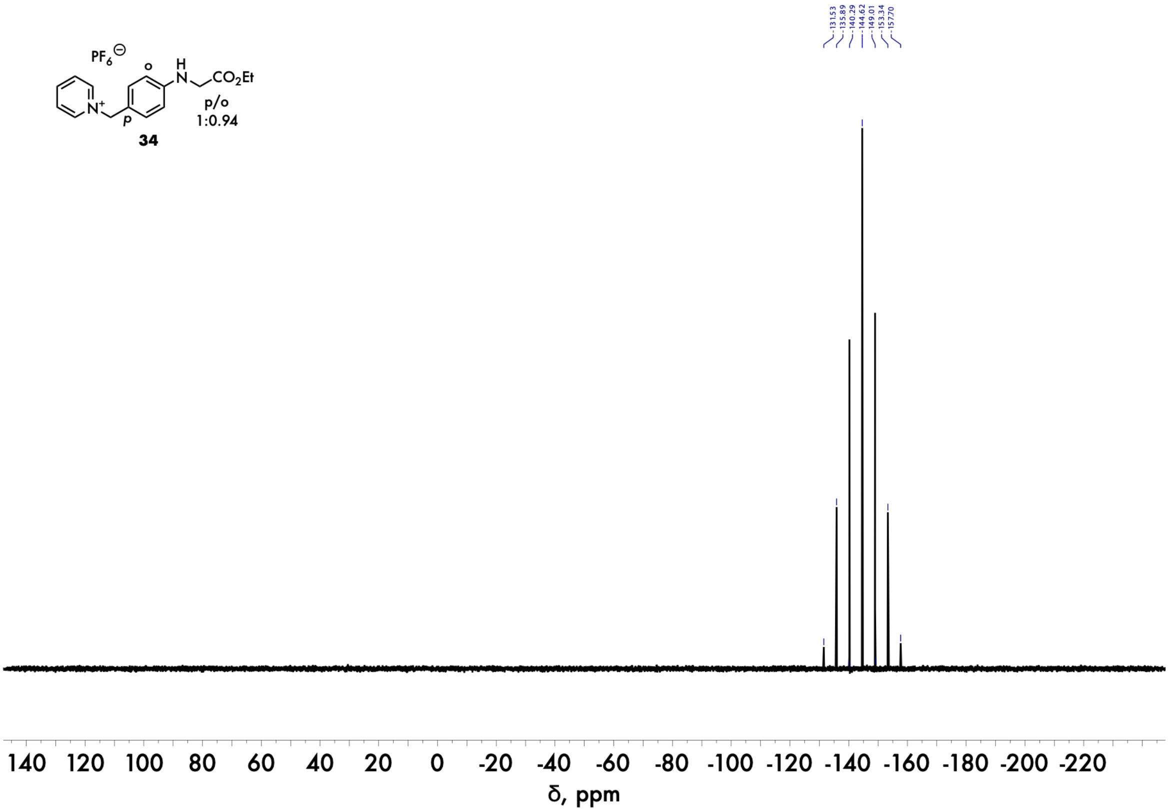

Figure S154. ${}^{31}P$ NMR (162 MHz, CD_3CN) spectrum of **34**.

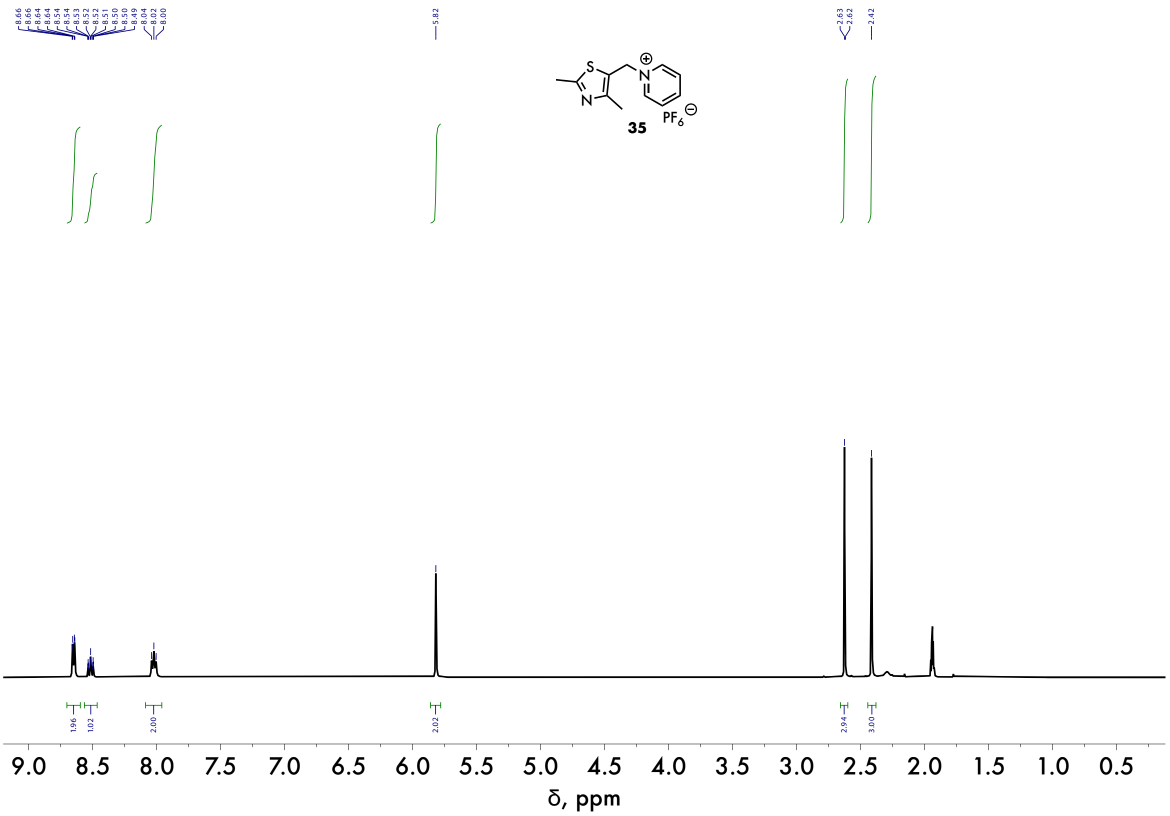

Figure S155. ^{1}H NMR (400 MHz, CD_3CN) spectrum of **35**.

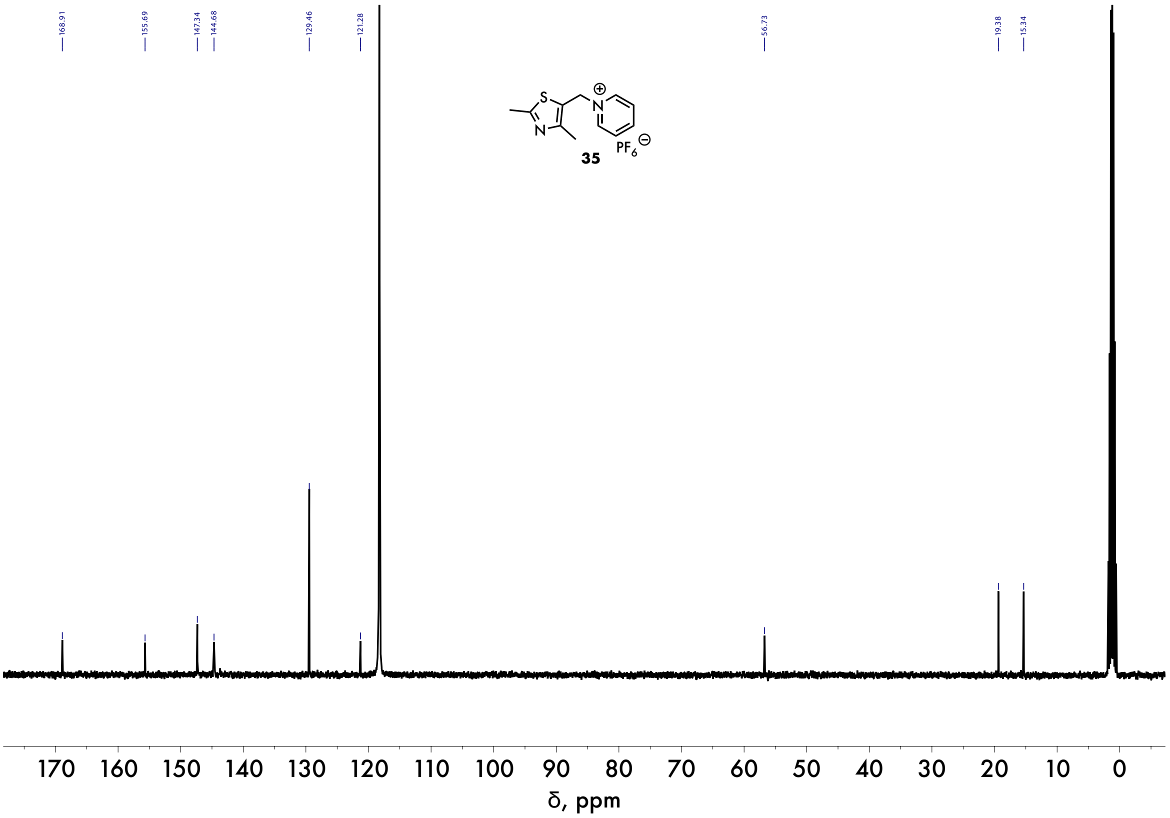

Figure S156. ^{13}C NMR (101 MHz, CD_3CN) spectrum of **35**.

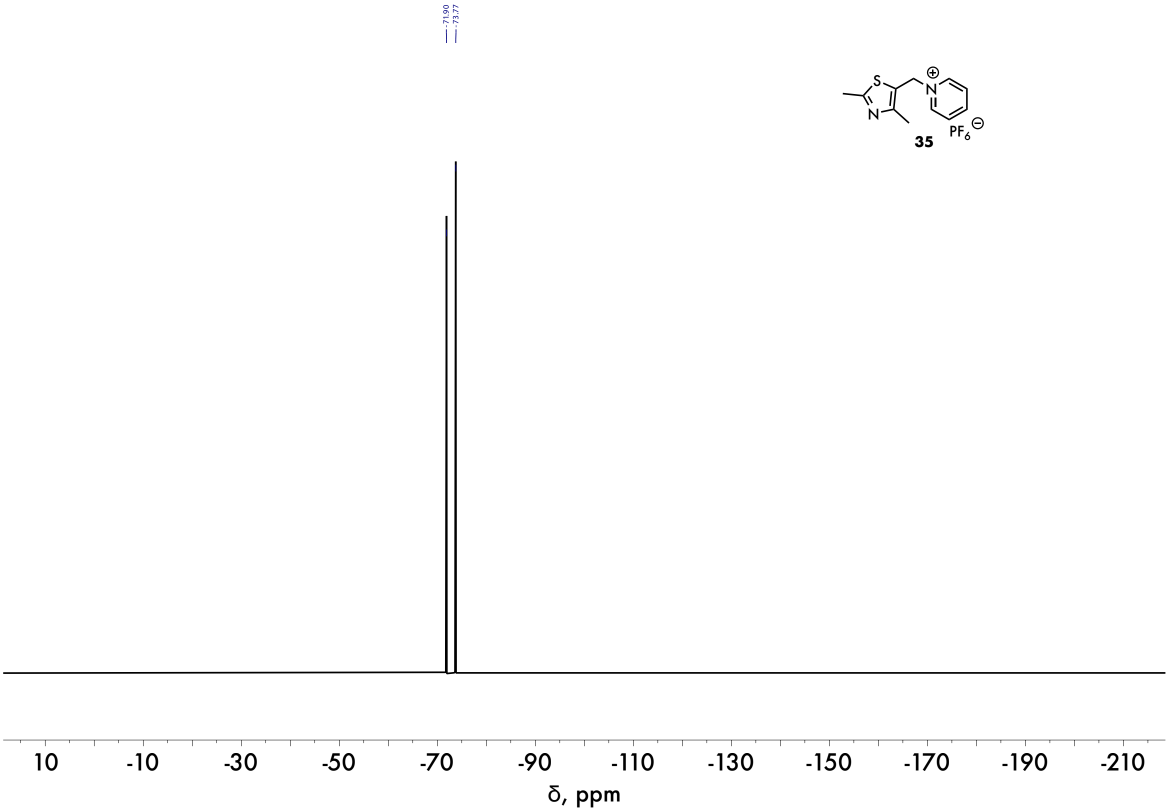

Figure S157. ^{19}F NMR (376 MHz, CD_3CN) spectrum of **35**.

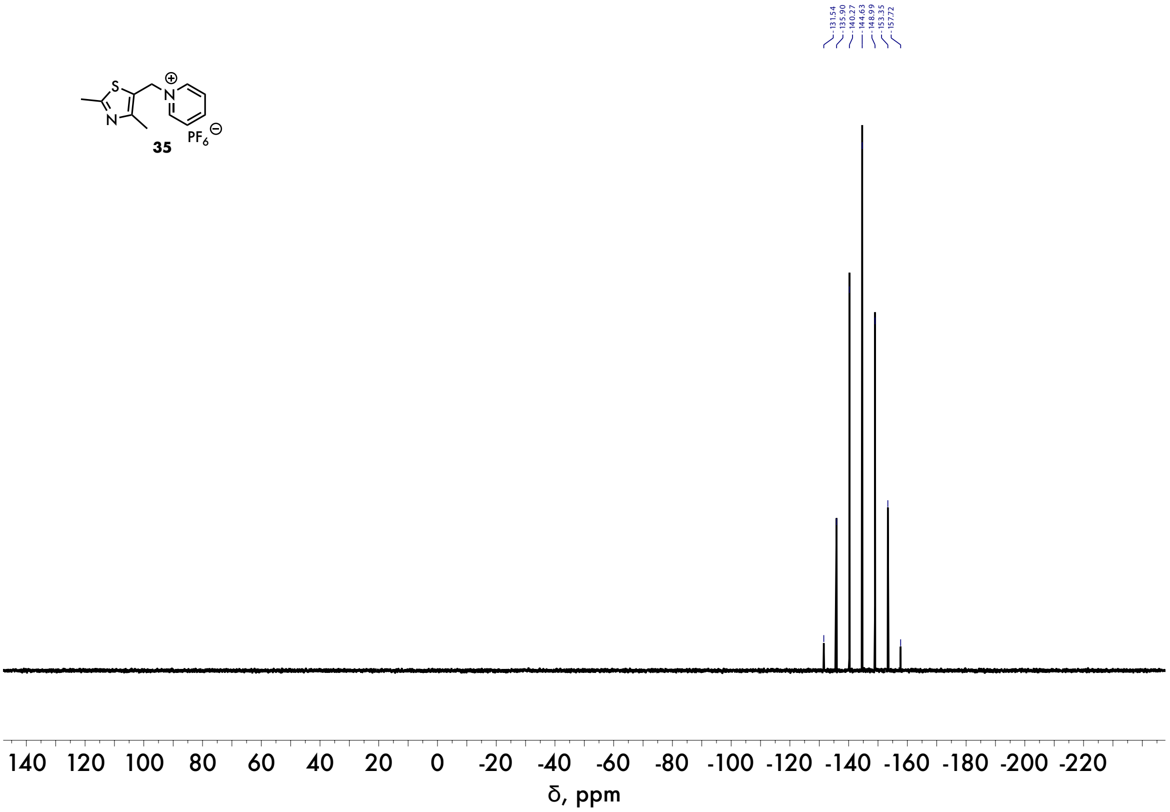

Figure S158. ^{31}P NMR (162 MHz, CD_3CN) spectrum of **35**.

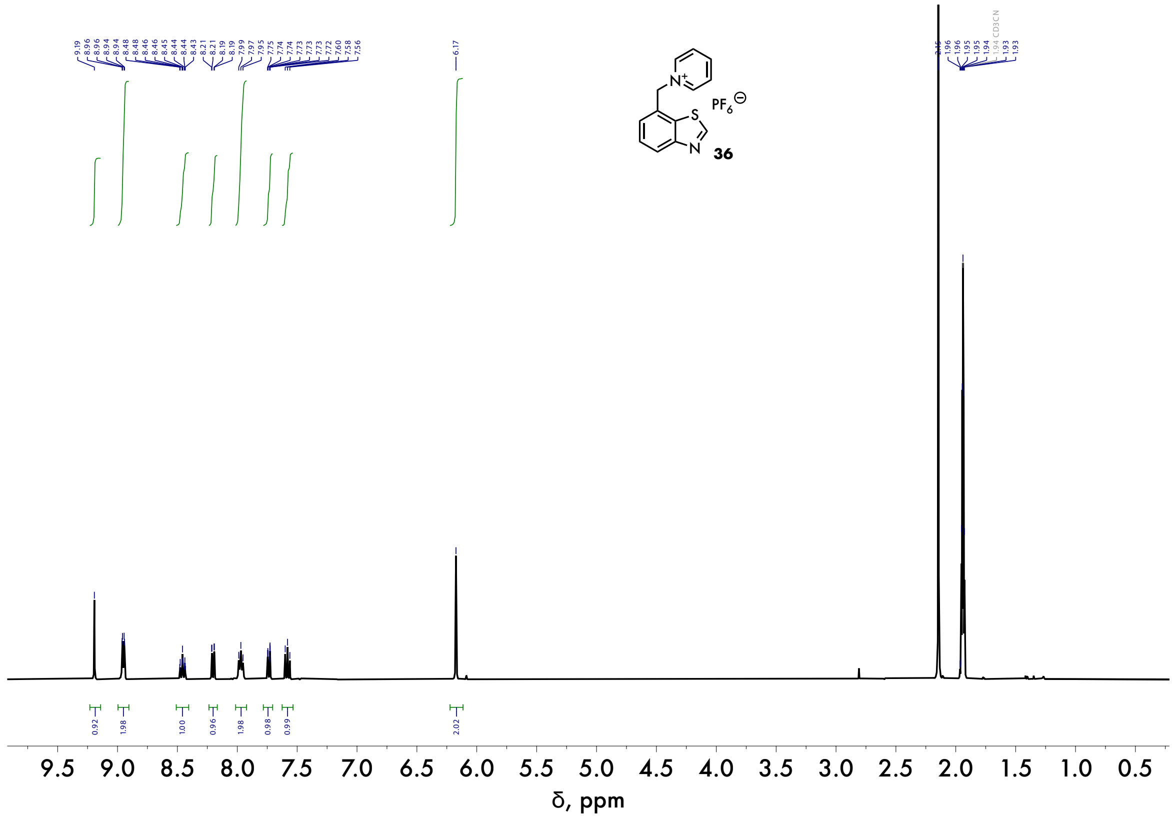

Figure S159. ^{1}H NMR (400 MHz, CD_3CN) spectrum of **36**.

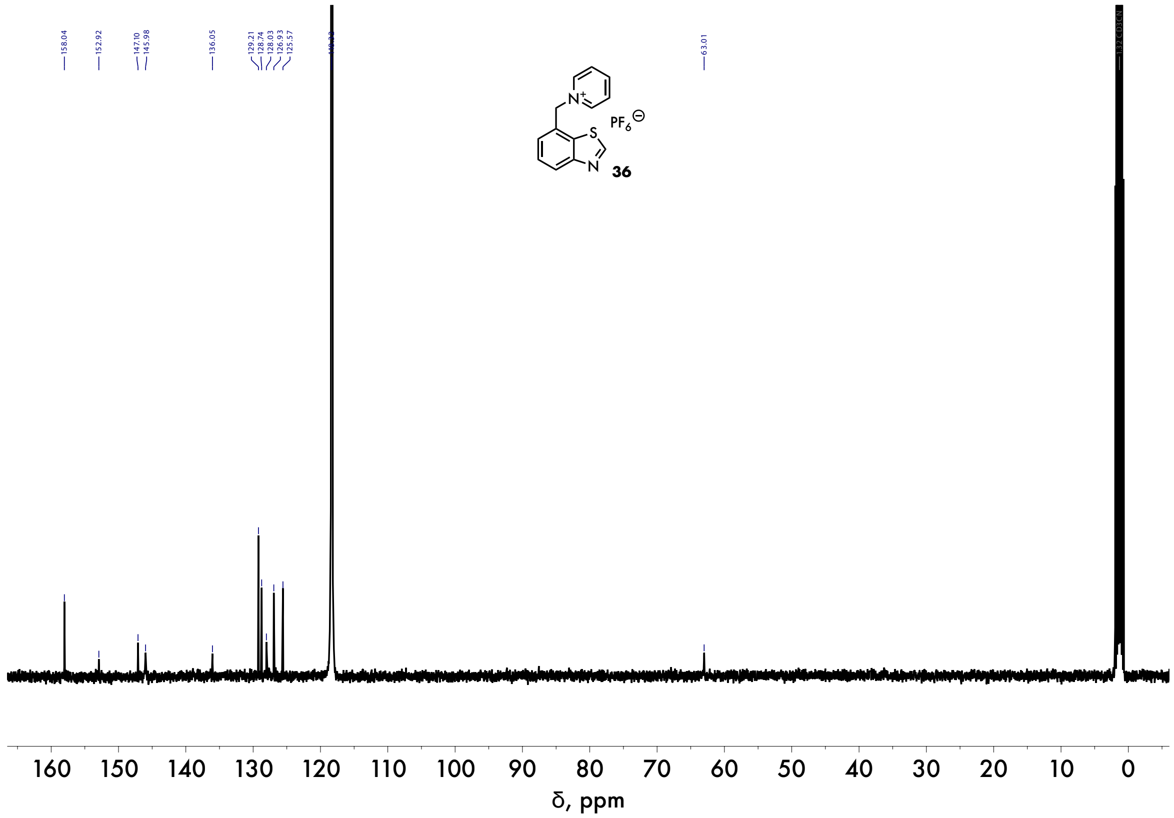

Figure S160. ^{13}C NMR (101 MHz, CD_3CN) spectrum of **36**.

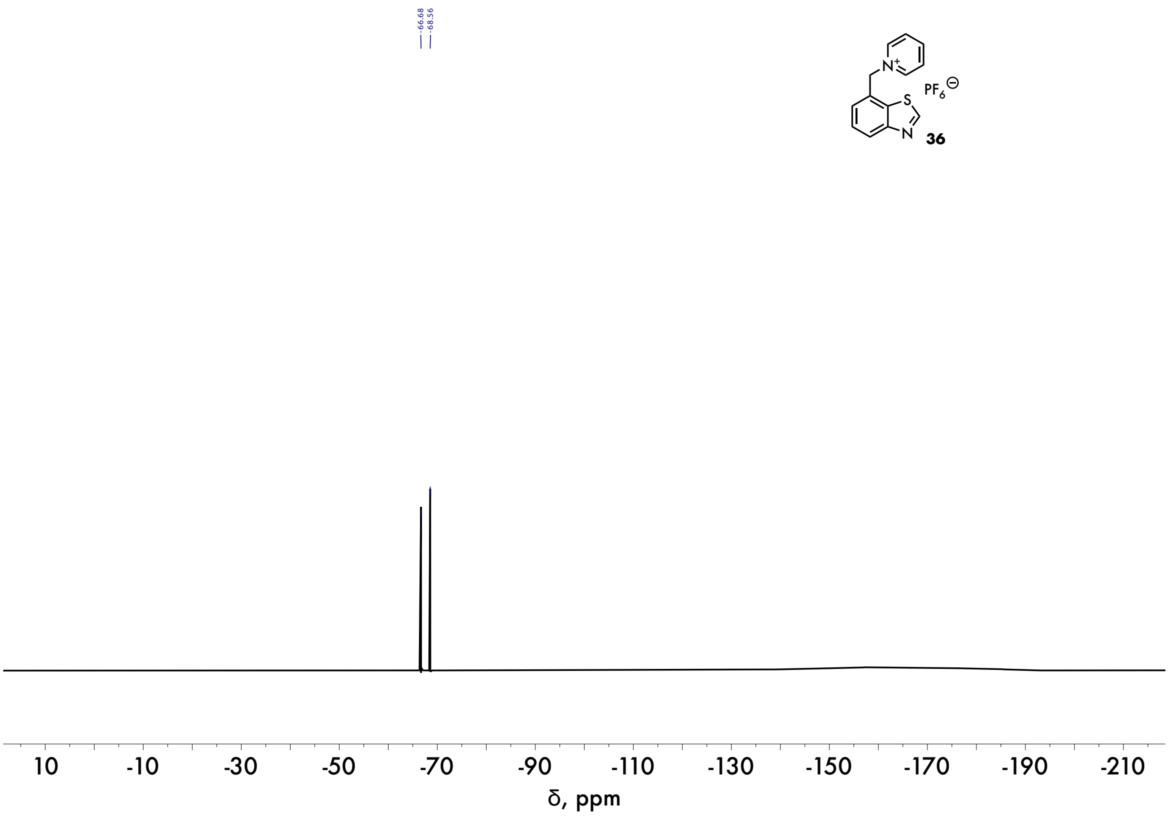

Figure S161. ^{19}F NMR (376 MHz, CD_3CN) spectrum of **36**.

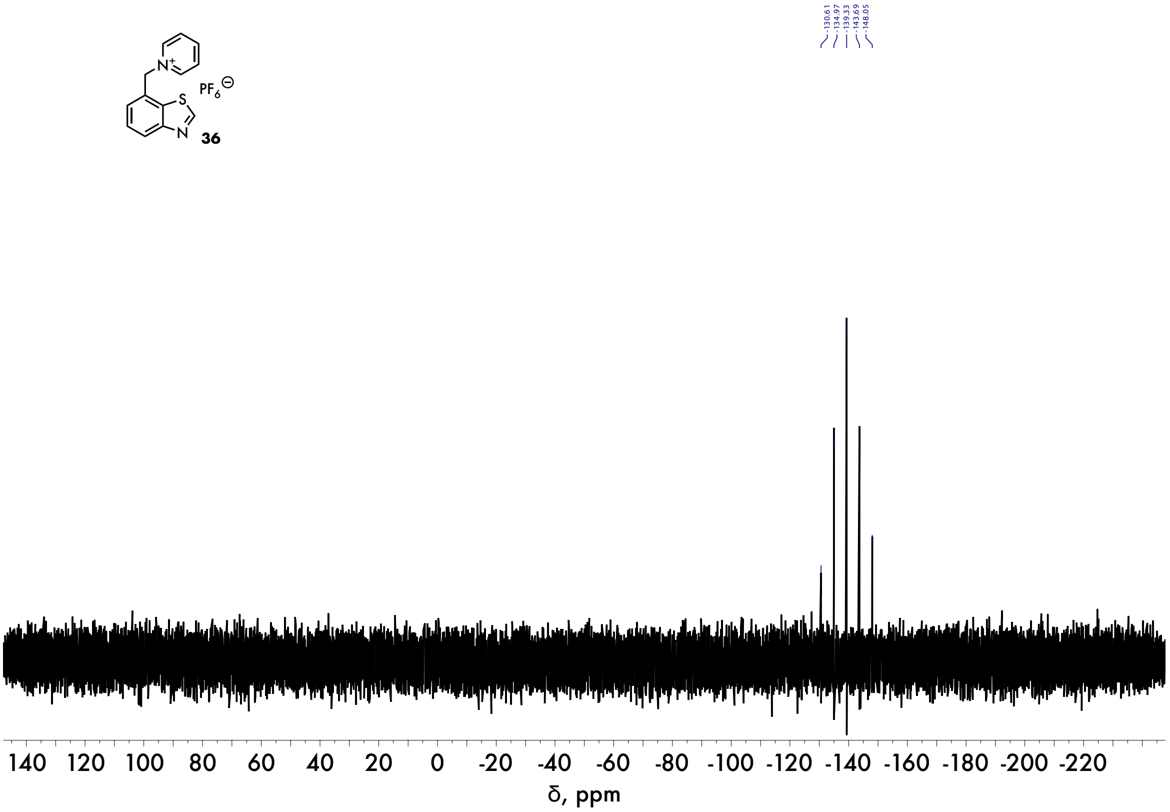

Figure S162. ^{31}P NMR (162 MHz, CD_3CN) spectrum of **36**.

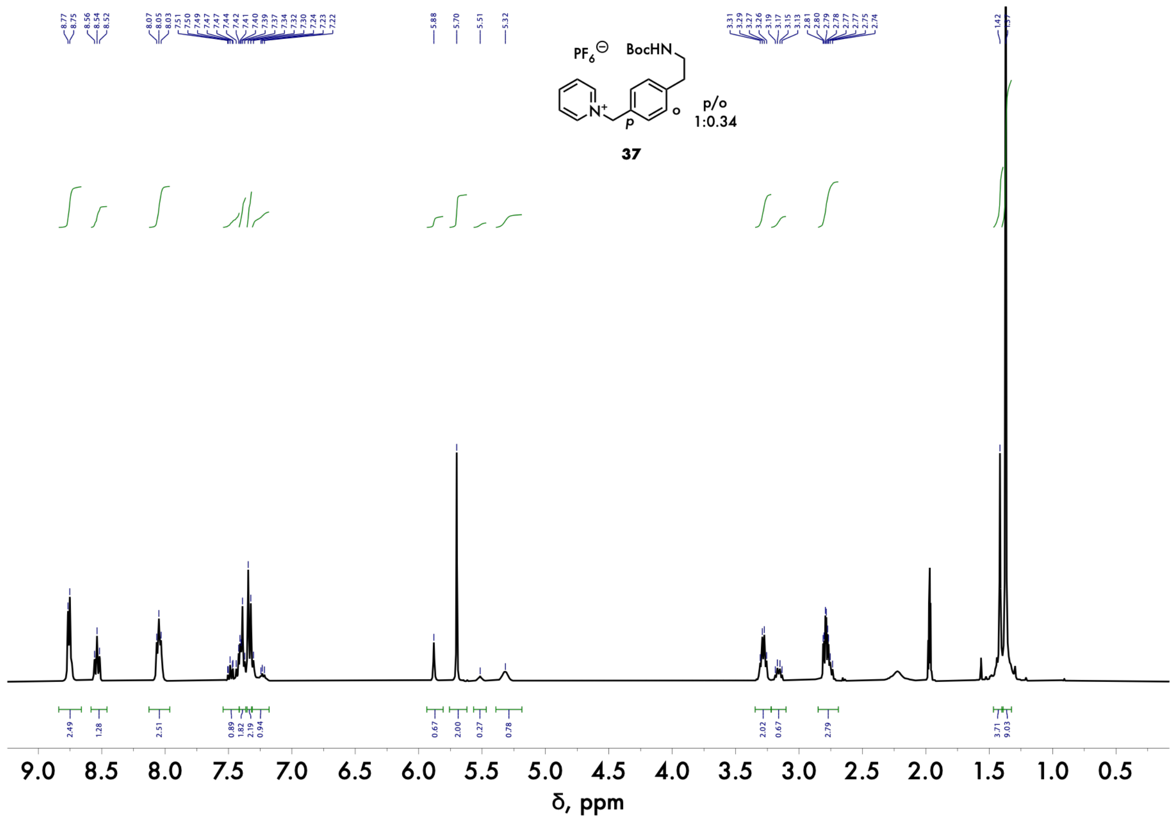

Figure S163. ^{1}H NMR (400 MHz, CD_3CN) spectrum of **37**.

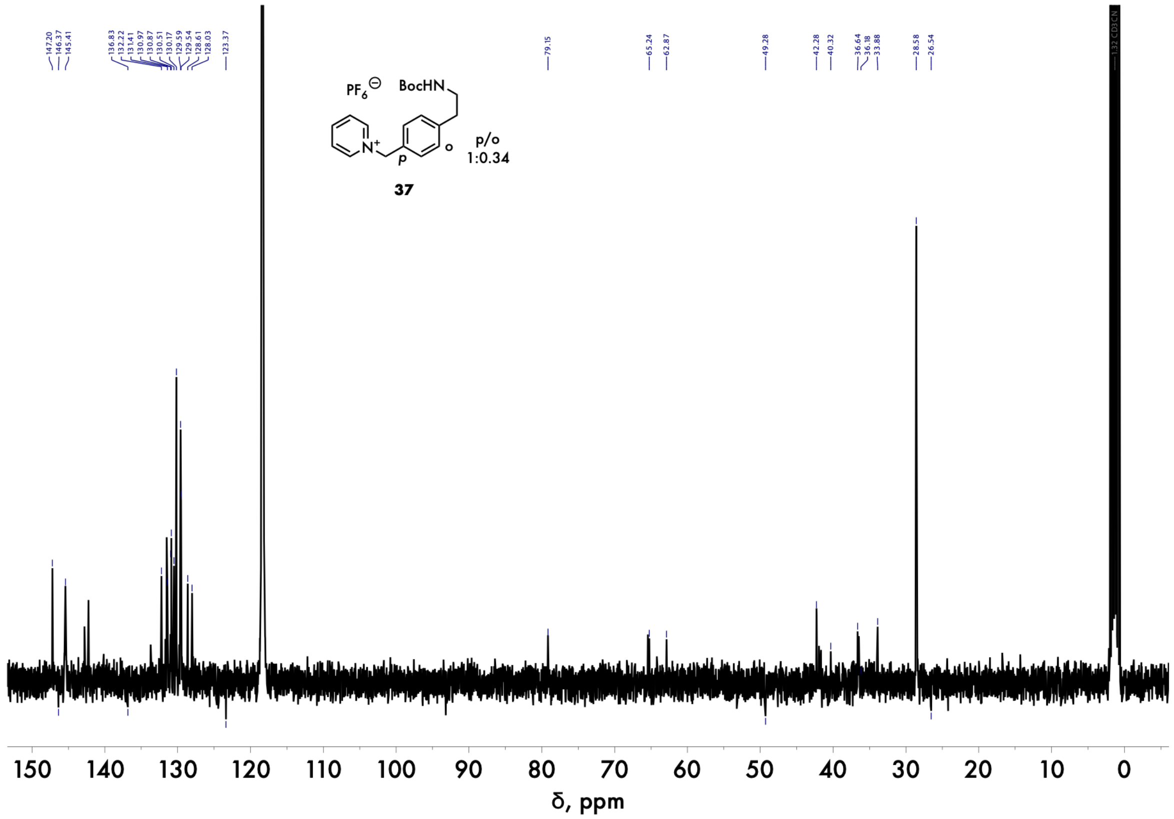

Figure S164. ^{13}C NMR (101 MHz, CD_3CN) spectrum of **37**.

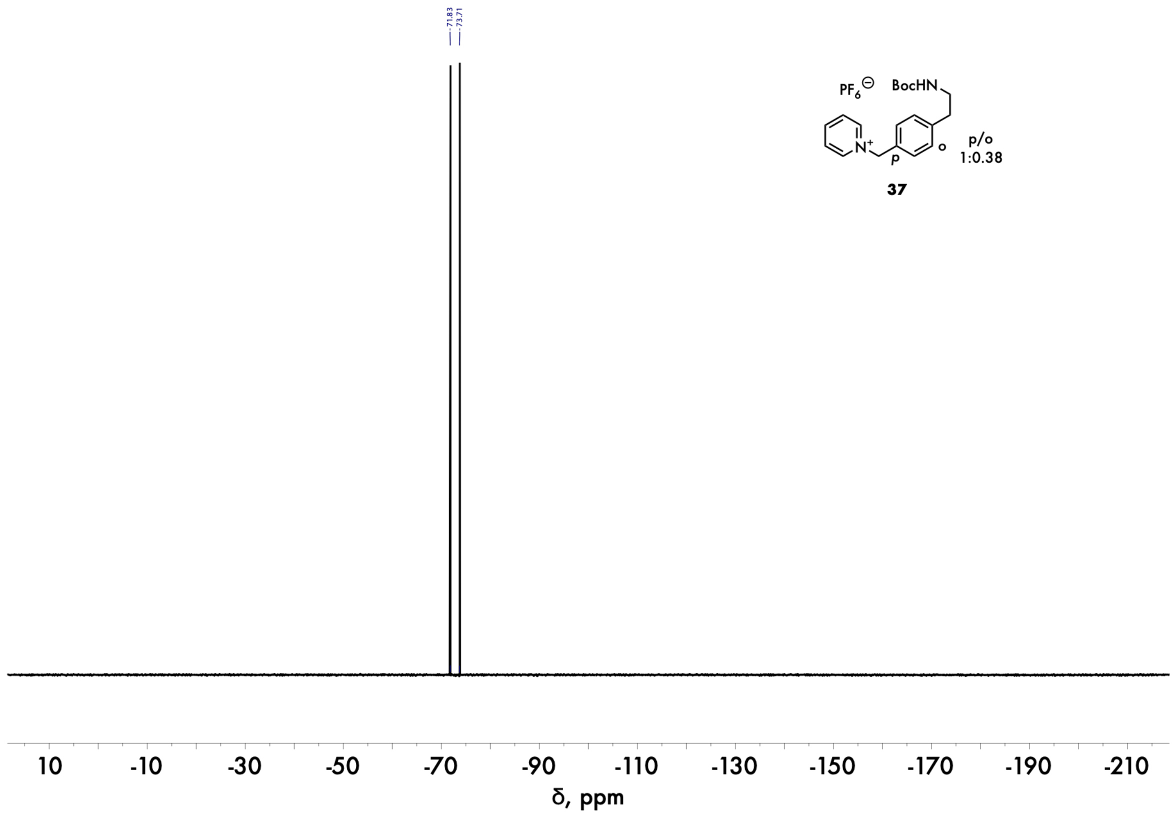

Figure S165. ^{19}F NMR (376 MHz, CD_3CN) spectrum of **37**.

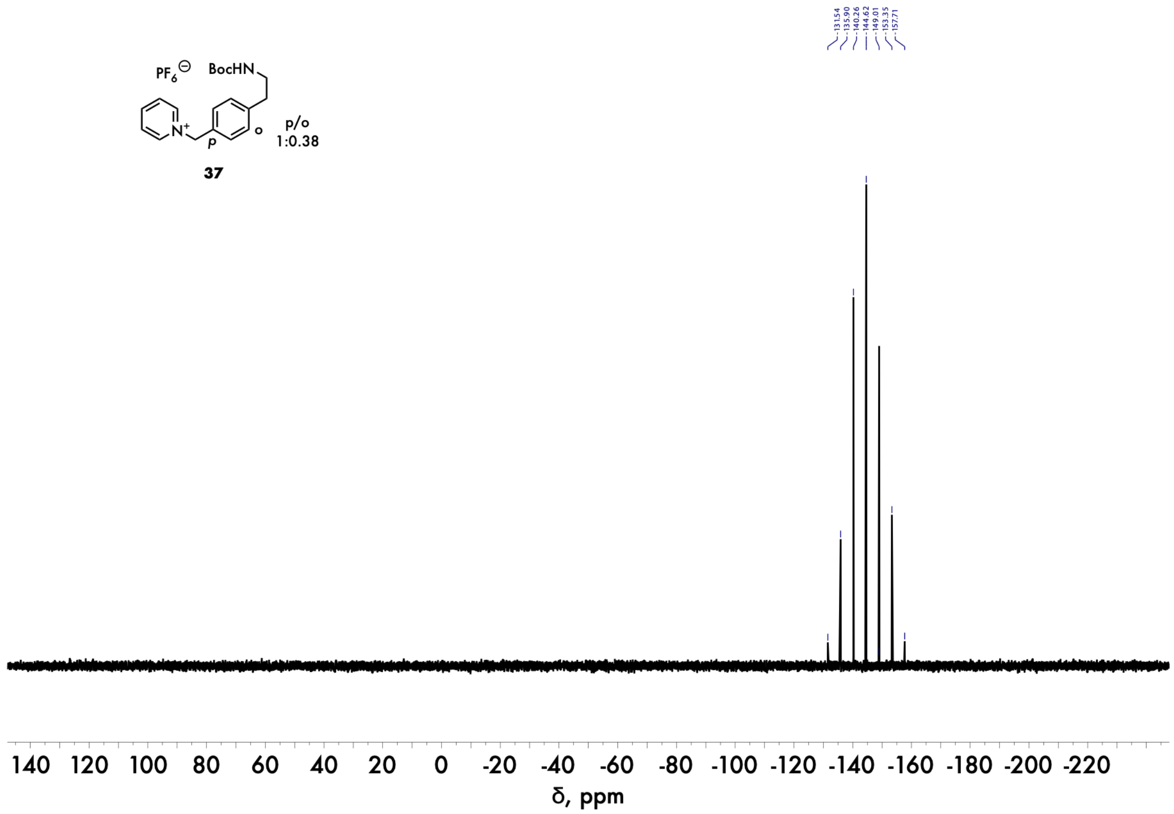

Figure S166. ^{31}P NMR (162 MHz, CD_3CN) spectrum of **37**.

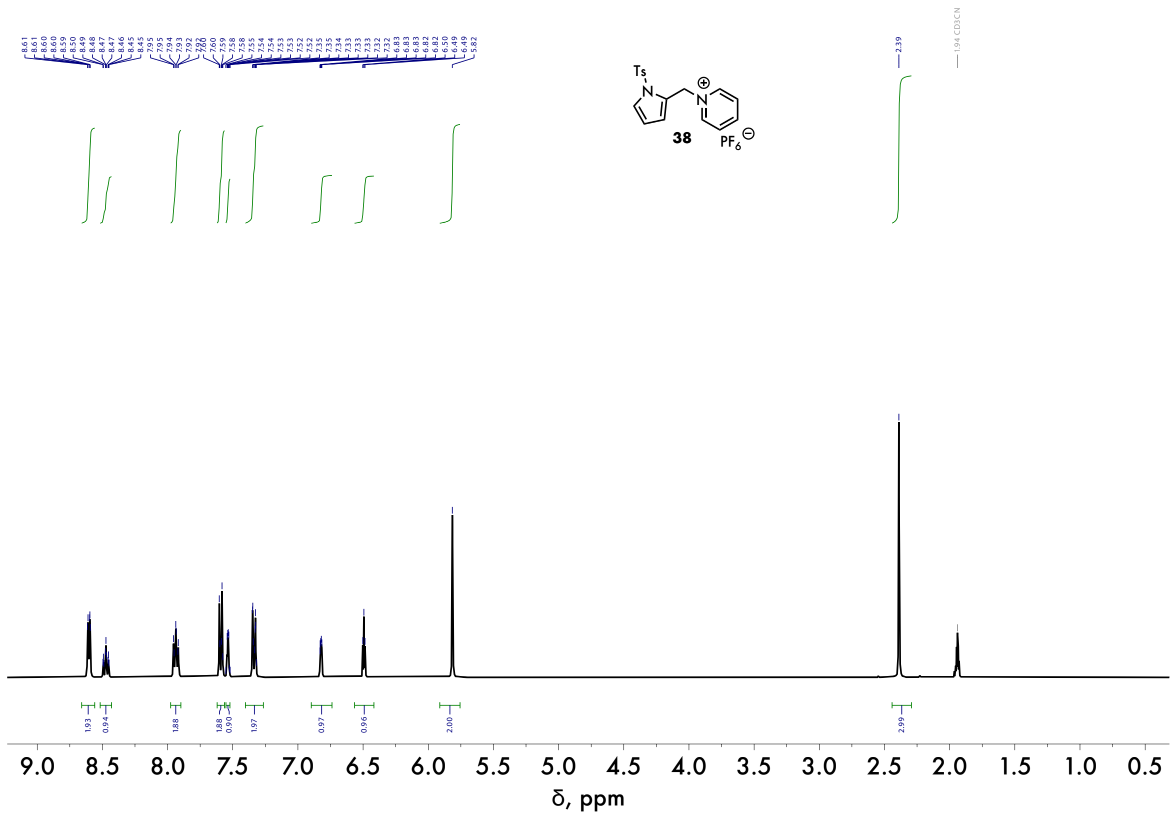

Figure S167. ^{1}H NMR (400 MHz, CD_3CN) spectrum of **38**.

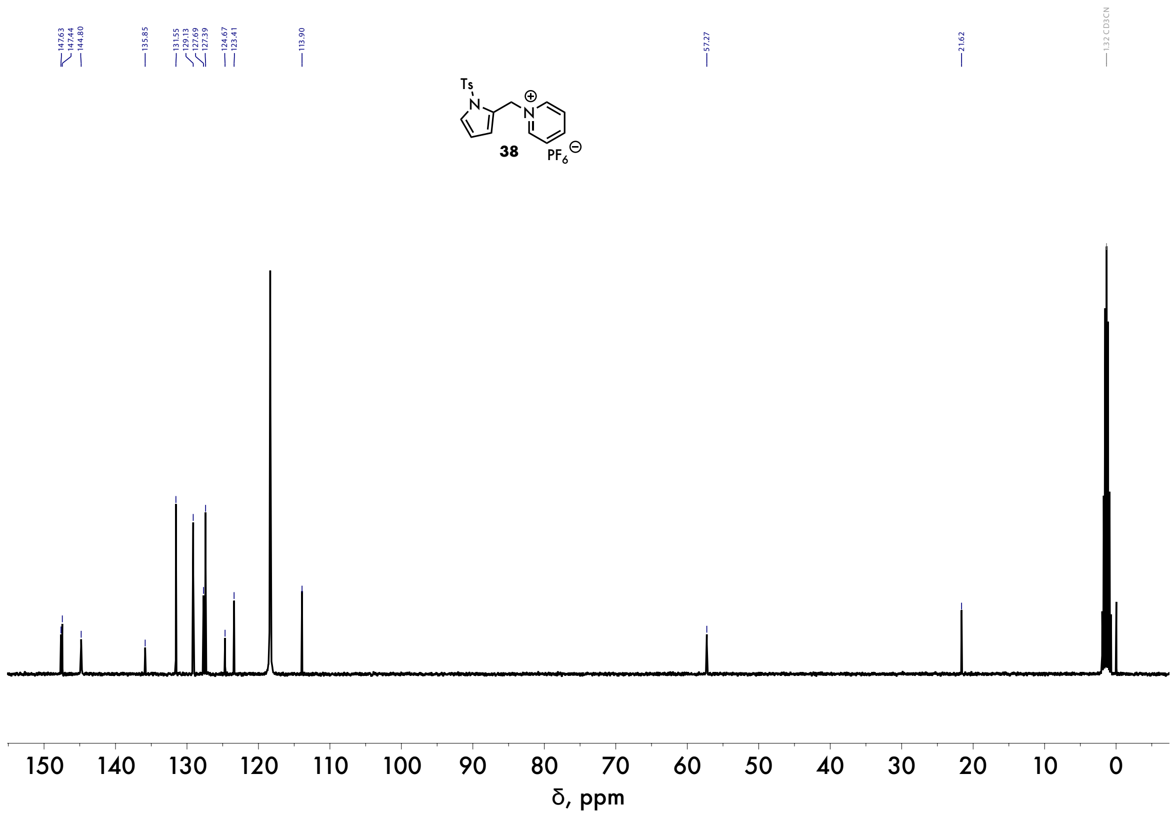

Figure S168. ^{13}C NMR (101 MHz, CD_3CN) spectrum of **38**.

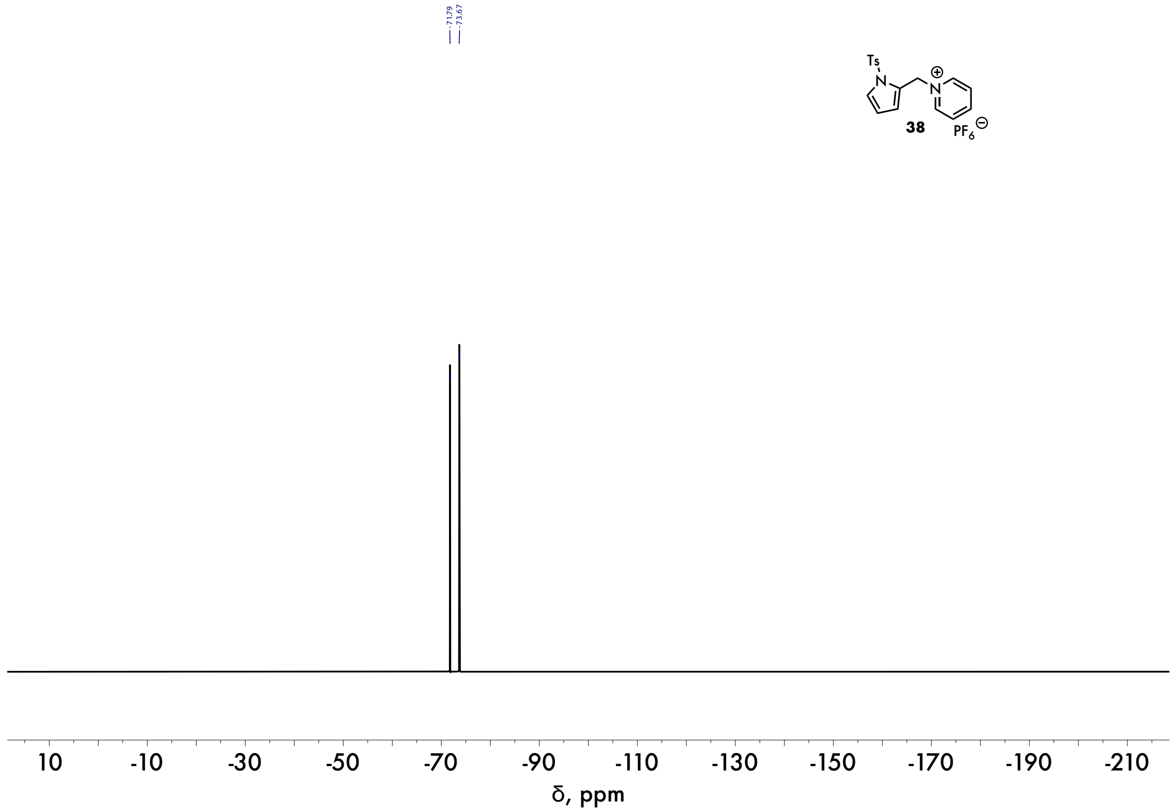

Figure S169. ^{19}F NMR (376 MHz, CD_3CN) spectrum of **38**.

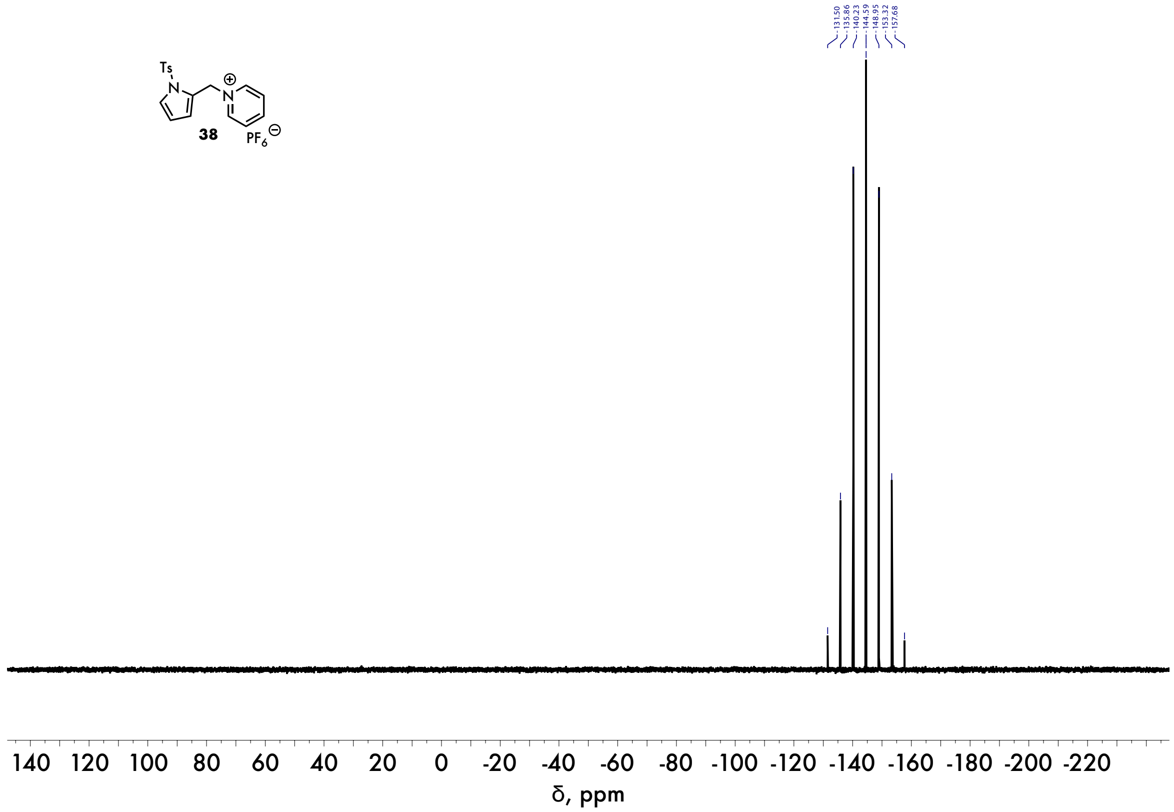

Figure S170. ^{31}P NMR (162 MHz, CD_3CN) spectrum of **38**.

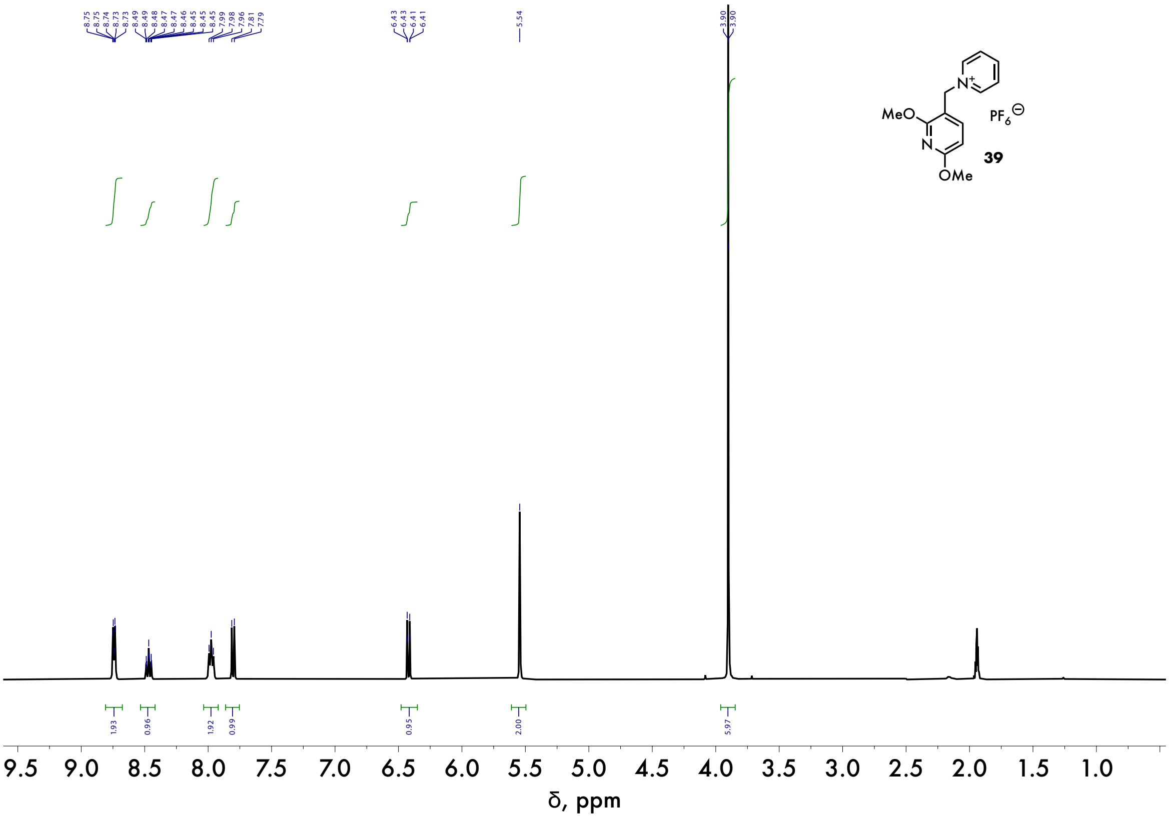

Figure S171. ^{1}H NMR (400 MHz, CD_3CN) spectrum of **39**.

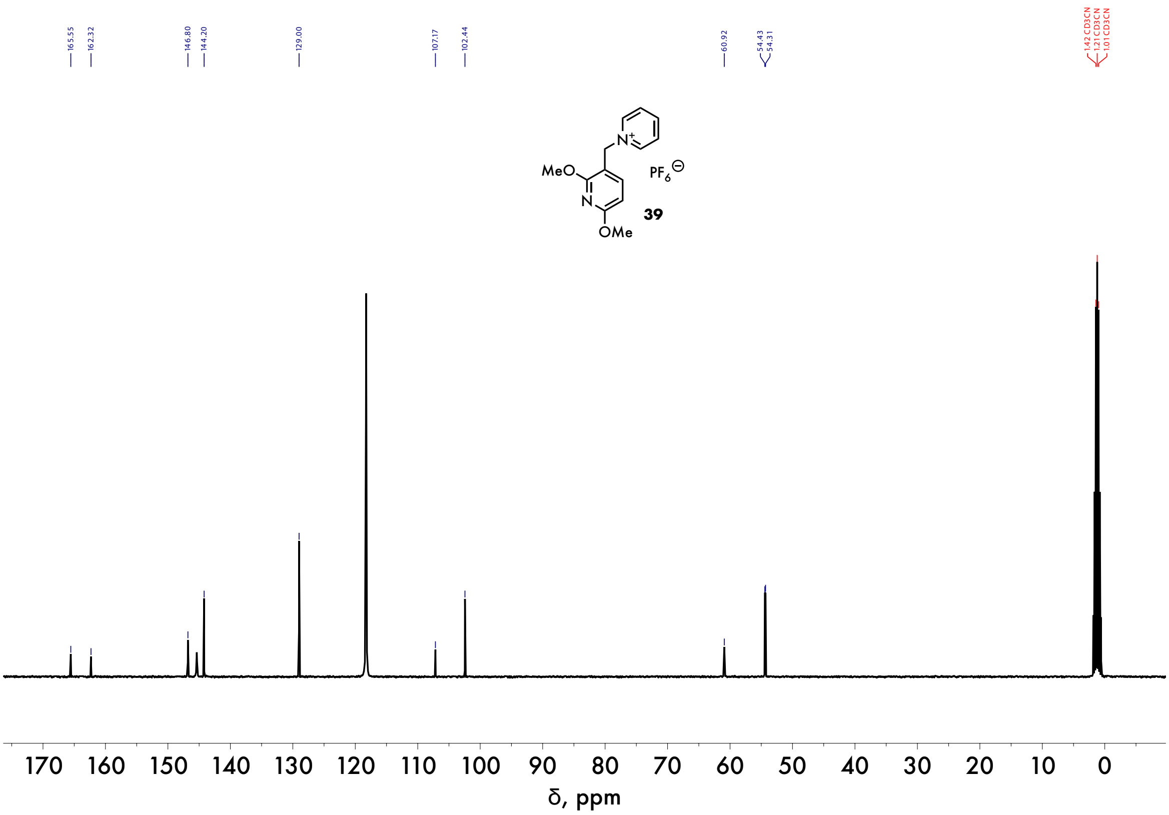

Figure S172. ^{13}C NMR (101 MHz, CD_3CN) spectrum of **39**.

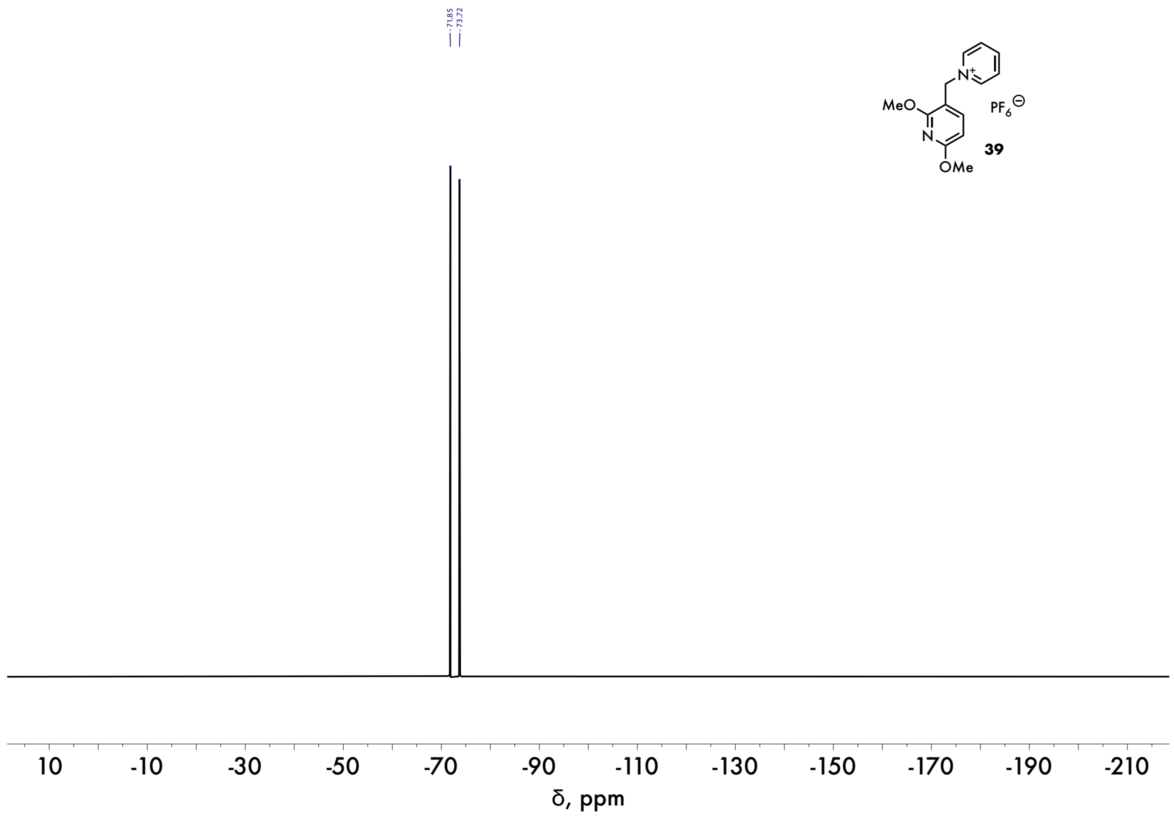

Figure S173. ^{19}F NMR (376 MHz, CD_3CN) spectrum of **39**.

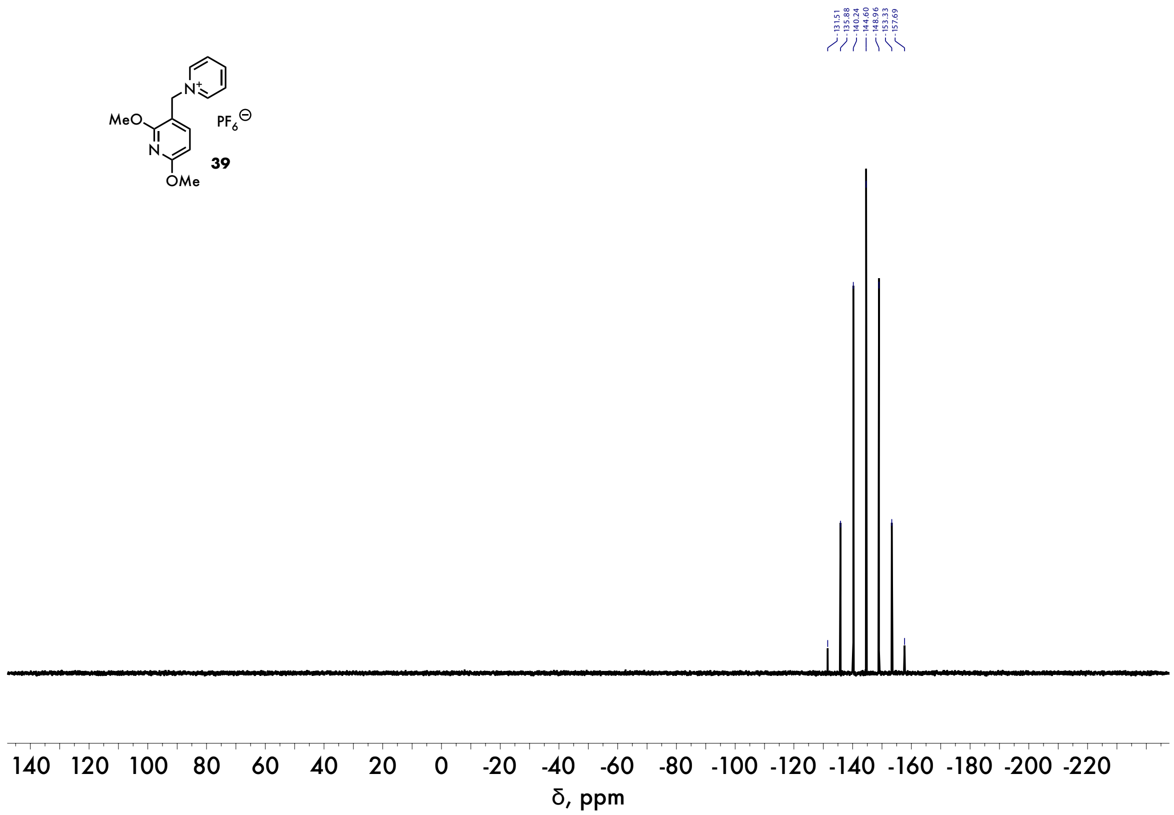

Figure S174. ^{31}P NMR (162 MHz, CD_3CN) spectrum of **39**.

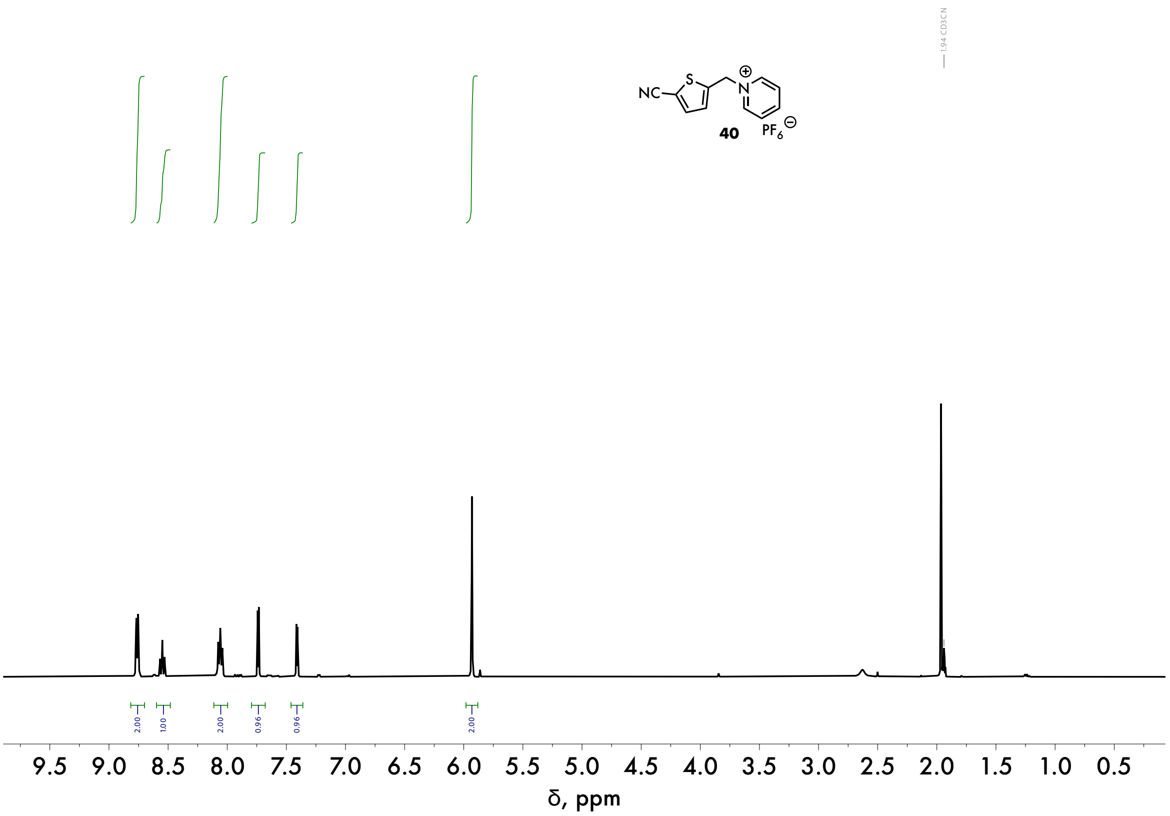

Figure S175. ^{1}H NMR (400 MHz, CD_3CN) spectrum of **40**.

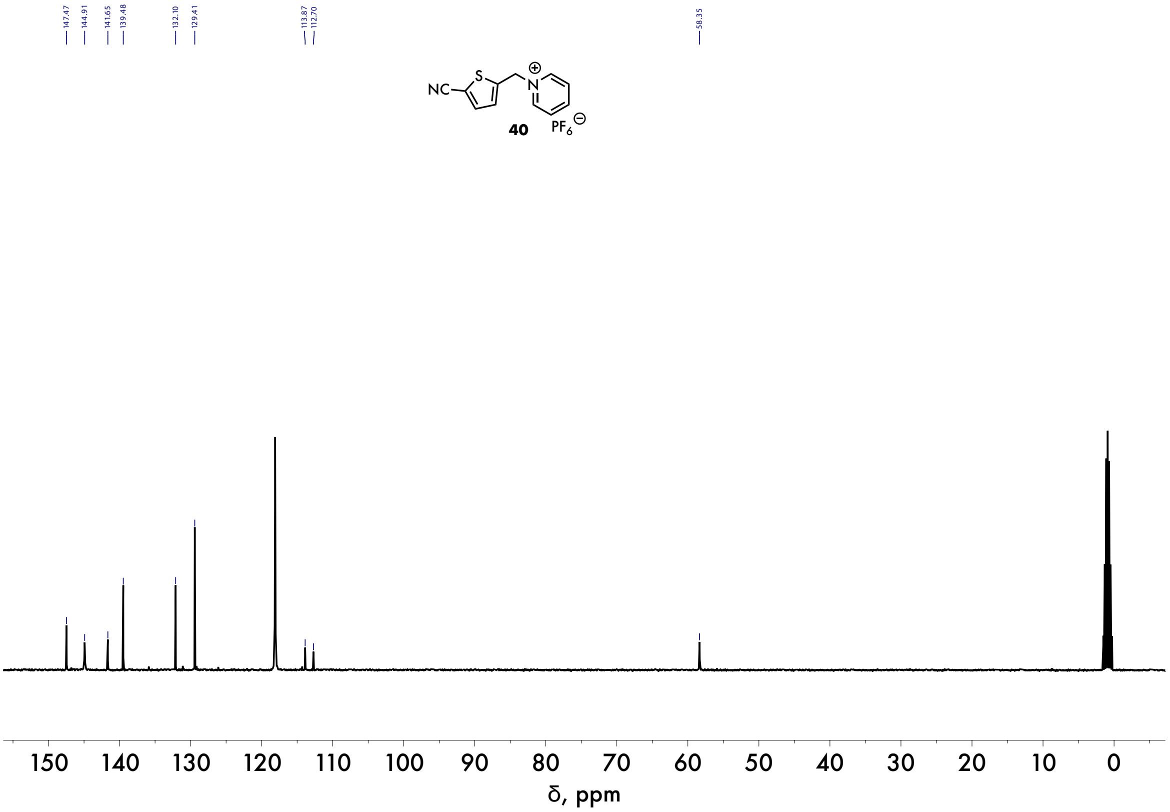

Figure S176. ^{13}C NMR (101 MHz, CD_3CN) spectrum of **40**.

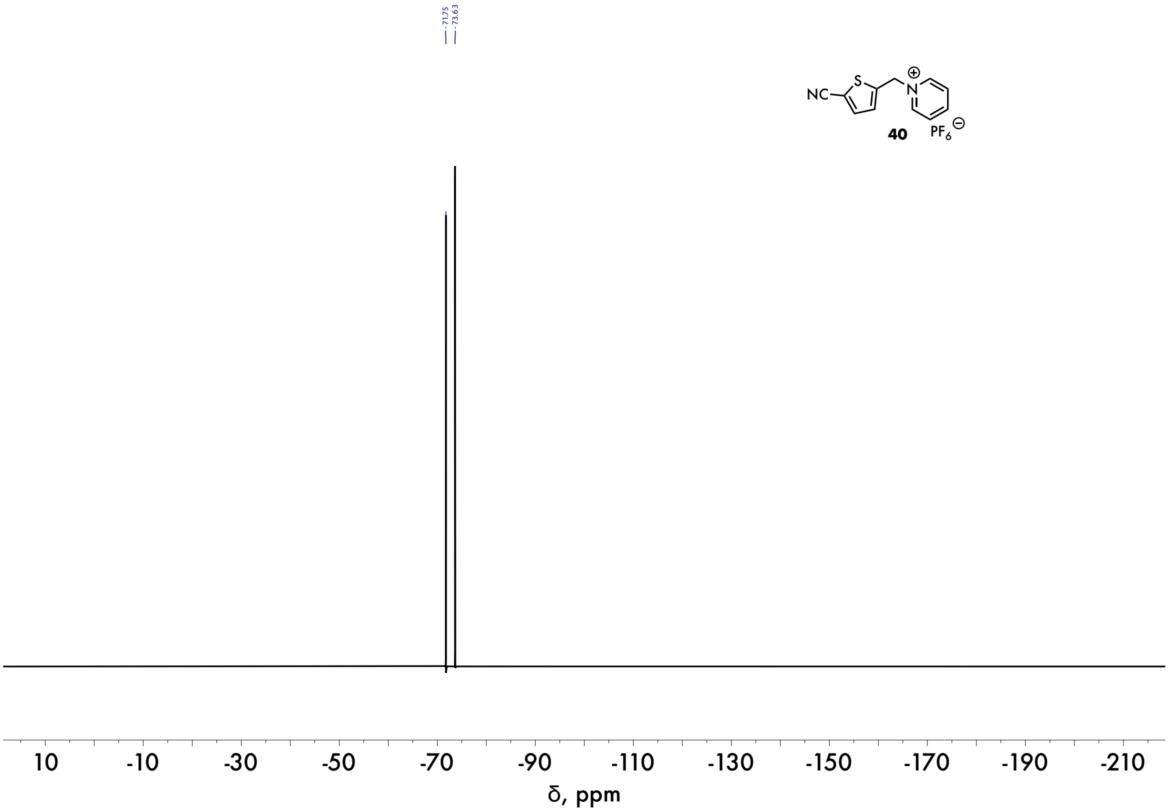

Figure S177. ^{19}F NMR (376 MHz, CD_3CN) spectrum of **40**.

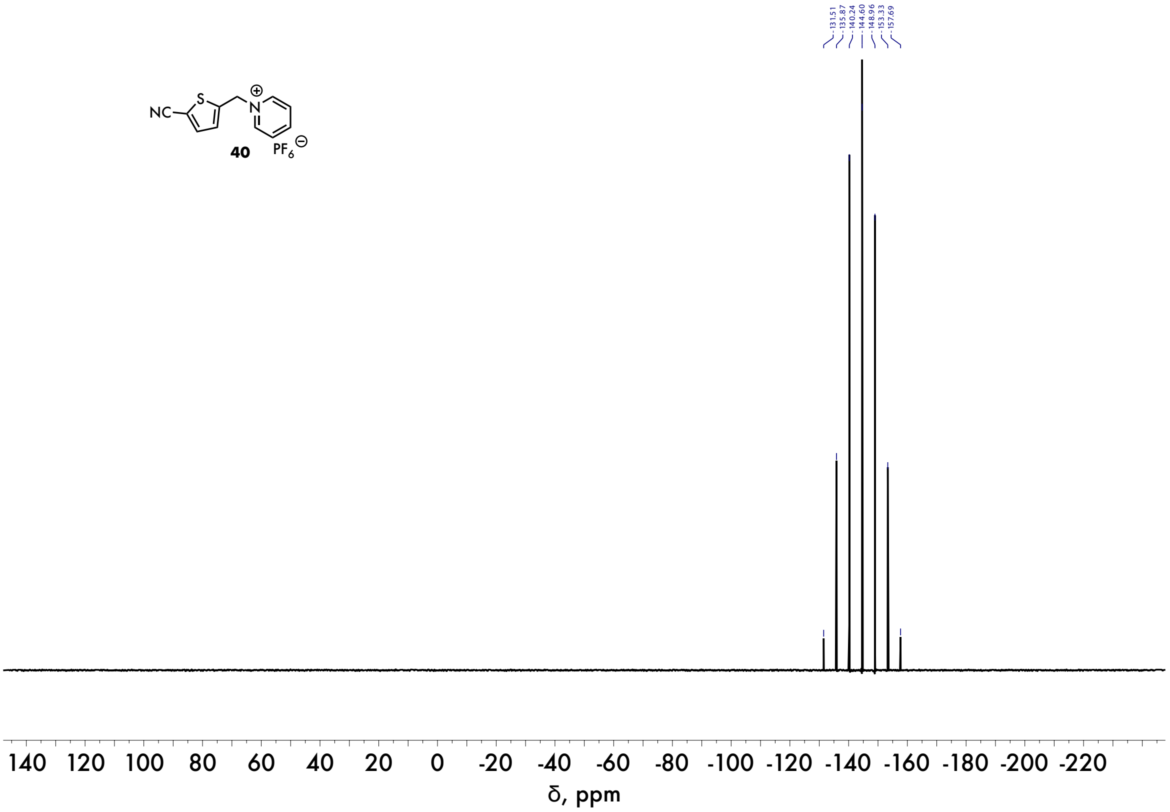

Figure S178. ^{31}P NMR (162 MHz, CD_3CN) spectrum of **40**.

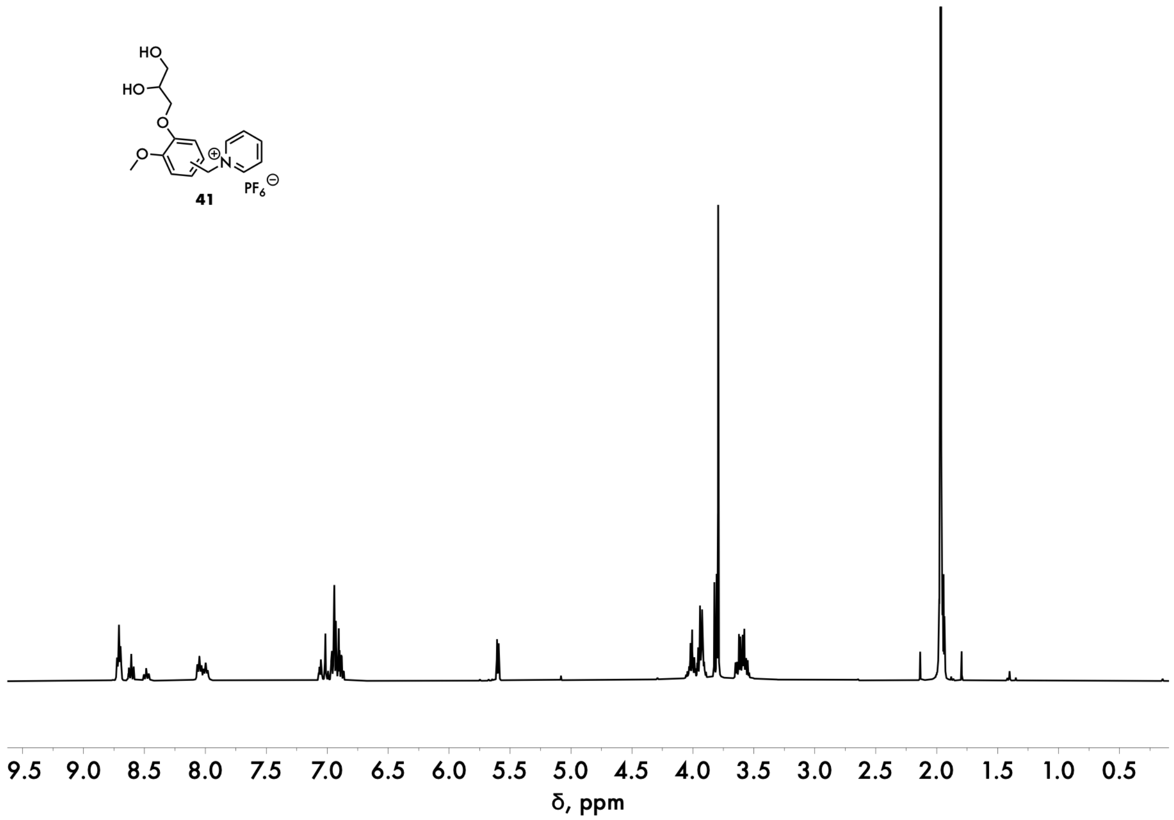

Figure S179. ^{1}H NMR (400 MHz, CD_3CN) spectrum of **41** (crude).

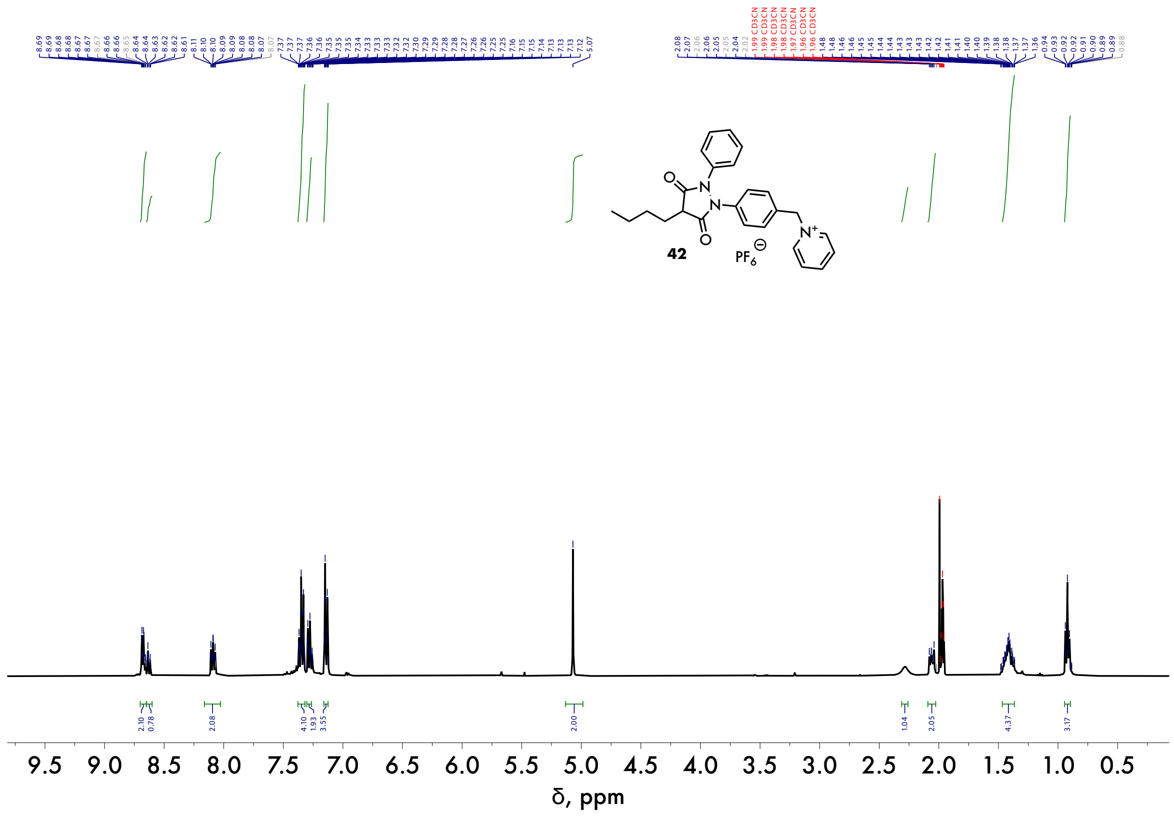

Figure S180. ^{1}H NMR (400 MHz, CD_3CN) spectrum of **42**.

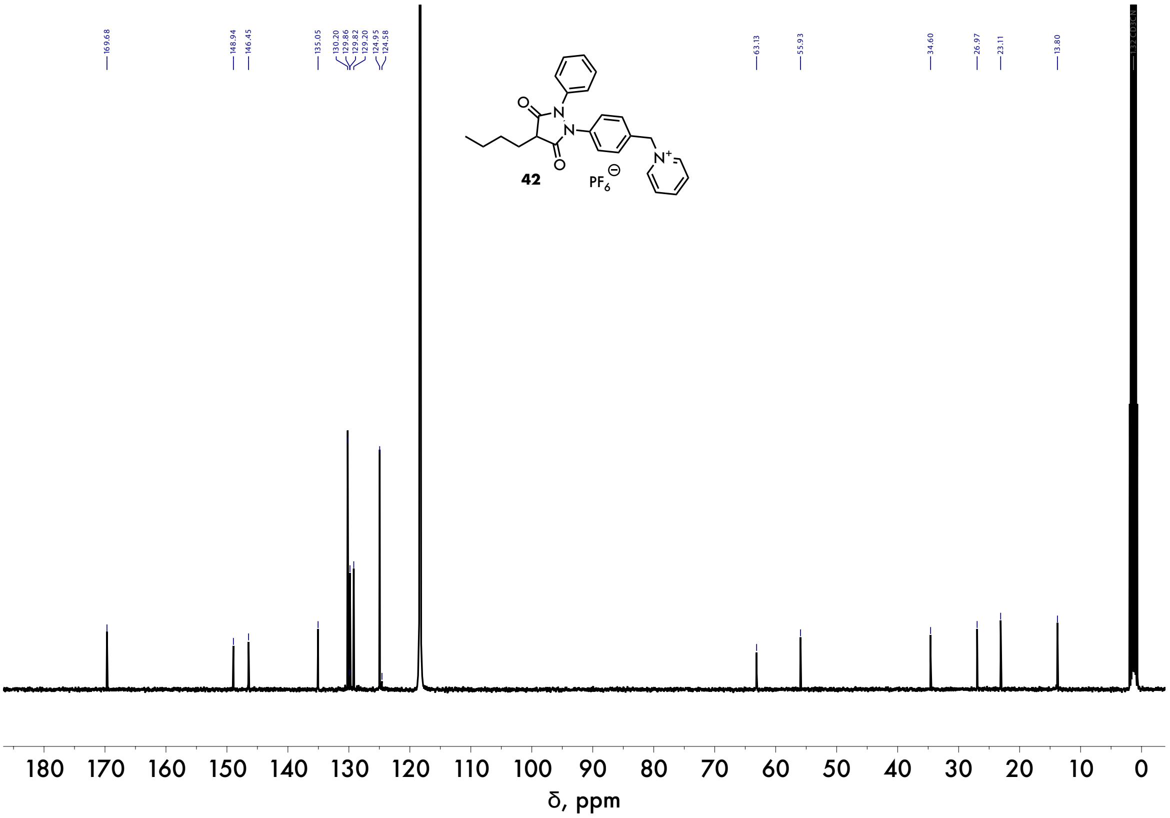

Figure S181. ^{13}C NMR (101 MHz, CD_3CN) spectrum of **42**.

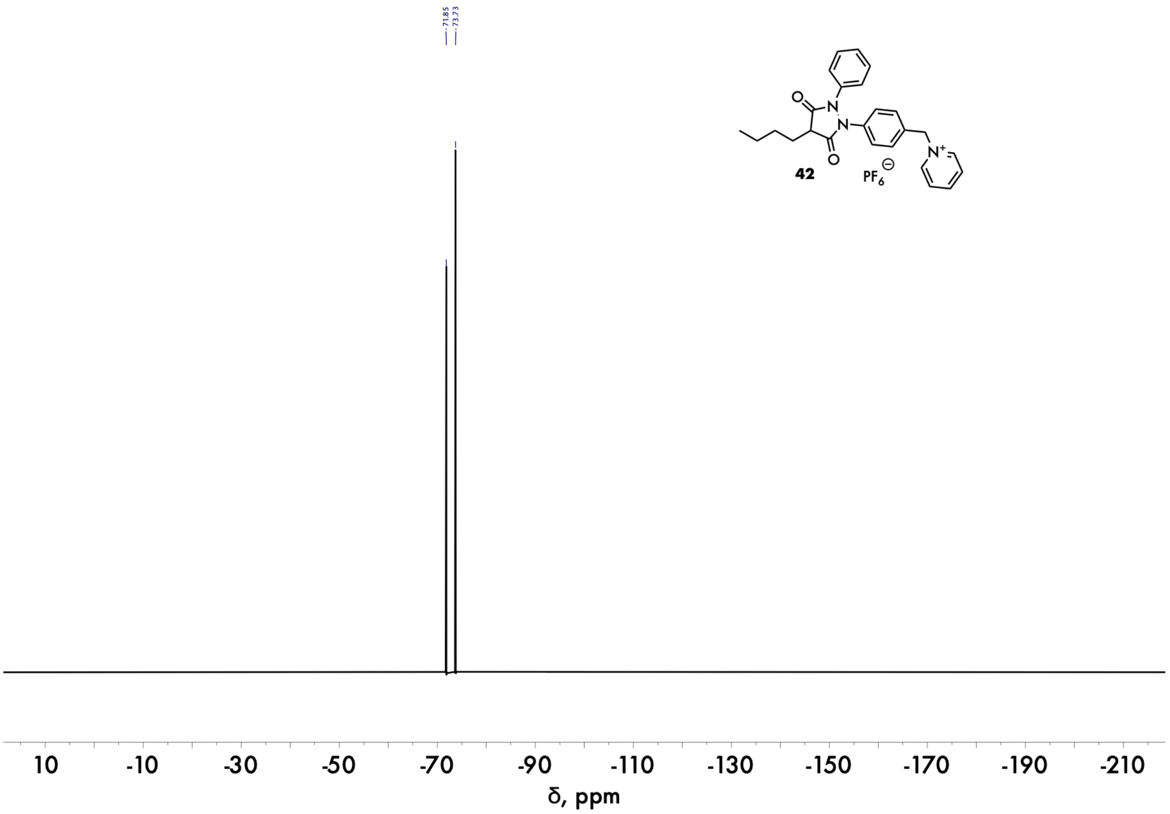

Figure S182. ^{19}F NMR (376 MHz, CD_3CN) spectrum of **42**.

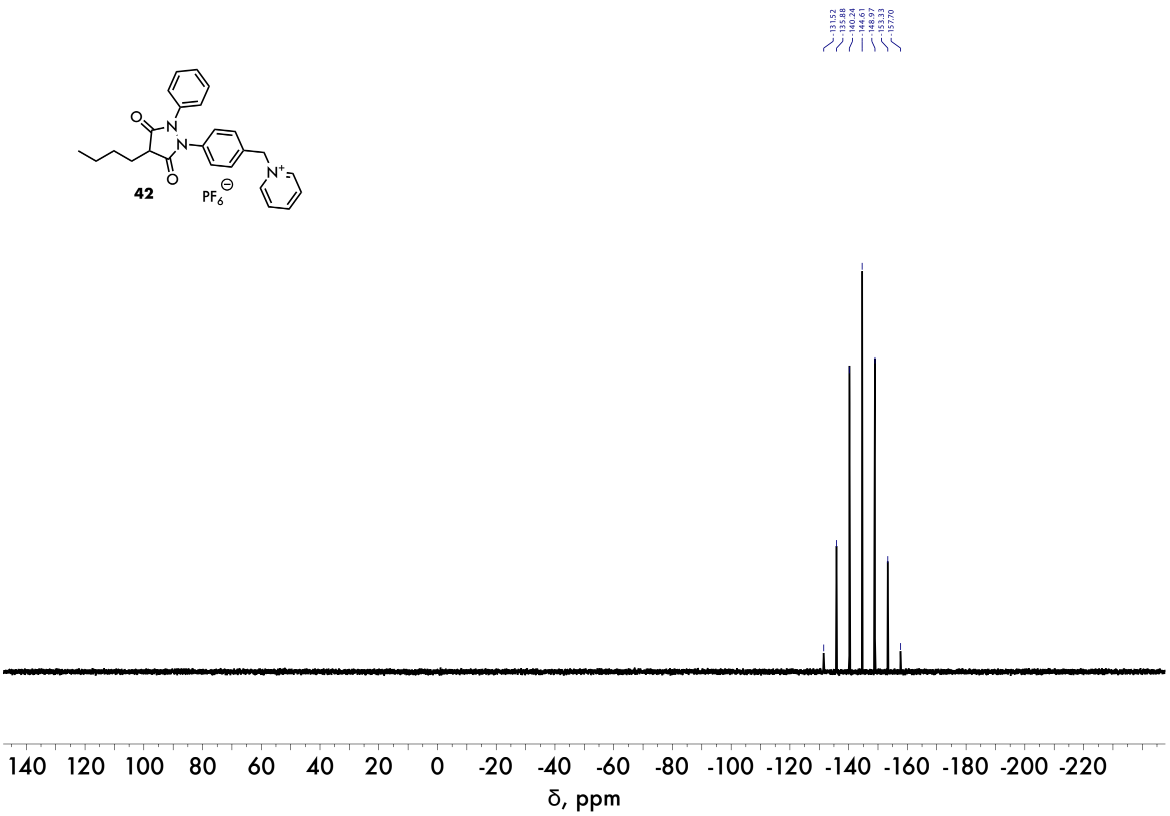

Figure S183. ^{31}P NMR (162 MHz, CD_3CN) spectrum of **42**.

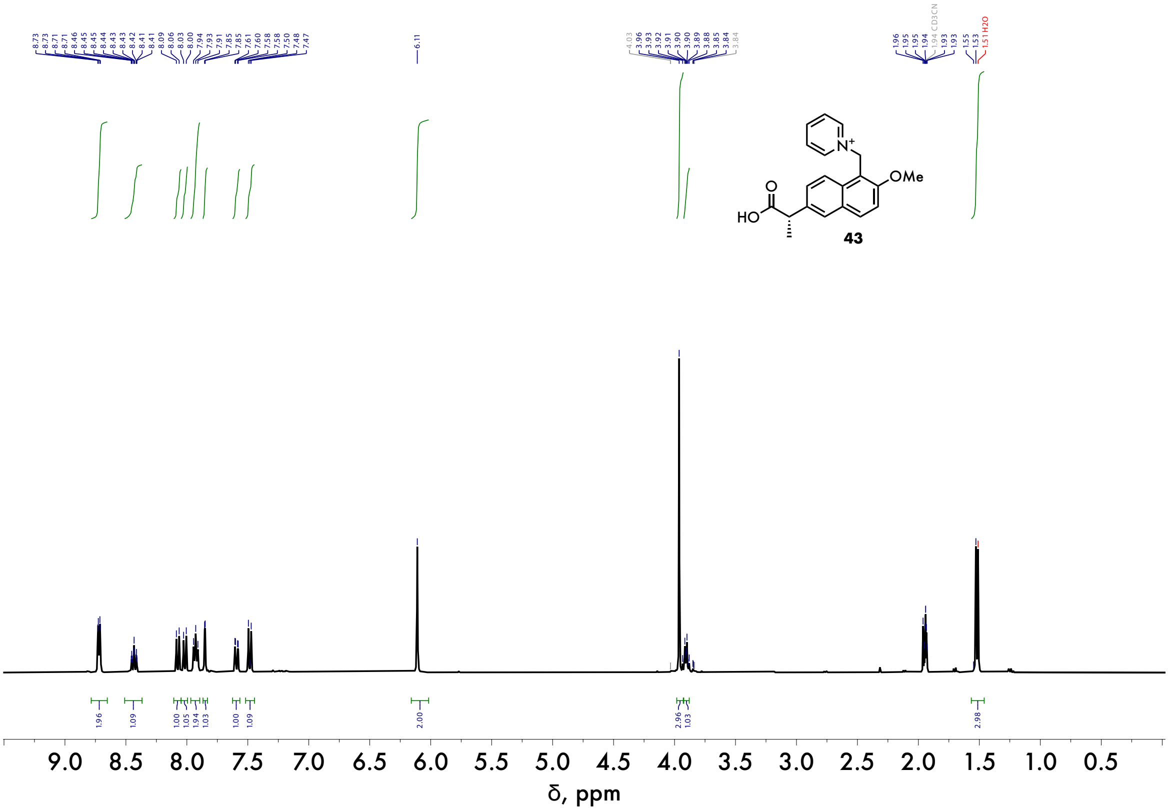

Figure S184. ^{1}H NMR (400 MHz, CD_3CN) spectrum of **43**.

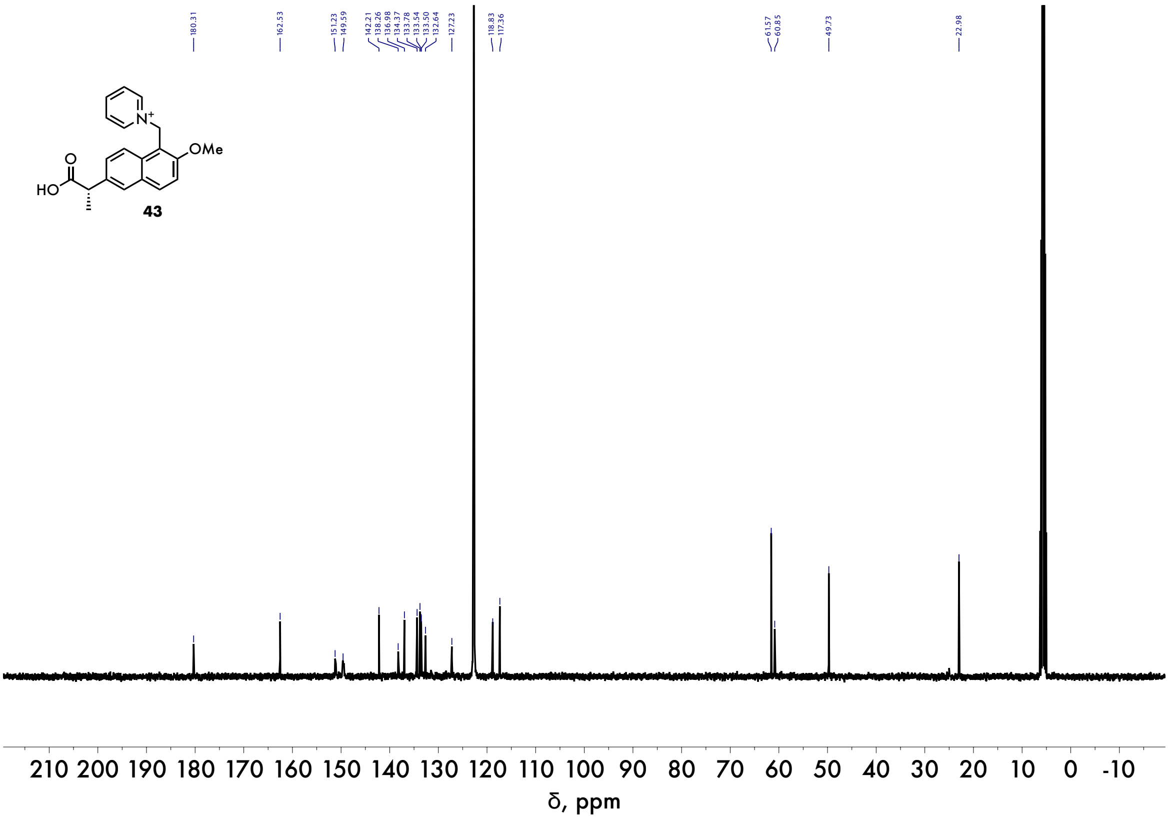

Figure S185. ^{13}C NMR (101 MHz, CD_3CN) spectrum of **43**.

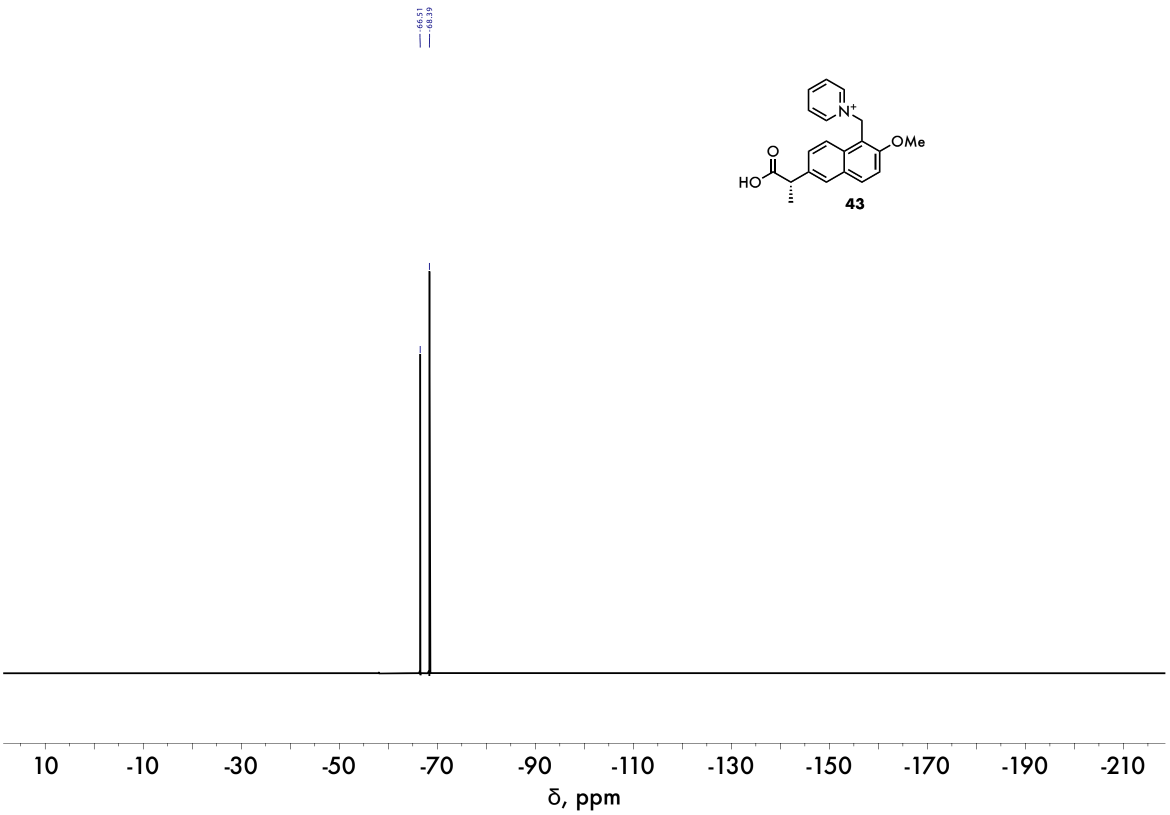

Figure S186. ^{19}F NMR (376 MHz, CD_3CN) spectrum of **43**.

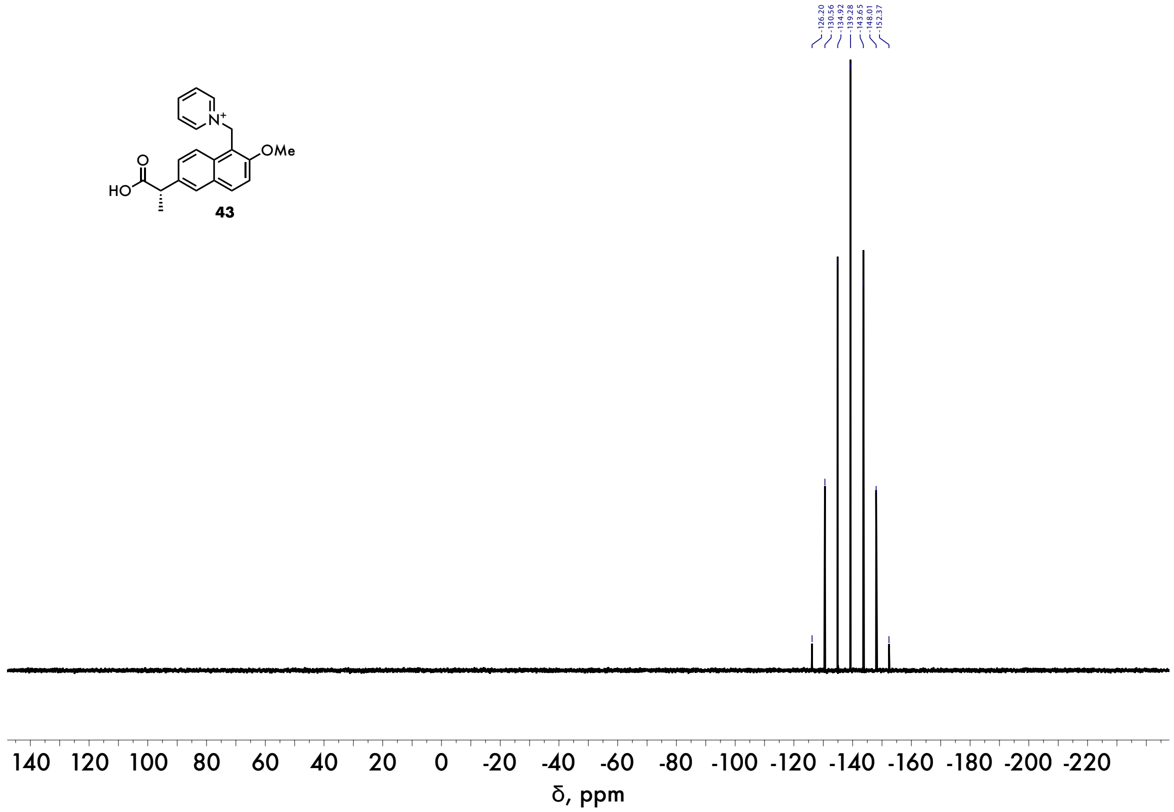

Figure S187. ^{31}P NMR (162 MHz, CD_3CN) spectrum of **43**.

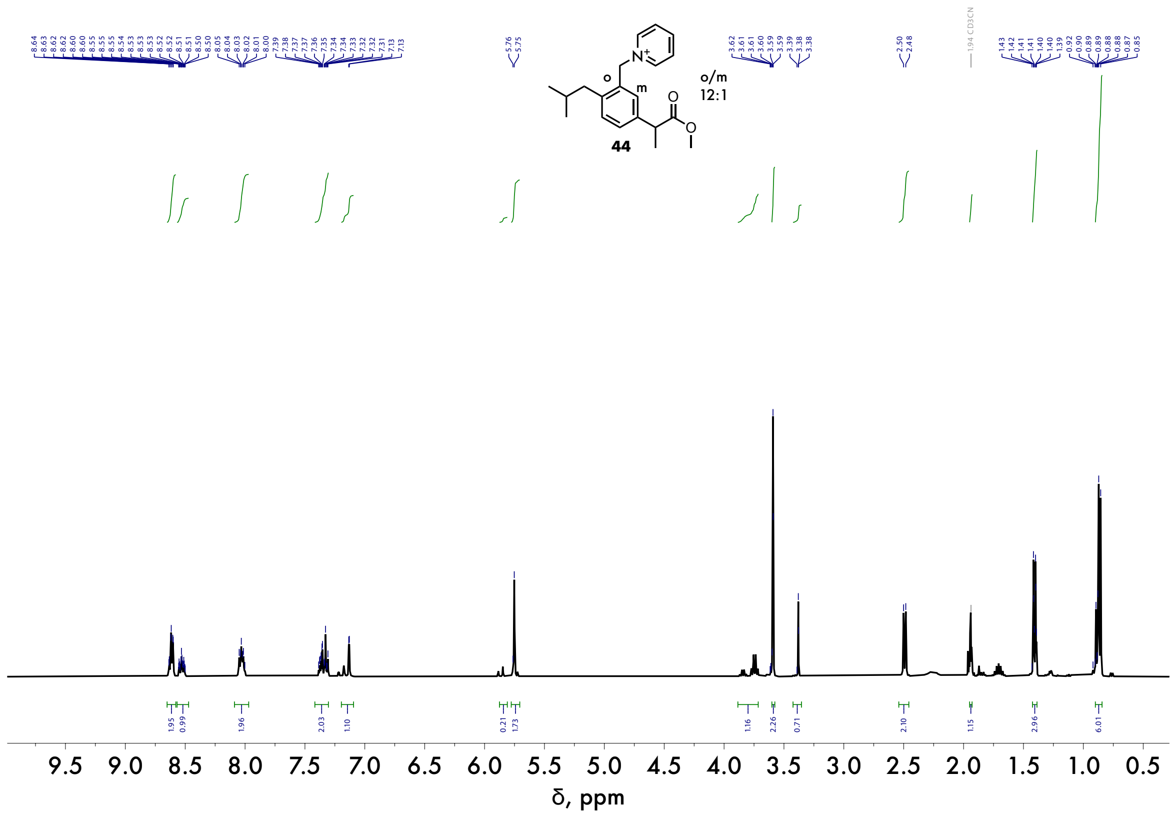

Figure S188. ^{1}H NMR (400 MHz, CD_3CN) spectrum of **44**.

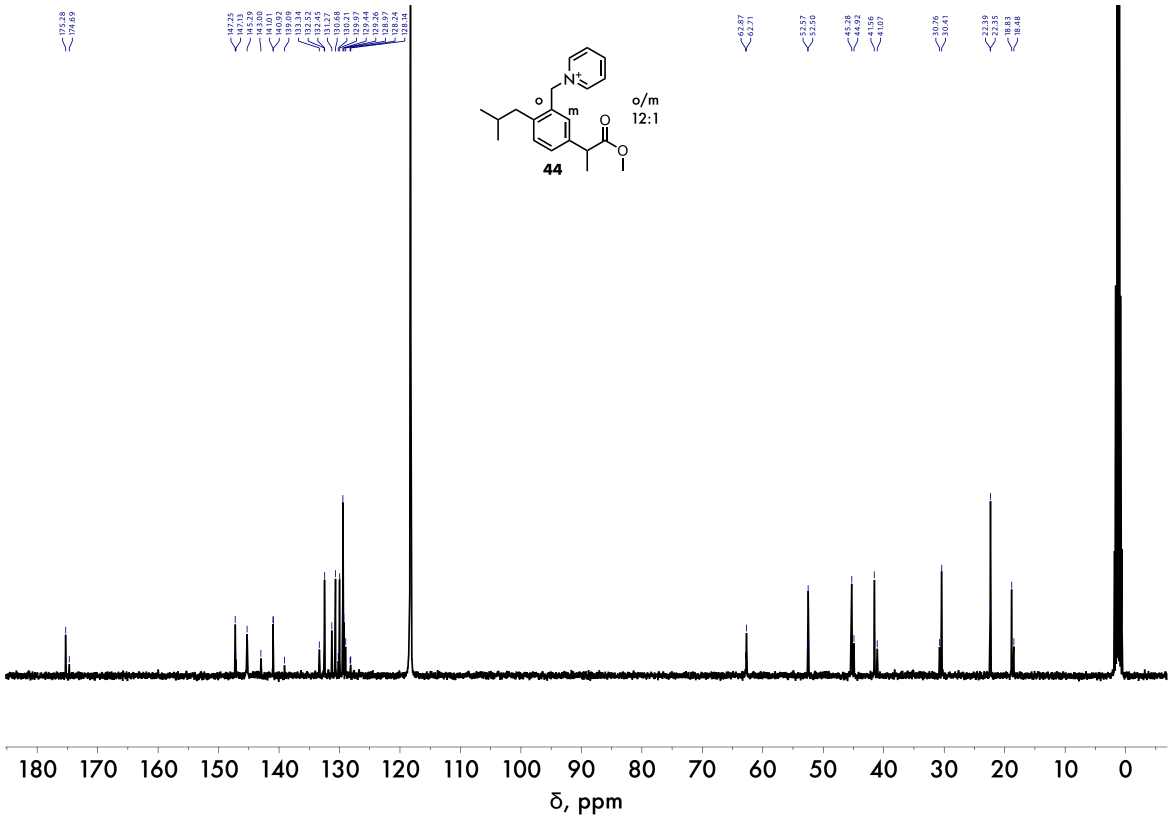

Figure S189. ^{13}C NMR (101 MHz, CD_3CN) spectrum of **44**.

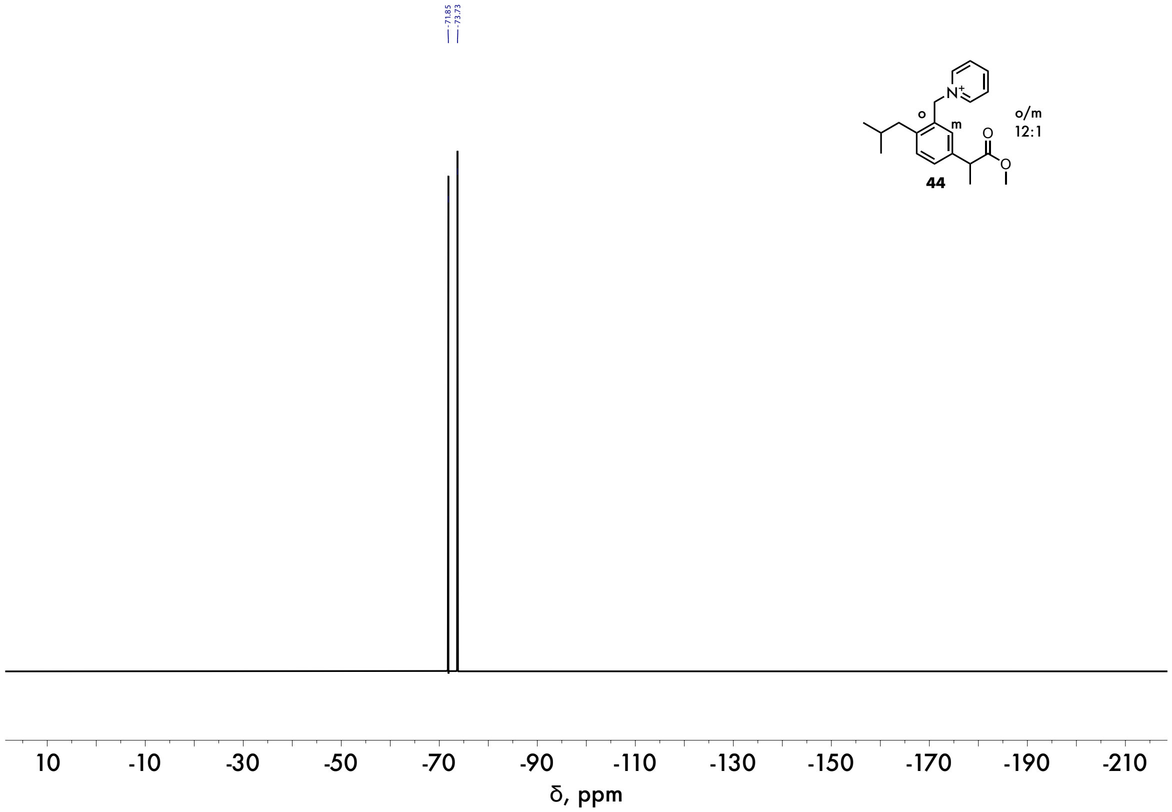

Figure S190. ^{19}F NMR (376 MHz, CD_3CN) spectrum of **44**.

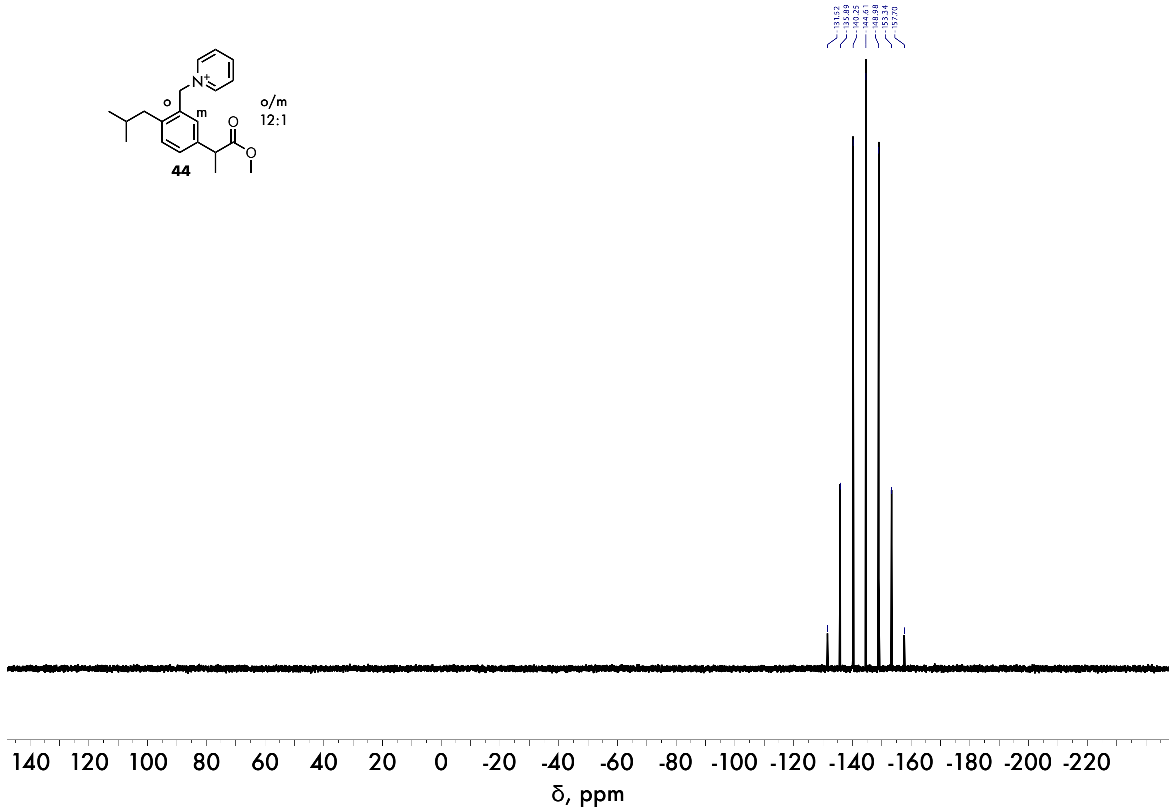

Figure S191. ^{31}P NMR (162 MHz, CD_3CN) spectrum of **44**.

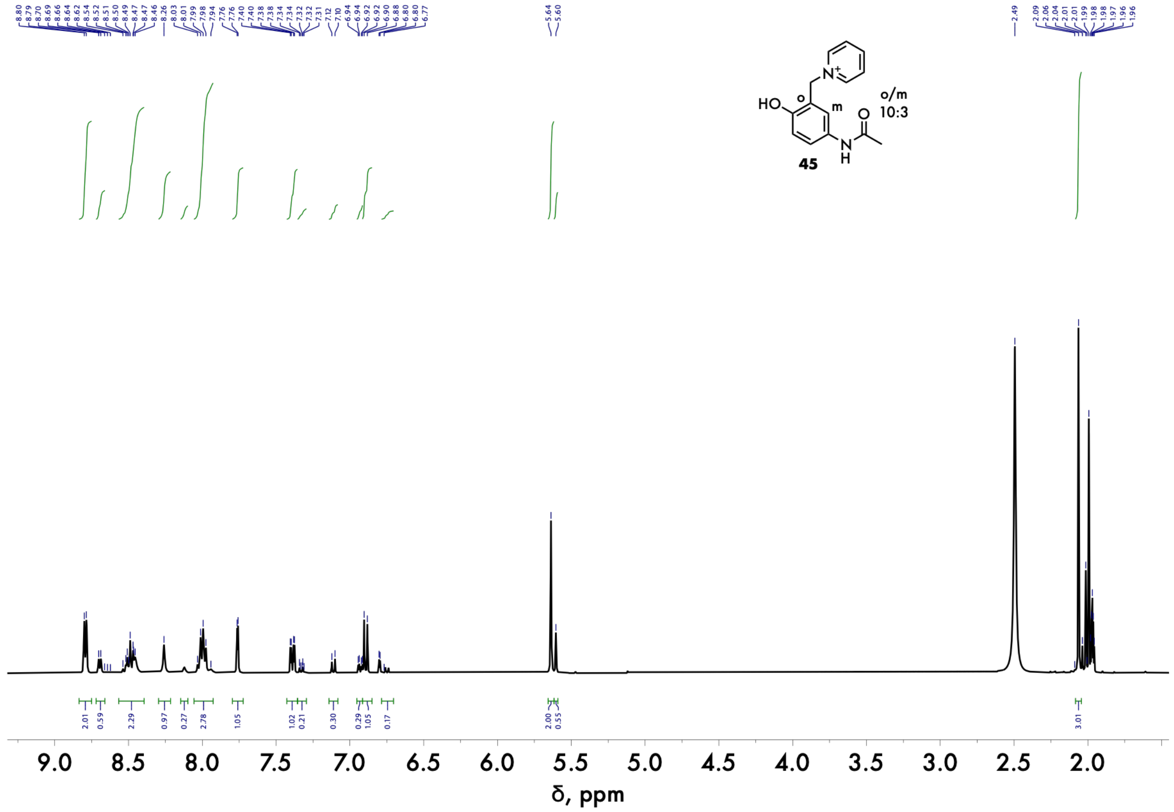

Figure S192. ^{1}H NMR (400 MHz, $CDCl_3$) spectrum of **45**.

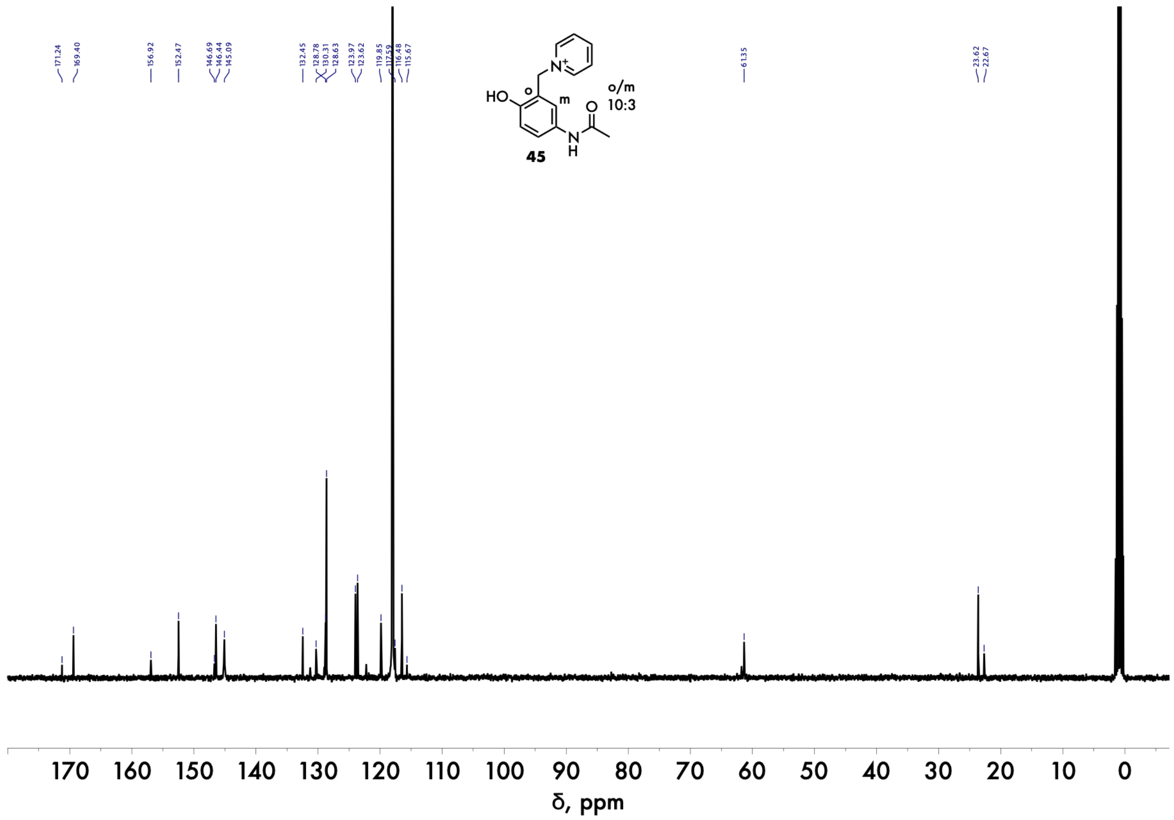

Figure S193. ^{13}C NMR (101 MHz, CD_3CN) spectrum of **45**.

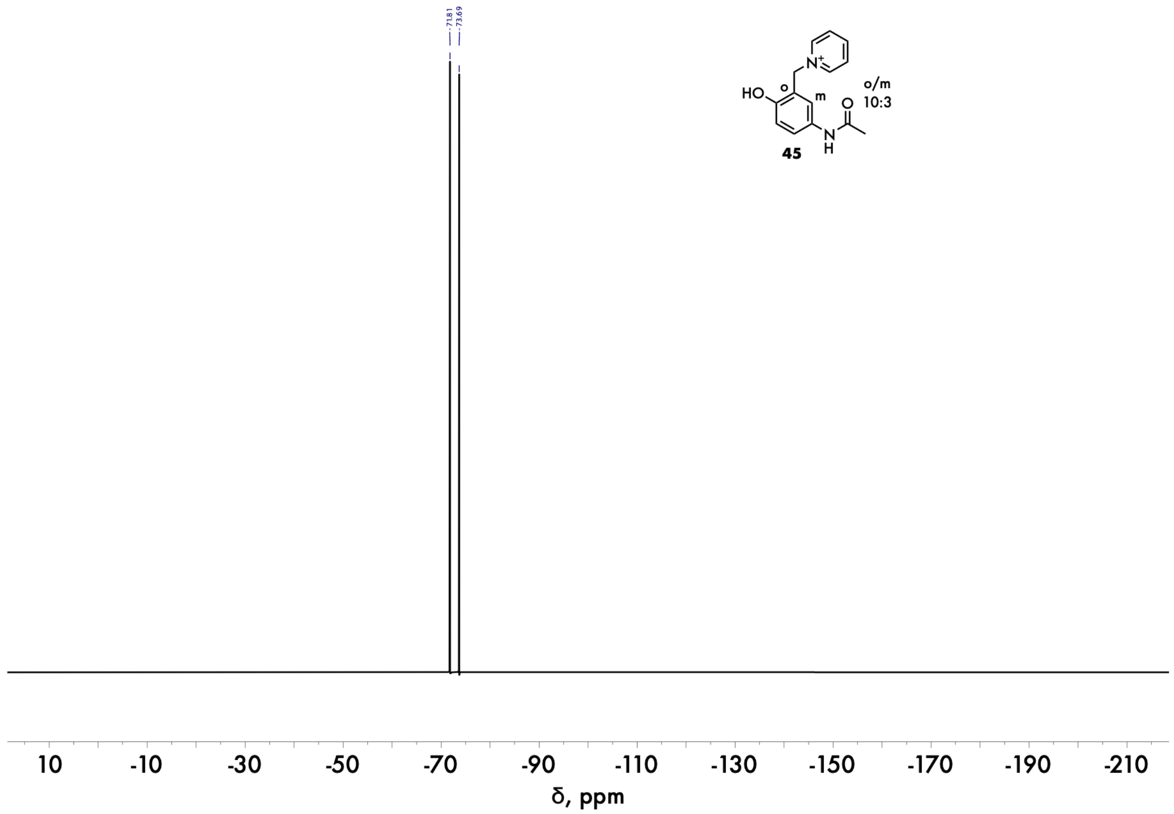

Figure S194. ^{19}F NMR (376 MHz, CD_3CN) spectrum of **45**.

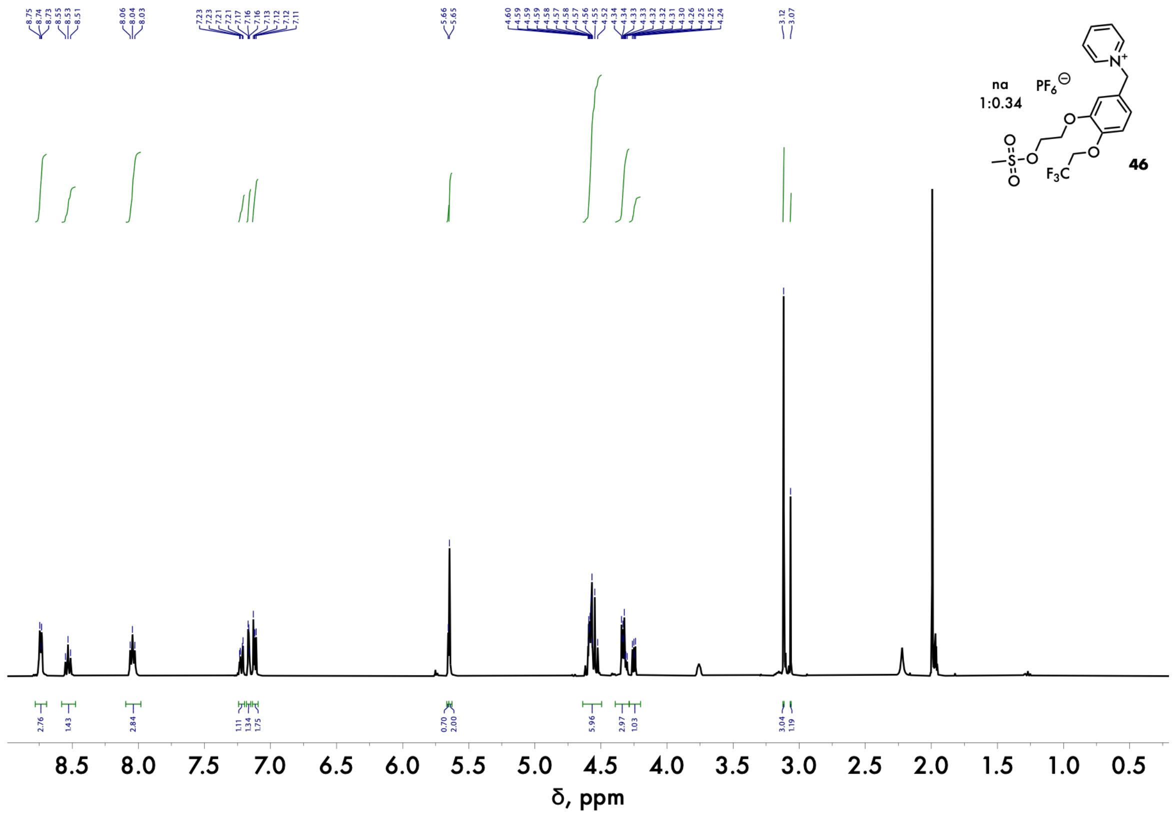

Figure S195. ^{1}H NMR (400 MHz, CD_3CN) spectrum of **46**.

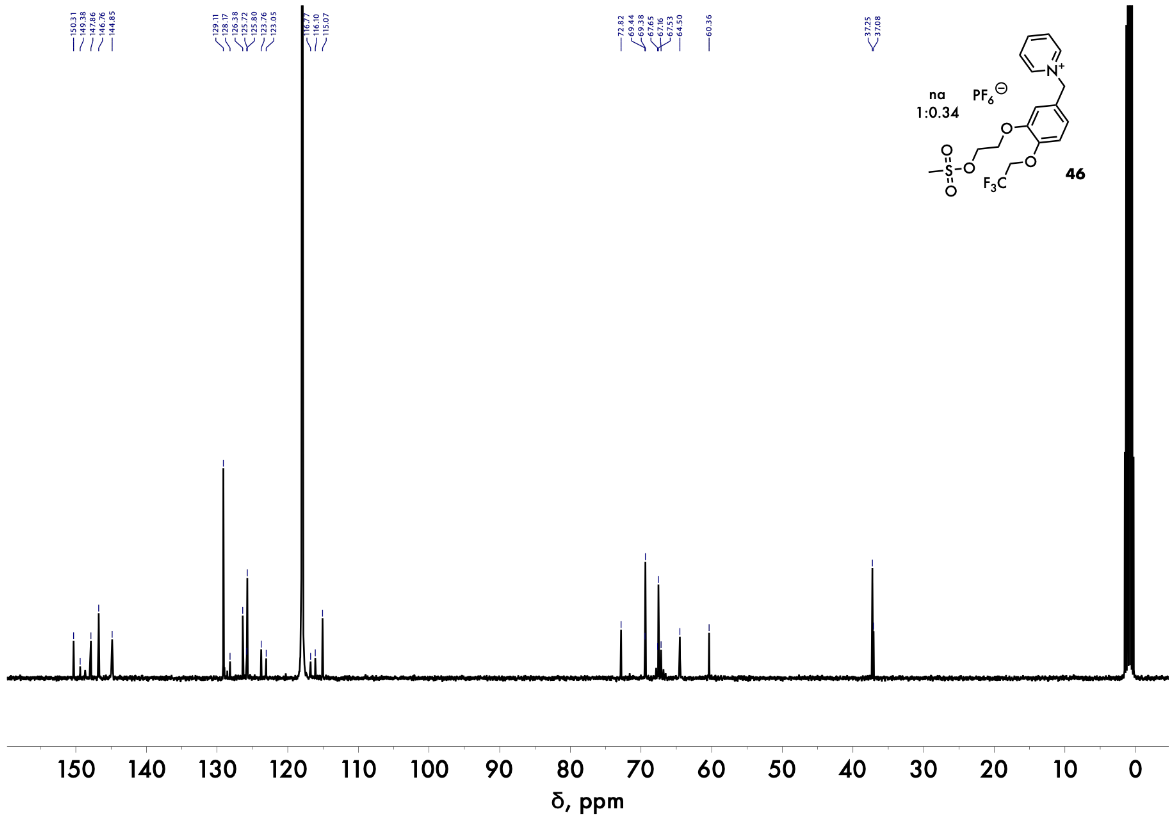

Figure S196. ^{13}C NMR (101 MHz, CD_3CN) spectrum of **46**.

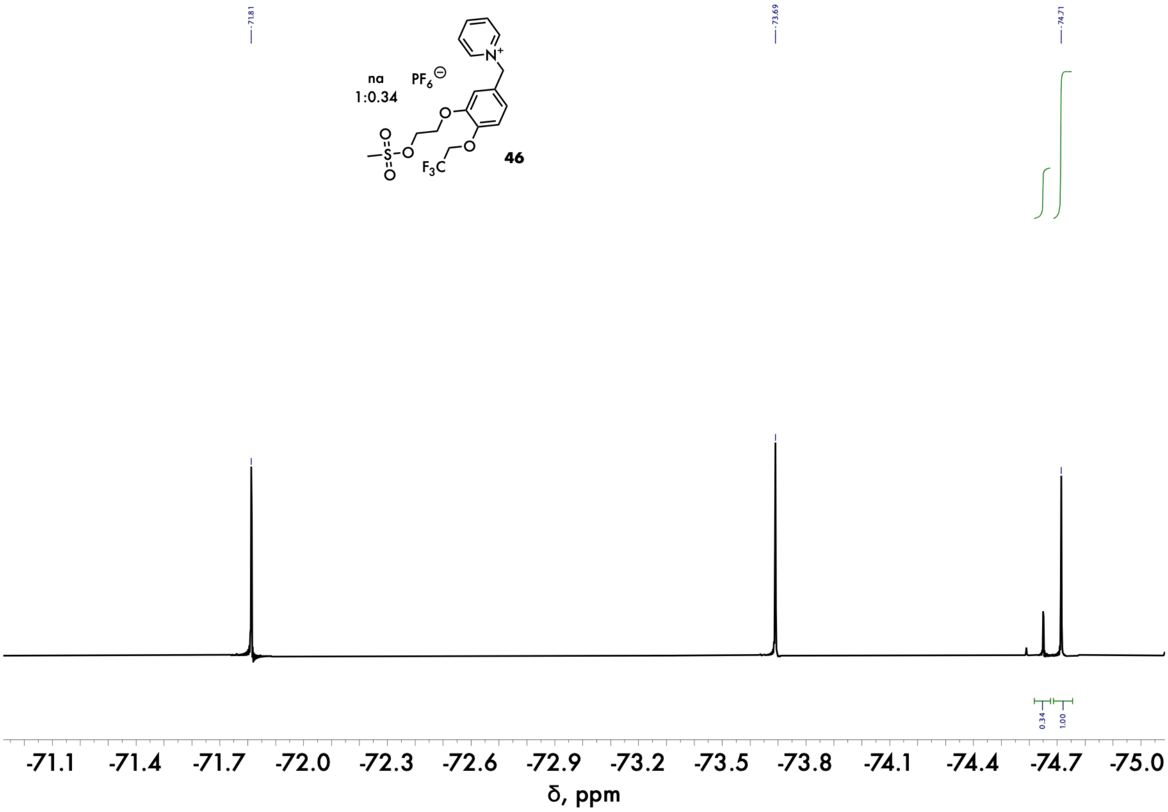

Figure S197. ^{19}F NMR (376 MHz, CD_3CN) spectrum of **46**.

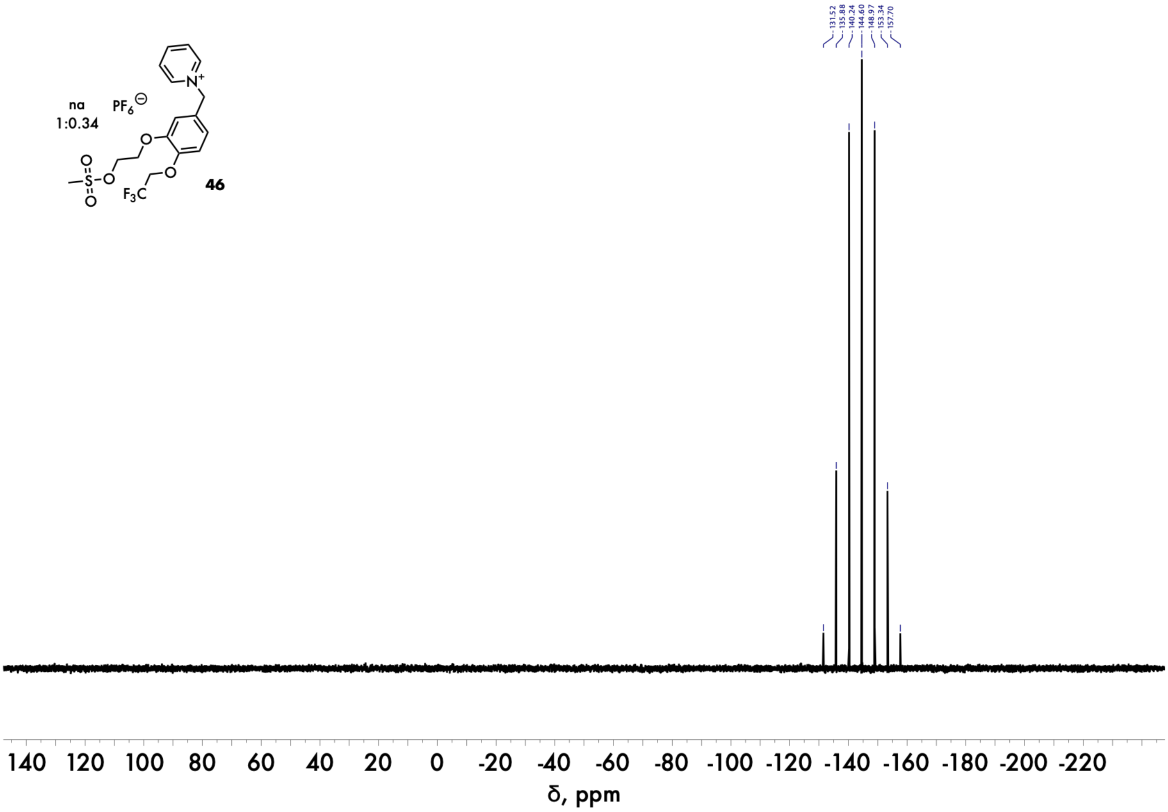

Figure S198. ^{31}P NMR (162 MHz, CD_3CN) spectrum of **44**.

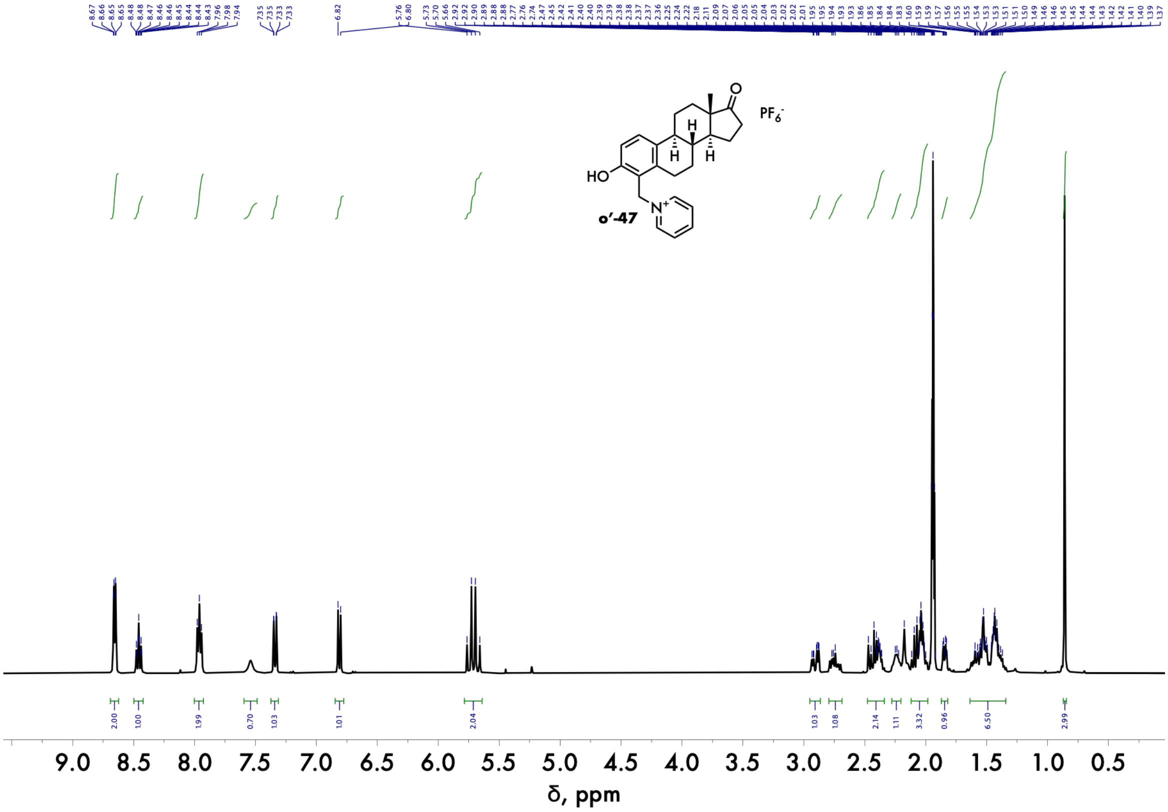

Figure S199. ^{1}H NMR (400 MHz, CD_3CN) spectrum of **o’-47**.

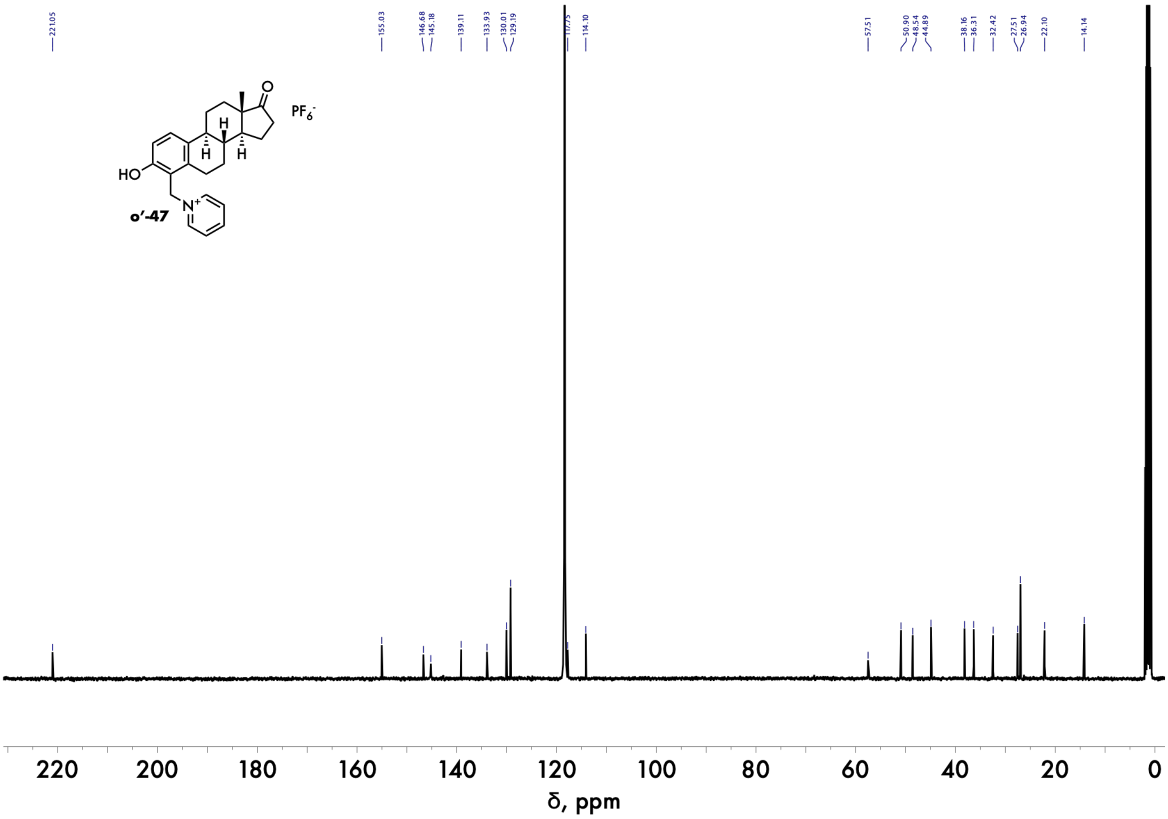

Figure S200. ^{13}C NMR (101 MHz, CD_3CN) spectrum of **o’-47**.

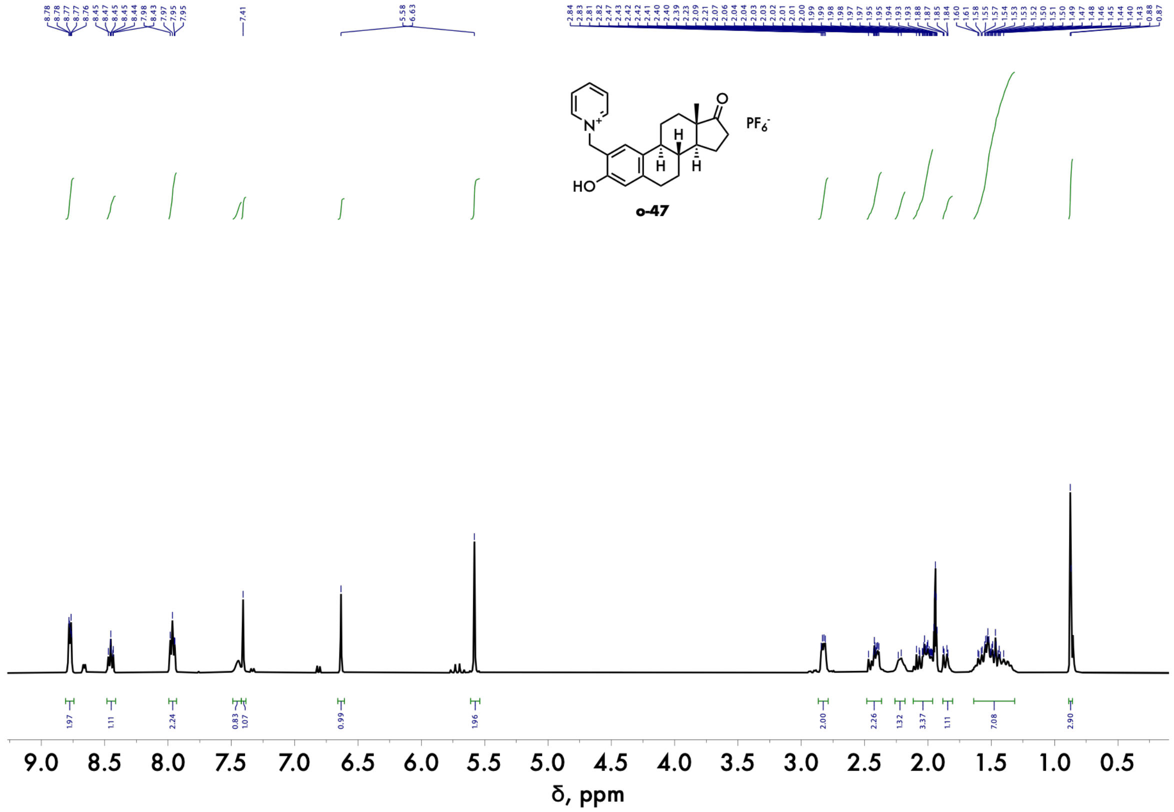

Figure S201. ^{1}H NMR (400 MHz, CD_3CN) spectrum of **o-47**.

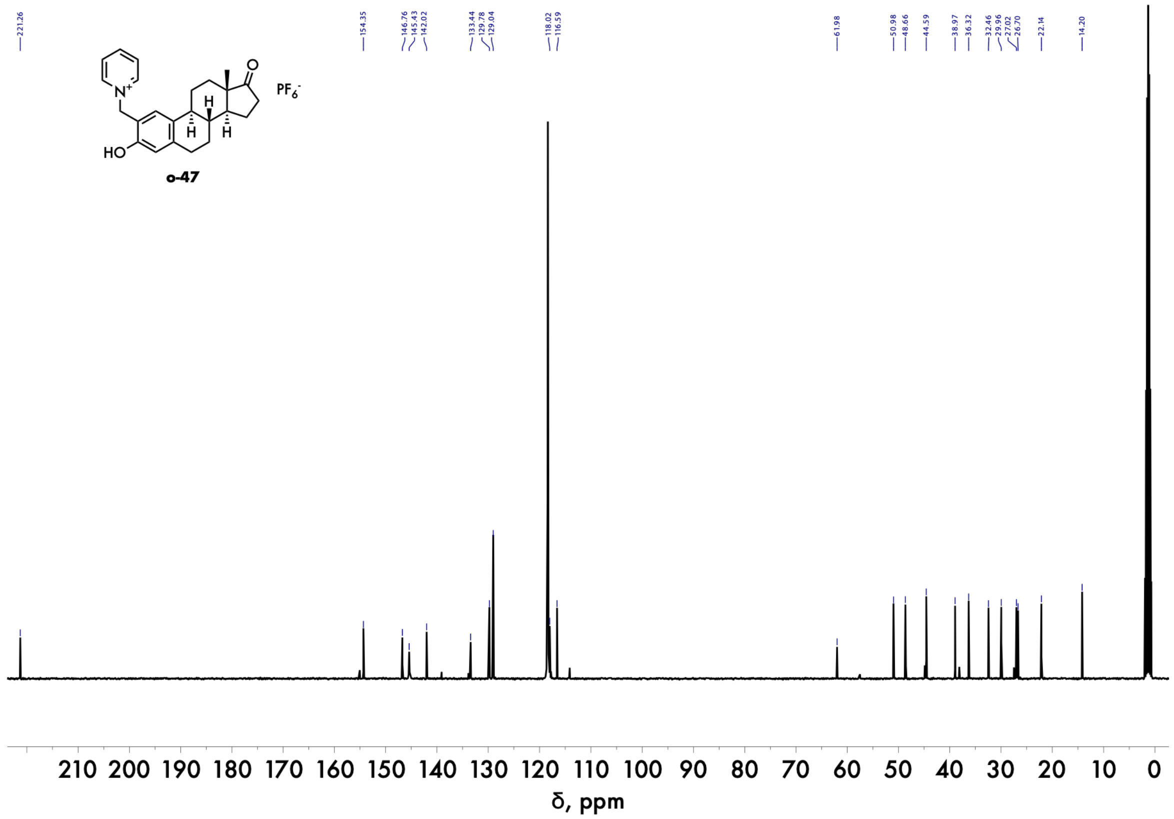

Figure S202. ^{13}C NMR (101 MHz, CD_3CN) spectrum of **o-47**.

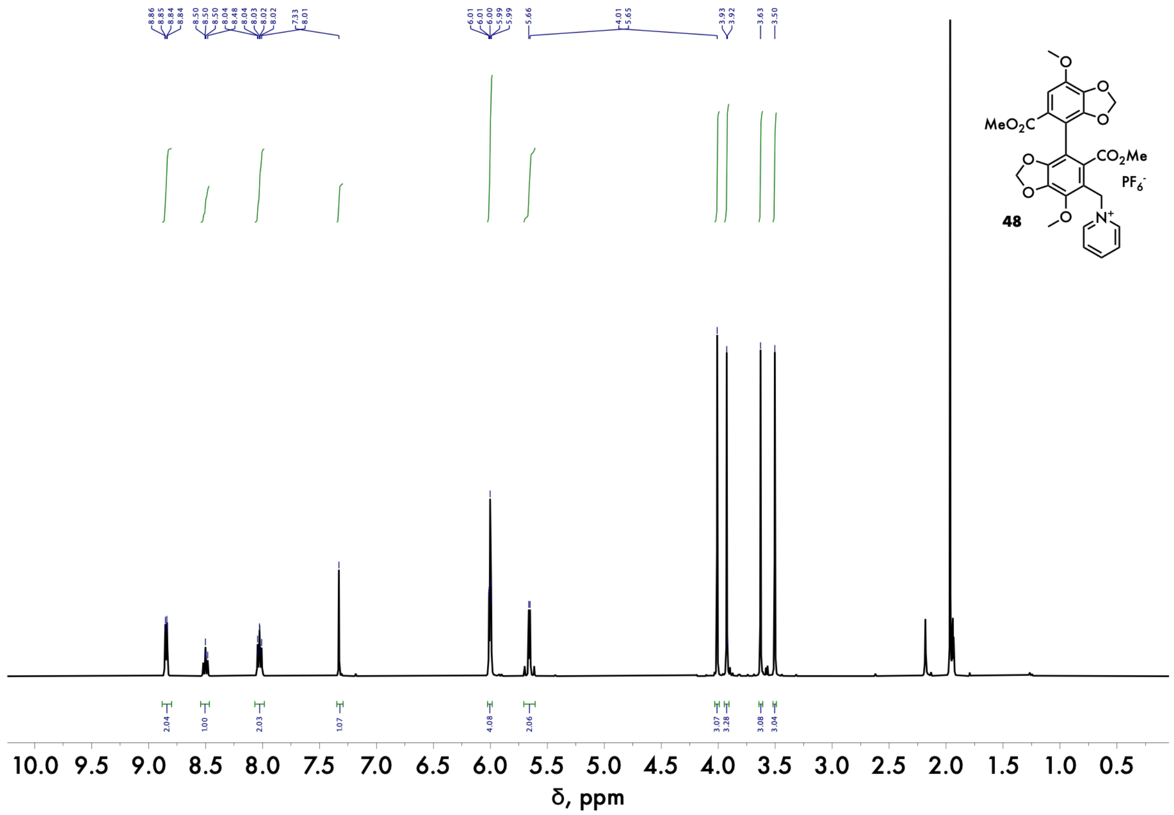

Figure S203. ^{1}H NMR (400 MHz, CD_3CN) spectrum of **48**.

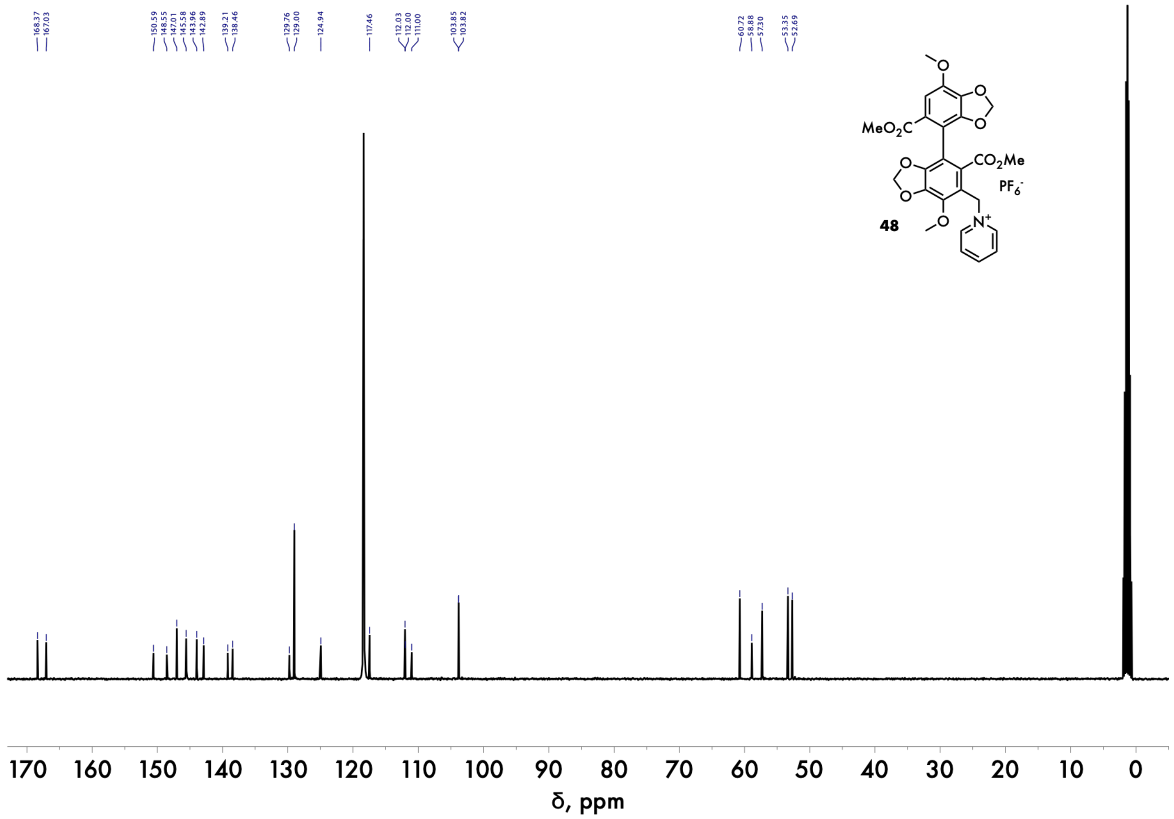

Figure S204. ^{13}C NMR (101 MHz, CD_3CN) spectrum of **48**.

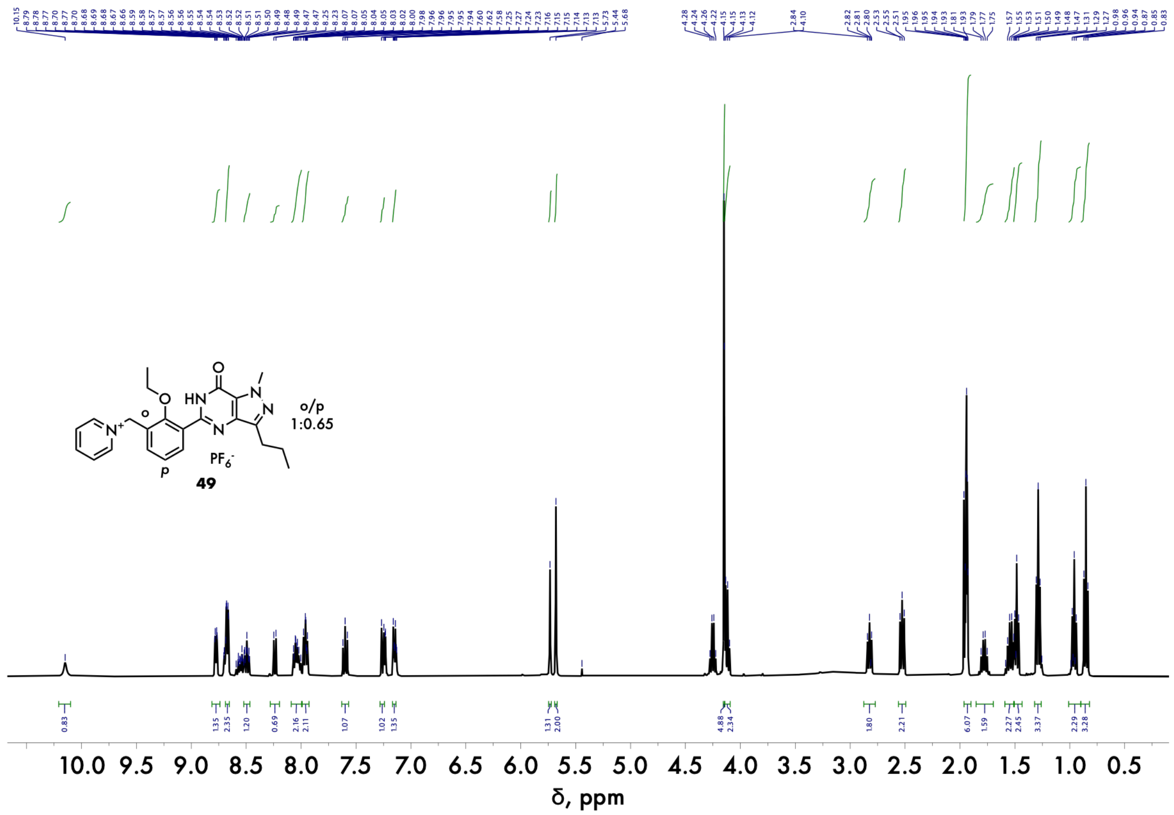

Figure S205. ^{1}H NMR (400 MHz, CD_3CN) spectrum of **49**.

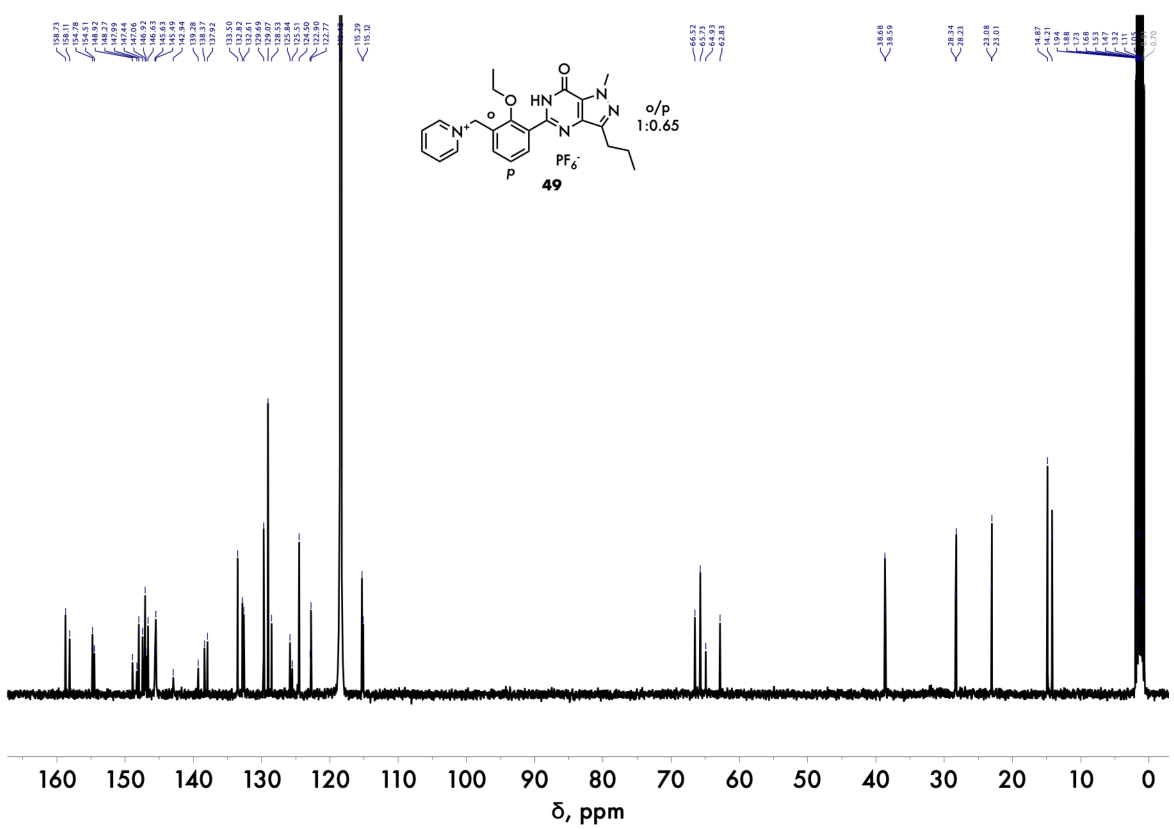

Figure S206. ^{13}C NMR (101 MHz, CD_3CN) spectrum of **49**.

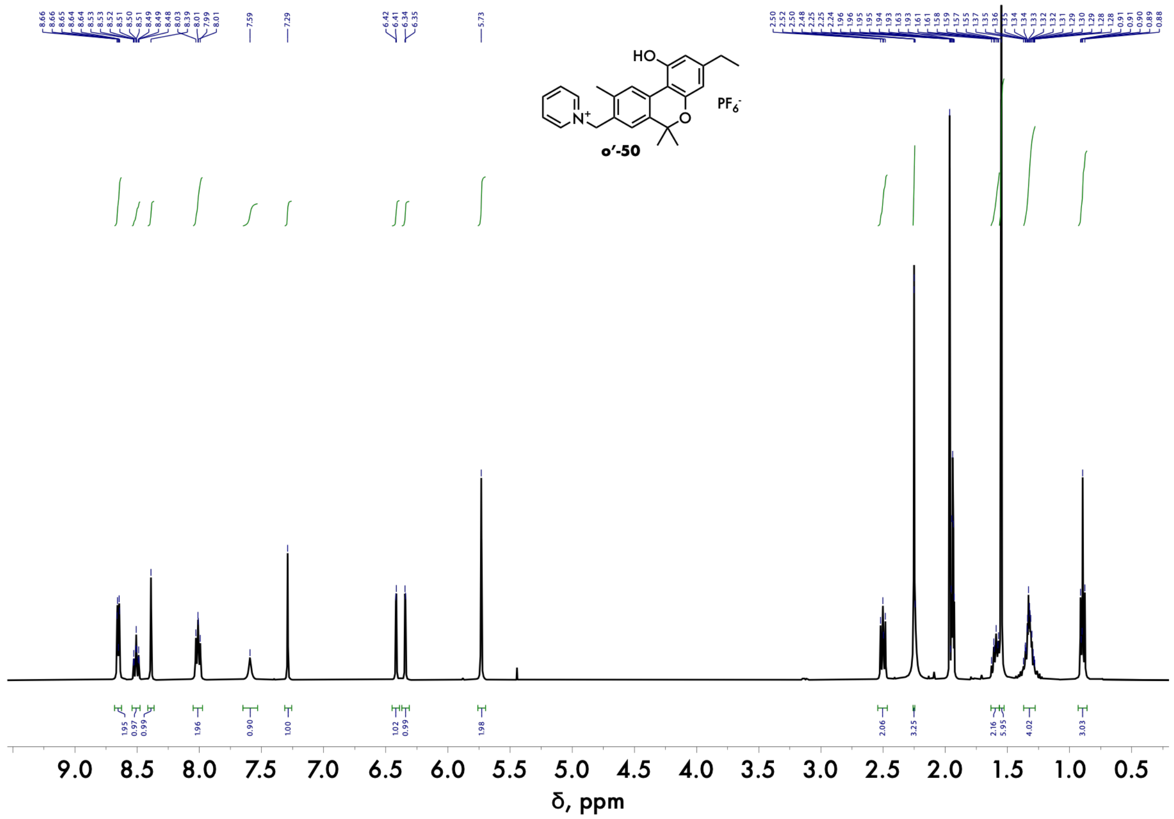

Figure S207. ^{1}H NMR (400 MHz, CD_3CN) spectrum of **o'-50**.

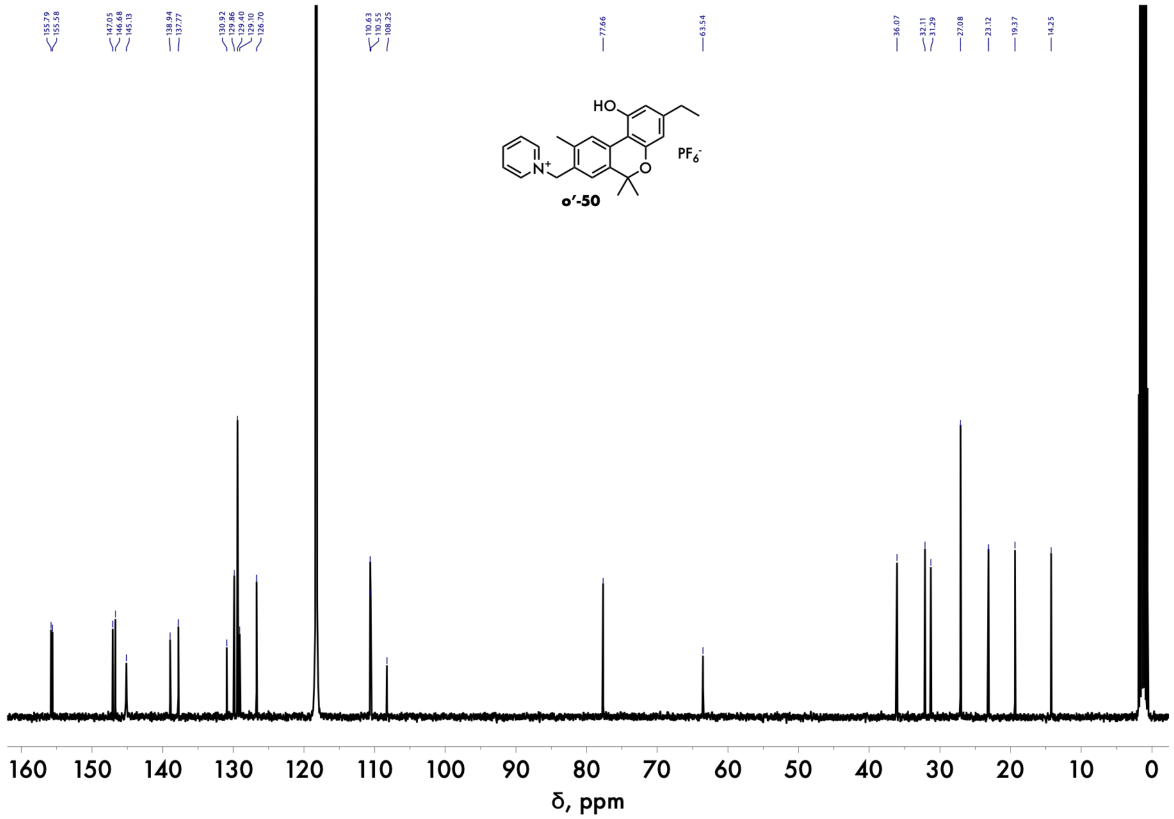

Figure S208. ^{13}C NMR (101 MHz, CD_3CN) spectrum of **o'-50**.

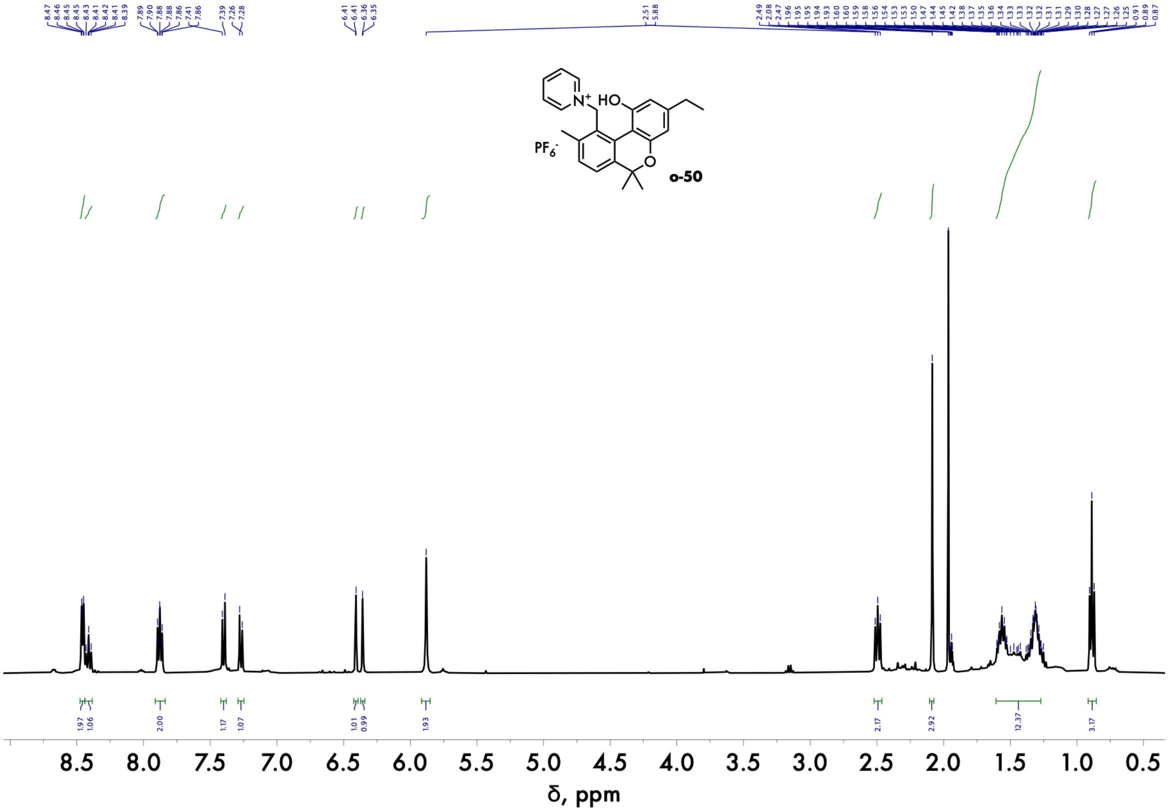

Figure S209. ^{1}H NMR (400 MHz, CD_3CN) spectrum of **o-50**.

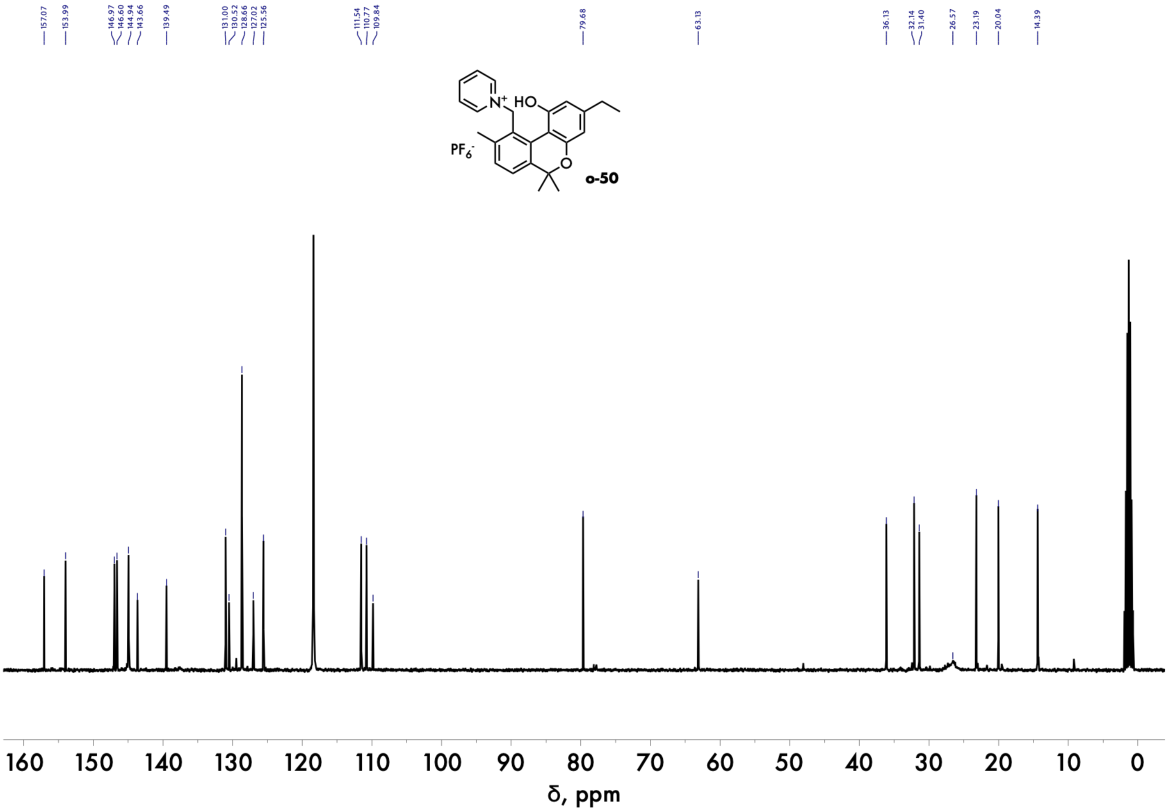

Figure S210. ^{13}C NMR (101 MHz, CD_3CN) spectrum of **o-50**.

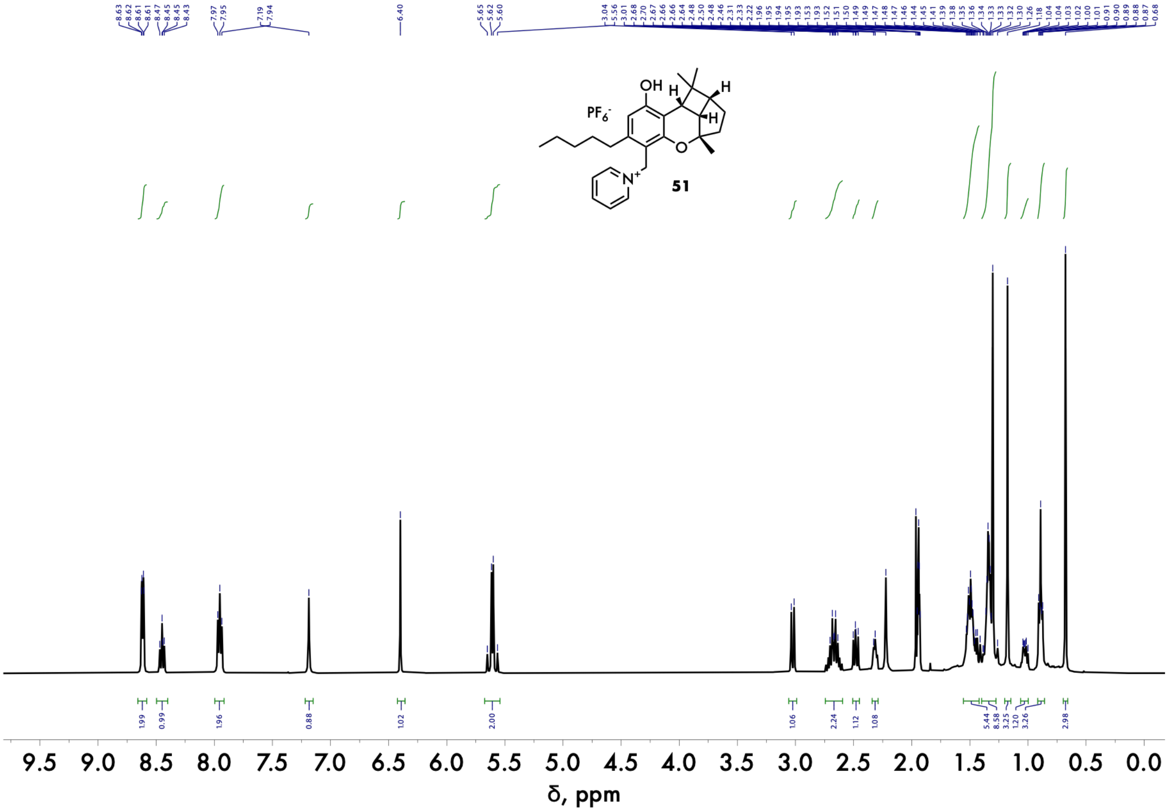

Figure S211. ^{1}H NMR (400 MHz, CD_3CN) spectrum of **51**

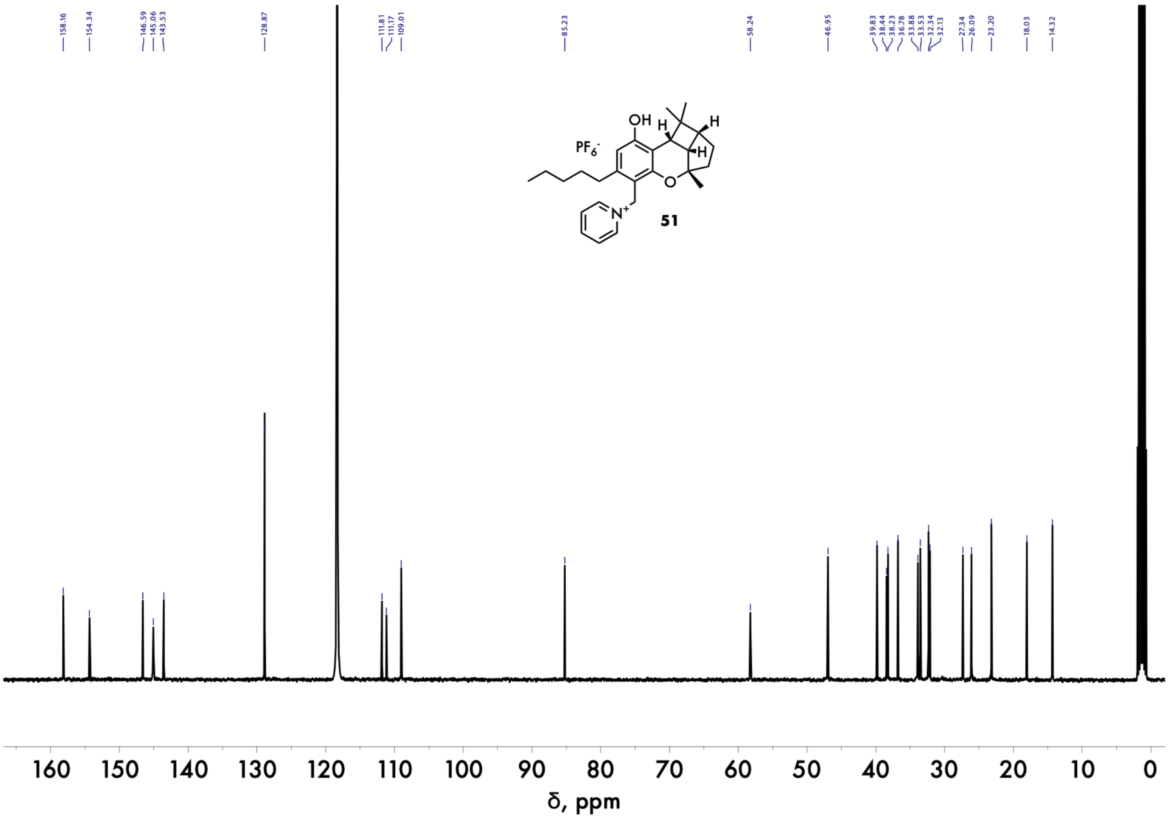

Figure S212. ^{13}C NMR (101 MHz, CD_3CN) spectrum of **51**.

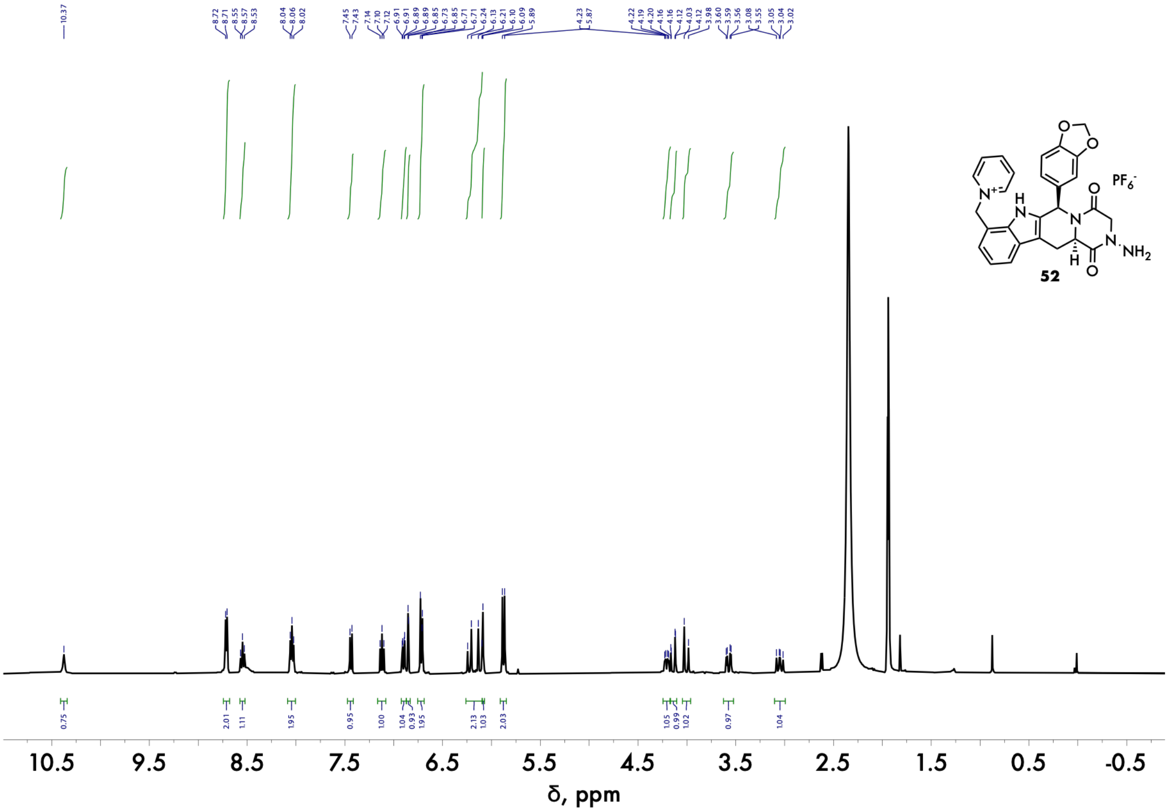

Figure S213. ^{1}H NMR (400 MHz, CD_3CN) spectrum of **52**.

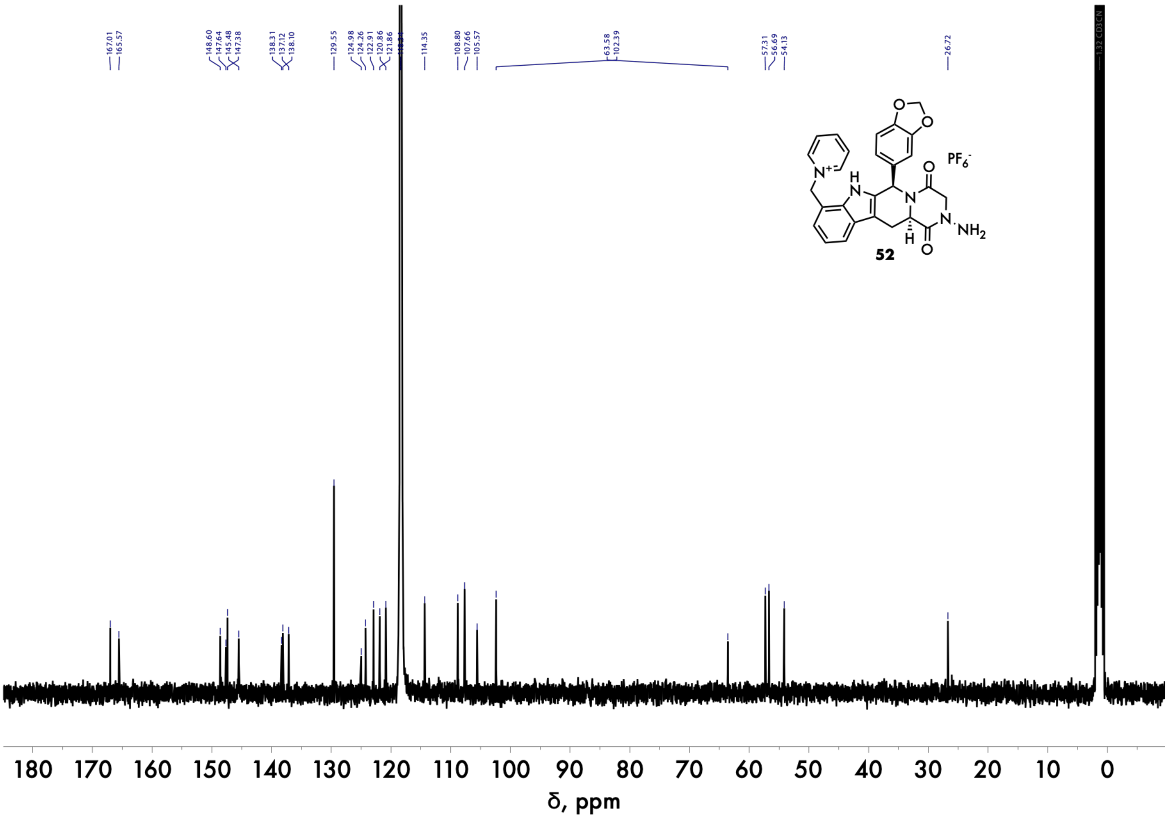

Figure S214. ^{13}C NMR (101 MHz, CD_3CN) spectrum of **52**.

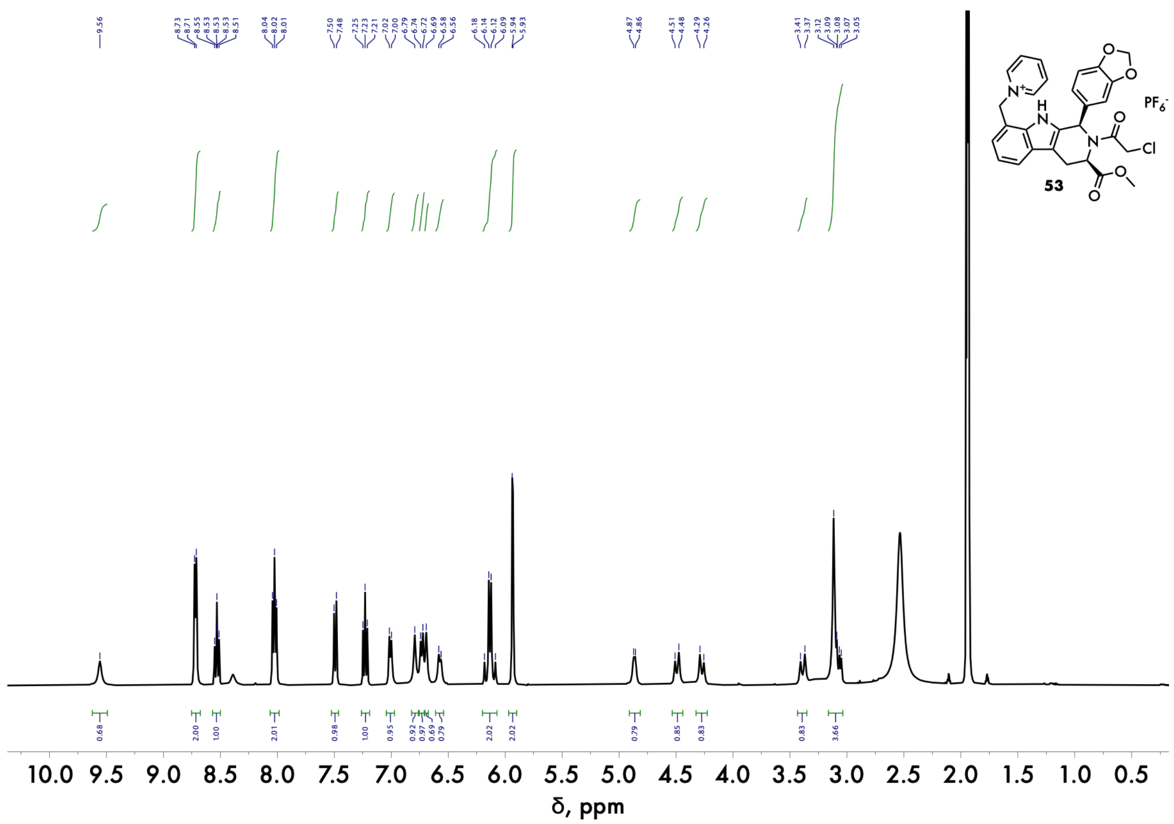

Figure S215. ^{1}H NMR (400 MHz, CD_3CN) spectrum of **53**.

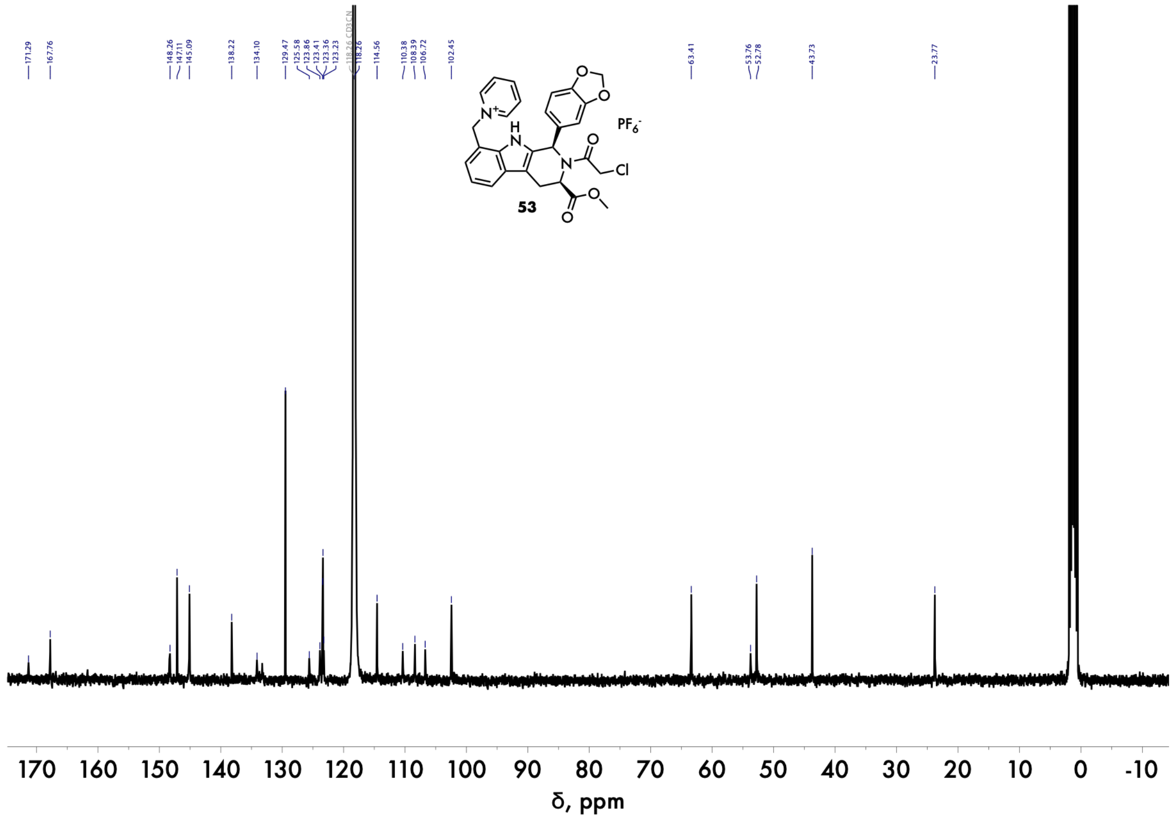

Figure S216. ^{13}C NMR (101 MHz, CD_3CN) spectrum of **53**.

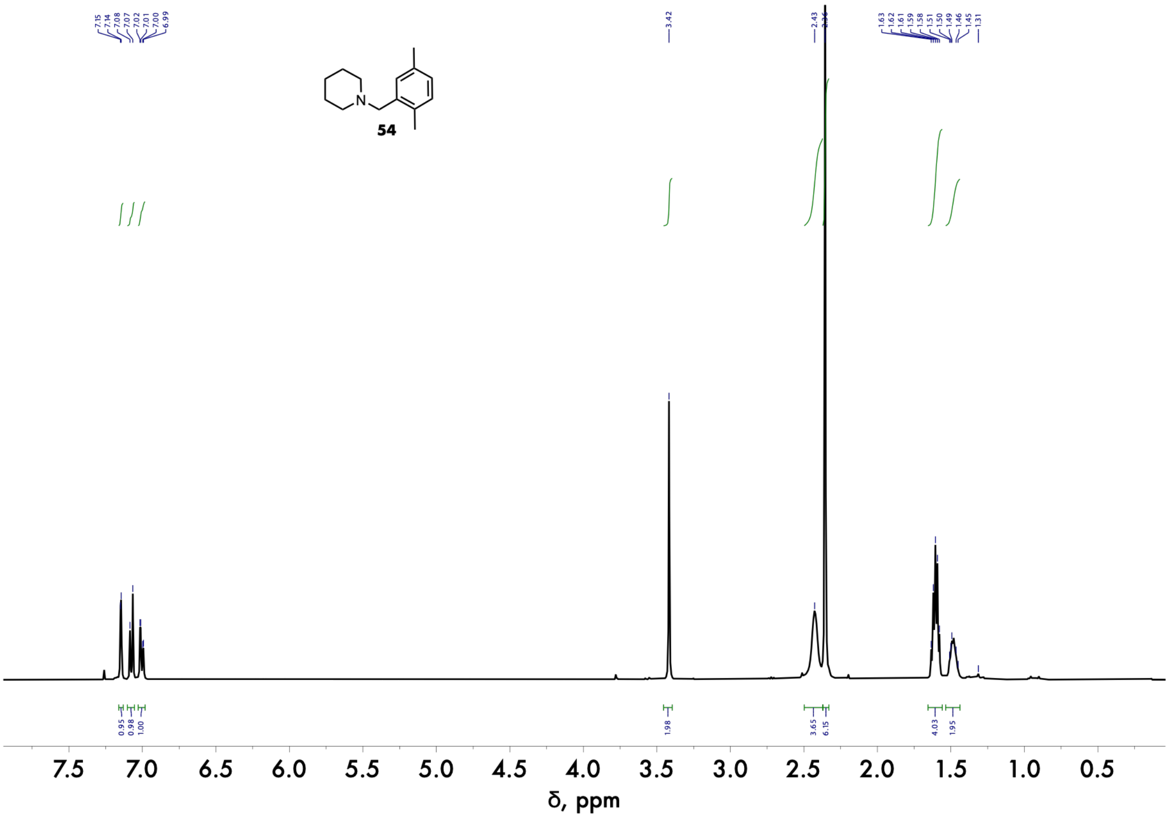

Figure S217. ^{1}H NMR (400 MHz, $CDCl_3$) spectrum of **54**.

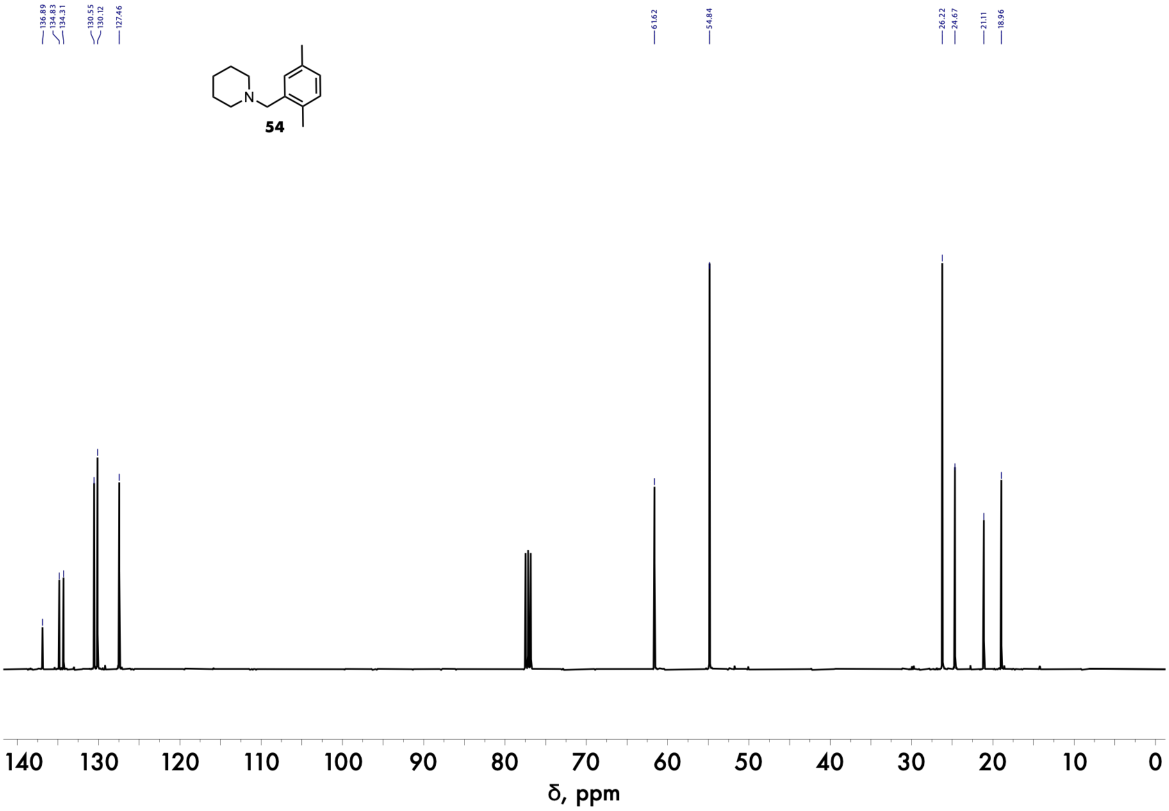

Figure S218. ^{13}C NMR (101 MHz, $CDCl_3$) spectrum of **54**.

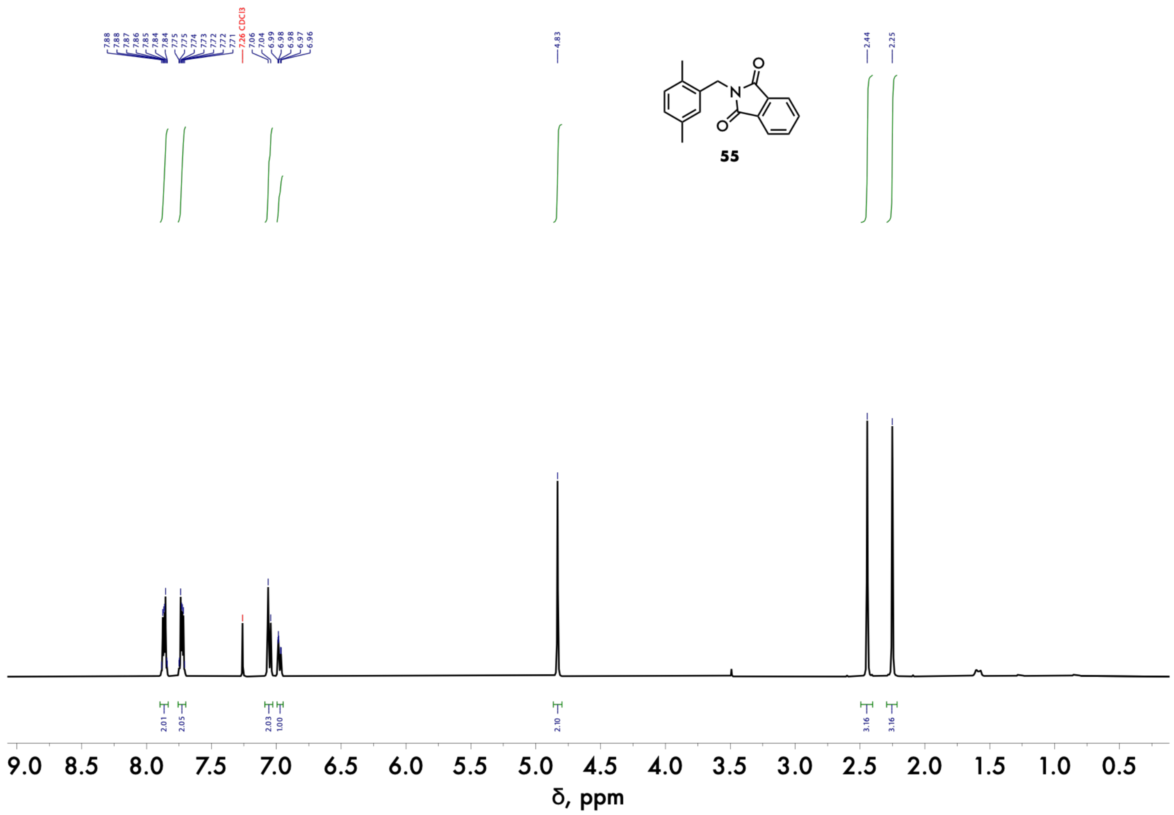

Figure S219. ^{1}H NMR (400 MHz, $CDCl_3$) spectrum of **55**.

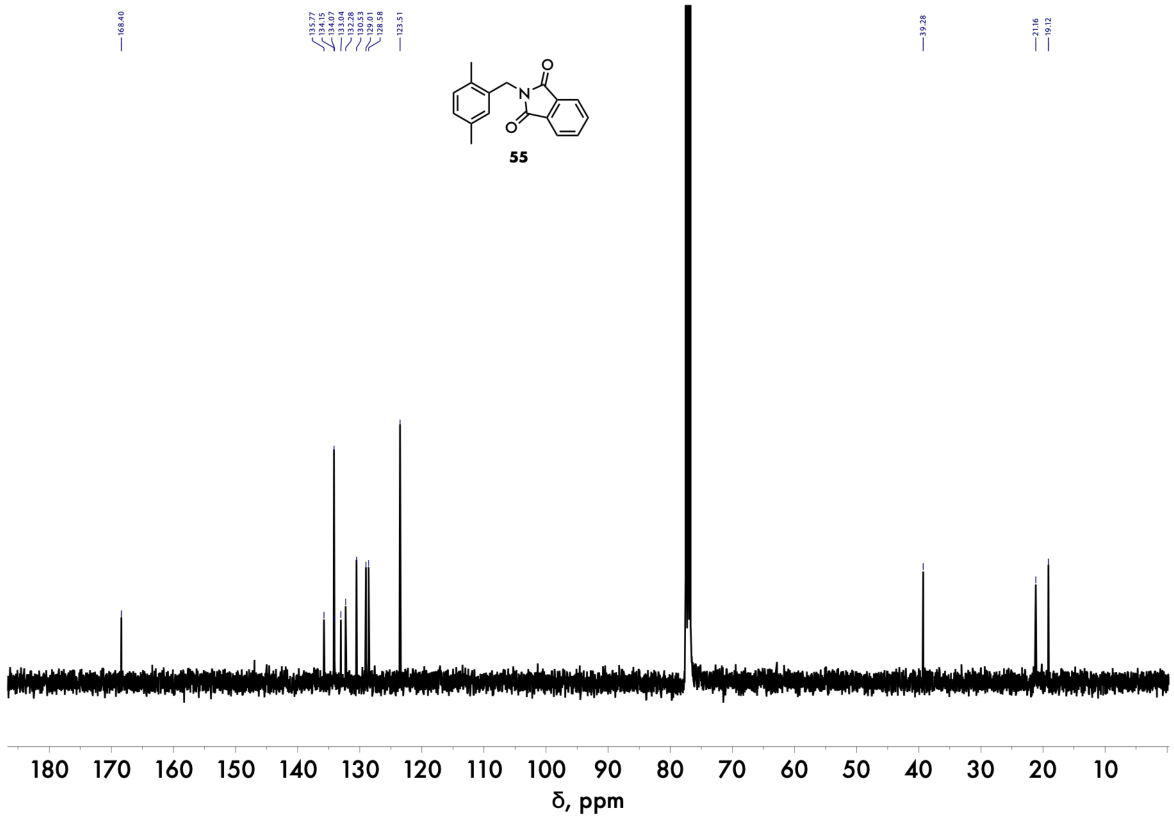

Figure S220. ^{13}C NMR (101 MHz, CD_3CN) spectrum of **55**.

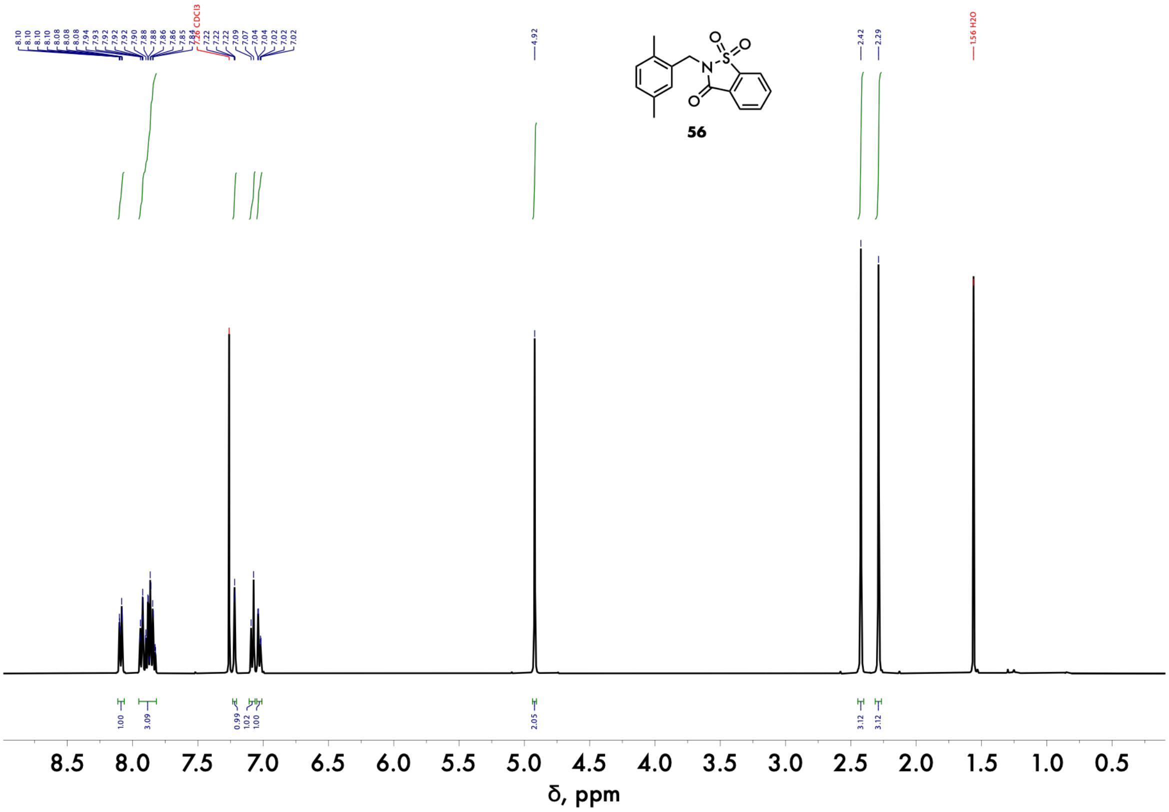

Figure S221. ^{1}H NMR (400 MHz, $CDCl_3$) spectrum of **56**.

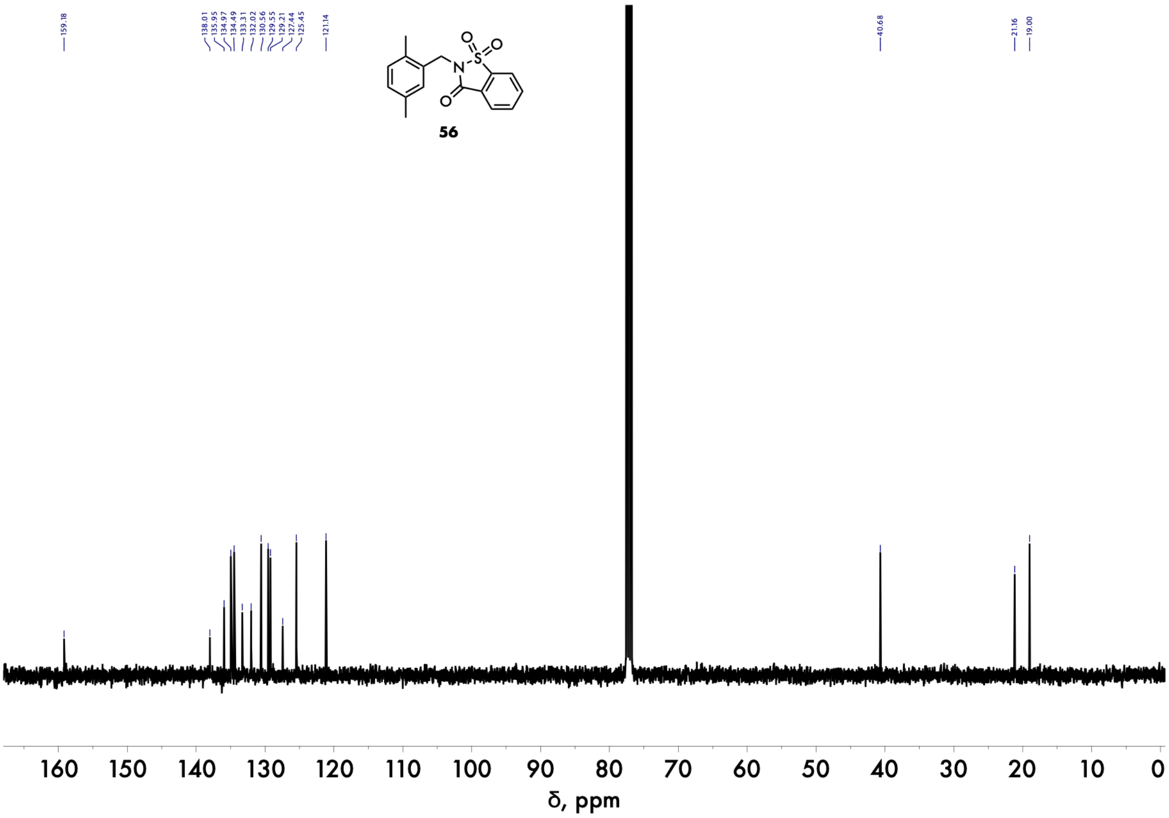

Figure S222. ^{13}C NMR (101 MHz, $CDCl_3$) spectrum of **56**.

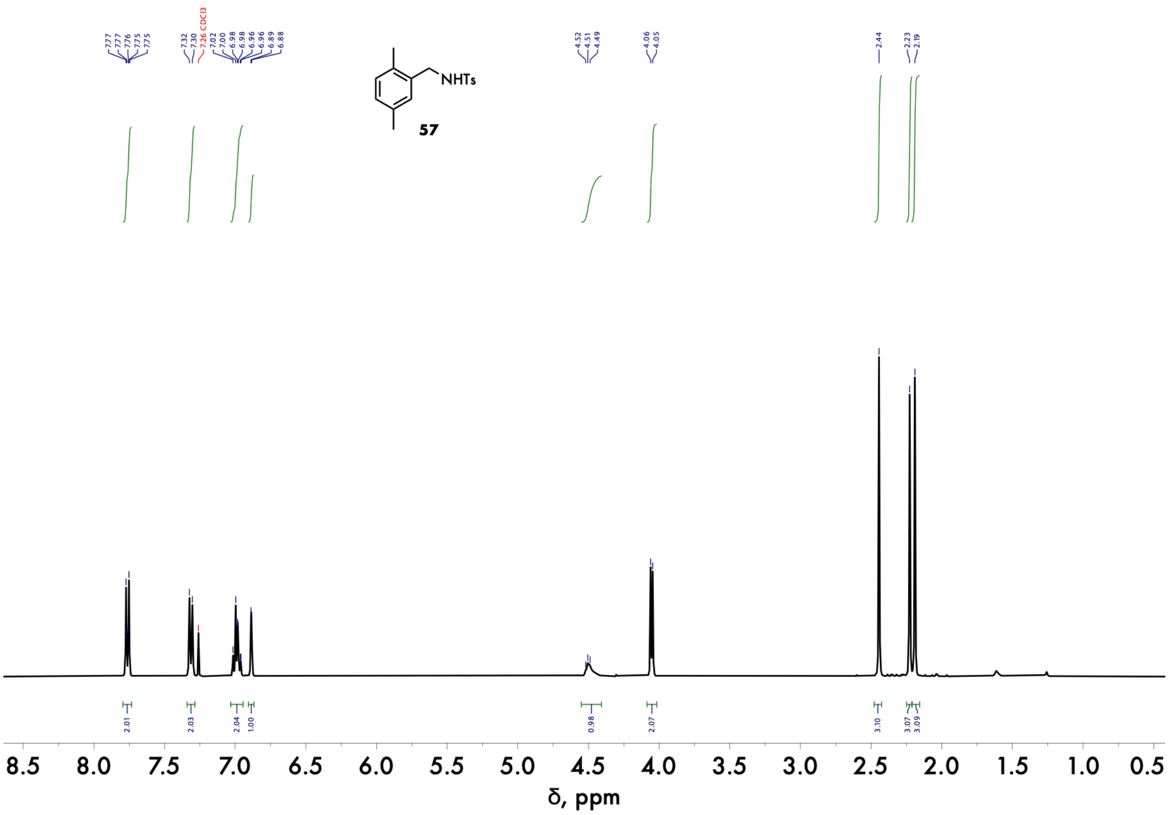

Figure S223. ^{1}H NMR (400 MHz, $CDCl_3$) spectrum of **57**.

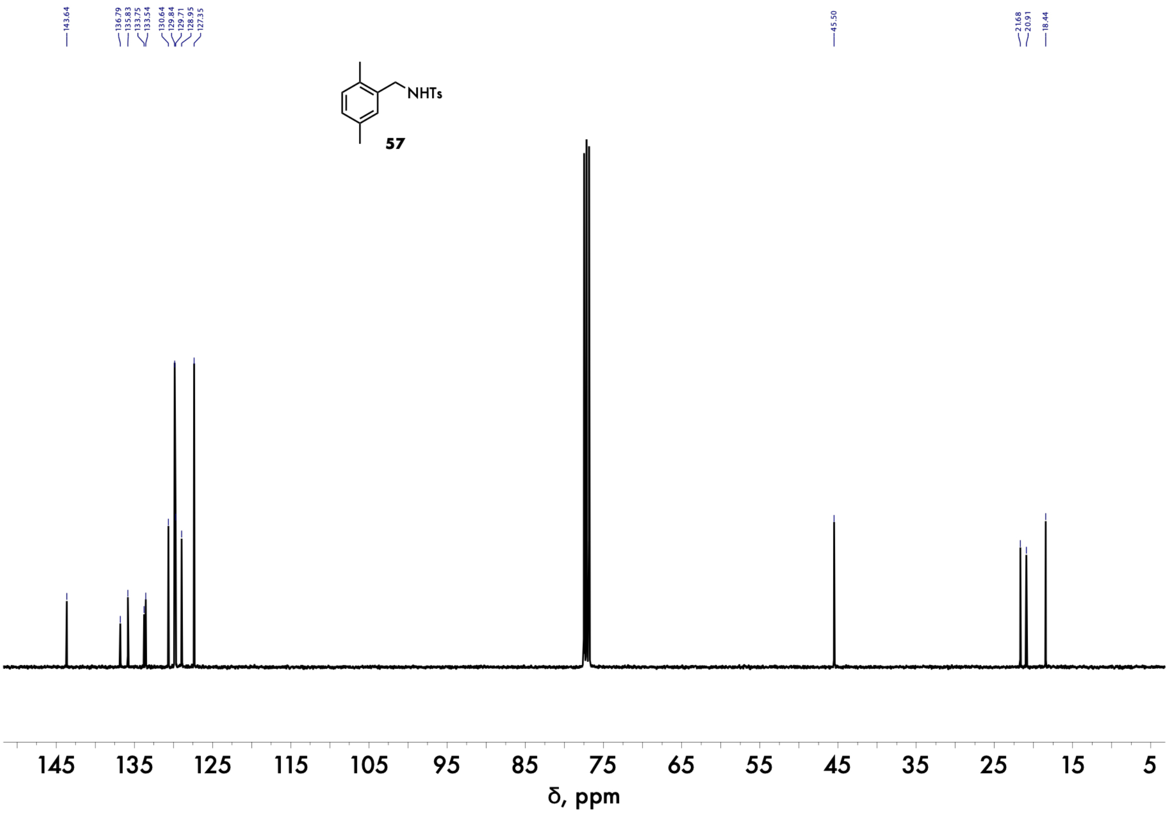

Figure S224. ^{13}C NMR (101 MHz, $CDCl_3$) spectrum of **57**.

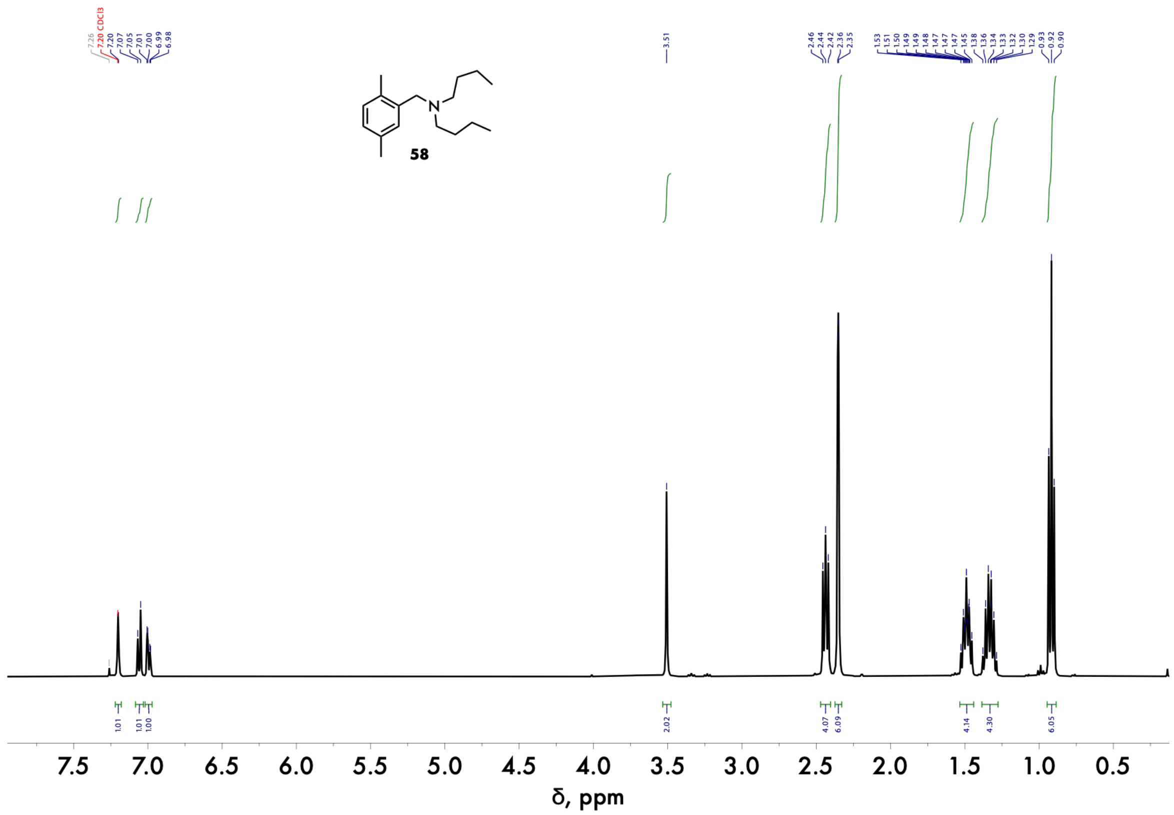

Figure S225. ^{1}H NMR (400 MHz, $CDCl_3$) spectrum of **58**.

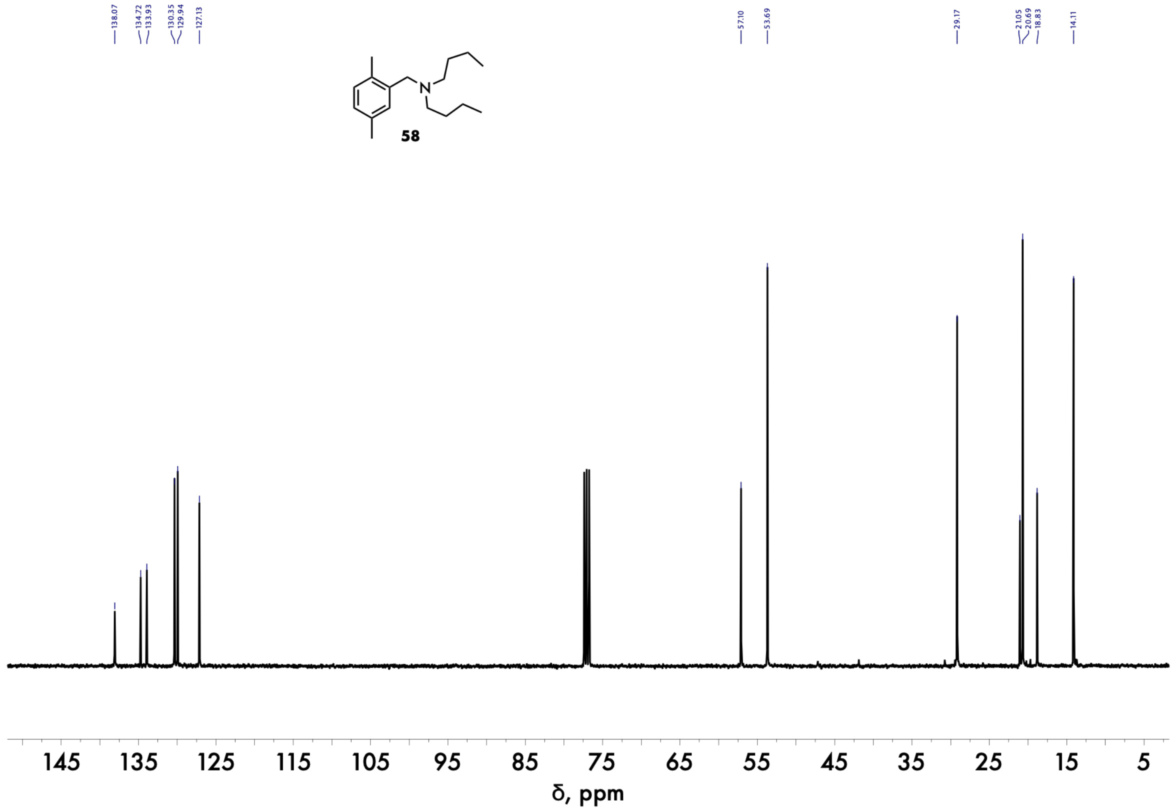

Figure S226. ^{13}C NMR (101 MHz, $CDCl_3$) spectrum of **58**.

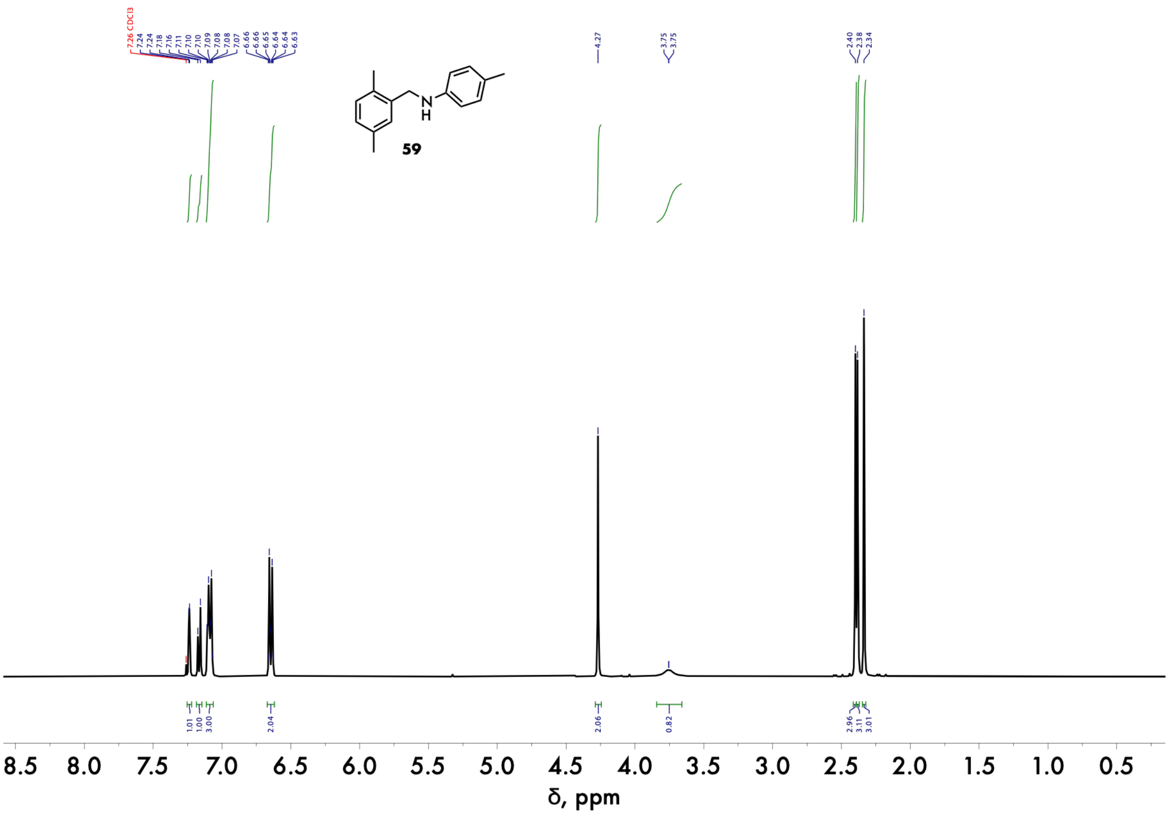

Figure S227. ^{1}H NMR (400 MHz, $CDCl_3$) spectrum of **59**.

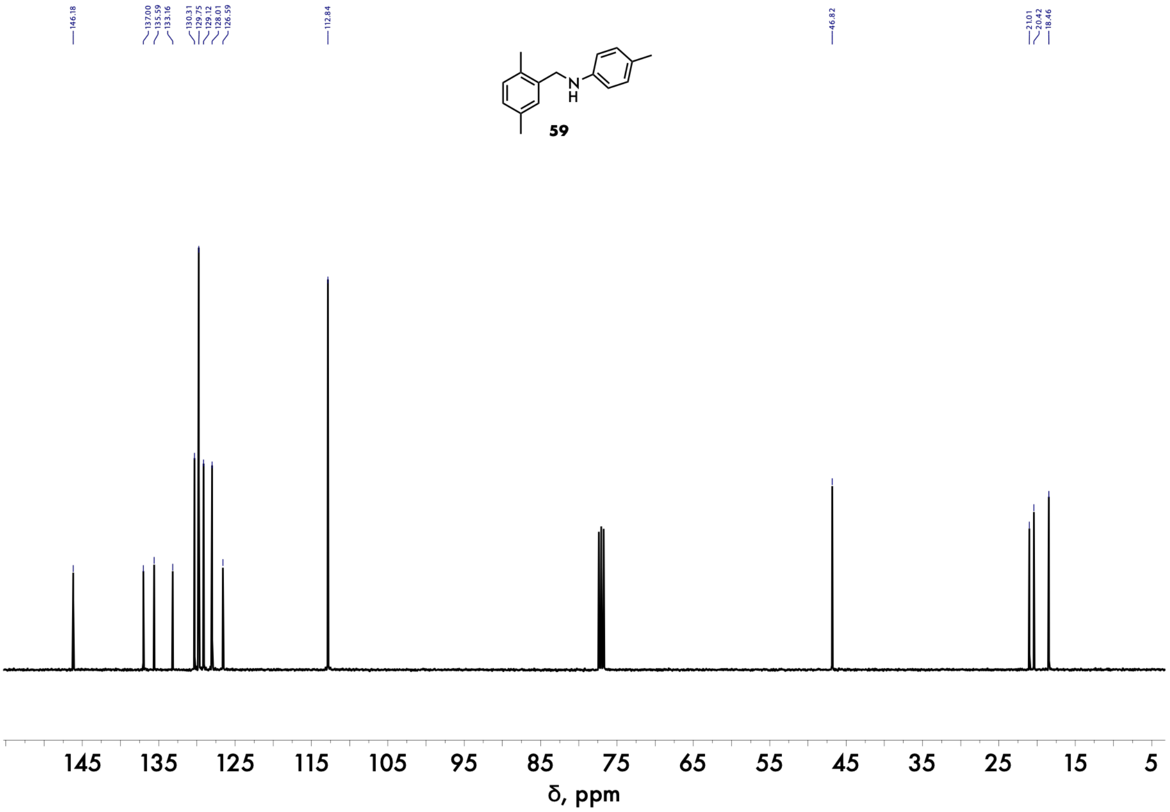

Figure S228. ^{13}C NMR (101 MHz, $CDCl_3$) spectrum of **59**.

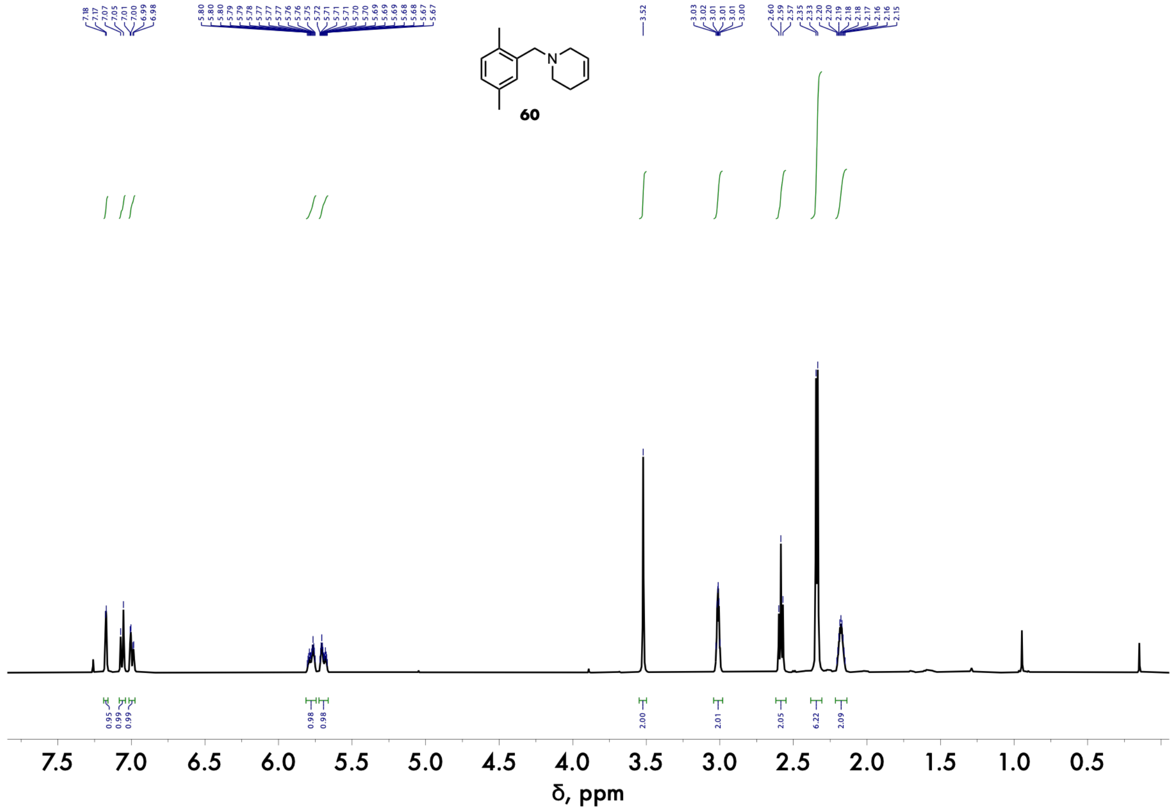

Figure S229. ^{1}H NMR (400 MHz, $CDCl_3$) spectrum of **60**.

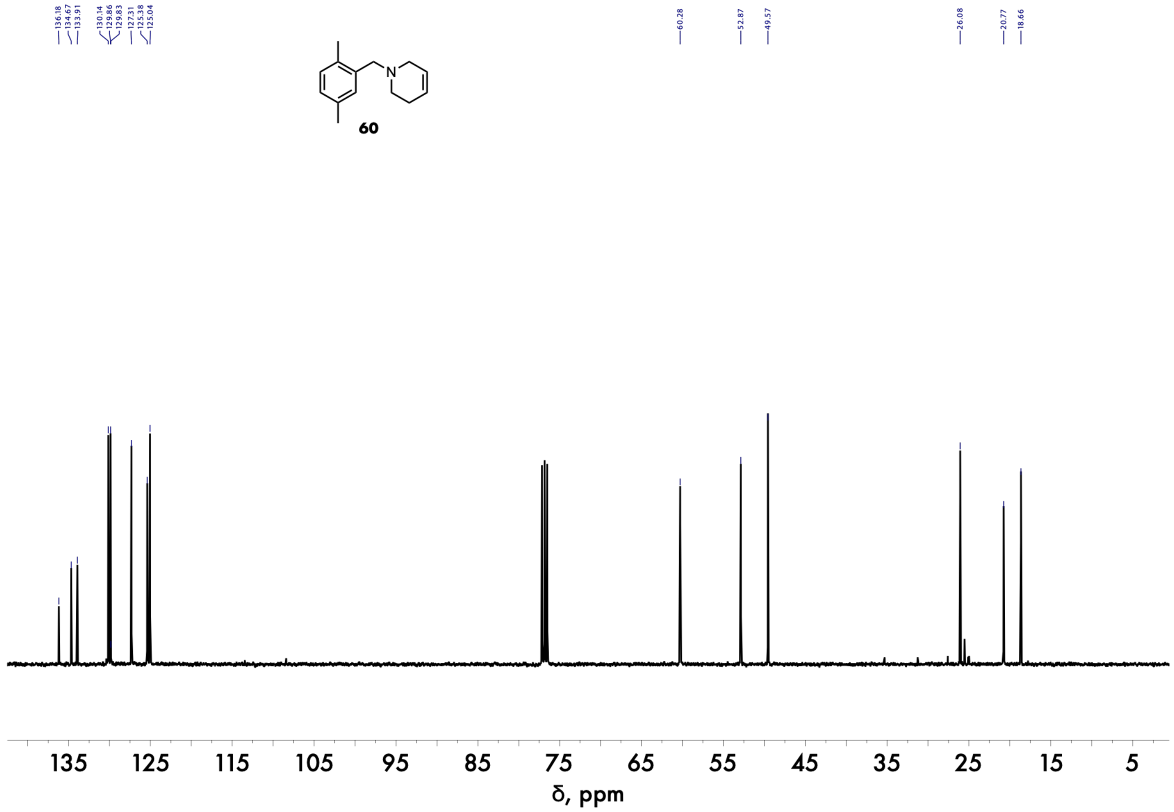

Figure S230. ^{13}C NMR (101 MHz, $CDCl_3$) spectrum of **60**.

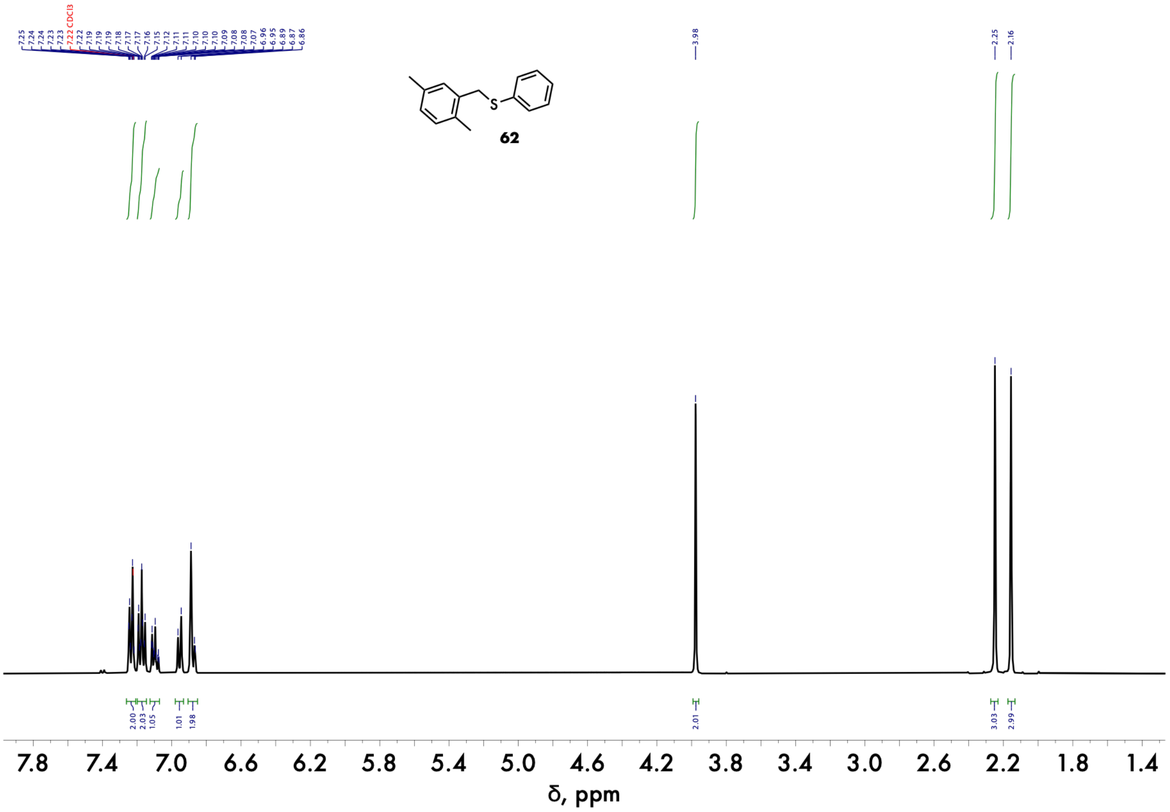

Figure S231. ^{1}H NMR (400 MHz, $CDCl_3$) spectrum of **62**.

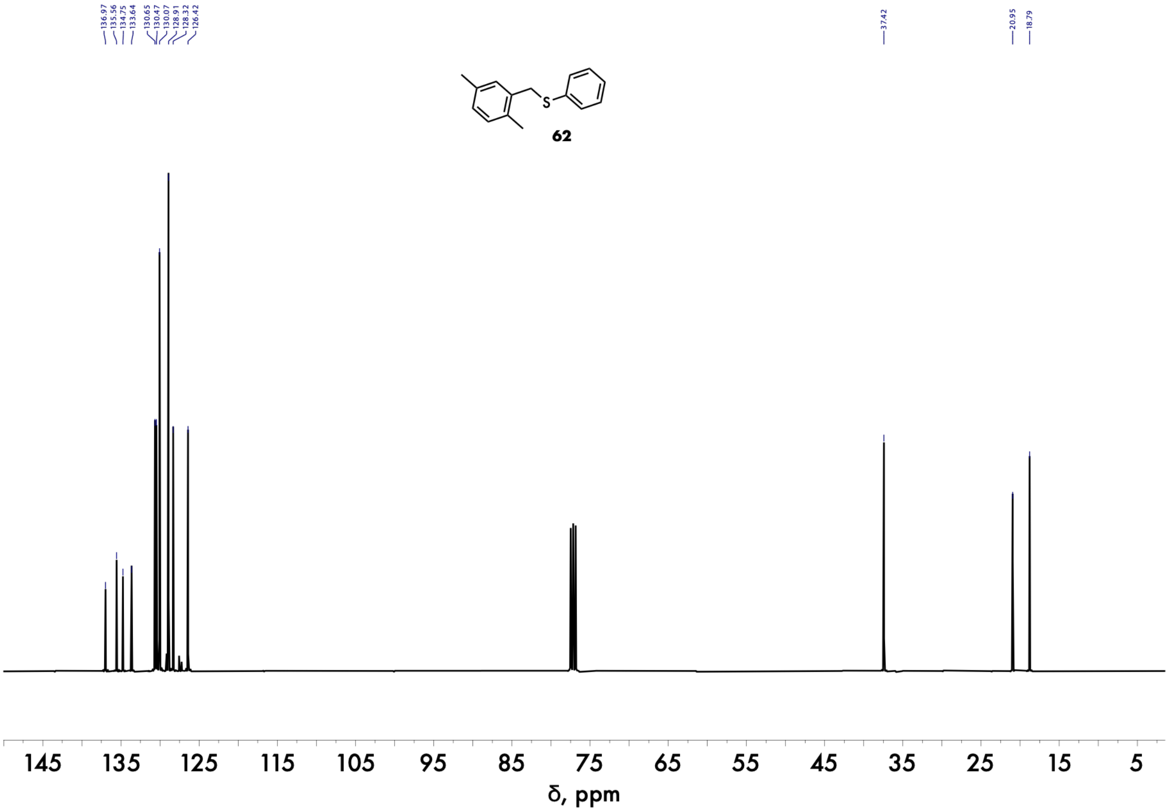

Figure S232. ^{13}C NMR (101 MHz, $CDCl_3$) spectrum of **62**.

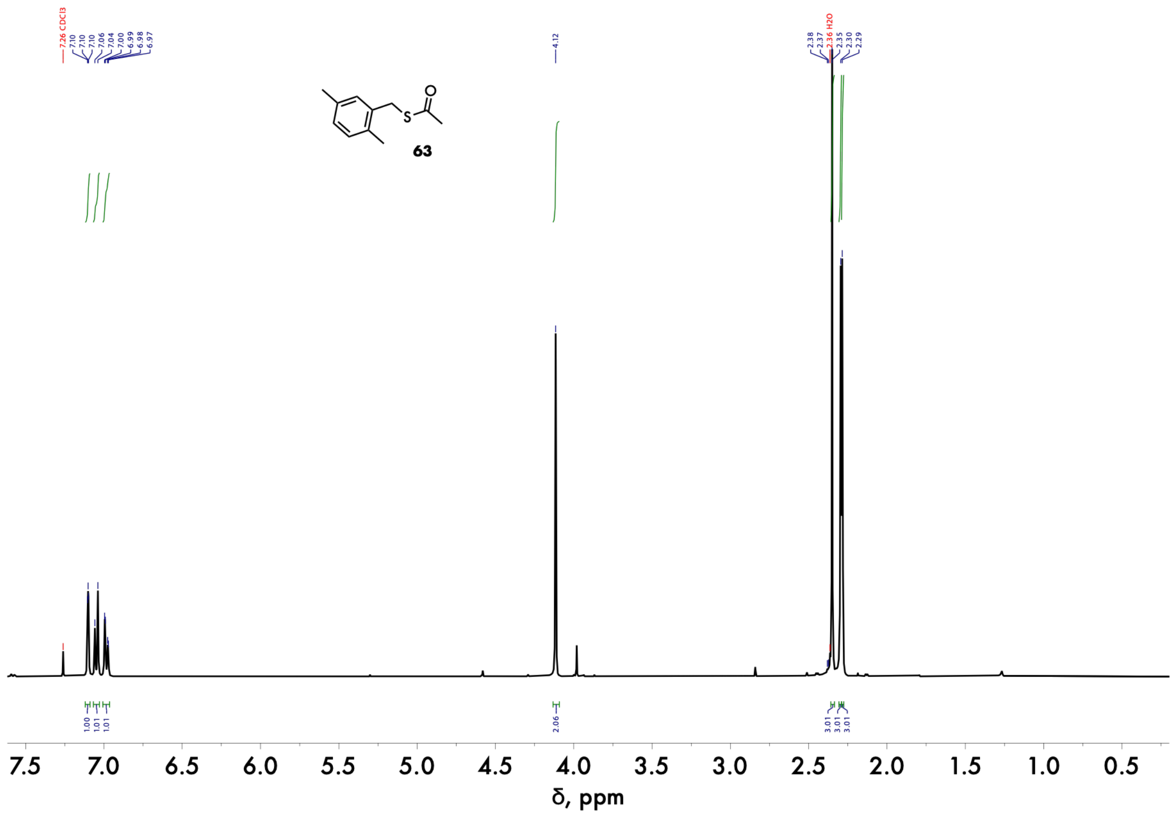

Figure S233. ^{1}H NMR (400 MHz, $CDCl_3$) spectrum of **63**.

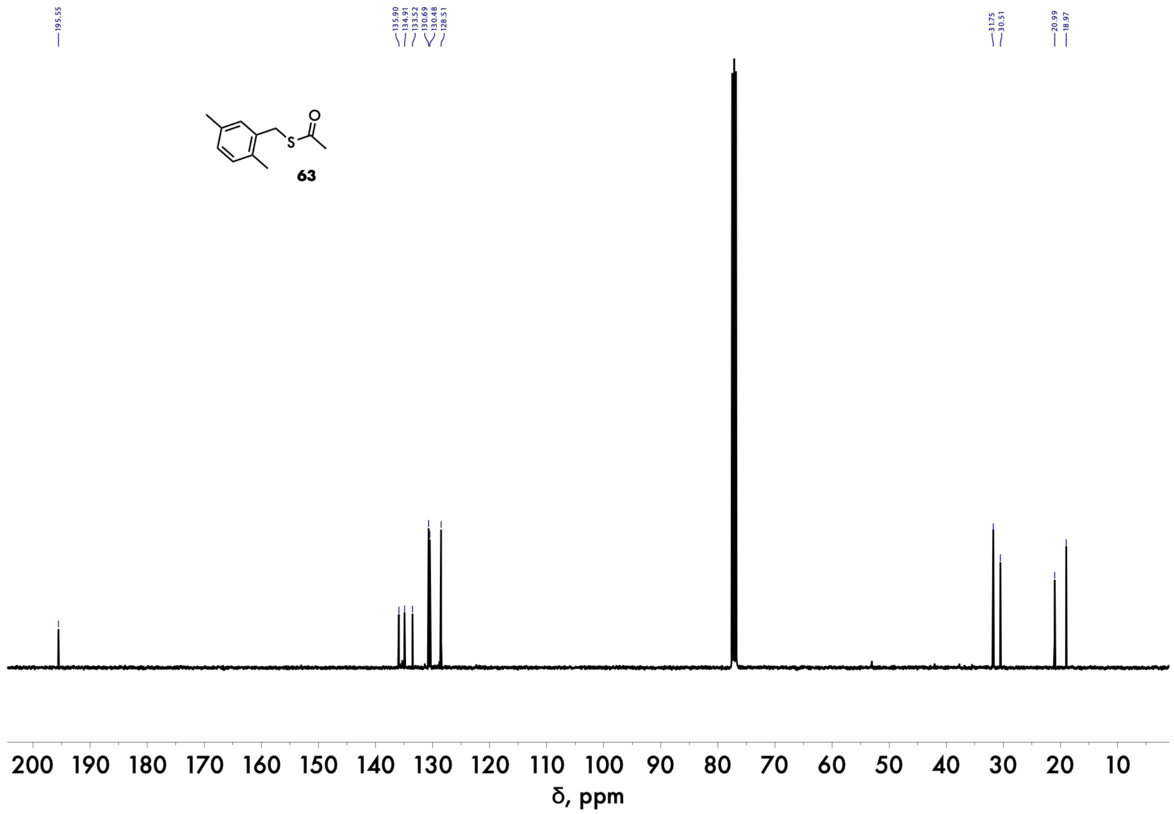

Figure S234. ^{13}C NMR (101 MHz, $CDCl_3$) spectrum of **63**.

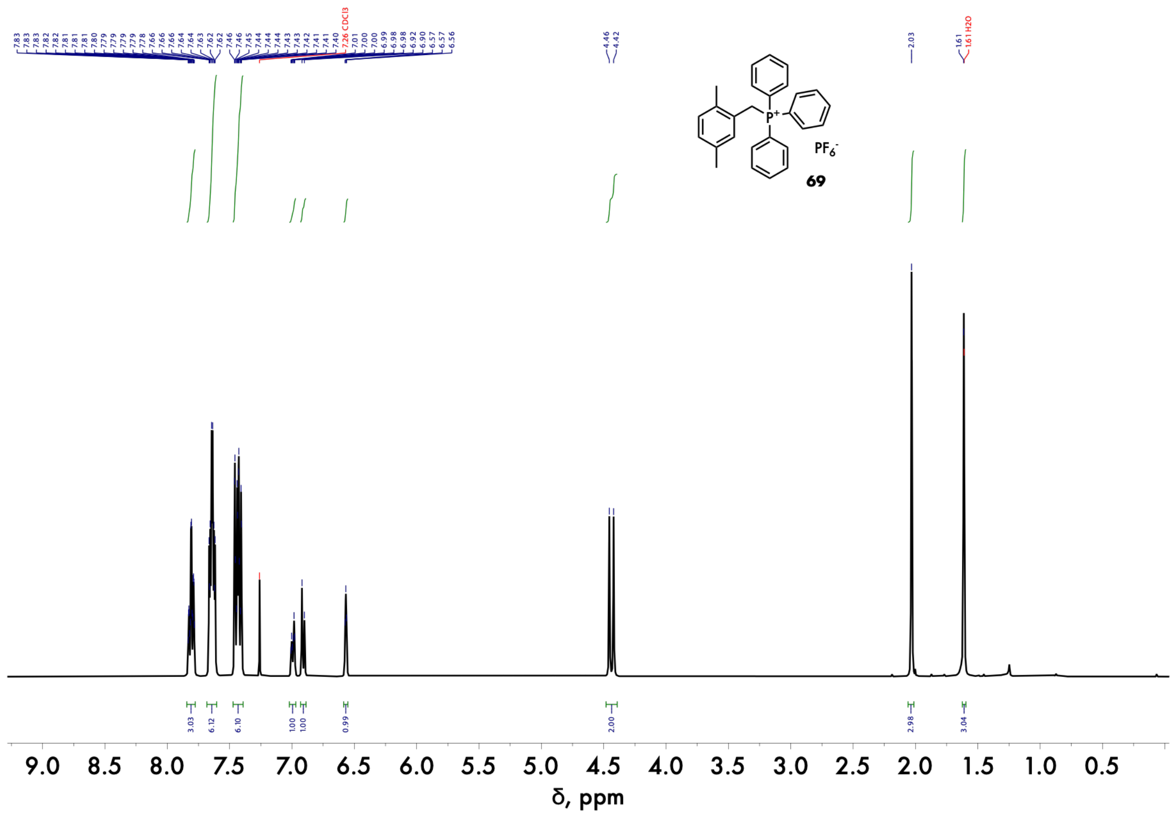

Figure S235. ^{1}H NMR (400 MHz, $CDCl_3$) spectrum of **69**.

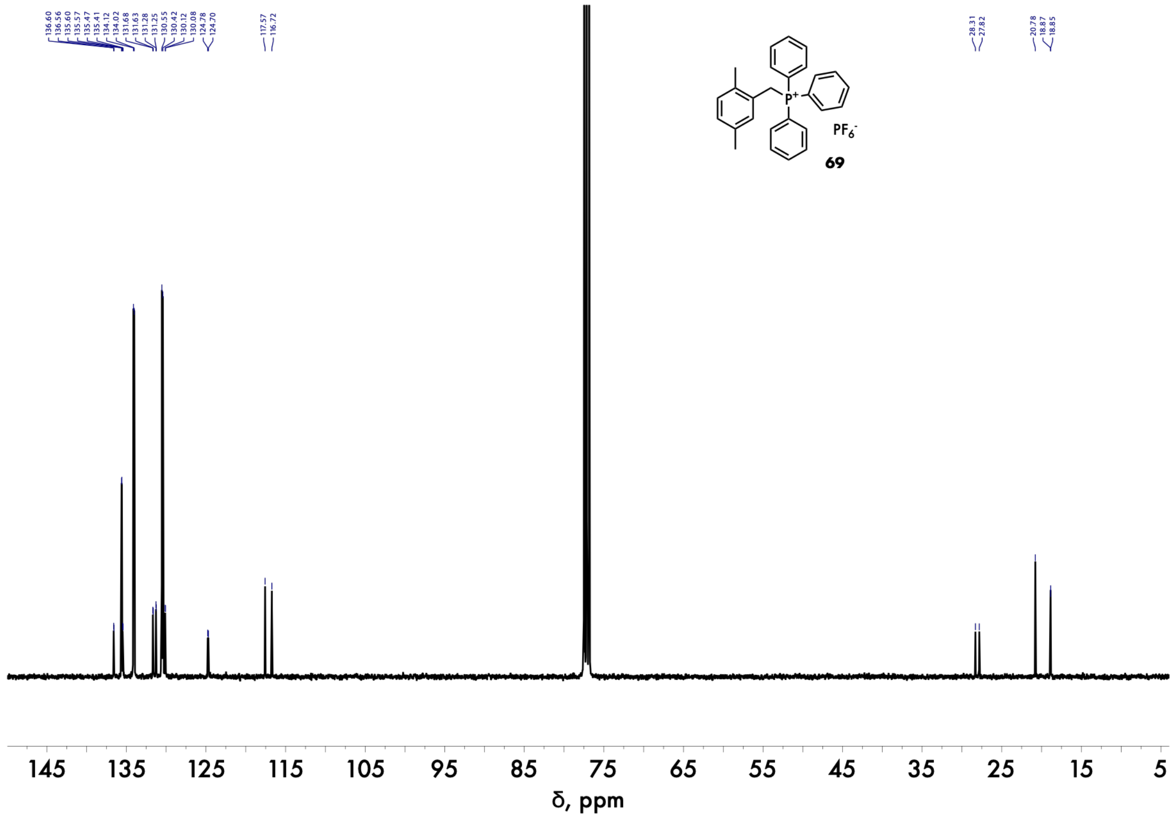

Figure S236. ^{13}C NMR (101 MHz, $CDCl_3$) spectrum of **69**.

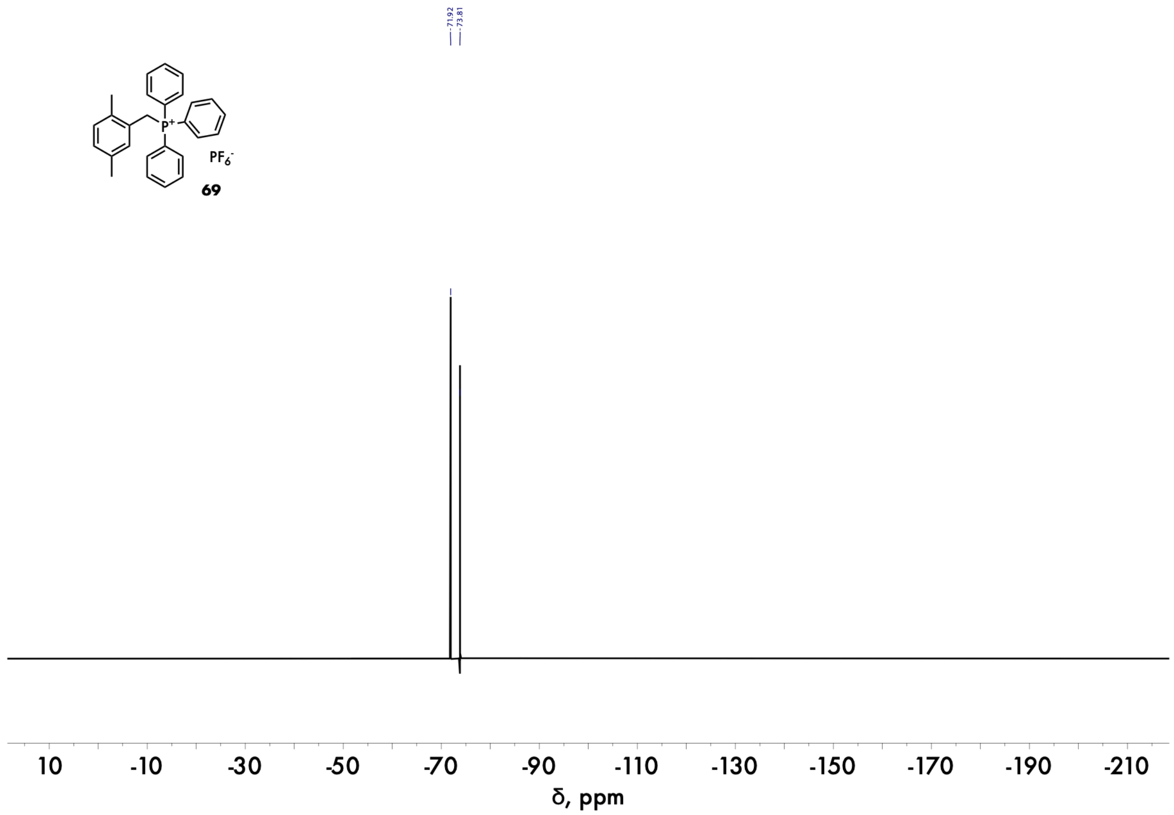

Figure S237. ^{19}F NMR (376 MHz, $CHCl_3$) spectrum of **69**.

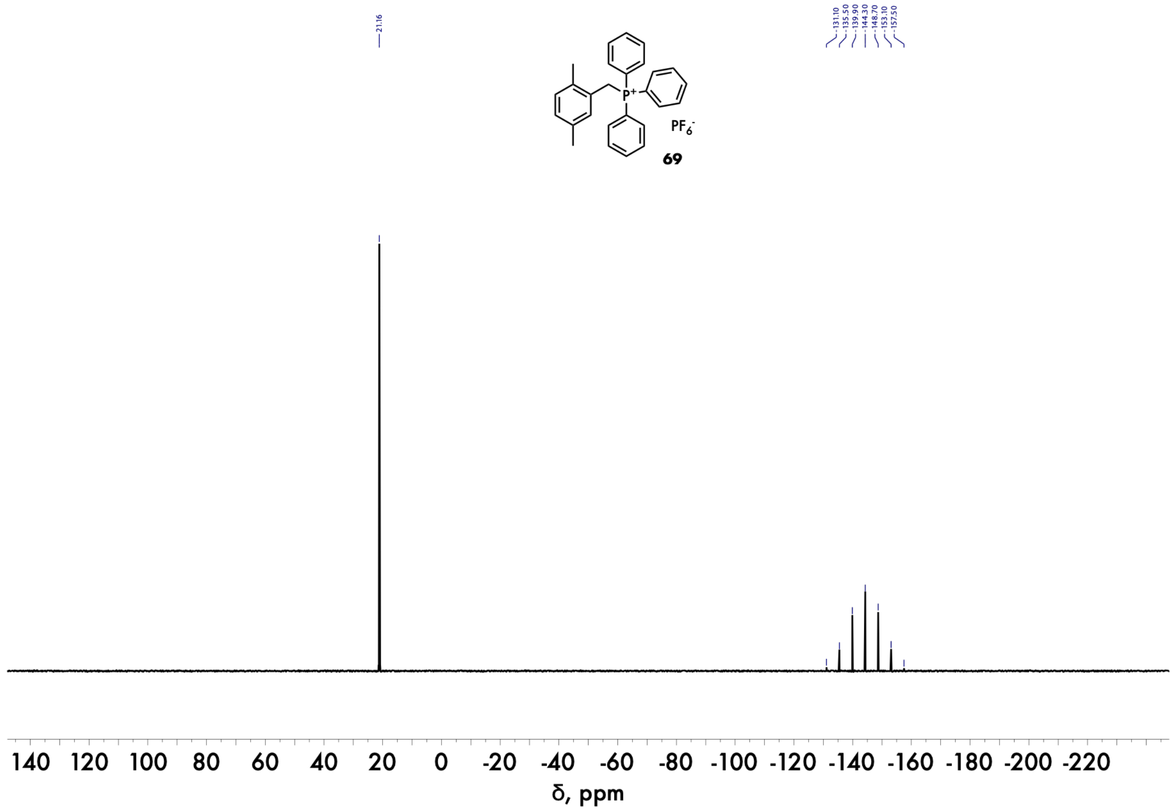

Figure S238. ^{31}P NMR (162 MHz, $CDCl_3$) spectrum of **69**.

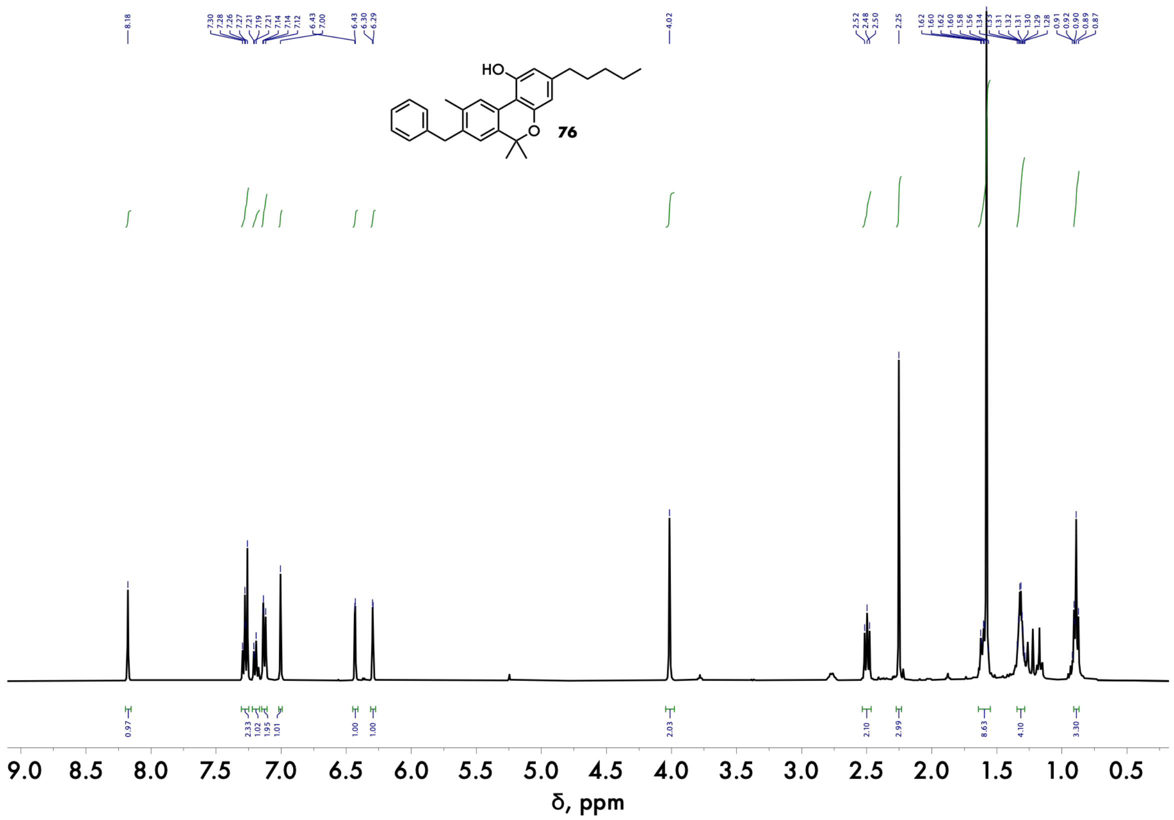

Figure S239. ^{1}H NMR (400 MHz, $CDCl_3$) spectrum of **76**.

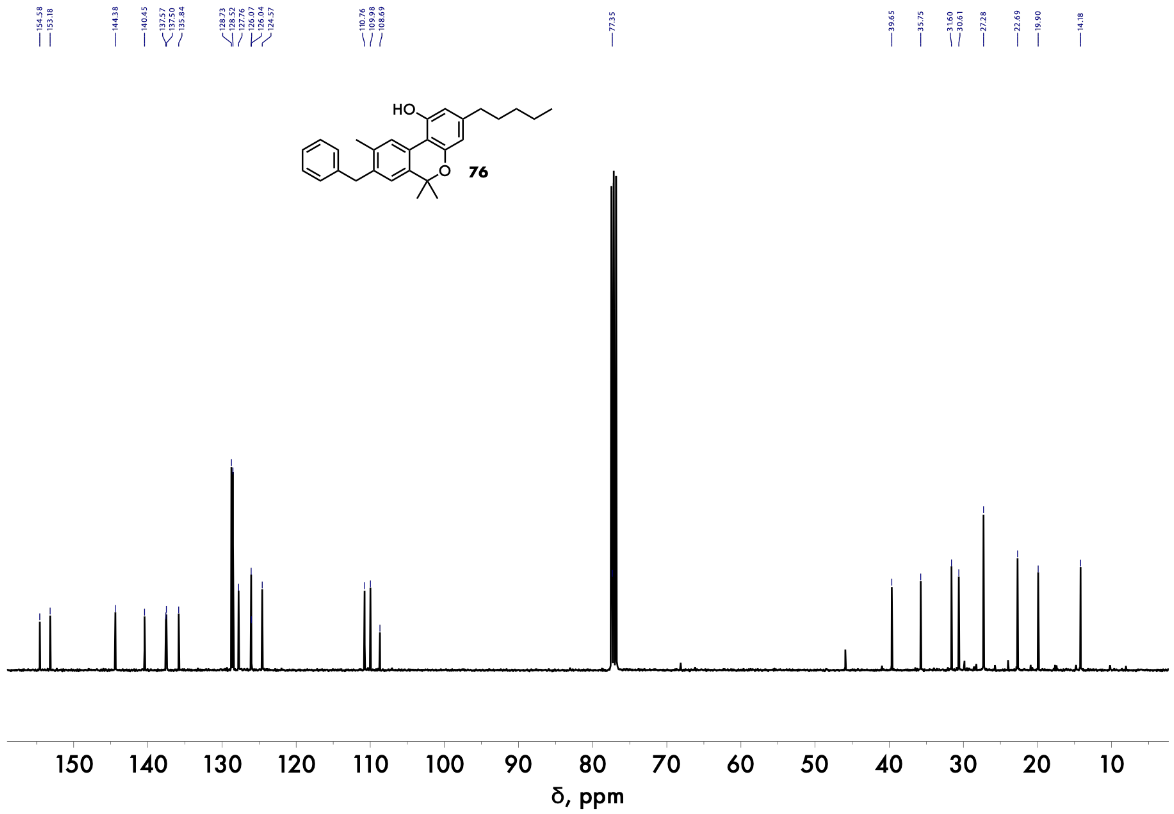

Figure S240. ^{13}C NMR (101 MHz, $CDCl_3$) spectrum of **76**.

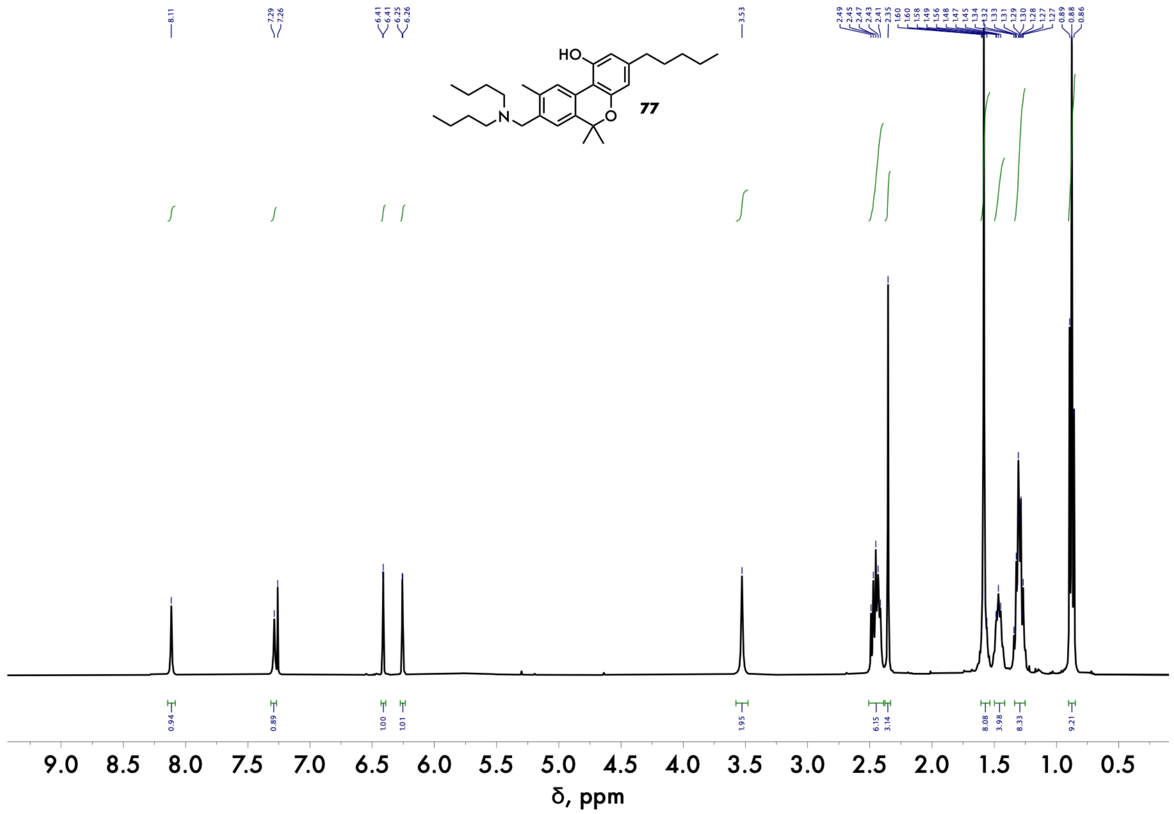

Figure S241. ^{1}H NMR (400 MHz, $CDCl_3$) spectrum of **77**.

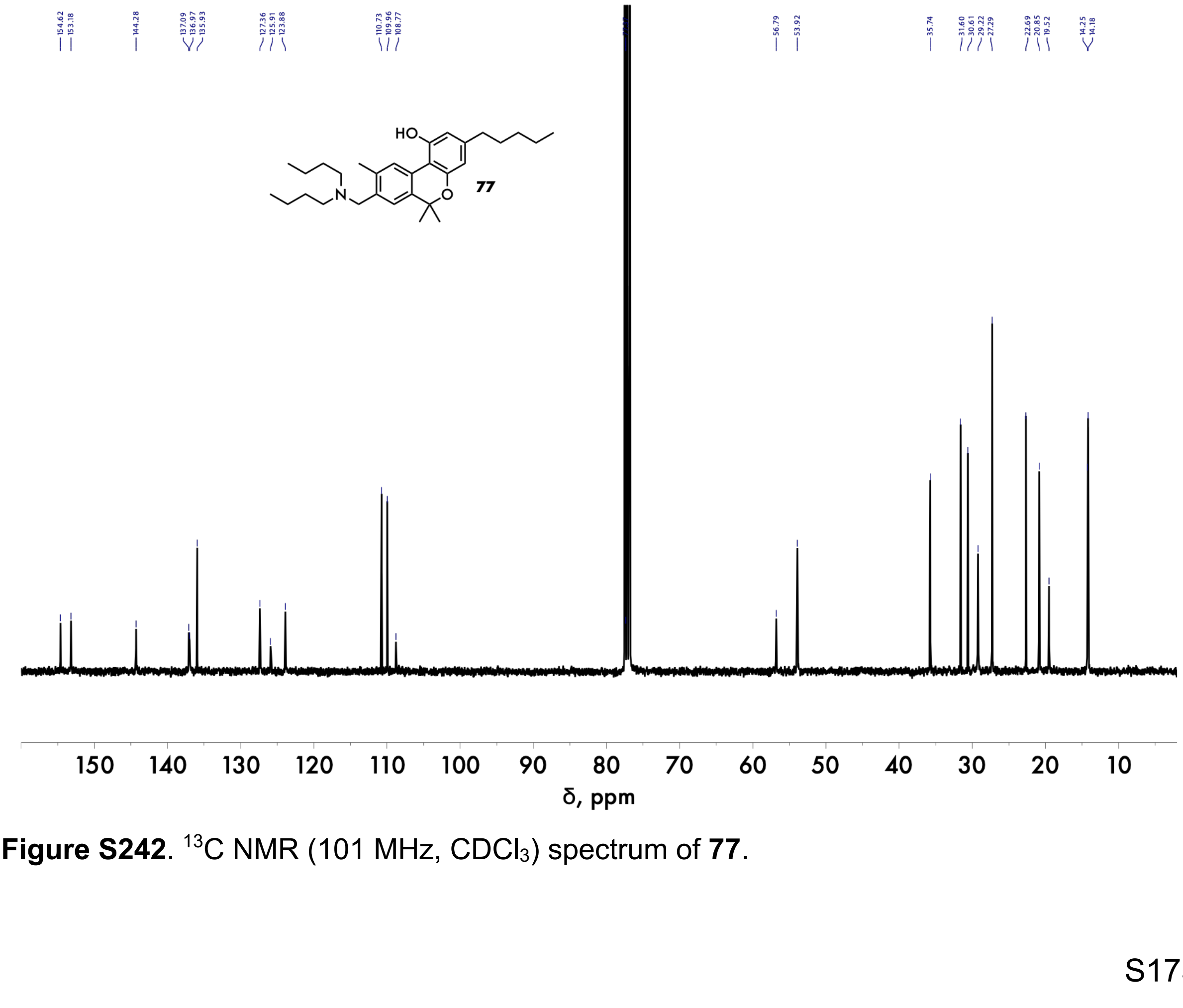

Figure S242. ^{13}C NMR (101 MHz, $CDCl_3$) spectrum of **77**.

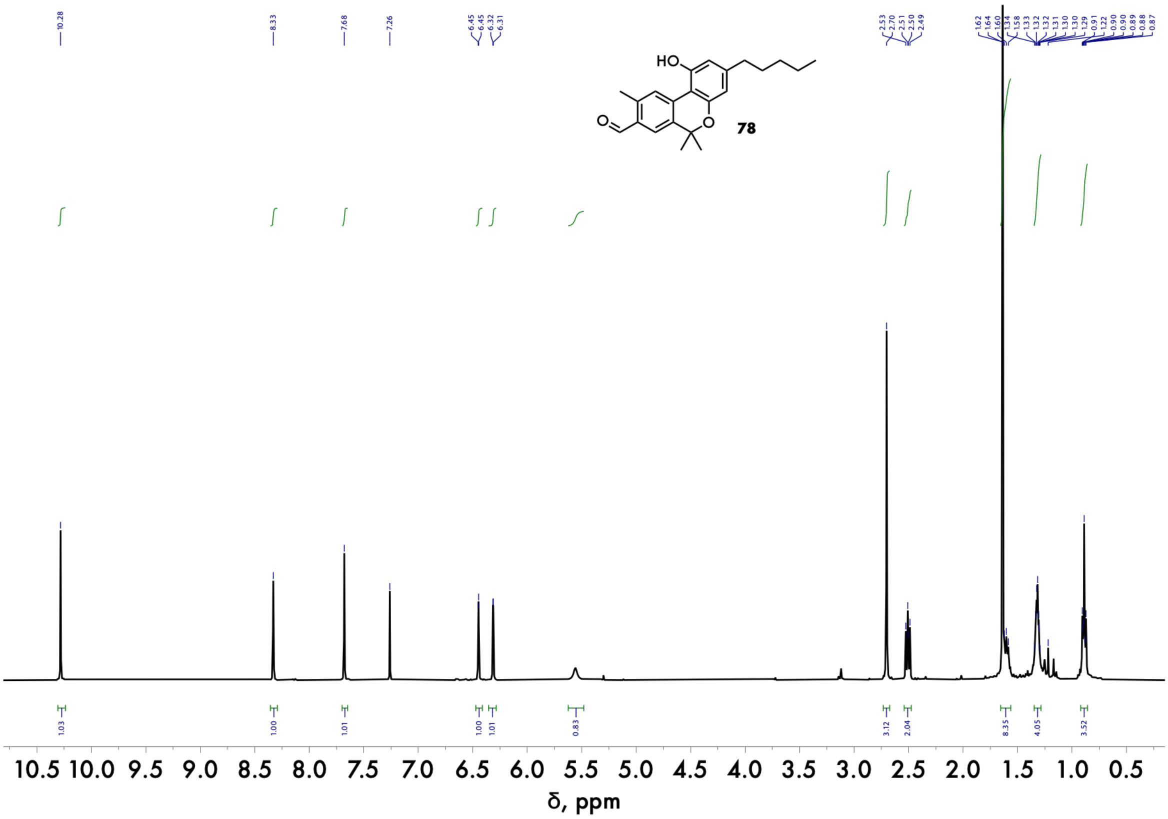

Figure S243. ^{1}H NMR (400 MHz, $CDCl_3$) spectrum of **78**.

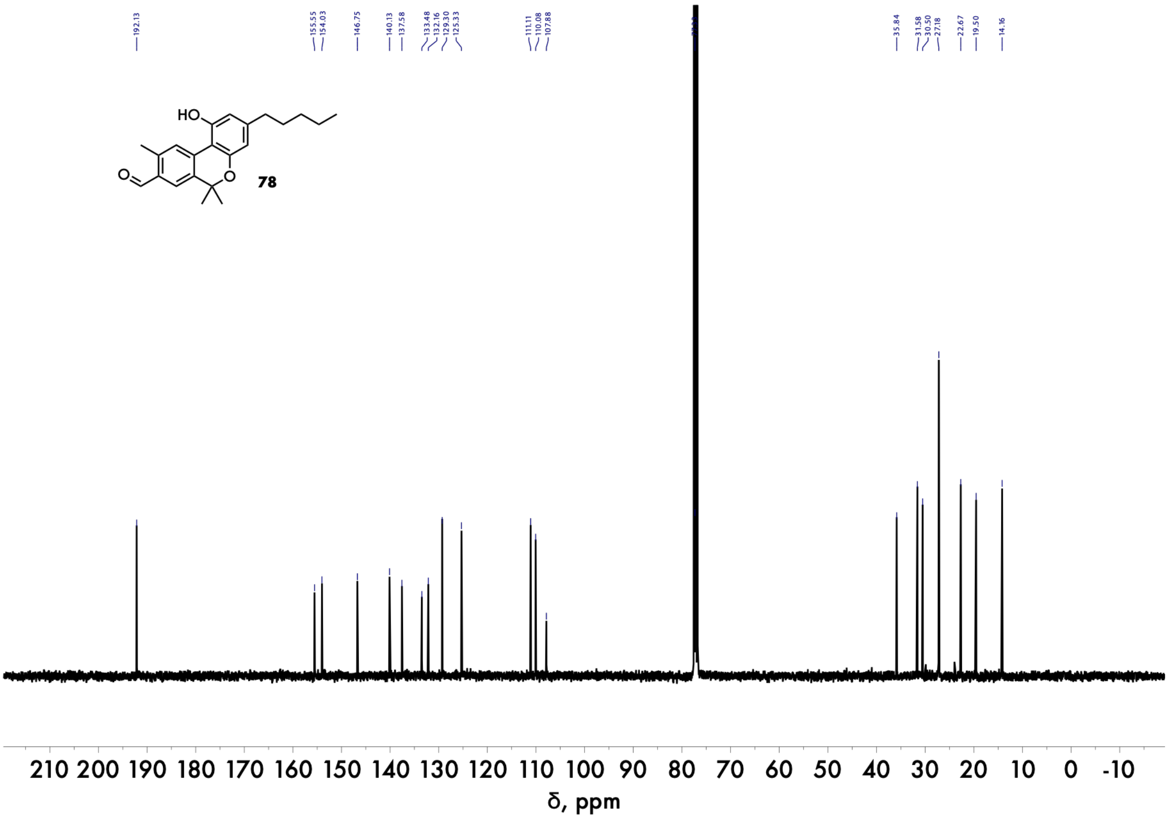

Figure S244. ^{13}C NMR (101 MHz, $CDCl_3$) spectrum of **78**.

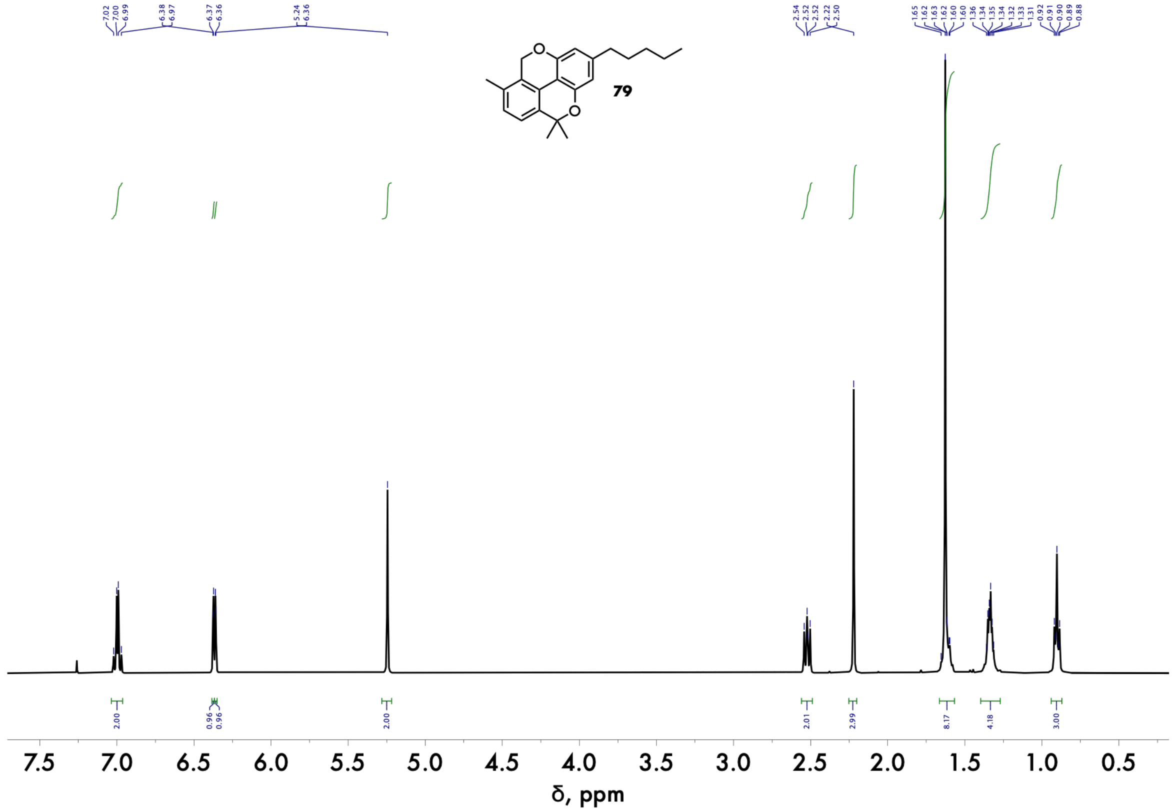

Figure S245. ^{1}H NMR (400 MHz, $CDCl_3$) spectrum of **79**.

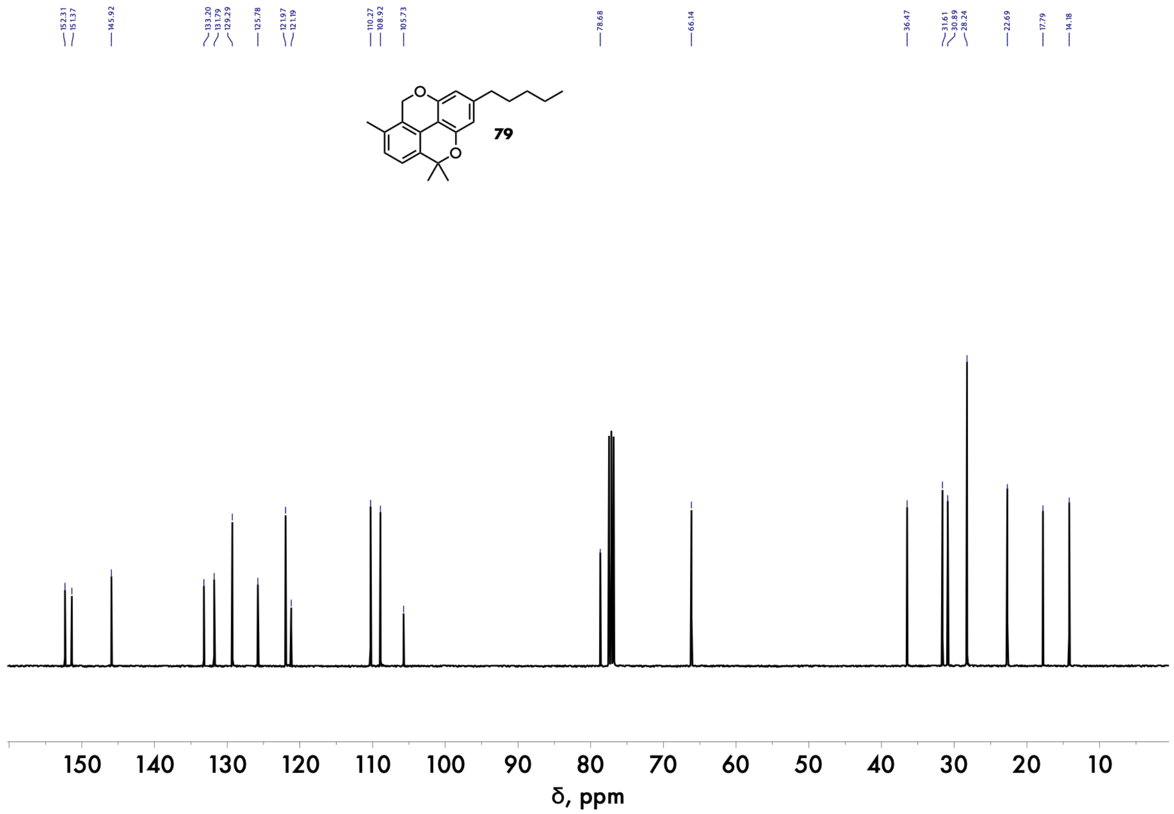

Figure S246. ^{13}C NMR (101 MHz, $CDCl_3$) spectrum of **79**.

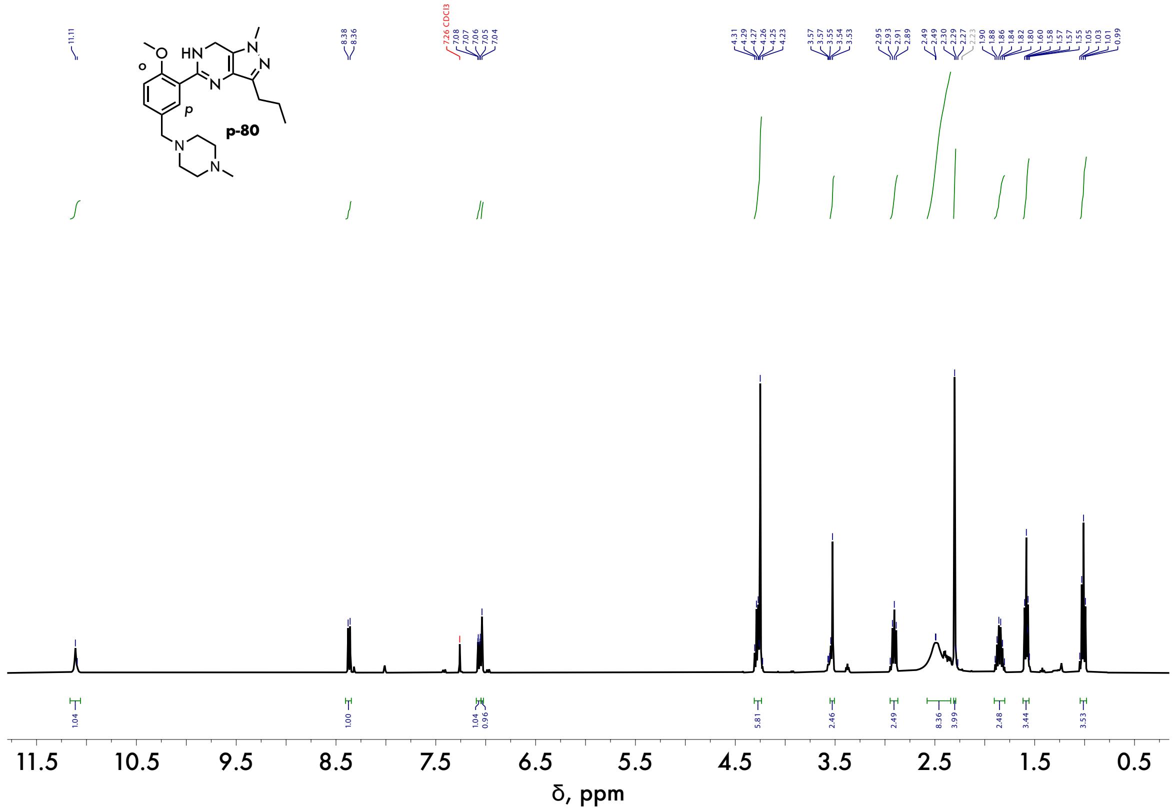

Figure S247. ^{1}H NMR (400 MHz, $CDCl_3$) spectrum of ***p*-80**.

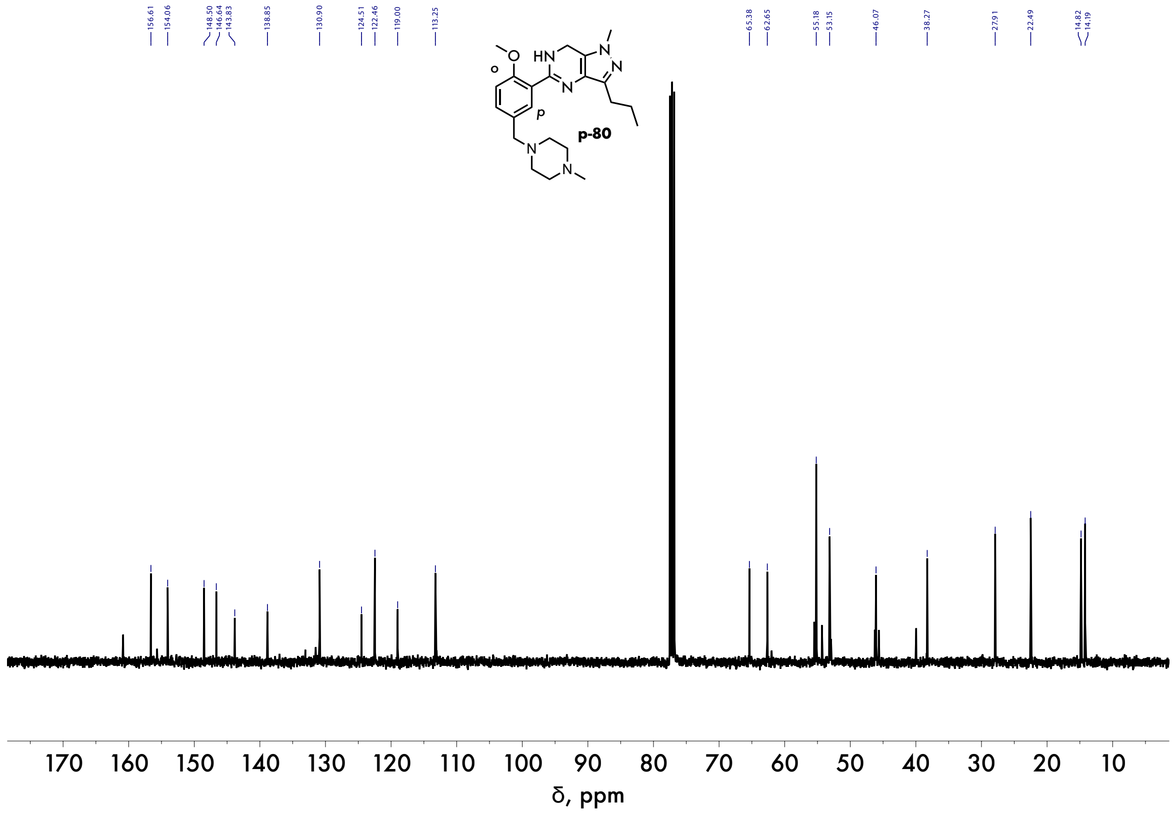

Figure S248. ^{13}C NMR (101 MHz, CD_3CN) spectrum of ***p*-80**.

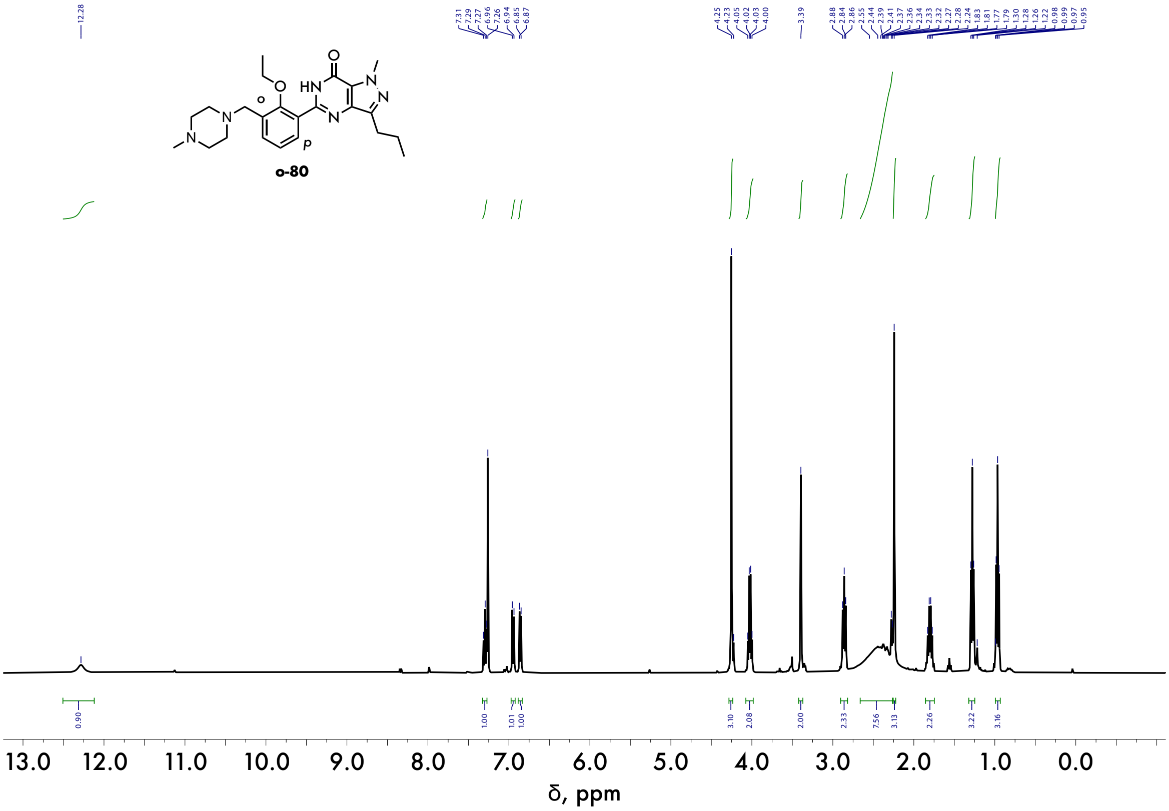

Figure S249. ^{1}H NMR (400 MHz, $CDCl_3$) spectrum of ***o*-80**.

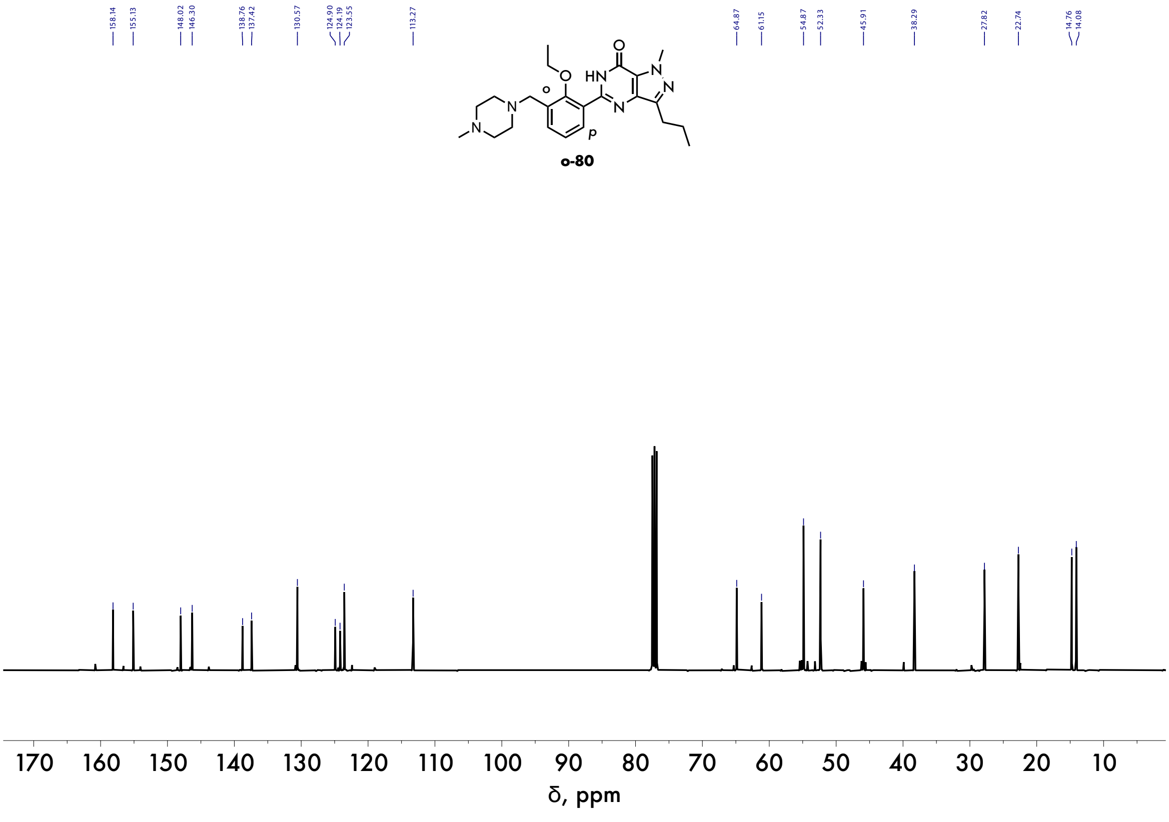

Figure S250. ^{13}C NMR (101 MHz, CD_3CN) spectrum of ***o*-80**.

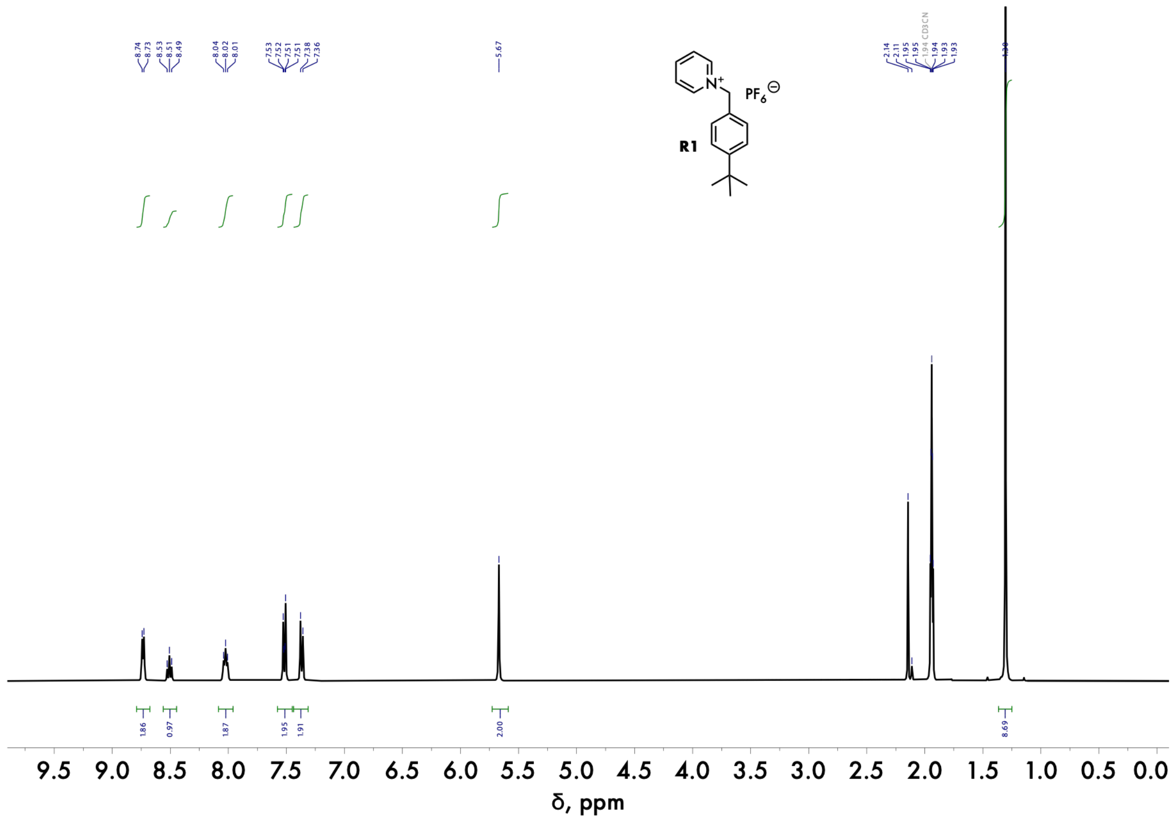

Figure S260. ^{1}H NMR (400 MHz, $CDCl_3$) spectrum of **R1**.

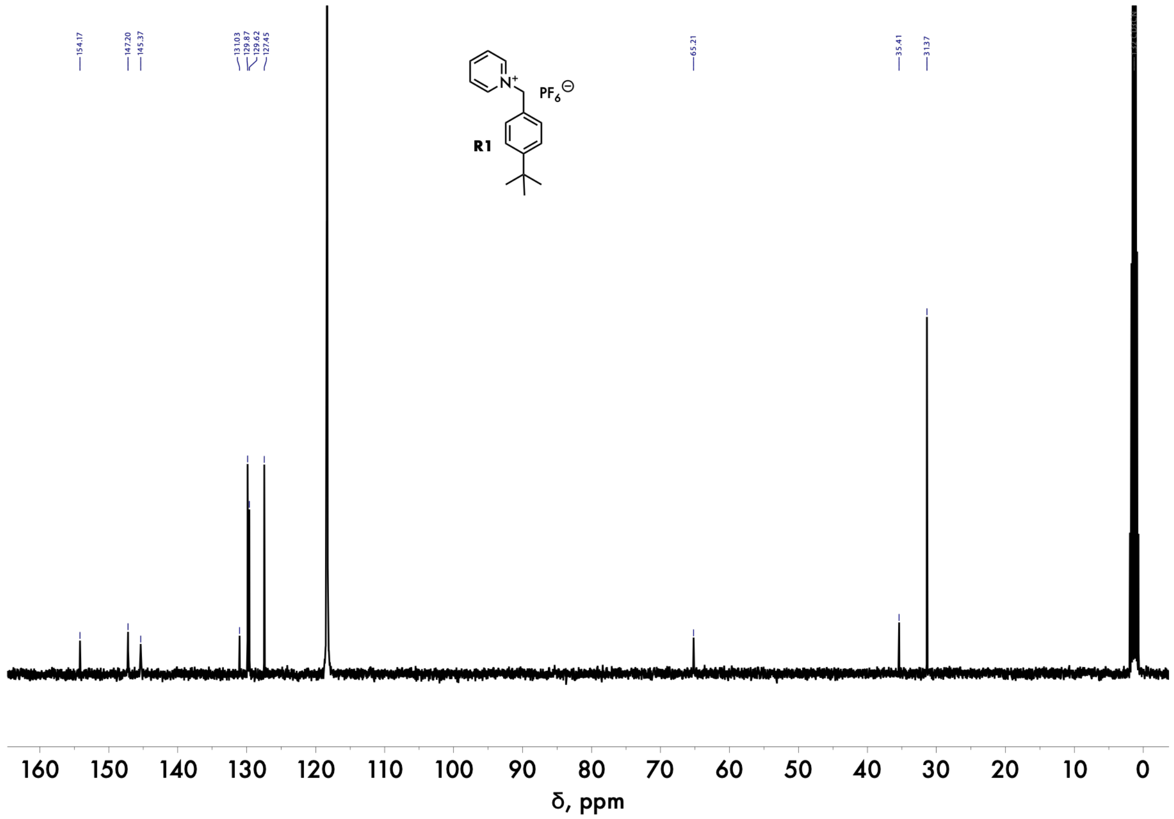

Figure S261. ^{13}C NMR (101 MHz, $CDCl_3$) spectrum of **R1**.

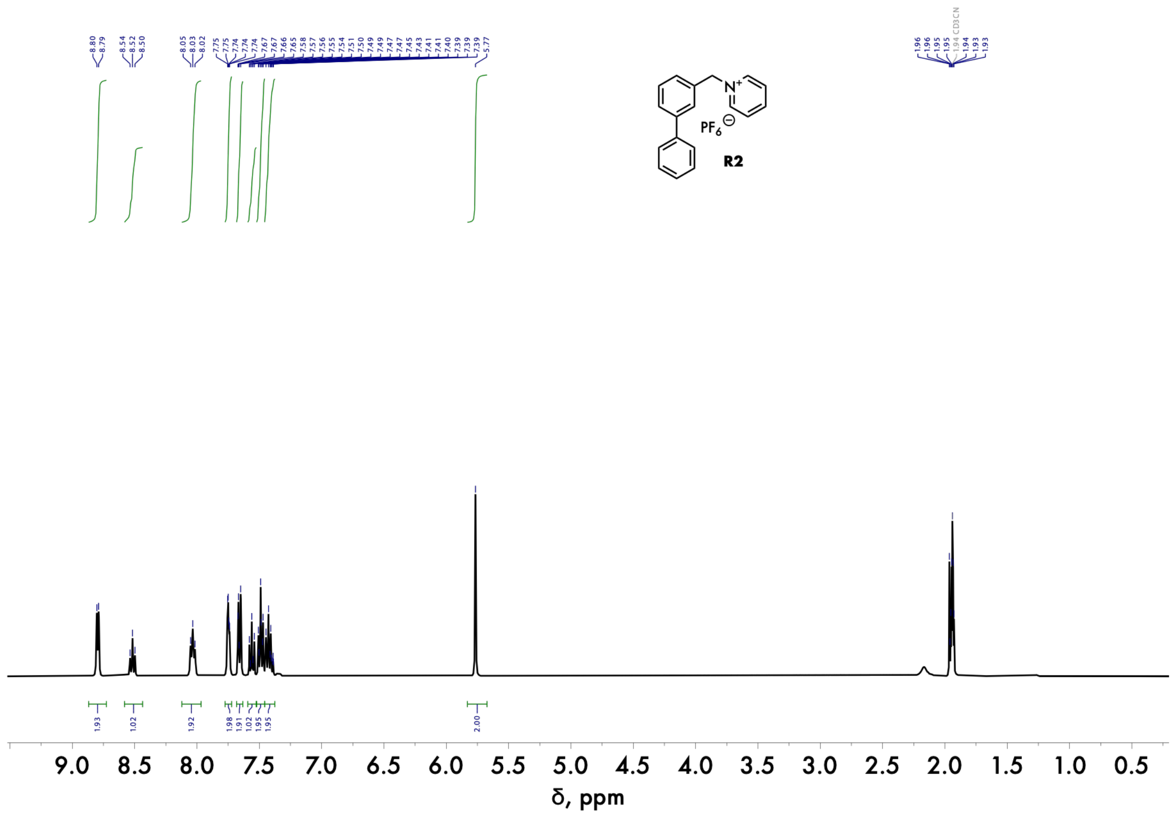

Figure S262. ^{1}H NMR (400 MHz, $CDCl_3$) spectrum of **R2**.

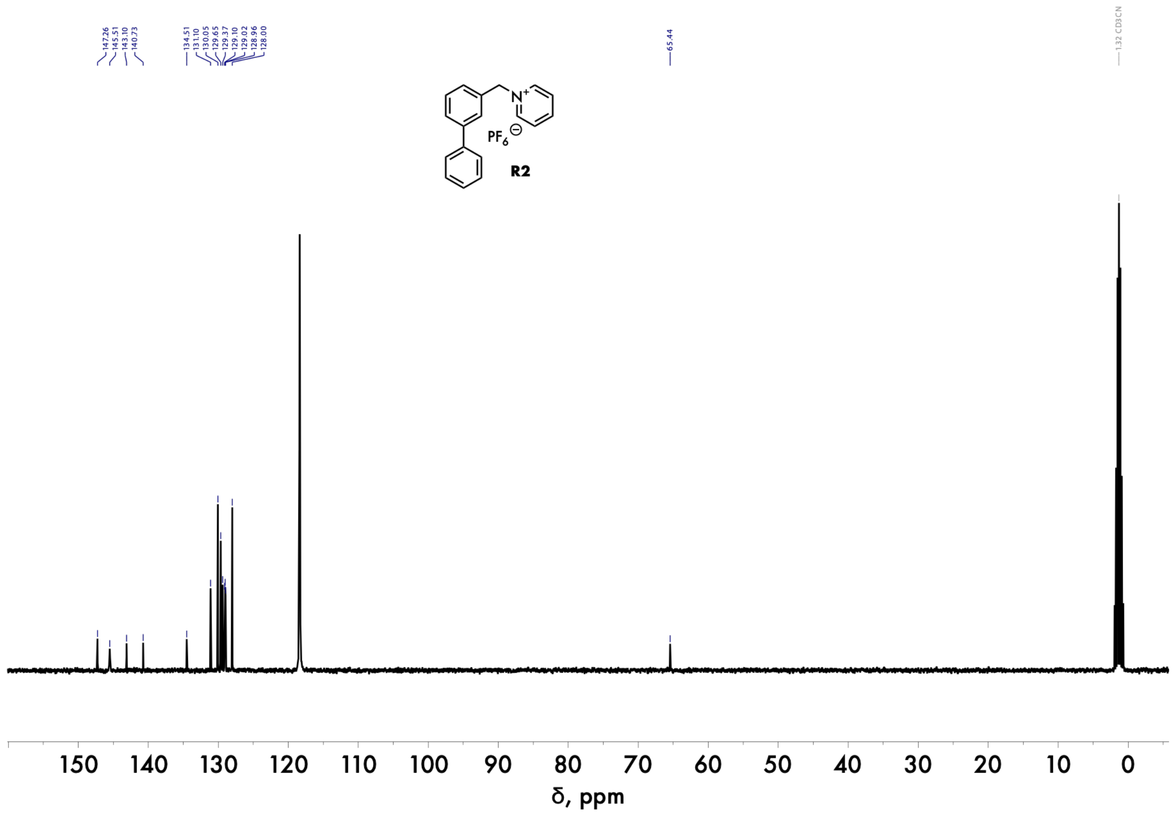

Figure S263. ^{13}C NMR (101 MHz, $CDCl_3$) spectrum of **R2**.

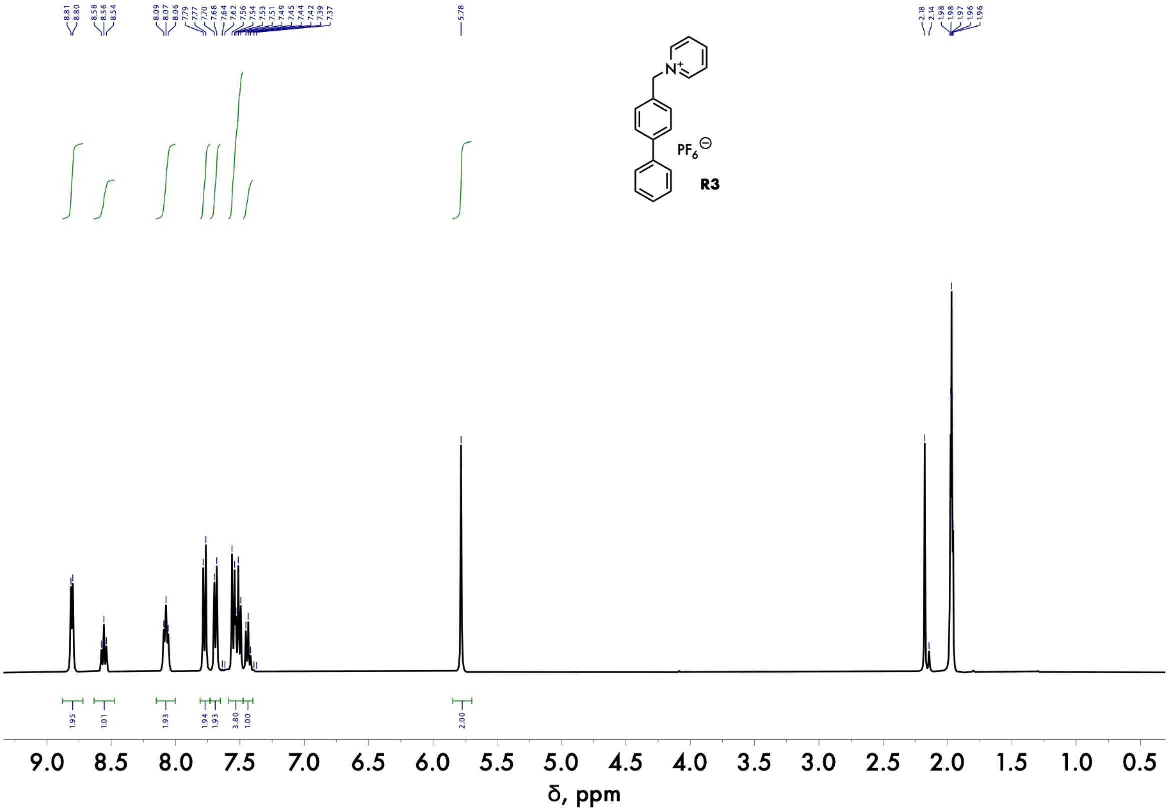

Figure S264. ^{1}H NMR (400 MHz, $CDCl_3$) spectrum of **R3**.

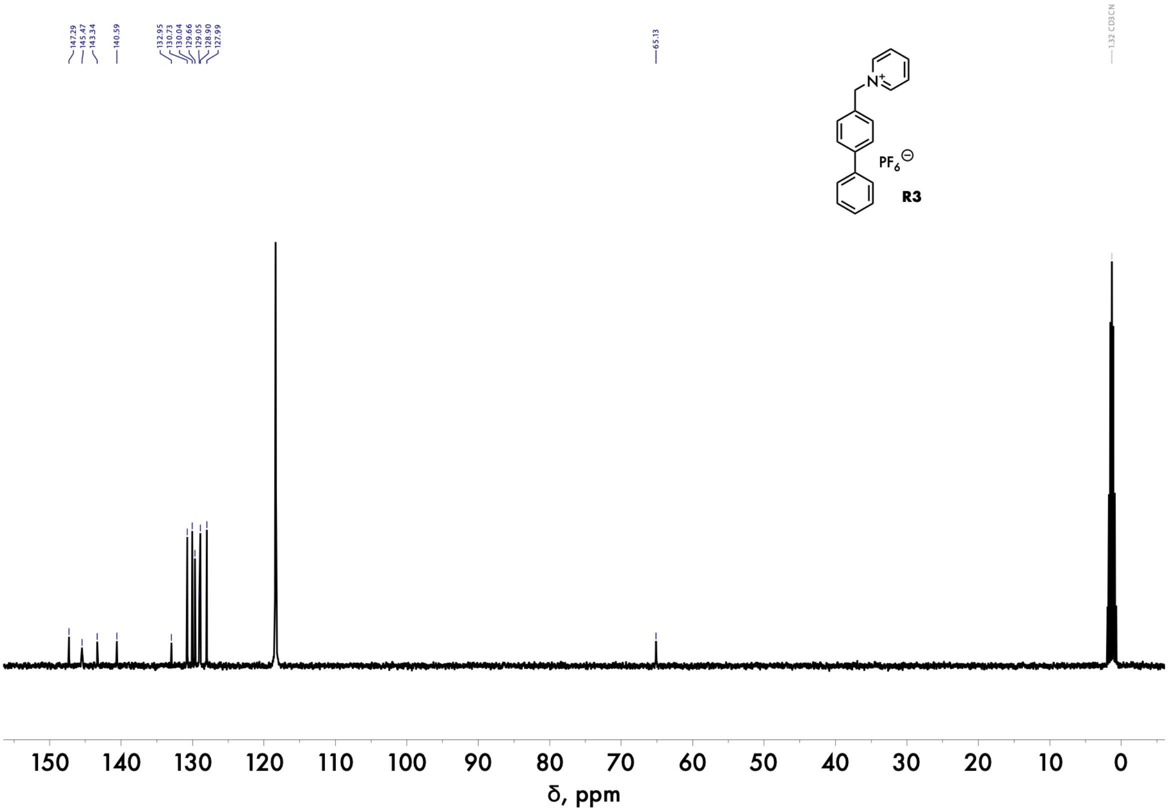

Figure S265. ^{13}C NMR (101 MHz, $CDCl_3$) spectrum of **R3**.

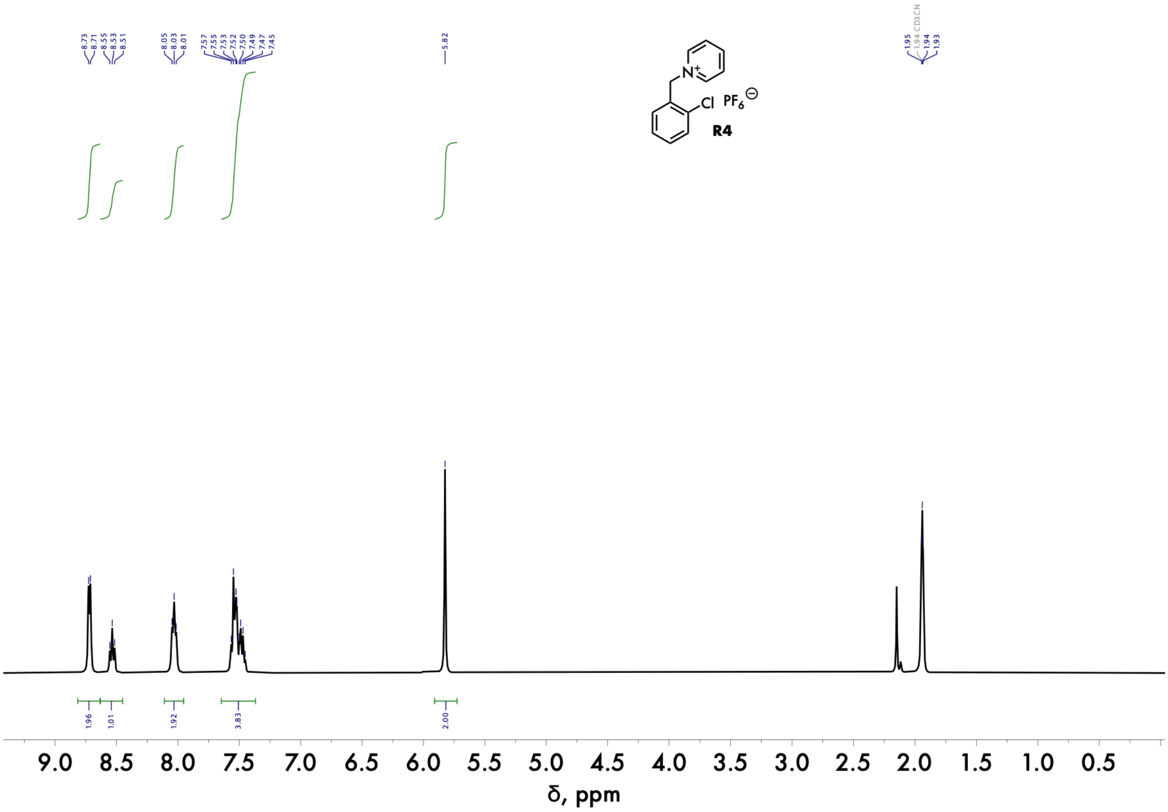

Figure S266. ^{1}H NMR (400 MHz, $CDCl_3$) spectrum of **R4**.

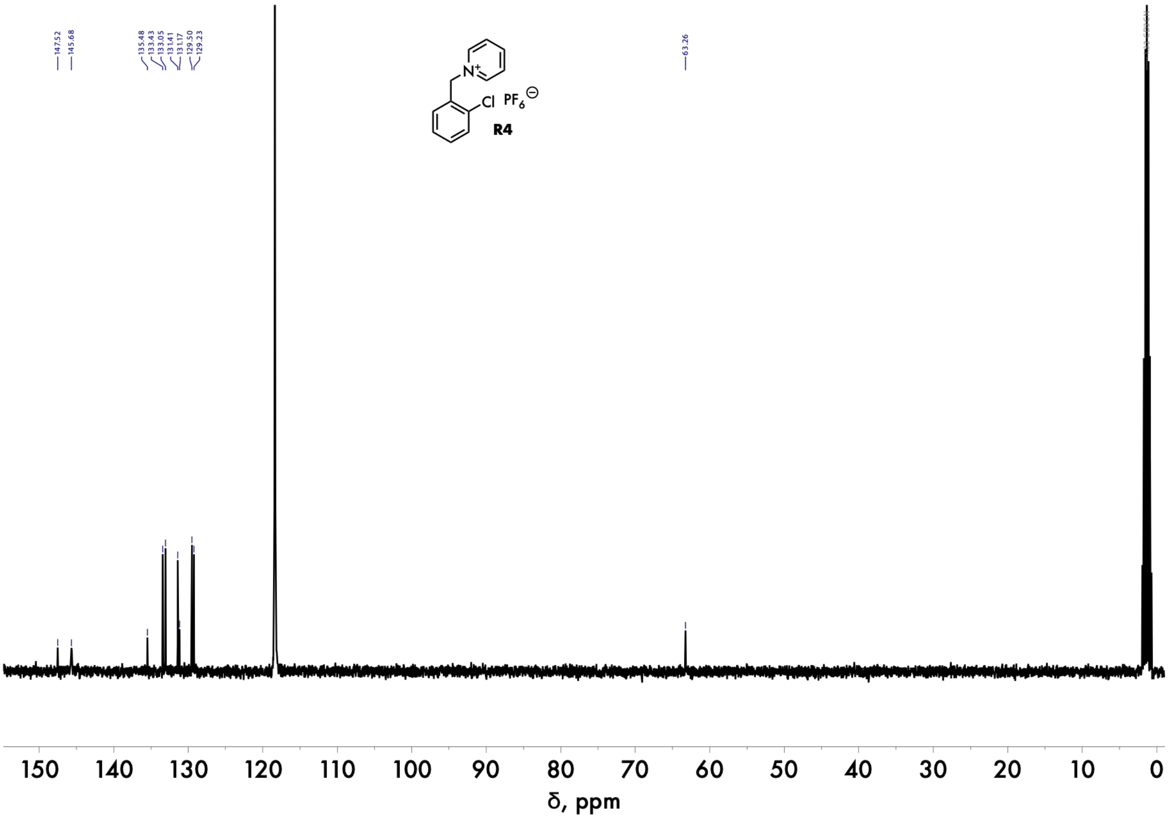

Figure S267. ^{13}C NMR (101 MHz, $CDCl_3$) spectrum of **R4**.

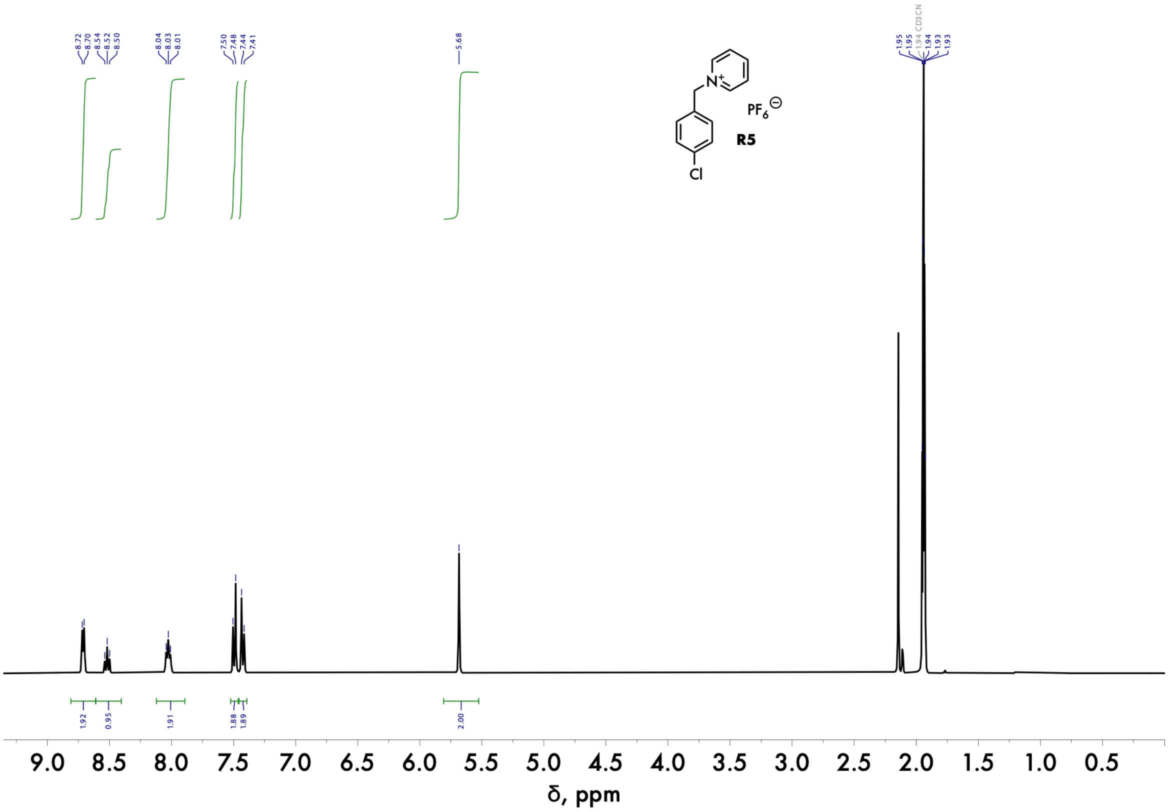

Figure S268. ^{1}H NMR (400 MHz, $CDCl_3$) spectrum of **R5**.

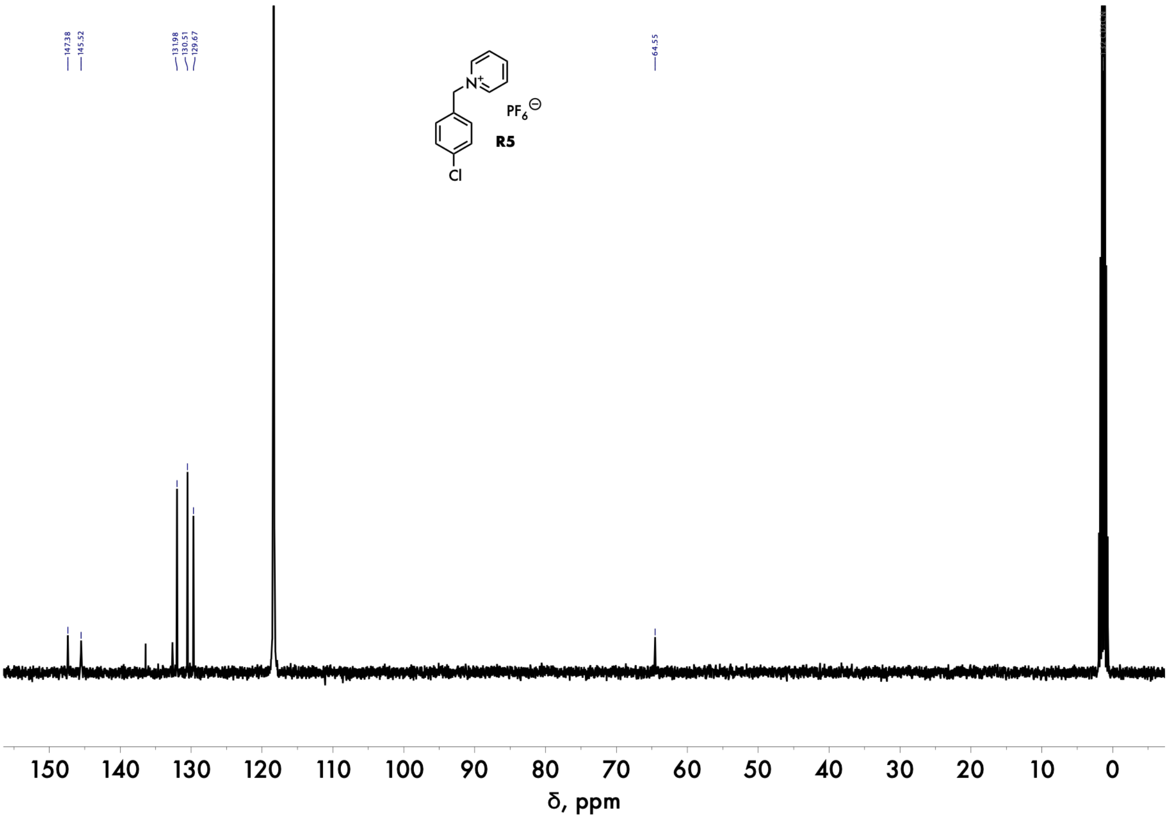

Figure S269. ^{13}C NMR (101 MHz, $CDCl_3$) spectrum of **R5**.

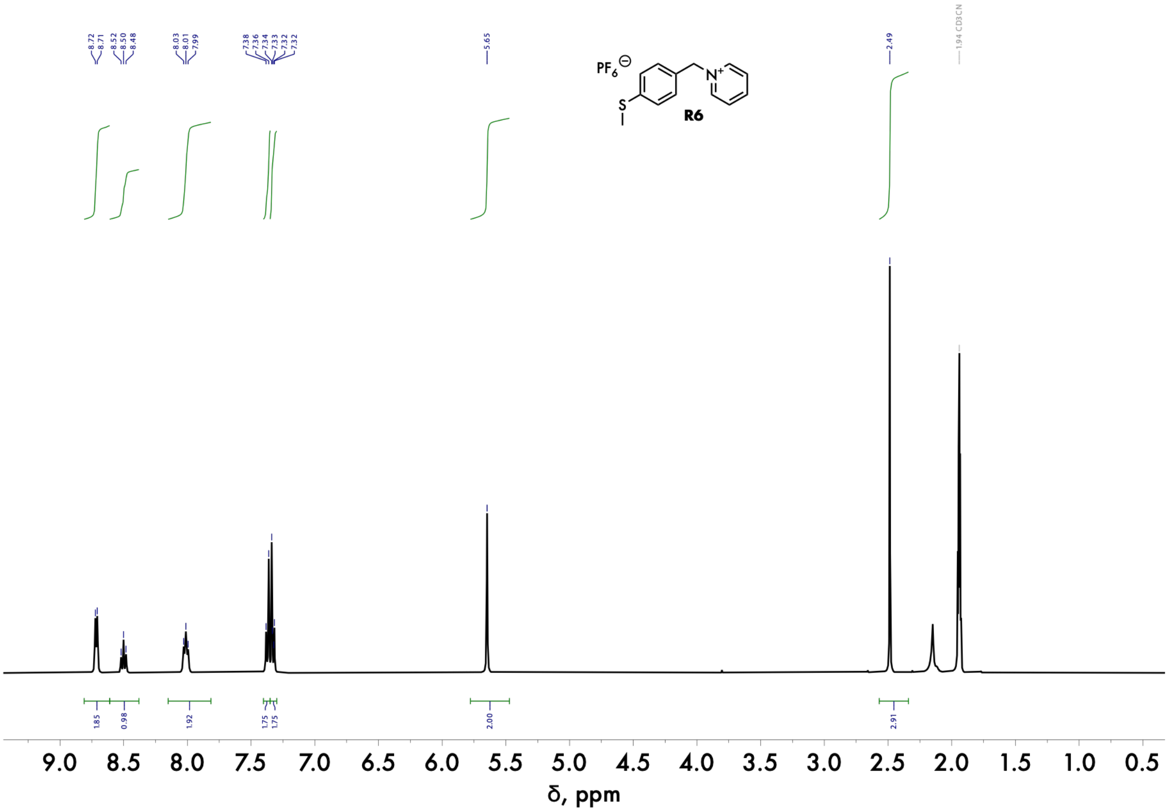

Figure S270. ^{1}H NMR (400 MHz, $CDCl_3$) spectrum of **R6**.

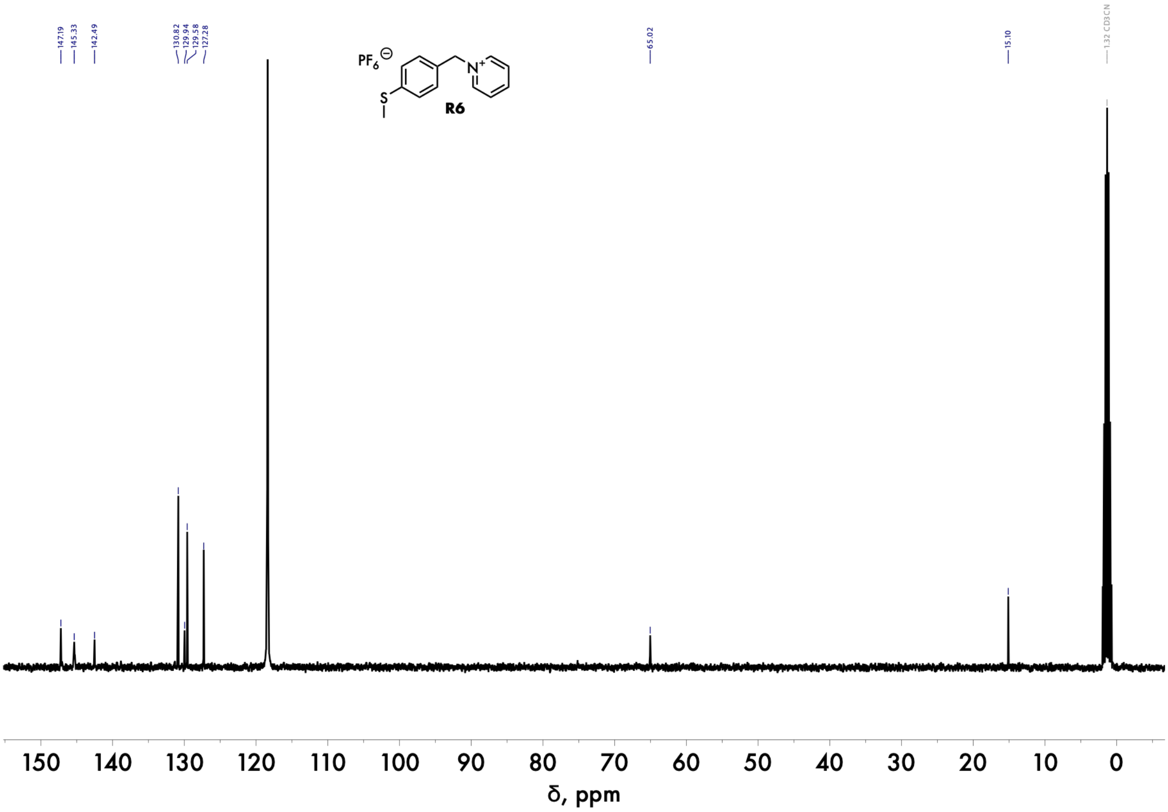

Figure S271. ^{13}C NMR (101 MHz, $CDCl_3$) spectrum of **R6**.

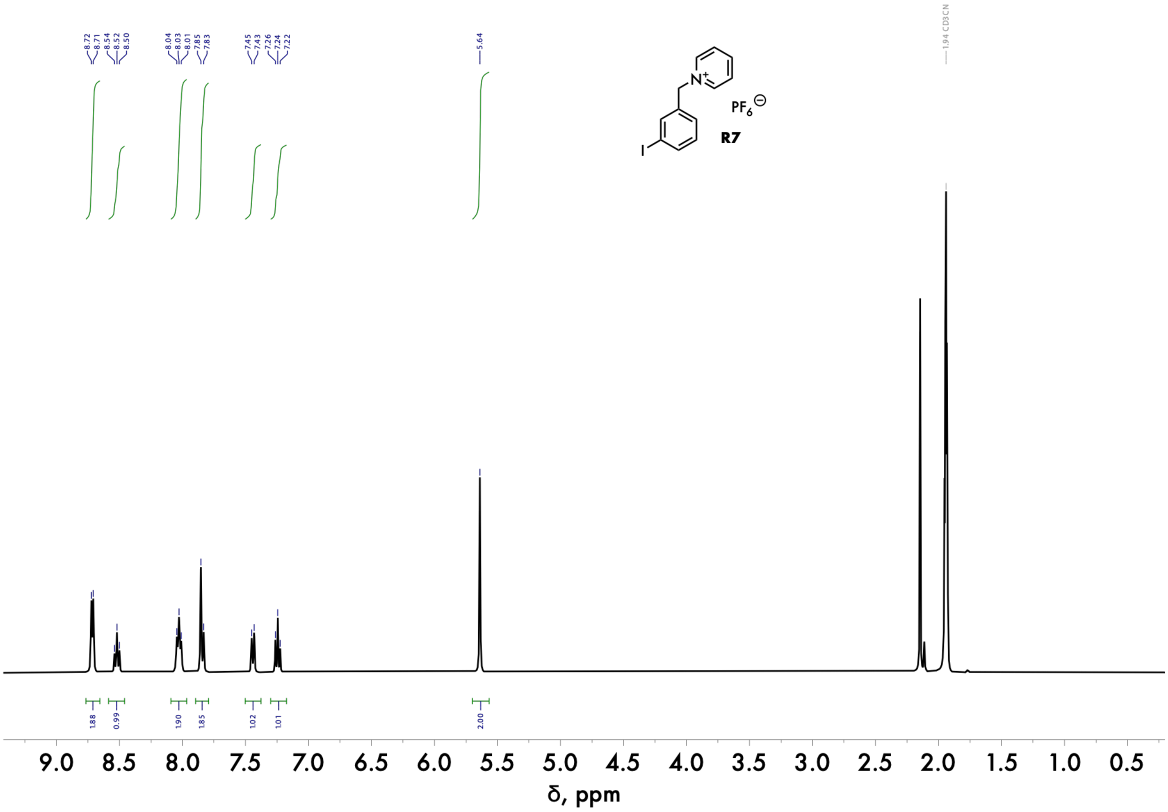

Figure S272. ^{1}H NMR (400 MHz, $CDCl_3$) spectrum of **R7**.

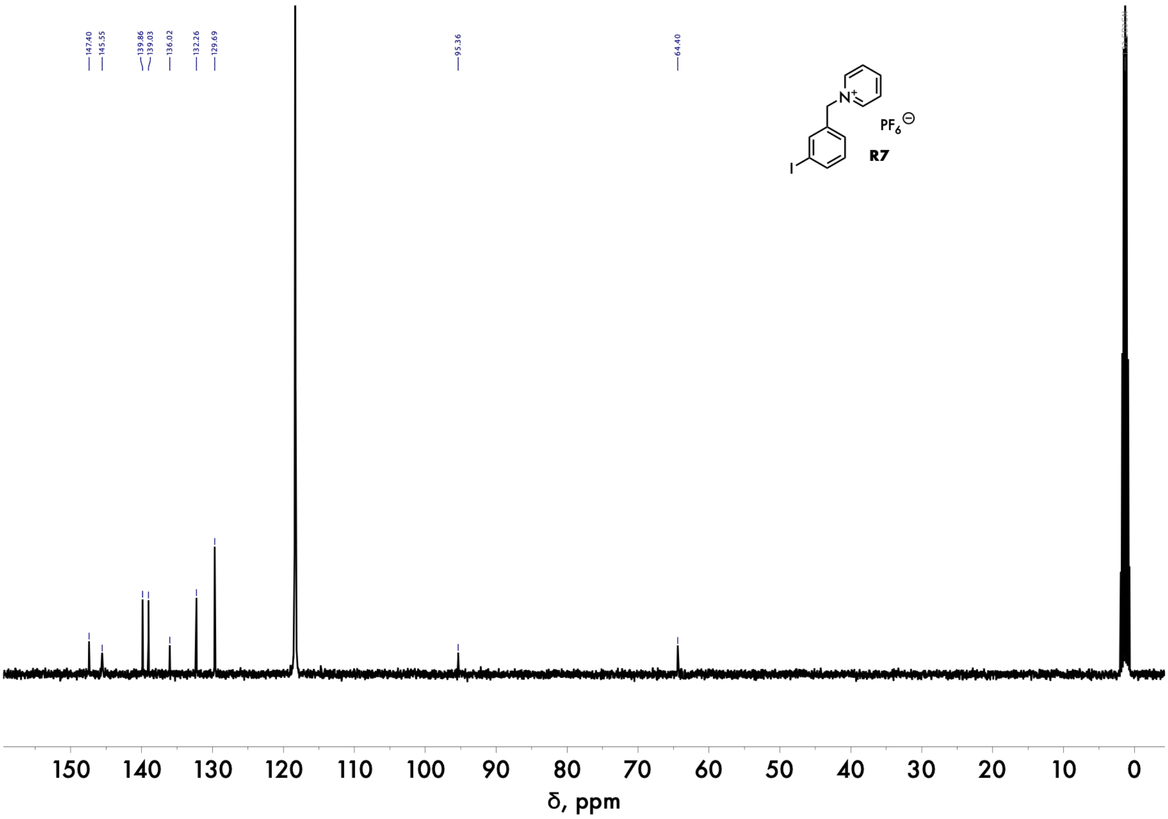

Figure S273. ^{13}C NMR (101 MHz, $CDCl_3$) spectrum of **R7**.

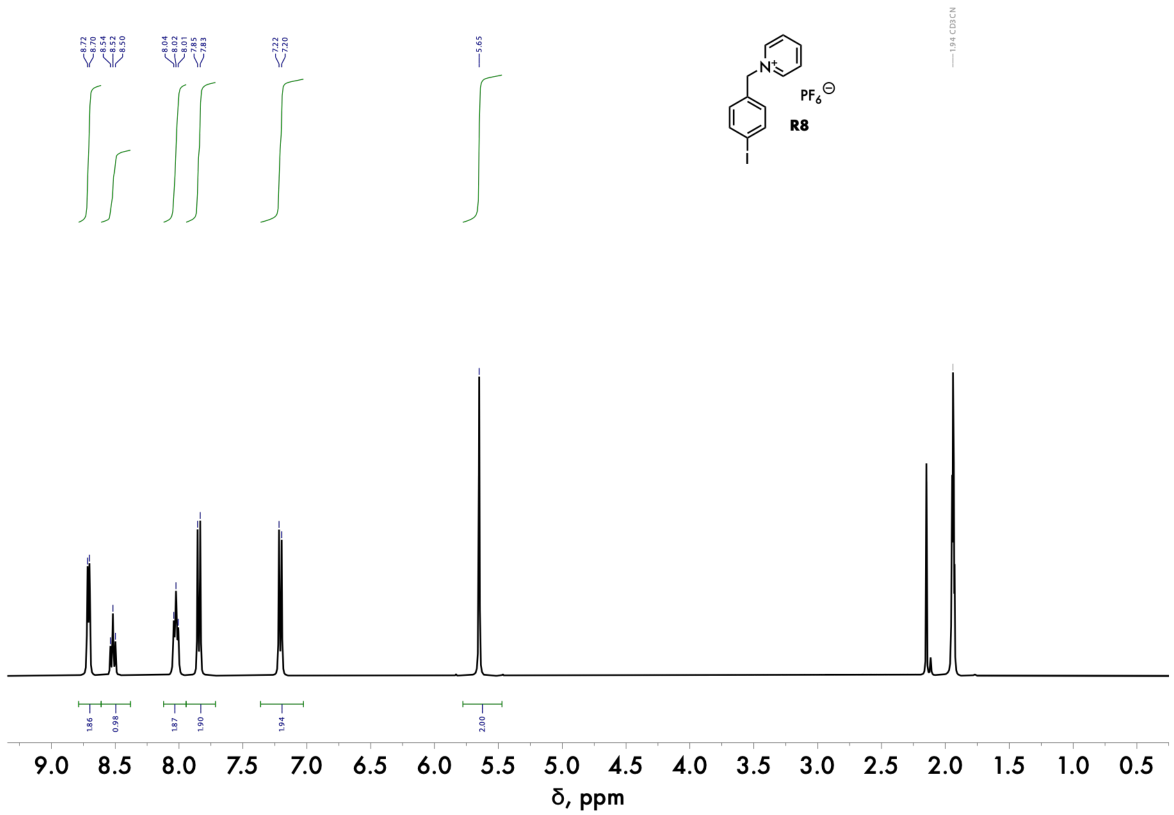

Figure S274. ^{1}H NMR (400 MHz, $CDCl_3$) spectrum of **R8.**

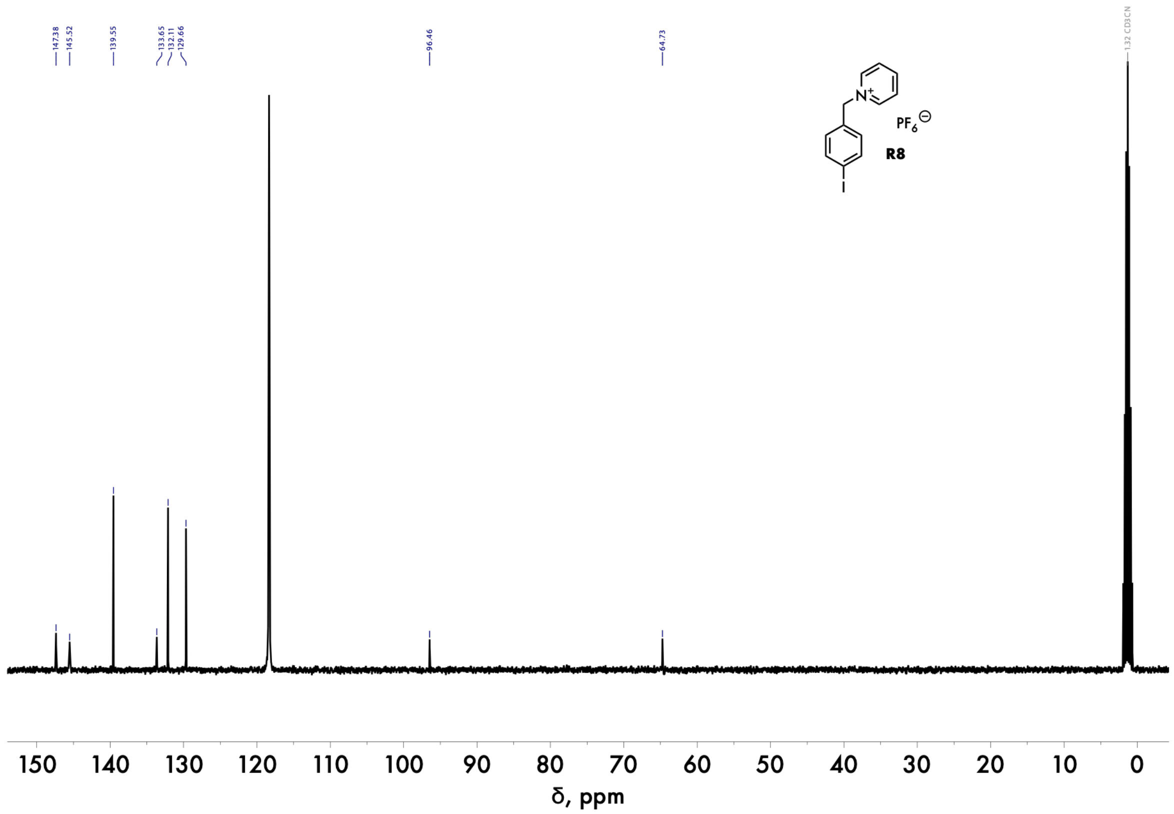

Figure S275. ^{13}C NMR (101 MHz, $CDCl_3$) spectrum of **R8**.

References

S1. Gu, C.; Wang, Z.; Shi, R. Nickel Catalyzed Three-Component Sulfonylation of Non-Activated Alkyl Chlorides. *Chem. Commun.* **2023**, *59* (45), 6889–6892. https://doi.org/10.1039/D3CC01447A.

S2. Yurino, T.; Hachiya, A.; Suzuki, K.; Ohkuma, T. Selective Conversion of Benzylic Phosphates into Diarylmethanes Through $Al(OTf)_3$-Catalyzed Friedel–Crafts-Type Benzylation. *Eur. J. Org. Chem.* **2020**, *2020* (15), 2225–2232. https://doi.org/10.1002/ejoc.202000077.

S3. Bering, L.; Jeyakumar, K.; Antonchick, A. P. Metal-Free C–O Bond Functionalization: Catalytic Intramolecular and Intermolecular Benzylation of Arenes. *Org. Lett.* **2018**, *20*, 3911–3914. https://doi.org/10.1021/acs.orglett.8b01495.

S4. Li, C.; Wang, J. Lewis Acid Catalyzed Propargylation of Arenes with O-Propargyl Trichloroacetimidates: Synthesis of 1,3-Diarylpropynes. *J. Org. Chem.* **2007**, *72* (19), 7431–7434. https://doi.org/10.1021/jo0709192.

S5. Sudmeijer, O.; Wilson, A. E.; Hays, G. R. Calculation of 13C NMR Chemical Shifts for Aromatic Carbons in Polyalkylated Benzenes. *Org. Magn. Res.* **1984**, *22* (7), 459–463. https://doi.org/10.1002/mrc.1270220714.

S6. Rabten, W.; Margarita, C.; Eriksson, L.; Andersson, P. G. Ir-Catalyzed Asymmetric and Regioselective Hydrogenation of Cyclic Allylsilanes and Generation of Quaternary Stereocenters via the Hosomi-Sakurai Allylation. *Chem. Eur. J.* **2018**, *24* (7), 1681–1685. https://doi.org/10.1002/chem.201704684.

S7. Fu, Z.; Zeng, J.; Xiong, C.; Guo, S.; Cai, H. Electrooxidative Ni-Catalyzed Decarboxylation of Arylacetic Acids Towards the Synthesis of Carbonyls under Air Conditions. *Chem. Eur. J.* **2024**, *30* (69), e202403077. https://doi.org/10.1002/chem.202403077.

S8. Zimmer, H.; Amer, A.; Baumann, F. M.; Haecker, M.; Hess, C. G. M.; Ho, D.; Huber, H. J.; Koch, K.; Mahnke, K.; Schumacher, C.; Wingfield, R. C. Synthesis of 8-Substituted Xanthines and Their Oxidative Skeleton Rearrangement to 1-Oxo-2,4,7,9-Tetraazaspiro[4,5]Dec-2-Ene-6,8,10-Triones. *Eur. J. Org. Chem.* **1999**, *1999* (9), 2419–2428. https://doi.org/10.1002/(SICI)1099-0690(199909)1999:9<2419::AID-EJOC2419>3.0.CO;2-1.

S9. Liu, F.; Li, P. Visible-Light-Promoted (Phenylsulfonyl)Methylation of Electron-Rich Heteroarenes and N-Arylacrylamides. *J. Org. Chem.* **2016**, *81* (16), 6972–6979. https://doi.org/10.1021/acs.joc.6b00689.
